# Supplementary material for: Asymmetric 1,4-functionalization of 1,3-enynes via dual photoredox and chromium catalysis
Source: Nat Commun. 2022 Aug 26;13:5036. doi: 10.1038/s41467-022-32614-4 (PMC9418150; doi:10.1038/s41467-022-32614-4)
Supplement: Supplementary file 1 — Supplementary information [file 41467_2022_32614_MOESM1_ESM.pdf]

Supplementary Information:

Asymmetric 1,4-Functionalization of 1,3-Enynes via Dual Photoredox and Chromium Catalysis

Authors: Feng-Hua Zhang[1,2,3], Xiaochong, Guo[1,2,3], Xianrong Zeng[1,2], Zhaobin Wang[1,2*]

Affiliations: [1] Key Laboratory of Precise Synthesis of Functional Molecules of Zhejiang Province, School of Science, Westlake University, Hangzhou 310024, Zhejiang Province, China;

[2] Institute of Natural Sciences, Westlake Institute for Advanced Study, Hangzhou 310024, Zhejiang Province, China;

[3] These authors contributed equally to this work

Table of Contents

I. Supplementary Methods S2

1.1 General Information S2

1.2 Preparation of Starting Materials S3

1.3 Asymmetric Radical 1,4-functionalization of 1,3-Enynes S6

1.4 Other Radical Precursors and Synthetic Transformations S44

1.5 Effect of Reaction Parameters S50

1.6 Preliminary Mechanistic Study S52

1.7 Assignment of the Absolute Configuration S57

1.8 NMR Spectra S60

1.9 Stereoselectivity Analysis S138

II Supplementary References S205

I. Supplementary Methods

1.1 General Information

Unless otherwise noted, reagents were used as received from Sigma-Aldrich, Alfa, TCI, Energy Chemical, J&K. The aldehydes used for the allenylation reaction had been purified by reduced pressure distillation or recrystallization. All reactions were performed under an atmosphere of dry nitrogen gas. Anhydrous THF was purchased from J&K and stored under nitrogen gas. Other solvents were purified with activated aluminum oxide using a solvent-purification system.

NMR spectra were recorded on a Bruker spectrometer with a Prodigy broadband cryoprobe (500 MHz or 600 MHz for ^{1}H and 126 MHz or 151 MHz for ^{13}C); chemical shifts (δ) are reported in ppm downfield from tertramethylsilane, using the solvent resonance as the internal standard. Optical rotation data were obtained with a Jasco P-2000 polarimeter at 589 nm, using a 50 mm path-length cell in the solvent and at the concentration indicated. High resolution mass spectrometric analysis was performed on ultra-performance liquid chromatography-time-of-flight mass spectrometer (Synapt-G2-Si, Waters, USA) with electron spray ionization (ESI) resource and atmosphere pressure chemical ionization (APCI) resource. SFC analysis was carried out on an Agilent 1260 series system with Daicel CHIRALPAK® or Daicel CHIRALCEL® columns (internal diameter 4.6 mm, column length 250 mm, particle size 5 μm). HPLC analysis was carried out on Waters Arc HPLC system with Daicel CHIRALPAK® or Daicel CHIRALCEL® columns (internal diameter 4.6 mm, column length 250 mm, particle size 5 μm). The visible lights (160-440 nm LED, 20 W) were purchased from Kessil (website: http://kessil.com/science/PR160L.php).

1.2 Preparation of Starting Materials

Ligand Preparation: The ligands (*R*,*S*)-**L1** and (*S*,*R*)-**L1** were prepared according to a reported literature procedure, and all the analytical data matched the report (*1*).

DHP Esters Preparation:

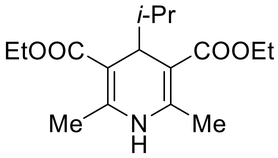

2

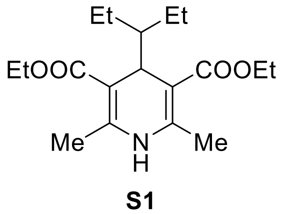

S1

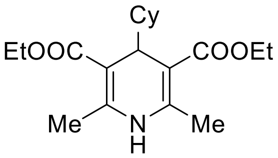

S2

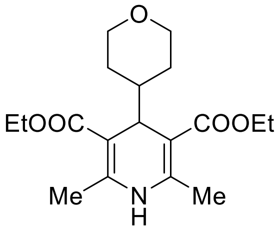

S3

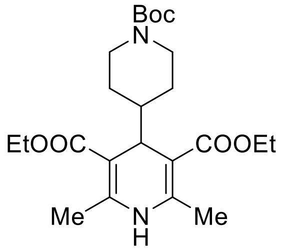

S4

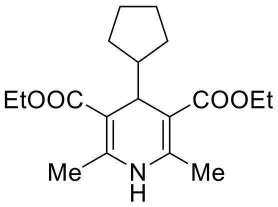

S5

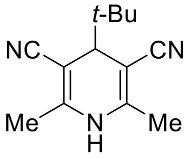

S6

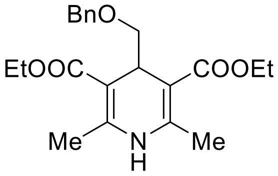

S7

The above DHP esters were prepared according to reported literature procedures, and all the analytical data matched the reports (*2*).

General Procedure 1 (GP-1): Preparation of DHP esters

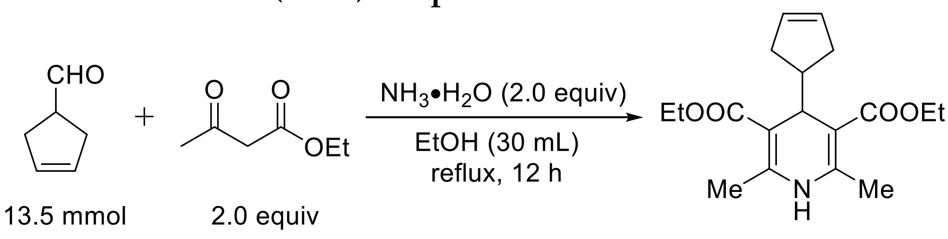

S8

The reaction flask was charged with ethyl acetoacetate (3.5 mL, 27 mmol), cyclopent-3-ene-1-carbaldehyde (1.3 g, 13.5 mmol), and ethanol (30 mL). To the above solution, ammonium hydroxide (1.1 mL, 27 mmol) was added slowly. Then the system was heated to reflux with stirring. After completion of the reaction, as determined by TLC, the solution was cooled, concentrated, and

purified by silica-gel column chromatography (PE/EtOAc) and followed by recrystallization (EtOH/PE) to afford the desired product **S8** as a white solid.

^{1}H NMR (500 MHz, $CDCl_3$) δ 5.68 – 5.59 (m, 3H), 4.26 – 4.08 (m, 5H), 2.30 (s, 6H), 2.24 – 2.05 (m,5H), 1.30 (t, J = 7.1 Hz, 6H).

^{13}C NMR (126 MHz, $CDCl_3$) δ 168.4, 144.6, 130.3, 102.8, 59.6, 46.3, 35.9, 34.5, 19.5, 14.4.

HRMS (ESI) m/z $[M + H]^+$ calcd for $C_{18}H_{26}NO_4$: 320.1862, found: 320.1824.

1,3-Enynes Preparation:

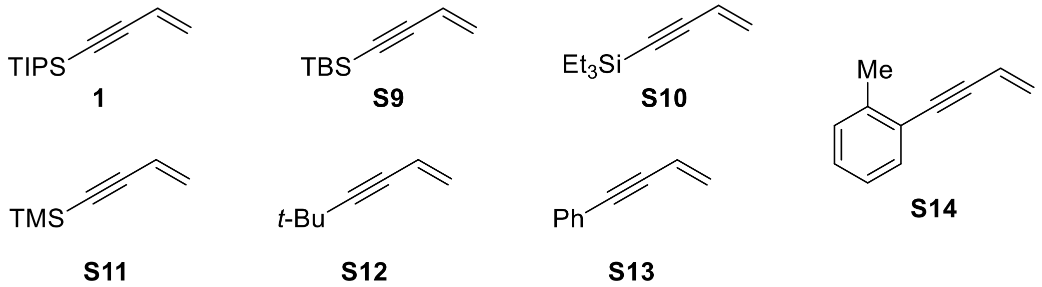

The above 1,3-enynes were prepared according to reported literature procedures, and all the analytical data matched the reports (*3*).

General Procedure 2 (GP-2): Preparation of 1,3-enynes

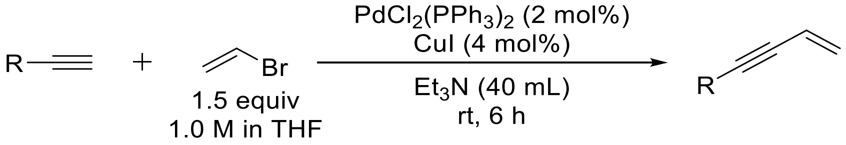

To an oven dried Schlenk flask equipped with a magnetic stir bar was successively added $PdCl_2(PPh_3)_2$ (0.8 mmol, 562 mg.) and CuI (1.6 mmol, 305 mg). The system was purged with nitrogen. Dry Et_3N (40 mL) was then added followed by the corresponding alkyne (40 mmol, 1.0 equiv.), vinyl bromide (60 mmol, 1.0 M in THF, 60 mL) via syringe. The reaction mixture was stirred at room temperature for 6 h. After evaporation under vacuum, the residue was purified by flash chromatography over silica gel and distilled under reduced pressure to give the desired 1,3-enynes.

All the yields have not been optimized.

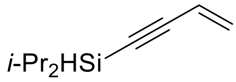

S15

but-3-en-1-yn-1-yldiisopropylsilane (S15). The title compound was prepared according to the **GP-2**, using ethynyldiisopropylsilane, purified by flash column chromatography: 100% hexanes, 85% yield, colorless liquid.

^{1}H NMR (500 MHz, $CDCl_3$) δ 5.83 (ddd, J = 17.6, 11.1, 0.9 Hz, 1H), 5.71 (dd,

J = 17.6, 2.3 Hz, 1H), 5.53 (dd, J = 11.1, 2.3 Hz, 1H), 3.76 (s, 1H), 1.13 – 0.99 (m, 14H).

^{13}C NMR (126 MHz, $CDCl_3$) δ 128.3, 117.2, 106.4, 88.8, 18.5, 18.2, 10.8.

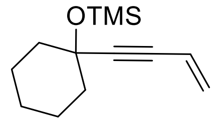

S16

((1-(but-3-en-1-yn-1-yl)cyclohexyl)oxy)trimethylsilane (S16). The title compound was prepared according to the **GP-2**, using ((1-ethynylcyclohexyl)oxy)trimethylsilane, purified by flash column chromatography: 100% hexanes, 76% yield, colorless liquid.

^{1}H NMR (500 MHz, $CDCl_3$) δ 5.84 (dd, J = 17.6, 11.1 Hz, 1H), 5.60 (dd, J = 17.6, 2.1 Hz, 1H), 5.45 (dd, J = 11.1, 2.1 Hz, 1H), 1.86 – 1.84 (m, 2H), 1.69 – 1.42 (m, 8H), 0.19 (s, 9H).

^{13}C NMR (126 MHz, $CDCl_3$) δ 126.3, 117.1, 94.1, 84.0, 70.2, 41.2, 25.3, 23.1, 1.9.

HRMS (APCI) m/z $[M + H]^+$ calcd for $C_{13}H_{23}SiO$: 223.1518, found: 223.1517.

NHPI Esters Preparation:

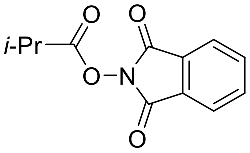

S17

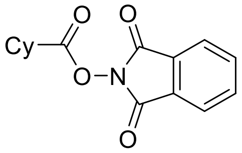

S18

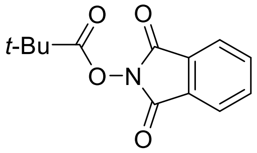

S19

The above NHPI esters were prepared according to reported literature procedures, and all the analytical data matched the reports (*4*).

1.3 Asymmetric Radical 1,4-functionalization of 1,3-Enynes

General procedure 3 (GP-3): Asymmetric 1,4-functionalization of 1,3-enynes with DHP esters.

Preparation of the catalyst solution: In a nitrogen-filled glovebox, and oven-dried 20 mL vial with a magnetic stir bar, were charged the $CrCl_2$ (5.0 mg, 0.04 mmol, 10 mol%) and (*S*,*R*)-**L1** (23 mg, 0.048 mmol, 12 mol%). Then 8.0 mL THF was added and the vial was closed with a PTFE septum cap, and then stirred at room temperature for 2 h.

Catalytic asymmetric radical 1,4-functionalization of 1,3-enynes: In a nitrogen-filled glovebox, to the prepared catalyst solution were added the 1,3-enynes (0.6 mmol, 1.5 equiv), the aldehydes (0.4 mmol, 1.0 equiv), the DHP esters (0.6 mmol, 1.5 equiv), and 4-CzIPN (6.4 mg, 0.008 mmol, 2 mol%) sequentially. Then the vial was closed with a PTFE septum cap, and taken out of the glovebox. Then, the reaction was irradiated with two 20 W 160-440 nm LED for 12 hours (tube 5 cm away from lights, fans for cooling, 30-35 °C). After that, the reaction mixture was concentrated and run through a short silica gel pad with hexanes/EtOAc (3:1) as the eluent. Then the solvent was removed under the reduced pressure. The diastereoselectivity was determined via ^{1}H NMR analysis of the crude reaction mixture. and the residue was purified by flash chromatography to provide the desired product and the ee was determined via HPLC analysis.

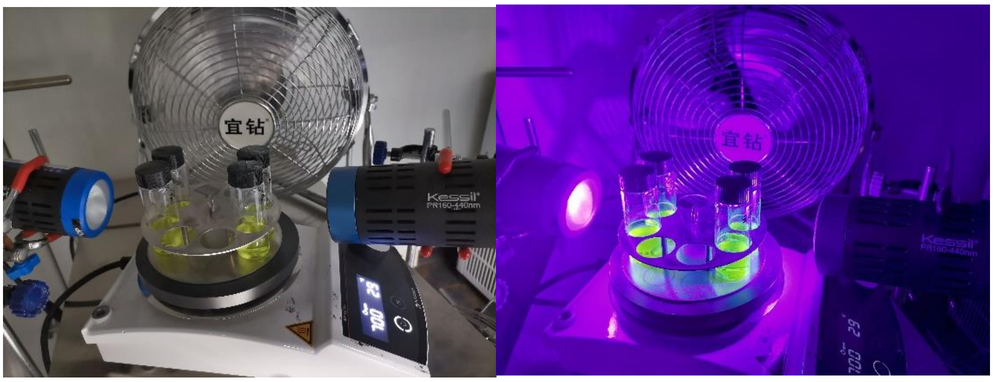

Supplementary Figure 1. Reaction Set-up

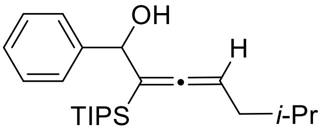

6-methyl-1-phenyl-2-(triisopropylsilyl)hepta-2,3-dien-1-ol (Figure 3, entry 3). The title compound was prepared according to the **GP-3** from benzaldehyde, enyne **1** and DHP ester **2**, purified by flash column chromatography on silica gel: 5→10% EtOAc in hexanes, colorless liquid.

(*R,S*)-**L1**: 131 mg, 92% yield, 20:1 dr, 95% ee; (*S,R*)-**L1**: 131 mg, 92% yield, 20:1 dr, 94% ee.

HPLC analysis: The ee was determined on a CHIRALCEL OD-3 column (5% *i*-PrOH in hexane, 1.0 mL/min); retention times for compound obtained using (*R,S*)-**L1**: 3.6 min (major), 4.4 min (minor).

^{1}H NMR (500 MHz, $CDCl_3$) δ 7.39 – 7.34 (m, 2H), 7.31 (dd, *J* = 10.2, 4.8 Hz, 2H), 7.24 (ddd, *J* = 6.3, 4.4, 1.3 Hz, 1H), 5.13 (s, 1H), 5.07 (td, *J* = 7.6, 2.1 Hz, 1H), 2.32 (brs, 1H), 1.99 – 1.86 (m, 2H), 1.57 (dp, *J* = 13.4, 6.7 Hz, 1H), 1.17 – 1.08 (m, 3H), 1.06 (d, *J* = 7.2 Hz, 9H), 0.93 (d, *J* = 7.3 Hz, 9H), 0.91 – 0.85 (m, 6H).

^{13}C NMR (126 MHz, $CDCl_3$) δ 206.6, 143.6, 128.2, 127.6, 127.2, 98.0, 88.9, 72.7, 38.0, 29.0, 22.3, 22.2, 18.7, 18.4, 11.6.

HRMS (ESI) m/z $[M – H_2O + H]^+$ calcd for $C_{23}H_{37}Si$: 341.2664, found: 341.2650.

$[\alpha]^{24}_{D}$ = –181.2 (*c* = 0.5, $CHCl_3$); 95% ee, from (*R,S*)-**L1**.

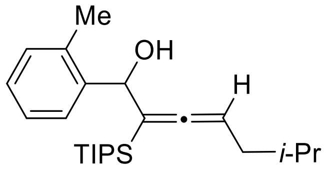

6-methyl-1-(*o*-tolyl)-2-(triisopropylsilyl)hepta-2,3-dien-1-ol (Figure 3, entry 4). The title compound was prepared according to the **GP-3** from 2-methylbenzaldehyde, enyne **1** and DHP ester **2**, purified by flash column chromatography on silica gel: 5→10% EtOAc in hexanes, colorless liquid.

(*R,S*)-**L1**: 141 mg, 95% yield, > 20:1 dr, 96% ee; (*S,R*)-**L1**: 142 mg, 96% yield, > 20:1 dr, 97% ee.

HPLC analysis: The ee was determined on a CHIRALCEL OD-3 column (3% *i*-PrOH in hexane, 1.0 mL/min); retention times for compound obtained using (*R,S*)-**L1**: 5.1 min (major), 5.5 min (minor).

^{1}H NMR (500 MHz, $CDCl_3$) δ 7.44 (dd, *J* = 7.5, 1.2 Hz, 1H), 7.18 (td, *J* = 7.4, 1.5 Hz, 1H), 7.13 (ddd, *J* = 13.6, 9.8, 4.5 Hz, 2H), 5.41 (s, 1H), 4.93 (ddd, *J* = 8.0, 7.2, 2.5 Hz, 1H), 2.39 (s, 3H), 2.08 (d, *J* = 5.3 Hz, 1H), 1.92 (dt, *J* = 13.8, 6.9 Hz, 1H), 1.83 (ddd, *J* = 13.9, 8.1, 7.0 Hz, 1H), 1.48 (dt, *J* = 13.4, 6.7 Hz, 1H), 1.21 – 1.11 (m, 3H), 1.08 (d, *J* = 7.3 Hz, 9H), 0.99 (d, *J* = 7.3 Hz, 9H), 0.82 (dd, *J* = 16.9, 6.6 Hz, 6H).

^{13}C NMR (126 MHz, $CDCl_3$) δ 207.2, 141.2, 135.6, 130.4, 127.4, 126.8, 125.8, 96.8, 88.2, 69.9, 37.8, 29.0, 22.3, 22.1, 19.3, 18.7, 18.5, 11.8.

HRMS (ESI) m/z $[M – H_2O + H]^+$ calcd for $C_{24}H_{39}Si$: 355.2821, found: 355.2830

$[\alpha]^{24}_{D}$ = –133.2 (*c* = 0.5, $CHCl_3$); 96% ee, from (*R,S*)-**L1**.

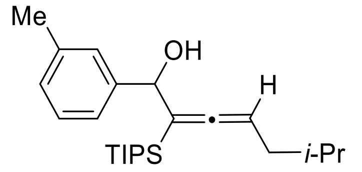

6-methyl-1-(*m*-tolyl)-2-(triisopropylsilyl)hepta-2,3-dien-1-ol (Figure 3, entry 5). The title compound was prepared according to the **GP-3** from 3-methylbenzaldehyde, enyne **1** and DHP ester **2**, purified by flash column chromatography on silica gel: 5→10% EtOAc in hexanes, colorless liquid.

(*R,S*)-**L1**: 128 mg, 86% yield, 13:1 dr, 93% ee; (*S,R*)-**L1**: 130 mg, 88% yield, 13:1 dr, 93% ee.

HPLC analysis: The ee was determined on a CHIRALCEL OD-3 column (3% *i*-PrOH in hexane, 1.0 mL/min); retention times for compound obtained using (*R,S*)-**L1**: 3.6 min (major), 4.3 min (minor).

^{1}H NMR (500 MHz, $CDCl_3$) δ 7.22 – 7.14 (m, 3H), 7.07 – 7.03 (m, 1H), 5.10 – 5.07 (m, 2H), 2.34 (s, 3H), 2.29 (d, *J* = 5.3 Hz, 1H), 2.00 – 1.88 (m, 2H), 1.64 – 1.56 (m, 1H), 1.17 – 1.09 (m, 3H), 1.06 (d, *J* = 7.1 Hz, 9H), 0.93 (d, *J* = 7.2 Hz, 9H), 0.89 (t, *J* = 6.6 Hz, 6H).

^{13}C NMR (126 MHz, $CDCl_3$) δ 206.6, 143.4, 137.7, 128.3, 128.1, 128.0, 124.3, 98.0, 88.8, 72.6, 38.1, 29.0, 22.3, 22.2, 21.4, 18.7, 18.4, 11.6.

HRMS (ESI) m/z $[M – H_2O + H]^+$ calcd for $C_{24}H_{39}Si$: 355.2821, found: 355.2816.

$[\alpha]^{24}_{D}$ = –146.8 (*c* = 0.5, $CHCl_3$); 93% ee, from (*R,S*)-**L1**.

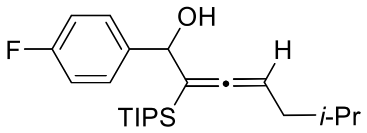

1-(4-fluorophenyl)-6-methyl-2-(triisopropylsilyl)hepta-2,3-dien-1-ol (Figure 3, entry 6). The title compound was prepared according to the **GP-3** from 4-fluorobenzaldehyde, enyne **1** and DHP ester **2**, purified by flash column chromatography on silica gel: 5→10% EtOAc in hexanes, colorless liquid.

(*R,S*)-**L1**: 142 mg, 95% yield, > 20:1 dr, 93% ee; (*S,R*)-**L1**: 142 mg, 95% yield, > 20:1 dr, 92% ee.

HPLC analysis: The ee was determined on a CHIRALCEL OD-3 column (3% *i*-PrOH in hexane, 1.0 mL/min); retention times for compound obtained using (*R,S*)-**L1**: 3.7 min (major), 4.4 min (minor).

^{1}H NMR (500 MHz, $CDCl_3$) δ 7.37 – 7.31 (m, 2H), 7.02 – 6.98 (m, 2H), 5.13 (s, 1H), 5.07 (td, *J* = 7.6, 2.1 Hz, 1H), 2.31 (s, 1H), 2.00 – 1.83 (m, 2H), 1.58 (dp, *J* = 13.3, 6.7 Hz, 1H), 1.18 – 1.09 (m, 3H), 1.06 (d, *J* = 7.0 Hz, 9H), 0.95 (d, *J* = 7.2 Hz, 9H), 0.88 (dd, *J* = 8.2, 6.7 Hz, 6H).

^{13}C NMR (126 MHz, $CDCl_3$) δ 206.7 (s), 162.2 (d, *J* = 245.5 Hz), 139.5 (d, *J* = 3.1 Hz), 128.8 (d, *J* = 8.1 Hz), 114.9 (d, *J* = 21.4 Hz), 98.1 (s), 89.0 (s), 72.0 (s), 38.0 (s), 29.0 (s), 22.3 (s), 22.2 (s), 18.6 (s), 18.4 (s), 11.6 (s).

^{19}F NMR (471 MHz, $CDCl_3$) δ -115.16.

HRMS (ESI) m/z [M – H_2O + H]$^+$ calcd for $C_{23}H_{36}SiF$: 359.2570, found: 359.2566.

$[\alpha]^{24}_D$ = –187.2 (*c* = 0.5, $CHCl_3$); 93% ee, from (*R,S*)-**L1**.

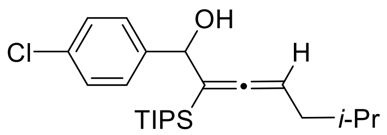

1-(4-chlorophenyl)-6-methyl-2-(triisopropylsilyl)hepta-2,3-dien-1-ol (Figure 3, entry 7). The title compound was prepared according to the **GP-3** from 4-chlorobenzaldehyde, enyne **1** and DHP ester **2**, purified by flash column chromatography on silica gel: 5→10% EtOAc in hexanes, colorless liquid.

(*R,S*)-**L1**: 122 mg, 78% yield, 15:1 dr, 91% ee; (*S,R*)-**L1**: 122 mg, 78% yield, 15:1 dr, 93% ee.

HPLC analysis: The ee was determined on a CHIRALCEL OD-3 column (5% *i*-PrOH in hexane, 1.0 mL/min); retention times for compound obtained using (*R,S*)-**L1**: 3.6 min (major), 4.2 min (minor).

^{1}H NMR (500 MHz, $CDCl_3$) δ 7.34 – 7.25 (m, 4H), 5.12 (s, 1H), 5.04 (t, *J* = 7.5 Hz, 1H), 2.28 (s, 1H), 1.96 – 1.81 (m, 2H), 1.54 (tt, *J* = 13.3, 6.7 Hz, 1H), 1.14 (dt, *J* = 14.5, 7.3 Hz, 3H), 1.06 (d, *J* = 7.5 Hz, 9H), 0.96 (d, *J* = 7.3 Hz, 9H), 0.87 (dd, *J* = 9.5, 7.6 Hz, 6H).

^{13}C NMR (126 MHz, $CDCl_3$) δ 207.1, 142.2, 133.2, 128.4, 128.3, 97.9, 89.0, 72.1, 37.9, 29.0, 22.3, 22.1, 18.6, 18.5, 11.6.

HRMS (ESI) m/z [M – H_2O + H]$^+$ calcd for $C_{23}H_{36}SiCl$: 375.2275, found: 375.2286.

$[\alpha]^{24}_D$ = –128.8 (*c* = 0.5, $CHCl_3$); 91% ee, from (*R,S*)-**L1**.

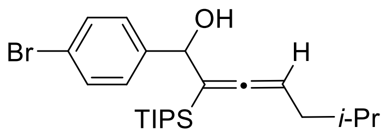

1-(4-bromophenyl)-6-methyl-2-(triisopropylsilyl)hepta-2,3-dien-1-ol (Figure 3, entry 8). The title compound was prepared according to the **GP-3** from 4-bromobenzaldehyde, enyne **1** and DHP ester **2**, purified by flash column chromatography on silica gel: 5→10% EtOAc in hexanes, colorless liquid.

(*R,S*)-**L1**: 118 mg, 68% yield, 13:1 dr, 93% ee; (*S,R*)-**L1**: 118 mg, 68% yield, 13:1 dr, 92% ee.

HPLC analysis: The ee was determined on a CHIRALCEL OD-3 column (3% *i*-PrOH in hexane, 1.0 mL/min); retention times for compound obtained using (*R,S*)-**L1**: 4.2 min (major), 5.2 min (minor).

^{1}H NMR (500 MHz, $CDCl_3$) δ 7.44 (d, *J* = 8.4 Hz, 2H), 7.25 (d, *J* = 8.5 Hz, 2H), 5.11 (s, 1H), 5.04 (td, *J* = 7.8, 1.9 Hz, 1H), 2.24 (s, 1H), 1.89 (dtd, *J* = 21.6, 14.0, 7.3 Hz, 2H), 1.58 – 1.50 (m, 1H), 1.17 – 1.09 (m, 3H), 1.06 (d, *J* = 7.1 Hz, 9H), 0.96 (d,

J = 7.2 Hz, 9H), 0.86 (dd, J = 10.7, 6.6 Hz, 6H).

^{13}C NMR (126 MHz, $CDCl_3$) δ 207.2, 142.8, 131.2, 128.8, 121.3, 97.9, 89.0, 72.1, 37.9, 29.0, 22.3, 22.1, 18.7, 18.5, 11.6.

HRMS (ESI) m/z [M – H_2O + H]$^+$ calcd for $C_{23}H_{36}SiBr$: 419.1770, found: 419.1735.

$[\alpha]^{24}_D$ = –135.2 (c = 0.5, $CHCl_3$); 93% ee, from (R,S)-**L1**.

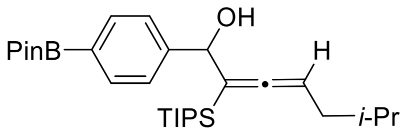

6-methyl-1-(4-(4,4,5,5-tetramethyl-1,3,2-dioxaborolan-2-yl)phenyl)-2-(triisopropylsilyl)hepta-2,3-dien-1-ol (Figure 3, entry 9). The title compound was prepared according to the **GP-3** from 4-(4,4,5,5-tetramethyl-1,3,2-dioxaborolan-2-yl)benzaldehyde, enyne **1** and DHP ester **2**, purified by flash column chromatography on silica gel: 5→10% EtOAc in hexanes, colorless liquid.

(R,S)-**L1**: 93 mg, 48% yield, 16:1 dr, 96% ee; (S,R)-**L1**: 94 mg, 49% yield, 16:1 dr, 95% ee.

HPLC analysis: The ee was determined on a CHIRALCEL OD-3 column (3% i-PrOH in hexane, 1.0 mL/min); retention times for compound obtained using (R,S)-**L1**: 3.6 min (major), 3.9 min (minor).

^{1}H NMR (500 MHz, $CDCl_3$) δ 7.75 (d, J = 8.0 Hz, 2H), 7.37 (d, J = 8.0 Hz, 2H), 5.13 (s, 1H), 5.05 (td, J = 7.7, 1.9 Hz, 1H), 2.26 (d, J = 4.8 Hz, 1H), 1.97 – 1.83 (m, 2H), 1.56 – 1.52 (m, 1H), 1.34 (s, 12H), 1.17 – 1.09 (m, 3H), 1.06 (d, J = 7.2 Hz, 9H), 0.94 (d, J = 7.3 Hz, 9H), 0.86 (dd, J = 8.0, 6.7 Hz, 6H).

^{13}C NMR (126 MHz, $CDCl_3$) δ 207.1, 146.8, 134.7, 126.4, 97.8, 88.8, 83.7, 72.7, 37.9, 29.0, 24.9, 24.8, 22.3, 22.2, 18.7, 18.5, 11.6.

HRMS (ESI) m/z [M – H_2O + H]$^+$ calcd for $C_{29}H_{48}SiBO_2$: 467.3522, found: 467.3521.

$[\alpha]^{24}_D$ = –105.2 (c = 0.5, $CHCl_3$); 96% ee, from (R,S)-**L1**.

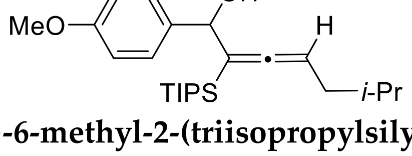

1-(4-methoxyphenyl)-6-methyl-2-(triisopropylsilyl)hepta-2,3-dien-1-ol (Figure 3, entry 10). The title compound was prepared according to the **GP-3** from 4-methoxybenzaldehyde, enyne **1** and DHP ester **2**, purified by flash column chromatography on silica gel: 10→15% EtOAc in hexanes, colorless liquid.

(R,S)-**L1**: 125 mg, 81% yield, > 20:1 dr, 95% ee; (S,R)-**L1**: 136 mg, 88% yield, > 20:1 dr, 95% ee.

HPLC analysis: The ee was determined on a CHIRALPAK IC-3 column (3% *i*-PrOH in hexane, 1.0 mL/min); retention times for compound obtained using (*R,S*)-**L1**: 5.6 min (major), 5.2 min (minor).

^{1}H NMR (500 MHz, $CDCl_3$) δ 7.32 – 7.27 (m, 2H), 6.87 – 6.83 (m, 2H), 5.13 – 5.05 (m, 2H), 3.80 (s, 3H), 2.28 (d, *J* = 5.1 Hz, 1H), 2.03 – 1.90 (m, 2H), 1.62 (td, *J* = 13.4, 6.7 Hz, 1H), 1.16 – 1.07 (m, 3H), 1.05 (d, *J* = 6.9 Hz, 9H), 0.95 – 0.88 (m, 15H).

^{13}C NMR (126 MHz, $CDCl_3$) δ 206.2, 159.1, 135.8, 128.5, 113.6, 98.1, 88.9, 72.1, 55.3, 38.2, 29.0, 22.4, 22.2, 18.7, 18.4, 11.6.

HRMS (ESI) m/z [M – H_2O + H]$^+$ calcd for $C_{24}H_{39}SiO$: 371.2770, found: 371.2737.

$[\alpha]^{24}_D$ = –130.8 (*c* = 0.5, $CHCl_3$); 95% ee, from (*R,S*)-**L1**.

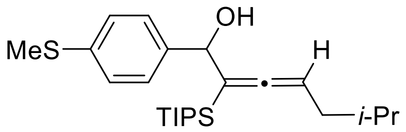

6-methyl-1-(4-(methylthio)phenyl)-2-(triisopropylsilyl)hepta-2,3-dien-1-ol (Figure 3, entry 11). The title compound was prepared according to the **GP-3** from 4-(methylthio)benzaldehyde, enyne **1** and DHP ester **2**, purified by flash column chromatography on silica gel: 10→15% EtOAc in hexanes, colorless liquid.

(*R,S*)-**L1**: 143 mg, 89% yield, > 20:1 dr, 95% ee; (*S,R*)-**L1**: 142 mg, 88% yield, > 20:1 dr, 94% ee.

HPLC analysis: The ee was determined on a CHIRALPAK IC-3 column (5% *i*-PrOH in hexane, 1.0 mL/min); retention times for compound obtained using (*R,S*)-**L1**: 4.6 min (major), 4.3 min (minor).

^{1}H NMR (500 MHz, $CDCl_3$) δ 7.31 – 7.27 (m, 2H), 7.23 – 7.20 (m, 2H), 5.12 – 5.04 (m, 2H), 2.47 (s, 3H), 2.26 (d, *J* = 5.4 Hz, 1H), 1.99 – 1.86 (m, 2H), 1.62 – 1.56 (m, 1H), 1.17 – 1.08 (m, 3H), 1.06 (d, *J* = 7.1 Hz, 9H), 0.94 (d, *J* = 7.2 Hz, 9H), 0.88 (dd, *J* = 8.4, 6.7 Hz, 6H).

^{13}C NMR (126 MHz, $CDCl_3$) δ 206.7, 140.7, 137.5, 127.7, 126.6, 97.9, 88.9, 72.2, 38.0, 29.0, 22.3, 22.2, 18.7, 18.5, 16.1, 11.6.

HRMS (ESI) m/z [M – H_2O + H]$^+$ calcd for $C_{24}H_{39}SiS$: 387.2542, found: 387.2507.

$[\alpha]^{24}_D$ = –172.4 (*c* = 0.5, $CHCl_3$); 95% ee, from (*R,S*)-**L1**.

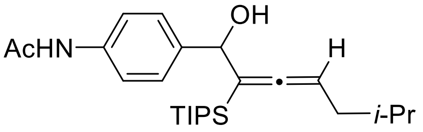

***N*-(4-(1-hydroxy-6-methyl-2-(triisopropylsilyl)hepta-2,3-dien-1-yl)phenyl)acetamide (Figure 3, entry 12).** The title compound was prepared

according to the **GP-3** from *N*-(4-formylphenyl)acetamide, enyne **1** and DHP ester **2**, purified by flash column chromatography on silica gel: 10→15% EtOAc in hexanes, white solid.

(*R,S*)-**L1**: 131 mg, 79% yield, > 20:1 dr, 94% ee; (*S,R*)-**L1**: 122 mg, 74% yield, > 20:1 dr, 94% ee.

HPLC analysis: The ee was determined on a CHIRALPAK IC-3 column (15% *i*-PrOH in hexane, 1.0 mL/min); retention times for compound obtained using (*R,S*)-**L1**: 9.6 min (major), 10.2 min (minor).

^{1}H NMR (500 MHz, $CDCl_3$) δ 7.47 (d, *J* = 8.4 Hz, 2H), 7.33 (d, *J* = 8.4 Hz, 2H), 7.17 (s, 1H), 5.10 – 5.07 (m, 2H), 2.30 (d, *J* = 5.6 Hz, 1H), 2.17 (s, 3H), 2.01 – 1.88 (m, 2H), 1.66 – 1.58 (m, 1H), 1.16 – 1.08 (m, 3H), 1.05 (d, *J* = 7.0 Hz, 9H), 0.93 (d, *J* = 7.2 Hz, 9H), 0.90 (t, *J* = 6.6 Hz, 6H).

^{13}C NMR (126 MHz, $CDCl_3$) δ 206.5, 168.1, 139.4, 137.4, 127.9, 119.2, 97.9, 89.0, 72.1, 38.0, 29.0, 24.7, 22.4, 22.2, 18.7, 18.4, 11.6.

HRMS (ESI) m/z $[M - H_2O + H]^+$ calcd for $C_{25}H_{40}SiNO$: 398.2879, found: 398.2893.

$[\alpha]^{24}_D$ = –201.2 (*c* = 0.5, $CHCl_3$); 94% ee, from (*R,S*)-**L1**.

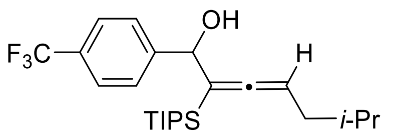

6-methyl-1-(4-(trifluoromethyl)phenyl)-2-(triisopropylsilyl)hepta-2,3-dien-1-ol (Figure 3, entry 13). The title compound was prepared according to the **GP-3** from 4-(trifluoromethyl)benzaldehyde, enyne **1** and DHP ester **2**, purified by flash column chromatography on silica gel: 5→10% EtOAc in hexanes, colorless liquid.

(*R,S*)-**L1**: 102 mg, 60% yield, 10:1 dr, 94% ee; (*S,R*)-**L1**: 102 mg, 60% yield, 10:1 dr, 94% ee.

HPLC analysis: The ee was determined on a CHIRALCEL OD-3 column (3% *i*-PrOH in hexane, 1.0 mL/min); retention times for compound obtained using (*R,S*)-**L1**: 3.9 min (major), 5.4 min (minor).

^{1}H NMR (500 MHz, $CDCl_3$) δ 7.58 (d, *J* = 8.1 Hz, 2H), 7.48 (d, *J* = 8.2 Hz, 2H), 5.22 (d, *J* = 4.5 Hz, 1H), 5.00 (ddd, *J* = 8.6, 7.0, 1.9 Hz, 1H), 2.25 (d, *J* = 5.9 Hz, 1H), 1.87 (dt, *J* = 13.7, 6.8 Hz, 1H), 1.77 (ddd, *J* = 14.0, 8.2, 7.0 Hz, 1H), 1.44 (dt, *J* = 13.4, 6.7 Hz, 1H), 1.23 – 1.11 (m, 3H), 1.08 (d, *J* = 7.2 Hz, 9H), 0.99 (d, *J* = 7.3 Hz, 9H), 0.81 (dd, *J* = 19.8, 6.6 Hz, 6H).

^{13}C NMR (126 MHz, $CDCl_3$) δ 207.8 (s), 147.8 (s), 129.6 (q, *J* = 32.3 Hz), 127.2 (s), 125.0 (q, *J* = 3.8 Hz), 124.2 (q, *J* = 271.9 Hz), 97.8 (s), 88.9 (s), 72.3 (s), 37.7 (s), 29.0 (s), 22.2 (s), 22.1 (s), 18.6 (s), 18.5 (s), 11.6 (s).

^{19}F NMR (471 MHz, $CDCl_3$) δ -62.45.

HRMS (APCI) m/z $[M - H_2O + H]^+$ calcd for $C_{24}H_{36}SiF_3$: 409.2538, found: 409.2509.

[α]$^{24}_{D}$ = –86.8 (*c* = 0.5, $CHCl_3$); 94% ee, from (*R,S*)-**L1**.

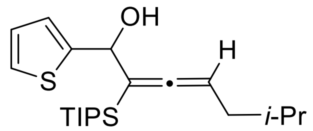

6-methyl-1-(thiophen-2-yl)-2-(triisopropylsilyl)hepta-2,3-dien-1-ol (Figure 3, entry 14). The title compound was prepared according to the **GP-3** from thiophene-2-carbaldehyde, enyne **1** and DHP ester **2**, purified by flash column chromatography on silica gel: 5→10% EtOAc in hexanes, colorless liquid.

(*R,S*)-**L1**: 125 mg, 86% yield, > 20:1 dr, 97% ee; (*S,R*)-**L1**: 129 mg, 89% yield, > 20:1 dr, 97% ee.

HPLC analysis: The ee was determined on a CHIRALCEL OD-3 column (3% *i*-PrOH in hexane, 1.0 mL/min); retention times for compound obtained using (*R,S*)-**L1**: 3.9 min (major), 4.4 min (minor).

^{1}H NMR (500 MHz, $CDCl_3$) δ 7.23 (dd, *J* = 5.1, 1.0 Hz, 1H), 7.01 (d, *J* = 3.0 Hz, 1H), 6.93 (dd, *J* = 5.0, 3.5 Hz, 1H), 5.33 (s, 1H), 5.16 (td, *J* = 7.6, 1.7 Hz, 1H), 2.46 (brs, 1H), 2.03 (t, *J* = 7.2 Hz, 2H), 1.72 – 1.59 (m, 1H), 1.21 – 1.14 (m, 3H), 1.09 (d, *J* = 7.3 Hz, 9H), 0.99 (d, *J* = 7.3 Hz, 9H), 0.93 (dd, *J* = 6.6, 5.3 Hz, 6H).

^{13}C NMR (126 MHz, $CDCl_3$) δ 206.5, 148.5, 126.4, 125.2 (two carbons), 98.4, 89.7, 67.8, 37.6, 29.0, 22.3, 22.2, 18.6, 18.4, 11.6.

HRMS (ESI) m/z [M – H_2O + H]$^+$ calcd for $C_{21}H_{35}SiS$: 347.2229, found: 347.2224.

[α]$^{24}_{D}$ = –148.8 (*c* = 0.5, $CHCl_3$); 97% ee, from (*R,S*)-**L1**.

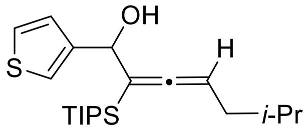

6-methyl-1-(thiophen-3-yl)-2-(triisopropylsilyl)hepta-2,3-dien-1-ol (Figure 3, entry 15). The title compound was prepared according to the **GP-3** from thiophene-3-carbaldehyde, enyne **1** and DHP ester **2**, purified by flash column chromatography on silica gel: 5→10% EtOAc in hexanes, colorless liquid.

(*R,S*)-**L1**: 126 mg, 87% yield, > 20:1 dr, 95% ee; (*S,R*)-**L1**: 127 mg, 88% yield, > 20:1 dr, 95% ee.

HPLC analysis: The ee was determined on a CHIRALCEL OD-3 column (3% *i*-PrOH in hexane, 1.0 mL/min); retention times for compound obtained using (*R,S*)-**L1**: 4.0 min (major), 4.8 min (minor).

^{1}H NMR (500 MHz, $CDCl_3$) δ 7.27 (dd, *J* = 3.1, 1.8 Hz, 1H), 7.22 – 7.19 (m, 1H), 7.10 (dd, *J* = 5.0, 1.2 Hz, 1H), 5.21 (d, *J* = 1.0 Hz, 1H), 5.10 (td, *J* = 7.6, 1.9 Hz, 1H), 2.25 (brs, 1H), 2.03 – 1.89 (m, 2H), 1.67 – 1.55 (m, 1H), 1.20 – 1.13 (m, 3H),

1.08 (d, J = 7.2 Hz, 9H), 0.98 (d, J = 7.3 Hz, 9H), 0.91 (t, J = 6.8 Hz, 6H).

^{13}C NMR (126 MHz, $CDCl_3$) δ 206.7, 145.3, 126.7, 125.8, 122.0, 97.8, 88.9, 68.4, 38.0, 29.0, 22.3, 22.2, 18.6, 18.4, 11.6.

HRMS (ESI) m/z [M – H_2O + H]$^+$ calcd for $C_{21}H_{35}SiS$: 347.2229, found: 347.2226.

$[\alpha]^{24}_D$ = –164.0 (c = 0.5, $CHCl_3$); 95% ee, from (R,S)-**L1**.

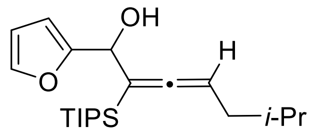

1-(furan-2-yl)-6-methyl-2-(triisopropylsilyl)hepta-2,3-dien-1-ol (Figure 3, entry 16). The title compound was prepared according to the **GP-3** from furan-2-carbaldehyde, enyne **1** and DHP ester **2**, purified by flash column chromatography on silica gel: 5→10% EtOAc in hexanes, colorless liquid.

(R,S)-**L1**: 116 mg, 84% yield, 8:1 dr, 88% ee; (S,R)-**L1**: 116 mg, 84% yield, 8:1 dr, 88% ee.

HPLC analysis: The ee was determined on a CHIRALCEL OD-3 column (3% i-PrOH in hexane, 1.0 mL/min); retention times for compound obtained using (R,S)-**L1**: 3.6 min (major), 4.0 min (minor).

^{1}H NMR (500 MHz, $CDCl_3$) δ 7.37 – 7.34 (m, 1H), 6.30 (dd, J = 3.2, 1.8 Hz, 1H), 6.25 (d, J = 3.2 Hz, 1H), 5.16 (td, J = 7.6, 1.9 Hz, 1H), 5.08 (s, 1H), 2.35 (brs, 1H), 2.05 – 1.92 (m, 2H), 1.71 – 1.59 (m, 1H), 1.18 – 1.09 (m, 3H), 1.07 (d, J = 7.1 Hz, 9H), 0.99 (d, J = 7.2 Hz, 9H), 0.92 (dd, J = 6.6, 3.7 Hz, 6H).

^{13}C NMR (126 MHz, $CDCl_3$) δ 206.9, 156.0, 142.0, 110.1, 107.2, 95.4, 89.4, 65.6, 37.9, 28.9, 22.4, 22.2, 18.6, 18.4, 11.5.

HRMS (ESI) m/z [M – H_2O + H]$^+$ calcd for $C_{21}H_{35}SiO$: 331.2457, found: 331.2455.

$[\alpha]^{24}_D$ = –156.8 (c = 0.5, $CHCl_3$); 88% ee, from (R,S)-**L1**.

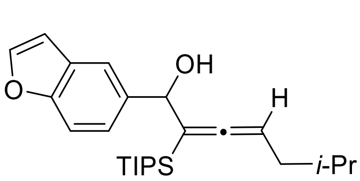

1-(benzofuran-5-yl)-6-methyl-2-(triisopropylsilyl)hepta-2,3-dien-1-ol (Figure 3, entry 17). The title compound was prepared according to the **GP-3** from benzofuran-5-carbaldehyde, enyne **1** and DHP ester **2**, purified by flash column chromatography on silica gel: 10→15% EtOAc in hexanes, colorless liquid.

(R,S)-**L1**: 138 mg, 87% yield, 15:1 dr, 92% ee; (S,R)-**L1**: 136 mg, 85% yield, 15:1 dr, 91% ee.

HPLC analysis: The ee was determined on a CHIRALCEL OD-3 column (3%

i-PrOH in hexane, 1.0 mL/min); retention times for compound obtained using (*R,S*)-**L1**: 5.0 min (major), 6.8 min (minor).

^{1}H NMR (500 MHz, $CDCl_3$) δ 7.61 (t, *J* = 2.4 Hz, 2H), 7.45 (d, *J* = 8.5 Hz, 1H), 7.33 (dd, *J* = 8.5, 1.7 Hz, 1H), 6.74 (dd, *J* = 2.1, 0.7 Hz, 1H), 5.24 (d, *J* = 2.7 Hz, 1H), 5.10 (td, *J* = 7.7, 2.2 Hz, 1H), 2.36 (d, *J* = 5.4 Hz, 1H), 2.02 – 1.89 (m, 2H), 1.62 – 1.56 (m, 1H), 1.18 – 1.09 (m, 3H), 1.06 (d, *J* = 7.1 Hz, 9H), 0.92 (d, *J* = 7.2 Hz, 9H), 0.87 (dd, *J* = 11.3, 6.6 Hz, 6H).

^{13}C NMR (126 MHz, $CDCl_3$) δ 206.5, 154.5, 145.3, 138.3, 127.2, 123.8, 119.9, 111.1, 106.7, 98.4, 89.0, 72.8, 38.1, 29.0, 22.3, 22.2, 18.7, 18.4, 11.6.

HRMS (ESI) m/z [M – H_2O + H]$^+$ calcd for $C_{25}H_{37}SiO$: 381.2614, found: 381.2601.

$[\alpha]^{24}_D$ = –156.8 (*c* = 0.5, $CHCl_3$); 92% ee, from (*R,S*)-**L1**.

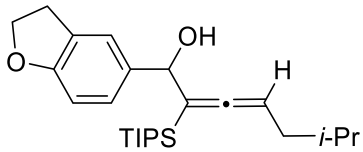

1-(2,3-dihydrobenzofuran-5-yl)-6-methyl-2-(triisopropylsilyl)hepta-2,3-dien-1-ol (Figure 3, entry 18). The title compound was prepared according to the **GP-3** from 2,3-dihydrobenzofuran-5-carbaldehyde, enyne **1** and DHP ester **2**, purified by flash column chromatography on silica gel: 10→15% EtOAc in hexanes, colorless liquid.

(*R,S*)-**L1**: 115 mg, 72% yield, > 20:1 dr, 94% ee; (*S,R*)-**L1**: 116 mg, 73% yield, > 20:1 dr, 92% ee.

SFC analysis: The ee was determined on a CHIRALPAK IC-3 column (5% *i*-PrOH in CO_2, 2.0 mL/min); retention times for compound obtained using (*R,S*)-**L1**: 6.0 min (major), 4.5 min (minor).

^{1}H NMR (500 MHz, $CDCl_3$) δ 7.23 (s, 1H), 7.11 (d, *J* = 8.2 Hz, 1H), 6.71 (d, *J* = 8.2 Hz, 1H), 5.10 (td, *J* = 7.6, 2.2 Hz, 1H), 5.08 – 5.03 (m, 1H), 4.56 (t, *J* = 8.7 Hz, 2H), 3.18 (t, *J* = 8.6 Hz, 2H), 2.27 (d, *J* = 5.7 Hz, 1H), 2.03 – 1.91 (m, 2H), 1.62 (dp, *J* = 13.3, 6.7 Hz, 1H), 1.12 (dt, *J* = 9.6, 7.2 Hz, 3H), 1.06 (d, *J* = 6.9 Hz, 9H), 0.96 – 0.88 (m, 15H).

^{13}C NMR (126 MHz, $CDCl_3$) δ 206.1, 159.6, 135.7, 127.3, 126.9, 124.0, 108.7, 98.2, 88.9, 72.3, 71.3, 38.2, 29.7, 29.1, 22.4, 22.2, 18.7, 18.4, 11.6.

HRMS (ESI) m/z [M – H_2O + H]$^+$ calcd for $C_{25}H_{39}SiO$: 383.2770, found: 383.2844.

$[\alpha]^{24}_D$ = –136.8 (*c* = 0.5, $CHCl_3$); 94% ee, from (*R,S*)-**L1**.

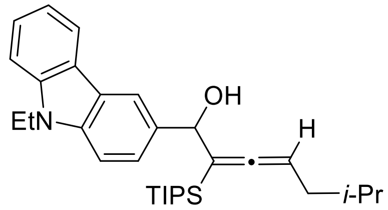

1-(9-ethyl-9*H*-carbazol-3-yl)-6-methyl-2-(triisopropylsilyl)hepta-2,3-dien-1-ol (Figure 3, entry 19). The title compound was prepared according to the **GP-3** from 9-ethyl-9*H*-carbazole-3-carbaldehyde, enyne **1** and DHP ester **2**, purified by flash column chromatography on silica gel: 10→15% EtOAc in hexanes, colorless liquid.

(*R,S*)-**L1**: 159 mg, 84% yield, 19:1 dr, 94% ee; (*S,R*)-**L1**: 159 mg, 84% yield, 19:1 dr, 94% ee.

HPLC analysis: The ee was determined on a CHIRALCEL OJ-3 column (3% *i*-PrOH in hexane, 1.0 mL/min); retention times for compound obtained using (*R,S*)-**L1**: 5.8 min (major), 7.4 min (minor).

^{1}H NMR (500 MHz, $CDCl_3$) δ 8.13 (d, *J* = 1.3 Hz, 1H), 8.07 (d, *J* = 7.7 Hz, 1H), 7.51 (dd, *J* = 8.4, 1.6 Hz, 1H), 7.48 – 7.43 (m, 1H), 7.40 (d, *J* = 8.1 Hz, 1H), 7.35 (d, *J* = 8.4 Hz, 1H), 7.22 (dd, *J* = 11.1, 4.0 Hz, 1H), 5.33 (dd, *J* = 6.0, 2.1 Hz, 1H), 5.16 (td, *J* = 7.6, 2.2 Hz, 1H), 4.35 (q, *J* = 7.2 Hz, 2H), 2.42 (d, *J* = 6.0 Hz, 1H), 2.12 – 1.98 (m, 2H), 1.64 (dp, *J* = 13.4, 6.7 Hz, 1H), 1.40 (t, *J* = 7.2 Hz, 3H), 1.16 (dd, *J* = 15.0, 7.5 Hz, 3H), 1.07 (d, *J* = 7.2 Hz, 9H), 0.94 – 0.86 (m, 15H).

^{13}C NMR (126 MHz, $CDCl_3$) δ 206.3, 140.3, 139.7, 134.0, 125.5, 125.4, 123.1, 122.7, 120.3, 119.4, 118.7, 108.5, 108.2, 98.4, 88.8, 73.0, 38.3, 37.6, 29.1, 22.4, 22.3, 18.7, 18.4, 13.7, 11.6.

HRMS (ESI) m/z [M – H_2O + H]$^+$ calcd for $C_{31}H_{44}SiN$: 458.3243, found: 458.3244.

$[\alpha]^{24}_D$ = –218.8 (*c* = 0.5, $CHCl_3$); 94% ee, from (*R,S*)-**L1**.

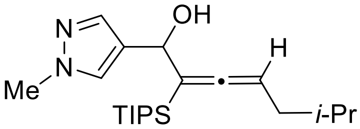

6-methyl-1-(1-methyl-1*H*-pyrazol-4-yl)-2-(triisopropylsilyl)hepta-2,3-dien-1-ol (Figure 3, entry 20). The title compound was prepared according to the **GP-3** from 1-methyl-1*H*-pyrazole-4-carbaldehyde, enyne **1** and DHP ester **2**, purified by flash column chromatography on silica gel: 10→15% EtOAc in hexanes, colorless liquid.

(*R,S*)-**L1**: 78 mg, 54% yield, 20:1 dr, 96% ee; (*S,R*)-**L1**: 82 mg, 57% yield, 20:1 dr, 95% ee.

HPLC analysis: The ee was determined on a CHIRALCEL OD-3 column (3% *i*-PrOH in hexane, 1.0 mL/min); retention times for compound obtained using (*R,S*)-**L1**: 11.4 min (major), 15.4 min (minor).

^{1}H NMR (500 MHz, $CDCl_3$) δ 7.43 (s, 1H), 7.29 (s, 1H), 5.09 – 5.05 (m, 2H),

3.85 (s, 3H), 2.18 (d, *J* = 6.1 Hz, 1H), 2.01 – 1.87 (m, 2H), 1.61 (dp, *J* = 13.3, 6.7 Hz, 1H), 1.19 – 1.10 (m, 3H), 1.06 (d, *J* = 7.3 Hz, 9H), 0.99 (d, *J* = 7.3 Hz, 9H), 0.89 (dd, *J* = 9.2, 6.7 Hz, 6H).

^{13}C NMR (126 MHz, $CDCl_3$) δ 207.0, 138.2, 128.7, 125.7, 97.9, 88.7, 64.7, 38.9, 38.1, 29.0, 22.4, 22.2, 18.7, 18.5, 11.6.

HRMS (ESI) m/z [M – H_2O + H]$^+$ calcd for $C_{21}H_{37}SiN_2$: 345.2726, found: 345.2691.

[α]$^{24}_{D}$ = –110.0 (*c* = 0.5, $CHCl_3$); 96% ee, from (*R,S*)-**L1**.

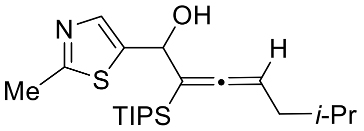

6-methyl-1-(2-methylthiazol-5-yl)-2-(triisopropylsilyl)hepta-2,3-dien-1-ol (Figure 3, entry 21). The title compound was prepared according to the **GP-3** from 2-methylthiazole-5-carbaldehyde, enyne **1** and DHP ester **2**, purified by flash column chromatography on silica gel: 10→15% EtOAc in hexanes, colorless liquid.

(*R,S*)-**L1**: 109 mg, 72% yield, 13:1 dr, 94% ee; (*S,R*)-**L1**: 103 mg, 68% yield, 13:1 dr, 96% ee.

HPLC analysis: The ee was determined on a CHIRALCEL OD-3 column (3% *i*-PrOH in hexane, 1.0 mL/min); retention times for compound obtained using (*R,S*)-**L1**: 6.1 min (major), 6.9 min (minor).

^{1}H NMR (500 MHz, $CDCl_3$) δ 7.48 (s, 1H), 5.29 (brs, 1H), 5.16 (td, *J* = 7.6, 1.5 Hz, 1H), 2.67 (s, 3H), 2.36 (d, *J* = 6.8 Hz, 1H), 2.00 (t, *J* = 7.2 Hz, 2H), 1.67 – 1.59 (m, 1H), 1.21 – 1.11 (m, 3H), 1.08 (d, *J* = 7.3 Hz, 9H), 1.00 (d, *J* = 7.3 Hz, 9H), 0.91 (t, *J* = 6.4 Hz, 6H).

^{13}C NMR (126 MHz, $CDCl_3$) δ 206.8, 166.5, 142.8, 139.7, 98.3, 90.1, 66.1, 37.6, 29.0, 22.3, 22.2, 19.4, 18.6, 18.5, 11.5.

HRMS (ESI) m/z [M – H_2O + H]$^+$ calcd for $C_{21}H_{36}SiNS$: 362.2338, found: 362.2365.

[α]$^{24}_{D}$ = –109.2 (*c* = 0.5, $CHCl_3$); 94% ee, from (*R,S*)-**L1**.

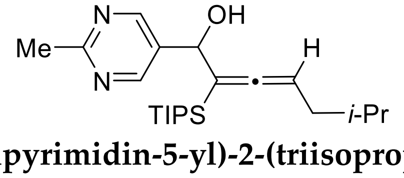

6-methyl-1-(2-methylpyrimidin-5-yl)-2-(triisopropylsilyl)hepta-2,3-dien-1-ol (Figure 3, entry 22). The title compound was prepared according to the **GP-3** from 2-methylpyrimidine-5-carbaldehyde, enyne **1** and DHP ester **2**, purified by flash column chromatography on silica gel: 15→20% EtOAc in hexanes, colorless liquid.

(*R,S*)-**L1**: 113 mg, 76% yield, 8:1 dr, 94% ee; (*S,R*)-**L1**: 114 mg, 77% yield, 8:1

dr, 94% ee.

HPLC analysis: The ee was determined on a CHIRALPAK IC-3 column (15% *i*-PrOH in hexane, 1.0 mL/min); retention times for compound obtained using (*R,S*)-**L1**: 6.8 min (major), 7.8 min (minor).

^{1}H NMR (500 MHz, $CDCl_3$) δ 8.63 (s, 2H), 5.19 (s, 1H), 5.07 – 5.00 (m, 1H), 2.72 (s, 3H), 1.90 (dt, *J* = 13.8, 6.8 Hz, 1H), 1.85 – 1.75 (m, 1H), 1.62 (brs, 1H), 1.56 – 1.44 (m, 1H), 1.21 – 1.13 (m, 3H), 1.08 (d, *J* = 7.2 Hz, 9H), 1.01 (d, *J* = 7.3 Hz, 9H), 0.83 (d, *J* = 6.7 Hz, 3H), 0.79 (d, *J* = 6.6 Hz, 3H).

^{13}C NMR (126 MHz, $CDCl_3$) δ 208.2, 167.1, 155.8, 133.5, 97.4, 89.4, 68.9, 37.6, 28.9, 25.7, 22.2, 22.0, 18.6 (two carbons), 11.6.

HRMS (ESI) m/z $[M - H_2O + H]^+$ calcd for $C_{22}H_{37}SiN_2$: 357.2726, found: 357.2695.

$[\alpha]^{24}_D$ = –63.6 (*c* = 0.5, $CHCl_3$); 94% ee, from (*R,S*)-**L1**.

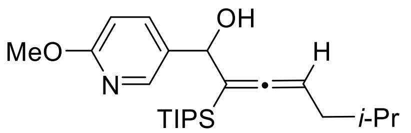

1-(6-methoxypyridin-3-yl)-6-methyl-2-(triisopropylsilyl)hepta-2,3-dien-1-ol (Figure 3, entry 23). The title compound was prepared according to the **GP-3** from 6-methoxynicotinaldehyde, enyne **1** and DHP ester **2**, purified by flash column chromatography on silica gel: 15→20% EtOAc in hexanes, colorless liquid.

(*R,S*)-**L1**: 118 mg, 76% yield, 20:1 dr, 94% ee; (*S,R*)-**L1**: 119 mg, 77% yield, 20:1 dr, 94% ee.

HPLC analysis: The ee was determined on a CHIRALCEL OD-3 column (3% *i*-PrOH in hexane, 1.0 mL/min); retention times for compound obtained using (*R,S*)-**L1**: 6.1 min (major), 5.7 min (minor).

^{1}H NMR (500 MHz, $CDCl_3$) δ 8.12 (d, *J* = 2.3 Hz, 1H), 7.61 (dd, *J* = 8.6, 2.4 Hz, 1H), 6.72 (d, *J* = 8.6 Hz, 1H), 5.14 – 5.11 (m, 1H), 5.08 (td, *J* = 7.6, 2.1 Hz, 1H), 3.92 (s, 3H), 2.28 (d, *J* = 5.5 Hz, 1H), 1.99 – 1.85 (m, 2H), 1.57 (td, *J* = 13.4, 6.7 Hz, 1H), 1.18 – 1.09 (m, 3H), 1.06 (d, *J* = 7.1 Hz, 9H), 0.95 (d, *J* = 7.2 Hz, 9H), 0.87 (dd, *J* = 9.8, 6.7 Hz, 6H).

^{13}C NMR (126 MHz, $CDCl_3$) δ 206.7, 163.7, 145.5, 137.8, 131.9, 110.7, 97.8, 89.2, 70.0, 53.5, 38.0, 29.0, 22.3, 22.1, 18.7, 18.5, 11.6.

HRMS (ESI) m/z $[M - H_2O + H]^+$ calcd for $C_{23}H_{38}SiON$: 372.2723, found: 372.3690.

$[\alpha]^{24}_D$ = –290.8 (*c* = 0.5, $CHCl_3$); 94% ee, from (*R,S*)-**L1**.

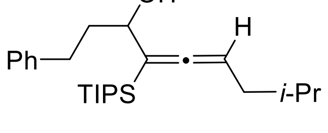

8-methyl-1-phenyl-4-(triisopropylsilyl)nona-4,5-dien-3-ol (Figure 3, entry 24). The title compound was prepared according to the **GP-3** from 3-phenylpropanal, enyne **1** and DHP ester **2**, purified by flash column chromatography on silica gel: 5→10% EtOAc in hexanes, colorless liquid.

(*R,S*)-**L1**: 134 mg, 87% yield, 10:1 dr, 88% ee; (*S,R*)-**L1**: 134 mg, 87% yield, 10:1 dr, 86% ee.

HPLC analysis: The ee was determined on a CHIRALCEL OD-3 column (3% *i*-PrOH in hexane, 1.0 mL/min); retention times for compound obtained using (*R,S*)-**L1**: 3.8 min (major), 4.1 min (minor).

^{1}H NMR (500 MHz, $CDCl_3$) δ 7.27 (dd, *J* = 12.4, 4.9 Hz, 2H), 7.18 (dd, *J* = 14.9, 7.3 Hz, 3H), 5.03 (td, *J* = 7.6, 1.4 Hz, 1H), 4.02 (d, *J* = 5.6 Hz, 1H), 2.89 – 2.80 (m, 1H), 2.68 (ddd, *J* = 13.7, 10.1, 6.6 Hz, 1H), 2.03 – 1.89 (m, 3H), 1.87 – 1.77 (m, 1H), 1.70 – 1.59 (m, 1H), 1.14 (ddd, *J* = 10.3, 8.6, 5.0 Hz, 3H), 1.09 – 1.02 (m, 18H), 0.92 (d, *J* = 6.7 Hz, 6H).

^{13}C NMR (126 MHz, $CDCl_3$) δ 206.6, 142.2, 128.5, 128.3, 125.7, 97.5, 88.2, 69.3, 40.4, 38.1, 32.5, 29.0, 22.4, 22.2, 18.7, 11.6.

HRMS (ESI) m/z $[M - H_2O + H]^+$ calcd for $C_{25}H_{41}Si$: 369.2978, found: 369.2950.

$[\alpha]^{24}_{D}$ = –40.0 (*c* = 0.5, $CHCl_3$); 88% ee, from (*R,S*)-**L1**.

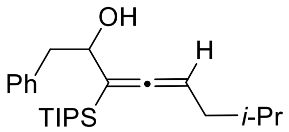

7-methyl-1-phenyl-3-(triisopropylsilyl)octa-3,4-dien-2-ol (Figure 3, entry 25). The title compound was prepared according to the **GP-3** from 2-phenylacetaldehyde, enyne **1** and DHP ester **2**, purified by flash column chromatography on silica gel: 5→10% EtOAc in hexanes, colorless liquid.

(*R,S*)-**L1**: 122 mg, 82% yield, 7:1 dr, 88% ee; (*S,R*)-**L1**: 122 mg, 82% yield, 7:1 dr, 88% ee.

HPLC analysis: The ee was determined on a CHIRALCEL OD-3 column (5% *i*-PrOH in hexane, 1.0 mL/min); retention times for compound obtained using (*R,S*)-**L1**: 3.2 min (major), 3.4 min (minor).

^{1}H NMR (600 MHz, $CDCl_3$) δ 7.30 (t, *J* = 7.4 Hz, 2H), 7.25 – 7.20 (m, 3H), 5.07 – 5.01 (m, 1H), 4.23 (d, *J* = 7.2 Hz, 1H), 3.02 (dd, *J* = 13.9, 3.3 Hz, 1H), 2.79 (dd, *J* = 13.9, 9.2 Hz, 1H), 2.01 – 1.89 (m, 2H), 1.65 (td, *J* = 13.4, 6.7 Hz, 1H), 1.58 (brs, 1H), 1.21 (dq, *J* = 14.2, 7.3 Hz, 3H), 1.09 (t, *J* = 7.2 Hz, 18H), 0.95 (dd, *J* = 6.6, 2.1 Hz, 6H).

^{13}C NMR (151 MHz, $CDCl_3$) δ 207.5, 139.2, 129.5, 128.4, 126.3, 96.8, 87.8, 71.1, 44.9, 38.0, 29.0, 22.4, 22.3, 18.7 (two carbons), 11.6.

HRMS (ESI) m/z $[M - H_2O + H]^+$ calcd for $C_{24}H_{39}Si$: 355.2821, found: 355.2815.

$[\alpha]^{24}_{D}$ = +8.8 (*c* = 0.5, $CHCl_3$); 88% ee, from (*R,S*)-**L1**.

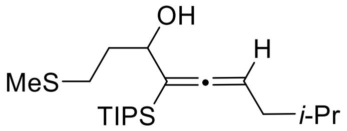

8-methyl-1-(methylthio)-4-(triisopropylsilyl)nona-4,5-dien-3-ol (Figure 3, entry 26). The title compound was prepared according to the **GP-3** from 3-(methylthio)propanal, enyne **1** and DHP ester **2**, purified by flash column chromatography on silica gel: 5→10% EtOAc in hexanes, colorless liquid.

(*R,S*)-**L1**: 104 mg, 73% yield, 10:1 dr, 80% ee; (*S,R*)-**L1**: 104 mg, 73% yield, 10:1 dr, 78% ee.

HPLC analysis: The ee was determined on a CHIRALCEL OD-3 column (5% *i*-PrOH in hexane, 1.0 mL/min); retention times for compound obtained using (*R,S*)-**L1**: 3.2 min (major), 3.5 min (minor).

^{1}H NMR (500 MHz, $CDCl_3$) δ 5.01 (td, *J* = 7.7, 1.4 Hz, 1H), 4.12 (d, *J* = 6.3 Hz, 1H), 2.71 – 2.55 (m, 2H), 2.10 (s, 3H), 1.99 – 1.87 (m, 3H), 1.80 (dtd, *J* = 13.9, 8.5, 5.3 Hz, 1H), 1.64 (dq, *J* = 13.3, 6.7 Hz, 2H), 1.22 – 1.13 (m, 3H), 1.12 – 1.05 (m, 18H), 0.93 (dd, *J* = 6.7, 2.1 Hz, 6H).

^{13}C NMR (126 MHz, $CDCl_3$) δ 206.8, 97.1, 88.1, 69.0, 38.1, 37.7, 31.0, 29.0, 22.4, 22.2, 18.7 (two carbons), 15.5, 11.6.

HRMS (ESI) m/z [M – H_2O + H]$^+$ calcd for $C_{20}H_{39}SiS$: 339.2542, found: 339.2549.

$[\alpha]^{24}_{D}$ = –23.6 (*c* = 0.5, $CHCl_3$); 80% ee, from (*R,S*)-**L1**.

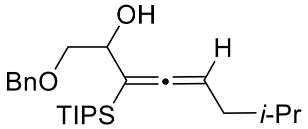

1-(benzyloxy)-7-methyl-3-(triisopropylsilyl)octa-3,4-dien-2-ol (Figure 3, entry 27). The title compound was prepared according to the **GP-3** from 2-(benzyloxy)acetaldehyde, enyne **1** and DHP ester **2**, purified by flash column chromatography on silica gel: 5→10% EtOAc in hexanes, colorless liquid.

(*R,S*)-**L1**: 132 mg, 82% yield, 6:1 dr, 82% ee; (*S,R*)-**L1**: 130 mg, 81% yield, 6:1 dr, 80% ee.

HPLC analysis: The ee was determined on a CHIRALCEL OD-3 column (3% *i*-PrOH in hexane, 1.0 mL/min); retention times for compound obtained using (*R,S*)-**L1**: 3.8 min (major), 6.0 min (minor).

^{1}H NMR (500 MHz, $CDCl_3$) δ 7.38 – 7.26 (m, 5H), 5.00 – 4.94 (m, 1H), 4.58 (q, *J* = 11.9 Hz, 2H), 4.26 (d, *J* = 8.3 Hz, 1H), 3.61 (dd, *J* = 9.9, 2.8 Hz, 1H), 3.44 (dd, *J* = 9.8, 8.8 Hz, 1H), 2.34 (brs, 1H), 1.99 – 1.79 (m, 2H), 1.62 (td, *J* = 13.4, 6.7 Hz, 1H), 1.21 – 1.12 (m, 3H), 1.07 (dd, *J* = 9.6, 7.3 Hz, 18H), 0.90 (dd, *J* = 6.7, 3.2 Hz, 6H).

^{13}C NMR (126 MHz, $CDCl_3$) δ 208.0, 138.1, 128.4, 127.7 (two carbons), 92.5, 86.9, 74.9, 73.2, 68.9, 38.0, 28.9, 22.4, 22.2, 18.6 (two carbons), 11.5.

HRMS (ESI) m/z [M – H_2O + H]$^+$ calcd for $C_{25}H_{41}SiO$: 385.2927, found:

385.2927.

$[\alpha]^{24}_{D}$ = –31.6 (c = 0.5, $CHCl_3$); 82% ee, from (R,S)-**L1**.

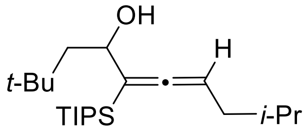

2,2,9-trimethyl-5-(triisopropylsilyl)deca-5,6-dien-4-ol (Figure 3, entry 28). The title compound was prepared according to the **GP-3** from 3,3-dimethylbutanal, enyne **1** and DHP ester **2**, purified by flash column chromatography on silica gel: 5→10% EtOAc in hexanes, colorless liquid.

(R,S)-**L1**: 98 mg, 70% yield, 5:1 dr, 82% ee; (S,R)-**L1**: 99 mg, 70% yield, 5:1 dr, 83% ee.

HPLC analysis: The ee was determined on a CHIRALPAK AD-3 column (0.5% i-PrOH in hexane, 1.0 mL/min); retention times for compound obtained using (R,S)-**L1**: 3.5 min (major), 3.8 min (minor).

^{1}H NMR (500 MHz, $CDCl_3$) δ 5.01 – 4.95 (m, 1H), 4.15 (d, J = 9.6 Hz, 1H), 2.01 – 1.87 (m, 2H), 1.66 (dt, J = 20.1, 6.7 Hz, 1H), 1.61 – 1.56 (m, 2H), 1.46 (dd, J = 14.7, 9.7 Hz, 1H), 1.22 – 1.13 (m, 3H), 1.08 (t, J = 7.1 Hz, 18H), 0.98 (s, 9H), 0.94 (dd, J = 6.6, 2.7 Hz, 6H).

^{13}C NMR (126 MHz, $CDCl_3$) δ 207.2, 99.0, 87.9, 68.2, 52.2, 38.1, 30.5, 30.3, 29.0, 22.5, 22.3, 18.7 (two carbons), 11.6.

HRMS (ESI) m/z $[M – H_2O + H]^+$ calcd for $C_{22}H_{43}Si$: 335.3134, found: 335.3112.

$[\alpha]^{24}_{D}$ = –16.0 (c = 0.5, $CHCl_3$); 82% ee, from (R,S)-**L1**.

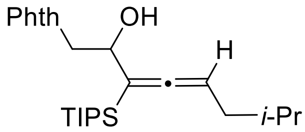

2-(2-hydroxy-7-methyl-3-(triisopropylsilyl)octa-3,4-dien-1-yl)isoindoline-1,3-dione (Figure 3, entry 29). The title compound was prepared according to the **GP-3** from 2-(1,3-dioxoisoindolin-2-yl)acetaldehyde, enyne **1** and DHP ester **2**, purified by flash column chromatography on silica gel: 10→15% EtOAc in hexanes, colorless liquid.

(R,S)-**L1**: 109 mg, 62% yield, 7:1 dr, 89% ee; (S,R)-**L1**: 111 mg, 63% yield, 7:1 dr, 87% ee.

HPLC analysis: The ee was determined on a CHIRALPAK AD-3 column (5% i-PrOH in hexane, 1.0 mL/min); retention times for compound obtained using (R,S)-**L1**: 4.7 min (major), 6.0 min (minor).

^{1}H NMR (500 MHz, $CDCl_3$) δ 7.85 – 7.80 (m, 2H), 7.72 – 7.67 (m, 2H), 5.12 – 5.07 (m, 1H), 4.28 (t, J = 8.0 Hz, 1H), 3.83 (ddd, J = 17.3, 14.2, 6.5 Hz, 2H), 2.04 – 1.92 (m, 2H), 1.85 (d, J = 9.6 Hz, 1H), 1.75 – 1.63 (m, 1H), 1.28 – 1.17 (m, 3H), 1.10 (dd, J = 7.4, 2.9 Hz, 18H), 0.96 (dd, J = 6.6, 5.6 Hz, 6H).

^{13}C NMR (126 MHz, $CDCl_3$) δ 207.4, 168.7, 133.9, 132.1, 123.2, 94.6, 88.4, 68.2, 44.9, 38.1, 29.0, 22.4, 22.3, 18.60 (two carbons), 11.4.

HRMS (ESI) m/z $[M - H_2O + H]^+$ calcd for $C_{26}H_{38}SiNO_2$: 424.2672, found: 424.2672.

$[\alpha]^{24}_D$ = +54.8 (*c* = 0.5, $CHCl_3$); 89% ee, from (*R,S*)-**L1**.

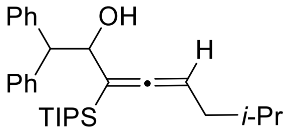

7-methyl-1,1-diphenyl-3-(triisopropylsilyl)octa-3,4-dien-2-ol (Figure 3, entry 30). The title compound was prepared according to the **GP-3** from 2,2-diphenylacetaldehyde, enyne **1** and DHP ester **2**, purified by flash column chromatography on silica gel: 5→10% EtOAc in hexanes, colorless liquid.

(*R,S*)-**L1**: 139 mg, 78% yield, 20:1 dr, 96% ee; (*S,R*)-**L1**: 138 mg, 78% yield, 20:1 dr, 94% ee.

HPLC analysis: The ee was determined on a CHIRALPAK AD-3 column (5% *i*-PrOH in hexane, 1.0 mL/min); retention times for compound obtained using (*R,S*)-**L1**: 3.0 min (major), 3.4 min (minor).

^1H NMR (500 MHz, $CDCl_3$) δ 7.34 (d, *J* = 8.1 Hz, 4H), 7.29 – 7.21 (m, 4H), 7.15 (ddd, *J* = 14.9, 4.9, 1.1 Hz, 2H), 4.86 (m, 2H), 4.25 (d, *J* = 6.9 Hz, 1H), 1.67 (d, *J* = 6.1 Hz, 1H), 1.46 (dd, *J* = 10.4, 4.2 Hz, 2H), 1.40 (dt, *J* = 13.8, 6.7 Hz, 1H), 1.24 – 1.15 (m, 3H), 1.06 (d, *J* = 7.6 Hz, 18H), 0.80 (dd, *J* = 6.4, 4.0 Hz, 6H).

^{13}C NMR (126 MHz, $CDCl_3$) δ 207.7, 143.3, 141.4, 129.5, 128.7, 128.3, 128.2, 126.5, 126.1, 95.3, 88.0, 72.2, 57.2, 36.7, 28.8, 22.2, 22.1, 18.7 (two carbons), 11.8.

HRMS (ESI) m/z $[M - H_2O + H]^+$ calcd for $C_{30}H_{43}Si$: 431.3134, found: 431.3128.

$[\alpha]^{24}_D$ = +41.6 (*c* = 0.5, $CHCl_3$); 96% ee, from (*R,S*)-**L1**.

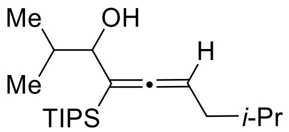

2,8-dimethyl-4-(triisopropylsilyl)nona-4,5-dien-3-ol (Figure 3, entry 31). The title compound was prepared according to the **GP-3** from isobutyraldehyde, enyne **1** and DHP ester **2**, purified by flash column chromatography on silica gel: 5→10% EtOAc in hexanes, colorless liquid.

(*R,S*)-**L1**: 108 mg, 84% yield, > 20:1 dr, 96% ee; (*S,R*)-**L1**: 108 mg, 84% yield, > 20:1 dr, 96% ee.

HPLC analysis: The ee was determined on a CHIRALPAK AD-3 column (1% *i*-PrOH in hexane, 1.0 mL/min); retention times for compound obtained using (*R,S*)-**L1**: 3.9 min (major), 4.2 min (minor).

^1H NMR (600 MHz, $CDCl_3$) δ 4.99 (ddd, *J* = 8.7, 7.2, 1.8 Hz, 1H), 3.82 (d, *J* = 3.2 Hz, 1H), 2.02 – 1.89 (m, 2H), 1.88 – 1.79 (m, 1H), 1.68 – 1.61 (m, 1H), 1.50 (brs,

1H), 1.18 (tt, J = 13.9, 7.1 Hz, 3H), 1.08 (dd, J = 10.2, 7.3 Hz, 18H), 1.00 (d, J = 6.8 Hz, 3H), 0.92 (dd, J = 6.6, 5.2 Hz, 9H).

^{13}C NMR (151 MHz, $CDCl_3$) δ 206.1, 96.5, 87.9, 74.6, 38.0, 33.4, 29.1, 22.4, 22.2, 20.8, 18.7 (two carbons), 15.9, 11.7.

HRMS (ESI) m/z [M – H_2O + H]$^+$ calcd for $C_{20}H_{39}Si$: 307.2821, found: 307.2755.

$[\alpha]^{24}_D$ = –29.6 (c = 0.5, $CHCl_3$); 96% ee, from (R,S)-**L1**.

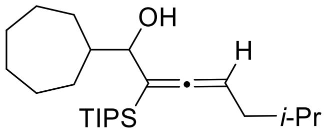

1-cycloheptyl-6-methyl-2-(triisopropylsilyl)hepta-2,3-dien-1-ol (Figure 3, entry 32). The title compound was prepared according to the **GP-3** from cycloheptanecarbaldehyde, enyne **1** and DHP ester **2**, purified by flash column chromatography on silica gel: 5→10% EtOAc in hexanes, colorless liquid.

(R,S)-**L1**: 99 mg, 66% yield, 20:1 dr, 97% ee; (S,R)-**L1**: 99 mg, 66% yield, 20:1 dr, 98% ee.

HPLC analysis: The ee was determined on a CHIRALCEL OD-3 column (0.5% i-PrOH in hexane, 1.0 mL/min); retention times for compound obtained using (R,S)-**L1**: 3.6 min (major), 4.0 min (minor).

^{1}H NMR (500 MHz, $CDCl_3$) δ 4.99 (ddd, J = 8.0, 7.2, 2.0 Hz, 1H), 3.92 (s, 1H), 2.02 – 1.88 (m, 2H), 1.83 (ddd, J = 13.7, 7.1, 3.4 Hz, 1H), 1.77 – 1.62 (m, 5H), 1.60 – 1.36 (m, 8H), 1.26 (dtd, J = 13.3, 10.3, 3.4 Hz, 1H), 1.22 – 1.12 (m, 3H), 1.08 (t, J = 7.1 Hz, 18H), 0.93 (dd, J = 6.7, 1.2 Hz, 6H).

^{13}C NMR (126 MHz, $CDCl_3$) δ 206.0, 96.8, 88.0, 74.8, 44.4, 38.2, 33.4, 29.1, 28.6, 28.2, 27.3, 26.8 (two carbons), 22.4, 22.2, 18.8, 18.7, 11.7.

HRMS (ESI) m/z [M – H_2O + H]$^+$ calcd for $C_{24}H_{45}Si$: 361.3290, found: .361.3287.

$[\alpha]^{24}_D$ = –50.8 (c = 0.5, $CHCl_3$); 97% ee, from (R,S)-**L1**.

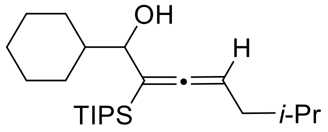

1-cyclohexyl-6-methyl-2-(triisopropylsilyl)hepta-2,3-dien-1-ol (Figure 3, entry 33). The title compound was prepared according to the **GP-3** from cyclohexanecarbaldehyde, enyne **1** and DHP ester **2**, purified by flash column chromatography on silica gel: 5→10% EtOAc in hexanes, colorless liquid.

(R,S)-**L1**: 123 mg, 85% yield, > 20:1 dr, 94% ee; (S,R)-**L1**: 123 mg, 85% yield, > 20:1 dr, 95% ee.

HPLC analysis: The ee was determined on a CHIRALCEL OD-3 column (1% i-PrOH in hexane, 1.0 mL/min); retention times for compound obtained using (R,S)-**L1**: 3.3 min (major), 3.6 min (minor).

^{1}H NMR (500 MHz, $CDCl_3$) δ 4.97 (ddd, *J* = 8.4, 7.1, 1.7 Hz, 1H), 3.78 (s, 1H), 2.00 – 1.85 (m, 3H), 1.83 – 1.72 (m, 2H), 1.64 (qd, *J* = 13.5, 6.9 Hz, 3H), 1.54 – 1.42 (m, 2H), 1.25 – 1.13 (m, 7H), 1.13 – 1.04 (m, 18H), 1.02 – 0.97 (m, 1H), 0.93 (d, *J* = 6.7 Hz, 6H).

^{13}C NMR (126 MHz, $CDCl_3$) δ 206.3, 95.8, 87.7, 74.2, 43.3, 37.9, 31.2, 29.1, 26.5 (three carbons), 26.2, 22.4, 22.3, 18.7 (two carbons), 11.7.

HRMS (ESI) m/z $[M - H_2O + H]^+$ calcd for $C_{23}H_{43}Si$: 347.3134, found: 347.3129.

$[\alpha]^{24}_D$ = –57.6 (*c* = 0.5, $CHCl_3$); 94% ee, from (*R,S*)-**L1**.

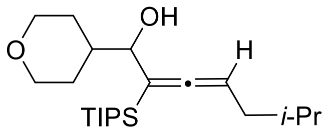

6-methyl-1-(tetrahydro-2H-pyran-4-yl)-2-(triisopropylsilyl)hepta-2,3-dien-1-ol (Figure 3, entry 34). The title compound was prepared according to the **GP-3** from tetrahydro-2*H*-pyran-4-carbaldehyde, enyne **1** and DHP ester **2**, purified by flash column chromatography on silica gel: 5→10% EtOAc in hexanes, colorless liquid.

(*R,S*)-**L1**: 117 mg, 80% yield, > 20:1 dr, 92% ee; (*S,R*)-**L1**: 117 mg, 80% yield, > 20:1 dr, 94% ee.

HPLC analysis: The ee was determined on a CHIRALCEL OD-3 column (5% *i*-PrOH in hexane, 1.0 mL/min); retention times for compound obtained using (*R,S*)-**L1**: 3.5 min (major), 4.0 min (minor).

^{1}H NMR (600 MHz, $CDCl_3$) δ 5.00 – 4.93 (m, 1H), 4.03 (dd, *J* = 11.3, 4.0 Hz, 1H), 3.98 (dd, *J* = 11.2, 2.9 Hz, 1H), 3.76 (s, 1H), 3.38 – 3.29 (m, 2H), 1.96 (dt, *J* = 13.6, 6.7 Hz, 1H), 1.93 – 1.84 (m, 1H), 1.82 – 1.73 (m, 2H), 1.63 (dp, *J* = 13.3, 6.7 Hz, 1H), 1.57 – 1.48 (m, 2H), 1.48 – 1.36 (m, 2H), 1.22 – 1.14 (m, 3H), 1.08 (dd, *J* = 10.2, 7.4 Hz, 18H), 0.92 (d, *J* = 6.7 Hz, 6H).

^{13}C NMR (151 MHz, $CDCl_3$) δ 206.9, 95.0, 87.6, 73.7, 68.1, 67.8, 40.8, 37.9, 30.8, 29.1, 27.7, 22.4, 22.2, 18.7, 11.6.

HRMS (ESI) m/z $[M - H_2O + H]^+$ calcd for $C_{22}H_{41}SiO$: 349.2927, found: 349.2924.

$[\alpha]^{24}_D$ = –33.2 (*c* = 0.5, $CHCl_3$); 92% ee, from (*R,S*)-**L1**.

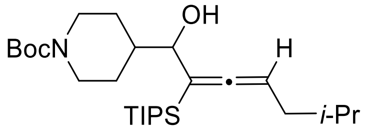

***tert*-butyl 4-(1-hydroxy-6-methyl-2-(triisopropylsilyl)hepta-2,3-dien-1-yl)piperidine-1-carboxylate (Figure 3, entry 35).** The title compound was prepared according to the **GP-3** from *tert*-butyl 4-formylpiperidine-1-carboxylate, enyne **1** and DHP ester **2**, purified by flash column chromatography on silica gel: 5→10% EtOAc in hexanes, colorless liquid.

(R,S)-**L1**: 158 mg, 85% yield, > 20:1 dr, 92% ee; (S,R)-**L1**: 158 mg, 85% yield, > 20:1 dr, 92% ee.

HPLC analysis: The ee was determined on a CHIRALCEL OD-3 column (5% *i*-PrOH in hexane, 1.0 mL/min); retention times for compound obtained using (R,S)-**L1**: 3.5 min (major), 4.8 min (minor).

^{1}H NMR (600 MHz, $CDCl_3$) δ 4.98 (t, J = 7.5 Hz, 1H), 4.14 (brs, 2H), 3.78 (s, 1H), 2.62 (brs, 2H), 2.00 – 1.83 (m, 3H), 1.73 – 1.57 (m, 4H), 1.45 (s, 9H), 1.33 (qd, J = 12.7, 4.4 Hz, 1H), 1.24 – 1.14 (m, 4H), 1.07 (dd, J = 11.1, 7.4 Hz, 18H), 0.93 (d, J = 6.7 Hz, 6H).

^{13}C NMR (151 MHz, $CDCl_3$) δ 206.7, 154.8, 95.4, 87.8, 79.2, 73.5, 44.3, 43.5, 41.9, 37.9, 30.0, 29.1, 28.5, 26.4, 22.4, 22.3, 18.7 (two carbons), 11.6.

HRMS (ESI) m/z [M – H_2O + H]$^+$ calcd for $C_{27}H_{50}SiNO_2$: 448.3611, found: 448.3606.

$[\alpha]^{24}_D$ = –23.2 (c = 0.5, $CHCl_3$); 92% ee, from (R,S)-**L1**.

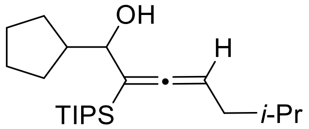

1-cyclopentyl-6-methyl-2-(triisopropylsilyl)hepta-2,3-dien-1-ol (Figure 3, entry 36). The title compound was prepared according to the **GP-3** from cyclopentanecarbaldehyde, enyne **1** and DHP ester **2**, purified by flash column chromatography on silica gel: 5→10% EtOAc in hexanes, colorless liquid.

(R,S)-**L1**: 100 mg, 72% yield, 20:1 dr, 92% ee; (S,R)-**L1**: 100 mg, 72% yield, 20:1 dr, 94% ee.

HPLC analysis: The ee was determined on a CHIRALCEL OD-3 column (1% *i*-PrOH in hexane, 1.0 mL/min); retention times for compound obtained using (R,S)-**L1**: 3.4 min (major), 3.7 min (minor).

^{1}H NMR (500 MHz, $CDCl_3$) δ 4.98 – 4.89 (m, 1H), 3.85 (d, J = 6.6 Hz, 1H), 2.24 – 2.14 (m, 1H), 2.02 – 1.88 (m, 2H), 1.78 – 1.58 (m, 5H), 1.57 – 1.40 (m, 4H)., 1.32 (ddd, J = 14.4, 11.8, 7.6 Hz, 1H), 1.25 – 1.14 (m, 3H), 1.09 (t, J = 7.1 Hz, 18H), 0.93 (dd, J = 6.7, 1.1 Hz, 6H).

^{13}C NMR (126 MHz, $CDCl_3$) δ 207.2, 96.5, 87.3, 73.7, 45.9, 38.0, 30.3, 29.1, 27.9, 25.9, 25.8, 22.4, 22.2, 18.7 (two carbons), 11.6.

HRMS (ESI) m/z [M – H_2O + H]$^+$ calcd for $C_{22}H_{41}Si$: 333.2978, found: 333.2967.

$[\alpha]^{24}_D$ = –30.4 (c = 0.5, $CHCl_3$); 92% ee, from (R,S)-**L1**.

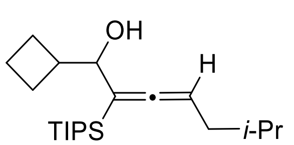

1-cyclobutyl-6-methyl-2-(triisopropylsilyl)hepta-2,3-dien-1-ol (Figure 3, entry 37). The title compound was prepared according to the **GP-3** from

cyclobutanecarbaldehyde, enyne **1** and DHP ester **2**, purified by flash column chromatography on silica gel: 5→10% EtOAc in hexanes, colorless liquid.

(*R,S*)-**L1**: 103 mg, 77% yield, > 20:1 dr, 92% ee; (*S,R*)-**L1**: 103 mg, 77% yield, > 20:1 dr, 92% ee.

HPLC analysis: The ee was determined on a CHIRALCEL OD-3 column (1% *i*-PrOH in hexane, 1.0 mL/min); retention times for compound obtained using (*R,S*)-**L1**: 3.5 min (major), 3.9 min (minor).

^{1}H NMR (600 MHz, $CDCl_3$) δ 4.92 (td, *J* = 7.5, 1.4 Hz, 1H), 3.92 (d, *J* = 6.1 Hz, 1H), 2.64 – 2.55 (m, 1H), 1.97 – 1.79 (m, 7H), 1.78 – 1.71 (m, 1H), 1.63 (dp, *J* = 13.4, 6.7 Hz, 1H), 1.49 (brs, 1H), 1.22 – 1.14 (m, 3H), 1.08 (dd, *J* = 7.5, 3.7 Hz, 18H), 0.95 – 0.90 (m, 6H).

^{13}C NMR (151 MHz, $CDCl_3$) δ 206.9, 94.9, 87.1, 72.8, 41.3, 38.3, 29.1, 24.8, 23.3, 22.3, 22.2, 18.7 (two carbons), 17.6, 11.6.

HRMS (ESI) m/z [M – H_2O + H]$^+$ calcd for $C_{21}H_{39}Si$: 319.2821, found: 319.2823.

$[\alpha]^{24}_{D}$ = –31.6 (*c* = 0.5, $CHCl_3$); 92% ee, from (*R,S*)-**L1**.

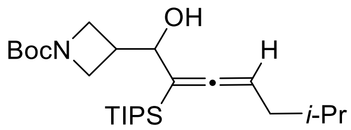

***tert*-butyl 3-(1-hydroxy-6-methyl-2-(triisopropylsilyl)hepta-2,3-dien-1-yl)azetidine-1-carboxylate (Figure 3, entry 38).** The title compound was prepared according to the **GP-3** from *tert*-butyl 3-formylazetidine-1-carboxylate, enyne **1** and DHP ester **2**, purified by flash column chromatography on silica gel: 5→10% EtOAc in hexanes, colorless liquid.

(*R,S*)-**L1**: 150 mg, 86% yield, 18:1 dr, 91% ee; (*S,R*)-**L1**: 150 mg, 86% yield, 18:1 dr, 91% ee.

HPLC analysis: The ee was determined on a CHIRALCEL OD-3 column (5% *i*-PrOH in hexane, 1.0 mL/min); retention times for compound obtained using (*R,S*)-**L1**: 3.4 min (major), 4.0 min (minor).

^{1}H NMR (600 MHz, $CDCl_3$) δ 4.96 (dd, *J* = 7.4, 6.5 Hz, 1H), 4.15 (d, *J* = 4.5 Hz, 1H), 3.89 (ddd, *J* = 15.0, 13.0, 7.6 Hz, 3H), 3.71 (s, 1H), 2.85 – 2.76 (m, 1H), 1.90 (t, *J* = 7.2 Hz, 2H), 1.73 (s, 1H), 1.64 (td, *J* = 13.4, 6.7 Hz, 1H), 1.44 (s, 9H), 1.23 – 1.16 (m, 3H), 1.08 (dd, *J* = 7.4, 3.2 Hz, 18H), 0.93 (dd, *J* = 8.2, 6.7 Hz, 6H).

^{13}C NMR (151 MHz, $CDCl_3$) δ 206.9, 156.4, 94.5, 87.7, 79.2, 71.0, 51.3, 38.1, 34.6, 29.7, 29.0, 28.4, 22.3 (two carbons), 18.7 (two carbons), 11.6.

HRMS (ESI) m/z [M + Na]$^+$ calcd for $C_{25}H_{47}SiNO_3Na$: 460.3223, found: 460.3212.

$[\alpha]^{24}_{D}$ = –7.6 (*c* = 0.5, $CHCl_3$); 91% ee, from (*R,S*)-**L1**.

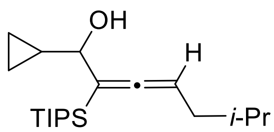

1-cyclopropyl-6-methyl-2-(triisopropylsilyl)hepta-2,3-dien-1-ol (Figure 3, entry 39). The title compound was prepared according to the **GP-3** from cyclopropanecarbaldehyde, enyne **1** and DHP ester **2**, purified by flash column chromatography on silica gel: 5→10% EtOAc in hexanes, colorless liquid.

(*R,S*)-**L1**: 90 mg, 70% yield, 20:1 dr, 90% ee; (*S,R*)-**L1**: 90 mg, 70% yield, 20:1 dr, 90% ee.

HPLC analysis: The ee was determined on a CHIRALCEL OD-3 column (3% *i*-PrOH in hexane, 1.0 mL/min); retention times for compound obtained using (*R,S*)-**L1**: 3.1 min (major), 3.5 min (minor).

^{1}H NMR (500 MHz, $CDCl_3$) δ 5.02 – 4.94 (m, 1H), 3.47 (d, *J* = 7.3 Hz, 1H), 2.04 – 1.87 (m, 2H), 1.65 (tt, *J* = 13.4, 6.7 Hz, 1H), 1.59 (brs, 1H), 1.24 – 1.14 (m, 4H), 1.09 (dd, *J* = 7.3, 5.4 Hz, 18H), 0.93 (d, *J* = 6.7 Hz, 6H), 0.53 – 0.45 (m, 2H), 0.41 – 0.34 (m, 1H), 0.32 – 0.27 (m, 1H).

^{13}C NMR (126 MHz, $CDCl_3$) δ 207.8, 96.8, 87.3, 73.9, 38.2, 29.1, 22.4, 22.3, 18.8, 18.7, 18.4, 11.7, 3.3, 2.7.

HRMS (ESI) m/z $[M – H_2O + H]^+$ calcd for $C_{20}H_{37}Si$: 305.2664, found: 305.2653.

$[\alpha]^{24}_D$ = –46.4 (*c* = 0.5, $CHCl_3$); 90% ee, from (*R,S*)-**L1**.

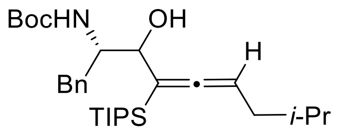

***tert*-butyl ((2S)-3-hydroxy-8-methyl-1-phenyl-4-(triisopropylsilyl)nona-4,5-dien-2-yl)carbamate (Figure 3, entry 40&41).** The title compound was prepared according to the **GP-3** from *tert*-butyl (*S*)-(1-oxo-3-phenylpropan-2-yl)carbamate, enyne **1** and DHP ester **2**, purified by flash column chromatography on silica gel: 10→15% EtOAc in hexanes.

(*R,S*)-**L1**: 140 mg, 70% yield, 99:1 dr; (*S,R*)-**L1**: 124 mg, 62% yield, 1:99 dr.

HPLC analysis: The dr was determined on a CHIRALPAK AD-3 column (5% *i*-PrOH in hexane, 1.0 mL/min); retention times for compound obtained using (*R,S*)-**L1**: 3.6 min (major), 6.3 min (minor).

NMR spectra for product from (*R,S*)-**L1**:

^{1}H NMR (500 MHz, C_6D_6, 60 °C) δ 7.41 – 7.31 (m, 2H), 7.22 (t, *J* = 7.6 Hz, 2H), 7.13 (t, *J* = 7.4 Hz, 1H), 5.00 (dd, *J* = 17.1, 8.5 Hz, 2H), 4.28 (s, 1H), 4.12 (s, 1H), 3.19 (s, 1H), 3.12 – 3.01 (m, 1H), 2.11 (t, *J* = 6.8 Hz, 2H), 1.98 (s, 1H), 1.77 – 1.61 (m, 1H), 1.52 (s, 9H), 1.20 – 1.09 (m, 21H), 1.05 (dd, *J* = 9.7, 6.7 Hz, 6H).

^{13}C NMR (126 MHz, C_6D_6, 60 °C) δ 205.9, 154.7, 138.3, 129.0, 127.6, 125.5, 95.5, 88.0, 77.6, 68.5, 67.6, 55.5, 37.4, 28.3, 27.6, 21.5, 21.2, 17.9, 11.0.

NMR spectra for product from (*S,R*)-**L1**:

^1H NMR (500 MHz, C_6D_6, 60 °C) δ 7.27 (d, *J* = 7.3 Hz, 2H), 7.18 (s, 1H), 7.15 (s, 1H), 7.06 (t, *J* = 7.4 Hz, 1H), 4.90 (td, *J* = 7.5, 2.5 Hz, 1H), 4.74 (brs, 1H), 4.48 (s, 1H), 4.21 (t, *J* = 9.4 Hz, 1H), 3.20 (dd, *J* = 14.2, 3.4 Hz, 1H), 2.86 (s, 1H), 2.55 (brs, 1H), 2.01 – 1.91 (m, 2H), 1.59 (dp, *J* = 13.3, 6.7 Hz, 1H), 1.40 – 1.24 (m, 12H), 1.19 (dd, *J* = 7.4, 4.3 Hz, 18H), 0.88 (dd, *J* = 6.6, 4.4 Hz, 6H).

^{13}C NMR (126 MHz, C_6D_6, 60 °C) δ 206.0, 154.9, 138.6, 128.7, 127.6, 125.4, 95.0, 86.8, 78.0, 72.1, 56.3, 37.3, 28.3, 27.4, 21.4, 21.3, 18.1 (two carbons), 11.3.

HRMS (ESI) m/z $[M - H_2O + H]^+$ calcd for $C_{30}H_{50}SiNO_2$: 484.3611, found: 484.3609.

$[\alpha]^{24}_D$ = –40.8 (*c* = 0.5, $CHCl_3$); 99:1 dr, from (*R,S*)-**L1**.

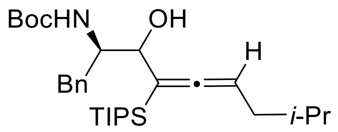

***tert*-butyl ((2*R*)-3-hydroxy-8-methyl-1-phenyl-4-(triisopropylsilyl)nona-4,5-dien-2-yl)carbamate (Figure 3, entry 42&43).** The title compound was prepared according to the **GP-3** from *tert*-butyl (*R*)-(1-oxo-3-phenylpropan-2-yl)carbamate, enyne **1** and DHP ester **2**, purified by flash column chromatography on silica gel: 10→15% EtOAc in hexanes.

(*R,S*)-**L1**: 128 mg, 64% yield, 99:1 dr; (*S,R*)-**L1**: 140 mg, 70% yield, 1:99 dr.

HPLC analysis: The dr was determined on a CHIRALPAK IC-3 column (5% *i*-PrOH in hexane, 1.0 mL/min); retention times for compound obtained using (*R,S*)-**L1**: 4.2 min (major), 3.8 min (minor).

NMR spectra for product from (*R,S*)-**L1**:

^1H NMR (500 MHz, C_6D_6, 60 °C) δ 7.27 (d, *J* = 7.4 Hz, 2H), 7.18 (s, 1H), 7.15 (s, 1H), 7.06 (t, *J* = 7.4 Hz, 1H), 4.90 (td, *J* = 7.5, 2.5 Hz, 1H), 4.75 (brs, 1H), 4.49 (s, 1H), 4.21 (t, *J* = 9.5 Hz, 1H), 3.20 (dd, *J* = 14.2, 3.5 Hz, 1H), 2.86 (s, 1H), 2.57 (brs, 1H), 1.97 (dd, *J* = 11.0, 4.6 Hz, 2H), 1.59 (dp, *J* = 13.4, 6.7 Hz, 1H), 1.39 – 1.28 (m, 12H), 1.19 (dd, *J* = 7.4, 4.4 Hz, 18H), 0.88 (dd, *J* = 6.6, 4.4 Hz, 6H).

^{13}C NMR (126 MHz, C_6D_6, 60 °C) δ 206.0, 155.0, 138.6, 128.7, 127.6, 125.4, 95.0, 86.8, 78.0, 72.1, 56.3, 37.3, 28.3, 27.4, 21.4, 21.3, 18.1 (two carbons), 11.3.

NMR spectra for product from (*S,R*)-**L1**:

^1H NMR (500 MHz, C_6D_6, 60 °C) δ 7.31 (d, *J* = 7.3 Hz, 2H), 7.18 – 7.14 (m, 2H), 7.06 (t, *J* = 7.4 Hz, 1H), 5.01 – 4.88 (m, 2H), 4.21 (s, 1H), 4.04 (brs, 1H), 3.12 (s, 1H), 3.00 (dd, *J* = 12.7, 9.2 Hz, 1H), 2.04 (t, *J* = 6.8 Hz, 2H), 1.98 (brs, 1H), 1.64 (dp, *J* = 12.9, 6.5 Hz, 1H), 1.45 (s, 9H), 1.13 – 1.02 (m, 21H), 0.98 (dd, *J* = 9.7, 6.7 Hz, 6H).

^{13}C NMR (126 MHz, C_6D_6, 60 °C) δ 206.0, 154.7, 138.3, 129.0, 127.6, 125.5, 95.5, 87.9, 77.6, 67.6, 55.5, 39.1, 37.4, 28.3, 27.6, 21.6, 21.2, 17.9, 11.0.

HRMS (ESI) m/z $[M - H_2O + H]^+$ calcd for $C_{30}H_{50}SiNO_2$: 484.3611, found: 484.3610.

$[\alpha]^{24}_{D}$ = –17.6 (*c* = 0.5, $CHCl_3$); 99:1 dr, from (*R,S*)-**L1**.

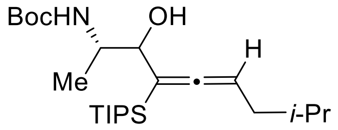

***tert*-butyl ((2*S*)-3-hydroxy-8-methyl-4-(triisopropylsilyl)nona-4,5-dien-2-yl)carbamate (Figure 3, entry 44&45).** The title compound was prepared according to the **GP-3** from *tert*-butyl (*S*)-(1-oxopropan-2-yl)carbamate, enyne **1** and DHP ester **2**, purified by flash column chromatography on silica gel: 10→15% EtOAc in hexanes.

(*R,S*)-**L1**: 113 mg, 67% yield, 99:1 dr; (*S,R*)-**L1**: 102 mg, 60% yield, 1:99 dr.

HPLC analysis: The dr was determined on a CHIRALPAK AD-3 column (5% *i*-PrOH in hexane, 1.0 mL/min); retention times for compound obtained using (*R,S*)-**L1**: 3.2 min (major), 4.1 min (minor).

NMR spectra for product from (*R,S*)-**L1**:

^{1}H NMR (500 MHz, C_6D_6, 60 °C) δ 4.86 (td, *J* = 7.5, 1.4 Hz, 1H), 4.75 (d, *J* = 7.4 Hz, 1H), 3.98 – 3.96 (m, 2H), 2.12 (s, 1H), 1.97 (t, *J* = 7.1 Hz, 2H), 1.63 – 1.53 (m, 1H), 1.44 (s, 9H), 1.27 – 1.20 (m, 6H), 1.15 (d, *J* = 7.4 Hz, 18H), 0.93 (t, *J* = 6.9 Hz, 6H).

^{13}C NMR (126 MHz, C_6D_6, 60 °C) δ 206.2, 155.0, 95.1, 87.1, 77.6, 72.3, 50.0, 37.2, 28.3, 27.6, 21.5, 21.2, 18.5, 18.0 (two carbons), 11.2.

NMR spectra for product from (*S,R*)-**L1**:

^{1}H NMR (500 MHz, C_6D_6, 60 °C) δ 4.95 (d, *J* = 8.5 Hz, 1H), 4.84 (td, *J* = 7.6, 2.6 Hz, 1H), 4.36 (s, 1H), 4.07 (dd, *J* = 8.1, 5.7 Hz, 1H), 2.02 (d, *J* = 5.1 Hz, 1H), 1.92 – 1.81 (m, 2H), 1.57 – 1.49 (m, 1H), 1.44 (s, 9H), 1.32 – 1.22 (m, 3H), 1.17 – 1.11 (m, 21H), 0.86 (dd, *J* = 6.7, 2.4 Hz, 6H).

^{13}C NMR (126 MHz, C_6D_6, 60 °C) δ 206.2, 155.1, 95.7, 87.8, 78.4, 72.2, 50.7, 37.7, 28.8, 28.1, 22.0, 21.8, 18.6 (two carbons), 13.5, 11.8.

HRMS (ESI) m/z $[M - H_2O + H]^+$ calcd for $C_{24}H_{36}SiNO_2$: 408.3298, found: 408.3294.

$[\alpha]^{24}_{D}$ = –42.4 (*c* = 0.5, $CHCl_3$); 99:1 dr, from (*R,S*)-**L1**.

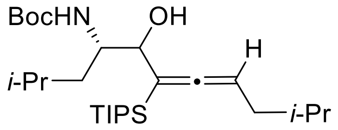

***tert*-butyl ((4*S*)-5-hydroxy-2,10-dimethyl-6-(triisopropylsilyl)undeca-6,7-dien-4-yl)carbamate (Figure 3, entry 46&47).** The title compound was prepared according to the **GP-3** from *tert*-butyl (*S*)-(4-methyl-1-oxopentan-2-yl)carbamate, enyne **1** and DHP ester **2**, purified by flash column chromatography on silica gel: 10→15% EtOAc in hexanes.

(R,S)-**L1**: 128 mg, 69% yield, 99:1 dr; (S,R)-**L1**: 108 mg, 58% yield, 1:99 dr.

HPLC analysis: The dr was determined on a CHIRALPAK AD-3 column (5% *i*-PrOH in hexane, 1.0 mL/min); retention times for compound obtained using (R,S)-**L1**: 3.0 min (major), 4.5 min (minor).

NMR spectra for product from (R,S)-**L1**:

^{1}H NMR (500 MHz, C_6D_6, 60 °C) δ 4.90 (td, J = 7.4, 1.9 Hz, 1H), 4.60 (d, J = 8.9 Hz, 1H), 4.08 (s, 1H), 3.96 (s, 1H), 2.02 (t, J = 6.9 Hz, 3H), 1.75 (dddd, J = 13.2, 11.5, 6.9, 4.5 Hz, 1H), 1.65 – 1.54 (m, 2H), 1.44 (s, 9H), 1.39 – 1.34 (m, 1H), 1.29 – 1.24 (m, 3H), 1.17 (dd, J = 7.4, 2.1 Hz, 18H), 1.03 (d, J = 6.5 Hz, 3H), 0.95 (t, J = 6.7 Hz, 9H).

^{13}C NMR (126 MHz, C_6D_6, 60 °C) δ 205.8, 155.1, 95.6, 87.7, 77.5, 71.5, 52.1, 43.0, 37.3, 28.3, 27.6, 24.4, 22.8, 21.5, 21.4, 21.2, 18.1, 18.0, 11.2.

NMR spectra for product from (S,R)-**L1**:

^{1}H NMR (500 MHz, C_6D_6, 60 °C) δ 4.86 (td, J = 7.5, 2.3 Hz, 2H), 4.38 (s, 1H), 4.06 (dd, J = 14.0, 6.9 Hz, 1H), 2.09 (d, J = 5.8 Hz, 1H), 1.94 (dd, J = 10.7, 4.1 Hz, 2H), 1.80 – 1.69 (m, 1H), 1.58 (dt, J = 13.4, 6.7 Hz, 1H), 1.53 – 1.49 (m, 1H), 1.44 (s, 9H), 1.31 (dq, J = 14.1, 7.1 Hz, 3H), 1.18 (dd, J = 7.4, 1.4 Hz, 18H), 1.03 (d, J = 6.5 Hz, 3H), 0.95 (d, J = 6.7 Hz, 3H), 0.89 (t, J = 6.6 Hz, 6H).

^{13}C NMR (126 MHz, C_6D_6, 60 °C) δ 205.9, 155.0, 95.0, 86.9, 77.8, 72.5, 53.0, 37.3, 36.4, 28.3, 27.5, 24.3, 23.2, 21.4 (two carbons), 21.1, 18.1 (two carbons), 11.2.

HRMS (ESI) m/z [M – H_2O + H]$^+$ calcd for $C_{27}H_{52}SiNO_2$: 450.3767, found: 450.3768.

$[\alpha]^{24}_{D}$ = –61.6 (c = 0.5, $CHCl_3$); 99:1 dr, from (R,S)-**L1**.

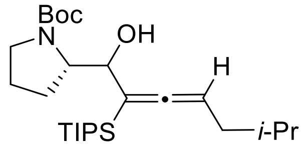

***tert*-butyl (2*S*)-2-(1-hydroxy-6-methyl-2-(triisopropylsilyl)hepta-2,3-dien-1-yl)pyrrolidine-1-carboxylate (Figure 3, entry 48&49).** The title compound was prepared according to the **GP-3** from *tert*-butyl (S)-2-formylpyrrolidine-1-carboxylate, enyne **1** and DHP ester **2**, purified by flash column chromatography on silica gel: 10→15% EtOAc in hexanes.

(R,S)-**L1**: 93 mg, 52% yield, 99:1 dr; (S,R)-**L1**: 81 mg, 45% yield, 1:99 dr.

HPLC analysis: The dr was determined on a CHIRALPAK AD-3 column (5% *i*-PrOH in hexane, 1.0 mL/min); retention times for compound obtained using (R,S)-**L1**: 3.2 min (major), 3.5 min (minor).

NMR spectra for product from (R,S)-**L1**:

^{1}H NMR (500 MHz, C_6D_6, 60 °C) δ 4.88 (brs, 1H), 4.77 (td, J = 7.4, 3.0 Hz, 1H), 4.24 – 4.14 (m, 1H), 3.47 (s, 1H), 3.32 (dt, J = 10.6, 6.9 Hz, 1H), 2.05 (dt, J = 12.6, 6.3 Hz, 1H), 1.98 – 1.86 (m, 2H), 1.78 – 1.64 (m, 2H), 1.63 – 1.51 (m, 1H), 1.48 (s, 9H), 1.43 – 1.33 (m, 5H), 1.23 (dd, J = 11.2, 7.4 Hz, 18H), 0.88 (d, J = 6.6 Hz, 6H).

^{13}C NMR (126 MHz, C_6D_6, 60 °C) δ 206.2, 154.0, 94.0, 85.2, 78.0, 70.2, 61.7, 47.1, 37.5, 28.4, 27.7, 25.4, 23.7, 21.5, 21.3, 18.2 (two carbons), 11.3.

NMR spectra for product from (*S,R*)-**L1**:

^1H NMR (500 MHz, C_6D_6, 60 °C) δ 4.89 (s, 1H), 4.79 (td, *J* = 7.4, 2.8 Hz, 1H), 4.19 – 4.10 (m, 1H), 3.48 (s, 1H), 3.36 (dt, *J* = 10.5, 7.0 Hz, 1H), 2.11 – 2.00 (m, 1H), 1.97 – 1.86 (m, 2H), 1.82 – 1.65 (m, 2H), 1.56 (td, *J* = 13.3, 6.6 Hz, 1H), 1.47 (s, 9H), 1.42 – 1.32 (m, 4H), 1.21 (dd, *J* = 9.5, 7.5 Hz, 18H), 0.88 (d, *J* = 6.7 Hz, 6H).

^{13}C NMR (126 MHz, C_6D_6, 60 °C) δ 206.1, 153.9, 94.4, 85.6, 77.9, 70.0, 61.3, 47.1, 37.4, 28.3, 27.7, 24.9, 23.7, 21.5, 21.3, 18.2 (two carbons), 11.4.

HRMS (ESI) m/z [M – H_2O + H]$^+$ calcd for $C_{26}H_{48}SiNO_2$: 434.3454, found: 434.3459.

$[\alpha]^{24}_D$ = –101.6 (*c* = 0.5, $CHCl_3$); 99:1 dr, from (*R,S*)-**L1**.

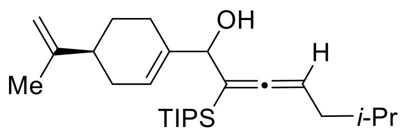

6-methyl-1-((*S*)-4-(prop-1-en-2-yl)cyclohex-1-en-1-yl)-2-(triisopropylsilyl)hepta-2,3-dien-1-ol (Figure 3, entry 50&51). The title compound was prepared according to the **GP-3** from (*S*)-4-(prop-1-en-2-yl)cyclohex-1-ene-1-carbaldehyde, enyne **1** and DHP ester **2**, purified by flash column chromatography on silica gel: 5→10% EtOAc in hexanes.

(*R,S*)-**L1**: 137 mg, 85% yield, 99:1 dr; (*S,R*)-**L1**: 138 mg, 86% yield, 1:99 dr.

HPLC analysis: The dr was determined on a CHIRALCEL OD-3 column (1% *i*-PrOH in hexane, 1.0 mL/min); retention times for compound obtained using (*R,S*)-**L1**: 3.6 min (major), 3.8 min (minor).

NMR spectra for product from (*R,S*)-**L1**:

^1H NMR (500 MHz, $CDCl_3$) δ 5.73 (d, *J* = 2.2 Hz, 1H), 5.07 (td, *J* = 7.7, 2.1 Hz, 1H), 4.71 (dd, *J* = 6.8, 1.1 Hz, 2H), 4.48 (s, 1H), 2.22 – 1.83 (m, 9H), 1.73 (s, 3H), 1.66 (dt, *J* = 13.3, 6.7 Hz, 1H), 1.42 (ddd, *J* = 24.1, 11.7, 5.5 Hz, 1H), 1.21 – 1.13 (m, 3H), 1.08 (d, *J* = 7.2 Hz, 9H), 1.05 (d, *J* = 7.3 Hz, 9H), 0.94 (d, *J* = 6.6 Hz, 6H).

^{13}C NMR (126 MHz, $CDCl_3$) δ 205.8, 150.0, 138.4, 123.9, 108.5, 96.0, 88.6, 74.2, 41.2, 38.2, 30.6, 29.1, 27.5, 23.6, 22.4, 22.2, 20.8, 18.7, 18.6, 11.6.

NMR spectra for product from (*S,R*)-**L1**:

^1H NMR (500 MHz, $CDCl_3$) δ 5.72 (s, 1H), 5.08 (td, *J* = 7.5, 2.0 Hz, 1H), 4.71 (s, 2H), 4.47 (s, 1H), 2.21 – 1.82 (m, 9H), 1.73 (s, 3H), 1.66 (tt, *J* = 13.4, 6.8 Hz, 1H), 1.46 (qd, *J* = 11.9, 5.4 Hz, 1H), 1.21 – 1.13 (m, 3H), 1.09 (d, *J* = 7.2 Hz, 9H), 1.05 (d, *J* = 7.3 Hz, 9H), 0.94 (dd, *J* = 6.6, 1.6 Hz, 6H).

^{13}C NMR (126 MHz, $CDCl_3$) δ 205.8, 149.9, 138.5, 124.4, 108.6, 95.8, 88.7, 73.9, 41.2, 38.2, 30.7, 29.1, 27.7, 23.6, 22.4, 22.3, 20.7, 18.8, 18.6, 11.7.

HRMS (ESI) m/z [M – H_2O + H]$^+$ calcd for $C_{26}H_{45}Si$: 385.3290, found: 385.3307.

$[\alpha]^{24}_{D}$ = –98.4 (c = 0.5, $CHCl_3$); 99:1 dr, from (*R,S*)-**L1**.

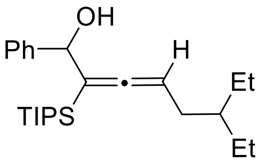

6-ethyl-1-phenyl-2-(triisopropylsilyl)octa-2,3-dien-1-ol (Figure 4, entry 52). The title compound was prepared according to the **GP-3** from benzaldehyde, enyne **1** and DHP ester **S1**, purified by flash column chromatography on silica gel: 5→10% EtOAc in hexanes, colorless liquid.

(*R,S*)-**L1**: 137 mg, 89% yield, 20:1 dr, 95% ee; (*S,R*)-**L1**: 137 mg, 89% yield, 20:1 dr, 94% ee.

HPLC analysis: The ee was determined on a CHIRALCEL OD-3 column (5% *i*-PrOH in hexane, 1.0 mL/min); retention times for compound obtained using (*R,S*)-**L1**: 3.5 min (major), 4.4 min (minor).

^{1}H NMR (500 MHz, $CDCl_3$) δ 7.39 – 7.35 (m, 2H), 7.31 (t, *J* = 7.5 Hz, 2H), 7.26 – 7.22 (m, 1H), 5.16 – 5.11 (m, 1H), 5.10 – 5.04 (m, 1H), 2.31 (d, *J* = 6.0 Hz, 1H), 2.07 – 1.93 (m, 2H), 1.31 – 1.23 (m, 4H), 1.22 – 1.10 (m, 4H), 1.06 (d, *J* = 7.0 Hz, 9H), 0.93 (d, *J* = 7.2 Hz, 9H), 0.84 (td, *J* = 7.4, 1.2 Hz, 6H).

^{13}C NMR (126 MHz, $CDCl_3$) δ 206.7, 143.6, 128.2, 127.6, 127.3, 98.1, 88.7, 72.8, 41.5, 32.2, 25.4, 25.3, 18.7, 18.5, 11.7, 11.1, 11.0.

HRMS (ESI) m/z $[M – H_2O + H]^+$ calcd for $C_{25}H_{41}Si$: 369.2978, found: 369.2933.

$[\alpha]^{24}_{D}$ = –129.6 (c = 0.5, $CHCl_3$); 95% ee, from (*R,S*)-**L1**.

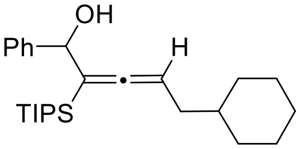

5-cyclohexyl-1-phenyl-2-(triisopropylsilyl)penta-2,3-dien-1-ol (Figure 4, entry 53). The title compound was prepared according to the **GP-3** from benzaldehyde, enyne **1** and DHP ester **S2**, purified by flash column chromatography on silica gel: 5→10% EtOAc in hexanes, colorless liquid.

(*R,S*)-**L1**: 119 mg, 75% yield, 18:1 dr, 95% ee; (*S,R*)-**L1**: 119 mg, 75% yield, 18:1 dr, 94% ee.

HPLC analysis: The ee was determined on a CHIRALCEL OD-3 column (5% *i*-PrOH in hexane, 1.0 mL/min); retention times for compound obtained using (*R,S*)-**L1**: 3.7 min (major), 5.3 min (minor).

^{1}H NMR (500 MHz, $CDCl_3$) δ 7.39 – 7.34 (m, 2H), 7.31 (dd, *J* = 10.2, 4.8 Hz, 2H), 7.24 (ddd, *J* = 7.3, 4.0, 1.3 Hz, 1H), 5.12 (s, 1H), 5.06 (ddd, *J* = 9.1, 7.1, 2.1 Hz, 1H), 2.34 (s, 1H), 2.01 – 1.83 (m, 2H), 1.71 – 1.62 (m, 5H), 1.23 – 1.09 (m, 7H), 1.06 (d, *J* = 7.3 Hz, 9H), 0.93 (t, *J* = 8.8 Hz, 9H), 0.91 – 0.78 (m, 2H).

^{13}C NMR (126 MHz, $CDCl_3$) δ 206.6, 143.6, 128.1, 127.5, 127.1, 98.0, 88.7, 72.7,

38.5, 36.6, 33.1, 33.0, 26.4, 26.3, 26.2, 18.7, 18.4, 11.6.

HRMS (ESI) m/z $[M - H_2O + H]^+$ calcd for $C_{26}H_{41}Si$: 381.2978, found: 381.3005.

$[\alpha]^{24}_D = -139.6$ (c = 0.5, $CHCl_3$); 95% ee, from (*R,S*)-**L1**.

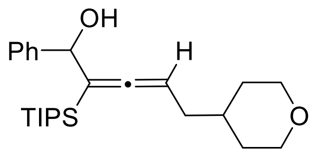

1-phenyl-5-(tetrahydro-2*H*-pyran-4-yl)-2-(triisopropylsilyl)penta-2,3-dien-1-ol (Figure 4, entry 54). The title compound was prepared according to the **GP-3** from benzaldehyde, enyne **1** and DHP ester **S3**, purified by flash column chromatography on silica gel: 10→15% EtOAc in hexanes, colorless liquid.

(*R,S*)-**L1**: 75 mg, 47% yield, 15:1 dr, 93% ee; (*S,R*)-**L1**: 75 mg, 47% yield, 15:1 dr, 92% ee.

HPLC analysis: The ee was determined on a CHIRALPAK AD-3 column (3% *i*-PrOH in hexane, 1.0 mL/min); retention times for compound obtained using (*R,S*)-**L1**: 9.9 min (major), 10.7 min (minor).

^{1}H NMR (500 MHz, $CDCl_3$) δ 7.37 – 7.33 (m, 2H), 7.33 – 7.28 (m, 2H), 7.24 (ddt, J = 5.2, 3.9, 2.0 Hz, 1H), 5.16 (s, 1H), 4.97 (ddd, J = 8.8, 7.0, 2.1 Hz, 1H), 3.94 – 3.83 (m, 2H), 3.29 (tdd, J = 11.7, 6.7, 2.1 Hz, 2H), 2.34 (d, J = 4.4 Hz, 1H), 1.91 (ddq, J = 14.1, 8.1, 7.0 Hz, 2H), 1.54 – 1.43 (m, 2H), 1.40 – 1.30 (m, 1H), 1.24 – 1.11 (m, 5H), 1.07 (d, J = 7.3 Hz, 9H), 0.97 (d, J = 7.3 Hz, 9H).

^{13}C NMR (126 MHz, $CDCl_3$) δ 207.3, 143.6, 128.1, 127.5, 127.0, 98.3, 87.1, 72.8, 67.9 (two carbons), 36.0, 35.7, 32.7, 32.6, 18.6, 18.5, 11.6.

HRMS (ESI) m/z $[M + Na]^+$ calcd for $C_{25}H_{40}SiO_2Na$: 423.2695, found: 423.2695.

$[\alpha]^{24}_D = -110.4$ (c = 0.5, $CHCl_3$); 93% ee, from (*R,S*)-**L1**.

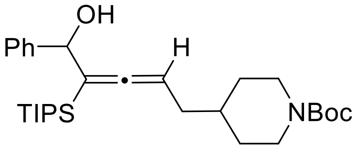

***tert*-butyl 4-(5-hydroxy-5-phenyl-4-(triisopropylsilyl)penta-2,3-dien-1-yl)piperidine-1-carboxylate (Figure 4, entry 55).** The title compound was prepared according to the **GP-3** from benzaldehyde, enyne **1** and DHP ester **S4**, purified by flash column chromatography on silica gel: 10→15% EtOAc in hexanes, colorless liquid.

(*R,S*)-**L1**: 93 mg, 47% yield, 15:1 dr, 92% ee; (*S,R*)-**L1**: 93 mg, 47% yield, 15:1 dr, 92% ee.

HPLC analysis: The ee was determined on a CHIRALPAK AD-3 column (3% *i*-PrOH in hexane, 1.0 mL/min); retention times for compound obtained using (*R,S*)-**L1**: 14.3 min (major), 11.3 min (minor).

^{1}H NMR (500 MHz, $CDCl_3$) δ 7.37 – 7.32 (m, 2H), 7.32 – 7.28 (m, 2H), 7.26 – 7.22 (m, 1H), 5.16 (s, 1H), 4.99 – 4.92 (m, 1H), 4.02 (s, 2H), 2.59 (s, 2H), 2.26 (s, 1H), 1.98 – 1.81 (m, 2H), 1.59 – 1.48 (m, 2H), 1.45 (s, 9H), 1.25 (ddt, *J* = 14.8, 11.4, 3.8 Hz, 1H), 1.19 – 1.10 (m, 3H), 1.07 (d, *J* = 7.3 Hz, 9H), 0.97 (d, *J* = 7.3 Hz, 9H).

^{13}C NMR (126 MHz, $CDCl_3$) δ 207.3, 154.8, 143.6, 128.1, 127.5, 127.0, 98.4, 87.2, 79.2, 72.8, 43.9, 43.6, 36.8, 35.6, 31.7, 29.6, 28.4, 18.7, 18.5, 11.6.

HRMS (ESI) m/z $[M + Na]^+$ calcd for $C_{30}H_{49}SiNO_3Na$: 522.3380, found: 522.3381.

$[\alpha]^{24}_D$ = –91.6 (*c* = 0.5, $CHCl_3$); 92% ee, from (*R,S*)-**L1**.

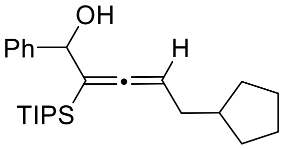

5-cyclopentyl-1-phenyl-2-(triisopropylsilyl)penta-2,3-dien-1-ol (Figure 4, entry 56). The title compound was prepared according to the **GP-3** from benzaldehyde, enyne **1** and DHP ester **S5**, purified by flash column chromatography on silica gel: 5→10% EtOAc in hexanes, colorless liquid.

(*R,S*)-**L1**: 119 mg, 78% yield, 16:1 dr, 95% ee; (*S,R*)-**L1**: 119 mg, 78% yield, 16:1 dr, 95% ee.

HPLC analysis: The ee was determined on a CHIRALCEL OD-3 column (5% *i*-PrOH in hexane, 1.0 mL/min); retention times for compound obtained using (*R,S*)-**L1**: 3.7 min (major), 4.9 min (minor).

^{1}H NMR (500 MHz, $CDCl_3$) δ 7.39 – 7.35 (m, 2H), 7.31 (dd, *J* = 10.2, 4.8 Hz, 2H), 7.26 – 7.21 (m, 1H), 5.13 (s, 1H), 5.08 (td, *J* = 7.7, 2.1 Hz, 1H), 2.35 (s, 1H), 2.10 – 1.97 (m, 2H), 1.85 – 1.65 (m, 3H), 1.64 – 1.45 (m, 4H), 1.18 – 1.09 (m, 5H), 1.06 (d, *J* = 7.2 Hz, 9H), 0.93 (d, *J* = 7.3 Hz, 9H).

^{13}C NMR (126 MHz, $CDCl_3$) δ 206.4, 143.6, 128.1, 127.5, 127.2, 98.1, 89.6, 72.6, 40.6, 35.0, 32.3, 32.2, 25.2, 25.1, 18.7, 18.4, 11.6.

HRMS (ESI) m/z $[M – H_2O + H]^+$ calcd for $C_{25}H_{39}Si$: 367.2821, found: 367.2795.

$[\alpha]^{24}_D$ = –150.4 (*c* = 0.5, $CHCl_3$); 95% ee, from (*R,S*)-**L1**.

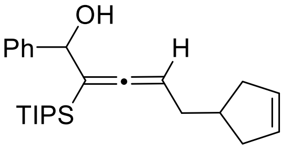

5-(cyclopent-3-en-1-yl)-1-phenyl-2-(triisopropylsilyl)penta-2,3-dien-1-ol (Figure 4, entry 57). The title compound was prepared according to the **GP-3** from benzaldehyde, enyne **1** and DHP ester **S8**, purified by flash column chromatography on silica gel: 5→10% EtOAc in hexanes, colorless liquid.

(*R,S*)-**L1**: 99 mg, 65% yield, 16:1 dr, 92% ee; (*S,R*)-**L1**: 99 mg, 65% yield, 16:1 dr, 94% ee.

HPLC analysis: The ee was determined on a CHIRALCEL OD-3 column (5% *i*-PrOH in hexane, 1.0 mL/min); retention times for compound obtained using (*R,S*)-**L1**: 3.9 min (major), 5.1 min (minor).

^{1}H NMR (500 MHz, $CDCl_3$) δ 7.37 (d, *J* = 7.2 Hz, 2H), 7.31 (t, *J* = 7.5 Hz, 2H), 7.24 (dd, *J* = 10.1, 4.2 Hz, 1H), 5.65 – 5.60 (m, 2H), 5.14 (s, 1H), 5.08 – 5.02 (m, 1H), 2.49 – 2.36 (m, 2H), 2.31 (brs, 1H), 2.28 – 2.19 (m, 1H), 2.17 – 2.02 (m, 2H), 2.01 – 1.90 (m, 2H), 1.18 – 1.09 (m, 3H), 1.06 (d, *J* = 7.2 Hz, 9H), 0.94 (d, *J* = 7.3 Hz, 9H).

^{13}C NMR (126 MHz, $CDCl_3$) δ 206.8, 143.5, 129.8, 129.7, 128.2, 127.6, 127.2, 98.2, 89.0, 72.7, 38.6, 38.4, 37.6, 35.4, 18.7, 18.5, 11.7.

HRMS (ESI) m/z $[M – H_2O + H]^+$ calcd for $C_{25}H_{37}Si$: 365.2664, found: 365.2664.

$[\alpha]^{24}_D$ = –130.0 (*c* = 0.5, $CHCl_3$); 92% ee, from (*R,S*)-**L1**.

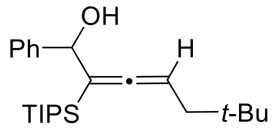

6,6-dimethyl-1-phenyl-2-(triisopropylsilyl)hepta-2,3-dien-1-ol (Figure 4, entry 58). The title compound was prepared according to the **GP-3** from benzaldehyde, enyne **1** and DHP ester **S6**, purified by flash column chromatography on silica gel: 5→10% EtOAc in hexanes, colorless liquid.

(*R,S*)-**L1**: 132 mg, 89% yield, >20:1 dr, 96% ee; (*S,R*)-**L1**: 130 mg, 88% yield, >20:1 dr, 96% ee.

HPLC analysis: The ee was determined on a CHIRALCEL OD-3 column (5% *i*-PrOH in hexane, 1.0 mL/min); retention times for compound obtained using (*R,S*)-**L1**: 3.4 min (major), 3.9 min (minor).

^{1}H NMR (500 MHz, $CDCl_3$) δ 7.40 – 7.34 (m, 2H), 7.31 (dd, *J* = 10.2, 4.8 Hz, 2H), 7.27 – 7.21 (m, 1H), 5.15 – 5.09 (m, 2H), 2.33 (s, 1H), 2.03 – 1.90 (m, 2H), 1.16 – 1.09 (m, 3H), 1.06 (d, *J* = 7.0 Hz, 9H), 0.92 (d, *J* = 7.2 Hz, 9H), 0.88 (s, 9H).

^{13}C NMR (126 MHz, $CDCl_3$) δ 206.8, 143.5, 128.2, 127.6, 127.3, 97.6, 87.1, 72.7, 43.3, 31.0, 29.2, 18.7, 18.4, 11.6.

HRMS (ESI) m/z $[M – H_2O + H]^+$ calcd for $C_{24}H_{39}Si$: 355.2821, found: 355.2810.

$[\alpha]^{24}_D$ = –154.8 (*c* = 0.5, $CHCl_3$); 96% ee, from (*R,S*)-**L1**.

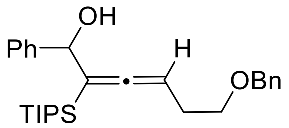

6-(benzyloxy)-1-phenyl-2-(triisopropylsilyl)hexa-2,3-dien-1-ol (Figure 4, entry 59a). The title compound was prepared according to the **GP-3** from benzaldehyde, enyne **1** and DHP ester **S7**, purified by flash column chromatography on silica gel: 5→10% EtOAc in hexanes, colorless liquid.

(*R,S*)-**L1**: 73 mg, 42% yield, >20:1 dr, 84% ee; (*S,R*)-**L1**: 72 mg, 42% yield, >20:1

dr, 86% ee.

HPLC analysis: The ee was determined on a CHIRALCEL OD-3 column (10% *i*-PrOH in hexane, 1.0 mL/min); retention times for compound obtained using (*R,S*)-**L1**: 4.8 min (major), 6.2 min (minor).

^{1}H NMR (500 MHz, $CDCl_3$) δ 7.40 – 7.17 (m, 10H), 5.16 – 5.07 (m, 2H), 4.47 (s, 2H), 3.44 – 3.31 (m, 2H), 2.67 (d, *J* = 6.9 Hz, 1H), 2.40 – 2.19 (m, 2H), 1.15 – 1.08 (m, 3H), 1.05 (d, *J* = 6.9 Hz, 9H), 0.92 (d, *J* = 7.1 Hz, 9H).

^{13}C NMR (126 MHz, $CDCl_3$) δ 207.2, 143.7, 138.2, 128.3, 128.1, 127.7, 127.6, 127.5, 127.1, 98.9, 86.3, 72.9, 72.7, 69.6, 29.4, 18.6, 18.4, 11.6.

HRMS (ESI) m/z [M – H_2O + H]$^+$ calcd for $C_{28}H_{39}SiO$: 419.2770, found: 419.2771.

$[\alpha]^{24}_{D}$ = –122.4 (*c* = 0.5, $CHCl_3$); 85% ee, from (*R,S*)-**L1**.

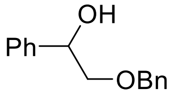

2-(benzyloxy)-1-phenylethan-1-ol (Figure 4, entry 59b). The title compound was prepared according to the **GP-3** from benzaldehyde, enyne **1** and DHP ester **S7**, purified by flash column chromatography on silica gel: 5→15% EtOAc in hexanes, colorless liquid.

(*R,S*)-**L1**: 25 mg, 28% yield, 76% ee; (*S,R*)-**L1**: 26 mg, 28% yield, 76% ee.

HPLC analysis: The ee was determined on a CHIRALCEL OD-3 column (10% *i*-PrOH in hexane, 1.0 mL/min); retention times for compound obtained using (*R,S*)-**L1**: 8.7 min (major), 9.6 min (minor).

^{1}H NMR (500 MHz, $CDCl_3$) δ 7.41 – 7.27 (m, 10H), 4.94 (d, *J* = 8.8 Hz, 1H), 4.65 – 4.57 (m, 2H), 3.65 (dd, *J* = 9.8, 3.2 Hz, 1H), 3.52 (t, *J* = 9.4 Hz, 1H), 2.85 (d, *J* = 1.7 Hz, 1H).

^{13}C NMR (126 MHz, $CDCl_3$) δ 140.2, 137.8, 128.5, 128.4, 127.9 (two carbons), 127.8, 126.2, 75.8, 73.4, 72.9.

The NMR data matched the reports (*5*).

HRMS (ESI) m/z [M – H_2O + H]$^+$ calcd for $C_{15}H_{15}O$: 211.1123, found: 211.1106.

$[\alpha]^{24}_{D}$ = +28.8 (*c* = 0.5, $CHCl_3$); 76% ee, from (*R,S*)-**L1**.

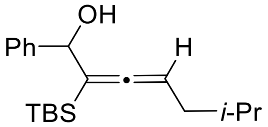

2-(*tert*-butyldimethylsilyl)-6-methyl-1-phenylhepta-2,3-dien-1-ol (Figure 4, entry 60). The title compound was prepared according to the **GP-3** from benzaldehyde, enyne **S9** and DHP ester **2**, purified by flash column chromatography on silica gel: 5→10% EtOAc in hexanes, colorless liquid.

(*R,S*)-**L1**: 92 mg, 73% yield, 10:1 dr, 92% ee; (*S,R*)-**L1**: 99 mg, 78% yield, 10:1 dr, 91% ee.

HPLC analysis: The ee was determined on a CHIRALCEL OD-3 column (3% *i*-PrOH in hexane, 1.0 mL/min); retention times for compound obtained using (*R,S*)-**L1**: 4.3 min (major), 6.8 min (minor).

^{1}H NMR (500 MHz, $CDCl_3$) δ 7.37 – 7.29 (m, 4H), 7.28 – 7.23 (m, 1H), 5.17 – 5.14 (m, 1H), 5.12 (td, *J* = 7.5, 2.5 Hz, 1H), 2.31 (d, *J* = 5.2 Hz, 1H), 2.00 – 1.86 (m, 2H), 1.62 (td, *J* = 13.3, 6.7 Hz, 1H), 0.90 (t, *J* = 6.9 Hz, 6H), 0.86 (s, 9H), 0.01 (s, 3H), -0.13 (s, 3H).

^{13}C NMR (126 MHz, $CDCl_3$) δ 205.6, 143.4, 128.2, 127.6, 127.1, 100.0, 89.2, 73.0, 38.1, 29.0, 26.7, 22.4, 22.2, 17.9, -5.2, -5.5.

HRMS (ESI) m/z $[M – H_2O + H]^+$ calcd for $C_{20}H_{31}Si$: 299.2195, found: 299.2208.

$[\alpha]^{24}_{D}$ = –158.4 (*c* = 0.5, $CHCl_3$); 92% ee, from (*R,S*)-**L1**.

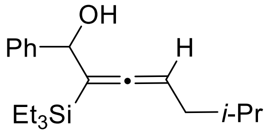

6-methyl-1-phenyl-2-(triethylsilyl)hepta-2,3-dien-1-ol (Figure 4, entry 61). The title compound was prepared according to the **GP-3** from benzaldehyde, enyne **S10** and DHP ester **2**, purified by flash column chromatography on silica gel: 5→10% EtOAc in hexanes, colorless liquid.

(*R,S*)-**L1**: 98 mg, 77% yield, 8:1 dr, 93% ee; (*S,R*)-**L1**: 93 mg, 783yield, 8:1 dr, 94% ee.

HPLC analysis: The ee was determined on a CHIRALPAK AD-3 column (1% *i*-PrOH in hexane, 1.0 mL/min); retention times for compound obtained using (*R,S*)-**L1**: 8.8 min (major), 8.1 min (minor).

^{1}H NMR (500 MHz, $CDCl_3$) δ 7.36 – 7.30 (m, 4H), 7.28 – 7.23 (m, 1H), 5.16 – 5.09 (m, 2H), 2.38 (d, *J* = 3.2 Hz, 1H), 1.97 (t, *J* = 7.1 Hz, 2H), 1.64 (dp, *J* = 13.3, 6.7 Hz, 1H), 0.93 (dd, *J* = 6.6, 4.0 Hz, 6H), 0.84 (t, *J* = 7.9 Hz, 9H), 0.54 – 0.37 (m, 6H).

^{13}C NMR (126 MHz, $CDCl_3$) δ 204.4, 143.3, 128.2, 127.7, 127.0, 99.2, 88.9, 72.8, 38.4, 29.0, 22.4, 22.3, 7.2, 3.3.

HRMS (ESI) m/z $[M – H_2O + H]^+$ calcd for $C_{20}H_{31}Si$: 299.2195, found: 299.2195.

$[\alpha]^{24}_{D}$ = –173.6 (*c* = 0.5, $CHCl_3$); 93% ee, from (*R,S*)-**L1**.

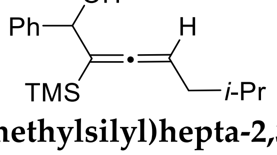

6-methyl-1-phenyl-2-(trimethylsilyl)hepta-2,3-dien-1-ol (Figure 4, entry 62). The title compound was prepared according to the **GP-3** from benzaldehyde, enyne **S11** and DHP ester **2**, purified by flash column chromatography on silica gel: 5→10% EtOAc in hexanes, colorless liquid.

(*R,S*)-**L1**: 65 mg, 60% yield, 6:1 dr, 95% ee; (*S,R*)-**L1**: 65 mg, 60% yield, 6:1 dr, 95% ee.

HPLC analysis: The ee was determined on a CHIRALCEL OD-3 column (1% *i*-PrOH in hexane, 1.0 mL/min); retention times for compound obtained using (*R,S*)-**L1**: 6.8 min (major), 9.0 min (minor).

^{1}H NMR (500 MHz, $CDCl_3$) δ 7.37 – 7.30 (m, 4H), 7.29 – 7.24 (m, 1H), 5.18 (d, *J* = 2.7 Hz, 1H), 5.15 (td, *J* = 7.4, 2.8 Hz, 1H), 1.96 (t, *J* = 7.0 Hz, 2H), 1.70 – 1.58 (m, 1H), 0.93 (dd, *J* = 6.6, 4.4 Hz, 6H), -0.04 (s, 9H).

^{13}C NMR (126 MHz, $CDCl_3$) δ 204.6, 144.2, 129.2, 128.7, 128.1, 103.4, 90.1, 74.0, 39.2, 29.9, 23.4, 23.2, -0.0.

HRMS (ESI) m/z $[M – H_2O + H]^+$ calcd for $C_{17}H_{25}Si$: 257.1725, found: 257.1729.

$[\alpha]^{24}_D$ = –145.6 (*c* = 0.5, $CHCl_3$); 95% ee, from (*R,S*)-**L1**.

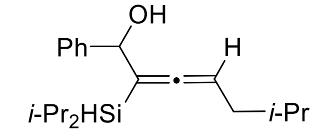

2-(diisopropylsilyl)-6-methyl-1-phenylhepta-2,3-dien-1-ol (Figure 4, entry 63). The title compound was prepared according to the **GP-3** from benzaldehyde, enyne **S15** and DHP ester **2**, purified by flash column chromatography on silica gel: 5→10% EtOAc in hexanes, colorless liquid.

(*R,S*)-**L1**: 82 mg, 65% yield, 16:1 dr, 96% ee; (*S,R*)-**L1**: 82 mg, 65% yield, 16:1 dr, 94% ee.

HPLC analysis: The ee was determined on a CHIRALCEL OD-3 column (3% *i*-PrOH in hexane, 1.0 mL/min); retention times for compound obtained using (*R,S*)-**L1**: 4.3 min (major), 4.7 min (minor).

^{1}H NMR (500 MHz, $CDCl_3$) δ 7.36 – 7.29 (m, 4H), 7.27 – 7.22 (m, 1H), 5.18 (d, *J* = 2.2 Hz, 1H), 5.10 (td, *J* = 7.6, 2.7 Hz, 1H), 3.54 (s, 1H), 2.38 (s, 1H), 1.99 – 1.89 (m, 2H), 1.69 – 1.55 (m, 1H), 0.99 (d, *J* = 2.1 Hz, 3H), 0.96 – 0.89 (m, 17H).

^{13}C NMR (126 MHz, $CDCl_3$) δ 205.0, 143.1, 128.1, 127.6, 126.9, 97.2, 88.9, 74.1, 38.2, 28.9, 22.3, 22.2, 18.6 (two carbons), 18.5, 18.4, 11.3, 10.9.

HRMS (ESI) m/z $[M – H_2O + H]^+$ calcd for $C_{20}H_{31}Si$: 299.2195, found: 299.2190.

$[\alpha]^{24}_D$ = –103.2 (*c* = 0.5, $CHCl_3$); 96% ee, from (*R,S*)-**L1**.

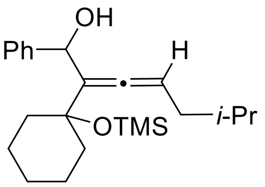

6-Methyl-1-phenyl-2-(1-((trimethylsilyl)oxy)cyclohexyl)hepta-2,3-dien-1-ol (Figure 4, entry 64). The title compound was prepared according to the **GP-3** from benzaldehyde, enyne **S16** and DHP ester **2**, purified by flash column chromatography on silica gel: 5→10% EtOAc in hexanes, colorless liquid.

(*R,S*)-**L1**: 82 mg, 45% yield, > 20:1 dr, 97% ee; (*S,R*)-**L1**: 82 mg, 45% yield, >

20:1 dr, 97% ee.

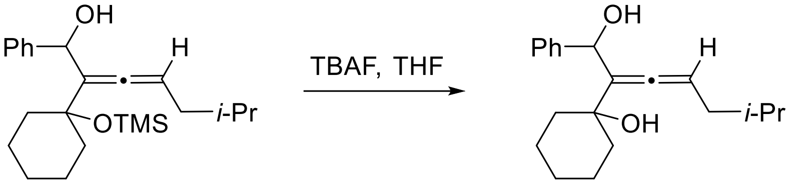

The following HPLC and NMR data were collected after deprotection by TBAF.

HPLC analysis: The ee was determined on a CHIRALCEL OD-3 column (10% *i*-PrOH in hexane, 1.0 mL/min); retention times for compound obtained using (*R,S*)-**L1**: 4.7 min (major), 5.0 min (minor).

^{1}H NMR (500 MHz, $CDCl_3$) δ 7.36 (d, *J* = 7.3 Hz, 2H), 7.30 (t, *J* = 7.6 Hz, 2H), 7.22 (t, *J* = 7.2 Hz, 1H), 5.52 (s, 1H), 5.14 (td, *J* = 7.5, 1.7 Hz, 1H), 3.25 (s, 1H), 2.16 (s, 1H), 1.89 – 1.80 (m, 1H), 1.77 – 1.68 (m, 4H), 1.67 – 1.44 (m, 6H), 1.43 – 1.27 (m, 2H), 0.77 (d, *J* = 6.6 Hz, 3H), 0.73 (d, *J* = 6.7 Hz, 3H).

^{13}C NMR (126 MHz, $CDCl_3$) δ 202.0, 143.2, 128.0, 127.2, 126.5, 113.8, 95.0, 73.6, 72.4, 38.2, 38.1, 37.6, 28.5, 25.6, 22.4, 22.2, 22.1.

HRMS (ESI) m/z [M – H_2O + H]$^+$ calcd for $C_{20}H_{27}O$: 283.2062, found: 283.2078.

$[\alpha]^{24}_{D}$ = +7.2 (*c* = 0.5, $CHCl_3$); 97% ee, from (*R,S*)-**L1**.

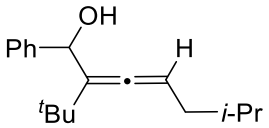

2-(*tert*-butyl)-6-methyl-1-phenylhepta-2,3-dien-1-ol (Figure 4, entry 65). The title compound was prepared according to the **GP-3** from benzaldehyde, enyne **S12** and DHP ester **2**, purified by flash column chromatography on silica gel: 5→10% EtOAc in hexanes, colorless liquid.

(*R,S*)-**L1**: 82 mg, 80% yield, 20:1 dr, 80% ee; (*S,R*)-**L1**: 81 mg, 79% yield, 20:1 dr, 78% ee.

HPLC analysis: The ee was determined on a CHIRALPAK IC-3 column (3% *i*-PrOH in hexane, 1.0 mL/min); retention times for compound obtained using (*R,S*)-**L1**: 5.9 min (major), 5.4 min (minor).

^{1}H NMR (500 MHz, $CDCl_3$) δ 7.39 – 7.34 (m, 2H), 7.30 (t, *J* = 7.5 Hz, 2H), 7.26 – 7.21 (m, 1H), 5.36 (td, *J* = 7.4, 1.2 Hz, 1H), 5.20 (s, 1H), 2.12 (brs, 1H), 1.83 (t, *J* = 7.1 Hz, 2H), 1.53 (dp, *J* = 13.3, 6.7 Hz, 1H), 1.07 (s, 9H), 0.86 (dd, *J* = 9.6, 6.7 Hz, 6H).

^{13}C NMR (126 MHz, $CDCl_3$) δ 200.3, 144.0, 128.1, 127.3, 126.8, 118.0, 96.0, 71.0, 38.6, 33.4, 30.0, 28.7, 22.4, 22.3.

HRMS (ESI) m/z [M – H_2O + H]$^+$ calcd for $C_{18}H_{25}$: 241.1956, found: 241.1954.

$[\alpha]^{24}_{D}$ = –136.8 (*c* = 0.5, $CHCl_3$); 80% ee, from (*R,S*)-**L1**.

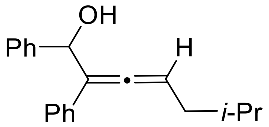

6-methyl-1,2-diphenylhepta-2,3-dien-1-ol (Figure 4, entry 66). The title compound was prepared according to the **GP-3** from benzaldehyde, enyne **S13** and DHP ester **2**, purified by flash column chromatography on silica gel: 5→10% EtOAc in hexanes, colorless liquid.

(*R,S*)-**L1**: 70 mg, 63% yield, 11:1 dr, 98% ee; (*S,R*)-**L1**: 70 mg, 63% yield, 11:1 dr, 98% ee.

HPLC analysis: The ee was determined on a CHIRALPAK AS-3 column (5% *i*-PrOH in hexane, 1.0 mL/min); retention times for compound obtained using (*R,S*)-**L1**: 5.1 min (major), 4.8 min (minor).

^{1}H NMR (600 MHz, $CDCl_3$) δ 7.44 (d, *J* = 7.2 Hz, 2H), 7.38 – 7.34 (m, 2H), 7.32 (t, *J* = 7.6 Hz, 2H), 7.27 – 7.22 (m, 3H), 7.16 (t, *J* = 7.4 Hz, 1H), 5.71 – 5.64 (m, 2H), 2.32 (d, *J* = 3.7 Hz, 1H), 2.05 – 1.94 (m, 2H), 1.65 (dp, *J* = 13.3, 6.7 Hz, 1H), 0.91 (dd, *J* = 11.5, 6.7 Hz, 6H).

^{13}C NMR (151 MHz, $CDCl_3$) δ 203.0, 142.1, 134.8, 128.2, 128.1, 127.5, 126.7, 126.6 (two carbons), 109.8, 96.7, 72.3, 38.1, 28.3, 22.1 (two carbons).

HRMS (ESI) m/z $[M – H_2O + H]^+$ calcd for $C_{20}H_{21}$: 261.1643, found: 261.1622.

$[\alpha]^{24}_{D}$ = –46.8 (*c* = 0.5, $CHCl_3$); 98% ee, from (*R,S*)-**L1**.

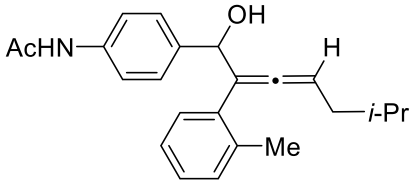

***N*-(4-(1-hydroxy-6-methyl-2-(*o*-tolyl)hepta-2,3-dien-1-yl)phenyl)acetamide (Figure 4, entry 67).** The title compound was prepared according to the **GP-3** from *N*-(4-formylphenyl)acetamide, enyne **S14** and DHP ester **2**, purified by flash column chromatography on silica gel: 20→50% EtOAc in hexanes, colorless liquid.

(*R,S*)-**L1**: 95 mg, 68% yield, 7:1 dr, 96% ee; (*S,R*)-**L1**: 97 mg, 69% yield, 7:1 dr, 95% ee.

SFC analysis: The ee was determined on a CHIRALCEL IG-3 column (20% *i*-PrOH in CO_2, 2.0 mL/min); retention times for compound obtained using (*R,S*)-**L1**: 3.9 min (major), 3.0 min (minor).

^{1}H NMR (500 MHz, $CDCl_3$) δ 7.40 (d, *J* = 8.4 Hz, 2H), 7.20 (d, *J* = 8.4 Hz, 2H), 7.15 – 7.03 (m, 5H), 5.48 (td, *J* = 7.5, 2.7 Hz, 1H), 5.35 (s, 1H), 2.40 (brs, 1H), 2.18 – 2.12 (m, 6H), 2.04 – 1.97 (m, 2H), 1.67 (dp, *J* = 13.4, 6.7 Hz, 1H), 0.92 (dd, *J* = 6.6, 5.1 Hz, 6H).

^{13}C NMR (126 MHz, $CDCl_3$) δ 200.1, 168.1, 137.9, 137.3, 136.5, 134.8, 132.0, 130.3, 128.9, 127.3, 125.6, 119.2, 108.7, 95.1, 74.2, 38.1, 28.6, 24.7, 22.3 (two carbons), 20.1.

HRMS (ESI) m/z $[M - H_2O + H]^+$ calcd for $C_{23}H_{26}NO$: 332.2014, found: 332.2052.

$[\alpha]^{24}_{D}$ = –22.0 (*c* = 0.5, $CHCl_3$); 96% ee, from (*R,S*)-**L1**.

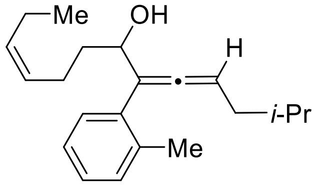

(*Z*)-12-methyl-8-(*o*-tolyl)trideca-3,8,9-trien-7-ol (Figure 4, entry 68). The title compound was prepared according to the **GP-3** from (*Z*)-hept-4-enal, enyne **S14** and DHP ester **2**, purified by flash column chromatography on silica gel: 5→10% EtOAc in hexanes, colorless liquid.

(*R,S*)-**L1**: 78 mg, 65% yield, 4:1 dr, 93% ee; (*S,R*)-**L1**: 78 mg, 65% yield, 4:1 dr, 95% ee.

HPLC analysis: The ee was determined on a CHIRALPAK IC-3 column (5% *i*-PrOH in hexane, 1.0 mL/min); retention times for compound obtained using (*R,S*)-**L1**: 4.3 min (major), 4.0 min (minor).

^{1}H NMR (500 MHz, $CDCl_3$) δ 7.25 – 7.15 (m, 4H), 5.46 – 5.35 (m, 2H), 5.30 (ddd, *J* = 10.7, 9.8, 7.2 Hz, 1H), 4.41 – 4.34 (m, 1H), 2.35 (s, 3H), 2.27 – 2.10 (m, 2H), 2.09 – 1.97 (m, 4H), 1.78 (d, *J* = 5.5 Hz, 1H), 1.75 – 1.64 (m, 2H), 1.61 – 1.49 (m, 1H), 0.94 (dd, *J* = 10.2, 4.2 Hz, 9H).

^{13}C NMR (126 MHz, $CDCl_3$) δ 200.6, 136.4, 135.6, 132.3, 130.5, 128.6, 128.4, 127.2, 125.8, 108.5, 94.2, 71.7, 38.3, 36.0, 28.7, 23.4, 22.4, 22.3, 20.5, 20.4, 14.4.

HRMS (ESI) m/z $[M - H_2O + H]^+$ calcd for $C_{21}H_{29}$: 281.2269, found: 281.2267.

$[\alpha]^{24}_{D}$ = –60.4 (*c* = 0.5, $CHCl_3$); 93% ee, from (*R,S*)-**L1**.

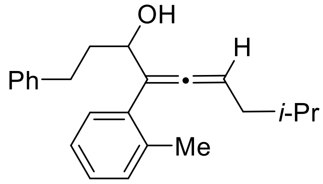

8-methyl-1-phenyl-4-(*o*-tolyl)nona-4,5-dien-3-ol (Figure 4, entry 69). The title compound was prepared according to the **GP-3** from 3-phenylpropanal, enyne **S14** and DHP ester **2**, purified by flash column chromatography on silica gel: 5→10% EtOAc in hexanes, colorless liquid.

(*R,S*)-**L1**: 92 mg, 72% yield, 4:1 dr, 94% ee; (*S,R*)-**L1**: 92 mg, 72% yield, 4:1 dr, 94% ee.

HPLC analysis: The ee was determined on a CHIRALCEL OD-3 column (5% *i*-PrOH in hexane, 1.0 mL/min); retention times for compound obtained using (*R,S*)-**L1**: 6.6 min (major), 5.5 min (minor).

^{1}H NMR (500 MHz, $CDCl_3$) δ 7.28 – 7.21 (m, 2H), 7.18 (ddt, *J* = 14.9, 9.4, 4.9

Hz, 7H), 5.44 (td, J = 7.5, 2.5 Hz, 1H), 4.44 – 4.36 (m, 1H), 2.90 – 2.78 (m, 1H), 2.69 (ddd, J = 13.7, 10.4, 6.2 Hz, 1H), 2.33 (s, 3H), 2.03 (td, J = 7.2, 2.9 Hz, 2H), 1.99 – 1.90 (m, 1H), 1.84 (d, J = 5.5 Hz, 1H), 1.82 – 1.74 (m, 1H), 1.70 (td, J = 13.3, 6.7 Hz, 1H), 0.96 – 0.89 (m, 6H).

^{13}C NMR (126 MHz, $CDCl_3$) δ 200.6, 142.1, 136.4, 135.5, 130.6, 128.7, 128.5, 128.4, 127.3, 125.9, 125.8, 108.4, 94.4, 71.4, 38.3, 37.7, 32.0, 28.7, 22.4 (two carbons), 20.5.

HRMS (ESI) m/z [M – H_2O + H]$^+$ calcd for $C_{23}H_{27}$: 303.2113, found: 303.2112.

$[\alpha]^{24}_D$ = –34.8 (c = 0.5, $CHCl_3$); 94% ee, from (R,S)-**L1**.

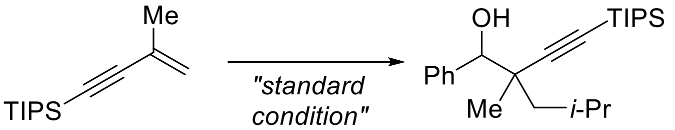

70

42%, 1:1 dr

2,4-dimethyl-1-phenyl-2-((triisopropylsilyl)ethynyl)pentan-1-ol (Figure 4, entry 70). The title compound was prepared according to the above equation, purified by flash column chromatography on silica gel: 5→10% EtOAc in hexanes, colorless liquid.

(R,S)-**L1**: 62 mg, 42% yield, 1:1 dr; (S,R)-**L1**: 63 mg, 42% yield, 1:1 dr.

^{1}H NMR (500 MHz, $CDCl_3$, mixture of two isomers) δ 7.44 – 7.37 (m, 2H), 7.34 – 7.25 (m, 3H), 4.46 (dd, J = 8.9, 4.4 Hz, 1H), 2.74 (d, J = 4.3 Hz, 0.5H), 2.56 (d, J = 4.7 Hz, 0.5H), 1.98 – 1.82 (m, 1H), 1.63 (dd, J = 13.6, 5.9 Hz, 0.5H), 1.45 (dd, J = 13.6, 6.2 Hz, 0.5H), 1.27 (s, 1.5H), 1.18 (dd, J = 13.6, 5.7 Hz, 0.5H), 1.07 (dt, J = 16.2, 4.6 Hz, 25H), 0.98 (d, J = 6.7 Hz, 1.5H), 0.94 (d, J = 6.7 Hz, 1.5H), 0.90 (d, J = 6.7 Hz, 1.5H).

^{13}C NMR (126 MHz, $CDCl_3$, mixture of two isomers) δ 139.9, 139.6, 128.0 (two carbons), 127.7, 127.6 (two carbons), 127.5, 112.8, 112.7, 85.2, 84.7, 80.4, 80.0, 46.7, 44.5, 43.2, 42.8, 25.4, 25.3, 24.9 (two carbons), 24.6, 24.5, 24.0, 22.1, 18.6, 11.3.

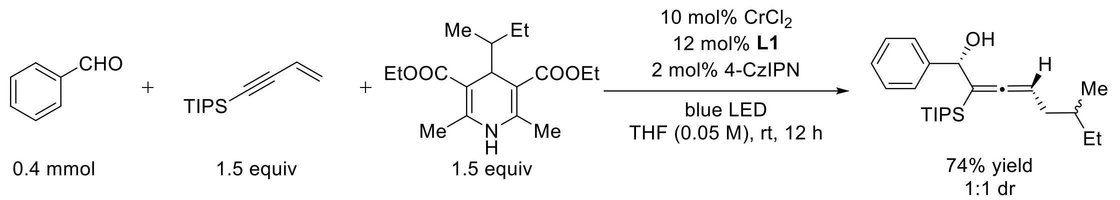

When the secondary alkyl-DHP bearing unsymmetric alkyl group was evaluated, the diastereoselectivity was low (as above showed).

^{1}H NMR (500 MHz, $CDCl_3$, mixture of two isomers) δ 7.42 – 7.34 (m, 2H), 7.31 (t, J = 7.5 Hz, 2H), 7.28 – 7.21 (m, 1H), 5.13 (d, J = 3.1 Hz, 1H), 5.07 (t, J = 7.5 Hz, 1H), 2.32 (s, 1H), 2.14 – 1.96 (m, 1H), 1.95 – 1.78 (m, 1H), 1.20 – 1.03 (m, 16H), 0.93 (dd, J = 7.2, 1.6 Hz, 10H), 0.90 – 0.82 (m, 6H).

^{13}C NMR (126 MHz, $CDCl_3$, mixture of two isomers) δ 206.8, 206.7, 143.6

(two carbons), 128.2 (two carbons), 127.6 (two carbons), 127.2 (two carbons), 98.1, 98.0, 88.8 (two carbons), 72.7 (two carbons), 35.9, 35.8, 35.5, 35.4, 29.2, 29.0, 19.2, 19.0, 18.7, 18.5, 11.7, 11.6, 11.5.

1.4 Other Radical Precursors and Synthetic Transformations

General procedure 4 (GP-4): Asymmetric 1,4-functionalization of 1,3-enynes with RBF_3K.

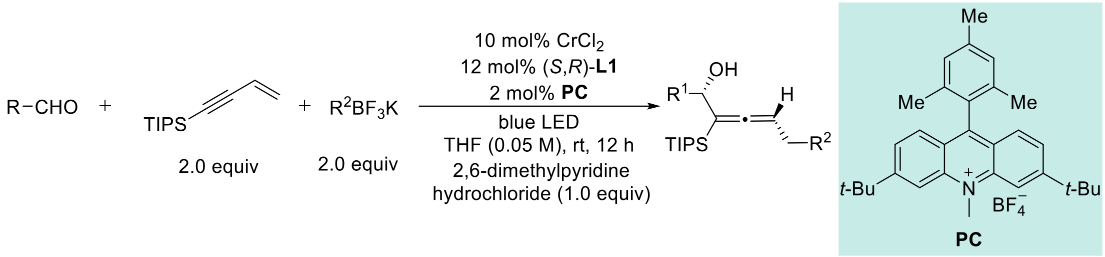

Preparation of the catalyst solution: In a nitrogen-filled glovebox, and oven-dried 20 mL vial with a magnetic stir bar, were charged the $CrCl_2$ (5.0 mg, 0.04 mmol, 10 mol%) and (*S*,*R*)-**L1** (23.2 mg, 0.048 mmol, 12 mol%). Then 8.0 mL THF was added and the vial was closed with a PTFE septum cap, and then stirred at room temperature for 2 h.

Catalytic asymmetric radical 1,4-functionalization of 1,3-enynes: In a nitrogen-filled glovebox, to the prepared catalyst solution were added the 1,3-enynes (0.8 mmol, 2.0 equiv), the aldehydes (0.4 mmol, 1.0 equiv), the potassium trifluoroborate (0.8 mmol, 2.0 equiv), 2,6-dimethylpyridine hydrochloride (0.4 mmol, 1.0 equiv), and photocatalyst 3,6-di-tert-butyl-9-mesityl-10-methylacridin-10-ium tetrafluoroborate (4.1 mg, 0.008 mmol, 2 mol%) sequentially. Then the vial was closed with a PTFE septum cap, and taken out of the glovebox. Then, the reaction was irradiated with two 20 W 160-440 nm LED for 12 hours (tube 5 cm away from lights, fans for cooling, 30-35 °C). After that, the reaction mixture was concentrated and run through a short silica gel pad with hexanes/EtOAc (3:1) as the eluent. Then the solvent was removed under the reduced pressure. The diastereoselectivity was determined via ^1H NMR analysis of the crude reaction mixture. and the residue was purified by flash chromatography to provide the desired product and the ee was determined via HPLC analysis.

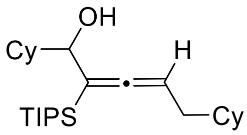

1,5-dicyclohexyl-2-(triisopropylsilyl)penta-2,3-dien-1-ol (Figure 5, entry 71). The title compound was prepared according to the **GP-4** from cyclohexanecarbaldehyde, enyne **1** and potassium cyclohexyltrifluoroborate, purified by flash column chromatography on silica gel: 5→10% EtOAc in hexanes, colorless liquid.

(*R,S*)-**L1**: 111 mg, 69% yield, 18:1 dr, 97% ee; (*S,R*)-**L1**: 111 mg, 69% yield, 18:1 dr, 96% ee.

HPLC analysis: The ee was determined on a CHIRALCEL OD-3 column (1% *i*-PrOH in hexane, 1.0 mL/min); retention times for compound obtained using (*R,S*)-**L1**: 3.4 min (major), 3.6 min (minor).

^{1}H NMR (500 MHz, $CDCl_3$) δ 4.96 (ddd, *J* = 8.4, 6.9, 1.7 Hz, 1H), 3.78 (s, 1H), 2.00 – 1.85 (m, 3H), 1.82 – 1.61 (m, 9H), 1.30 – 1.12 (m, 11H), 1.08 (dd, *J* = 9.0, 7.4 Hz, 18H), 1.04 – 0.86 (m, 5H).

^{13}C NMR (126 MHz, $CDCl_3$) δ 206.2, 95.8, 87.5, 74.2, 43.3, 38.7, 36.5, 33.2, 33.1, 31.2, 26.5 (four carbons), 26.3 (two carbons), 26.2, 18.7 (two carbons), 11.7.

HRMS (ESI) m/z $[M – H_2O + H]^+$ calcd for $C_{26}H_{47}Si$: 387.3447, found: 387.3443.

$[\alpha]^{24}_{D}$ = –42.0 (*c* = 0.5, $CHCl_3$); 97% ee, from (*R,S*)-**L1**.

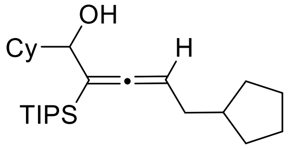

1-cyclohexyl-5-cyclopentyl-2-(triisopropylsilyl)penta-2,3-dien-1-ol (Figure 5, entry 72). The title compound was prepared according to the **GP-4** from cyclohexanecarbaldehyde, enyne **1** and potassium cyclopentyltrifluoroborate, purified by flash column chromatography on silica gel: 5→10% EtOAc in hexanes, colorless liquid.

(*R,S*)-**L1**: 106 mg, 68% yield, 16:1 dr, 97% ee; (*S,R*)-**L1**: 106 mg, 68% yield, 16:1 dr, 98% ee.

HPLC analysis: The ee was determined on a CHIRALCEL OD-3 column (1% *i*-PrOH in hexane, 1.0 mL/min); retention times for compound obtained using (*R,S*)-**L1**: 3.4 min (major), 3.6 min (minor).

^{1}H NMR (500 MHz, $CDCl_3$) δ 5.02 – 4.94 (m, 1H), 3.79 (s, 1H), 2.11 – 1.97 (m, 2H), 1.93 – 1.81 (m, 2H), 1.82 – 1.73 (m, 4H), 1.71 – 1.57 (m, 4H), 1.56 – 1.40 (m, 4H), 1.23 – 1.12 (m, 9H), 1.11 – 1.05 (m, 18H).

^{13}C NMR (126 MHz, $CDCl_3$) δ 206.0, 96.0, 88.4, 74.2, 43.3, 40.7, 34.9, 32.4, 32.3, 31.2, 26.5 (three carbons), 26.2, 25.2, 25.1, 18.7 (two carbons), 11.7.

HRMS (ESI) m/z $[M – H_2O + H]^+$ calcd for $C_{25}H_{45}Si$: 373.3290, found: 373.3282.

$[\alpha]^{24}_{D}$ = –36.4 (*c* = 0.5, $CHCl_3$); 97% ee, from (*R,S*)-**L1**.

General procedure 5 (GP-5): Radical Difunctionalization of 1,3-Enynes with NHPI ester.

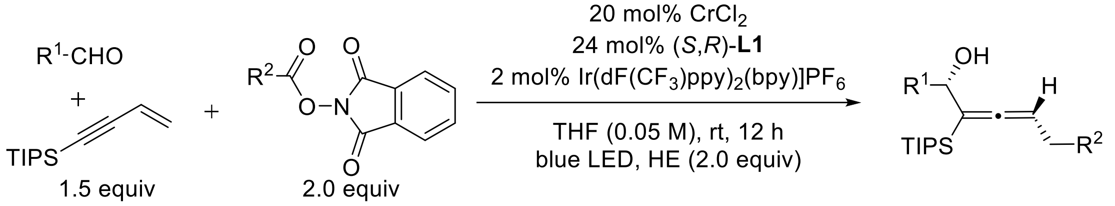

Preparation of the catalyst solution: In a nitrogen-filled glovebox, and oven-dried 20 mL vial with a magnetic stir bar, were charged the $CrCl_2$ (9.5 mg, 0.08 mmol, 20 mol%) and (*S,R*)-**L1** (46.4 mg, 0.096 mmol, 24 mol%). Then 8.0 mL THF was added and the vial was closed with a PTFE septum cap, and then stirred at room temperature for 2 h.

Catalytic asymmetric radical difunctionalization of 1,3-enynes: In a nitrogen-filled glovebox, to the prepared catalyst solution were added the 1,3-enynes (0.6 mmol, 1.5 equiv), the aldehydes (0.4 mmol, 1.0 equiv), the NHPI ester (0.8 mmol, 2.0 equiv), diethyl 1,4-dihydro-2,6-dimethyl-3,5-pyridinedicarboxylate (0.8 mmol, 2.0 equiv), and photocatalyst $Ir(dF(CF_3)ppy)_2(bpy)]PF_6$ (8.2 mg, 0.008 mmol, 2 mol%) sequentially. Then the vial was closed with a PTFE septum cap, and taken out of the glovebox. Then, the reaction was irradiated with two 20 W 160-440 nm LED for 12 hours (tube 5 cm away from lights, fans for cooling, 30-35 °C). After that, the reaction mixture was concentrated and run through a short silica gel pad with hexanes/EtOAc (3:1) as the eluent. Then the solvent was removed under the reduced pressure. The diastereoselectivity was determined via ^{1}H NMR analysis of the crude reaction mixture. and the residue was purified by flash chromatography to provide the desired product and the ee was determined via HPLC analysis.

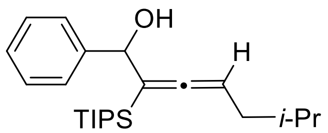

6-methyl-1-phenyl-2-(triisopropylsilyl)hepta-2,3-dien-1-ol (Figure 5, entry 1). The title compound was prepared according to the **GP-5** from benzaldehyde, enyne **1** and NHPI ester **S17**, purified by flash column chromatography on silica gel: 5→10% EtOAc in hexanes, colorless liquid.

(*R,S*)-**L1**: 91 mg, 64% yield, >20:1 dr, 90% ee; (*S,R*)-**L1**: 91 mg, 64% yield, >20:1 dr, 90% ee.

HPLC analysis: The ee was determined on a CHIRALCEL OD-3 column (5% *i*-PrOH in hexane, 1.0 mL/min); retention times for compound obtained using (*R,S*)-**L1**: 3.7 min (major), 4.6 min (minor).

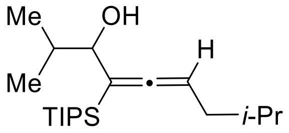

2,8-dimethyl-4-(triisopropylsilyl)nona-4,5-dien-3-ol (Figure 5, entry 31). The title compound was prepared according to the **GP-5** from isobutyraldehyde, enyne **1** and NHPI ester **S17**, purified by flash column chromatography on silica gel: 5→10% EtOAc in hexanes, colorless liquid.

(*R,S*)-**L1**: 71 mg, 55% yield, 20:1 dr, 96% ee; (*S,R*)-**L1**: 71 mg, 55% yield, 20:1 dr, 96% ee.

HPLC analysis: The ee was determined on a CHIRALPAK OD-3 column (1% *i*-PrOH in hexane, 1.0 mL/min); retention times for compound obtained using (*R*,*S*)-**L1**: 3.5 min (major), 3.3 min (minor).

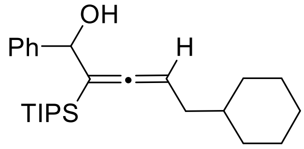

5-cyclohexyl-1-phenyl-2-(triisopropylsilyl)penta-2,3-dien-1-ol (Figure 5, entry 53). The title compound was prepared according to the **GP-5** from benzaldehyde, enyne **1** and NHPI ester **S18**, purified by flash column chromatography on silica gel: 5→10% EtOAc in hexanes, colorless liquid.

(*R*,*S*)-**L1**: 116 mg, 73% yield, >20:1 dr, 90% ee; (*S*,*R*)-**L1**: 115 mg, 72% yield, 20:1 dr, 89% ee.

HPLC analysis: The ee was determined on a CHIRALCEL OD-3 column (5% *i*-PrOH in hexane, 1.0 mL/min); retention times for compound obtained using (*R*,*S*)-**L1**: 3.8 min (major), 5.5 min (minor).

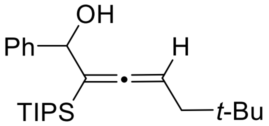

6,6-dimethyl-1-phenyl-2-(triisopropylsilyl)hepta-2,3-dien-1-ol (Figure 5, entry 58). The title compound was prepared according to the **GP-5** from benzaldehyde, enyne **1** and NHPI ester **S19**, purified by flash column chromatography on silica gel: 5→10% EtOAc in hexanes, colorless liquid.

(*R*,*S*)-**L1**: 111 mg, 75% yield, >20:1 dr, 89% ee; (*S*,*R*)-**L1**: 110 mg, 75% yield, >20:1 dr, 89% ee.

HPLC analysis: The ee was determined on a CHIRALCEL OD-3 column (5% *i*-PrOH in hexane, 1.0 mL/min); retention times for compound obtained using (*R*,*S*)-**L1**: 3.5 min (major), 3.9 min (minor).

Desilylation and cyclization reaction:

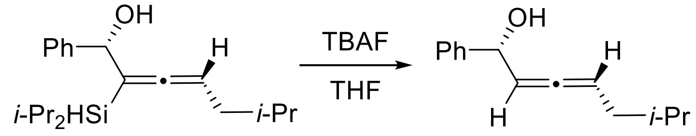

73

86%, >20:1 dr, 96% ee

(1*R*,3*R*)-6-methyl-1-phenylhepta-2,3-dien-1-ol (Figure 5c, 73): A solution of the compound **63** (680 mg, 2.2 mmol, 1.0 equiv) in 15 mL THF was cooled to – 78 °C, TBAF (3.3 mL, 3.3 mmol, 1.5 equiv, 1.0 M in THF) was added dropwise

under argon atmosphere. The mixture was stirred at –78 °C for 2 h. After completion of the reaction, 1.0 mL H_2O was added cautiously at –78 °C. The resulting mixture was extracted with EtOAc (2×20 mL), and the organic phase was combined and dried with anhydrous $MgSO_4$, and concentrated. The resulting mixture was purified by flash chromatography (hexanes/ethyl acetate), which furnished the compound **73**, colorless liquid, 382 mg, 86% yield, > 20:1 dr, 96% ee.

HPLC analysis: The ee was determined on a CHIRALPAK OD-3 column (5% *i*-PrOH in hexane, 1.0 mL/min); retention times for compound **73**: 5.6 min (major), 6.8 min (minor).

^{1}H NMR (500 MHz, $CDCl_3$) δ 7.42 – 7.33 (m, 4H), 7.31 – 7.26 (m, 1H), 5.43 – 5.37 (m, 1H), 5.36 – 5.29 (m, 1H), 5.23 (dd, *J* = 6.0, 2.1 Hz, 1H), 2.14 (brs, 1H), 1.99 – 1.92 (m, 2H), 1.73 – 1.63 (m, 1H), 0.93 (dd, *J* = 6.6, 2.3 Hz, 6H).

^{13}C NMR (126 MHz, $CDCl_3$) δ 202.7, 143.1, 128.5, 127.7, 126.2, 95.5, 93.7, 72.3, 38.3, 28.5, 22.2 (two carbons).

HRMS (ESI) m/z $[M – H_2O + H]^+$ calcd for $C_{14}H_{17}$: 185.1330, found: 185.1326.

$[\alpha]^{24}_D$ = –55.6 (*c* = 0.5, $CHCl_3$).

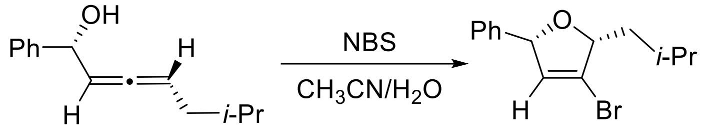

73

74

60%, >20:1 dr, 96% ee

(2*R*,5*R*)-3-bromo-2-isobutyl-5-phenyl-2,5-dihydrofuran (Figure 5c, 74): NBS (85 mg, 0.48 mmol, 1.2 equiv) in 1.0 mL MeCN was added to the solution of the compound **73** (83 mg, 0.4 mmol, 1.0 equiv) in 2.0 mL MeCN and 0.2 mL H_2O under argon atmosphere. The mixture was stirred at rt for 4 h. After completion of the reaction, the mixture was concentrated, purified by flash chromatography (hexanes/ethyl acetate), which furnished compound **74**, colorless liquid, 67 mg, 60% yield, >20:1 dr, 96% ee.

HPLC analysis: The ee was determined on a CHIRALPAK OJ-3 column (1% *i*-PrOH in hexane, 1.0 mL/min); retention times for compound **74**: 5.3 min (major), 5.5 min (minor).

^{1}H NMR (500 MHz, $CDCl_3$) δ 7.36 (ddd, *J* = 7.1, 4.3, 1.6 Hz, 2H), 7.33 – 7.29 (m, 3H), 5.99 (t, *J* = 1.9 Hz, 1H), 5.70 (dd, *J* = 4.2, 1.6 Hz, 1H), 4.88 – 4.80 (m, 1H), 2.02 – 1.89 (m, 1H), 1.74 (ddd, *J* = 13.9, 9.3, 2.7 Hz, 1H), 1.58 – 1.49 (m, 1H), 0.98 (t, *J* = 6.6 Hz, 6H).

^{13}C NMR (126 MHz, $CDCl_3$) δ 140.8, 129.4, 128.5, 128.1, 126.6, 121.6, 86.9, 85.6, 44.1, 24.8, 23.8, 21.6.

HRMS (ESI) m/z $[M – Br]^+$ calcd for $C_{14}H_{17}O$: 201.1279, found: 201.1277.

$[\alpha]^{24}_D$ = +45.6 (*c* = 0.5, $CHCl_3$).

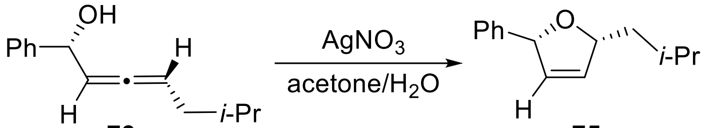

73 **75**

84%, >20:1 dr, 96% ee

(2*R*,5*R*)-2-isobutyl-5-phenyl-2,5-dihydrofuran (Figure 5c, 75): The compound **73** (83 mg, 0.4 mmol, 1.0 equiv) was added to the solution of $AgNO_3$ (13.6 mg, 0.08 mmol, 20 mol%) in 3.0 mL acetone and 2.0 mL H_2O under argon atmosphere. The mixture was stirred at rt for 12 h. After completion of the reaction, the mixture was concentrated, purified by flash chromatography (hexanes/ethyl acetate), which furnished compound **75**, colorless liquid, 68 mg, 84% yield, >20:1 dr, 96% ee.

HPLC analysis: The ee was determined on a CHIRALPAK OJ-3 column (3% *i*-PrOH in hexane, 1.0 mL/min); retention times for compound **75**: 5.4 min (major), 6.2 min (minor).

^1H NMR (500 MHz, $CDCl_3$) δ 7.37 – 7.30 (m, 4H), 7.29 – 7.25 (m, 1H), 5.92 (ddd, *J* = 6.0, 2.3, 1.4 Hz, 1H), 5.86 – 5.79 (m, 1H), 5.75 (dd, *J* = 4.0, 2.0 Hz, 1H), 5.01 – 4.94 (m, 1H), 1.94 – 1.80 (m, 1H), 1.63 (ddd, *J* = 14.2, 8.1, 6.3 Hz, 1H), 1.49 (ddd, *J* = 13.3, 7.7, 5.4 Hz, 1H), 0.97 (d, *J* = 6.7 Hz, 6H).

^{13}C NMR (126 MHz, $CDCl_3$) δ 142.3, 130.8, 129.9, 128.4, 127.6, 126.5, 87.6, 84.9, 46.2, 25.2, 23.3, 22.5.

HRMS (ESI) m/z $[M + H]^+$ calcd for $C_{14}H_{19}O$: 203.1436, found: 203.1432.

$[\alpha]^{24}_D$ = +142.0 (*c* = 0.5, $CHCl_3$).

1.5 Effect of Reaction Parameters

General procedure 6 (GP-6): Asymmetric radical 1,4-functionalization of 1,3-enynes with DHP esters.

Preparation of the catalyst solution: In a nitrogen-filled glovebox, and oven-dried 4 mL vial with a magnetic stir bar, were charged the $CrCl_2$ (1.3 mg, 0.01 mmol, 10 mol%) and (*S*,*R*)-**L1** (5.8 mg, 0.012 mmol, 12 mol%). Then 2.0 mL THF was added and the vial was closed with a PTFE septum cap, and then stirred at room temperature for 2 h.

Catalytic asymmetric radical 1,4-functionalization of 1,3-enynes: In a nitrogen-filled glovebox, to the prepared catalyst solution were added the 1,3-enyne (0.15 mmol, 1.5 equiv), the aldehyde (0.1 mmol, 1.0 equiv), the DHP ester (0.15 mmol, 1.5 equiv), and 4-CzIPN (1.6 mg, 0.002 mmol, 2 mol%) sequentially. Then the vial was closed with a PTFE septum cap, and taken out of the glovebox. Then, the reaction was irradiated with two 20 W 160-440 nm LED for 12 hours (tube 5 cm away from lights, fans for cooling, 30-35 °C). After that, the reaction mixture was concentrated and run through a short silica gel pad with hexanes/EtOAc (3:1) as the eluent. Then the solvent was removed under the reduced pressure. The yield and diastereoselectivity were determined via ^{1}H NMR analysis of the crude reaction mixture. The ee was determined via HPLC analysis after further purification by prep-TLC.

Supplementary Table 1 Effect of reaction parameters on the reaction of asymmetric 1,4-functionalization of 1,3-enynes[a]

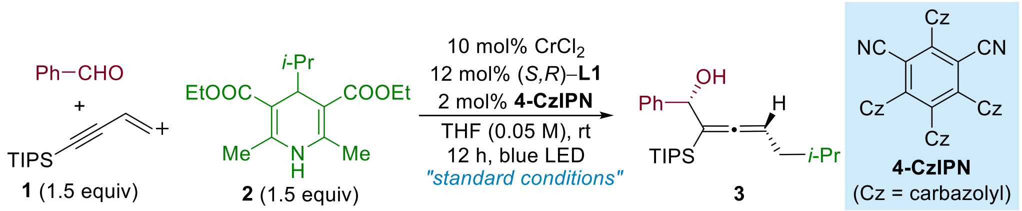

entry	variation from *"standard conditions"*	yield [%]	dr[b]	ee[c] [%]
1	None	>95	20:1	94
2	without $CrCl_2$	<2	–	–
3	without 4-CzIPN	<2	–	–
4	without blue LED	<2	–	–
5	**L2**, instead of (*S*,*R*)–**L1**	>95	20:1	92
6	**L3**, instead of (*S*,*R*)–**L1**	84	3.1:1	47
7	**L4**, instead of (*S*,*R*)–**L1**	68	1.6:1	19
8	**L5**, instead of (*S*,*R*)–**L1**	52	5.5:1	34
9	**L6**, instead of (*S*,*R*)–**L1**	71	1.8:1	46
10	**L7**, instead of (*S*,*R*)–**L1**	79	2.8:1	93
11	DME, instead of THF	72	10:1	94
12	MeCN, instead of THF	85	20:1	95
13	EtOAc, instead of THF	>95	20:1	94
14	$Ir(dF(CF_3)ppy)_2(dtbpy)]PF_6$ instead of 4-CzIPN	>95	11:1	88
15	5 mol% $CrCl_2$, 6 mol% (*S*,*R*)–**L1**	74	20:1	94
16	0.1M, instead of 0.05M, in THF	>95	12:1	93
17	1.2, instead of 1.5, equiv **1** and **2**	80	20:1	94
18	1.2, instead of 1.5, equiv **1**	90	18:1	93
19	1.0 equiv H_2O was added	<2	–	–
20	1 mL air (added via syringe)	55	14:1	87

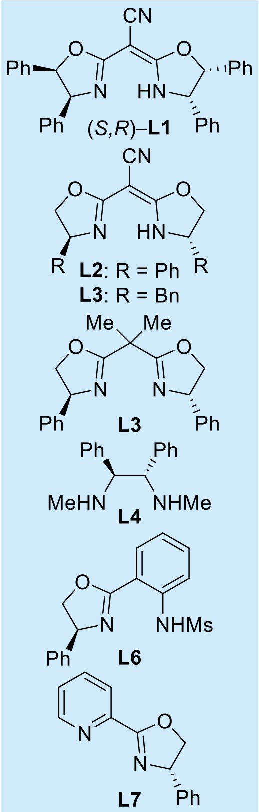

[a] Yields were determined via ^{1}H NMR analysis with 1,3,5-trimethoxybenzene as the internal standard. [b] Drs were determined via ^{1}H NMR analysis of the crude product. [c] Ee was determined by HPLC analysis. [d] The aldehyde was full consumed.

1.6 Preliminary Mechanistic Study

Radical trapping experiment

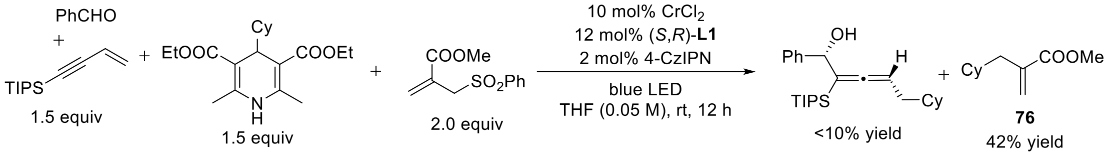

Preparation of the catalyst solution: In a nitrogen-filled glovebox, and oven-dried 20 mL vial with a magnetic stir bar, were charged the $CrCl_2$ (5.0 mg, 0.04 mmol, 10 mol%) and (*S,R*)-**L1** (23.2 mg, 0.048 mmol, 12 mol%). Then 8.0 mL THF was added and the vial was closed with a PTFE septum cap, and then stirred at room temperature for 2 h.

Catalytic asymmetric radical difunctionalization of 1,3-enynes: In a nitrogen-filled glovebox, to the prepared catalyst solution were added the 1,3-enyne (0.6 mmol, 1.5 equiv), the aldehyde (0.4 mmol, 1.0 equiv), the DHP ester (0.6 mmol, 1.5 equiv), methyl 2-((phenylsulfonyl)methyl)acrylate (192 mg, 0.8 mmol, 2.0 equiv), and 4-CzIPN (6.4 mg, 0.008 mmol, 2 mol%) sequentially. Then the vial was closed with a PTFE septum cap, and taken out of the glovebox. Then, the reaction was irradiated with one 20 W 160-440 nm LED for 12 hours (tube 5 cm away from lights, fans for cooling, 30-35 °C). After that, the reaction mixture was concentrated and run through a short silica gel pad with hexanes/EtOAc (3:1) as the eluent. Then the solvent was removed under the reduced pressure. The product **76** was isolated by flash chromatography with 42% yield.

Data for product **76**:

^{1}H NMR (500 MHz, $CDCl_3$) δ 6.13 (d, *J* = 1.6 Hz, 1H), 5.47 (d, *J* = 1.1 Hz, 1H), 3.73 (s, 3H), 2.18 (d, *J* = 7.0 Hz, 2H), 1.71 – 1.58 (m, 5H), 1.49 – 1.37 (m, 1H), 1.27 – 1.07 (m, 3H), 0.92 – 0.78 (m, 2H).

^{13}C NMR (126 MHz, $CDCl_3$) δ 168.0, 139.1, 125.7, 51.7, 39.9, 36.5, 33.0, 26.5, 26.2.

HRMS (APCI) m/z $[M + H]^+$ calcd for $C_{11}H_{19}O_2$: 183.1385, found: 183.1389.

Quantum Yield Analysis

Determination of the light intensity at 440 nm:

Following a literature procedure of Yoon (*6*), the photon flux of the spectrophotometer was determined by standard ferrioxalate actinometry. A 0.15 M solution of ferrioxalate was prepared by dissolving 2.21 g of potassium ferrioxalate hydrate in 30 mL of 0.05 M H_2SO_4. A buffered solution of phenanthroline was prepared by dissolving 50 mg of phenanthroline and 11.25 g of sodium acetate in 50 mL of 0.5 M H_2SO_4. Both solutions were stored in the

dark. To determine the photon flux of the spectrophotometer, 2.0 mL of the ferrioxalate solution was placed in a cuvette and irradiated for 90.0 seconds at λ = 440 nm with an emission slit width at 10.0 nm. After irradiation, 0.35 mL of the phenanthroline solution was added to the cuvette. The solution was then allowed to rest for 1 h to allow the ferrous ions to completely coordinate to the phenanthroline. The absorbance of the solution was measured at 510 nm. A non-irradiated sample was also prepared and the absorbance at 510 nm measured. Conversion was calculated using eq 1.

$$\text{mol } Fe^{2+} = \frac{V \bullet \Delta A}{l \bullet \varepsilon} \quad (1)$$

Where V is the total volume (0.00235 L) of the solution after addition of phenanthroline, ΔA is the difference in absorbance at 510 nm between the irradiated and non-irradiated solutions, l is the path length (1.000 cm), and ε is the molar absorptivity at 510 nm (11,100 L mol–1 cm–1). The photon flux can be calculated using eq 2.

$$\text{photon flux} = \frac{\text{mol } Fe^{2+}}{\Phi \bullet t \bullet f} \quad (2)$$

Where Φ is the quantum yield for the ferrioxalate actinometer (1.01 for a 0.15 M solution at λ = 436 nm), t is the time (90.0 s), and f is the fraction of light absorbed at λ = 436 nm (0.99833, *vide infra*). The photon flux was calculated to be 4.5936×10^{-9} einstein s^{-1}.

Sample calculation:

$$\text{mol } Fe^{2+} = \frac{0.00235 \text{ L} \bullet 1.969}{1.0 \text{ cm} \bullet 11100 \text{ L mol}^{-1} \text{ cm}^{-1}} = 4.1686 \times 10^{-7} \text{ mol}$$

$$\text{photon flux} = \frac{4.1686 \times 10^{-7} \text{ mol}}{1.01 \bullet 90.0 \text{ s} \bullet 0.99833} = 4.5936 \times 10^{-9} \text{ einstein s}^{-1}$$

Determination of the reaction quantum yield:

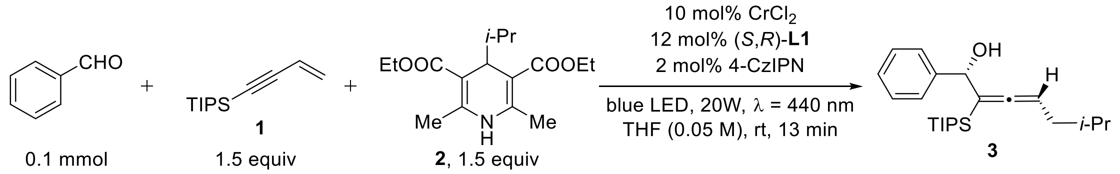

Preparation of the catalyst solution: In a nitrogen-filled glovebox, and oven-dried 4 mL vial with a magnetic stir bar, were charged the $CrCl_2$ (1.3 mg, 0.01 mmol, 10 mol%) and (*S*,*R*)-**L1** (5.8 mg, 0.012 mmol, 12 mol%). Then 2.0 mL THF was added and the vial was closed with a PTFE septum cap, and then stirred at room temperature for 2 h.

Catalytic asymmetric radical difunctionalization of 1,3-enynes: In a nitrogen-filled glovebox, to the prepared catalyst solution were added the 1,3-enyne **1** (0.15 mmol, 1.5 equiv), benzaldehyde (0.1 mmol, 1.0 equiv), the DHP ester **2** (0.15 mmol, 1.5 equiv), and 4-CzIPN (1.6 mg, 0.002 mmol, 2 mol%)

sequentially. Then the vial was closed with a PTFE septum cap, and taken out of the glovebox. The reaction mixture was stirred and irradiated with a single blue LED (20 W, λ_{max} = 440 nm) for 13 min. After irradiation, the yield was determined by GC-FID analysis using dodecane as an internal standard. The yield was determined to be 1.3% (1.25×10^{-6} mol). The reaction quantum yield (Φ) was determined using eq. 3 where the photon flux is 4.5936×10^{-9} einsteins s^{-1} (determined by actinometry as described above), t is the reaction time (780 s) and f_R is the fraction of incident light absorbed by the reaction mixture. An absorption spectrum of the reaction mixture gave an absorbance value of > 3 at 440 nm, indicating that essentially all the incident light (f_R > 0.999) is absorbed by the photocatalyst.

$$\Phi = \frac{\text{n (product)}}{\text{photon flux} \bullet t \bullet f} \quad (3)$$

The reaction quantum yield (Φ) was thus determined to be Φ = 0.35.

Light on-off experiments

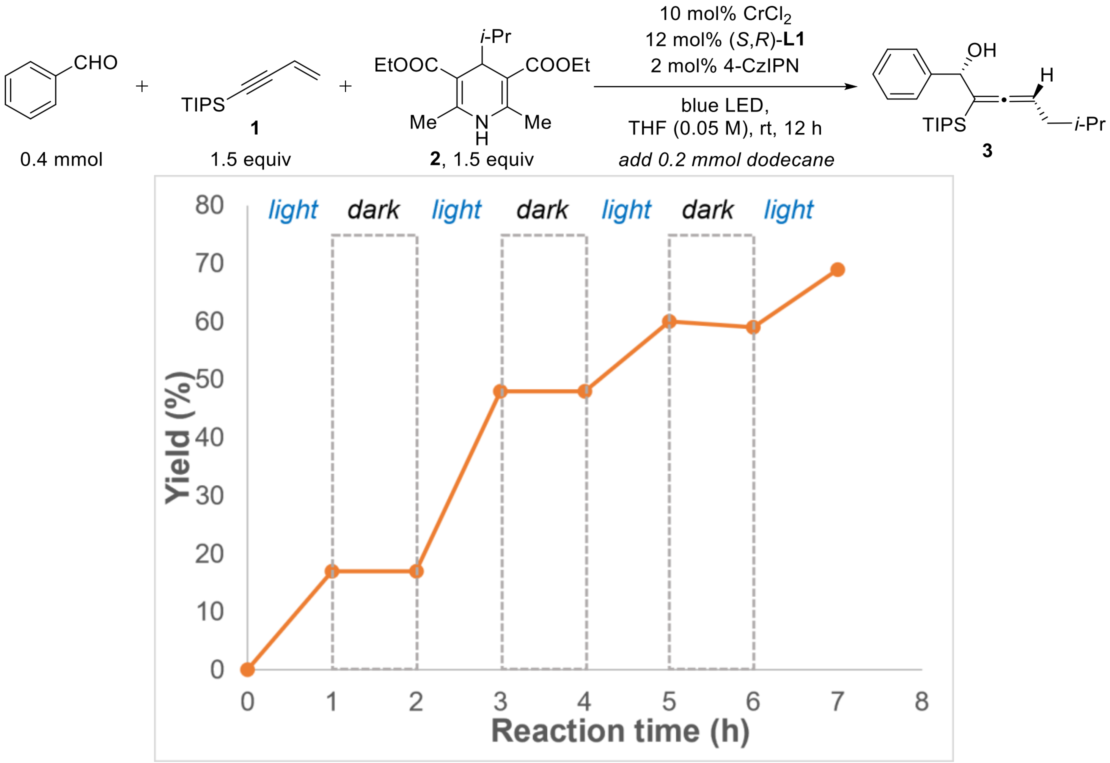

Supplementary Figure 2. Light on-off experiments

Preparation of the catalyst solution: In a nitrogen-filled glovebox, and oven-dried 20 mL vial with a magnetic stir bar, were charged the $CrCl_2$ (5.0 mg, 0.04 mmol, 10 mol%) and (*S*,*R*)-**L1** (23.2 mg, 0.048 mmol, 12 mol%). Then 8.0 mL THF was added and the vial was closed with a PTFE septum cap, and then stirred at room temperature for 2 h.

Catalytic asymmetric radical difunctionalization of 1,3-enynes: In a nitrogen-filled glovebox, to the prepared catalyst solution were added the 1,3-enyne **1** (0.6 mmol, 1.5 equiv), benzaldehyde (0.4 mmol, 1.0 equiv), the DHP ester **2** (0.6 mmol, 1.5 equiv), dodecane (0.2 mmol), and 4-CzIPN (6.4 mg, 0.008 mmol, 2 mol%) sequentially. Then the vial was closed with a PTFE septum cap, and taken out of the glovebox. Then, the reaction was irradiated with one 20 W 160-440 nm LED for 1 hour (tube 5 cm away from lights, fans for cooling, 30-35 °C). After that, light was turned off, sample (200 μL) was taken with a syringe and analyzed by GC to determine the yields. And the reaction was stirred in the dark for 1 hour. And sample (200 μL) was taken with a syringe and analyzed by GC to determine the yields. Above light on-off experiments were conducted for another three times.

Stern-Volmer fluorescence quenching experiments

A Hitachi F-7000 fluoresence spectrometer was used to record the emission intensities. All 4-CzIPN solutions were excited at 440 nm and the emission intensity at 531 nm was observed. THF was degassed with a stream of N_2 for 30 min. In a typical experiment, the emission spectrum of a 2×10^{-5} M solution of 4-CzIPN in THF was collected. Then, appropriate amount of quencher was added to the measured solution in a quartz cuvette and the emission spectrum of the sample was collected. I_0 and I represent the intensities of the emission in the absence and presence of the quencher at 531 nm.

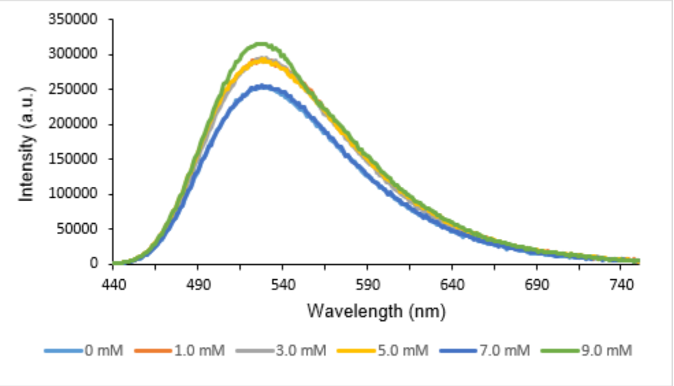

Supplementary Figure 3. Emission spectra of 2×10^{-5} M 4-CzIPN at λ_{ex} = 440nm showing the quenching effect of increasing of enyne **1**.

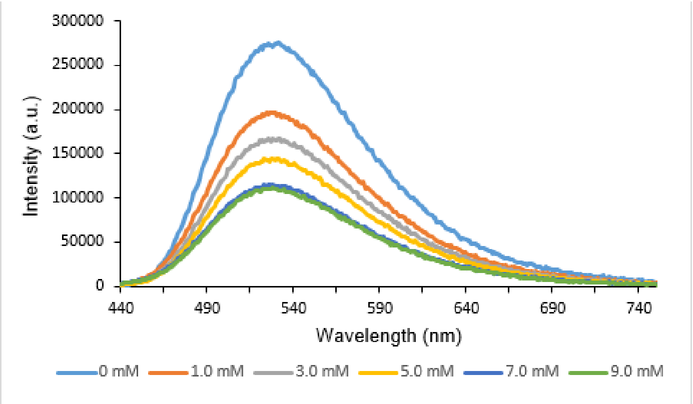

Supplementary Figure 4. Emission spectra of 2×10^{-5} M 4-CzIPN at λ_{ex} = 440nm showing the quenching effect of increasing of DHP ester **2**.

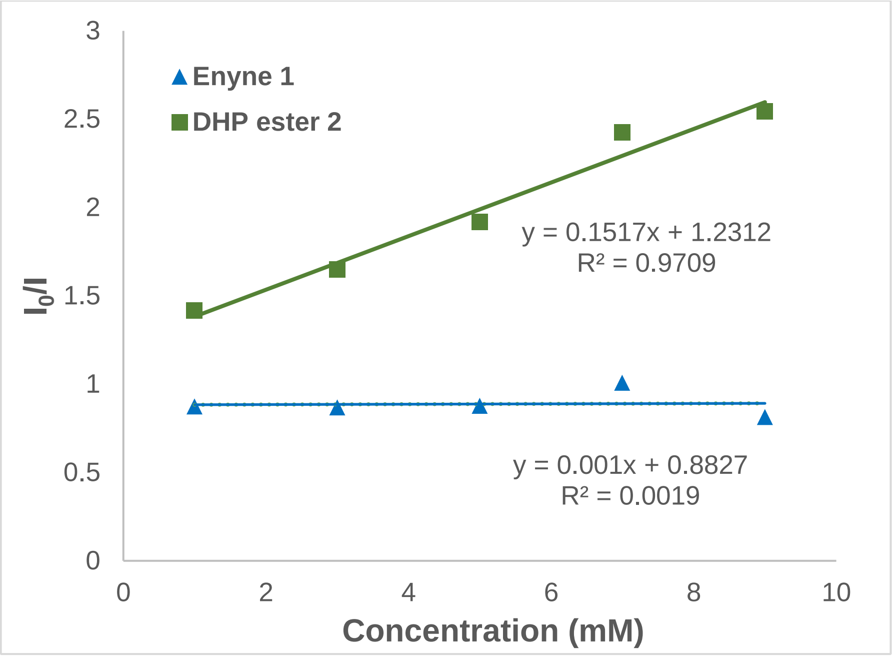

Supplementary Figure 5. The Stern-Volmer plot.

1.7 Assignment of the Absolute Configuration

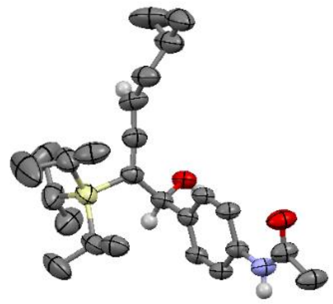

Supplementary Figure 6. Thermal ellipsoid plot at the 50% probability level. Hydrogen atoms are omitted for clarity. Metrical parameters for the structure of **12** are available free of charge from the Cambridge Crystallographic Data Centre (https://www.ccdc.cam.ac.uk/) under reference number CCDC 2130059.

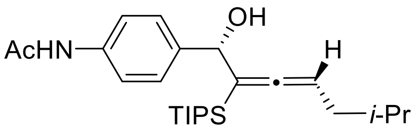

***N*-(4-((1*S*,3*S*)-1-hydroxy-6-methyl-2-(triisopropylsilyl)hepta-2,3-dien-1-yl)phenyl)acetamide (Figure 3, entry 12).** X-ray quality crystals were obtained by slow evaporation of a saturated solution in CH_2Cl_2/hexanes of a sample synthesized with (*S,R*)-**L1**. A single crystal of $C_{25}H_{41}NO_2Si$ was selected and mounted in a nylon loop in parabar oil. All measurements were performed on a Bruker Photon III diffractometer with filtered Cu-Kα radiation at a temperature of 100 K. Using Olex2 (*7*), the structure was solved with the ShelXS structure solution program (*8*) using Direct Methods and refined with the ShelXL refinement package (*9*) using Least Squares minimization. The absolute stereochemistry was determined on the basis of the absolute structure parameter. Crystal data, data collection parameters, and structure refinement details are given in Supplementary Table 2.

Supplementary Table 2. Crystal data and structure refinement for **12**.

Identification code	cu-full-no7_a	
Empirical formula	C6.25 H10.25 N0.25 O0.50 Si0.25	
Formula weight	103.92	
Temperature	100(2) K	
Wavelength	1.54178 Å	
Crystal system	Orthorhombic	
Space group	$P2_12_12_1$	
Unit cell dimensions	a = 8.5909(3) Å	a= 90°.
	b = 11.9281(5) Å	b= 90°.
	c = 26.4715(13) Å	g = 90°.

Volume	2712.6(2) Å^3
Z	16
Density (calculated)	1.018 Mg/m^3
Absorption coefficient	0.888 mm^{-1}
F(000)	912
Crystal size	0.54 x 0.08 x 0.065 mm^3
Theta range for data collection	3.339 to 66.797°.
Index ranges	-10<=h<=9, -14<=k<=14, -30<=l<=31
Reflections collected	28257
Independent reflections	4809 [R(int) = 0.1119]
Completeness to theta = 66.797°	99.9 %
Absorption correction	Semi-empirical from equivalents
Max. and min. transmission	0.7528 and 0.5118
Refinement method	Full-matrix least-squares on F^2
Data / restraints / parameters	4809 / 2 / 278
Goodness-of-fit on F^2	1.048
Final R indices [I>2sigma(I)]	R1 = 0.0647, wR2 = 0.1717
R indices (all data)	R1 = 0.0984, wR2 = 0.1981
Absolute structure parameter	-0.01(3)
Extinction coefficient	0.0038(9)
Largest diff. peak and hole	0.336 and -0.196 e.Å^{-3}

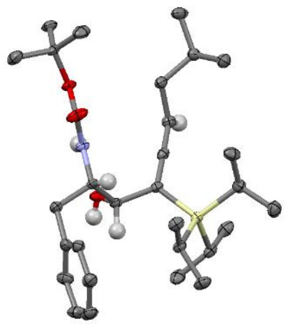

Supplementary Figure 7. Thermal ellipsoid plot at the 50% probability level. Hydrogen atoms are omitted for clarity. Metrical parameters for the structure of **42** are available free of charge from the Cambridge Crystallographic Data Centre (https://www.ccdc.cam.ac.uk/) under reference number CCDC 2130062.

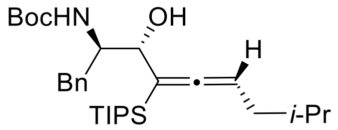

***tert*-butyl ((2*R*,3*S*,5*S*)-3-hydroxy-8-methyl-1-phenyl-4-(triisopropylsilyl)nona-4,5-dien-2-yl)carbamate (Figure 3, entry 42).** X-ray quality crystals were obtained by slow evaporation of a saturated solution in CH_2Cl_2/hexanes of a sample synthesized with (*S*,*R*)-**L1**. A single crystal of

$C_{30}H_{51}NO_3Si$ was selected and mounted in a nylon loop in parabar oil. All measurements were performed on a Bruker Photon III diffractometer with filtered Mo-Kα radiation at a temperature of 100 K. Using Olex2 (*5*), the structure was solved with the ShelXS structure solution program (*6*) using Direct Methods and refined with the ShelXL refinement package (*7*) using Least Squares minimization. The absolute stereochemistry was determined on the basis of the absolute structure parameter. Crystal data, data collection parameters, and structure refinement details are given in Supplementary Table 3.

Supplementary Table 3. Crystal data and structure refinement for **42**.

Identification code	mo_ZFH_0930_3962_0m_a	
Empirical formula	C30 H51 N O3 Si	
Formula weight	501.80	
Temperature	100(2) K	
Wavelength	0.71073 Å	
Crystal system	Orthorhombic	
Space group	$P2_12_12_1$	
Unit cell dimensions	a = 10.7957(7) Å	a= 90°.
	b = 11.6038(6) Å	b= 90°.
	c = 24.5221(15) Å	g = 90°.
Volume	3071.9(3) $Å^3$	
Z	4	
Density (calculated)	1.085 Mg/m^3	
Absorption coefficient	0.105 mm^{-1}	
F(000)	1104	
Crystal size	0.1 x 0.04 x 0.03 mm^3	
Theta range for data collection	1.942 to 28.278°.	
Index ranges	-14<=h<=14, -15<=k<=12, -28<=l<=32	
Reflections collected	58595	
Independent reflections	7613 [R(int) = 0.0764]	
Completeness to theta = 25.242°	99.7 %	
Absorption correction	Semi-empirical from equivalents	
Max. and min. transmission	0.7457 and 0.6649	
Refinement method	Full-matrix least-squares on F^2	
Data / restraints / parameters	7613 / 2 / 333	
Goodness-of-fit on F^2	0.998	
Final R indices [I>2sigma(I)]	R1 = 0.0356, wR2 = 0.0814	
R indices (all data)	R1 = 0.0439, wR2 = 0.0869	
Absolute structure parameter	-0.02(5)	
Extinction coefficient	n/a	
Largest diff. peak and hole	0.227 and -0.222 e.$Å^{-3}$	

1.8 NMR Spectra

^{1}H NMR (500 MHz, room temperature, $CDCl_3$)

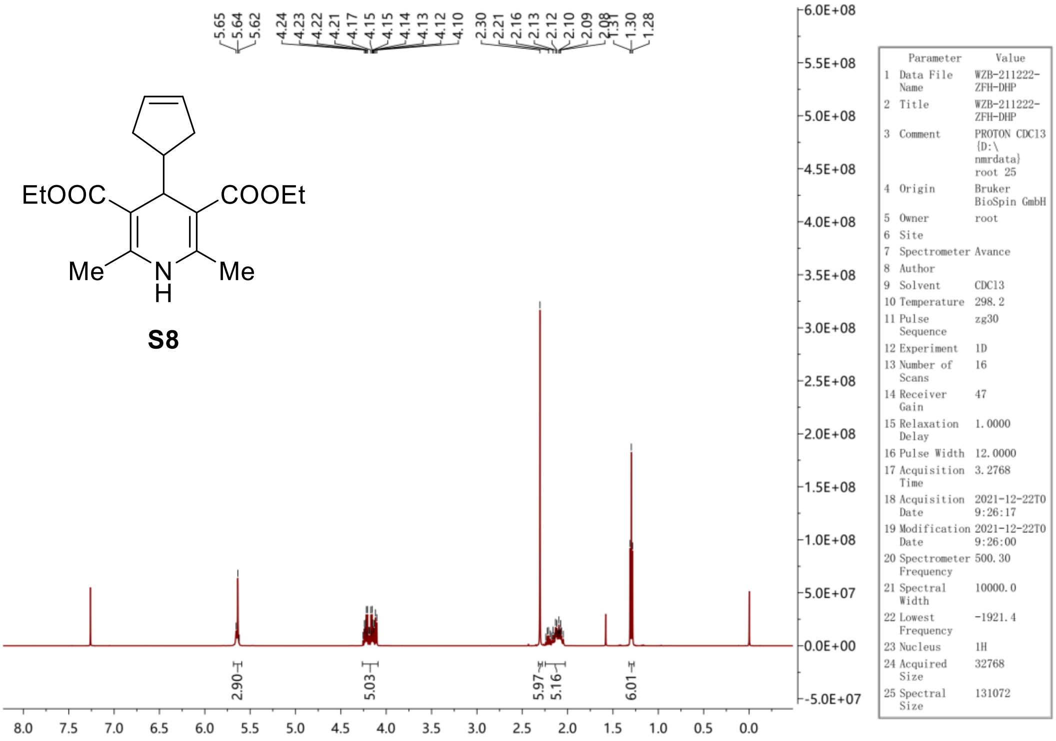

Supplementary Figure 8. ^{1}H NMR spectrum of compound **S8**

^{13}C NMR (126 MHz, room temperature, $CDCl_3$)

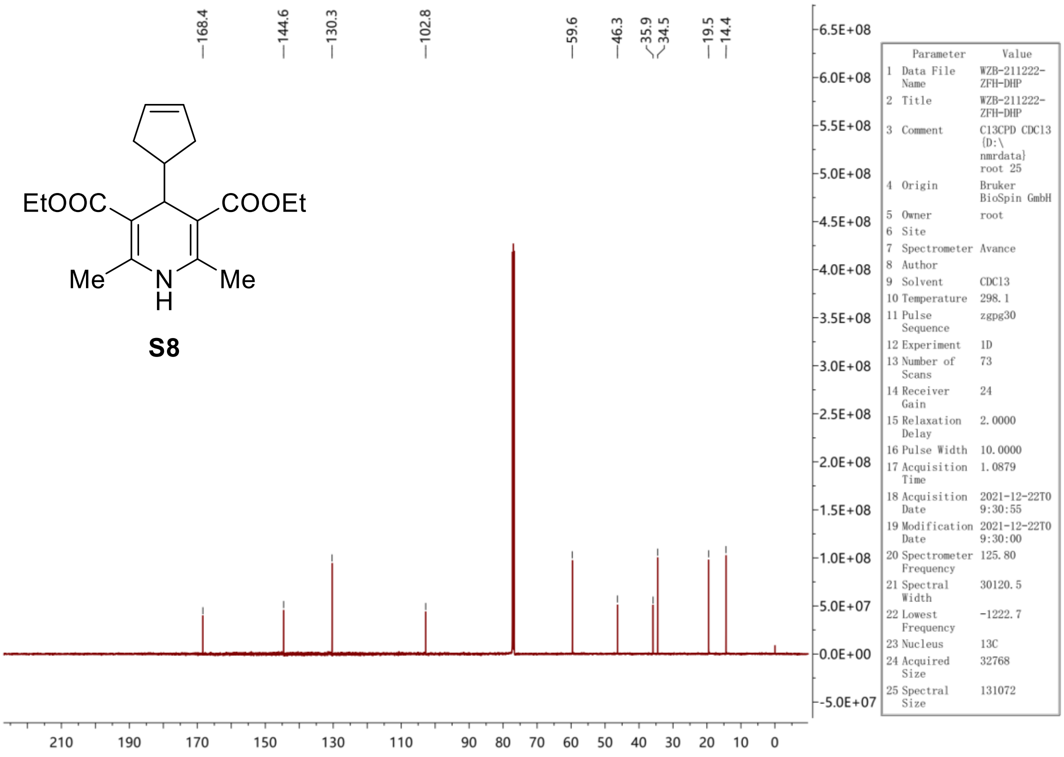

Supplementary Figure 9. ^{13}C NMR spectrum of compound **S8**

^1H NMR (500 MHz, room temperature, $CDCl_3$)

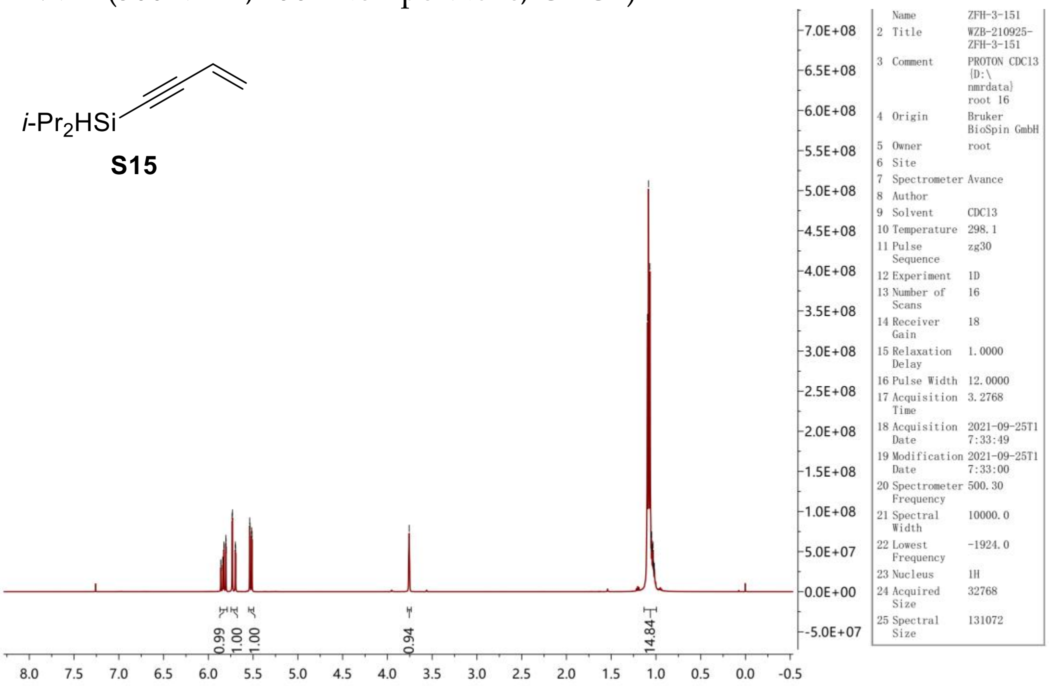

Supplementary Figure 10. ^1H NMR spectrum of compound **S15**

^{13}C NMR (126 MHz, room temperature, $CDCl_3$)

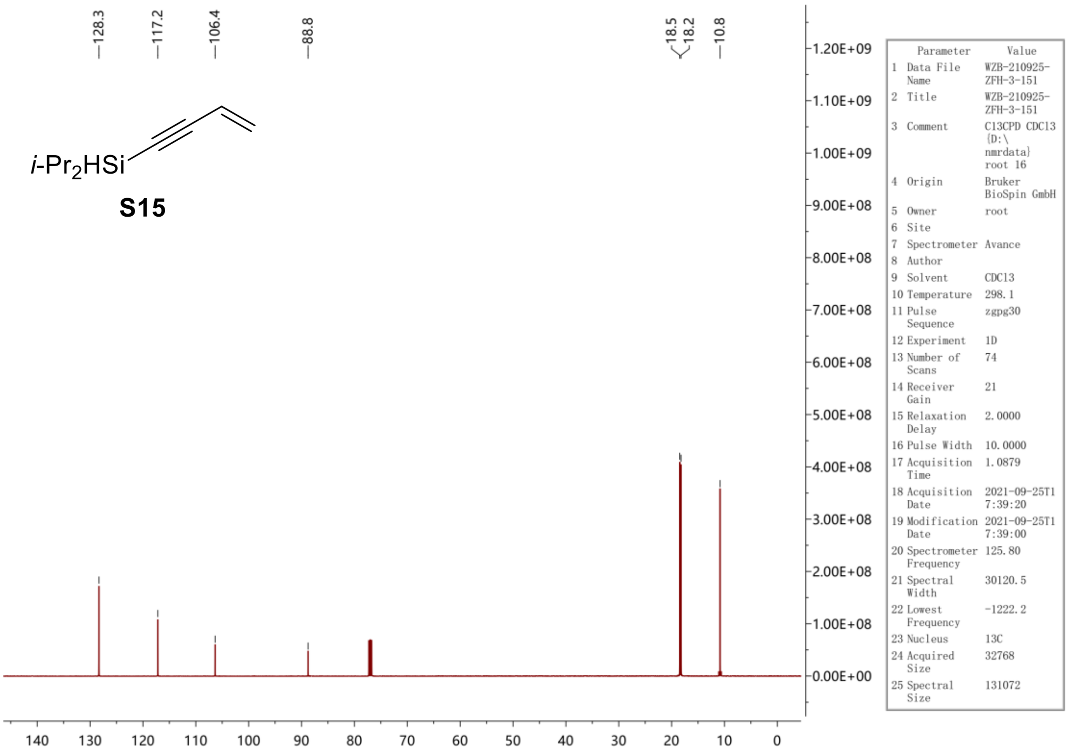

Supplementary Figure 11. ^{13}C NMR spectrum of compound **S15**

^{1}H NMR (500 MHz, room temperature, $CDCl_3$)

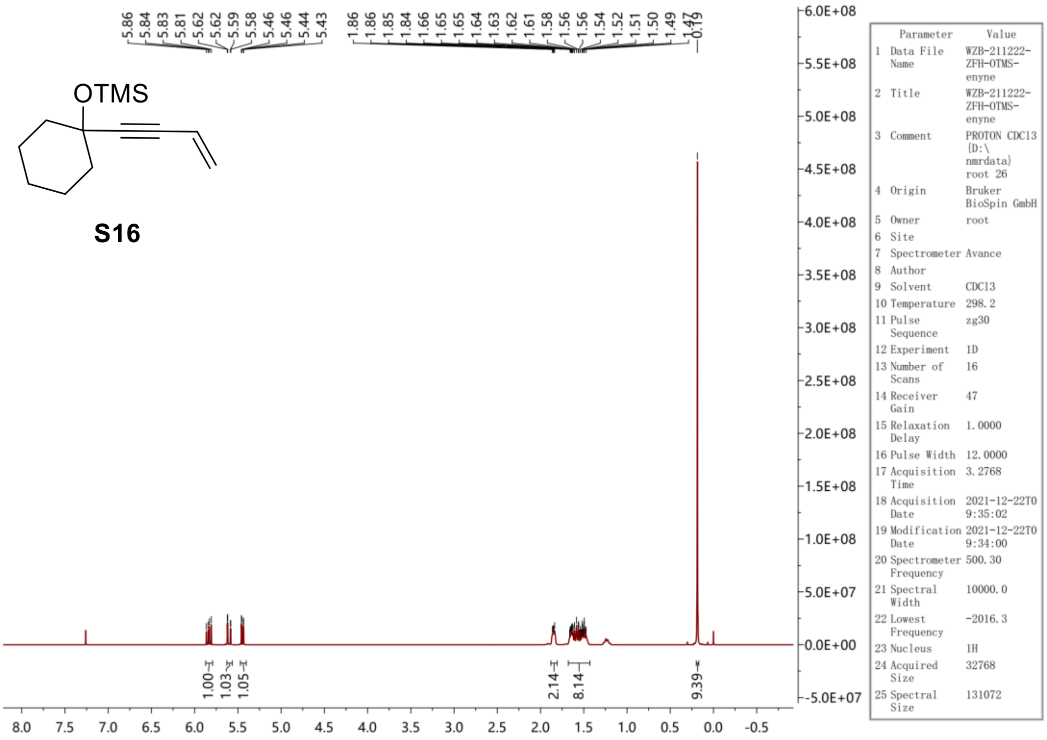

Supplementary Figure 12. ^{1}H NMR spectrum of compound **S16**

^{13}C NMR (126 MHz, room temperature, $CDCl_3$)

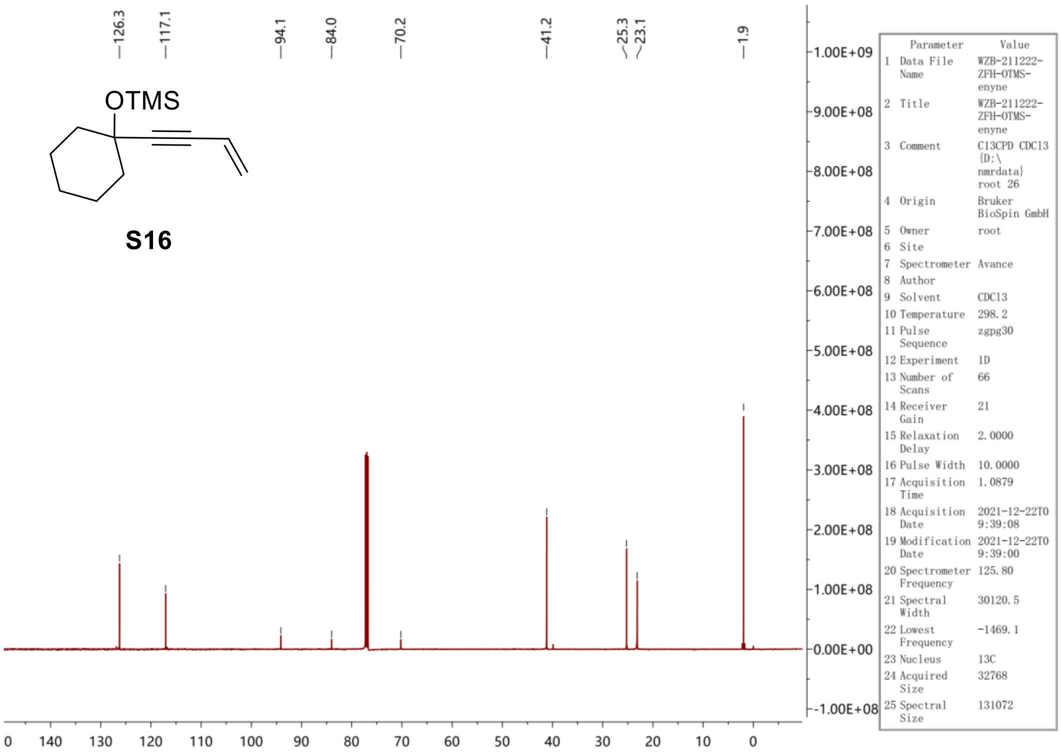

Supplementary Figure 13. ^{13}C NMR spectrum of compound **S16**

^{1}H NMR (500 MHz, room temperature, $CDCl_3$)

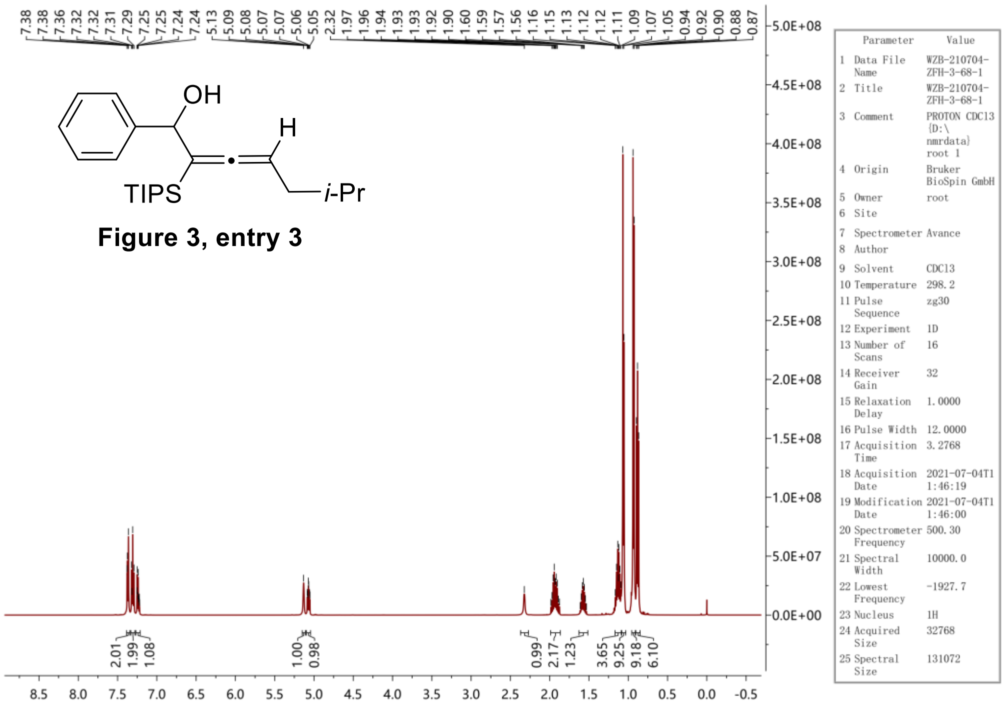

Supplementary Figure 14. ^{1}H NMR spectrum of compound **3**

^{13}C NMR (126 MHz, room temperature, $CDCl_3$)

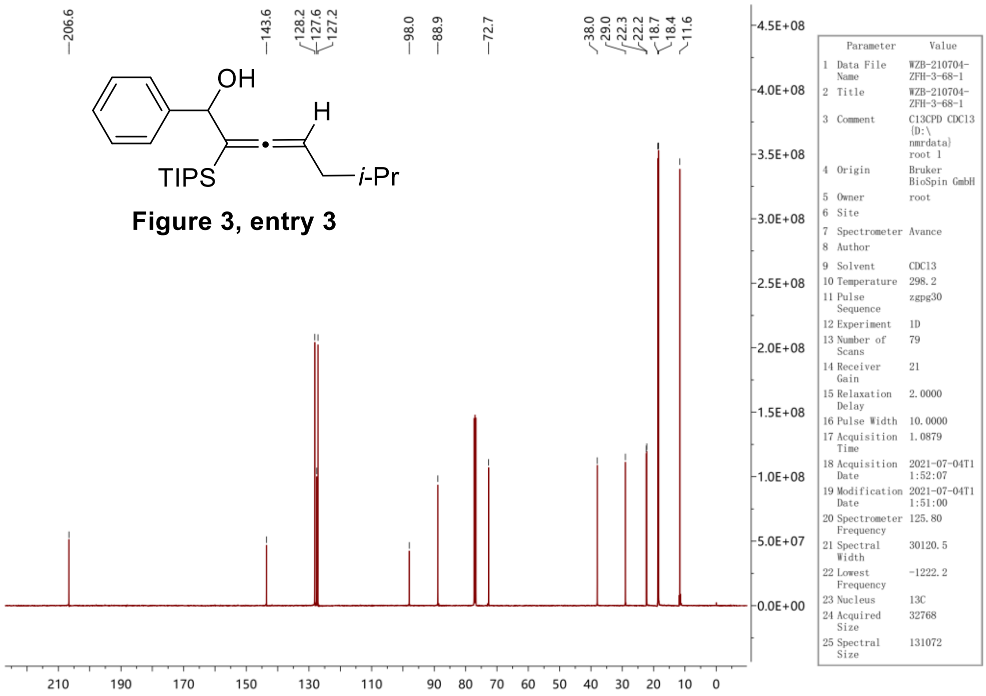

Supplementary Figure 15. ^{13}C NMR spectrum of compound **3**

^{1}H NMR (500 MHz, room temperature, $CDCl_3$)

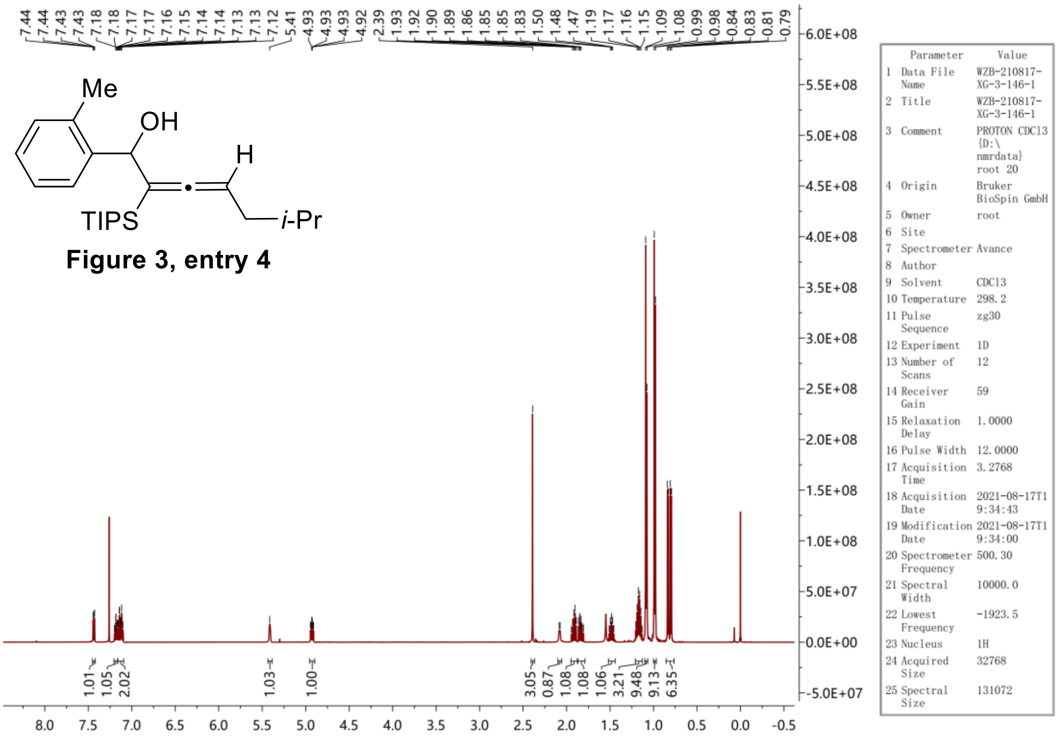

Supplementary Figure 16. ^{1}H NMR spectrum of compound **4**

^{13}C NMR (126 MHz, room temperature, $CDCl_3$)

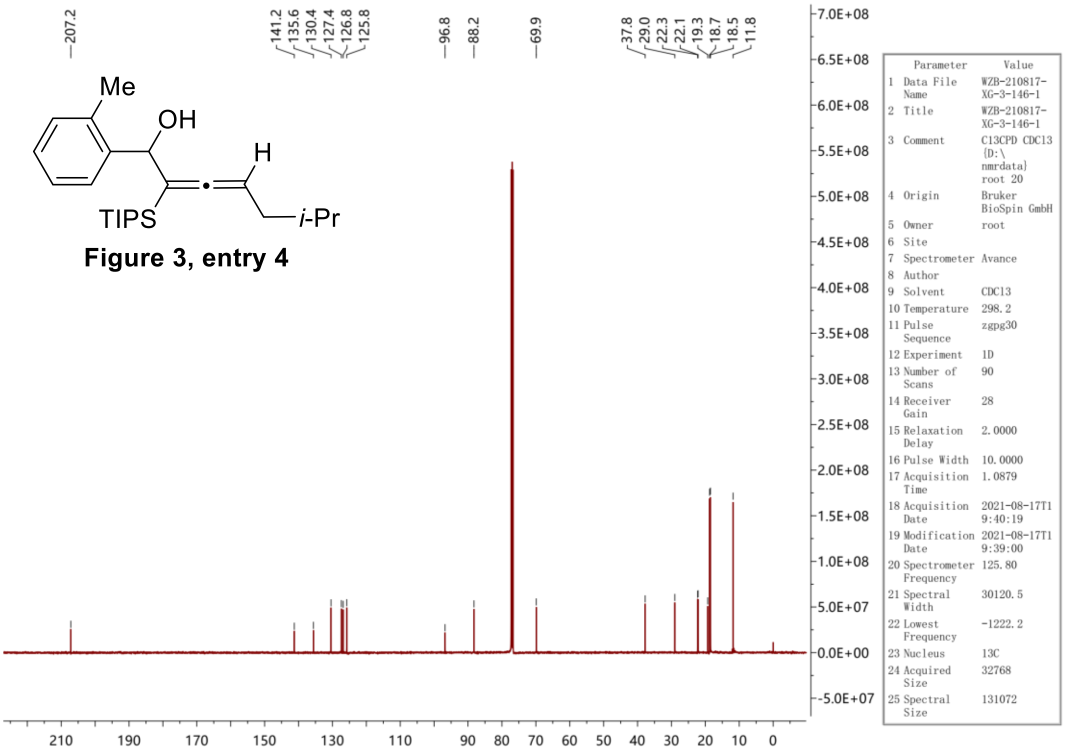

Supplementary Figure 17. ^{13}C NMR spectrum of compound **4**

^{1}H NMR (500 MHz, room temperature, $CDCl_3$)

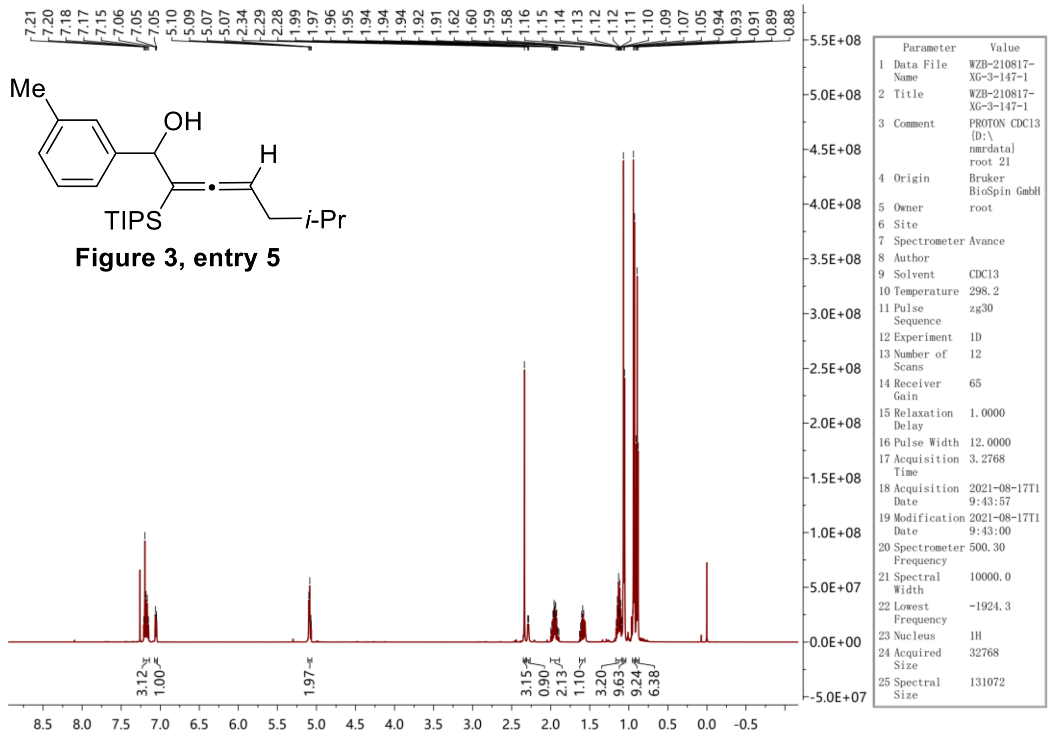

Supplementary Figure 18. ^{1}H NMR spectrum of compound **5**

^{13}C NMR (126 MHz, room temperature, $CDCl_3$)

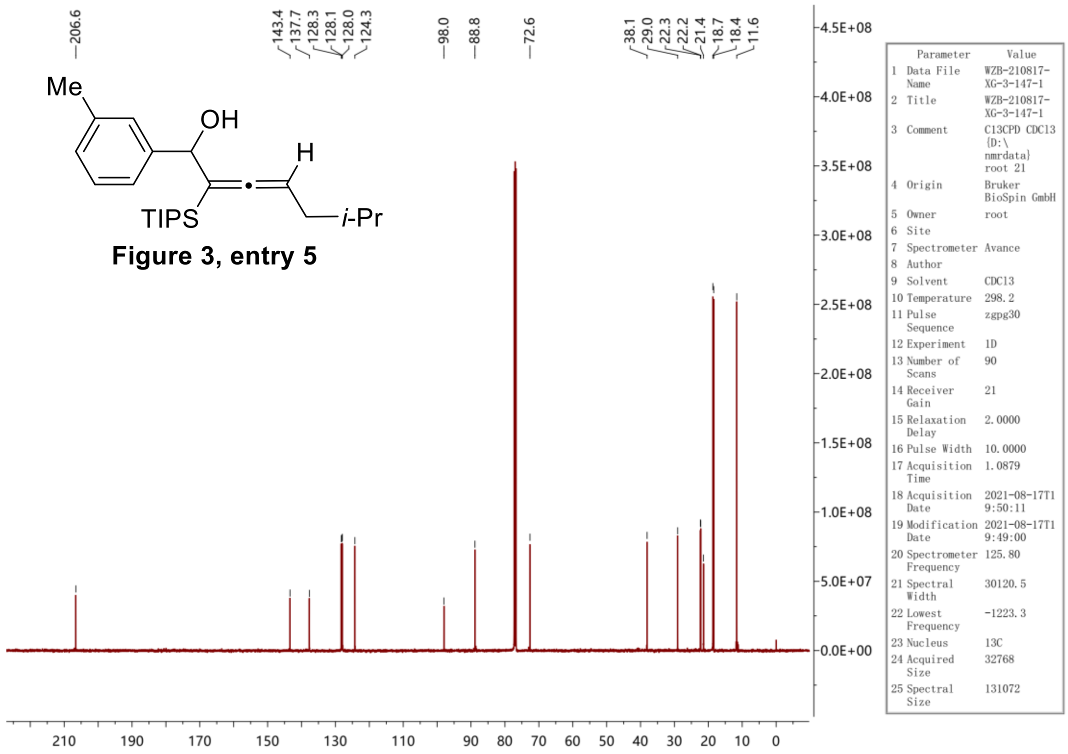

Supplementary Figure 19. ^{13}C NMR spectrum of compound **5**

^{1}H NMR (500 MHz, room temperature, $CDCl_3$)

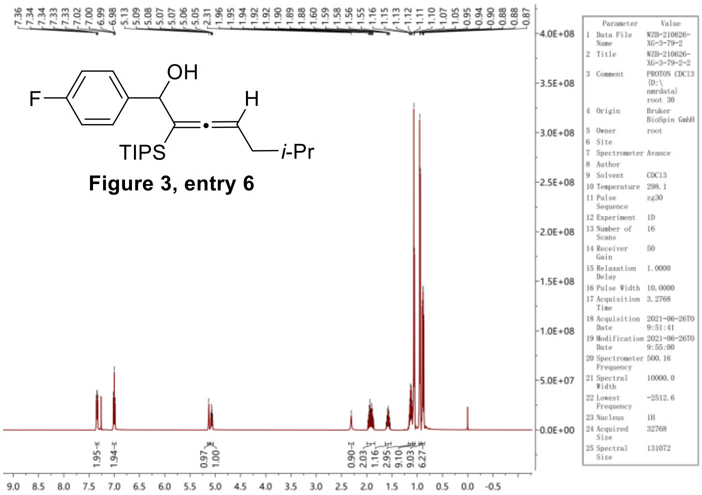

Supplementary Figure 20. ^{1}H NMR spectrum of compound **6**

^{13}C NMR (126 MHz, room temperature, $CDCl_3$)

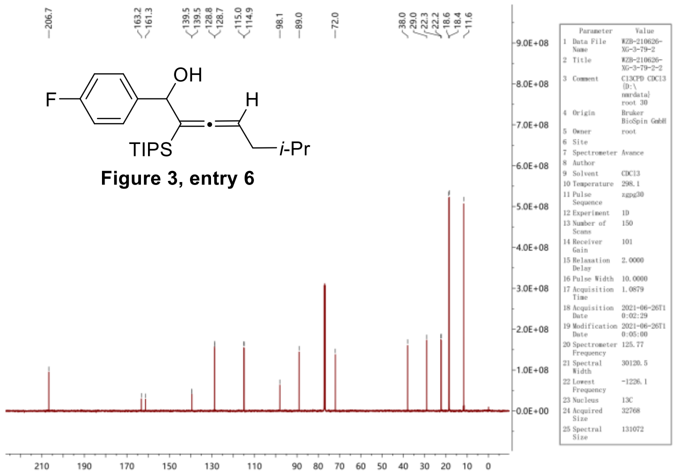

Supplementary Figure 21. ^{13}C NMR spectrum of compound **6**

^{19}F NMR (471 MHz, room temperature, $CDCl_3$)

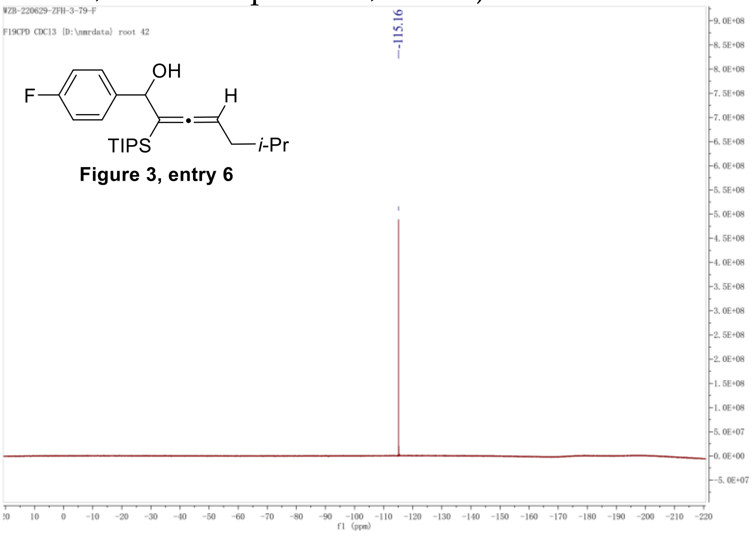

Supplementary Figure 22. ^{19}F NMR spectrum of compound **6**

^{1}H NMR (500 MHz, room temperature, $CDCl_3$)

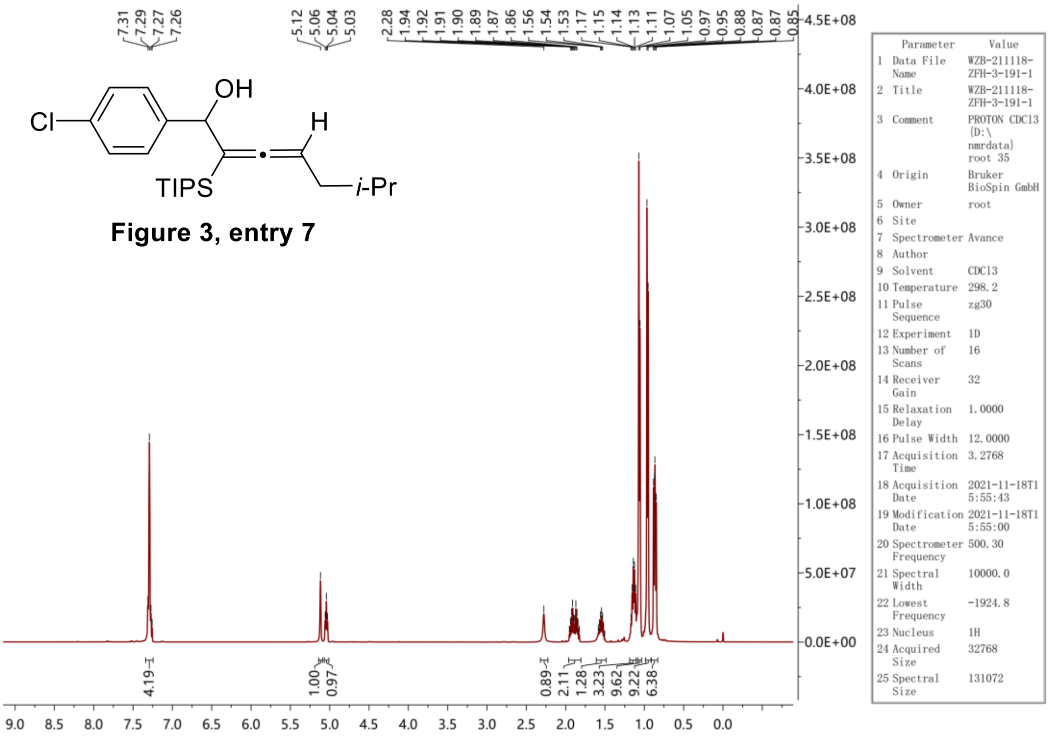

Supplementary Figure 23. ^{1}H NMR spectrum of compound **7**

^{13}C NMR (126 MHz, room temperature, $CDCl_3$)

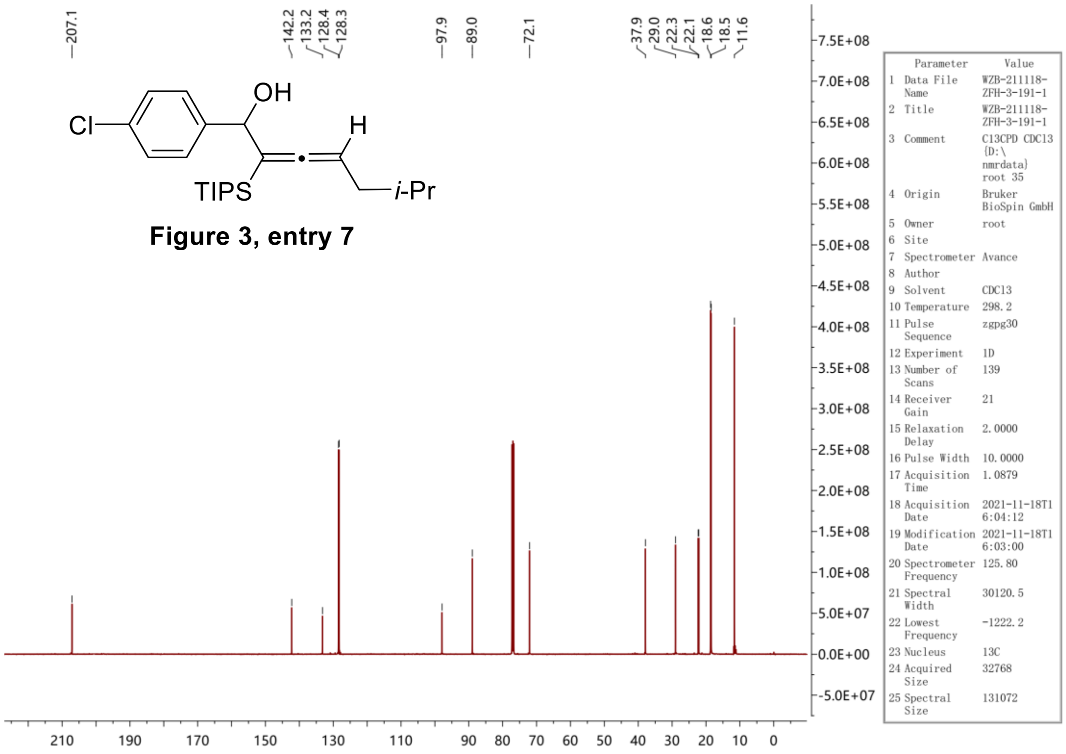

Supplementary Figure 24. ^{13}C NMR spectrum of compound **7**

^{1}H NMR (500 MHz, room temperature, $CDCl_3$)

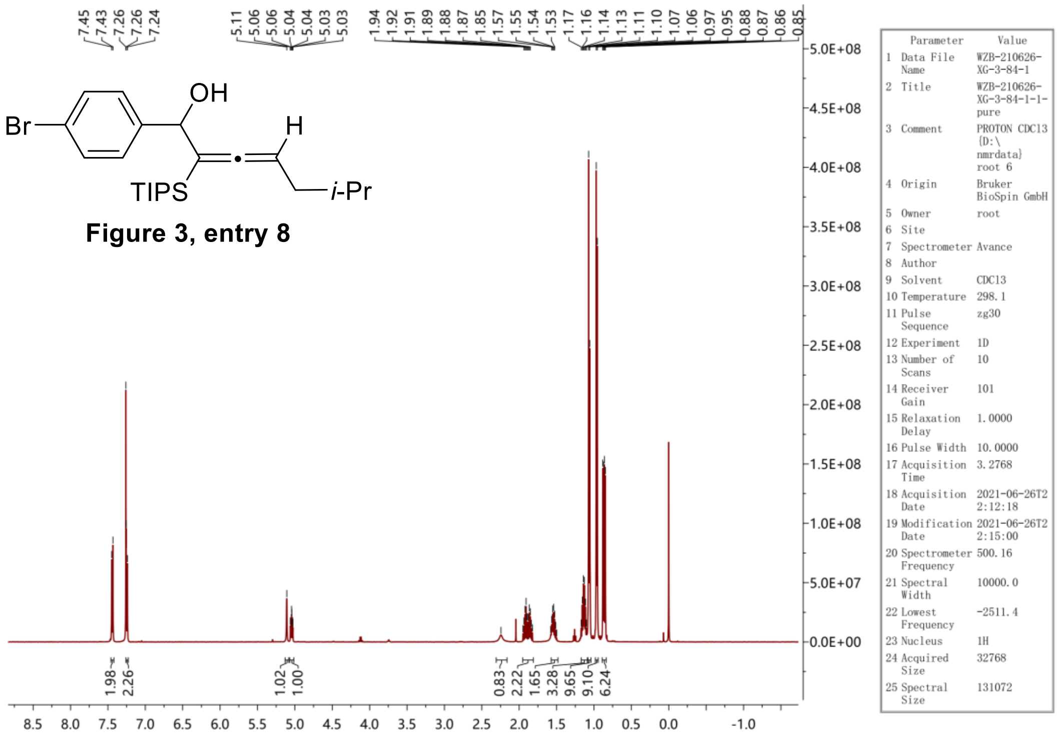

Supplementary Figure 25. ^{1}H NMR spectrum of compound **8**

^{13}C NMR (126 MHz, room temperature, $CDCl_3$)

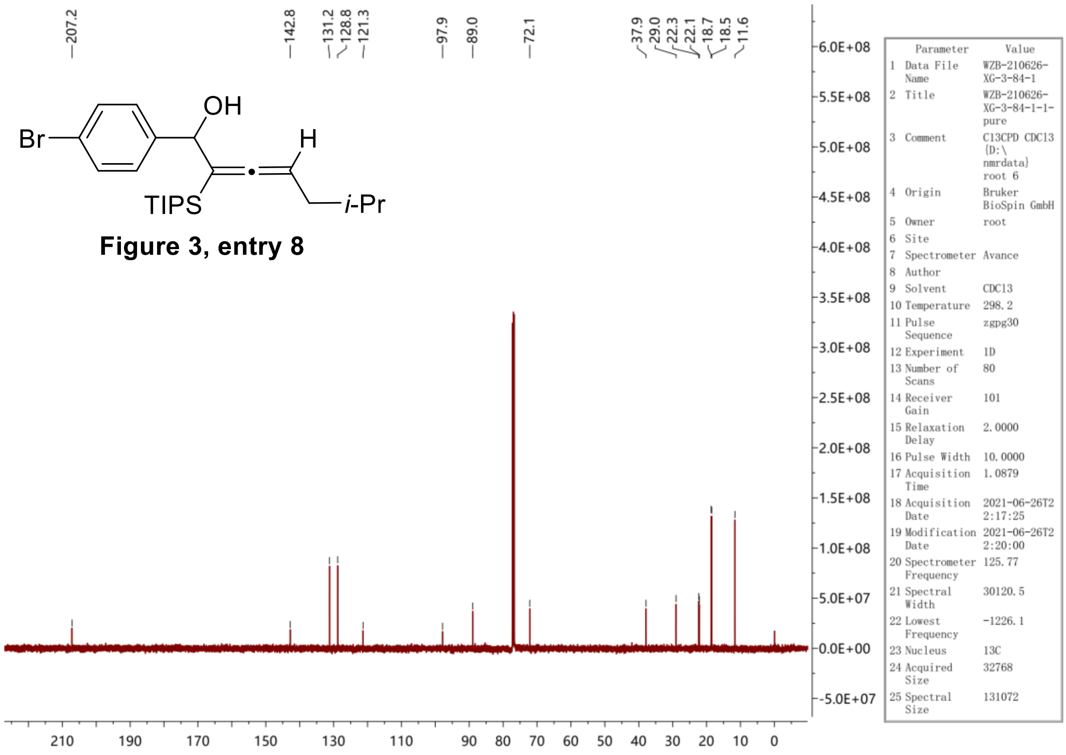

Supplementary Figure 26. ^{13}C NMR spectrum of compound **8**

^{1}H NMR (500 MHz, room temperature, $CDCl_3$)

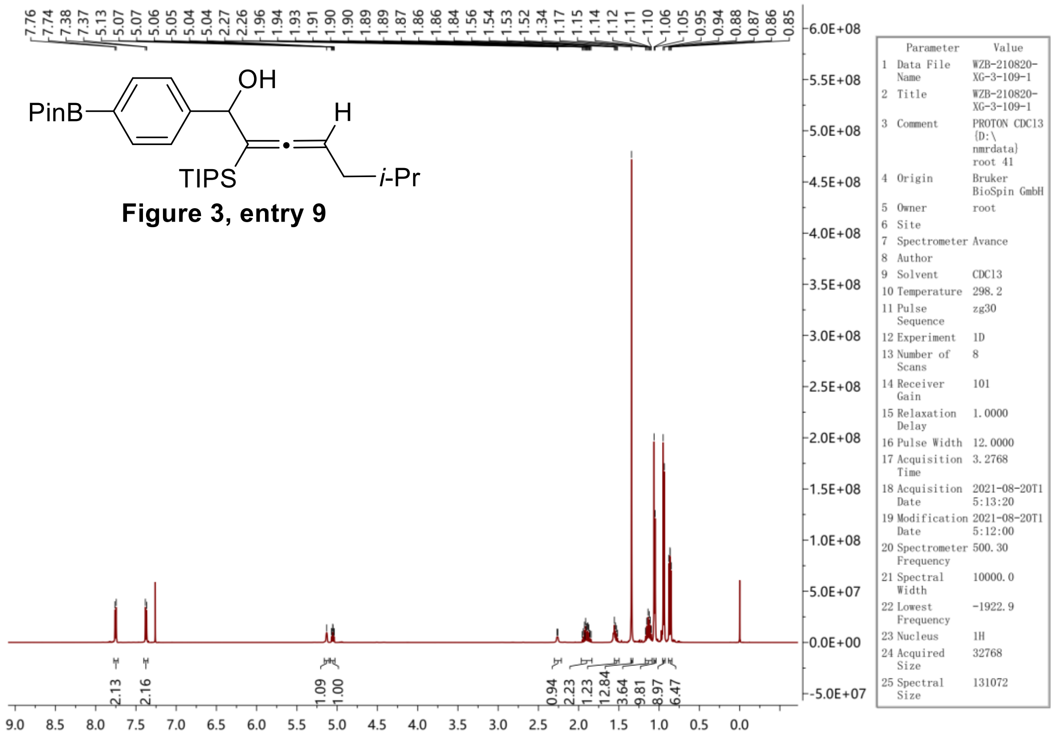

Supplementary Figure 27. ^{1}H NMR spectrum of compound **9**

^{13}C NMR (126 MHz, room temperature, $CDCl_3$)

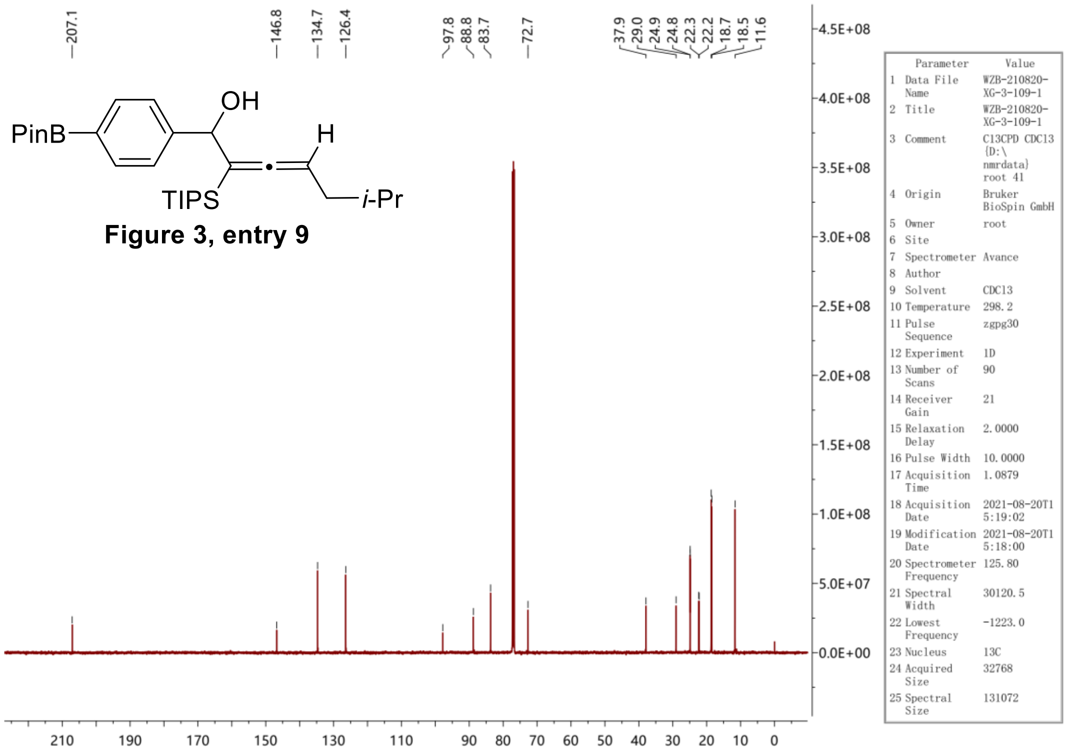

Supplementary Figure 28. ^{13}C NMR spectrum of compound **9**

^{1}H NMR (500 MHz, room temperature, $CDCl_3$)

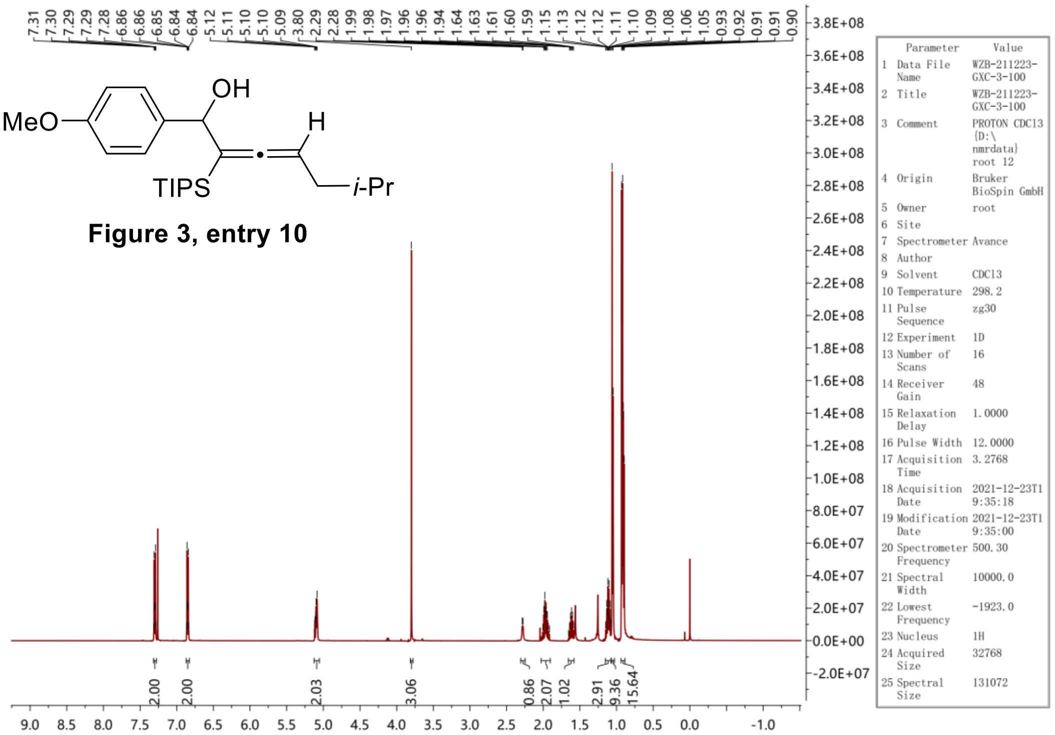

Supplementary Figure 29. ^{1}H NMR spectrum of compound **10**

^{13}C NMR (126 MHz, room temperature, $CDCl_3$)

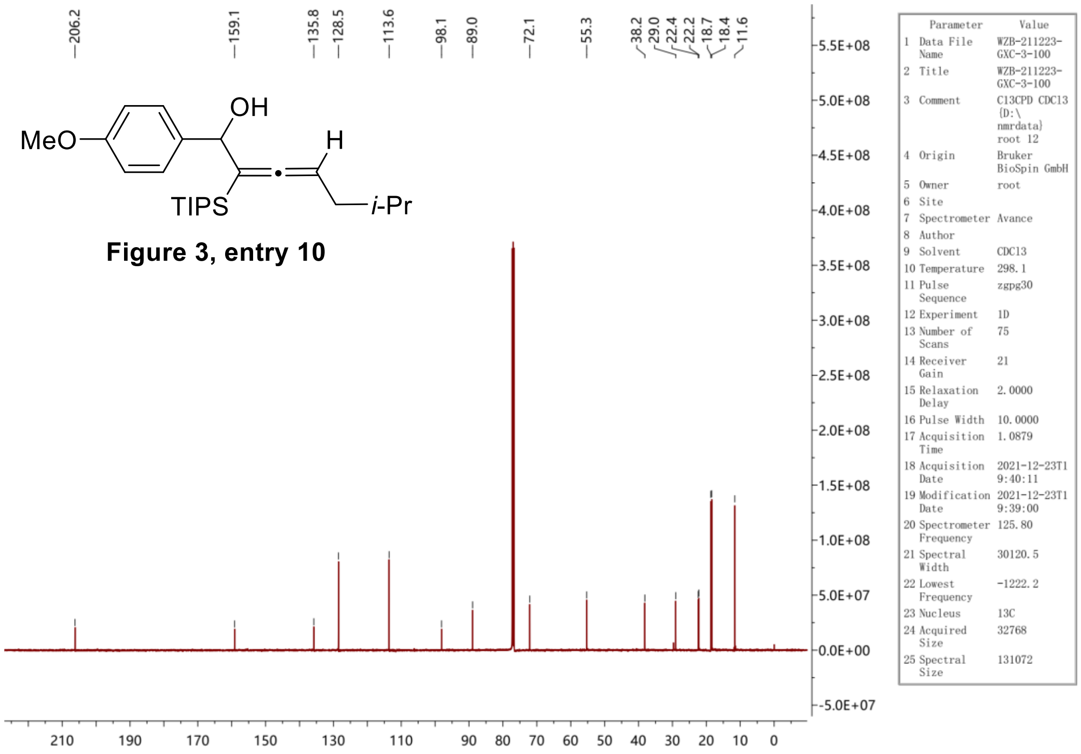

Supplementary Figure 30. ^{13}C NMR spectrum of compound **10**

^{1}H NMR (500 MHz, room temperature, $CDCl_3$)

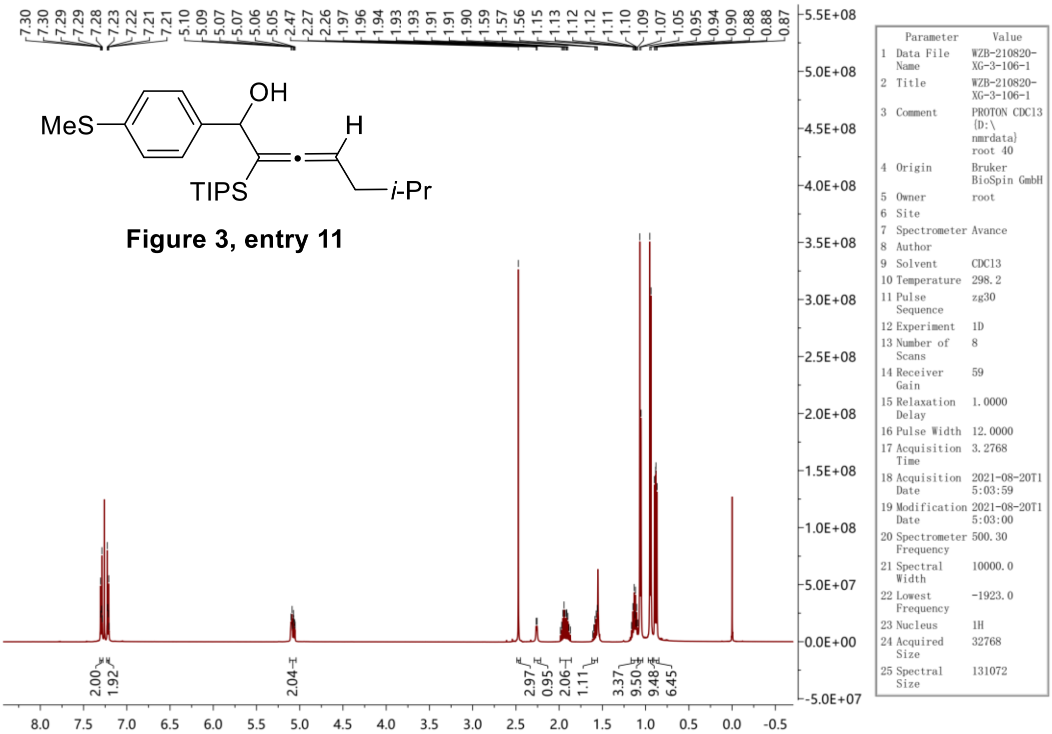

Supplementary Figure 31. ^{1}H NMR spectrum of compound **11**

^{13}C NMR (126 MHz, room temperature, $CDCl_3$)

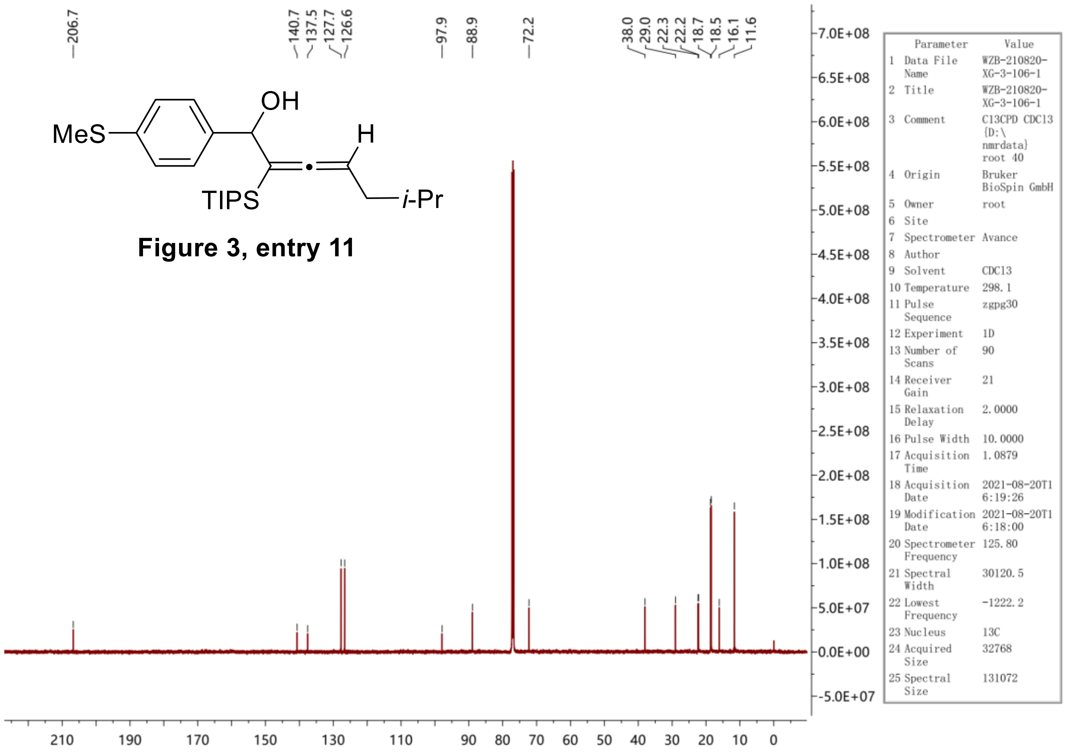

Supplementary Figure 32. ^{13}C NMR spectrum of compound **11**

^1H NMR (500 MHz, room temperature, $CDCl_3$)

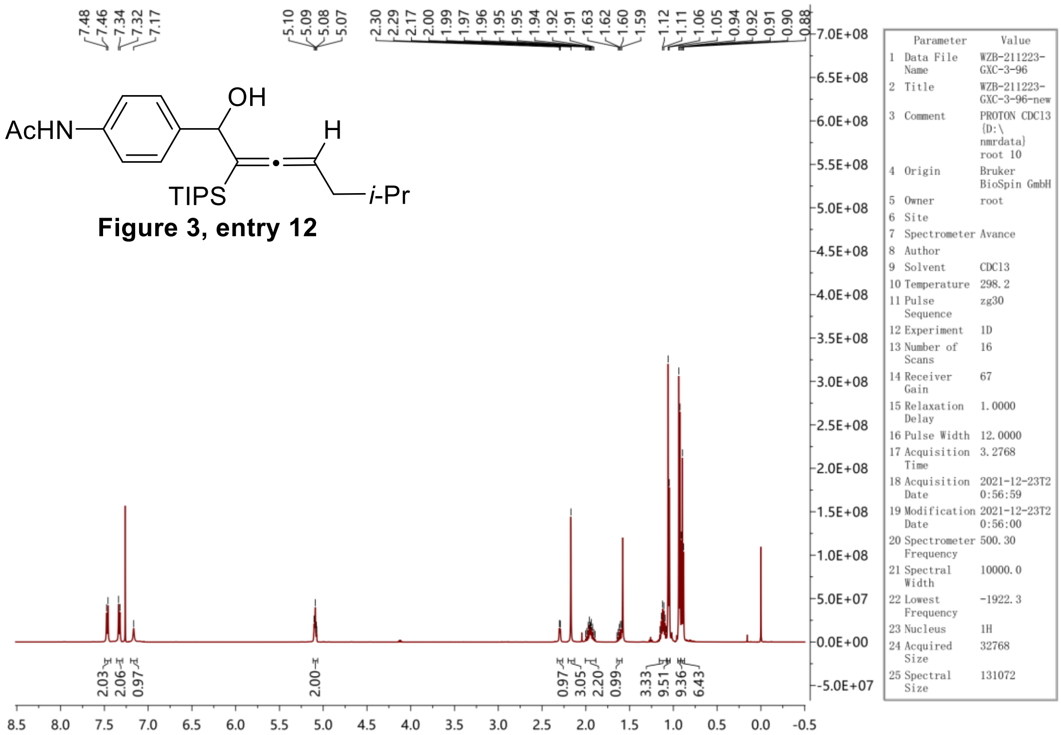

Supplementary Figure 33. ^1H NMR spectrum of compound **12**

^{13}C NMR (126 MHz, room temperature, $CDCl_3$)

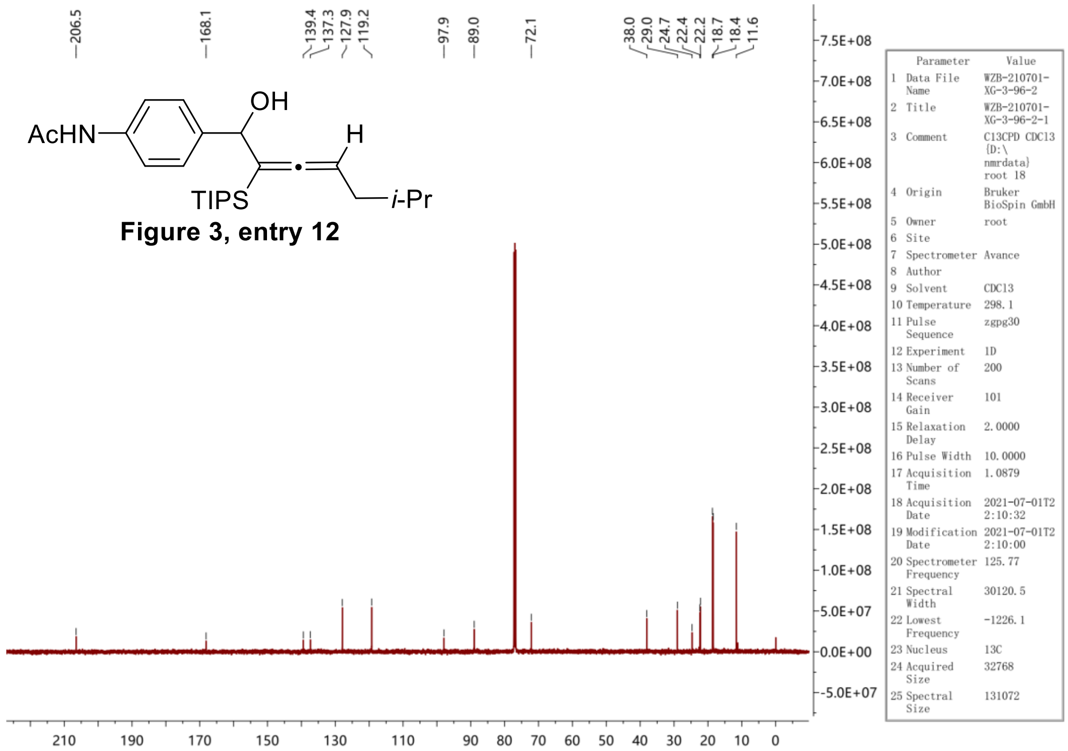

Supplementary Figure 34. ^{13}C NMR spectrum of compound **12**

^{1}H NMR (500 MHz, room temperature, $CDCl_3$)

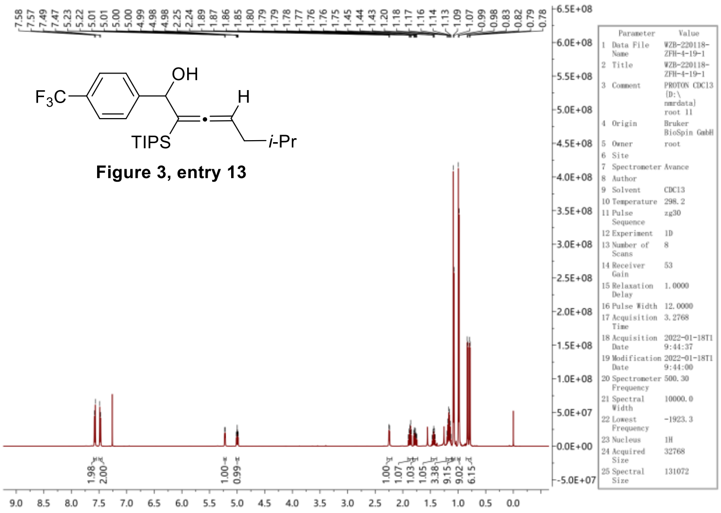

Supplementary Figure 35. ^{1}H NMR spectrum of compound **13**

^{13}C NMR (126 MHz, room temperature, $CDCl_3$)

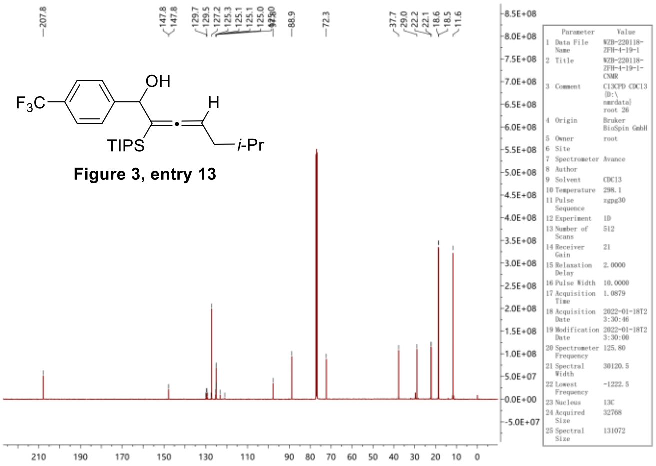

Supplementary Figure 36. ^{13}C NMR spectrum of compound **13**

^{19}F NMR (471 MHz, room temperature, $CDCl_3$)

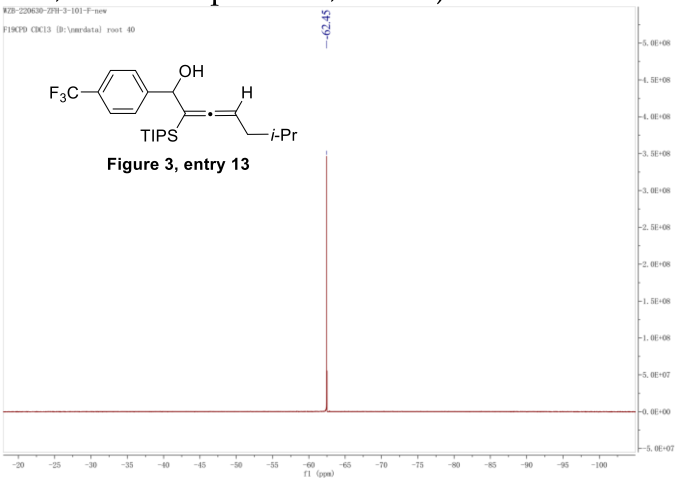

Supplementary Figure 37. ^{19}F NMR spectrum of compound **13**

^{1}H NMR (500 MHz, room temperature, $CDCl_3$)

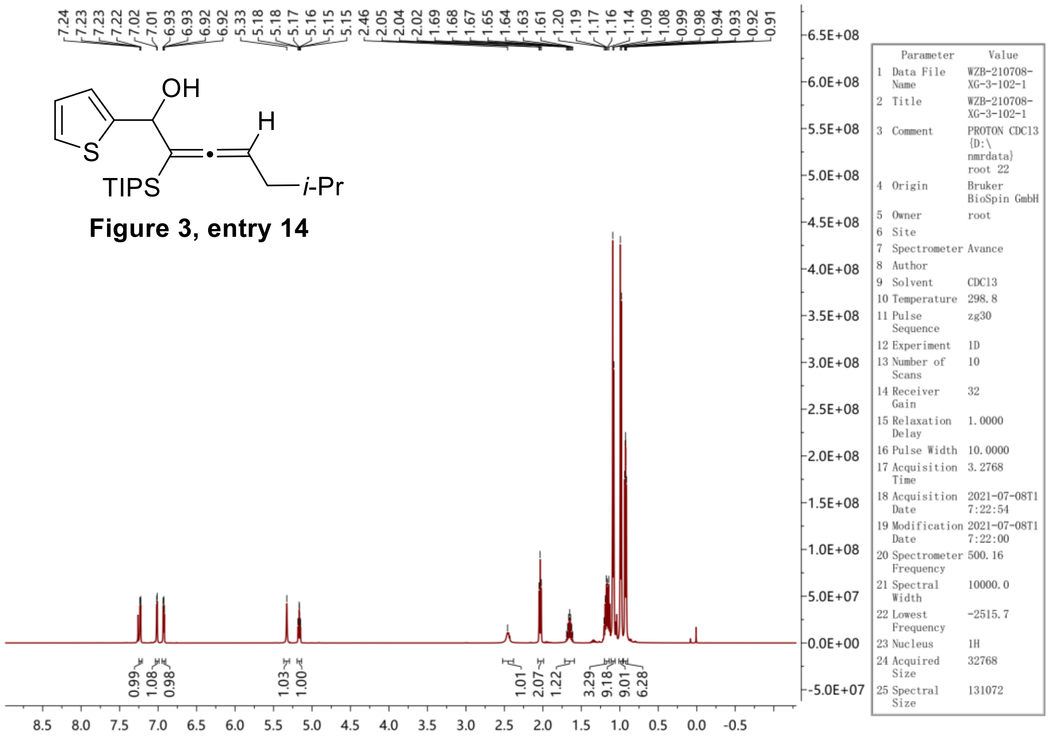

Supplementary Figure 38. ^{1}H NMR spectrum of compound **14**

^{13}C NMR (126 MHz, room temperature, $CDCl_3$)

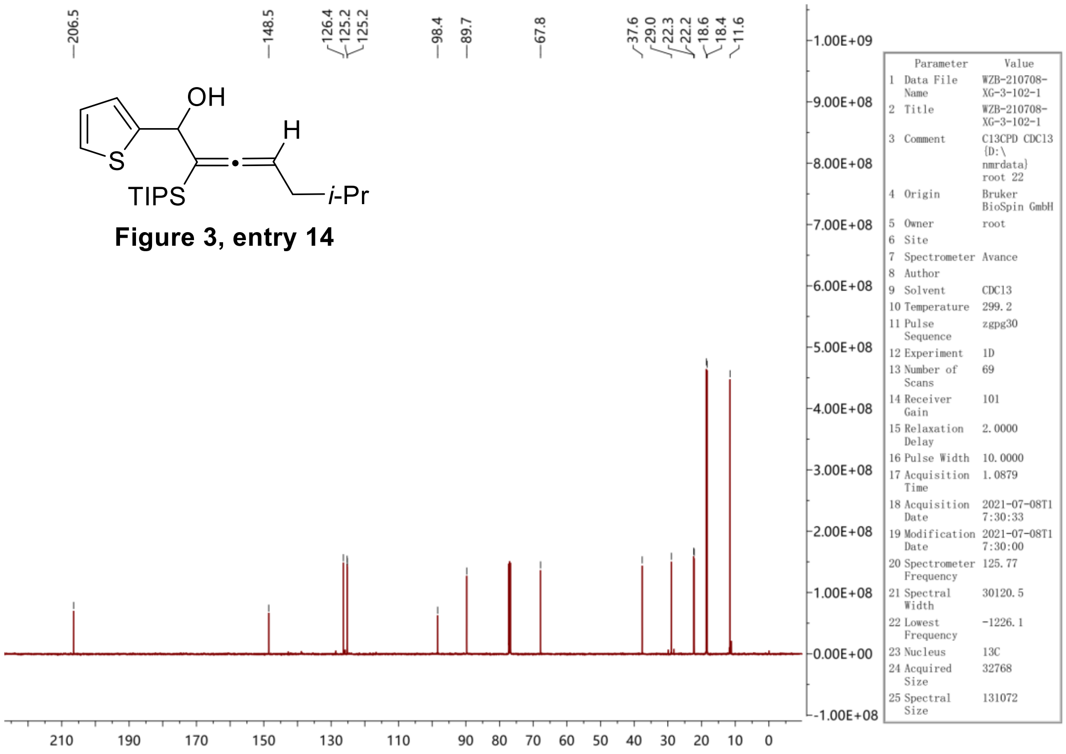

Supplementary Figure 39. ^{13}C NMR spectrum of compound **14**

^{1}H NMR (500 MHz, room temperature, $CDCl_3$)

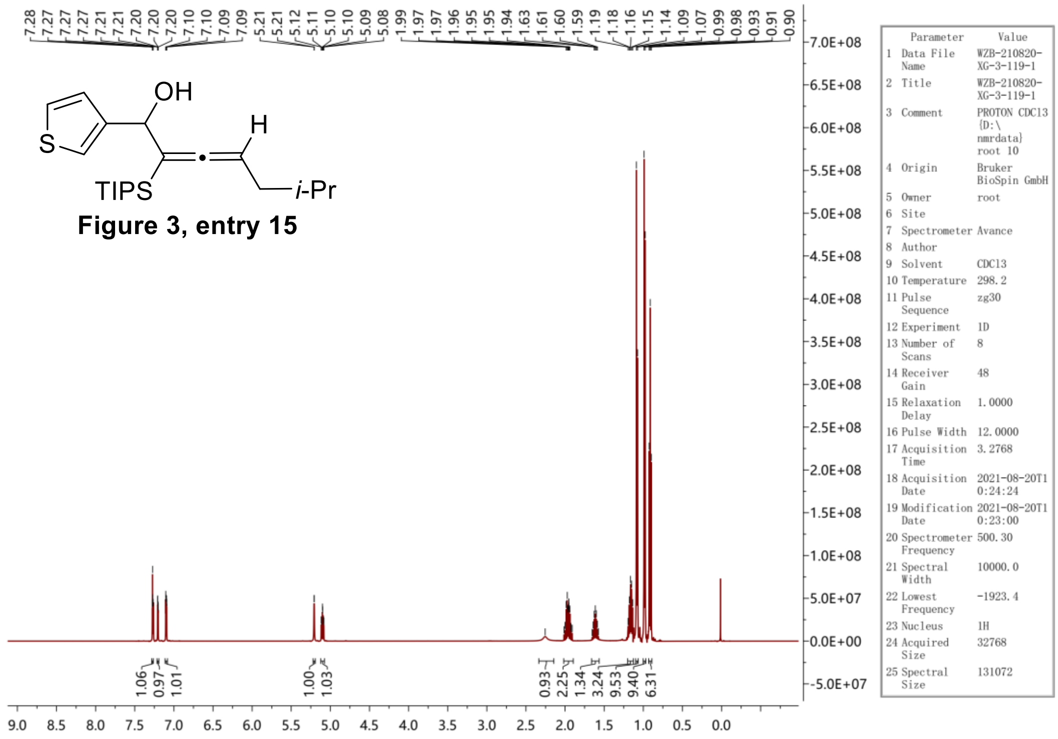

Supplementary Figure 40. ^{1}H NMR spectrum of compound **15**

^{13}C NMR (126 MHz, room temperature, $CDCl_3$)

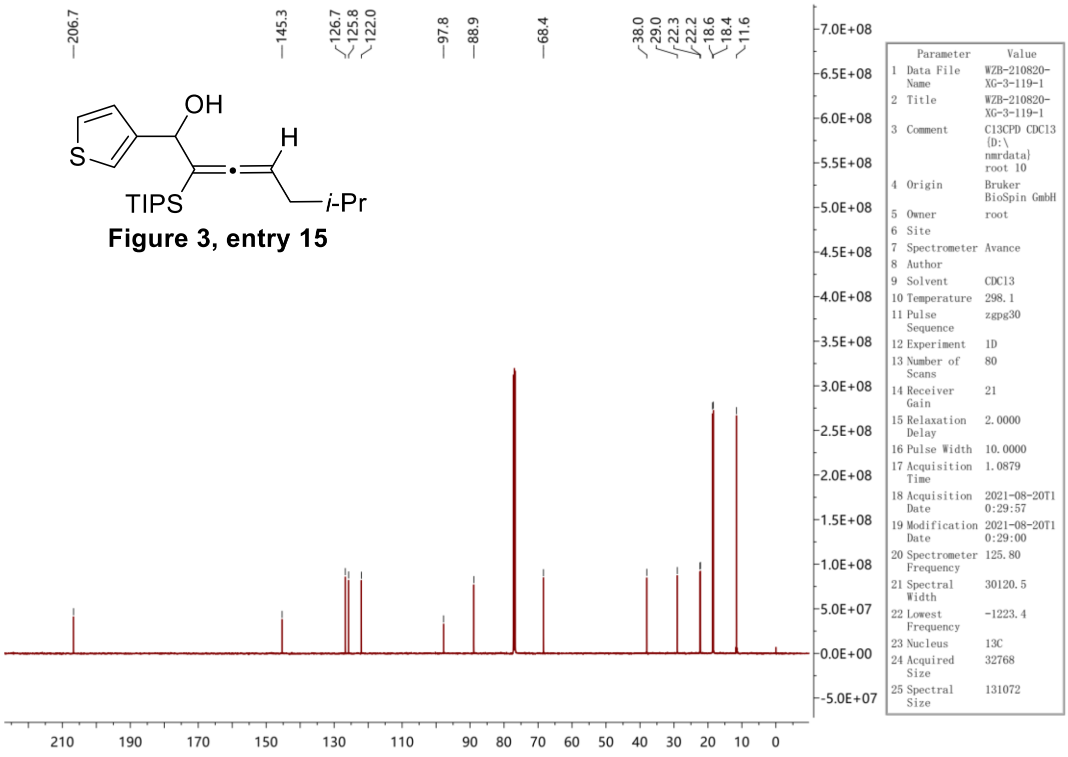

Supplementary Figure 41. ^{13}C NMR spectrum of compound **15**

^{1}H NMR (500 MHz, room temperature, $CDCl_3$)

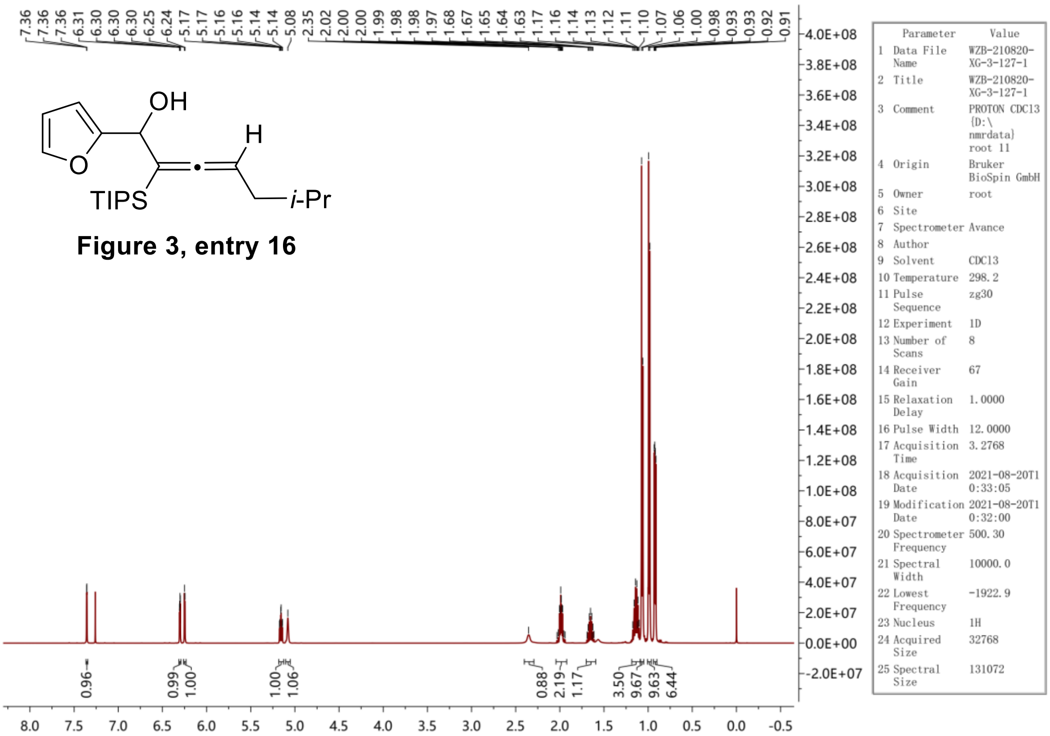

Supplementary Figure 42. ^{1}H NMR spectrum of compound **16**

^{13}C NMR (126 MHz, room temperature, $CDCl_3$)

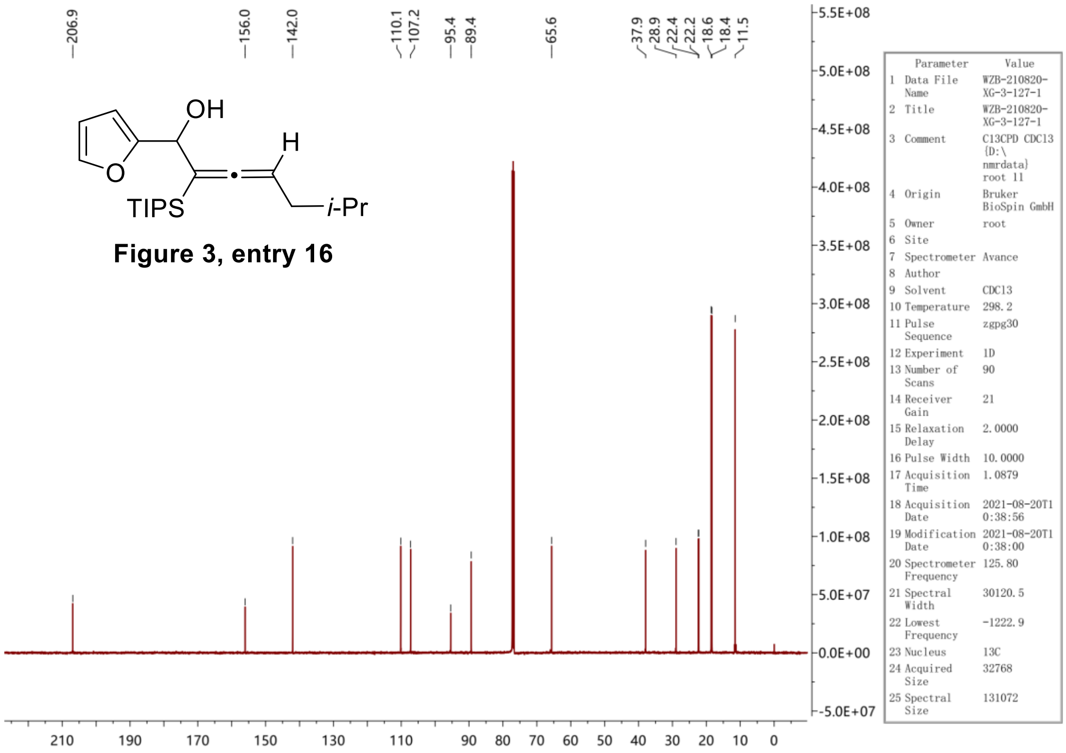

Supplementary Figure 43. ^{13}C NMR spectrum of compound **16**

^{1}H NMR (500 MHz, room temperature, $CDCl_3$)

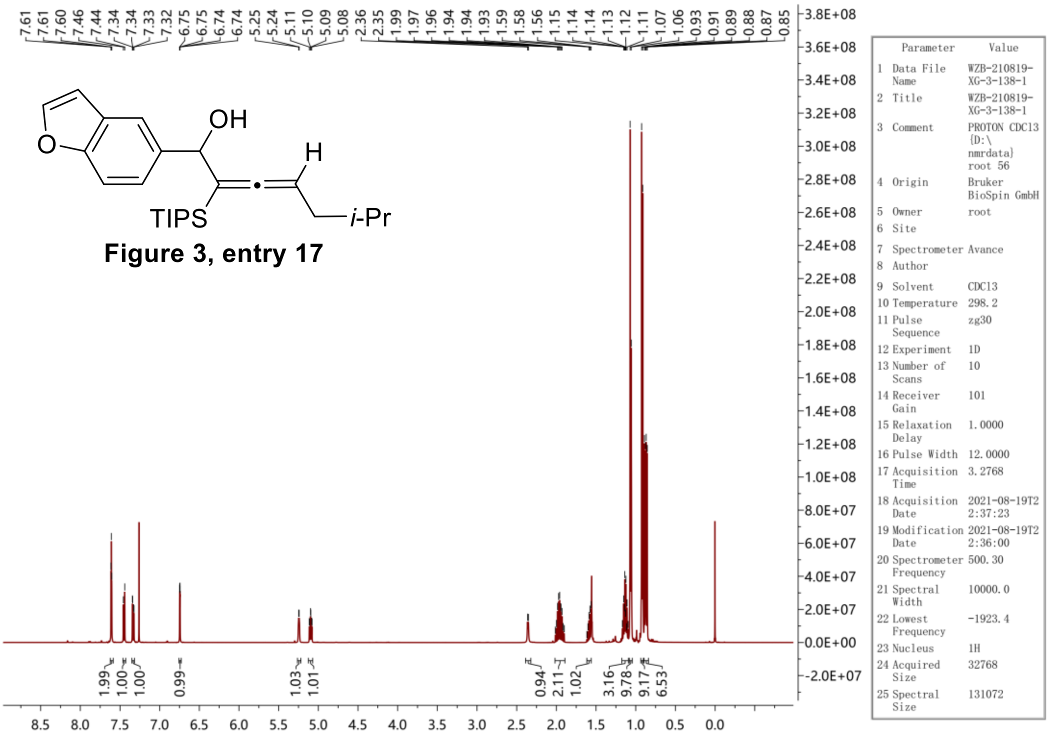

Supplementary Figure 44. ^{1}H NMR spectrum of compound **17**

^{13}C NMR (126 MHz, room temperature, $CDCl_3$)

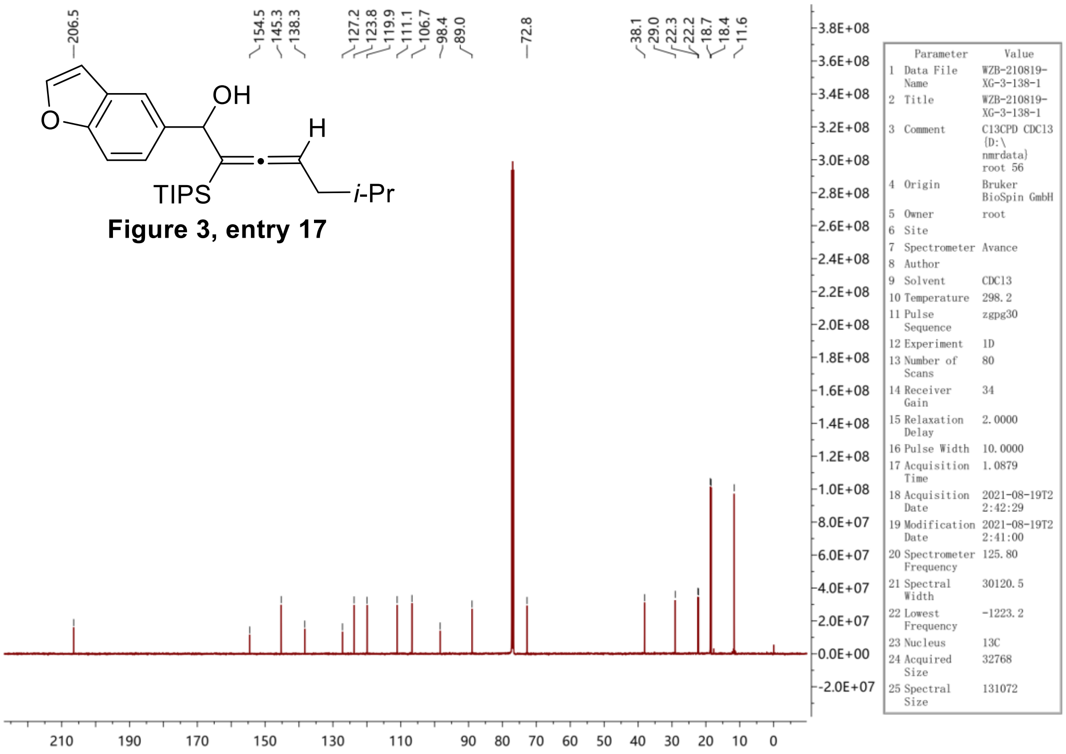

Supplementary Figure 45. ^{13}C NMR spectrum of compound **17**

^{1}H NMR (500 MHz, room temperature, $CDCl_3$)

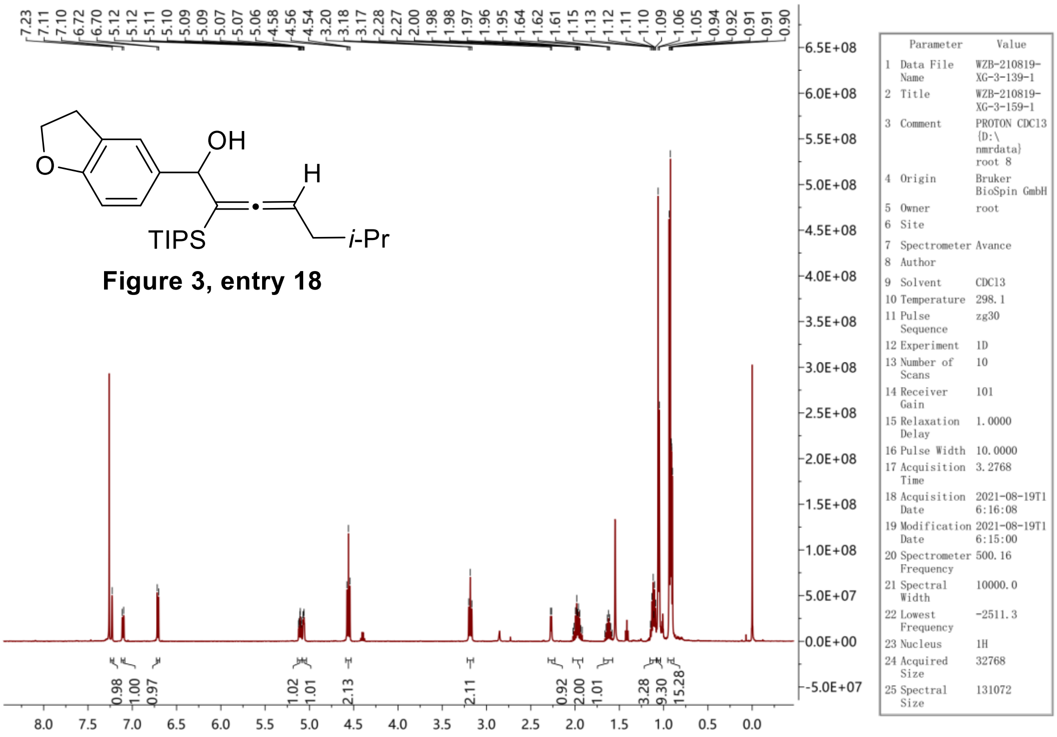

Supplementary Figure 46. ^{1}H NMR spectrum of compound **18**

^{13}C NMR (126 MHz, room temperature, $CDCl_3$)

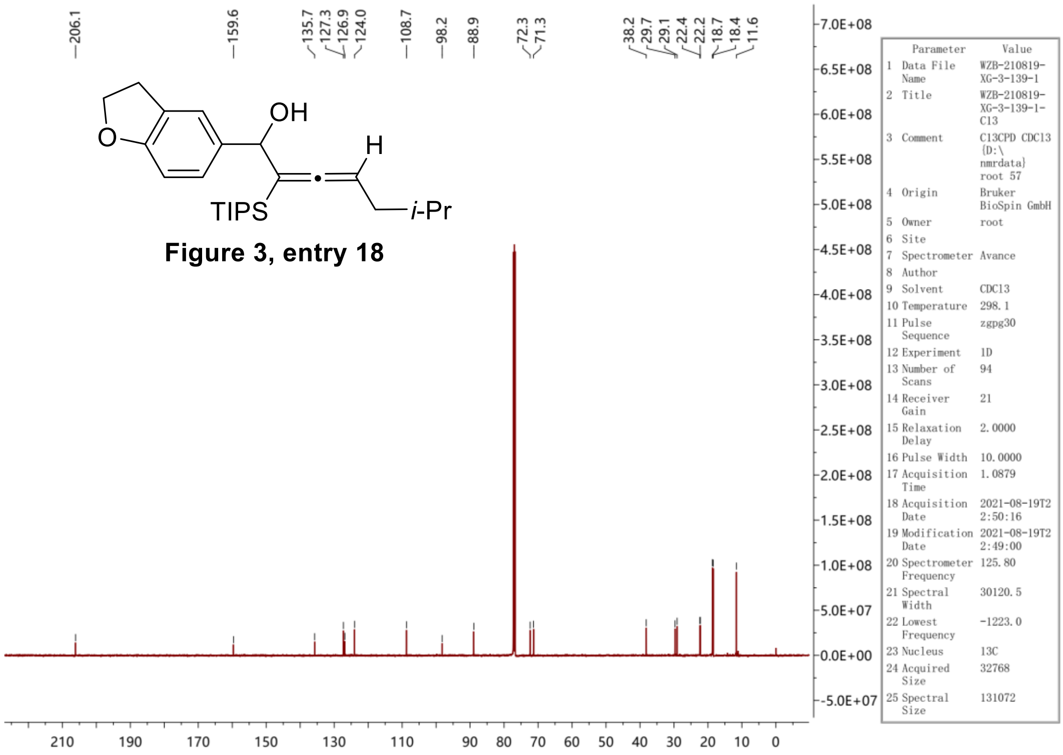

Supplementary Figure 47. ^{13}C NMR spectrum of compound **18**

^{1}H NMR (500 MHz, room temperature, $CDCl_3$)

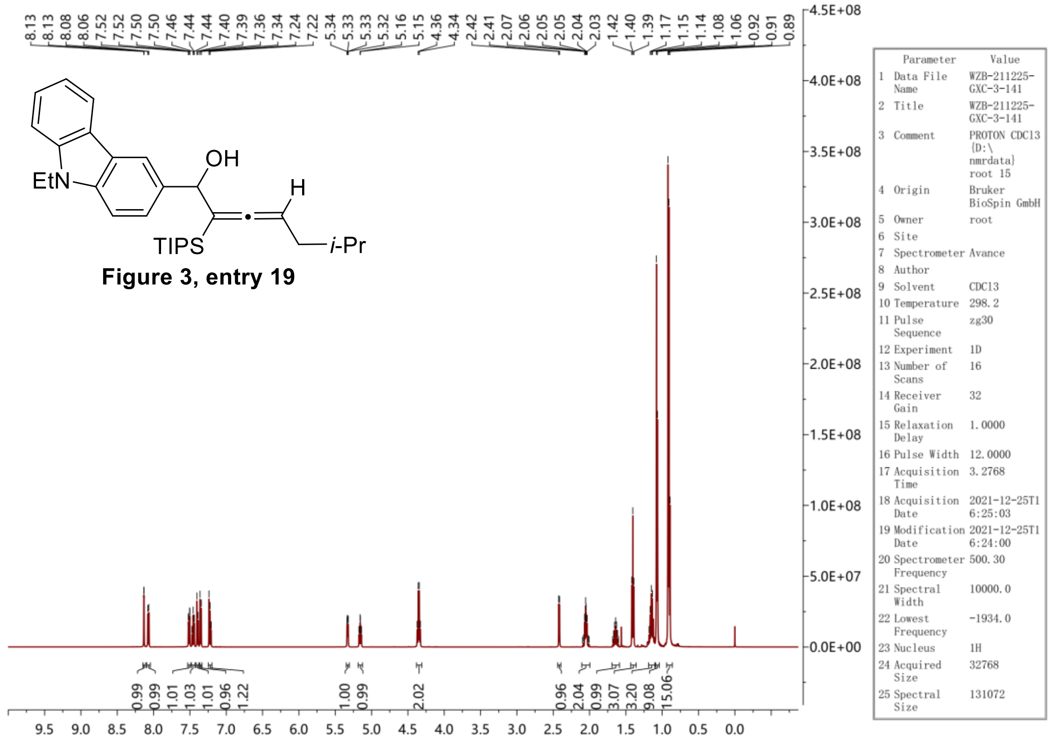

Supplementary Figure 48. ^{1}H NMR spectrum of compound **19**

^{13}C NMR (126 MHz, room temperature, $CDCl_3$)

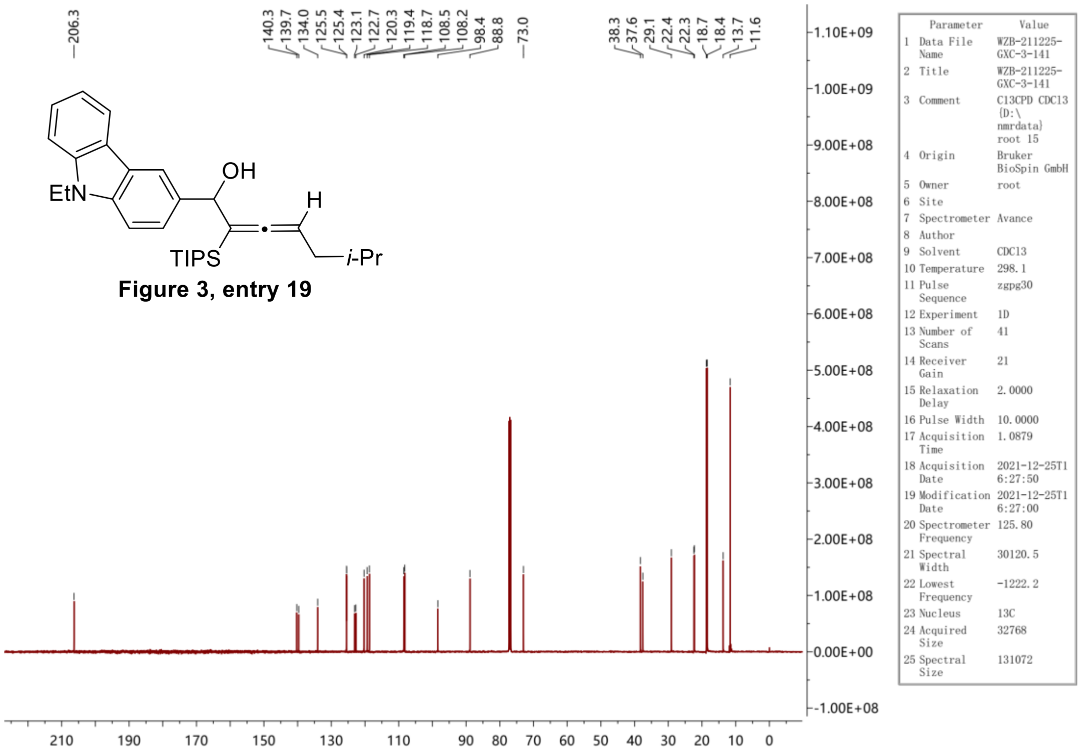

Supplementary Figure 49. ^{13}C NMR spectrum of compound **19**

^{1}H NMR (500 MHz, room temperature, $CDCl_3$)

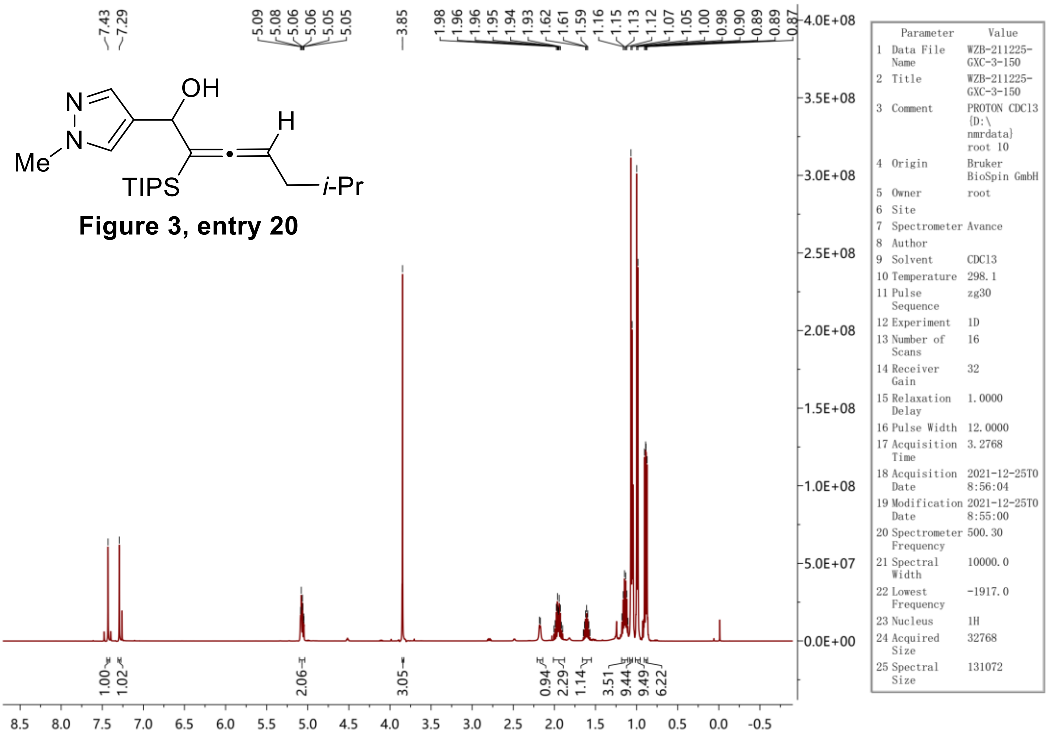

Supplementary Figure 50. ^{1}H NMR spectrum of compound **20**

^{13}C NMR (126 MHz, room temperature, $CDCl_3$)

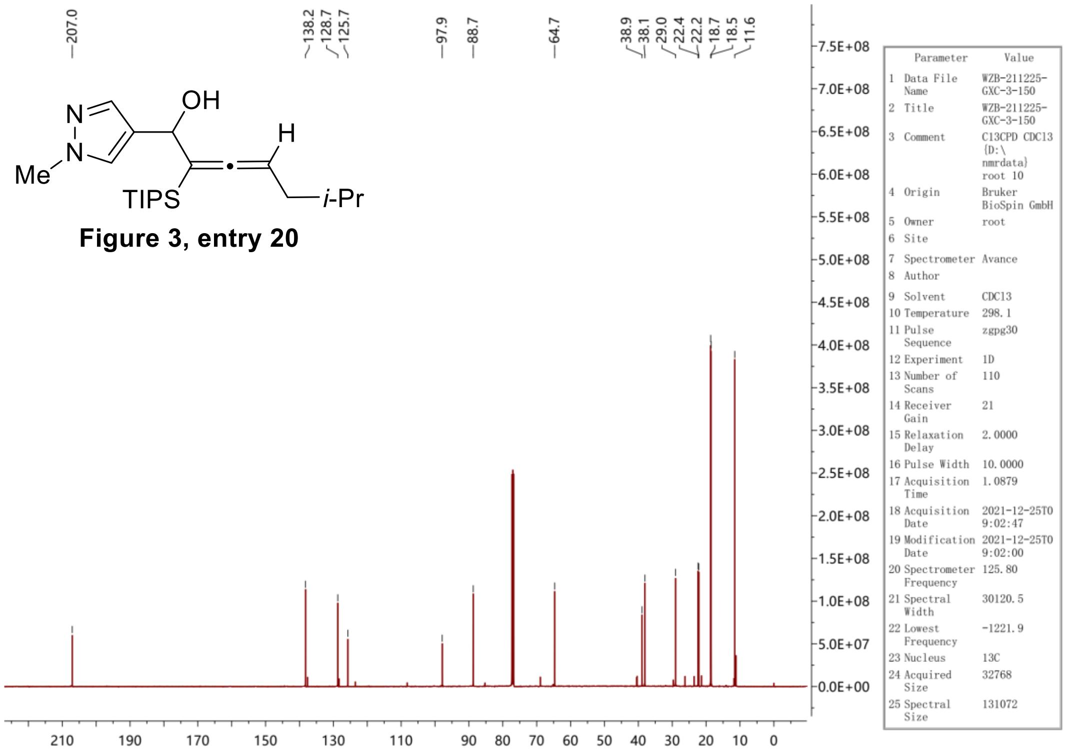

Supplementary Figure 51. ^{13}C NMR spectrum of compound **20**

^{1}H NMR (500 MHz, room temperature, $CDCl_3$)

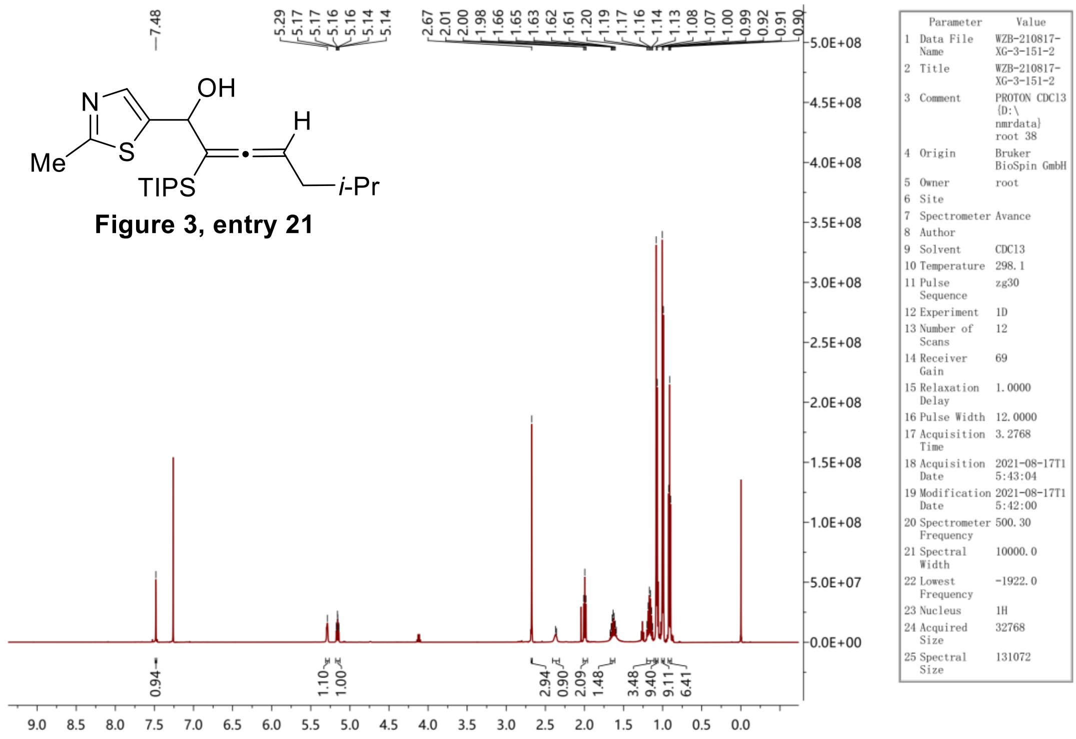

Supplementary Figure 52. ^{1}H NMR spectrum of compound **21**

^{13}C NMR (126 MHz, room temperature, $CDCl_3$)

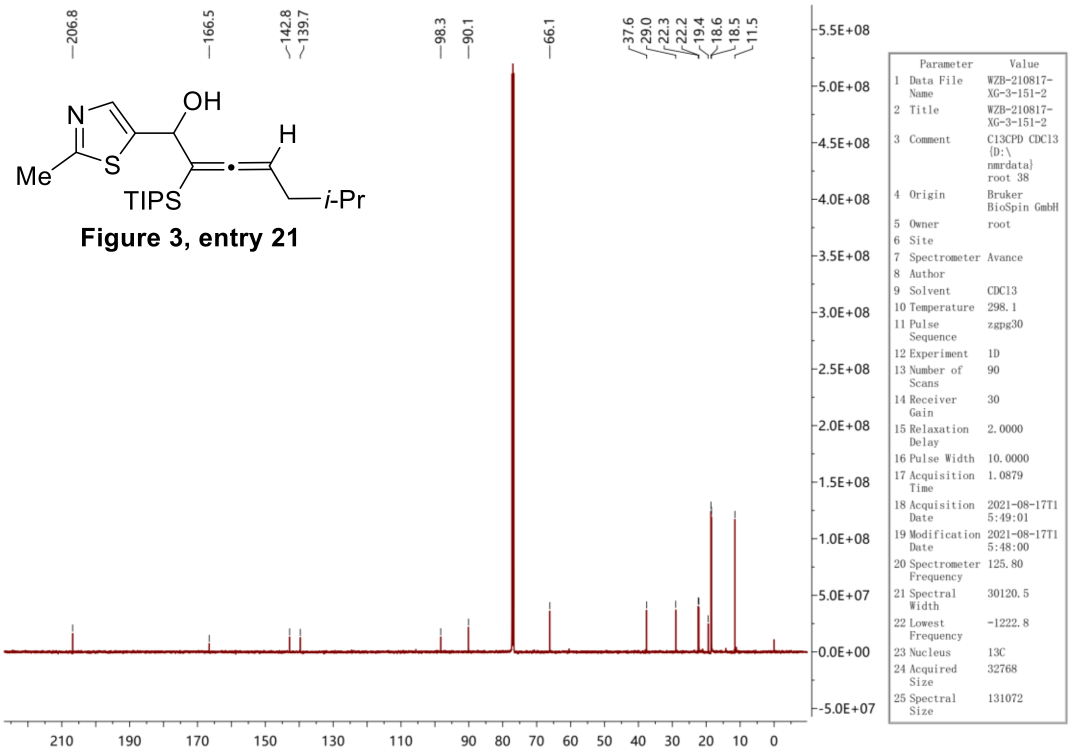

Supplementary Figure 53. ^{13}C NMR spectrum of compound **21**

^{1}H NMR (500 MHz, room temperature, $CDCl_3$)

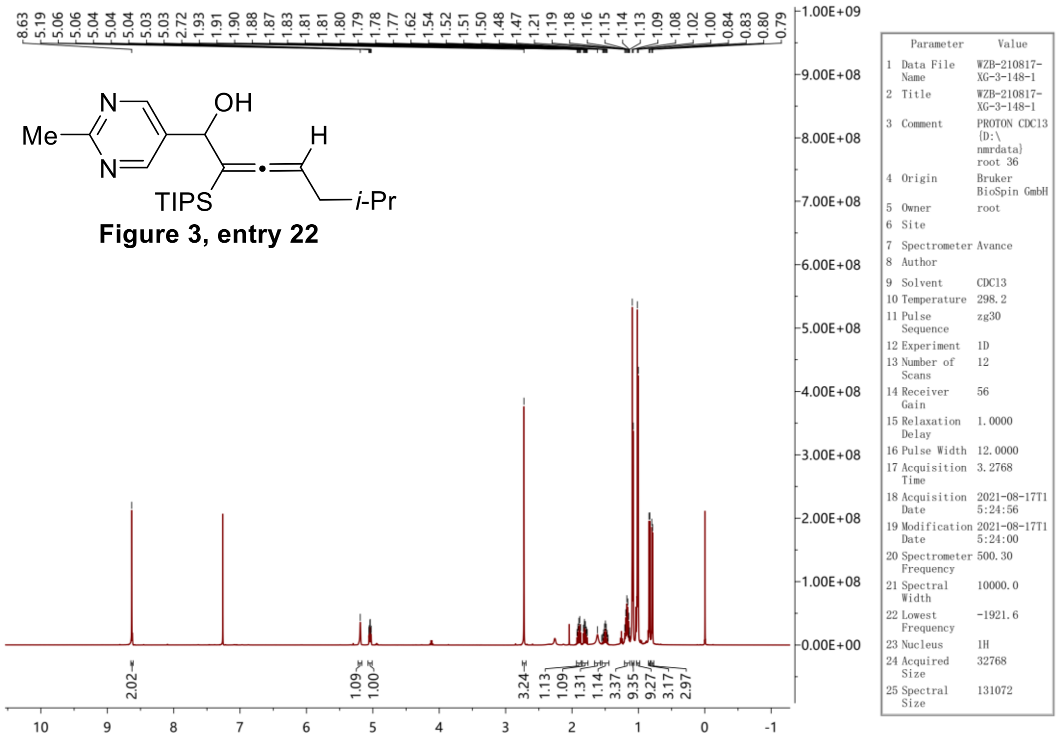

Supplementary Figure 54. ^{1}H NMR spectrum of compound **22**

^{13}C NMR (126 MHz, room temperature, $CDCl_3$)

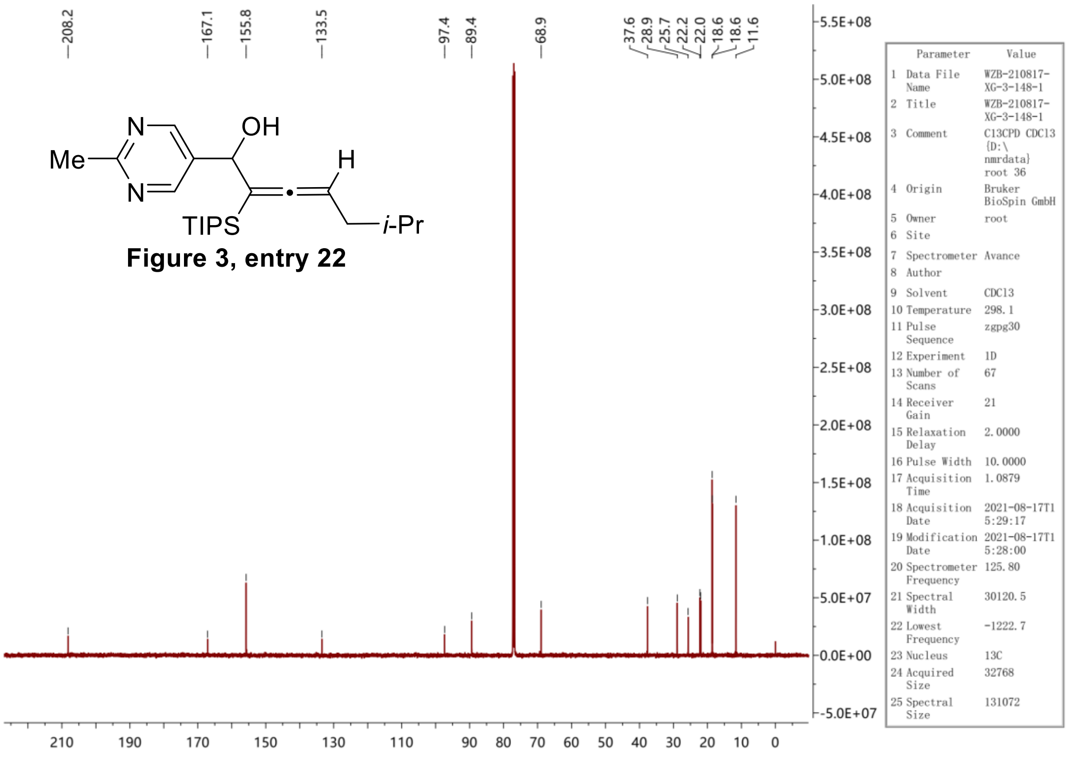

Supplementary Figure 55. ^{13}C NMR spectrum of compound **22**

^{1}H NMR (500 MHz, room temperature, $CDCl_3$)

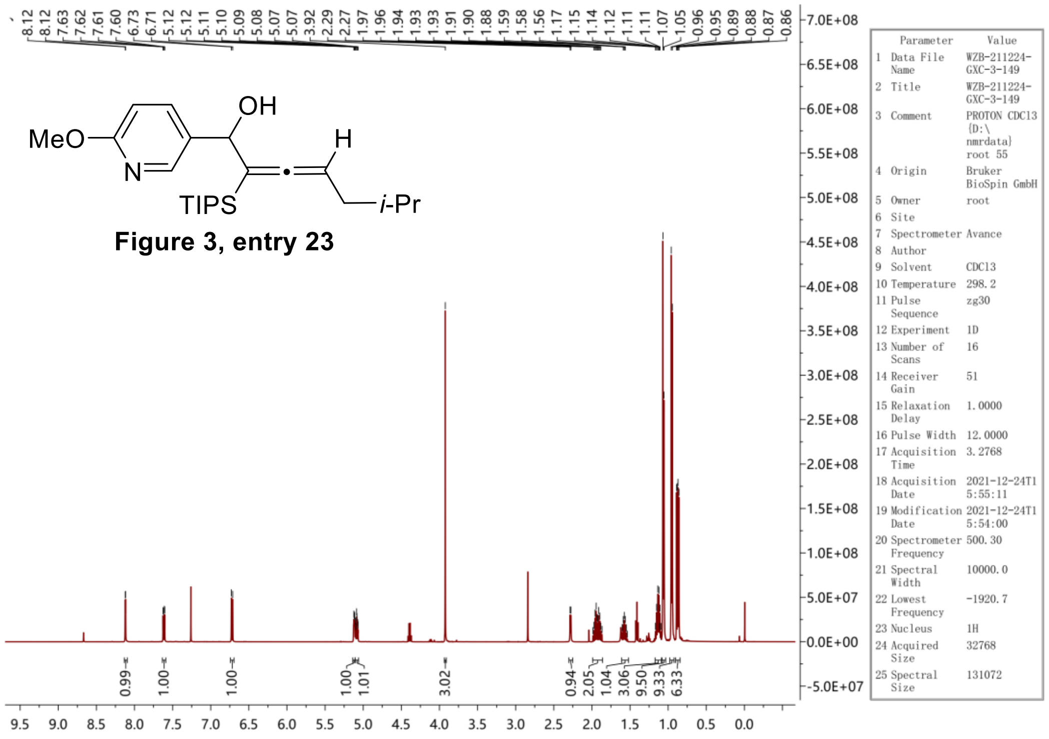

Supplementary Figure 56. ^{1}H NMR spectrum of compound **23**

^{13}C NMR (126 MHz, room temperature, $CDCl_3$)

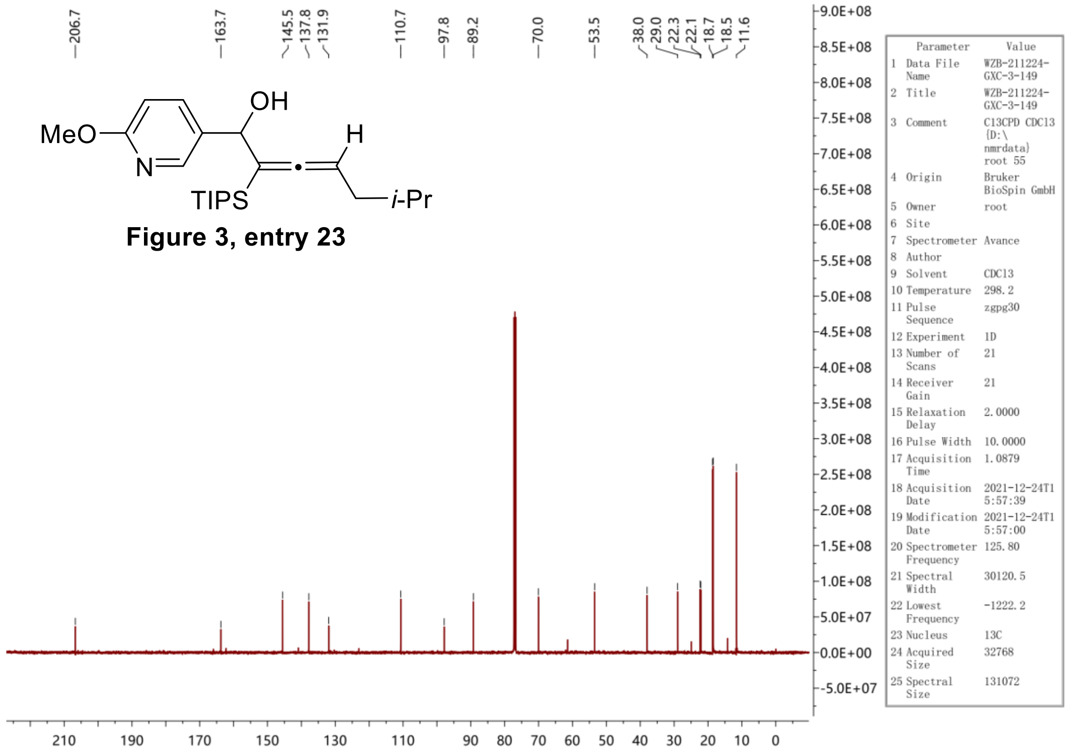

Supplementary Figure 57. ^{13}C NMR spectrum of compound **23**

^{1}H NMR (500 MHz, room temperature, $CDCl_3$)

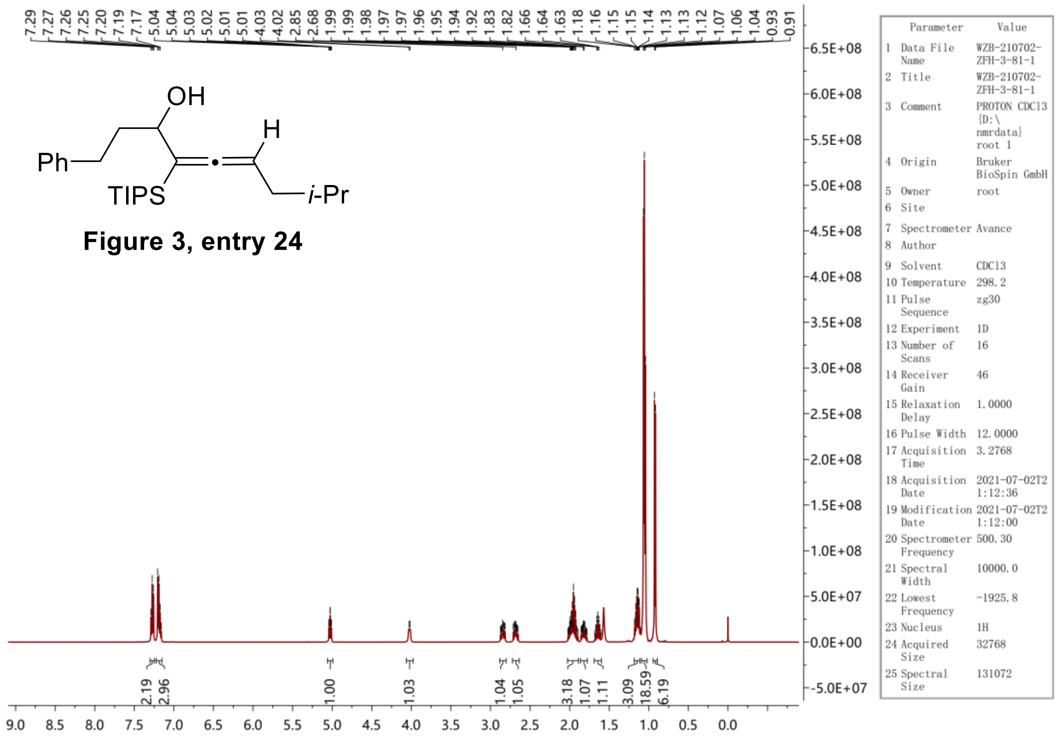

Supplementary Figure 58. ^{1}H NMR spectrum of compound **24**

^{13}C NMR (126 MHz, room temperature, $CDCl_3$)

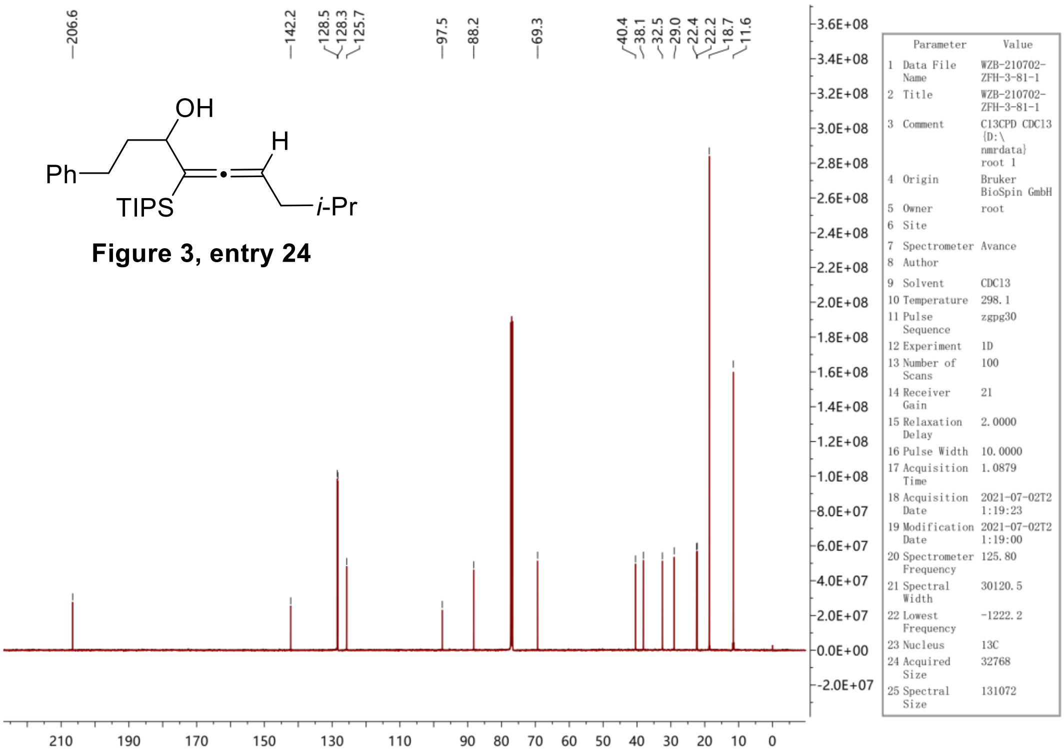

Supplementary Figure 59. ^{13}C NMR spectrum of compound **24**

^1H NMR (500 MHz, room temperature, $CDCl_3$)

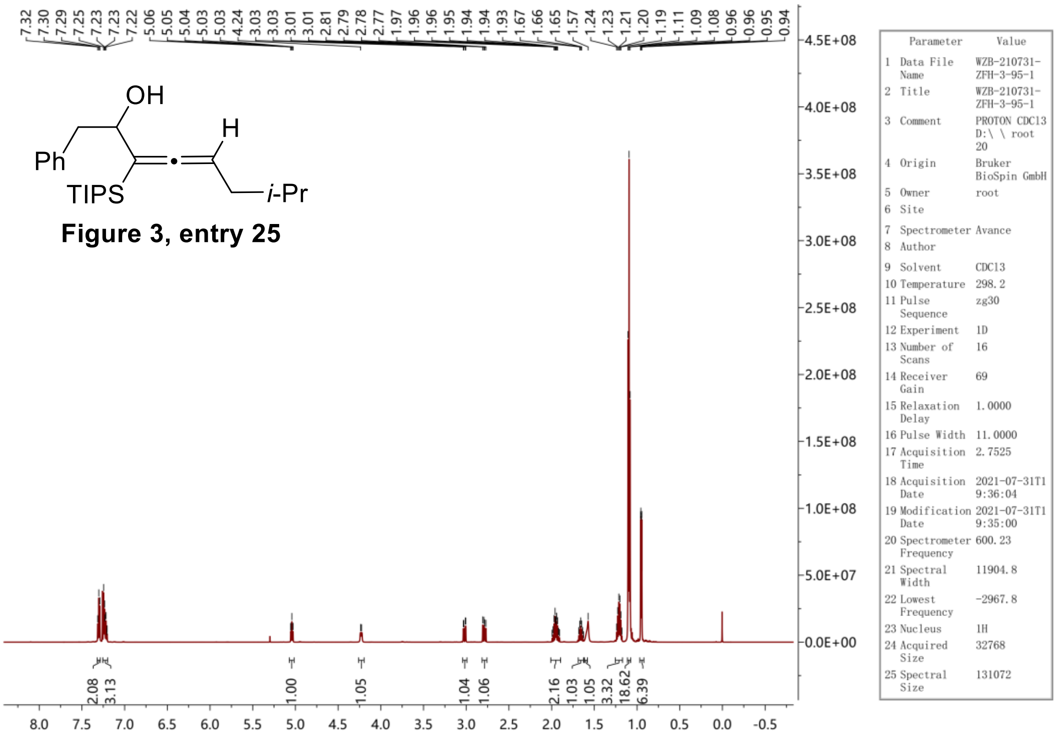

Supplementary Figure 60. ^1H NMR spectrum of compound **25**

^{13}C NMR (126 MHz, room temperature, $CDCl_3$)

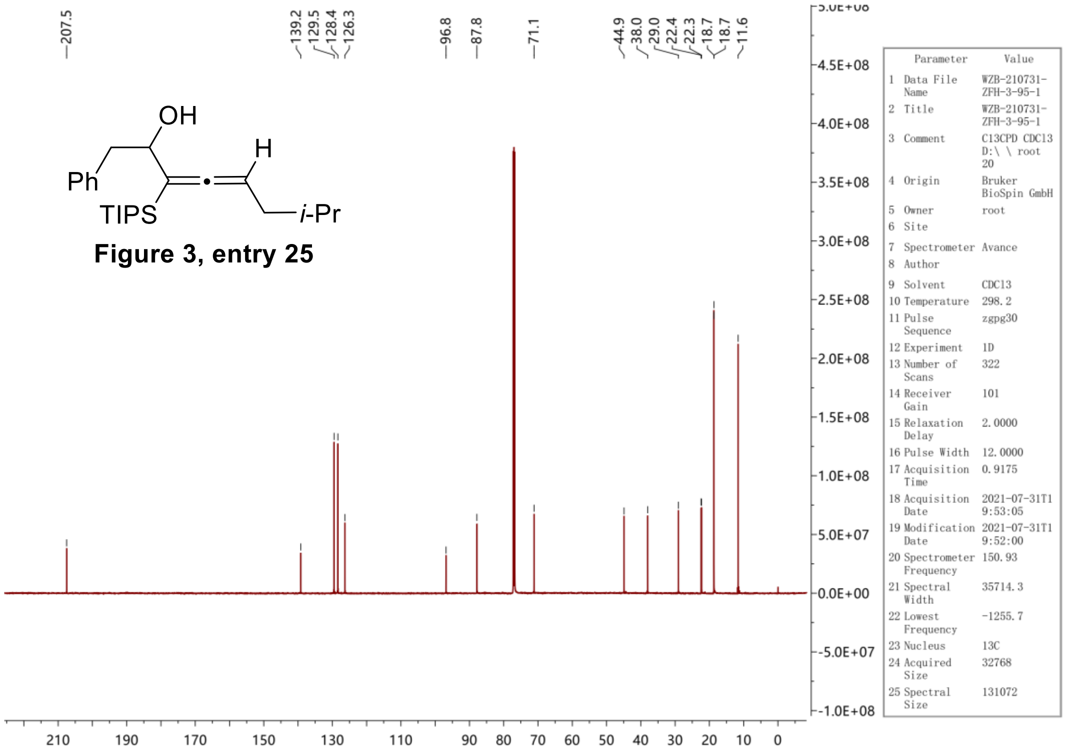

Supplementary Figure 61. ^{13}C NMR spectrum of compound **25**

^{1}H NMR (500 MHz, room temperature, $CDCl_3$)

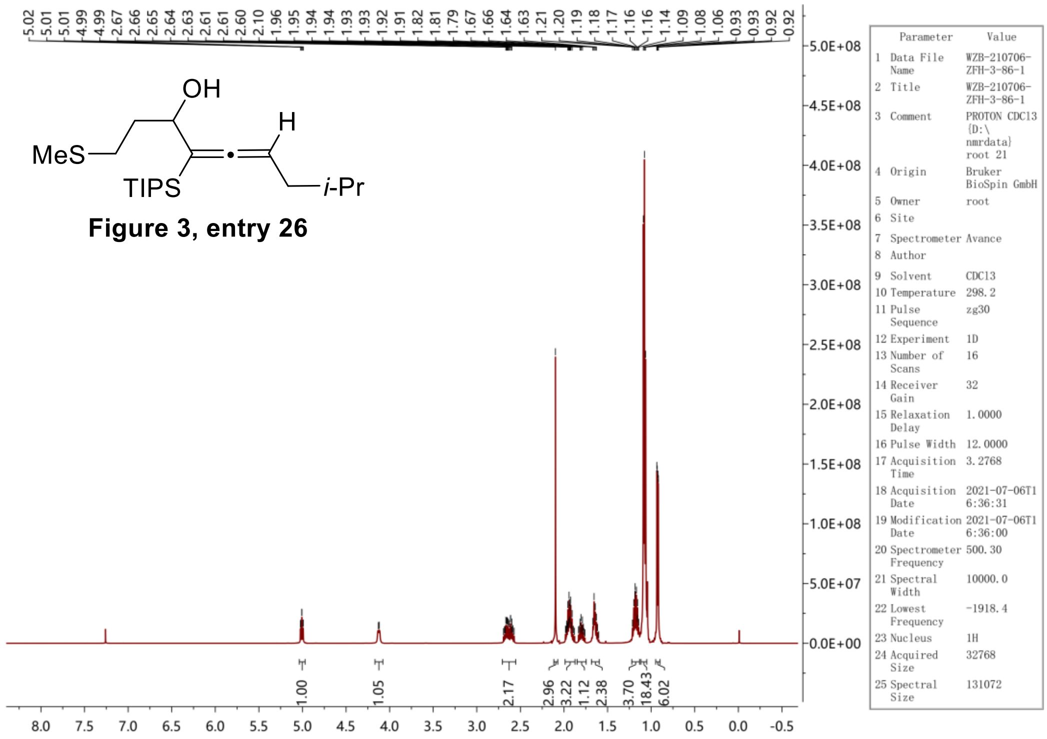

Supplementary Figure 62. ^{1}H NMR spectrum of compound **26**

^{13}C NMR (126 MHz, room temperature, $CDCl_3$)

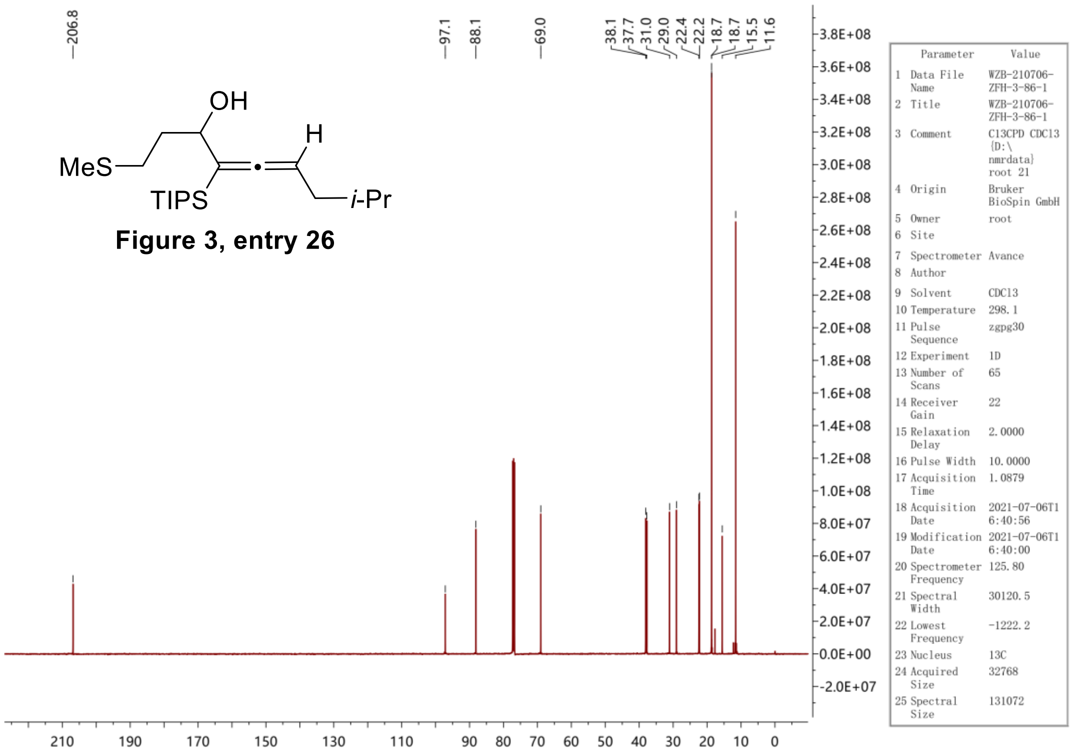

Supplementary Figure 63. ^{13}C NMR spectrum of compound **26**

^1H NMR (500 MHz, room temperature, $CDCl_3$)

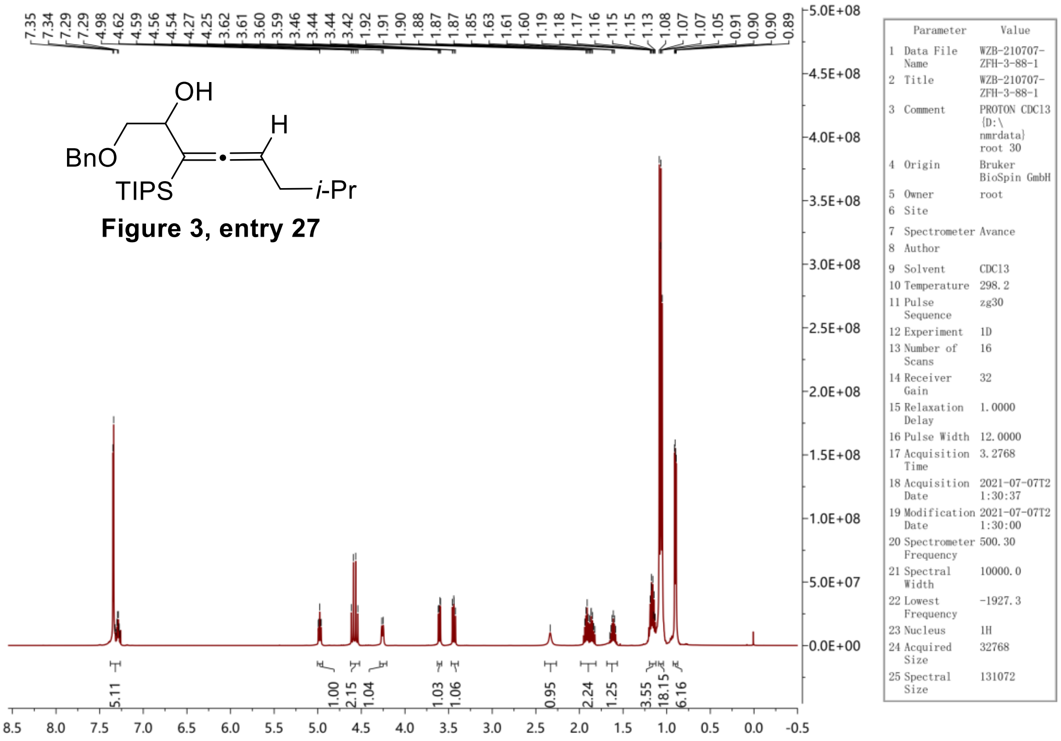

Supplementary Figure 64. ^1H NMR spectrum of compound **27**

^{13}C NMR (126 MHz, room temperature, $CDCl_3$)

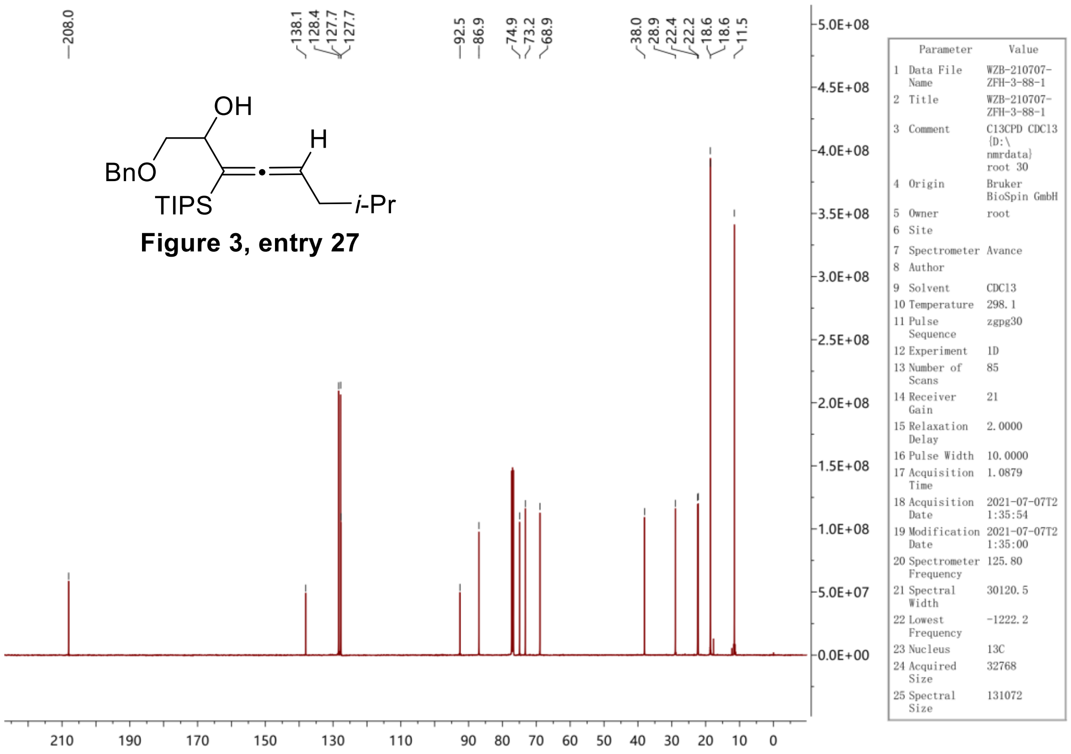

Supplementary Figure 65. ^{13}C NMR spectrum of compound **27**

^{1}H NMR (500 MHz, room temperature, $CDCl_3$)

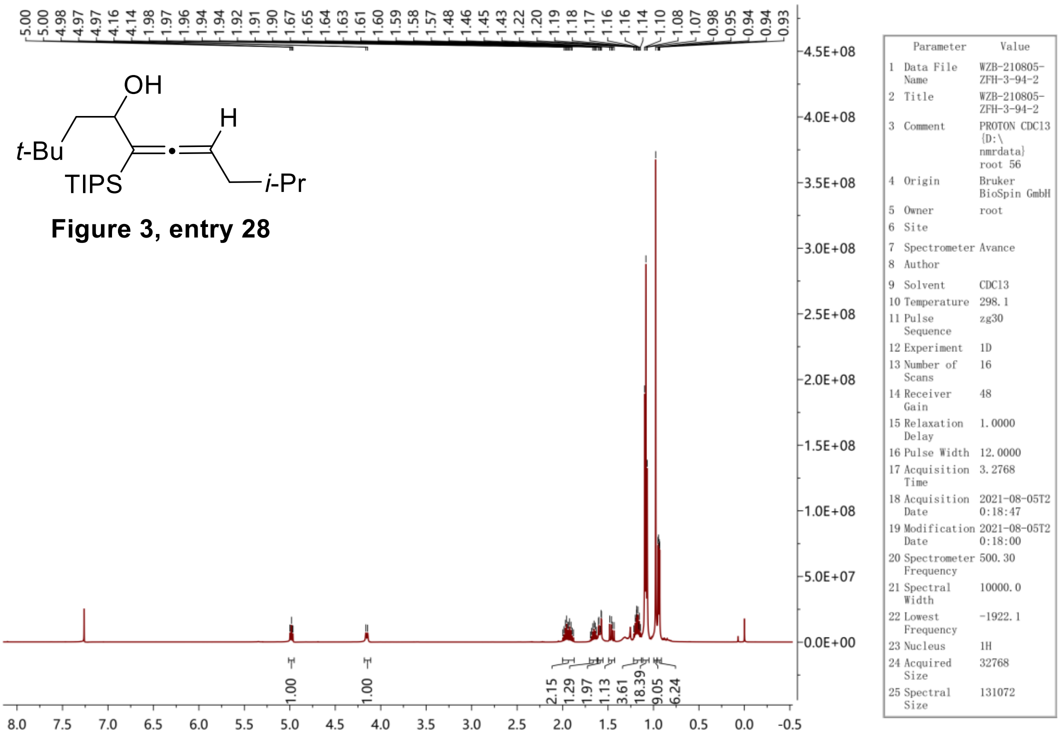

Supplementary Figure 66. ^{1}H NMR spectrum of compound **28**

^{13}C NMR (126 MHz, room temperature, $CDCl_3$)

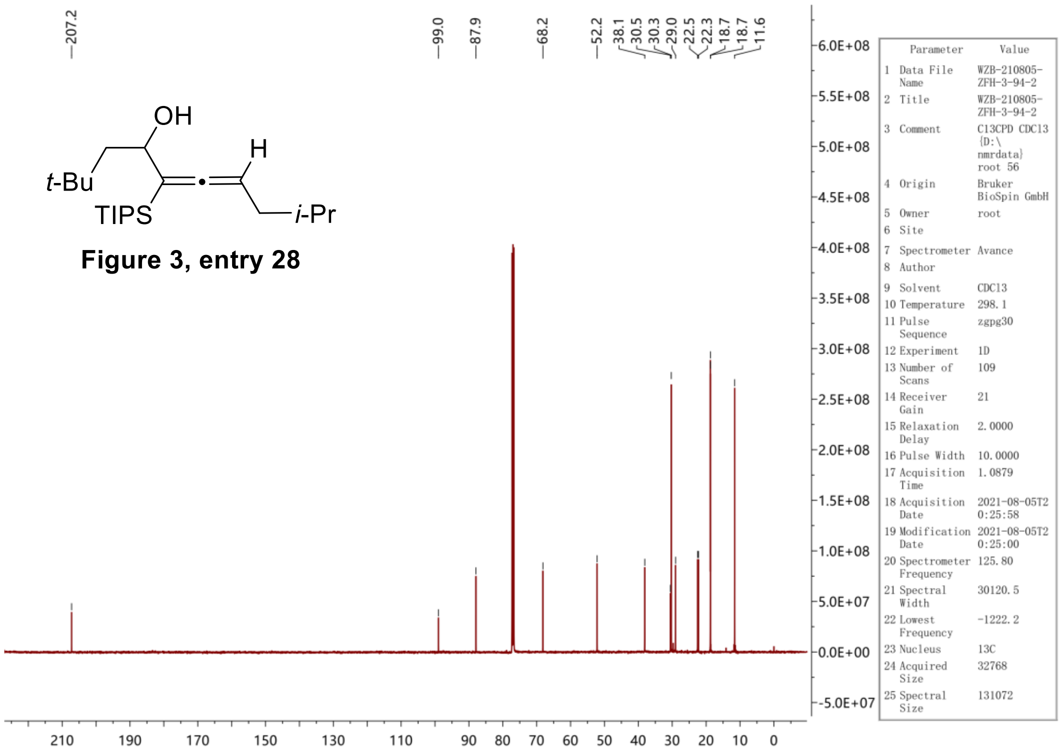

Supplementary Figure 67. ^{13}C NMR spectrum of compound **28**

^{1}H NMR (500 MHz, room temperature, $CDCl_3$)

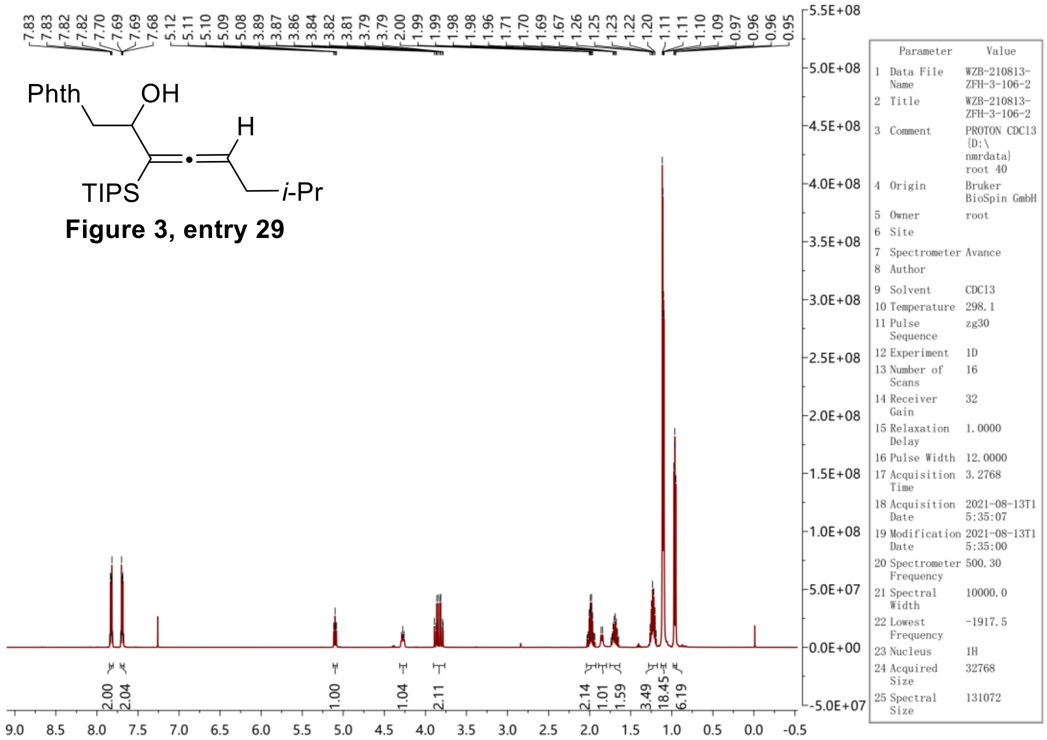

Supplementary Figure 68. ^{1}H NMR spectrum of compound **29**

^{13}C NMR (126 MHz, room temperature, $CDCl_3$)

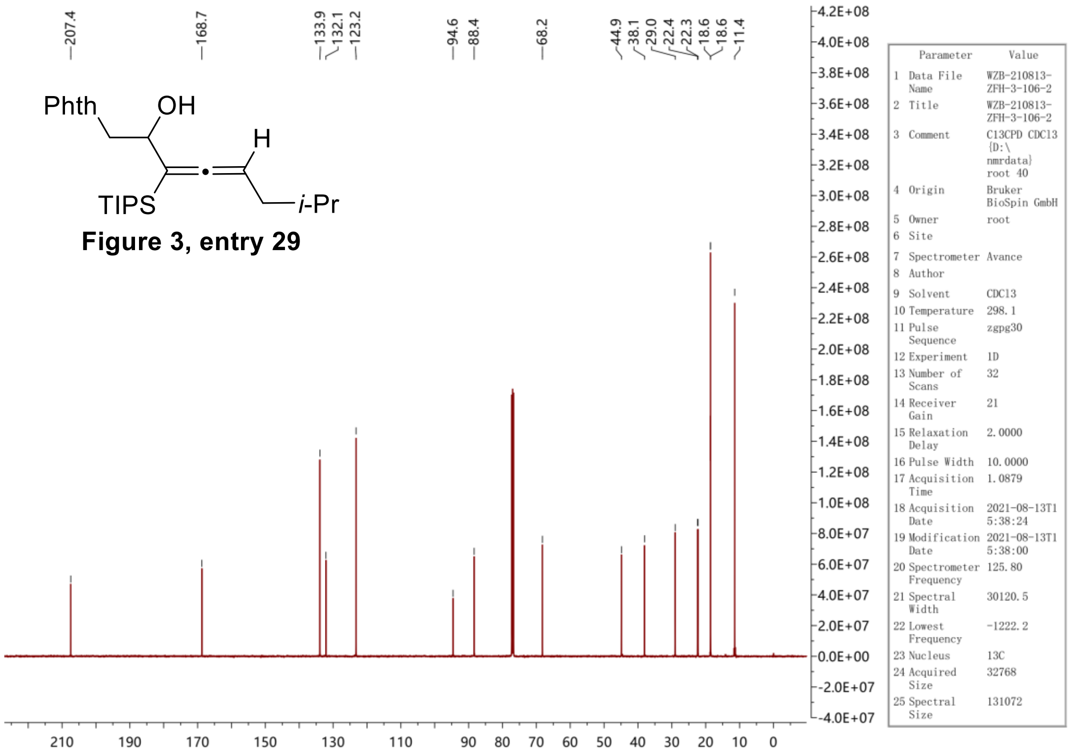

Supplementary Figure 69. ^{13}C NMR spectrum of compound **29**

^{1}H NMR (500 MHz, room temperature, $CDCl_3$)

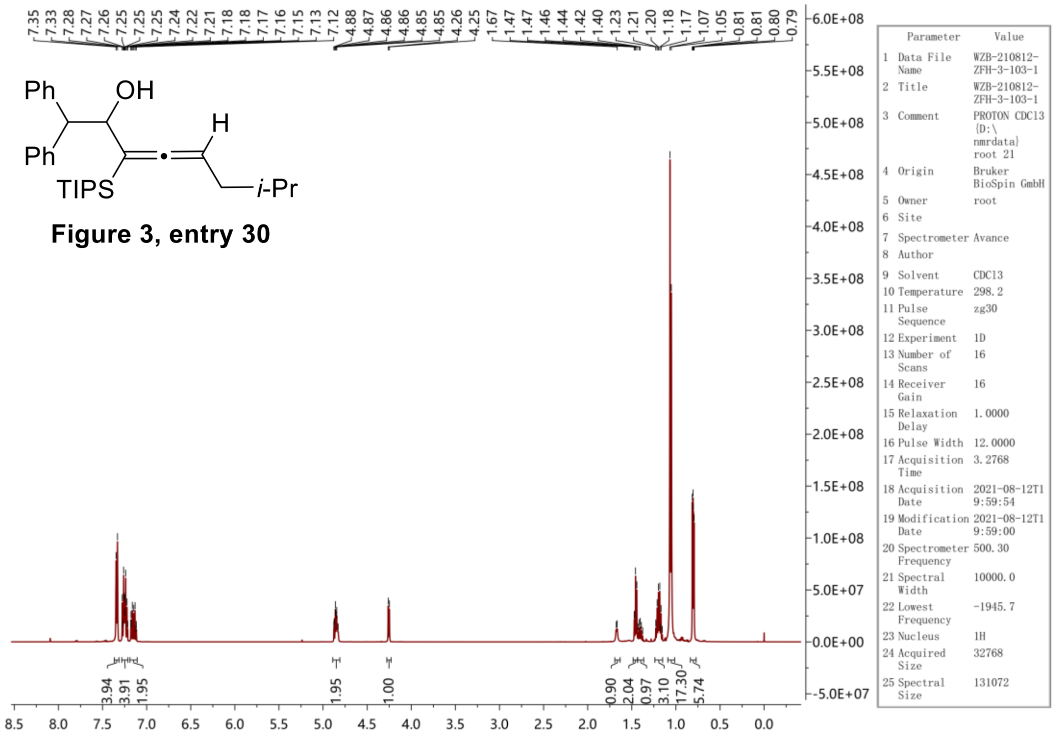

Supplementary Figure 70. ^{1}H NMR spectrum of compound **30**

^{13}C NMR (126 MHz, room temperature, $CDCl_3$)

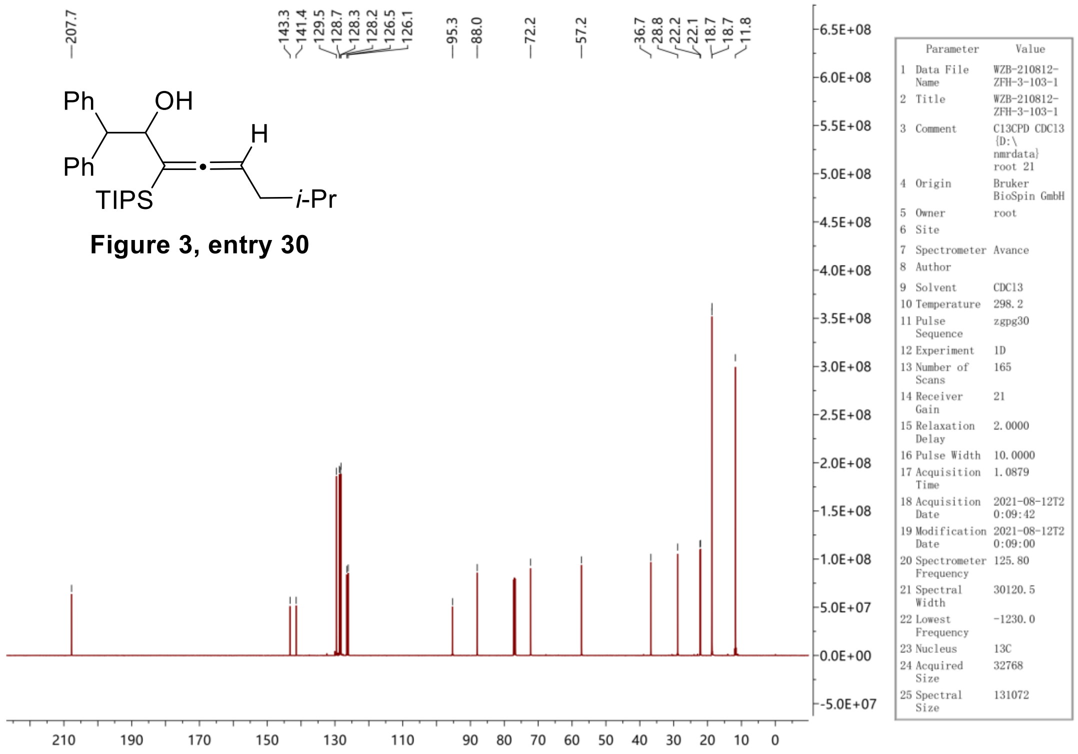

Supplementary Figure 71. ^{13}C NMR spectrum of compound **30**

^1H NMR (500 MHz, room temperature, $CDCl_3$)

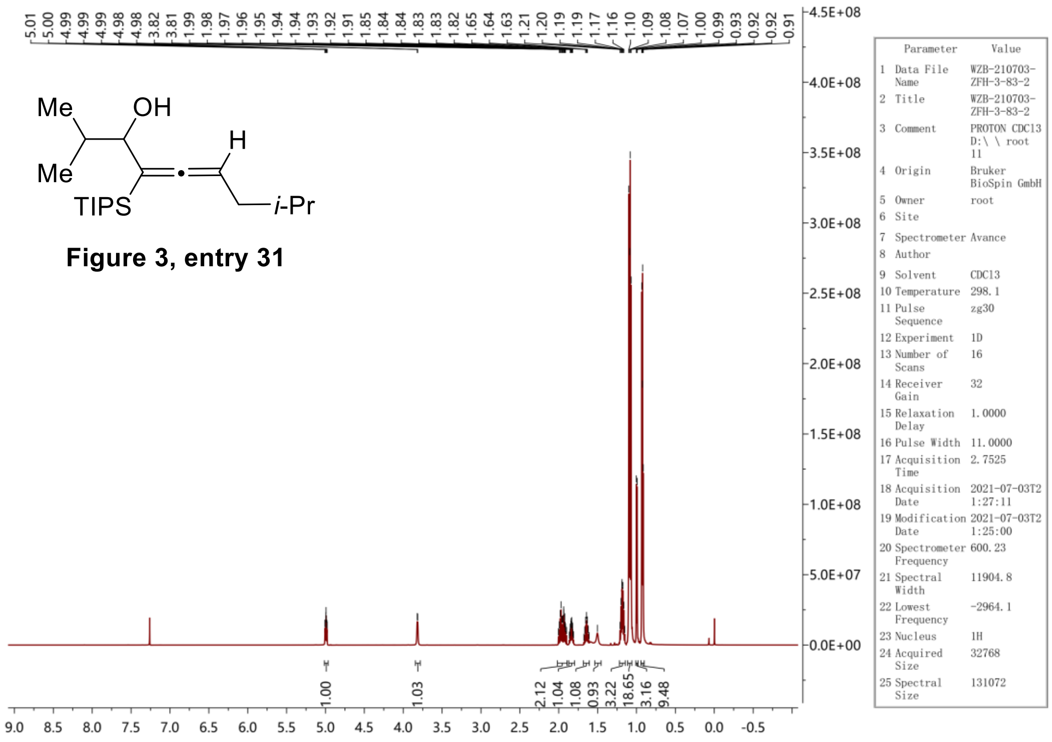

Supplementary Figure 72. ^1H NMR spectrum of compound **31**

^{13}C NMR (126 MHz, room temperature, $CDCl_3$)

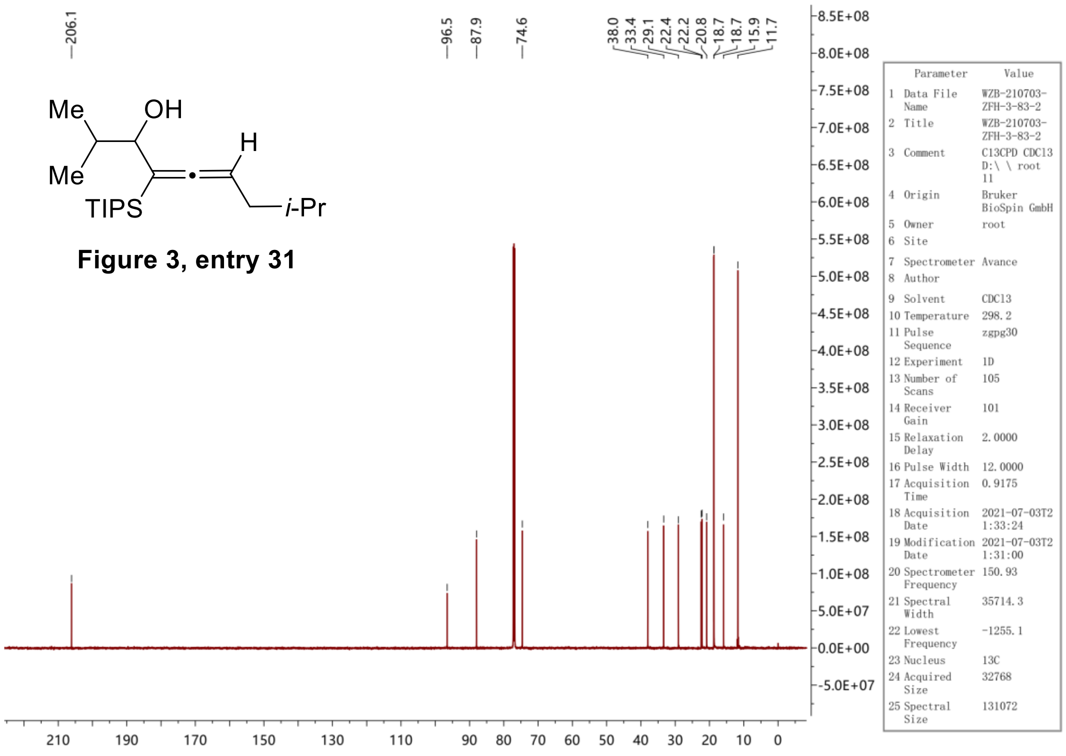

Supplementary Figure 73. ^{13}C NMR spectrum of compound **31**

^{1}H NMR (500 MHz, room temperature, $CDCl_3$)

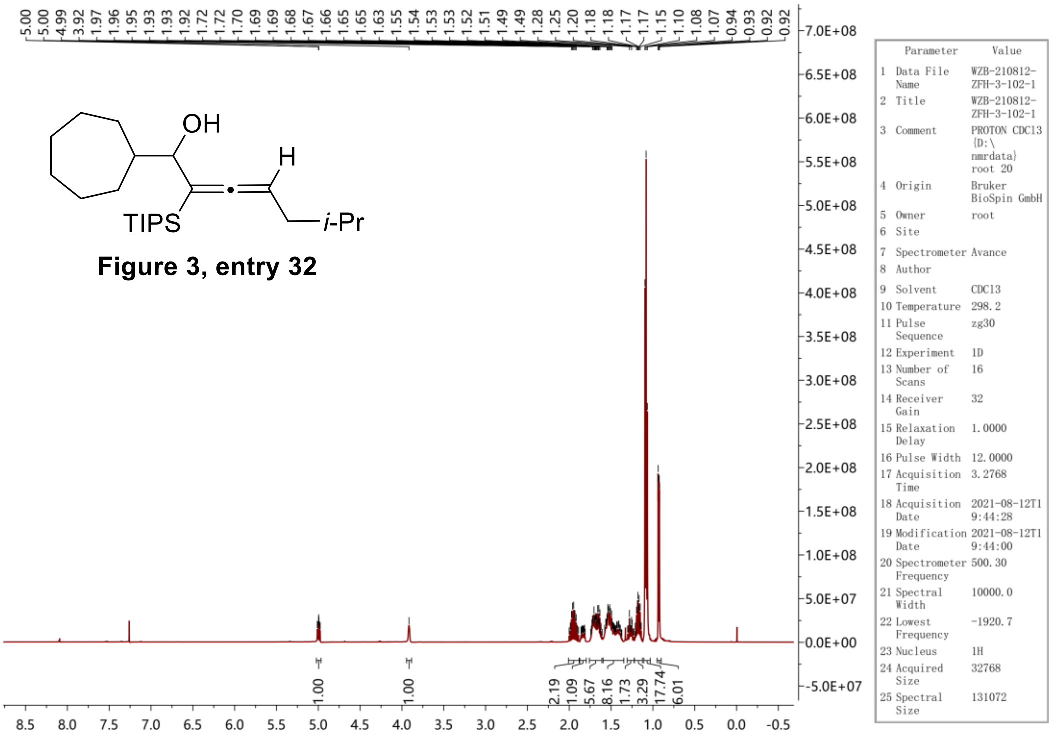

Supplementary Figure 74. ^{1}H NMR spectrum of compound **32**

^{13}C NMR (126 MHz, room temperature, $CDCl_3$)

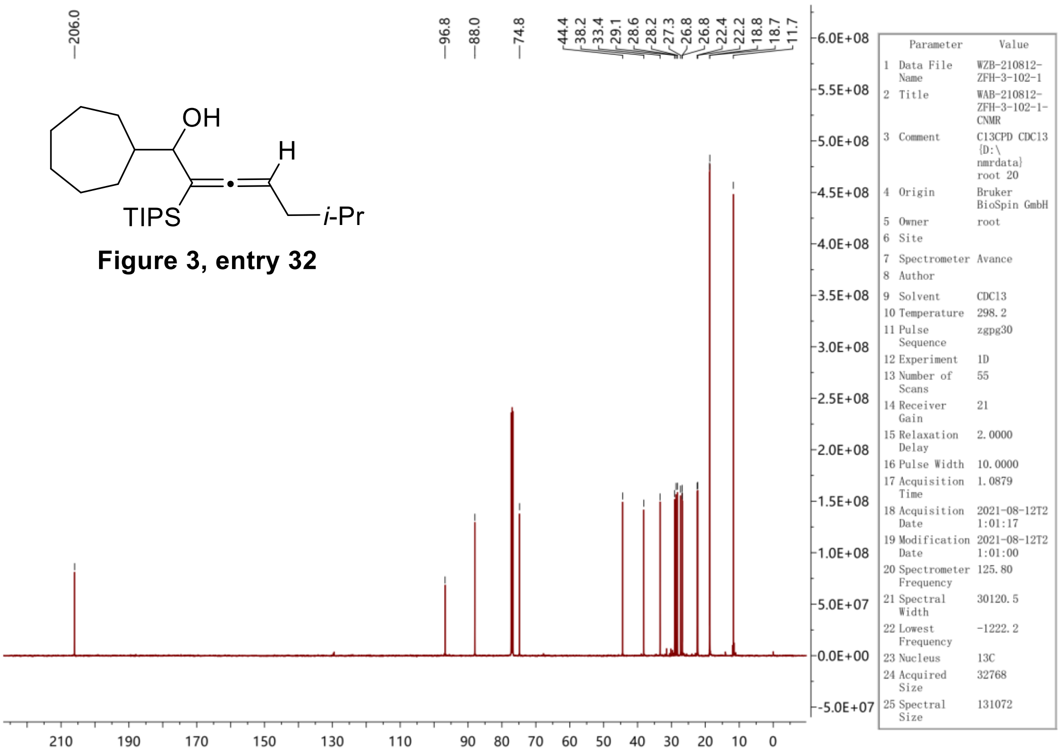

Supplementary Figure 75. ^{13}C NMR spectrum of compound **32**

^{1}H NMR (500 MHz, room temperature, $CDCl_3$)

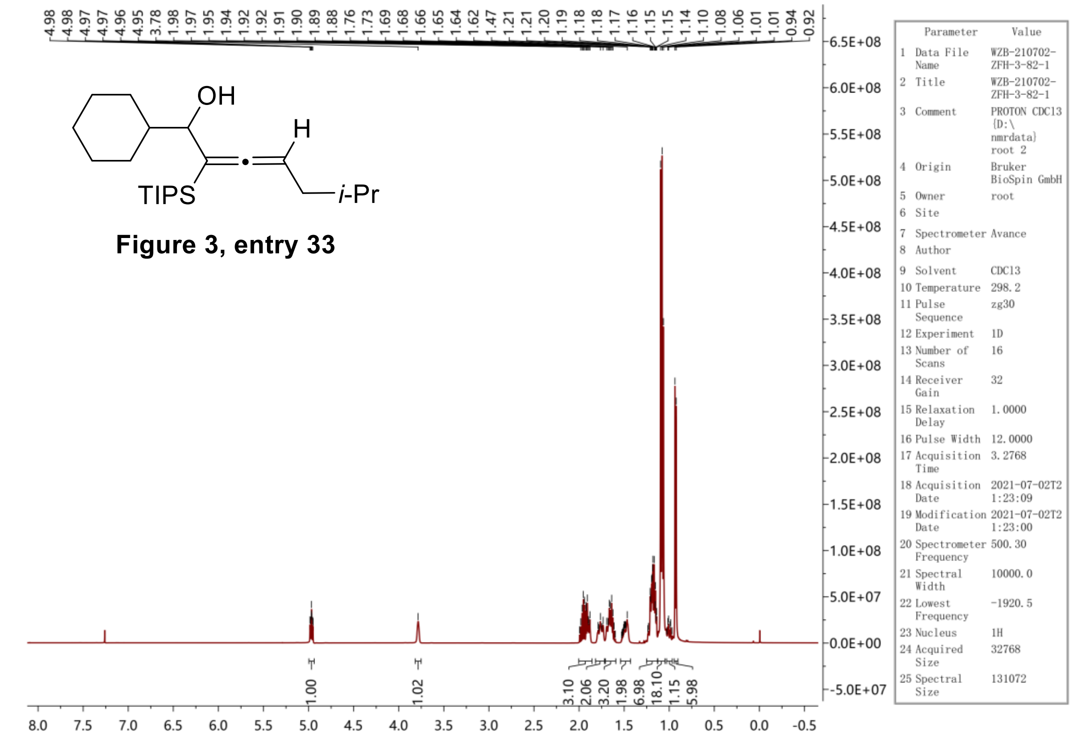

Supplementary Figure 76. ^{1}H NMR spectrum of compound **33**

^{13}C NMR (126 MHz, room temperature, $CDCl_3$)

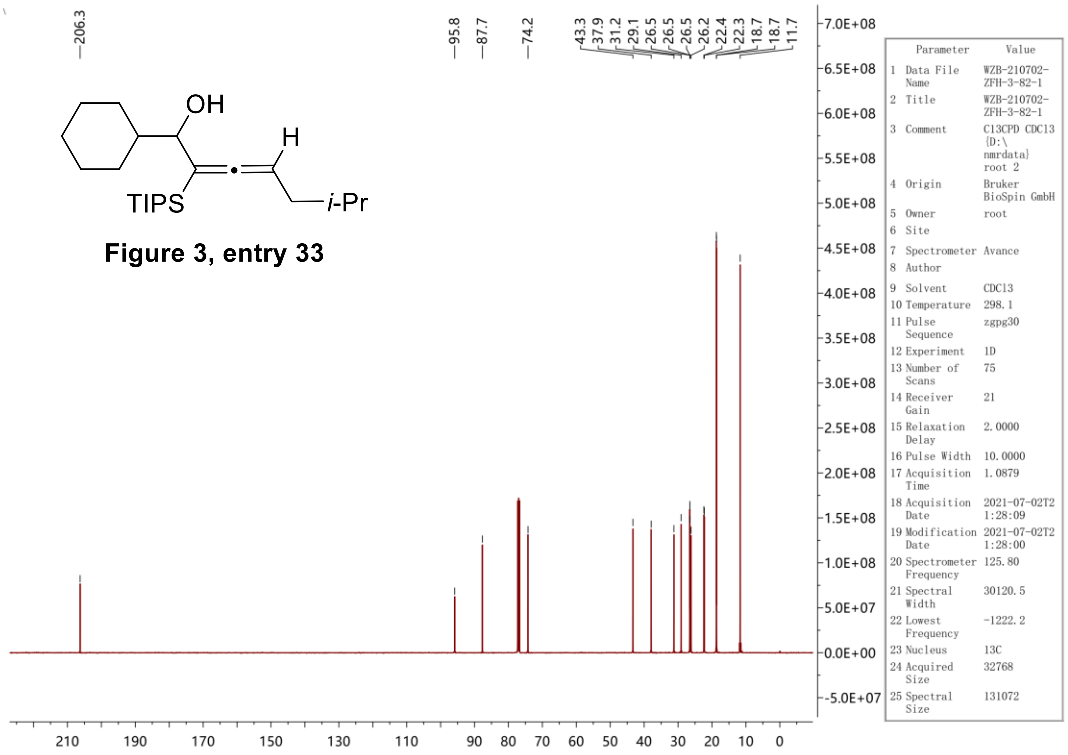

Supplementary Figure 77. ^{13}C NMR spectrum of compound **33**

^{1}H NMR (500 MHz, room temperature, $CDCl_3$)

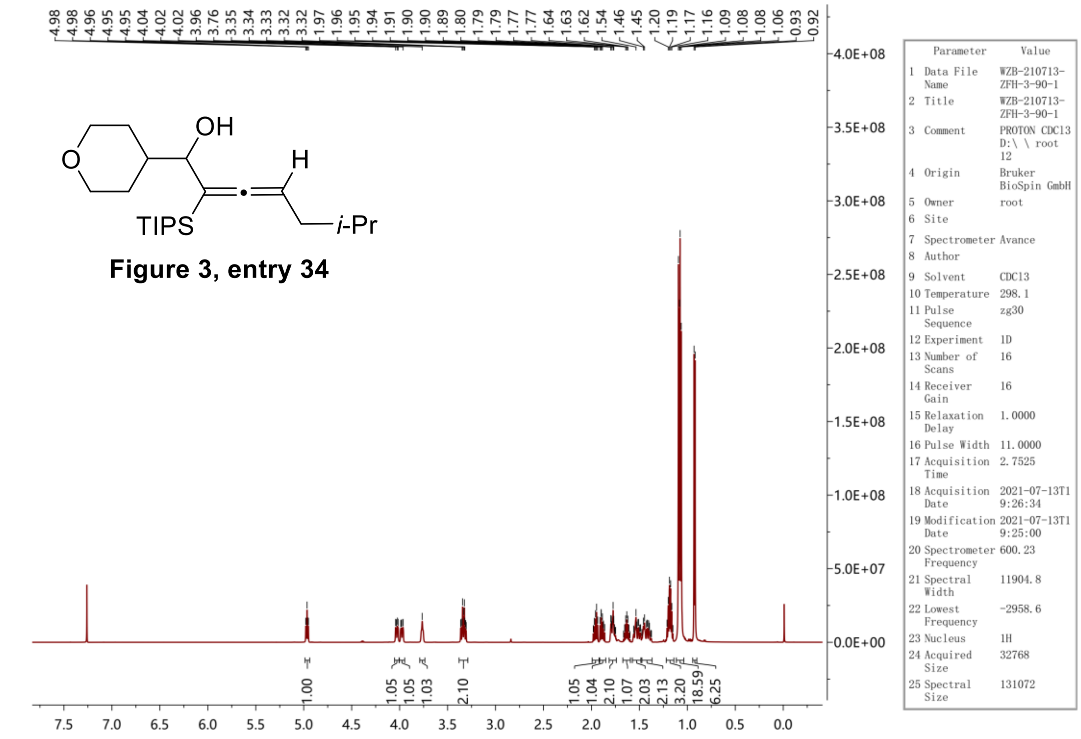

Supplementary Figure 78. ^{1}H NMR spectrum of compound **34**

^{13}C NMR (126 MHz, room temperature, $CDCl_3$)

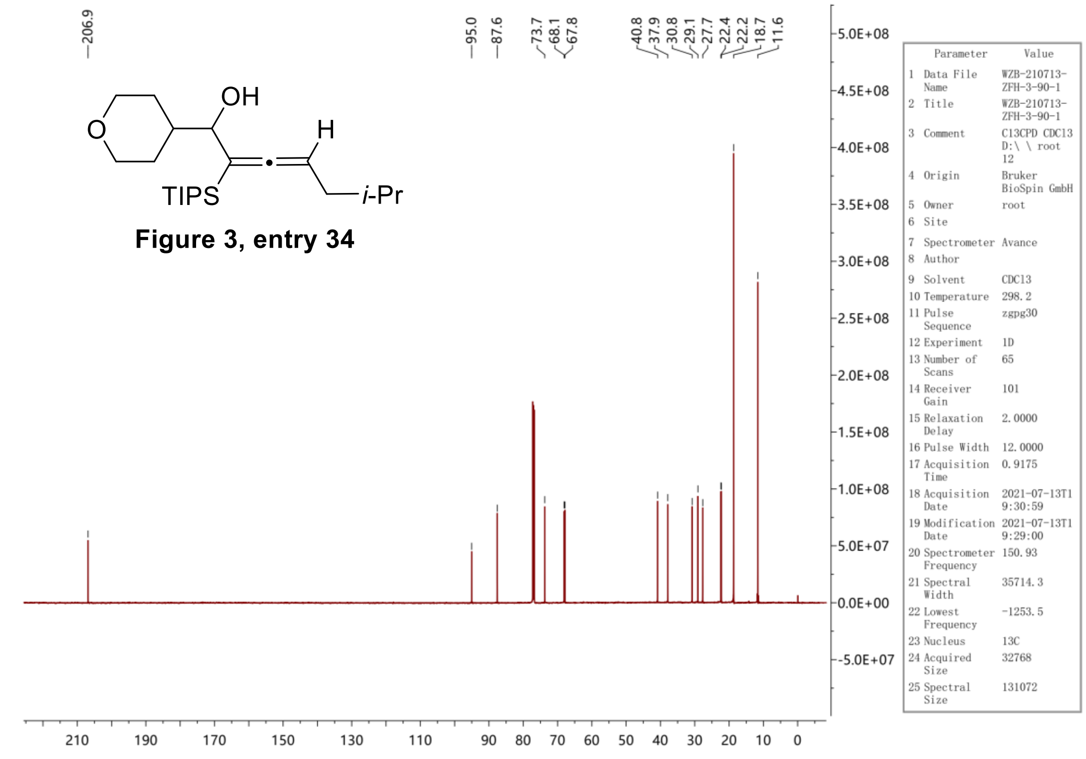

Supplementary Figure 79. ^{13}C NMR spectrum of compound **34**

^{1}H NMR (500 MHz, room temperature, $CDCl_3$)

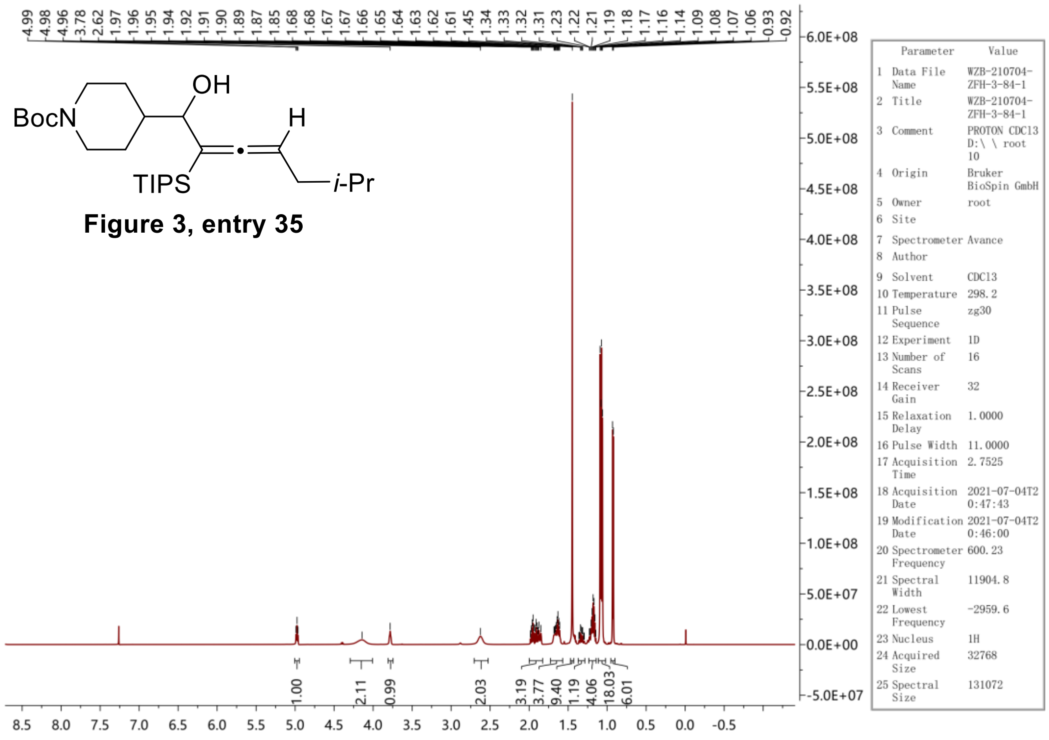

Supplementary Figure 80. ^{1}H NMR spectrum of compound **35**

^{13}C NMR (126 MHz, room temperature, $CDCl_3$)

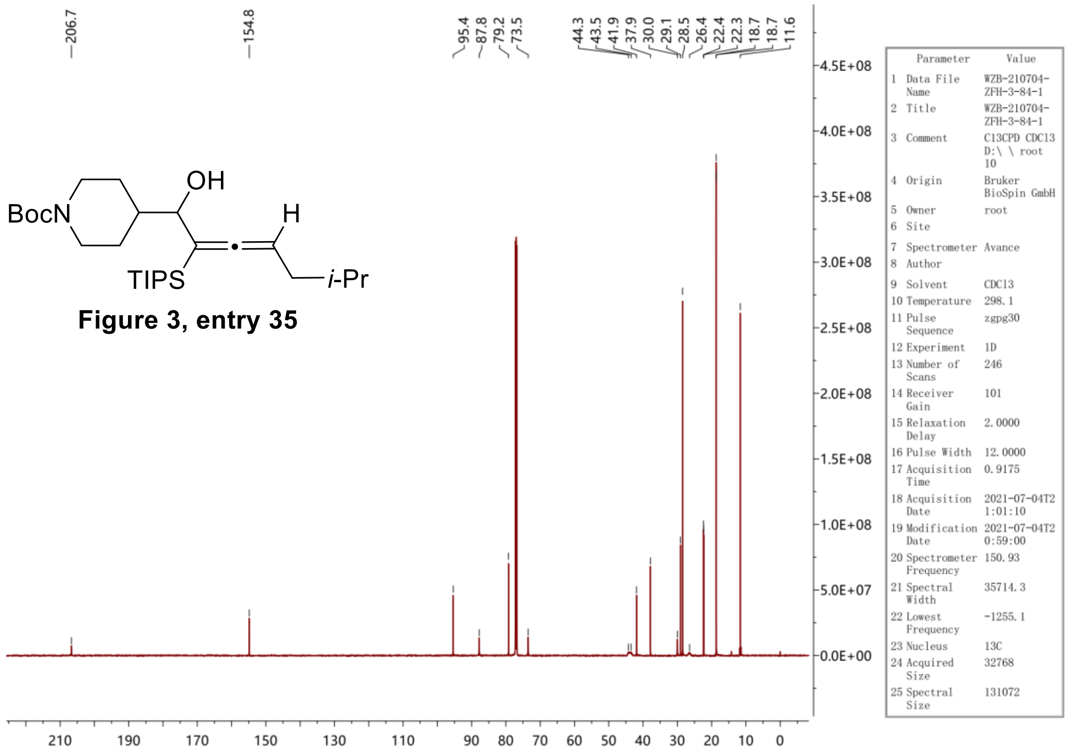

Supplementary Figure 81. ^{13}C NMR spectrum of compound **35**

^{1}H NMR (500 MHz, room temperature, $CDCl_3$)

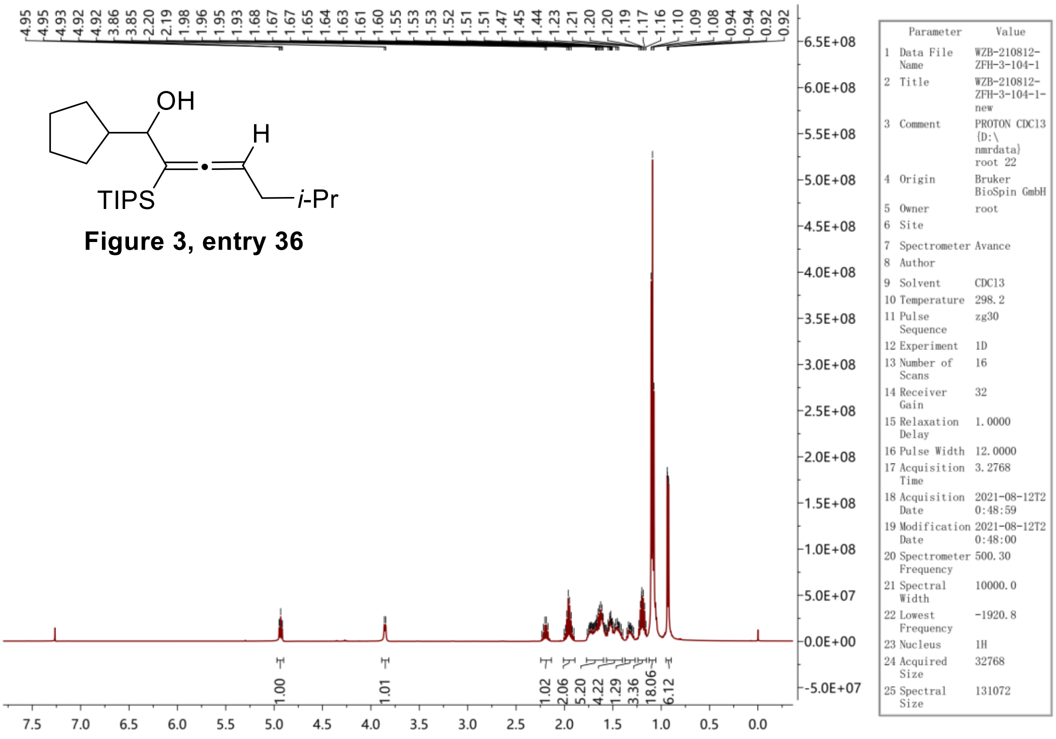

Supplementary Figure 82. ^{1}H NMR spectrum of compound **36**

^{13}C NMR (126 MHz, room temperature, $CDCl_3$)

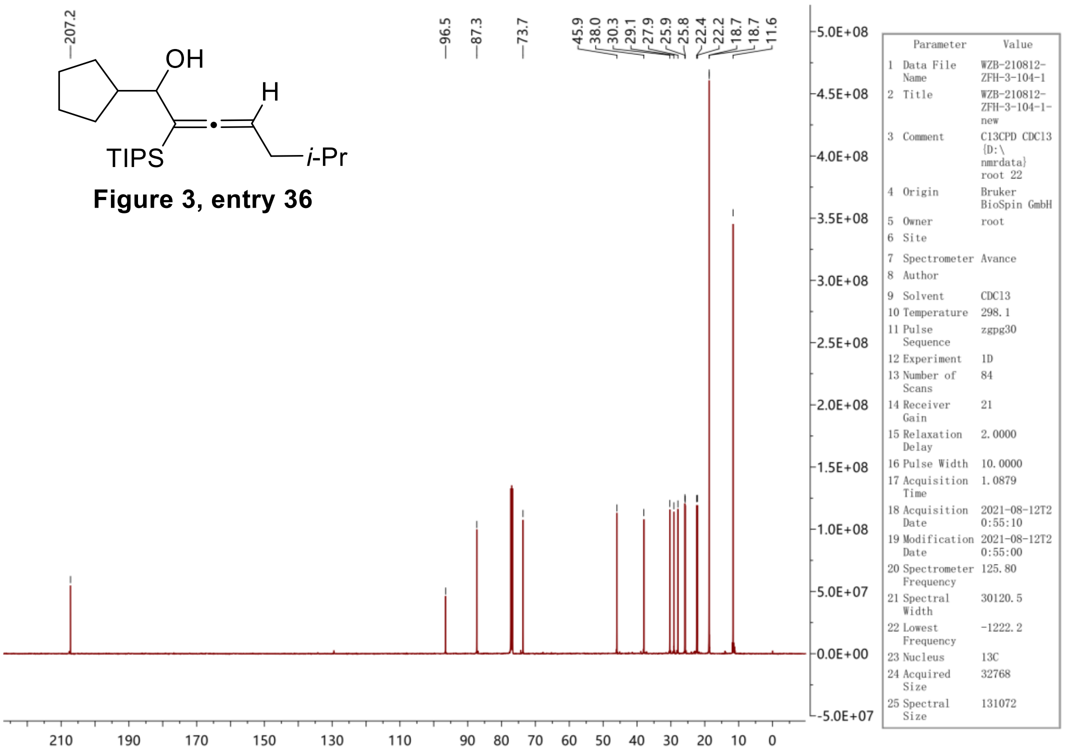

Supplementary Figure 83. ^{13}C NMR spectrum of compound **36**

^{1}H NMR (500 MHz, room temperature, $CDCl_3$)

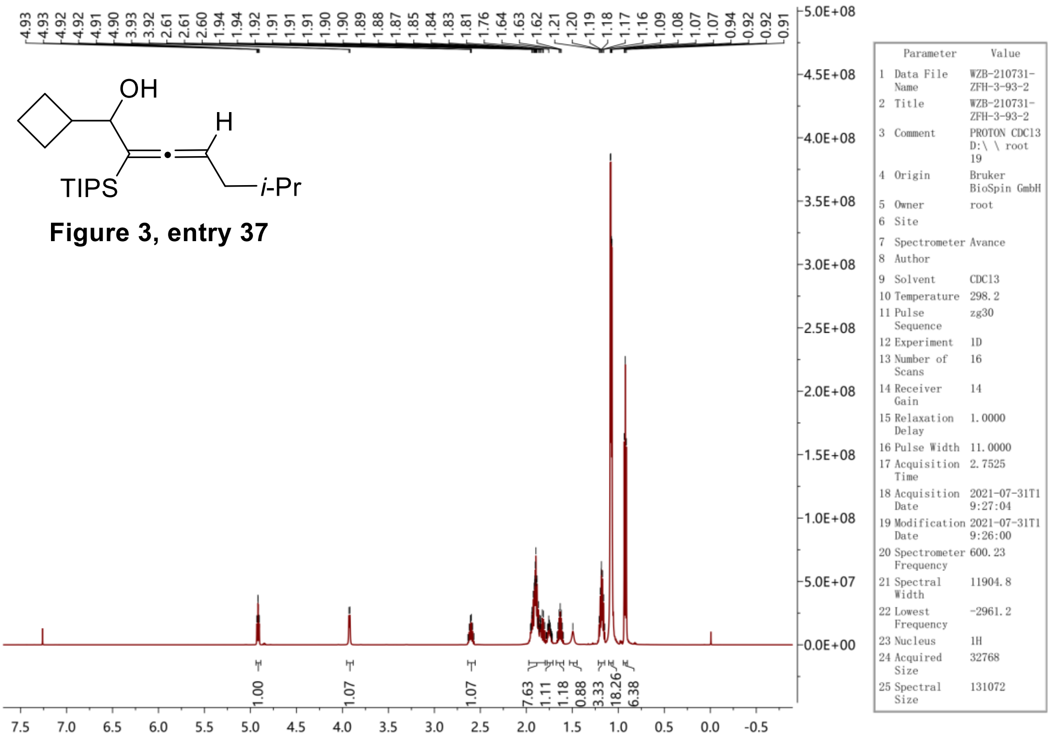

Supplementary Figure 84. ^{1}H NMR spectrum of compound **37**

^{13}C NMR (126 MHz, room temperature, $CDCl_3$)

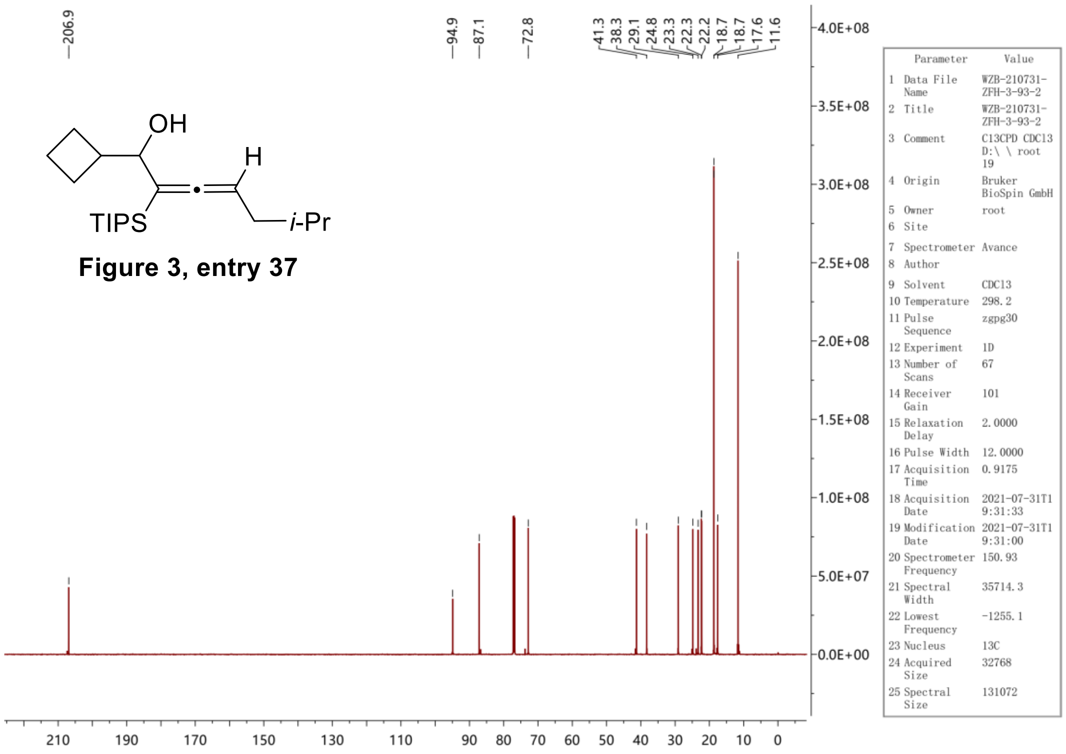

Supplementary Figure 85. ^{13}C NMR spectrum of compound **37**

^{1}H NMR (500 MHz, room temperature, $CDCl_3$)

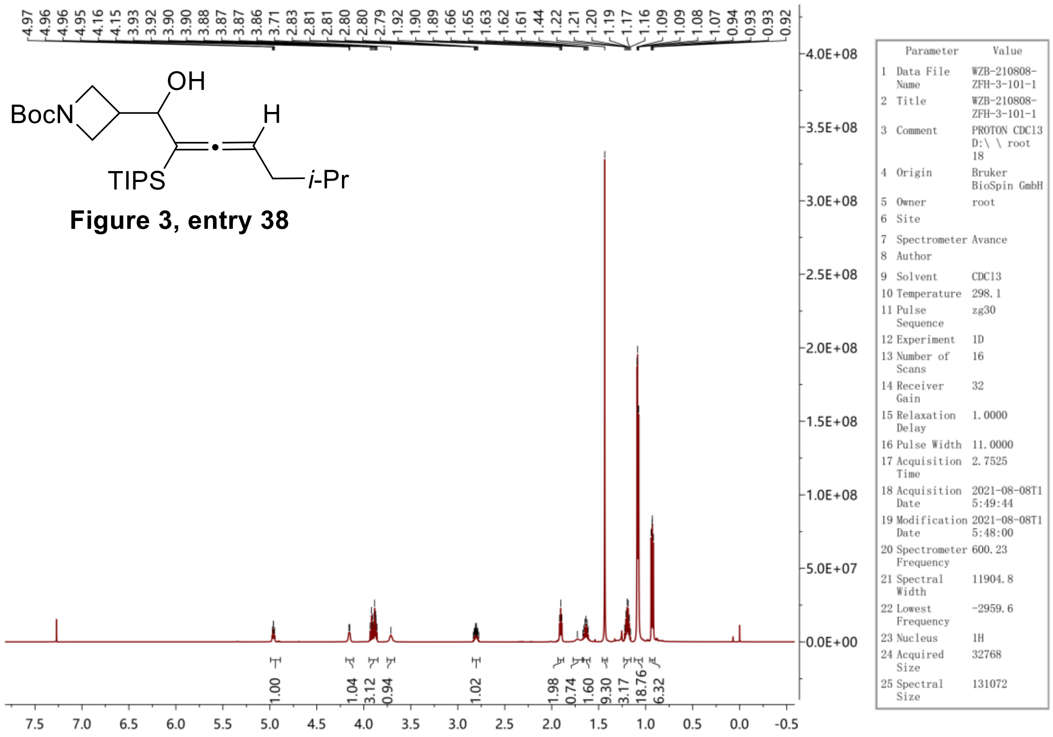

Supplementary Figure 86. ^{1}H NMR spectrum of compound **38**

^{13}C NMR (126 MHz, room temperature, $CDCl_3$)

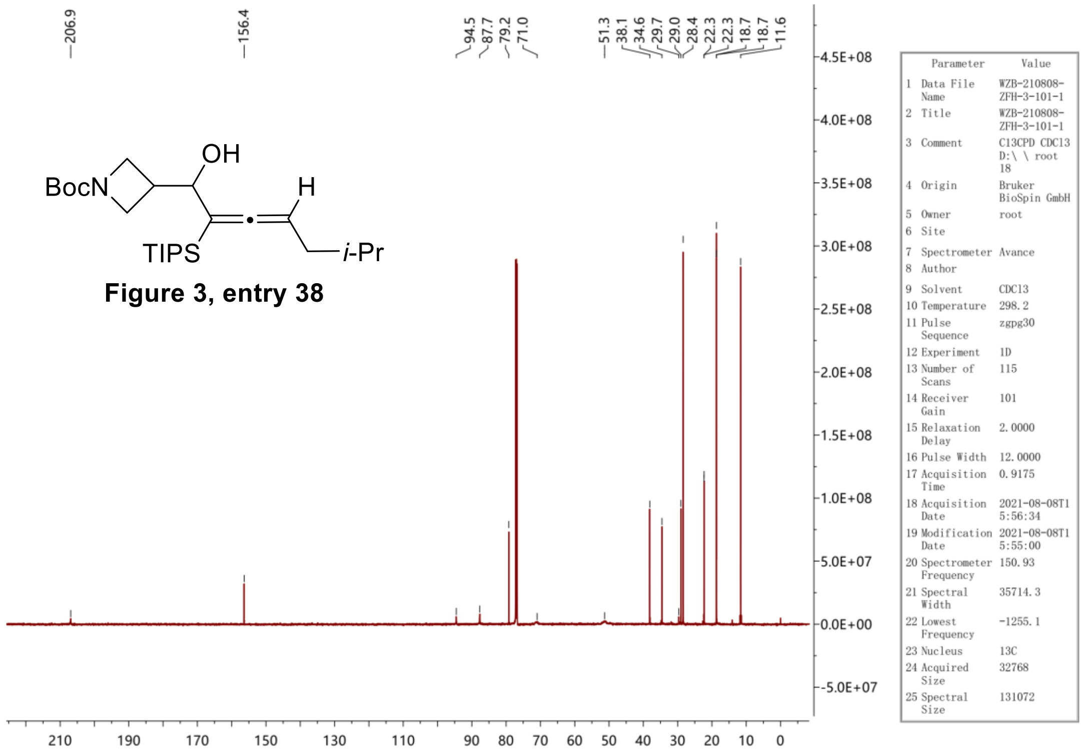

Supplementary Figure 87. ^{13}C NMR spectrum of compound **38**

^{1}H NMR (500 MHz, room temperature, $CDCl_3$)

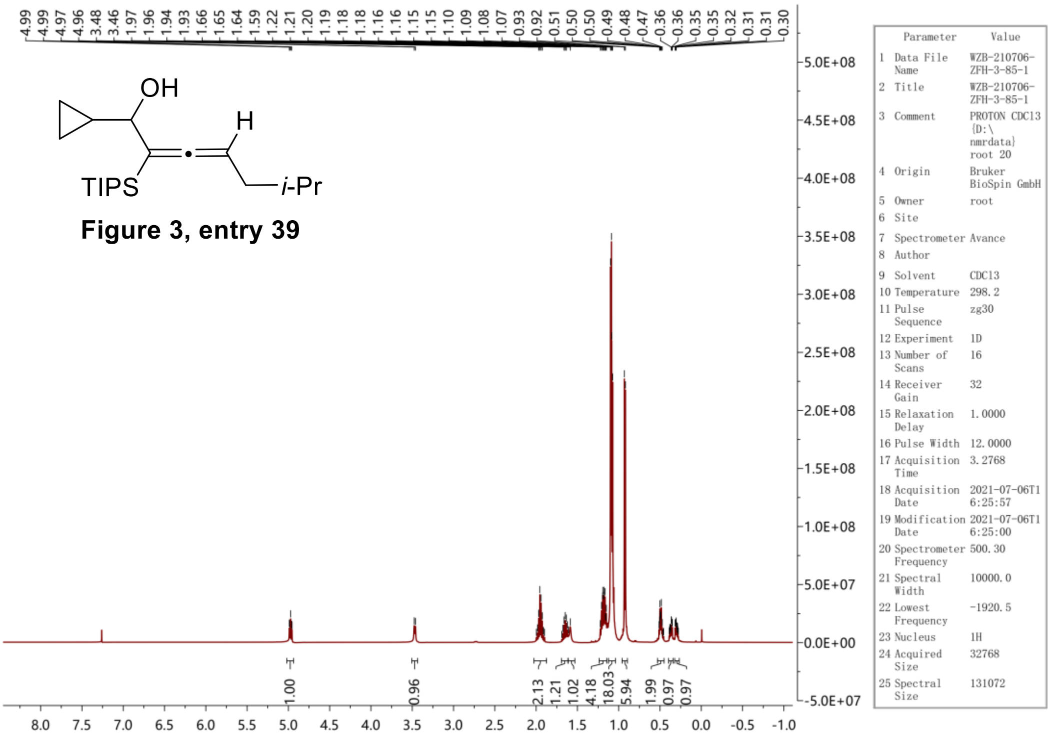

Supplementary Figure 88. ^{1}H NMR spectrum of compound **39**

^{13}C NMR (126 MHz, room temperature, $CDCl_3$)

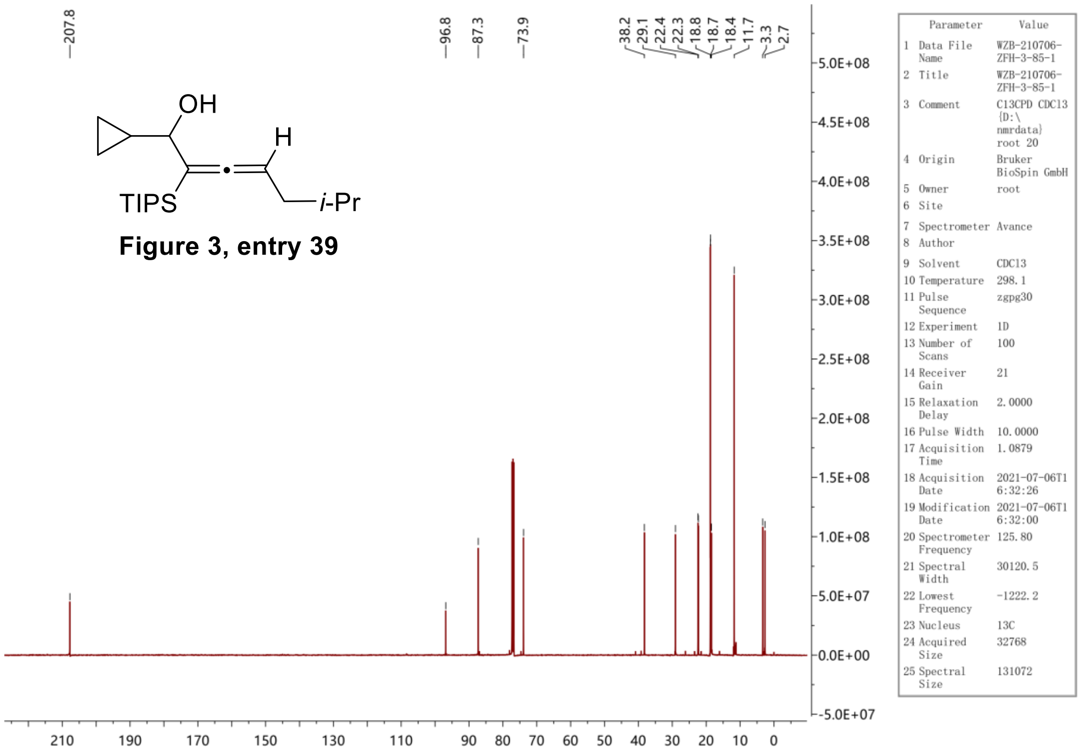

Supplementary Figure 89. ^{13}C NMR spectrum of compound **39**

^1H NMR (500 MHz, 60 °C, C_6D_6)

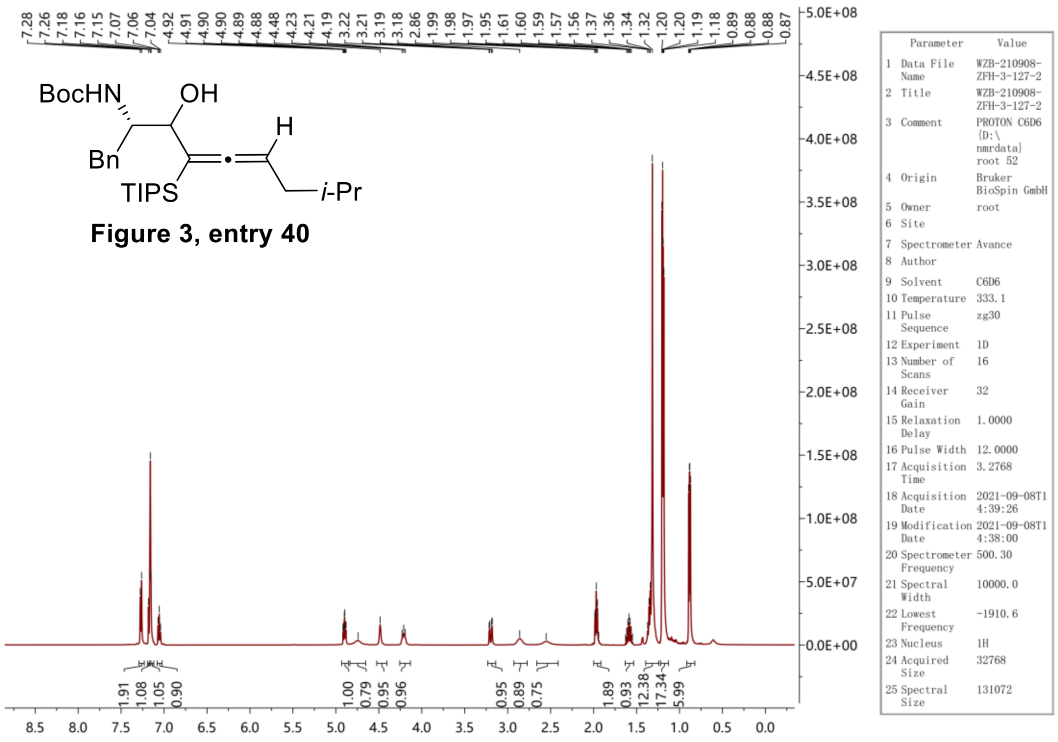

Supplementary Figure 90. ^1H NMR spectrum of compound **40**

^{13}C NMR (126 MHz, 60 °C, C_6D_6)

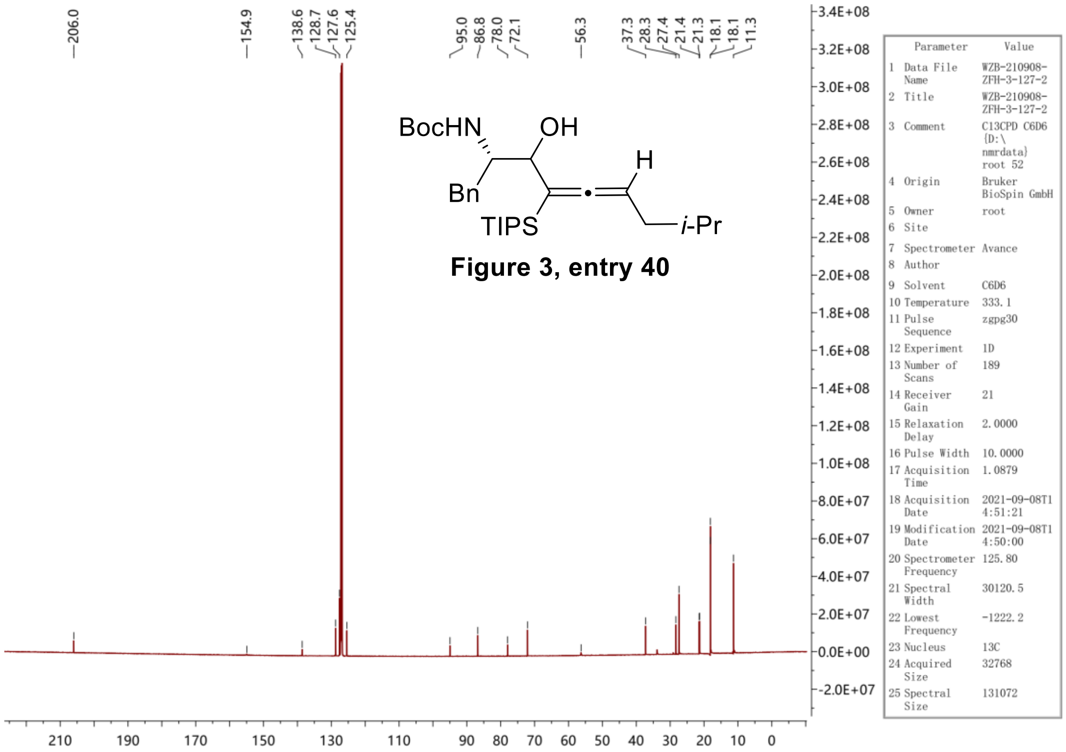

Supplementary Figure 91. ^{13}C NMR spectrum of compound **40**

^{1}H NMR (500 MHz, 60 °C, C_6D_6)

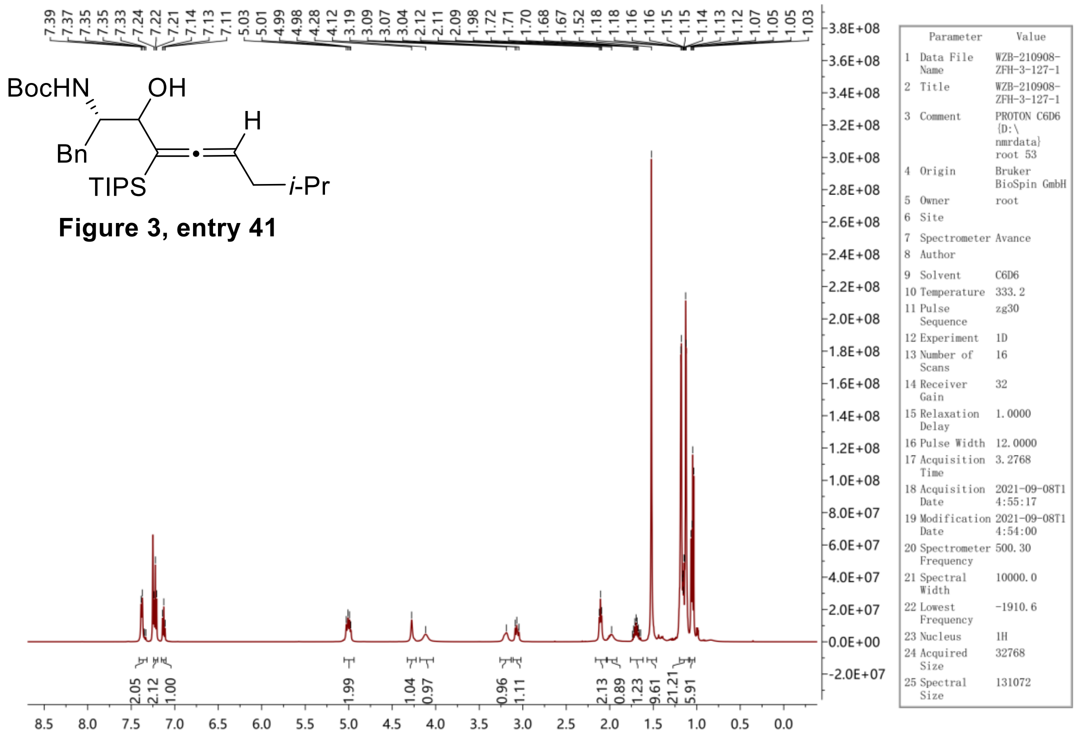

Supplementary Figure 92. ^{1}H NMR spectrum of compound **41**

^{13}C NMR (126 MHz, 60 °C, C_6D_6)

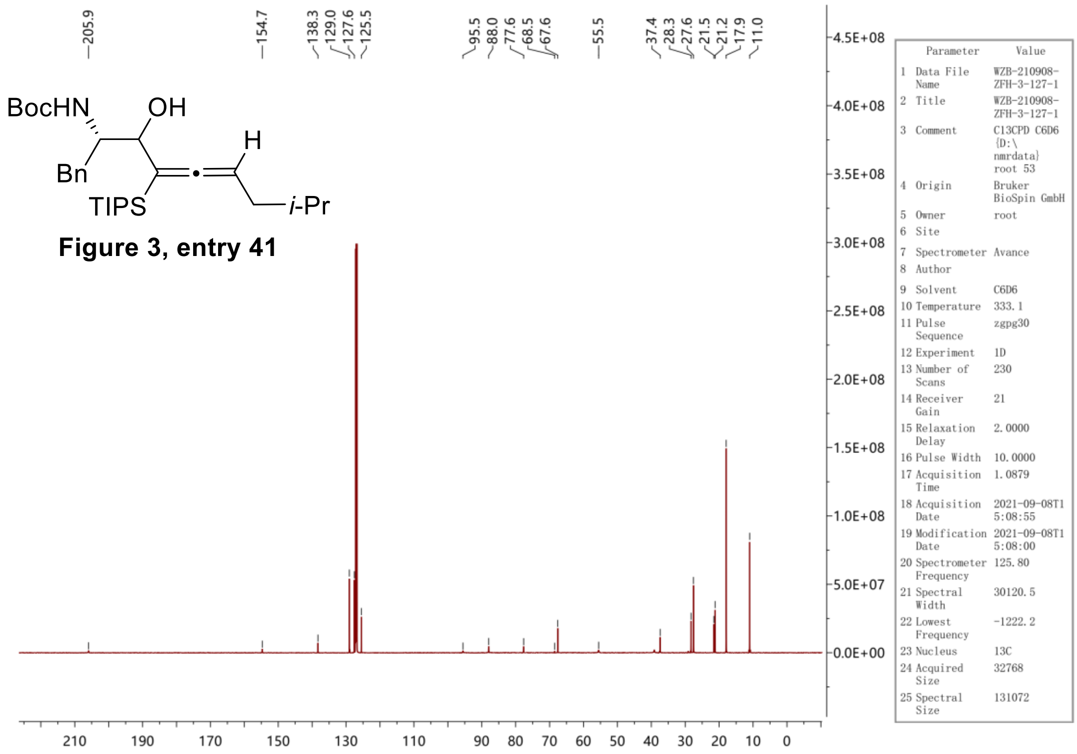

Supplementary Figure 93. ^{13}C NMR spectrum of compound **41**

^{1}H NMR (500 MHz, 60 °C, C_6D_6)

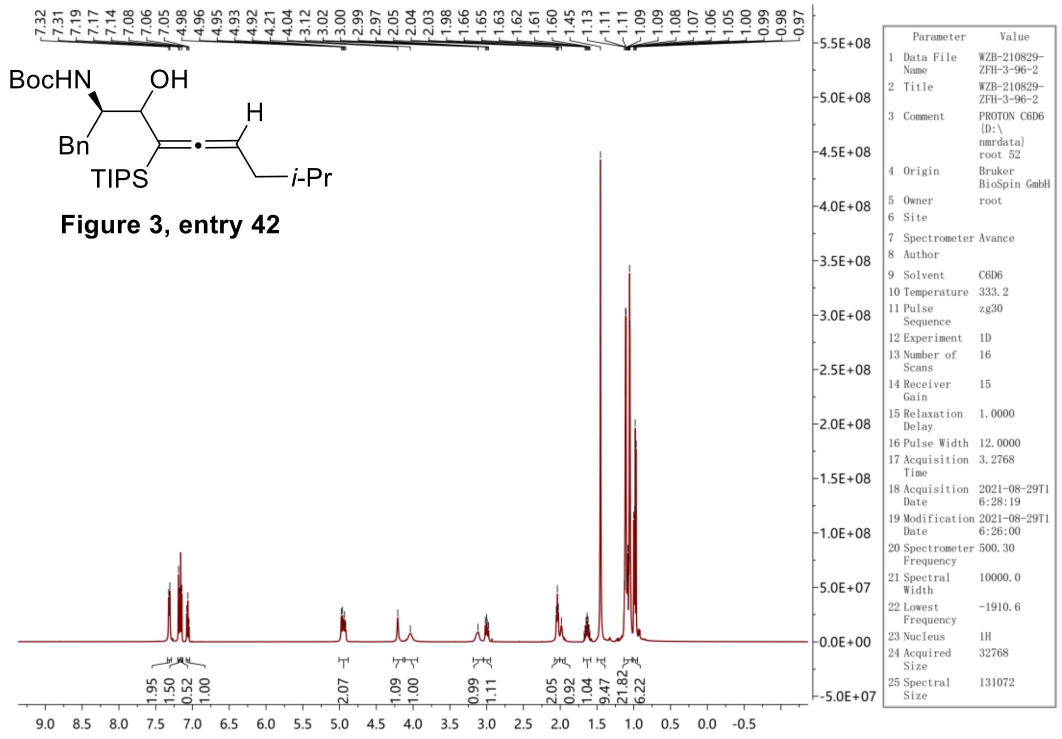

Supplementary Figure 94. ^{1}H NMR spectrum of compound **42**

^{13}C NMR (126 MHz, 60 °C, C_6D_6)

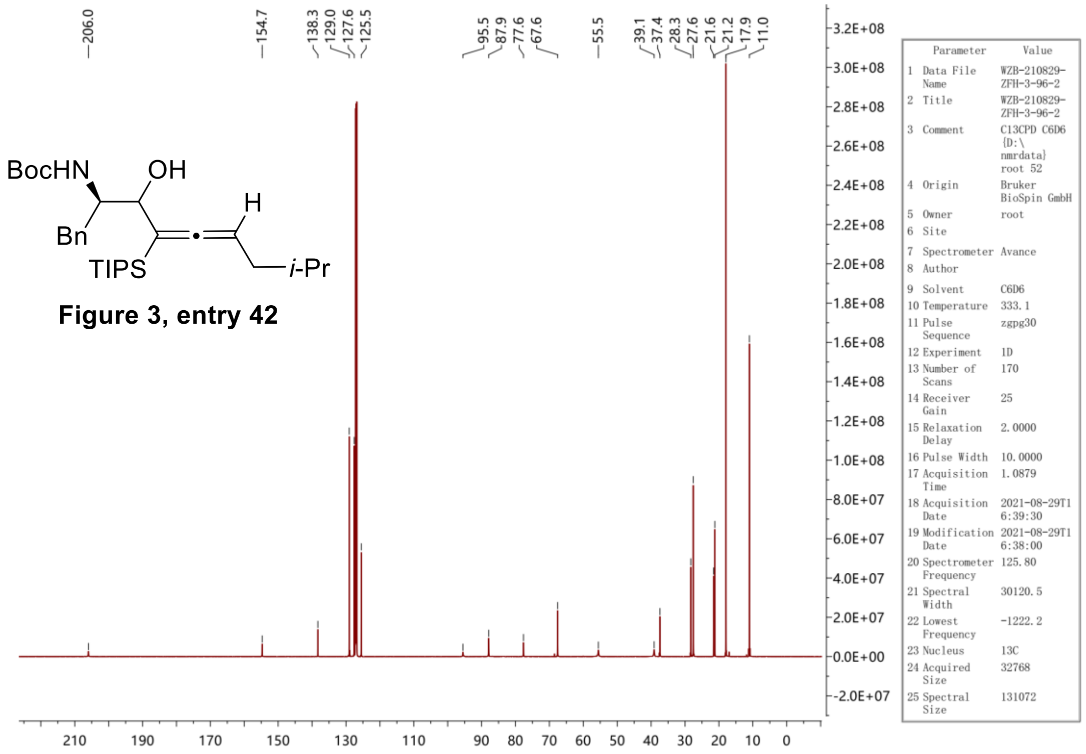

Supplementary Figure 95 ^{13}C NMR spectrum of compound **42**

^{1}H NMR (500 MHz, 60 °C, C_6D_6)

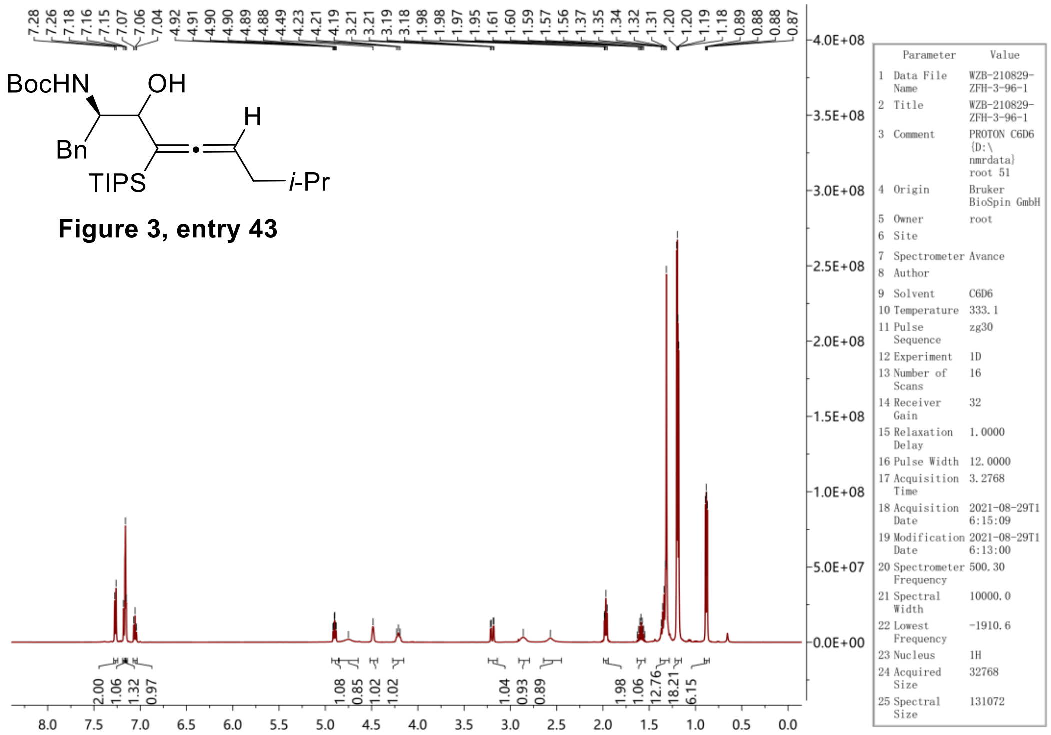

Supplementary Figure 96. ^{1}H NMR spectrum of compound **43**

^{13}C NMR (126 MHz, 60 °C, C_6D_6)

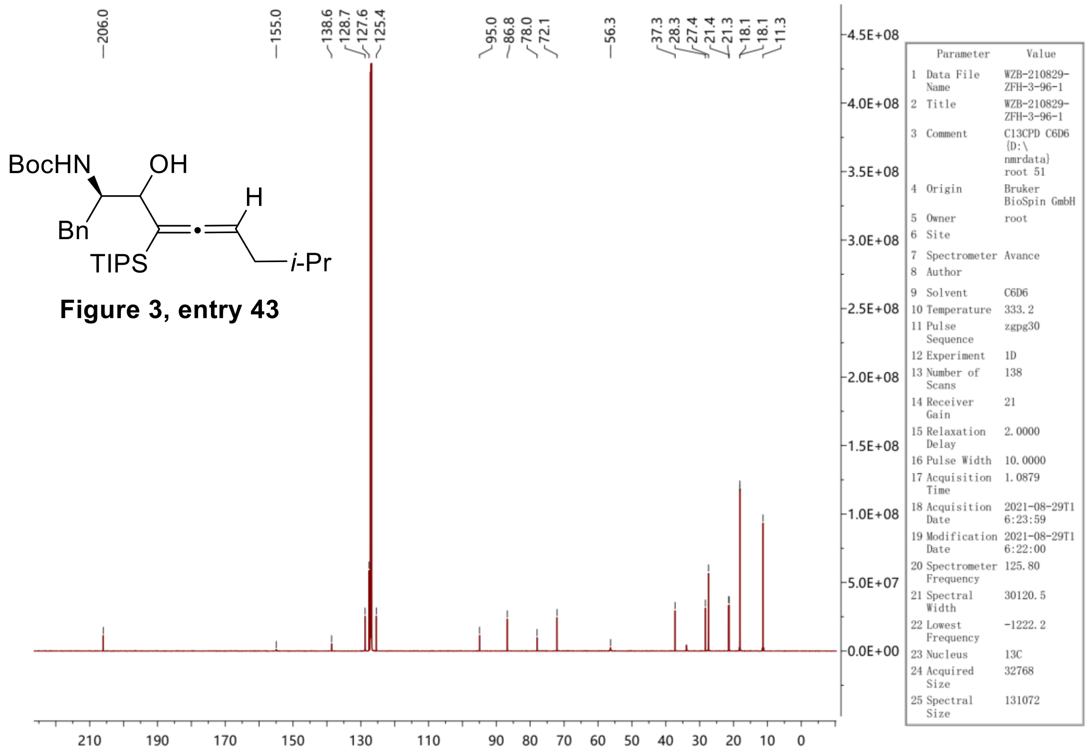

Supplementary Figure 97. ^{13}C NMR spectrum of compound **43**

^1H NMR (500 MHz, 60 °C, C_6D_6)

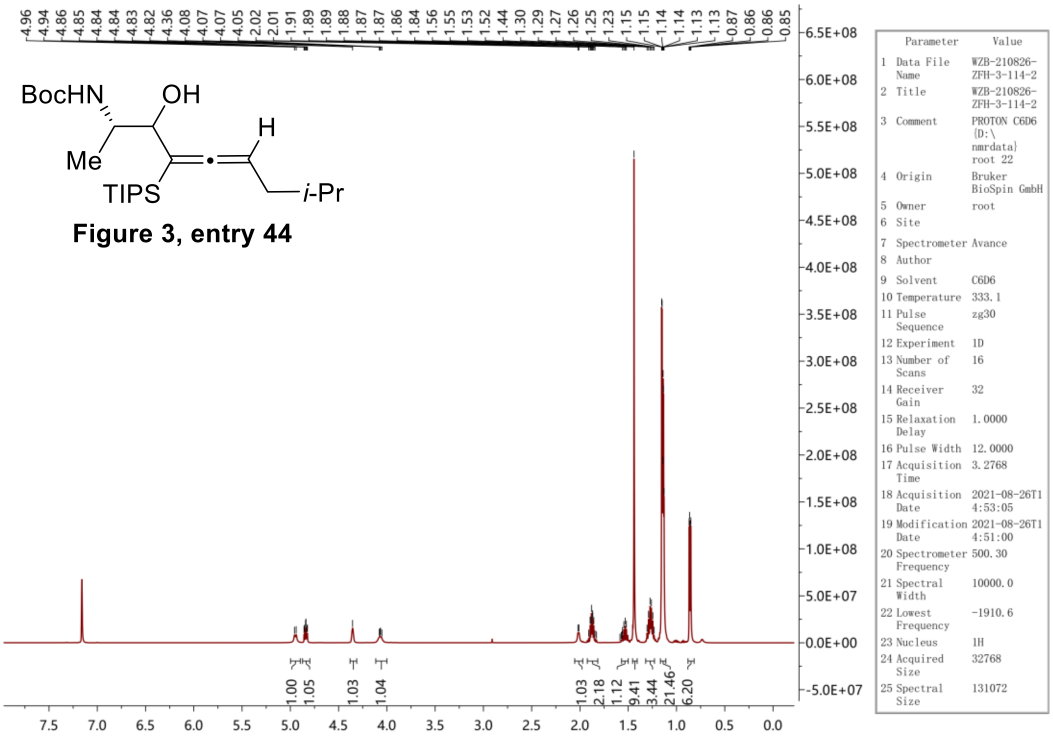

Supplementary Figure 98. ^1H NMR spectrum of compound **44**

^{13}C NMR (126 MHz, 60 °C, C_6D_6)

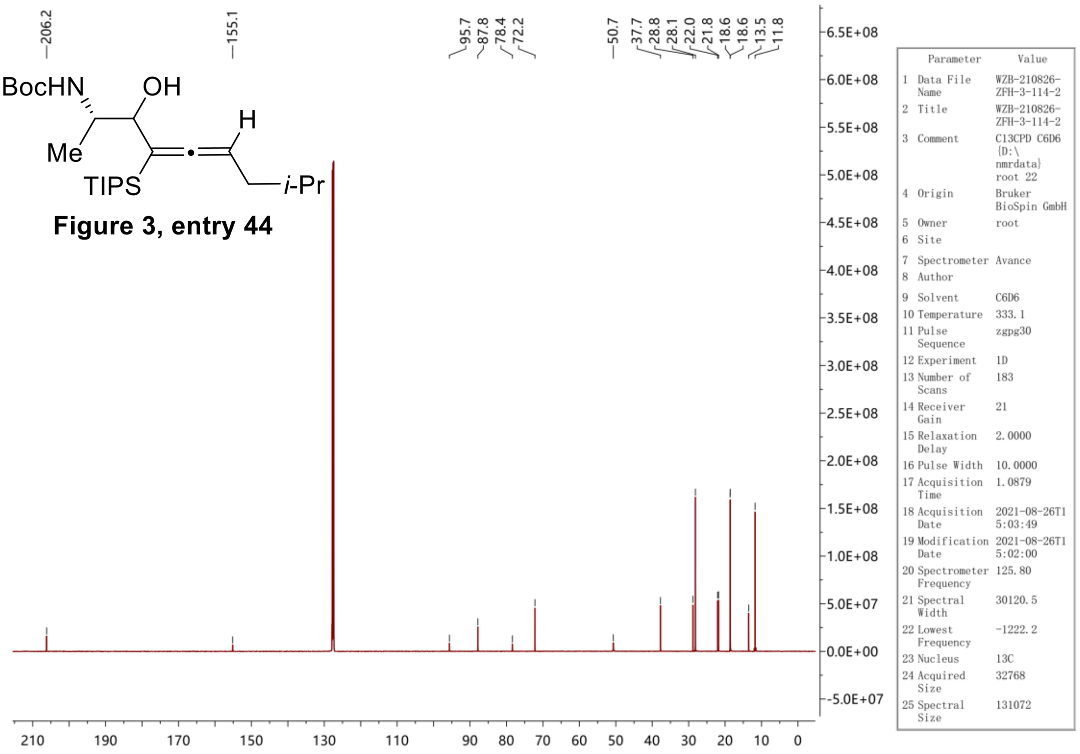

Supplementary Figure 99. ^{13}C NMR spectrum of compound **44**

^{1}H NMR (500 MHz, 60 °C, C_6D_6)

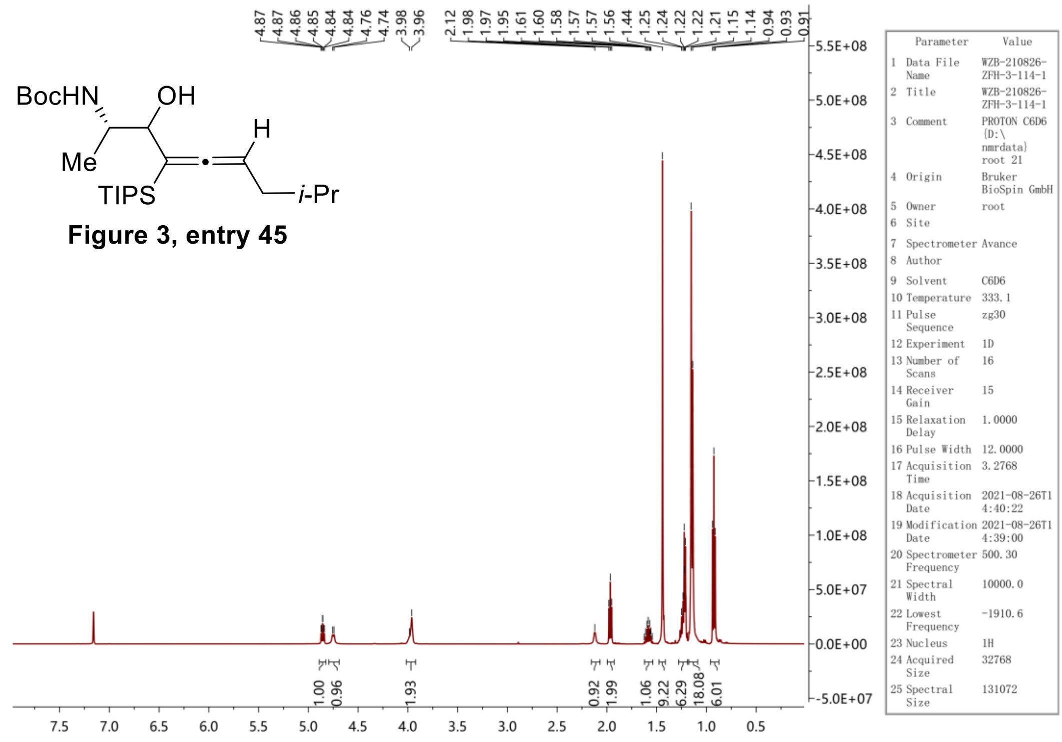

Supplementary Figure 100. ^{1}H NMR spectrum of compound **45**

^{13}C NMR (126 MHz, 60 °C, C_6D_6)

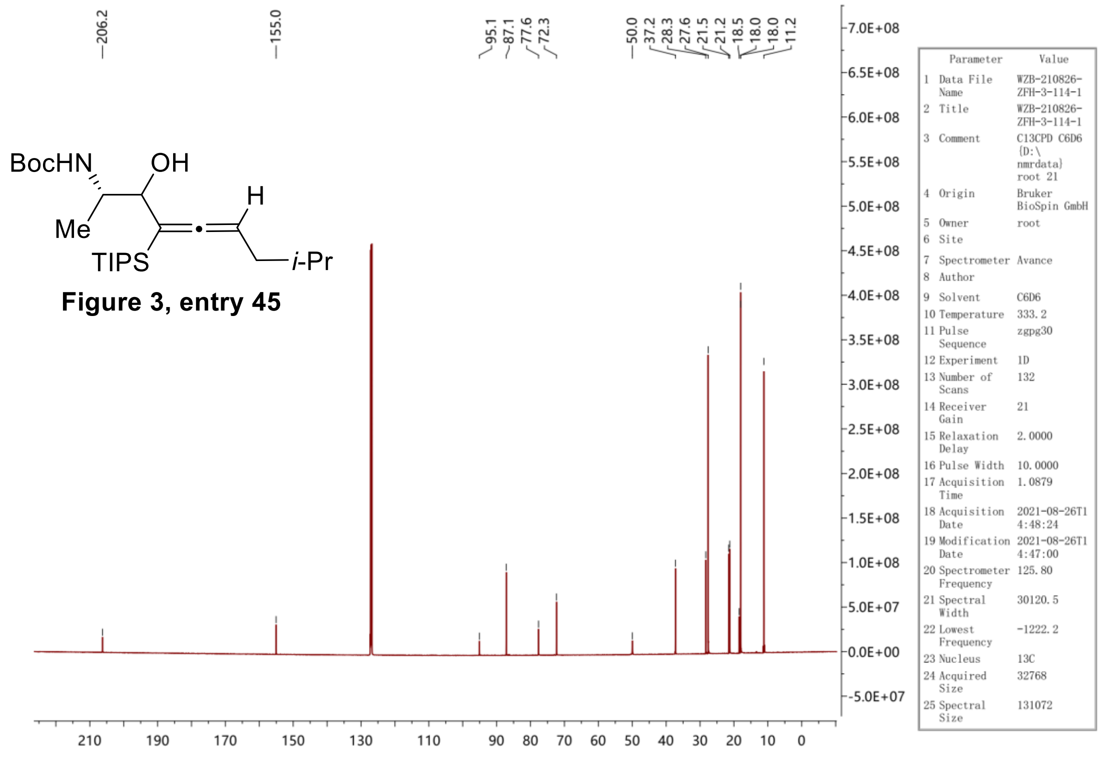

Supplementary Figure 101. ^{13}C NMR spectrum of compound **45**

^{1}H NMR (500 MHz, 60 °C, C_6D_6)

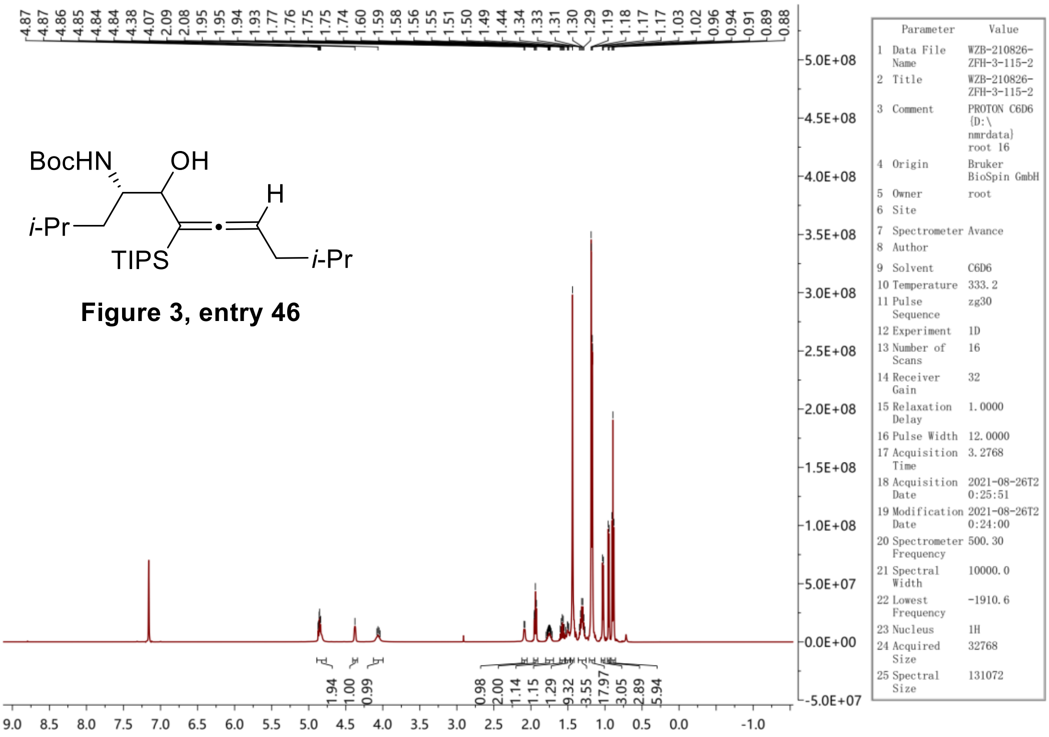

Supplementary Figure 102. ^{1}H NMR spectrum of compound **46**

^{13}C NMR (126 MHz, 60 °C, C_6D_6)

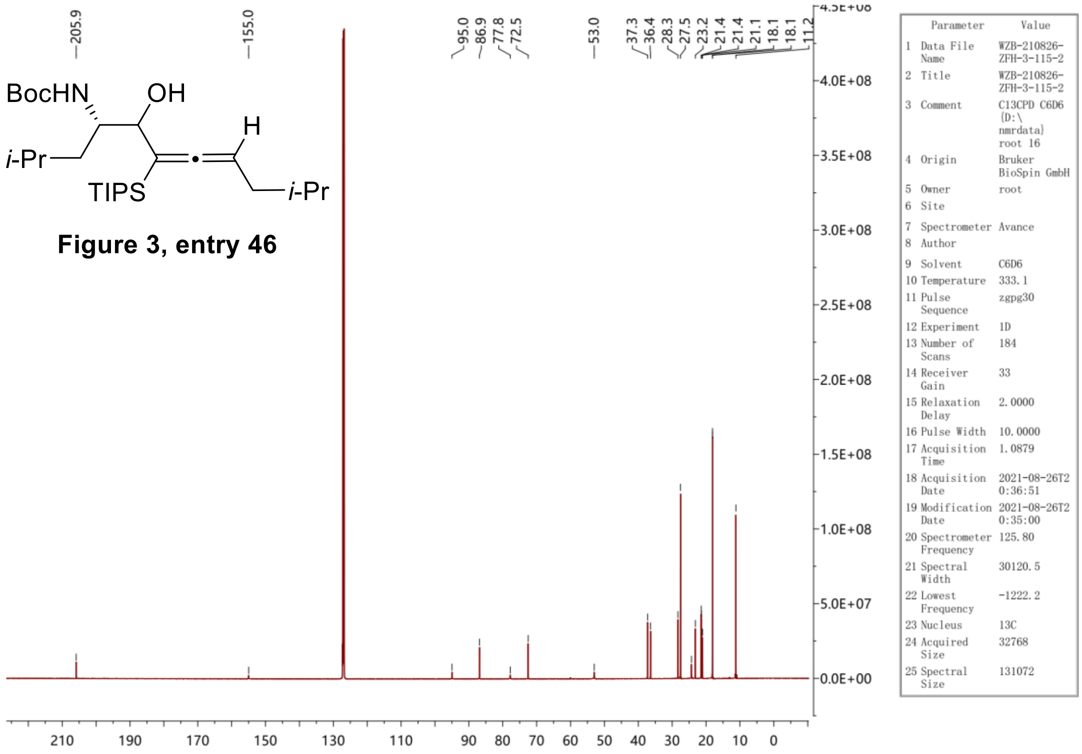

Supplementary Figure 103. ^{13}C NMR spectrum of compound **46**

^{1}H NMR (500 MHz, 60 °C, C_6D_6)

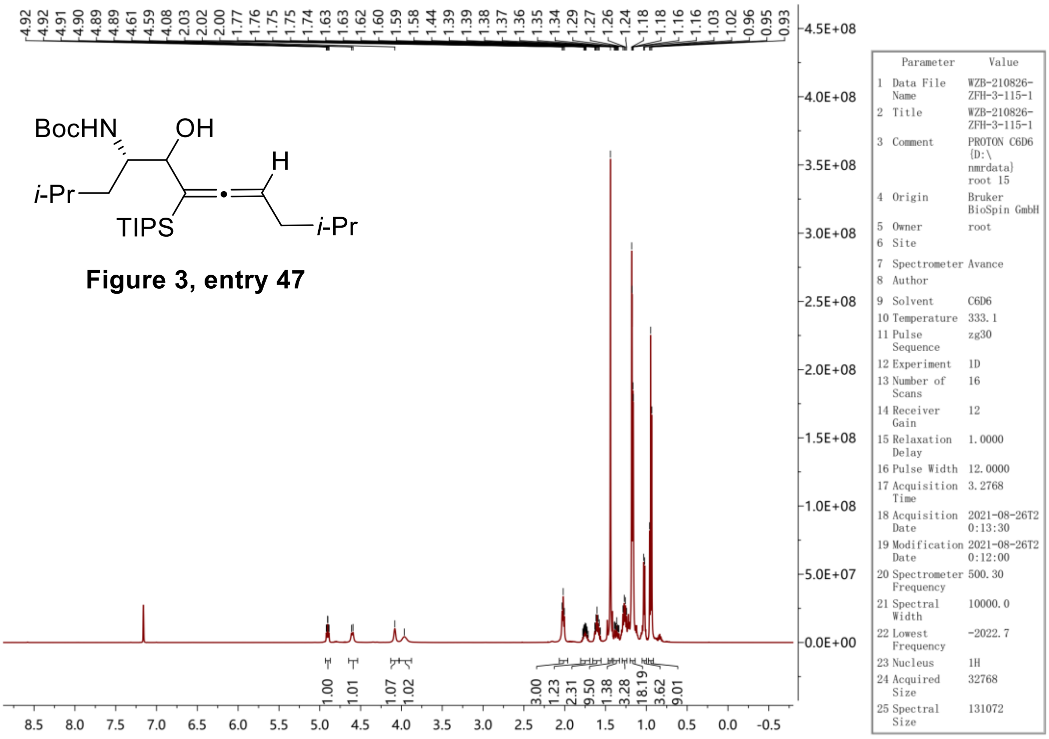

Supplementary Figure 104. ^{1}H NMR spectrum of compound **47**

^{13}C NMR (126 MHz, 60 °C, C_6D_6)

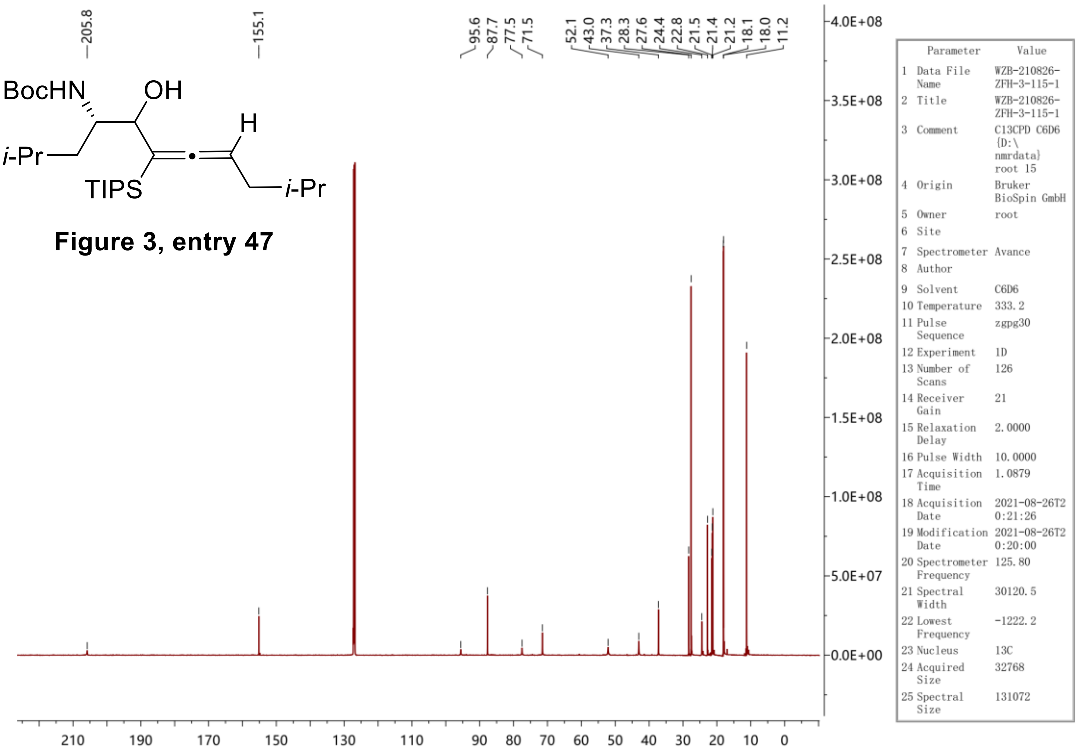

Supplementary Figure 105. ^{13}C NMR spectrum of compound **47**

^{1}H NMR (500 MHz, 60 °C, C_6D_6)

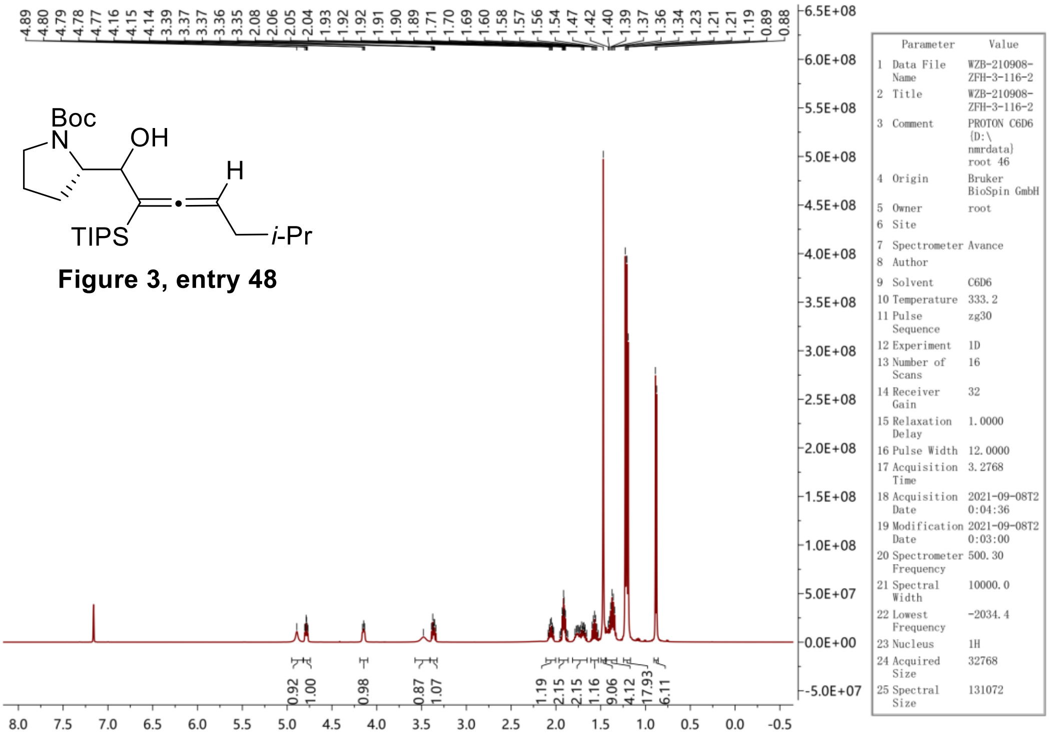

Supplementary Figure 106. ^{1}H NMR spectrum of compound **48**

^{13}C NMR (126 MHz, 60 °C, C_6D_6)

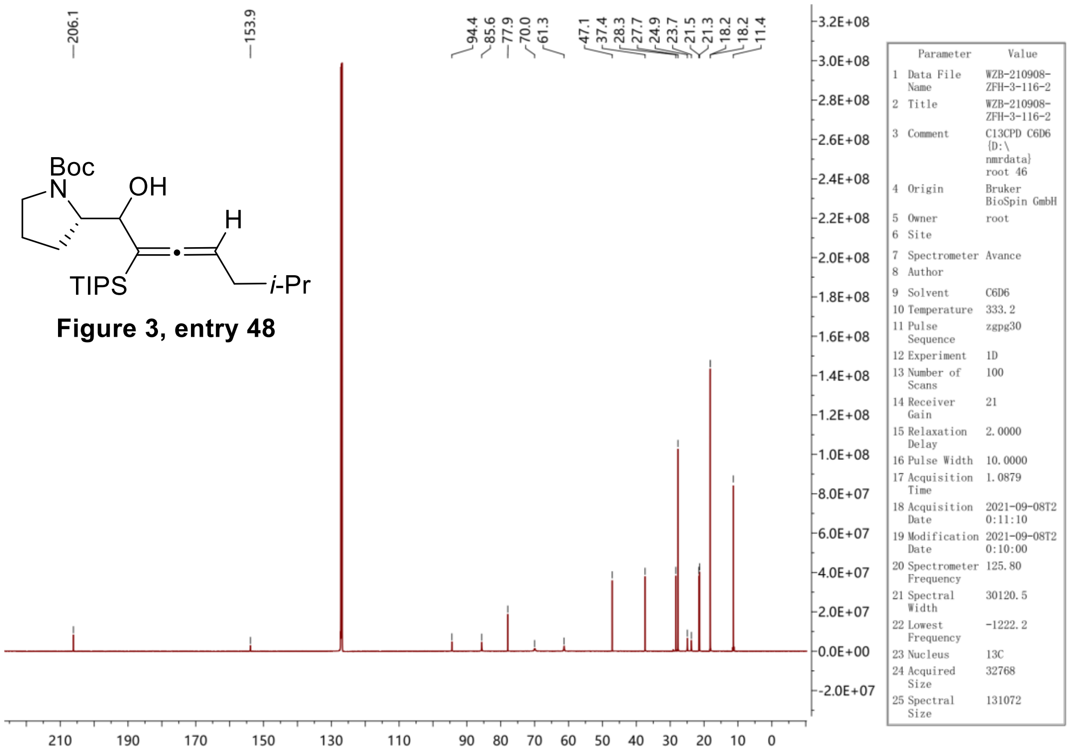

Supplementary Figure 107. ^{13}C NMR spectrum of compound **48**

^{1}H NMR (500 MHz, 60 °C, C_6D_6)

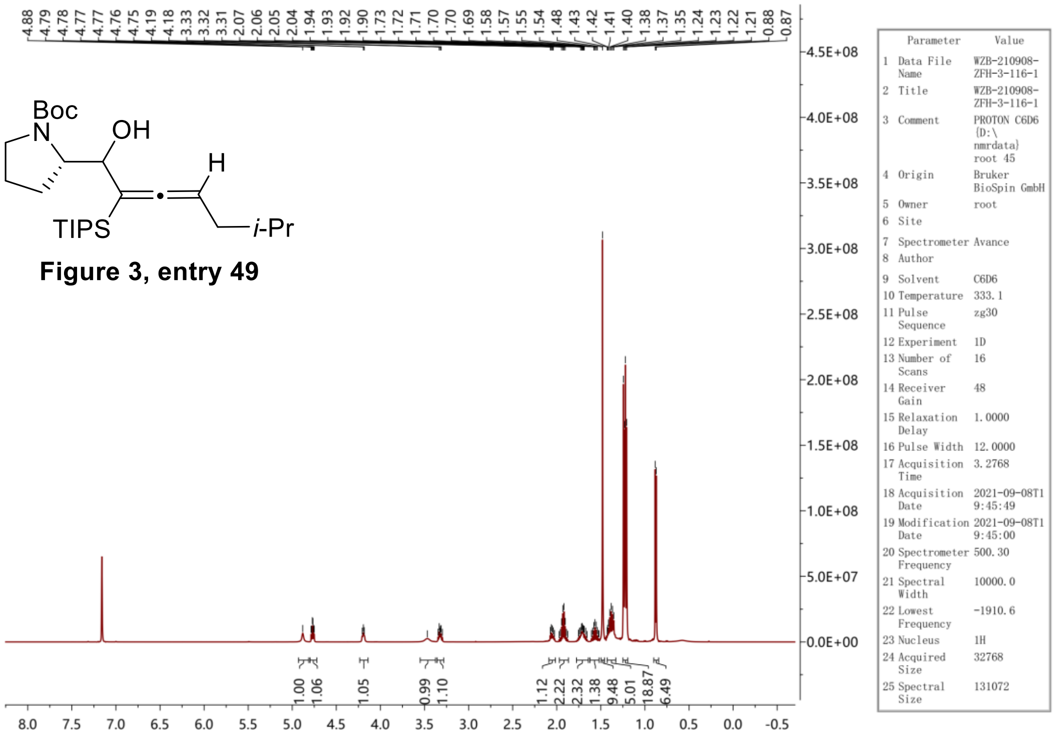

Supplementary Figure 108. ^{1}H NMR spectrum of compound **49**

^{13}C NMR (126 MHz, 60 °C, C_6D_6)

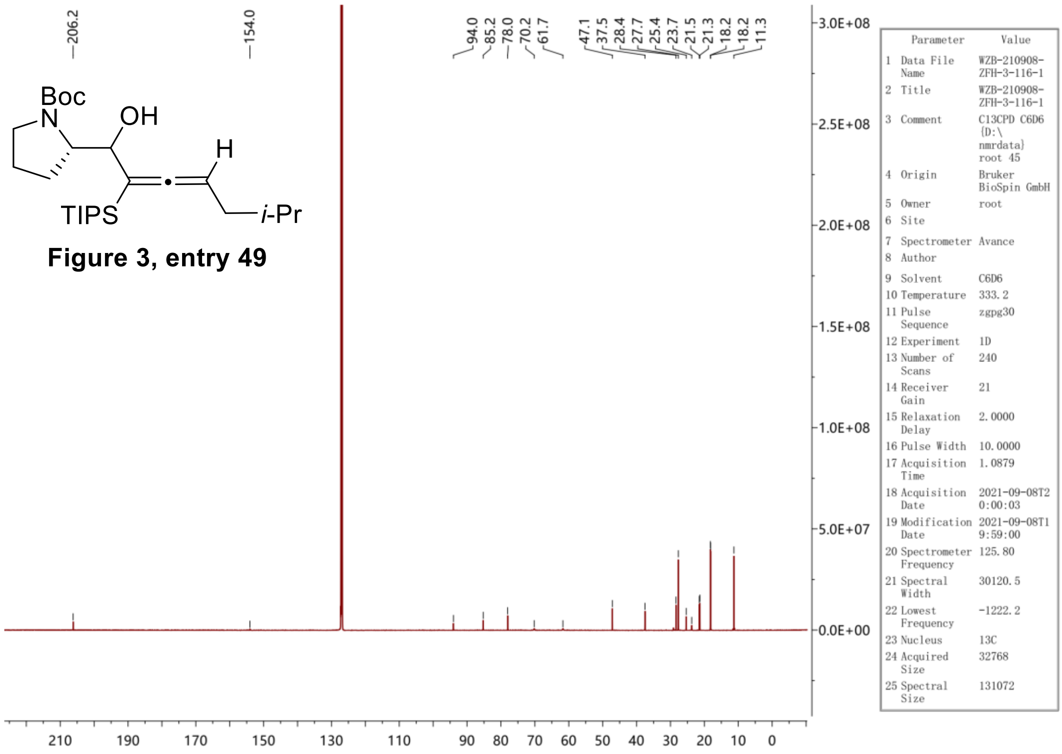

Supplementary Figure 109. ^{13}C NMR spectrum of compound **49**

^{1}H NMR (500 MHz, room temperature, $CDCl_3$)

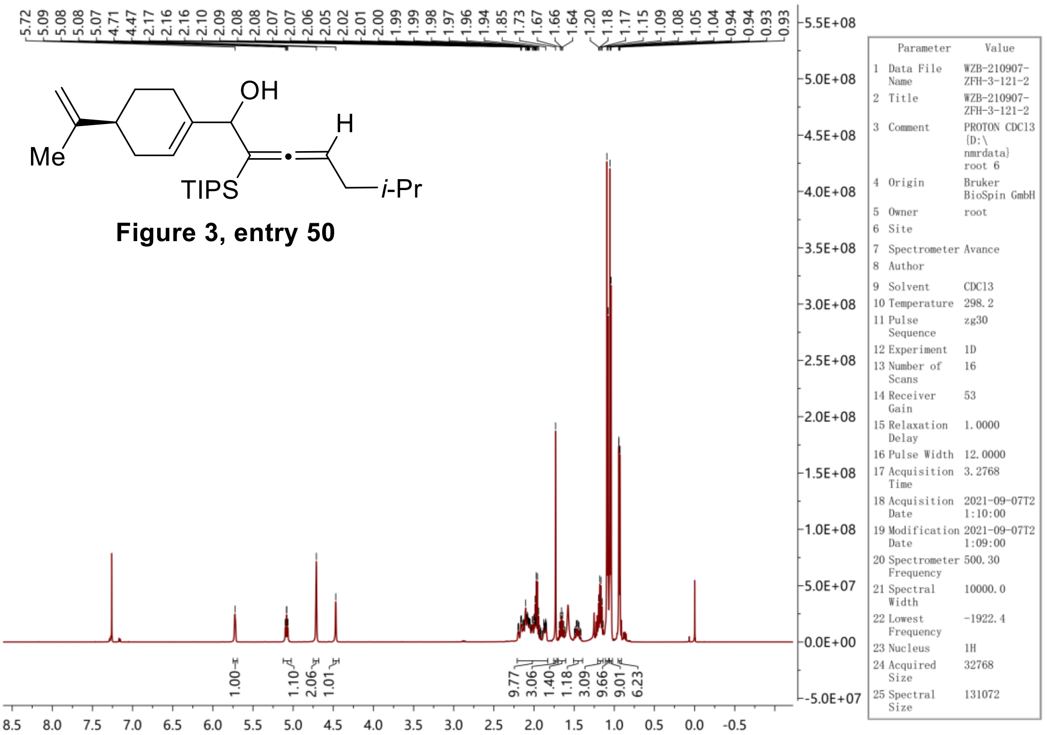

Supplementary Figure 110. ^{1}H NMR spectrum of compound **50**

^{13}C NMR (126 MHz, room temperature, $CDCl_3$)

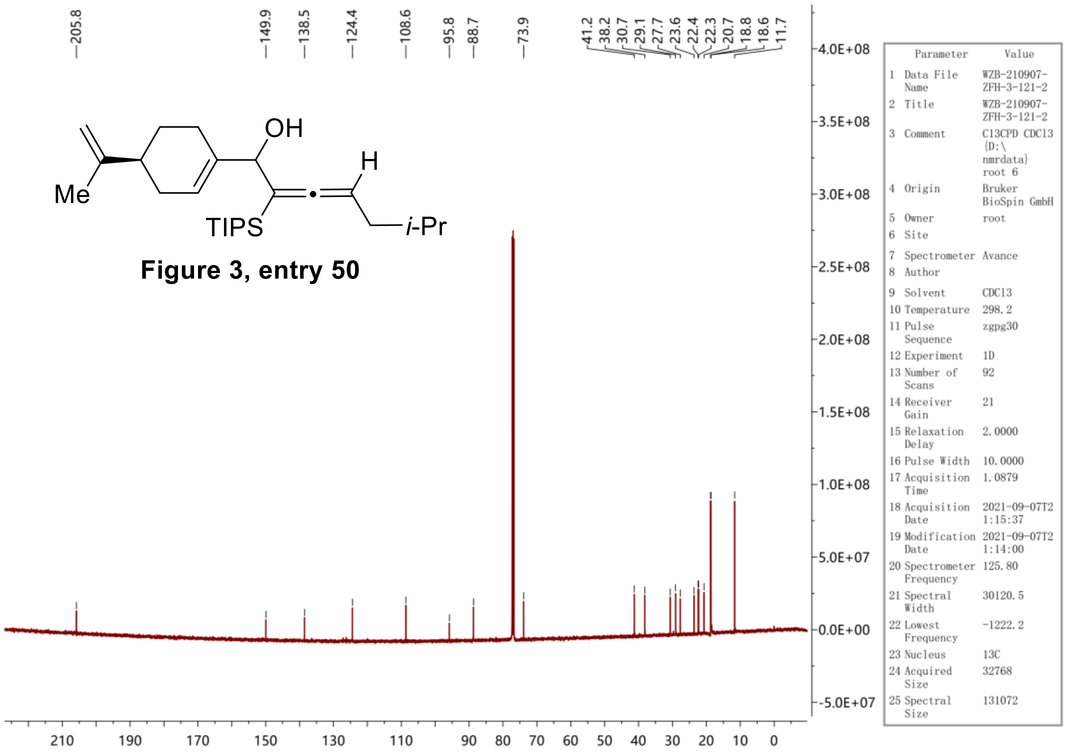

Supplementary Figure 111. ^{13}C NMR spectrum of compound **50**

^{1}H NMR (500 MHz, room temperature, $CDCl_3$)

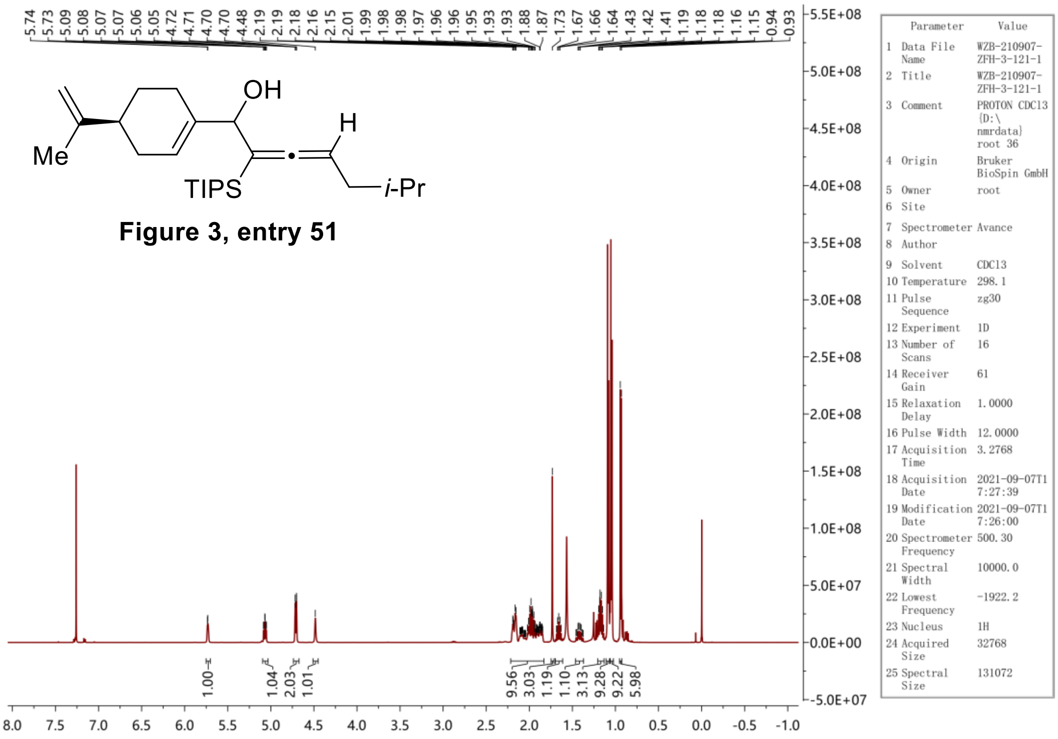

Supplementary Figure 112. ^{1}H NMR spectrum of compound **51**

^{13}C NMR (126 MHz, room temperature, $CDCl_3$)

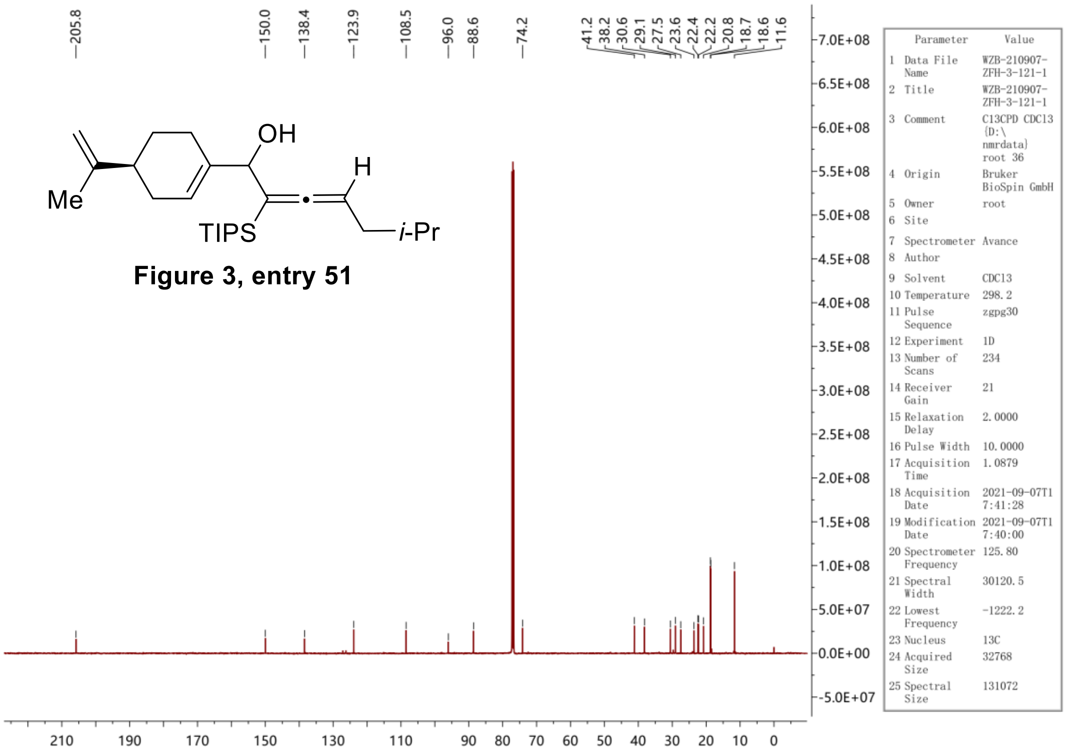

Supplementary Figure 113. ^{13}C NMR spectrum of compound **51**

^{1}H NMR (500 MHz, room temperature, $CDCl_3$)

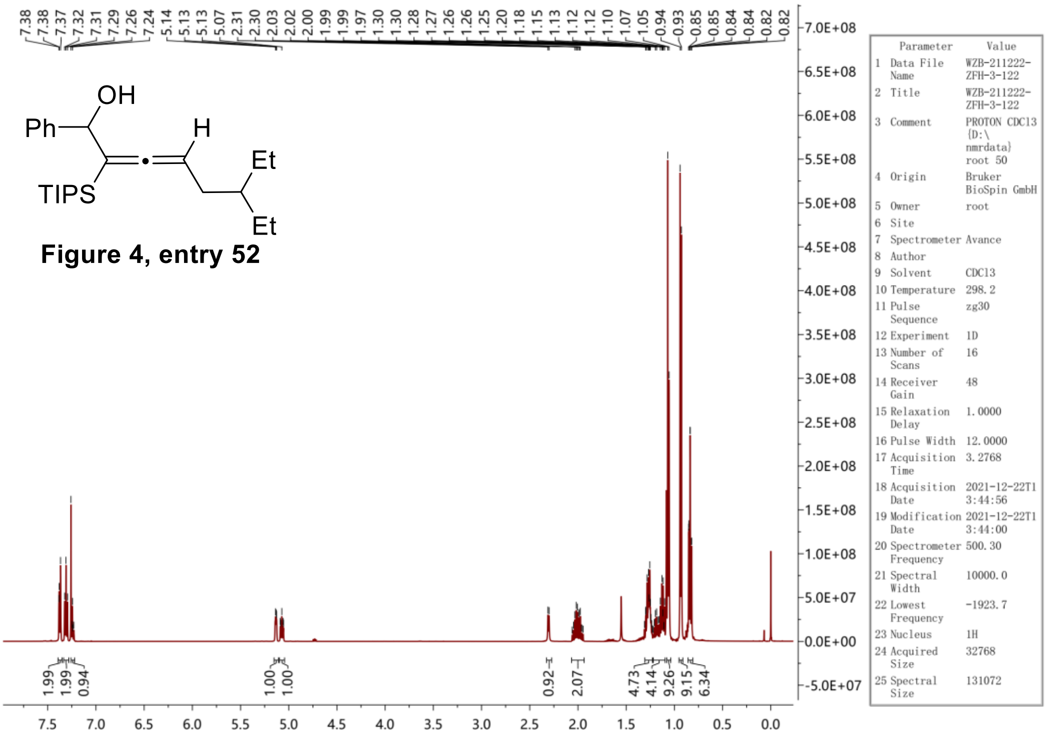

Supplementary Figure 114. ^{1}H NMR spectrum of compound **52**

^{13}C NMR (126 MHz, room temperature, $CDCl_3$)

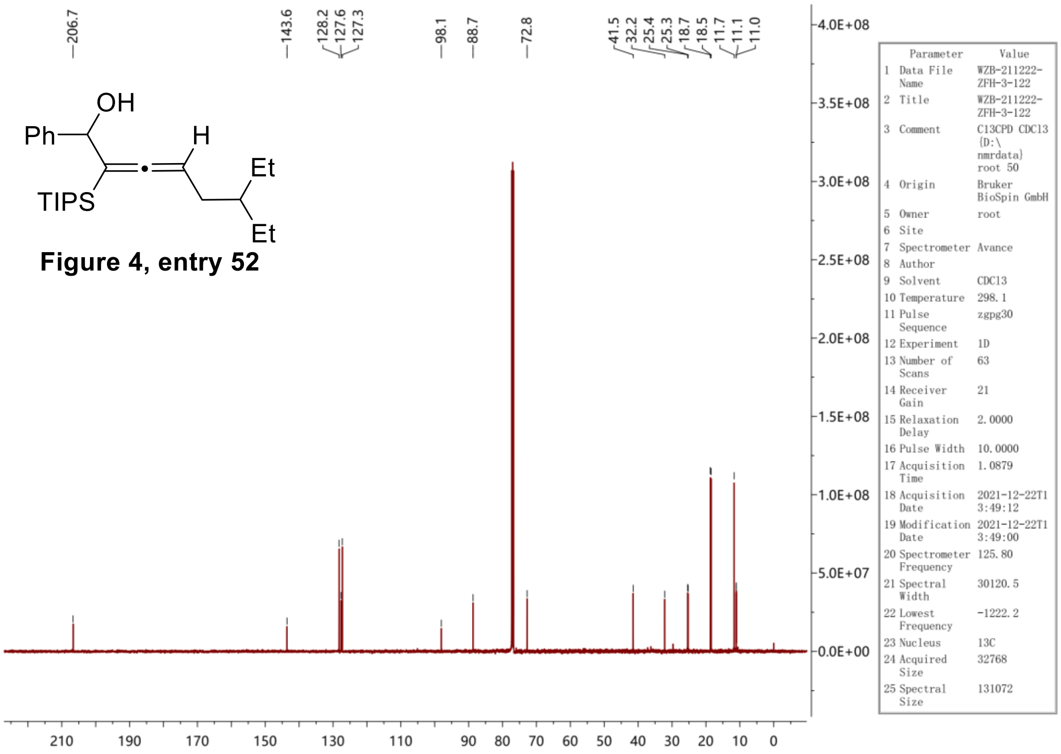

Supplementary Figure 115. ^{13}C NMR spectrum of compound **52**

^{1}H NMR (500 MHz, room temperature, $CDCl_3$)

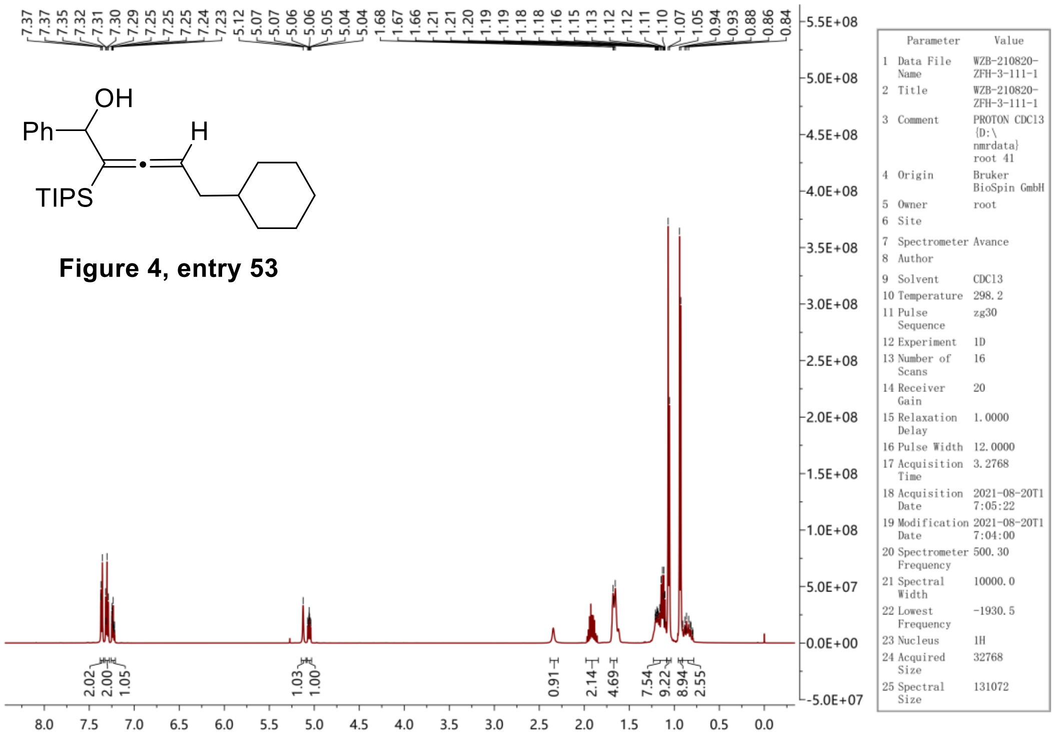

Supplementary Figure 116. ^{1}H NMR spectrum of compound **53**

^{13}C NMR (126 MHz, room temperature, $CDCl_3$)

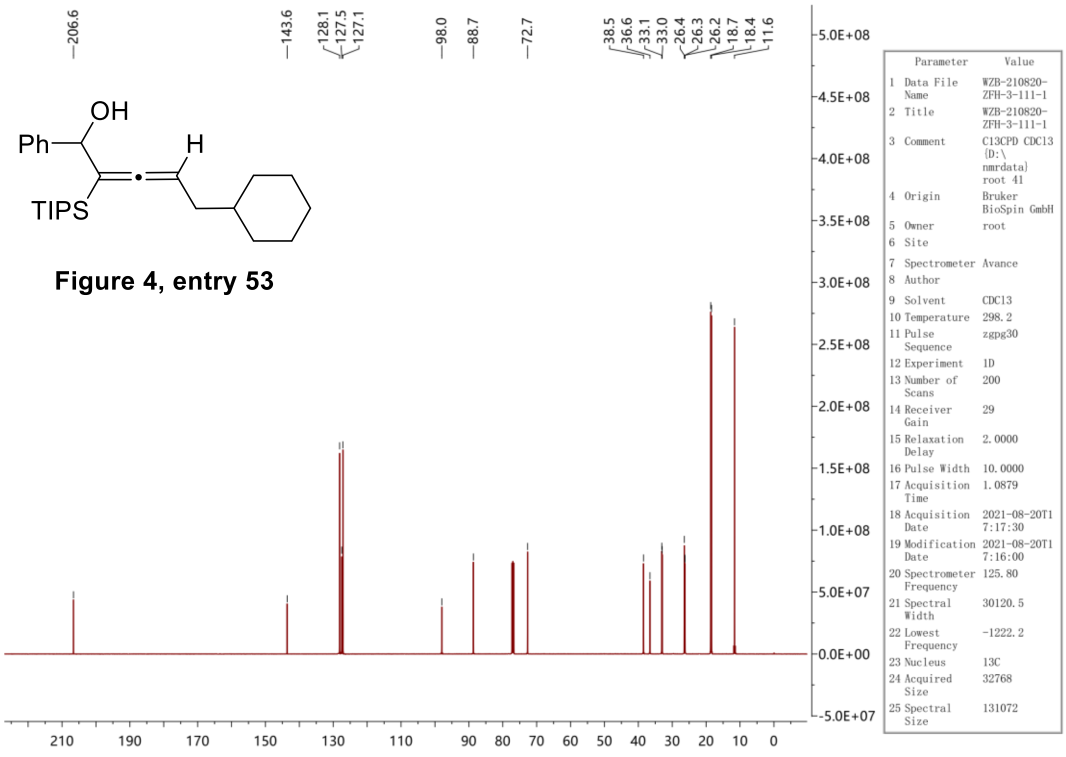

Supplementary Figure 117. ^{13}C NMR spectrum of compound **53**

^{1}H NMR (500 MHz, room temperature, $CDCl_3$)

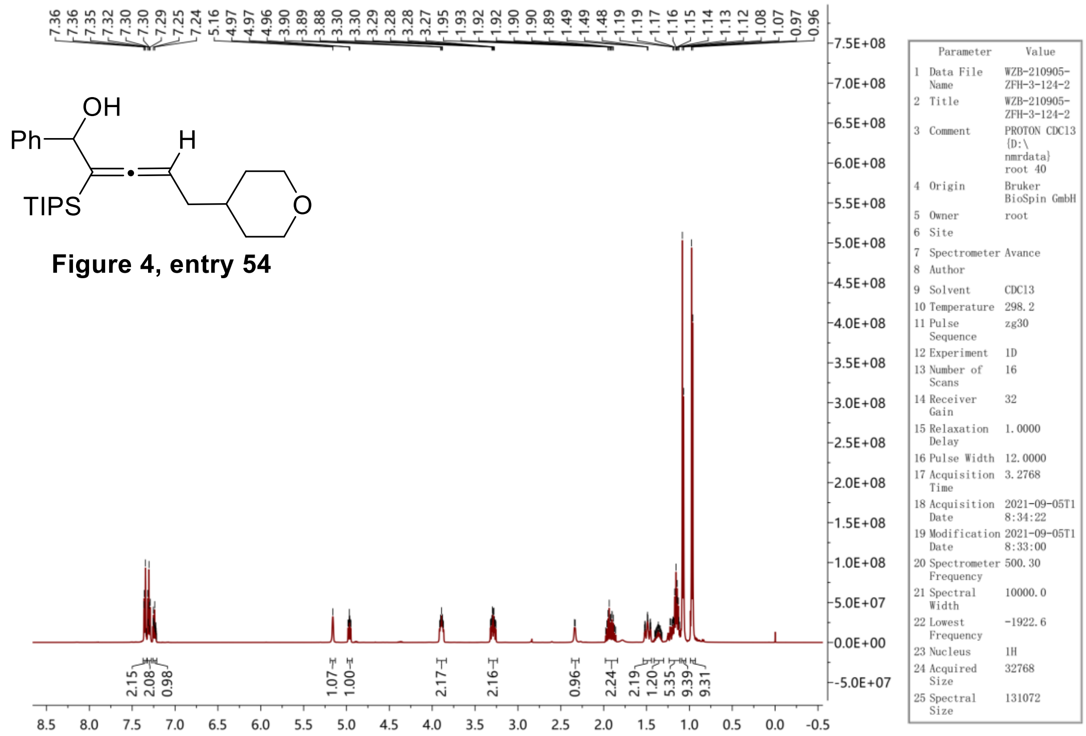

Supplementary Figure 118. ^{1}H NMR spectrum of compound **54**

^{13}C NMR (126 MHz, room temperature, $CDCl_3$)

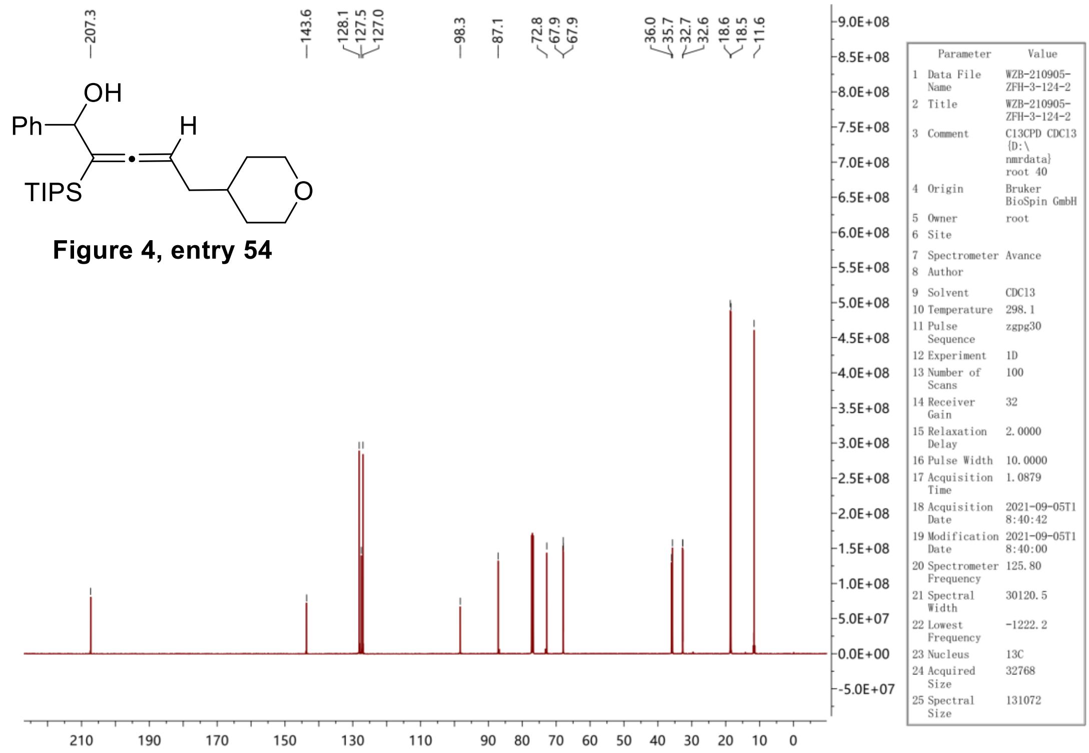

Supplementary Figure 119. ^{13}C NMR spectrum of compound **54**

^{1}H NMR (500 MHz, room temperature, $CDCl_3$)

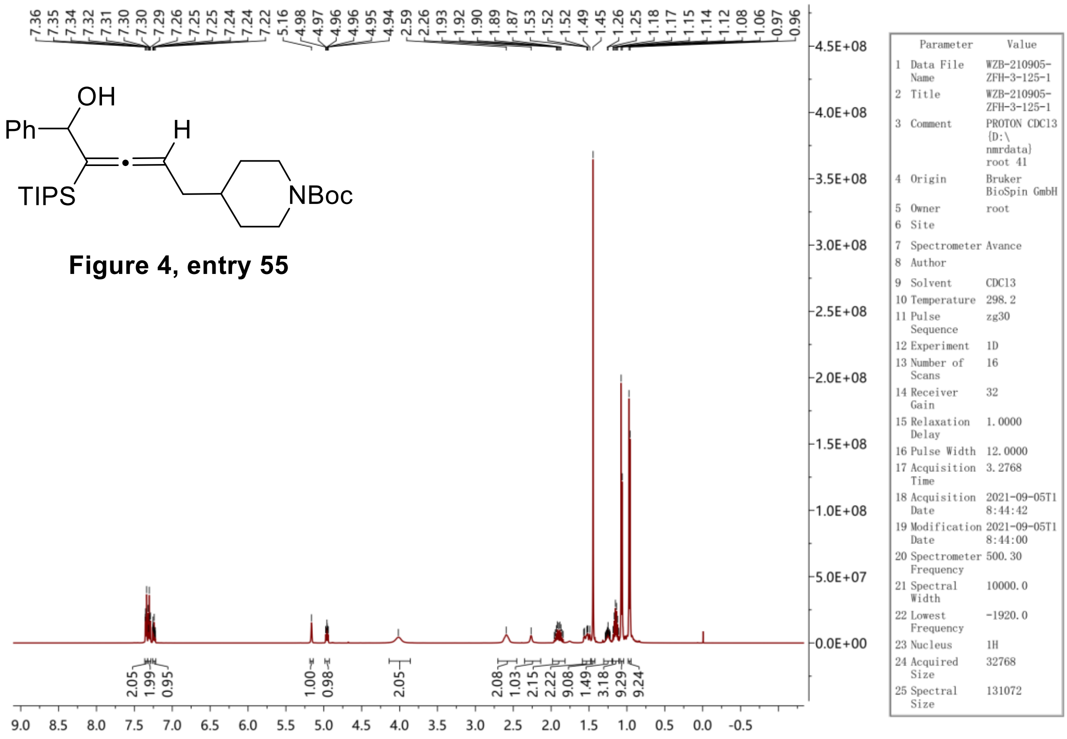

Supplementary Figure 120. ^{1}H NMR spectrum of compound **55**

^{13}C NMR (126 MHz, room temperature, $CDCl_3$)

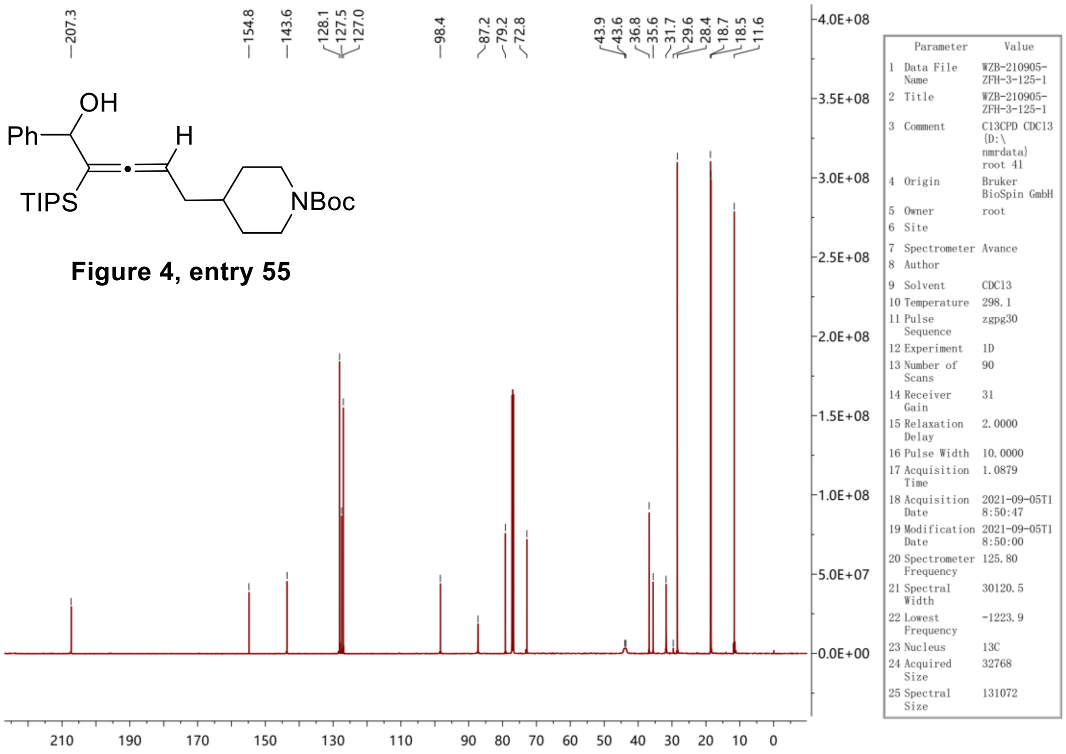

Supplementary Figure 121. ^{13}C NMR spectrum of compound **55**

^{1}H NMR (500 MHz, room temperature, $CDCl_3$)

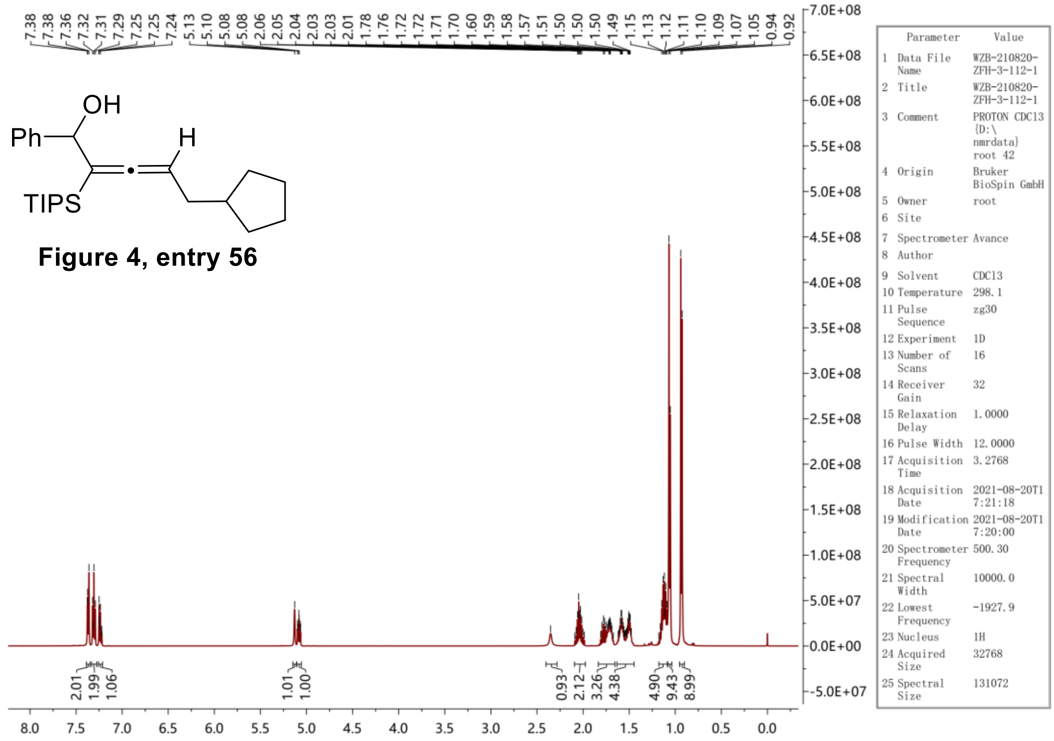

Supplementary Figure 122. ^{1}H NMR spectrum of compound **56**

^{13}C NMR (126 MHz, room temperature, $CDCl_3$)

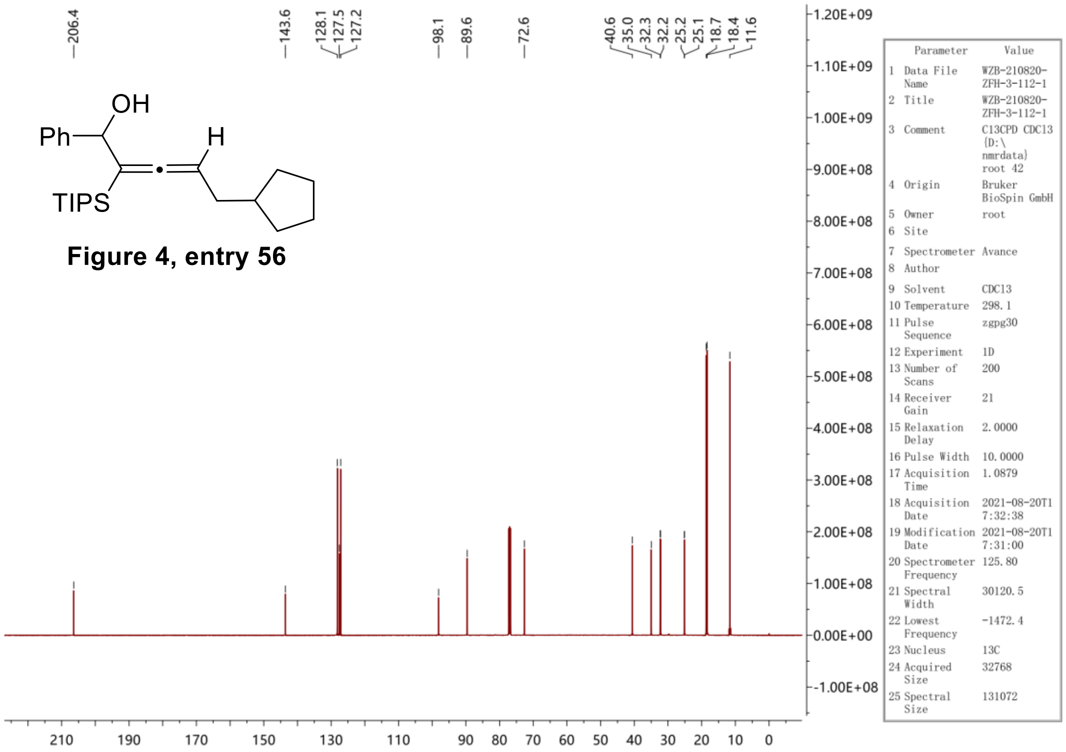

Supplementary Figure 123. ^{13}C NMR spectrum of compound **56**

^{1}H NMR (500 MHz, room temperature, $CDCl_3$)

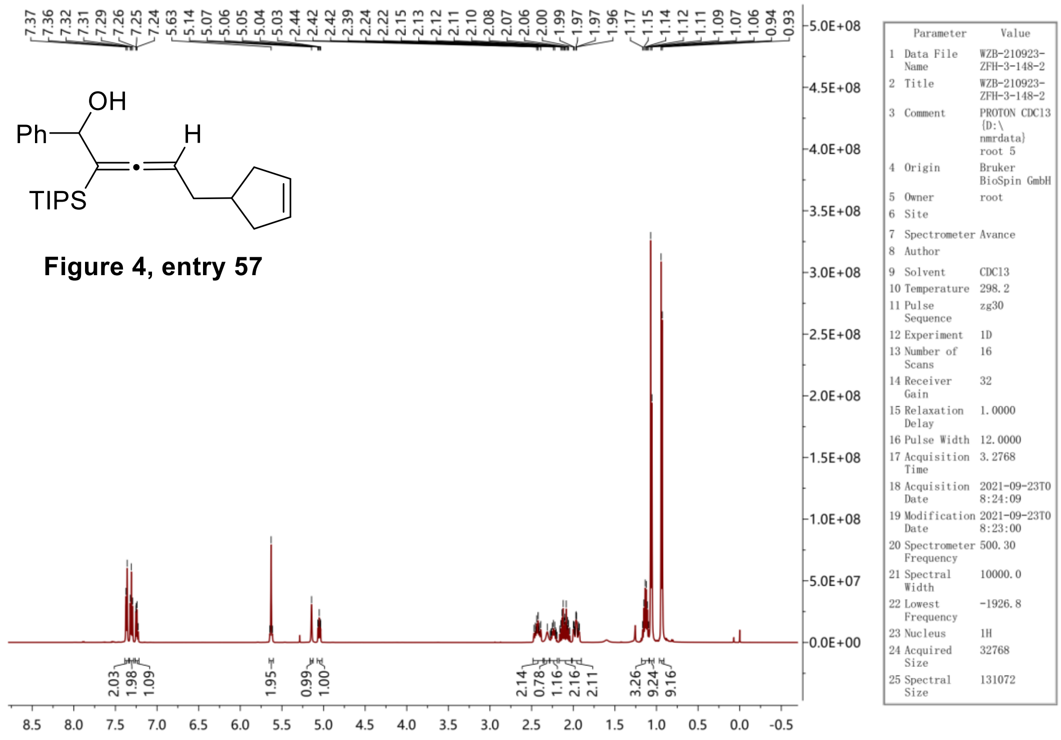

Supplementary Figure 124. ^{1}H NMR spectrum of compound **57**

^{13}C NMR (126 MHz, room temperature, $CDCl_3$)

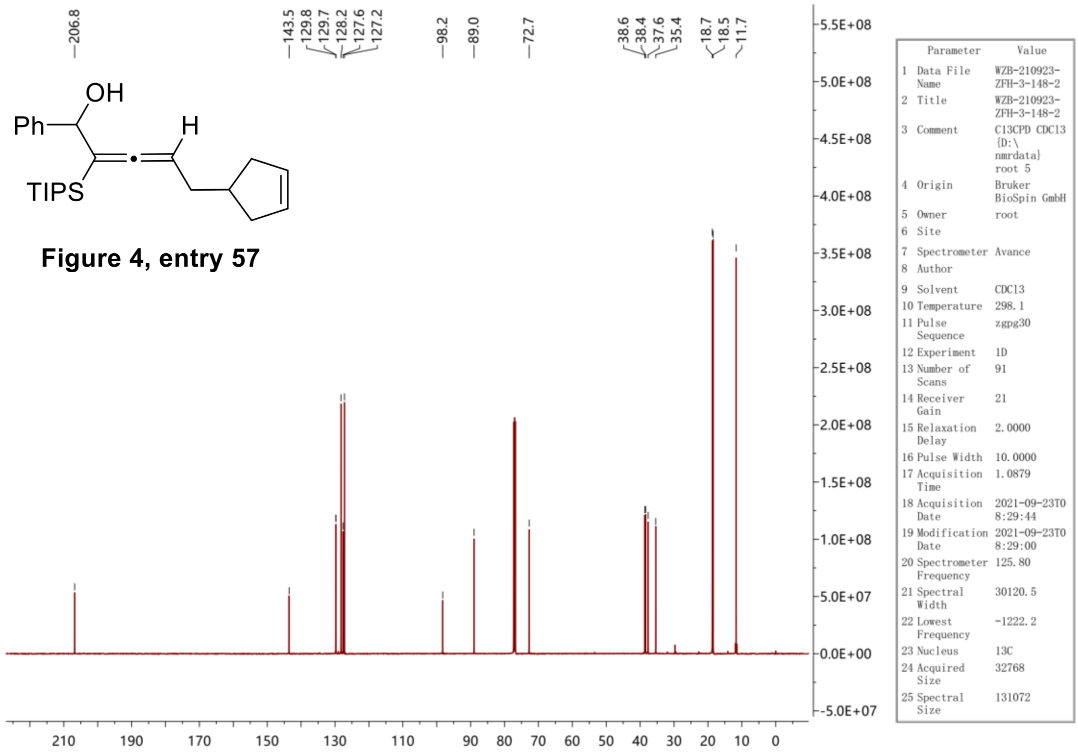

Supplementary Figure 125. ^{13}C NMR spectrum of compound **57**

^{1}H NMR (500 MHz, room temperature, $CDCl_3$)

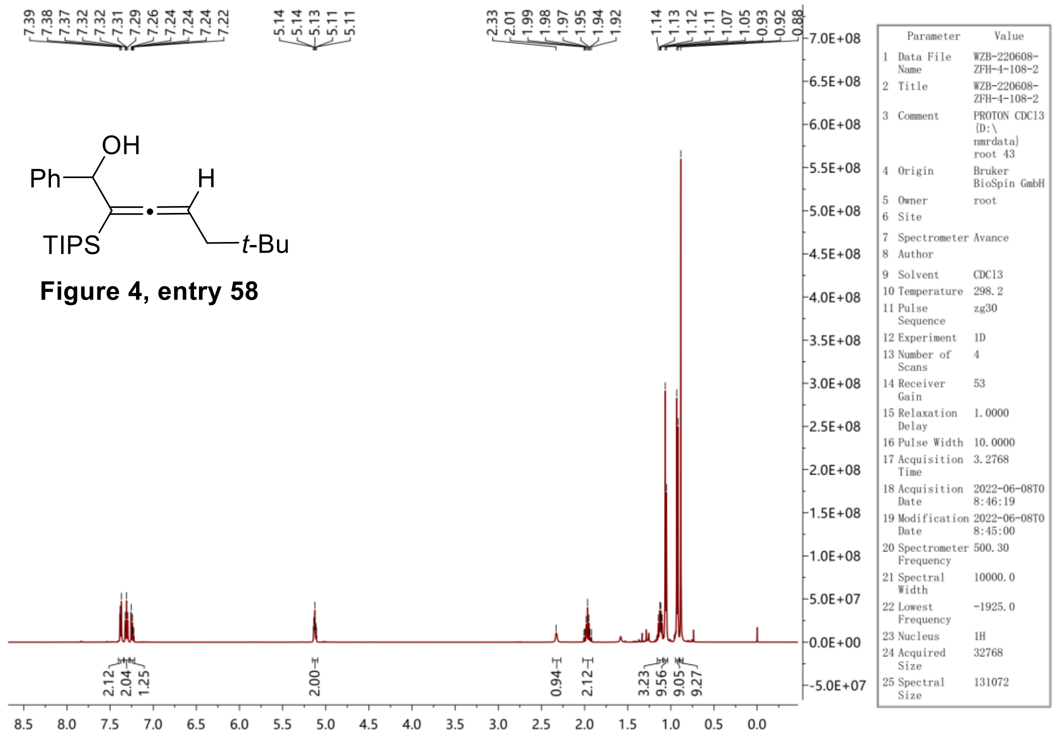

Supplementary Figure 126. ^{1}H NMR spectrum of compound **58**

^{13}C NMR (126 MHz, room temperature, $CDCl_3$)

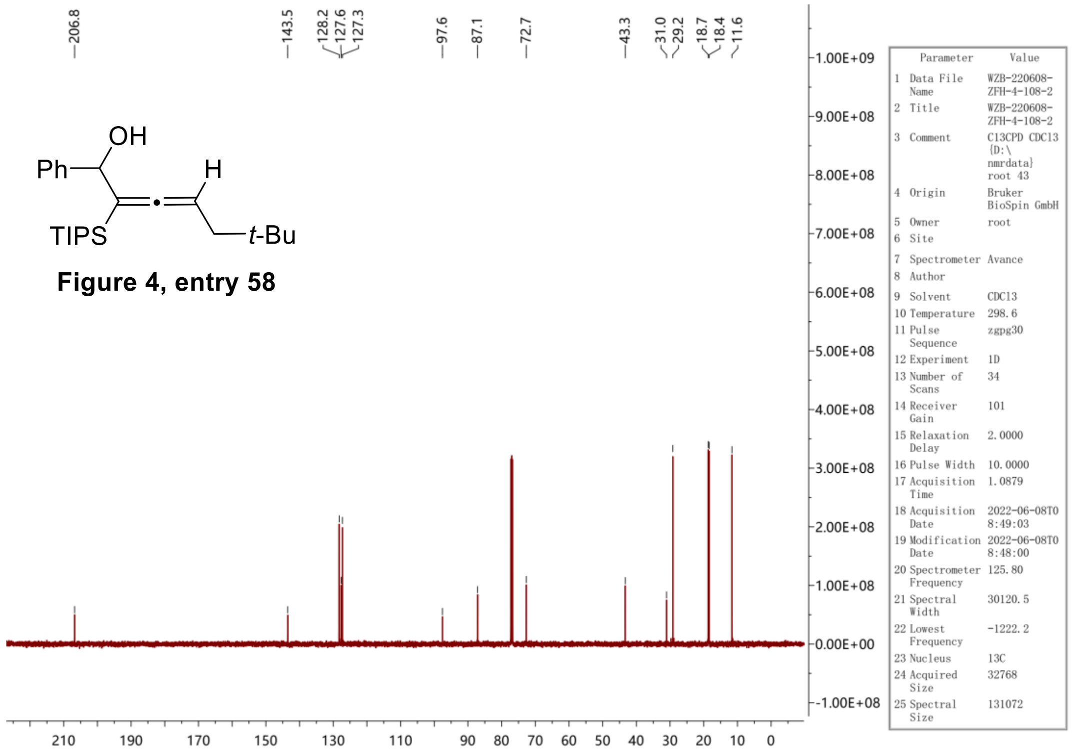

Supplementary Figure 127. ^{13}C NMR spectrum of compound **58**

^{1}H NMR (500 MHz, room temperature, $CDCl_3$)

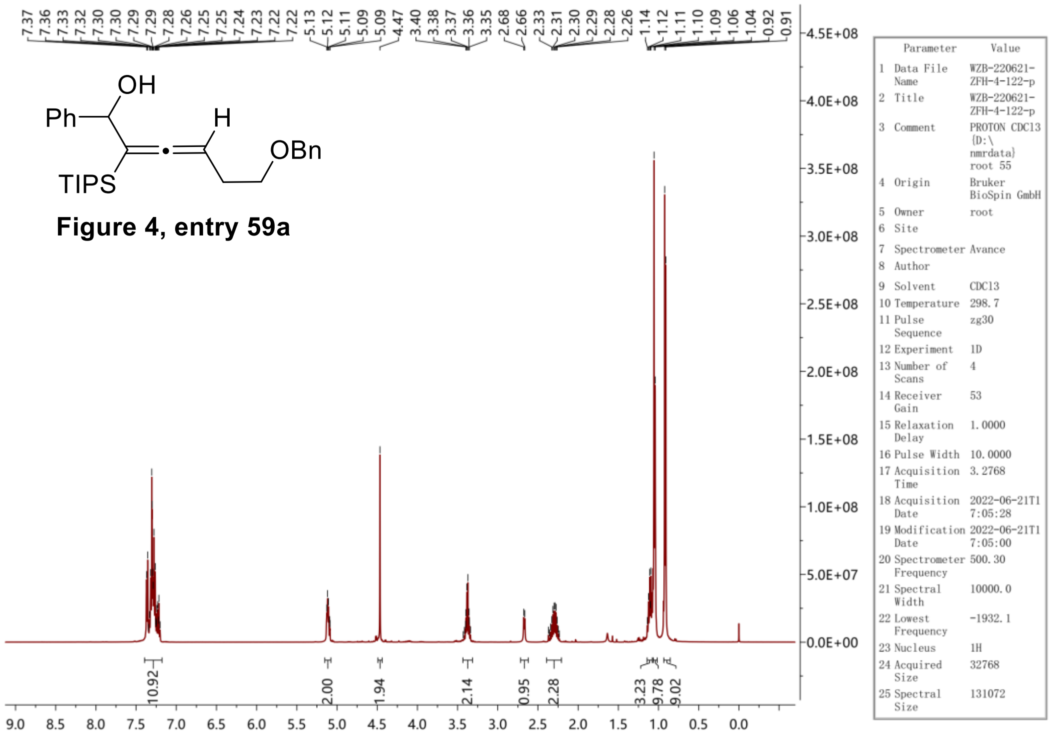

Supplementary Figure 128. ^{1}H NMR spectrum of compound **59a**

^{13}C NMR (126 MHz, room temperature, $CDCl_3$)

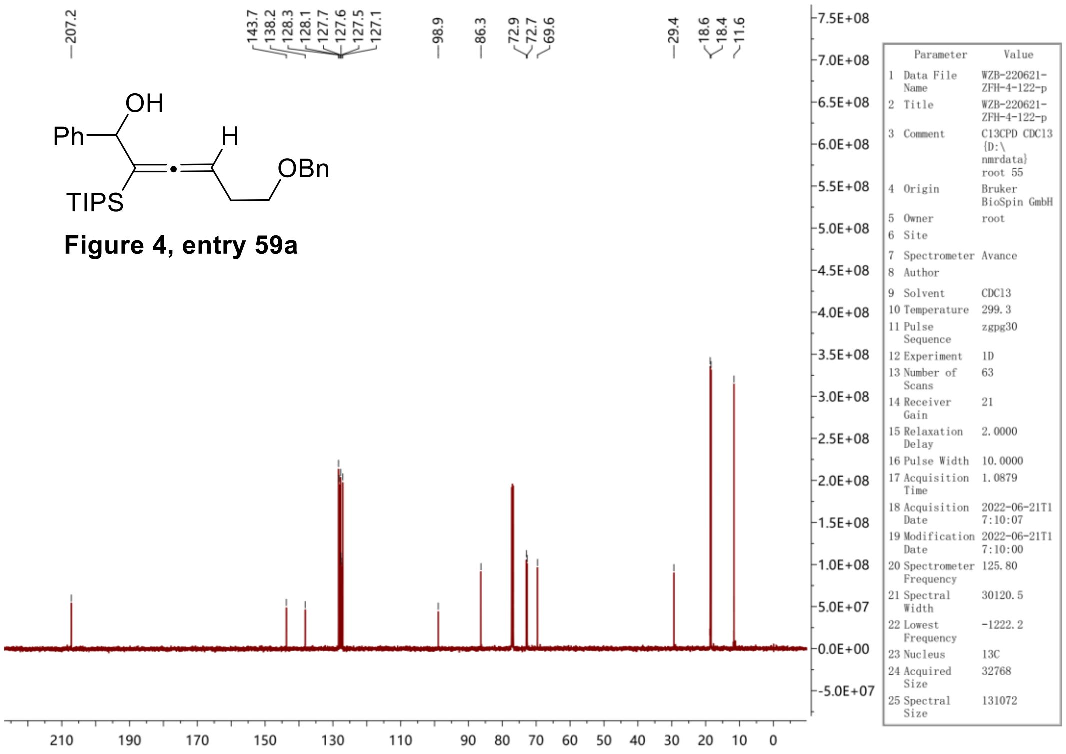

Supplementary Figure 129. ^{13}C NMR spectrum of compound **59a**

^{1}H NMR (500 MHz, room temperature, $CDCl_3$)

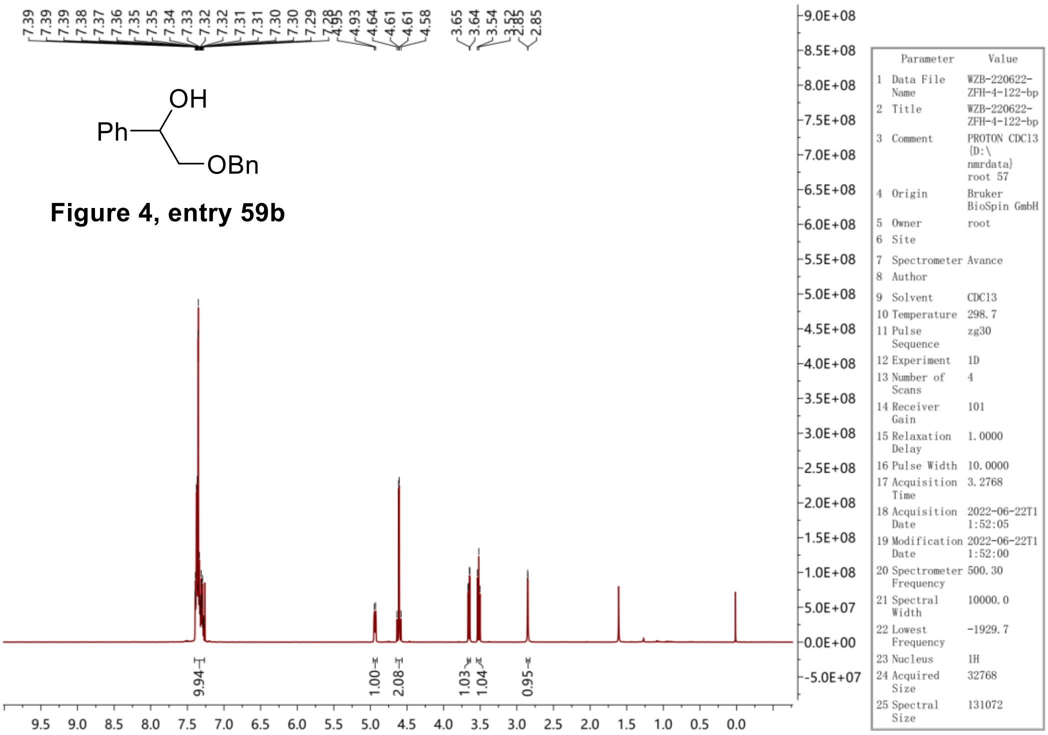

Supplementary Figure 130. ^{1}H NMR spectrum of compound **59a**

^{13}C NMR (126 MHz, room temperature, $CDCl_3$)

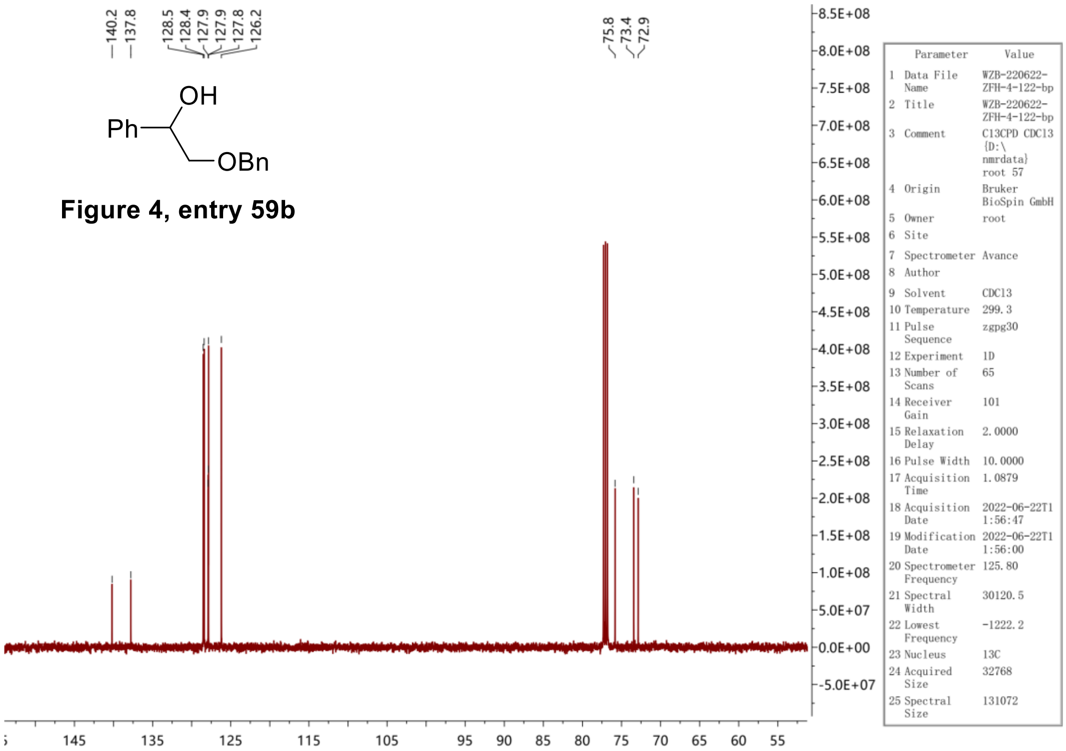

Supplementary Figure 131. ^{13}C NMR spectrum of compound **59b**

^{1}H NMR (500 MHz, room temperature, $CDCl_3$)

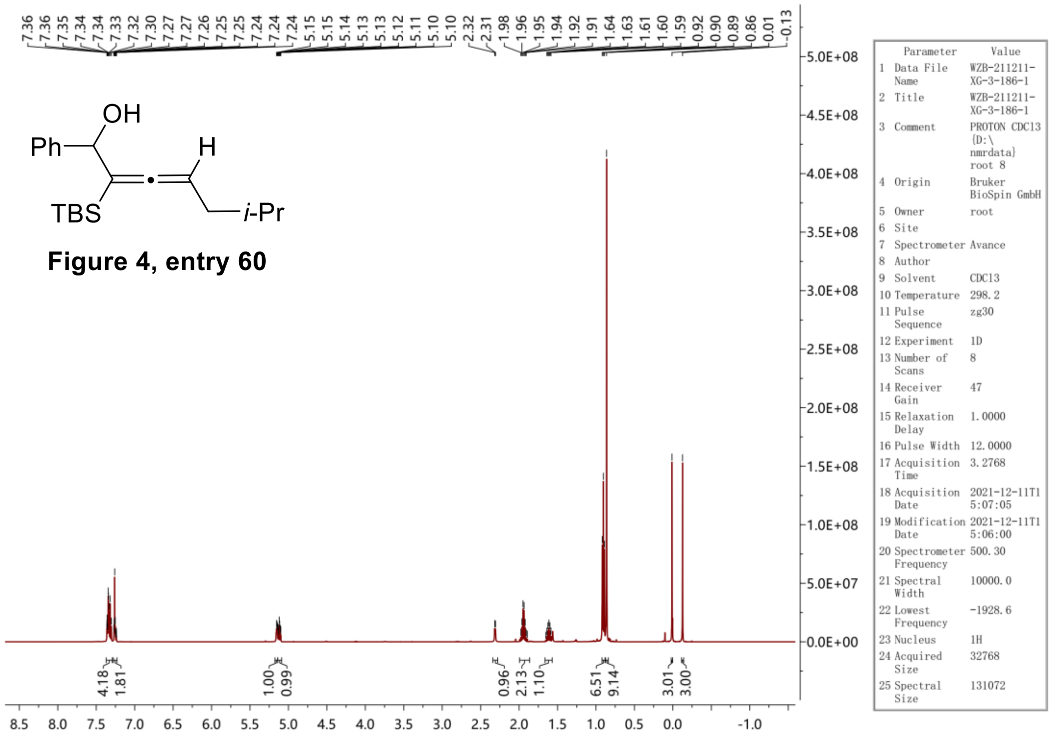

Supplementary Figure 132. ^{1}H NMR spectrum of compound **60**

^{13}C NMR (126 MHz, room temperature, $CDCl_3$)

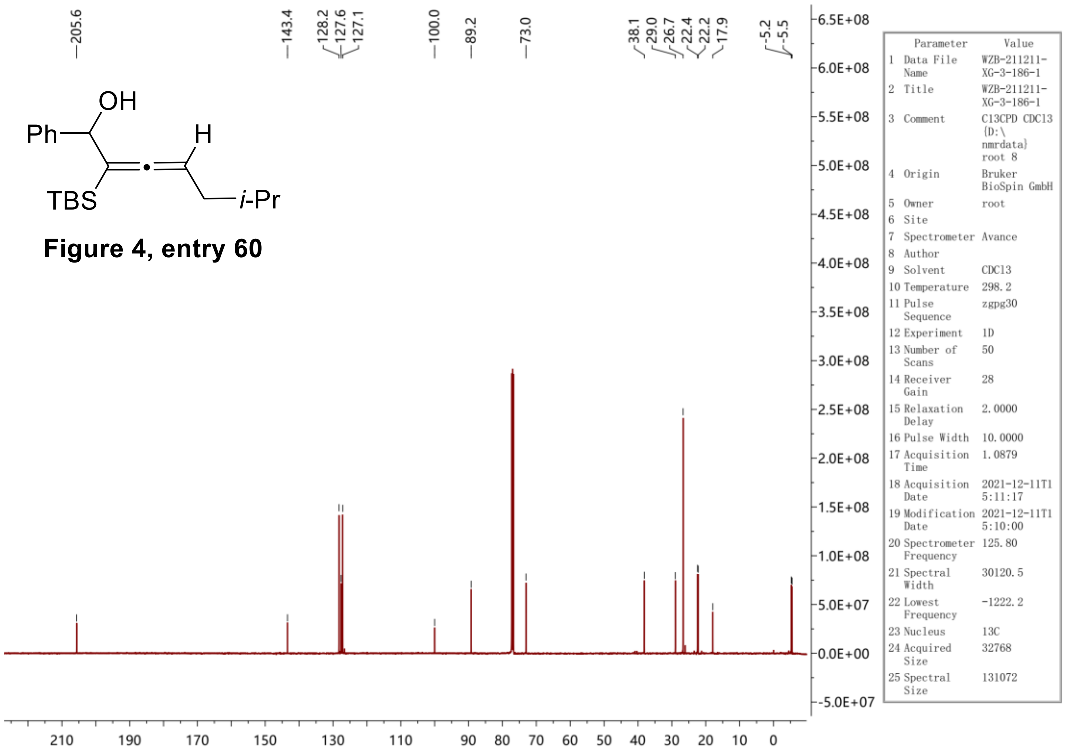

Supplementary Figure 133. ^{13}C NMR spectrum of compound **60**

^{1}H NMR (500 MHz, room temperature, $CDCl_3$)

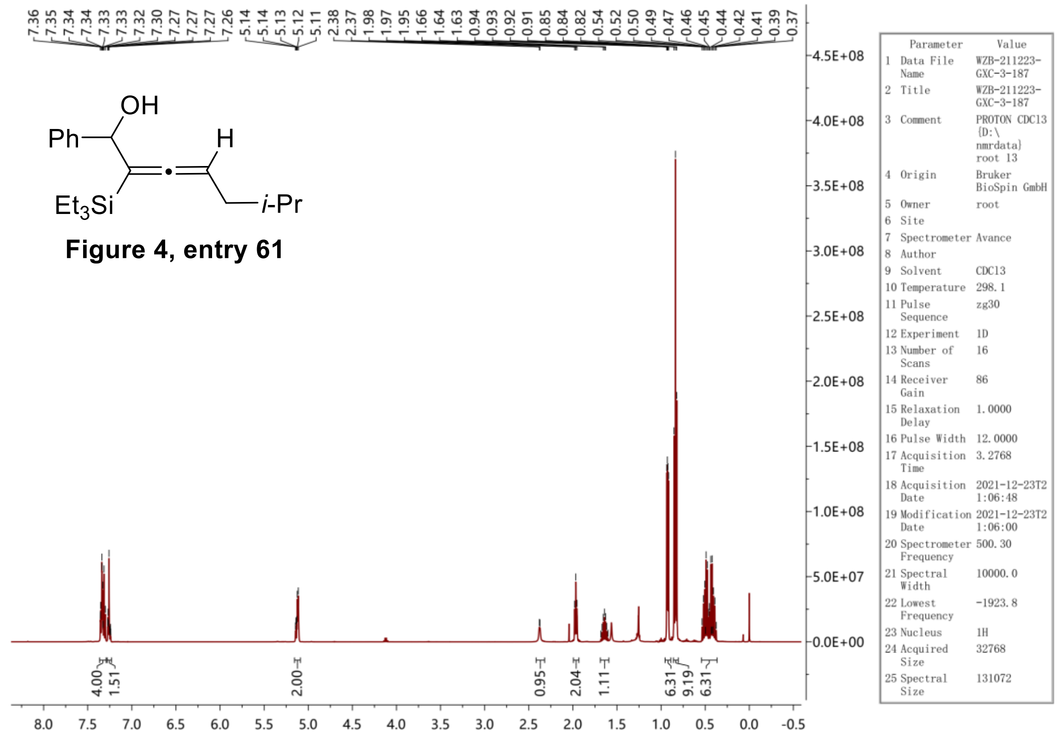

Supplementary Figure 134. ^{1}H NMR spectrum of compound **61**

^{13}C NMR (126 MHz, room temperature, $CDCl_3$)

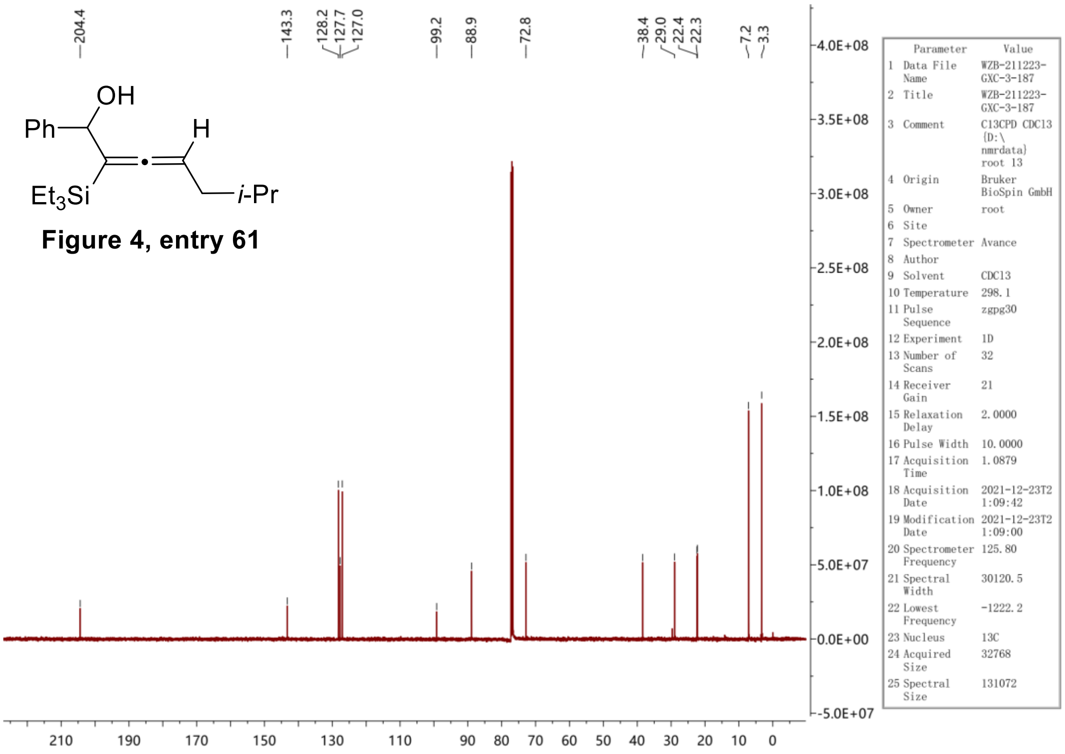

Supplementary Figure 135. ^{13}C NMR spectrum of compound **61**

^{1}H NMR (500 MHz, room temperature, $CDCl_3$)

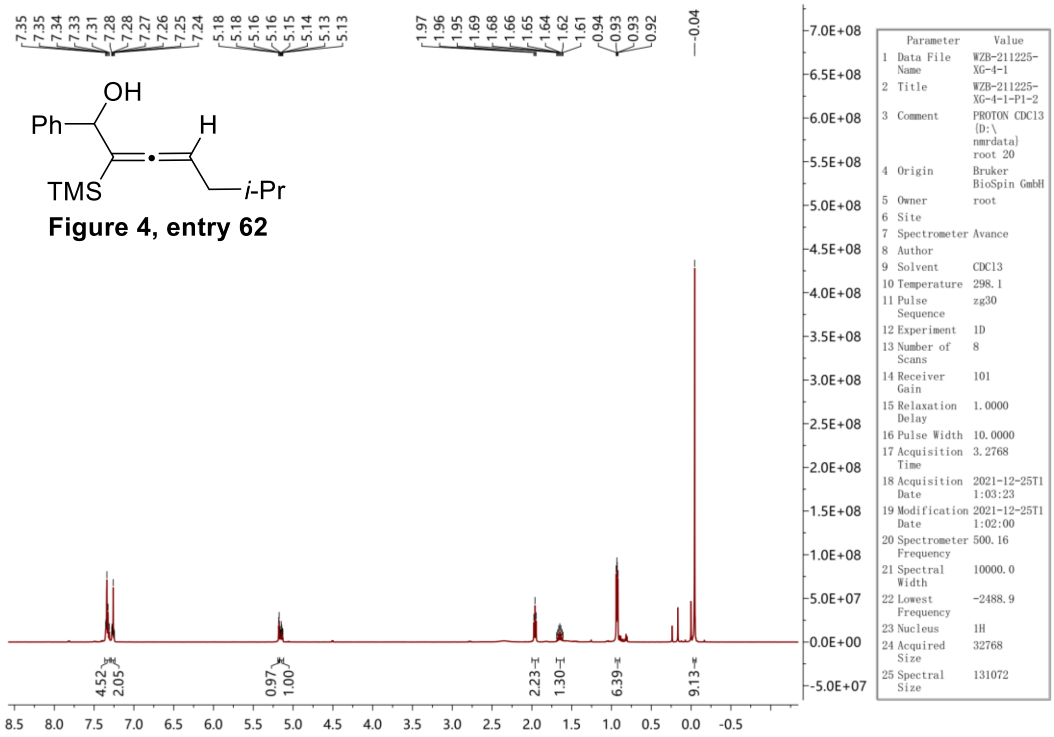

Supplementary Figure 136. ^{1}H NMR spectrum of compound **62**

^{13}C NMR (126 MHz, room temperature, $CDCl_3$)

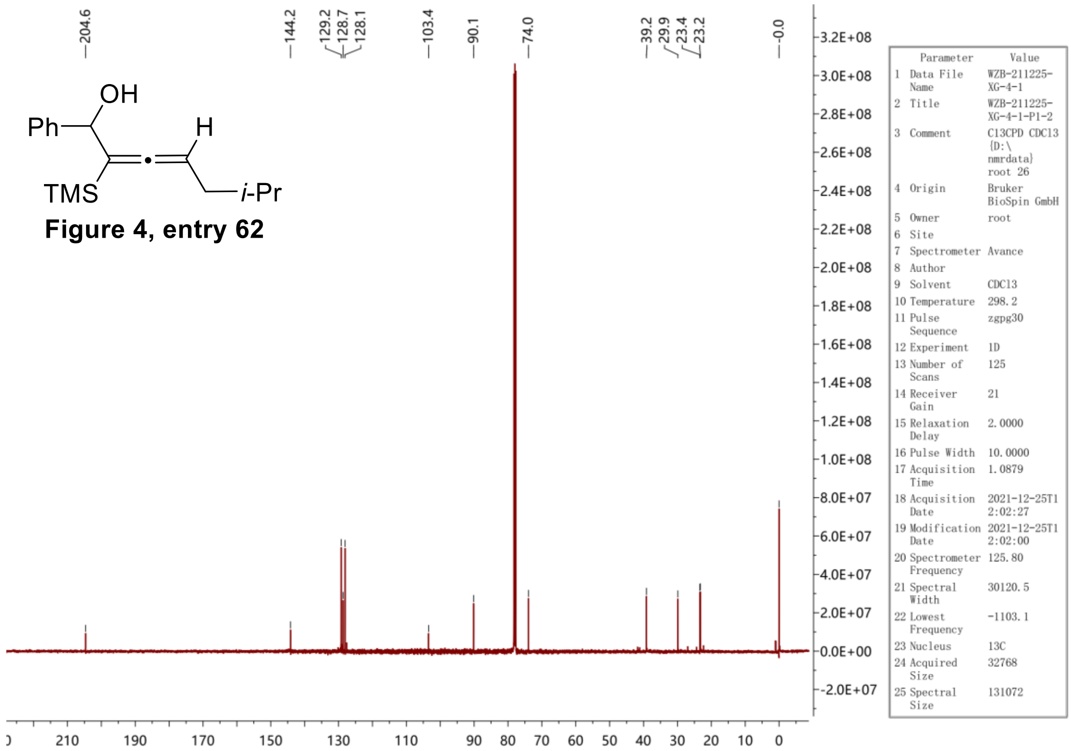

Supplementary Figure 137. ^{13}C NMR spectrum of compound **62**

^{1}H NMR (500 MHz, room temperature, $CDCl_3$)

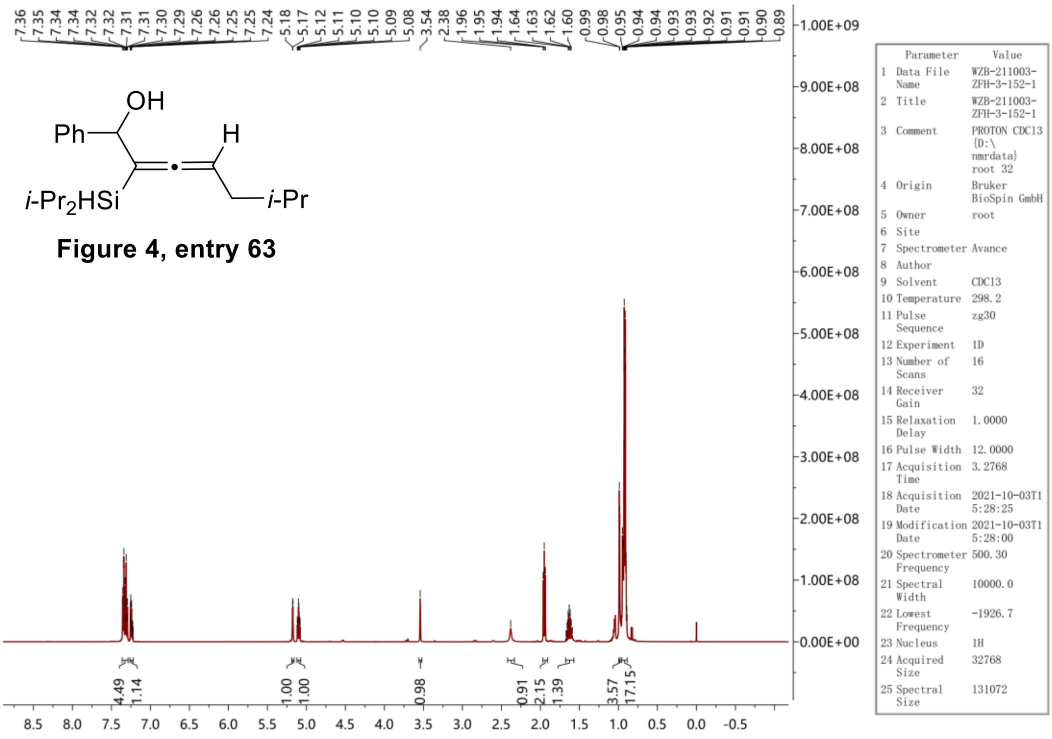

Supplementary Figure 138. ^{1}H NMR spectrum of compound **63**

^{13}C NMR (126 MHz, room temperature, $CDCl_3$)

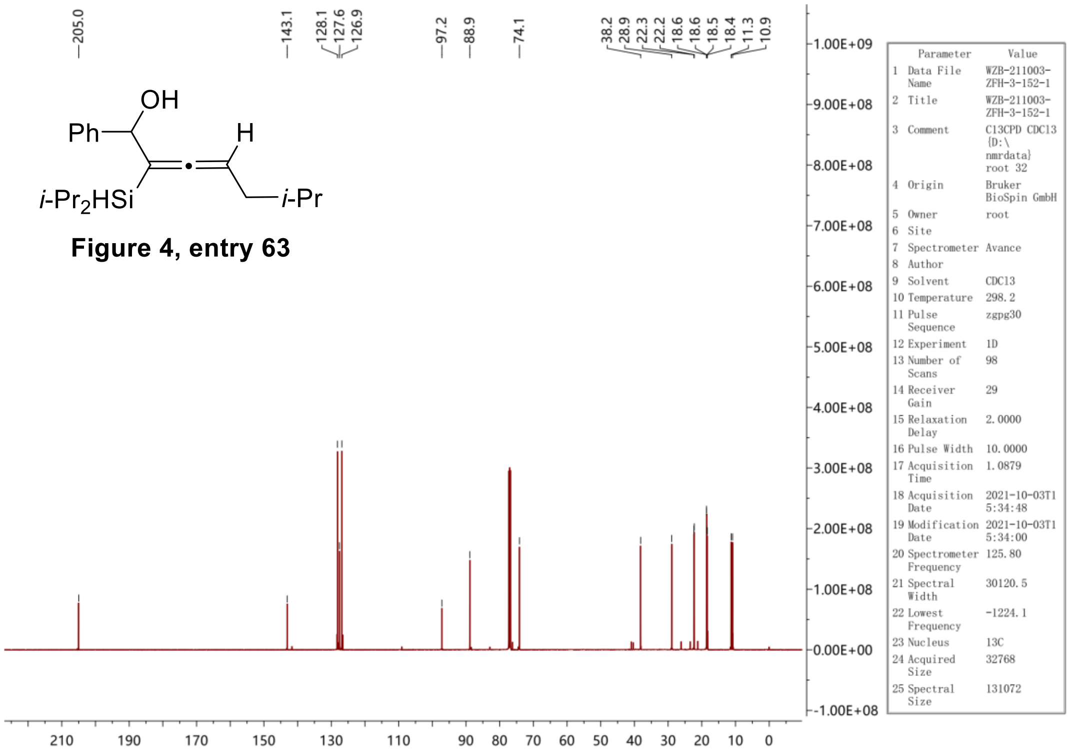

Supplementary Figure 139. ^{13}C NMR spectrum of compound **63**

^{1}H NMR (500 MHz, room temperature, $CDCl_3$)

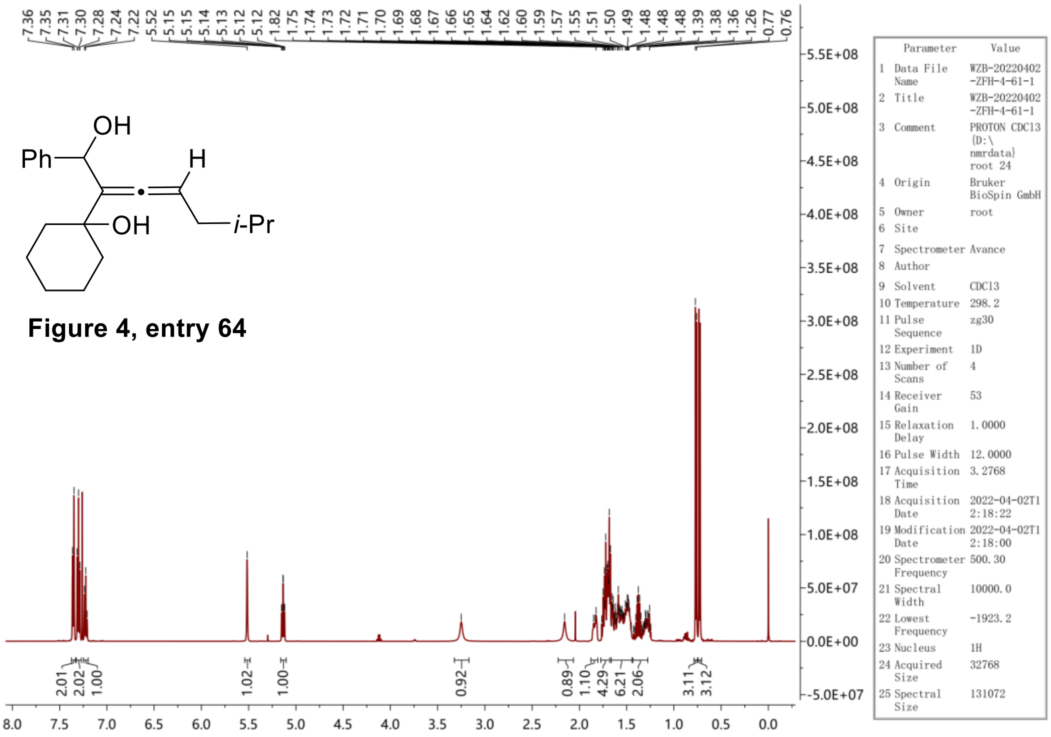

Supplementary Figure 140. ^{1}H NMR spectrum of compound **64**

^{13}C NMR (126 MHz, room temperature, $CDCl_3$)

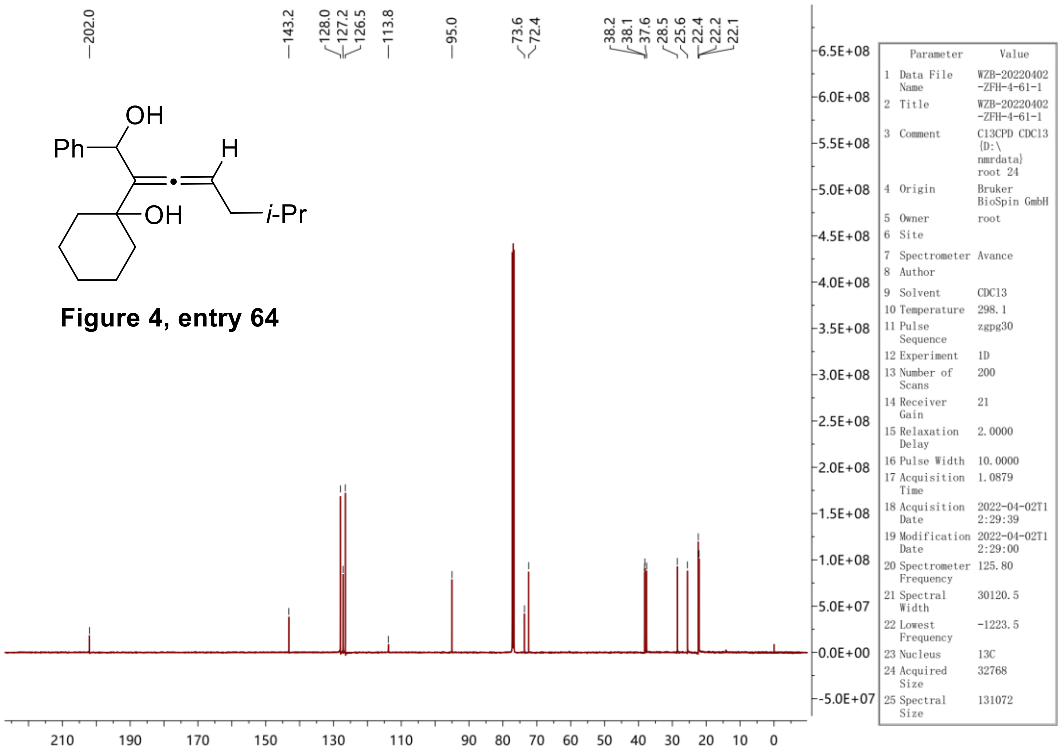

Supplementary Figure 141. ^{13}C NMR spectrum of compound **64**

^{1}H NMR (500 MHz, room temperature, $CDCl_3$)

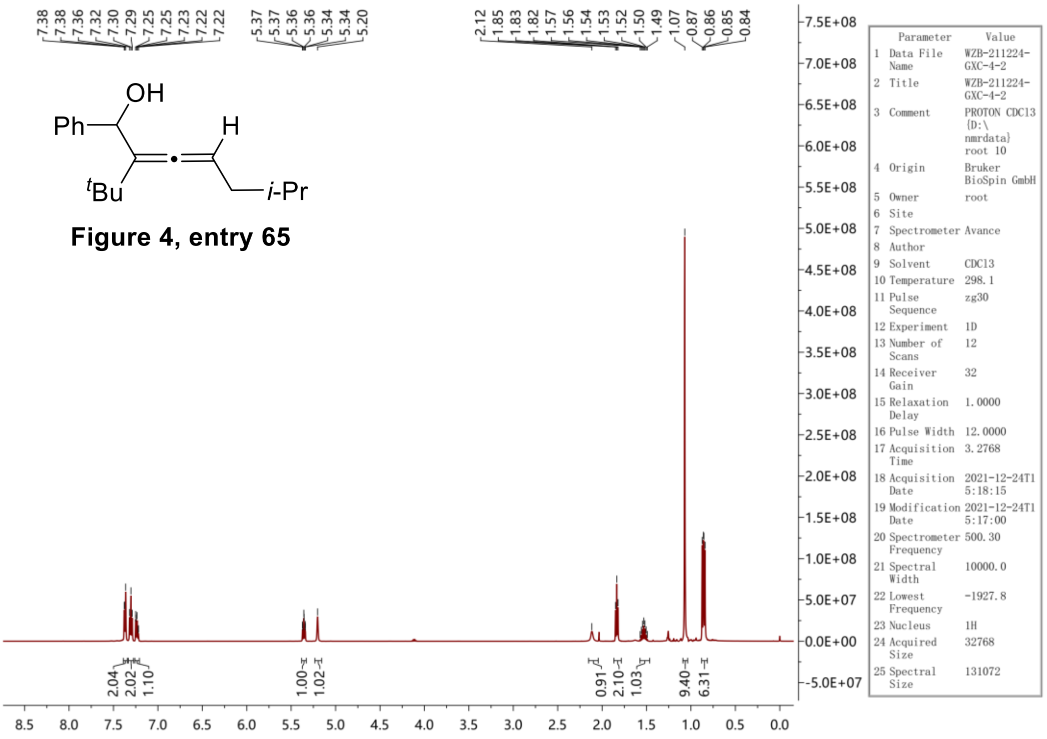

Supplementary Figure 142. ^{1}H NMR spectrum of compound **65**

^{13}C NMR (126 MHz, room temperature, $CDCl_3$)

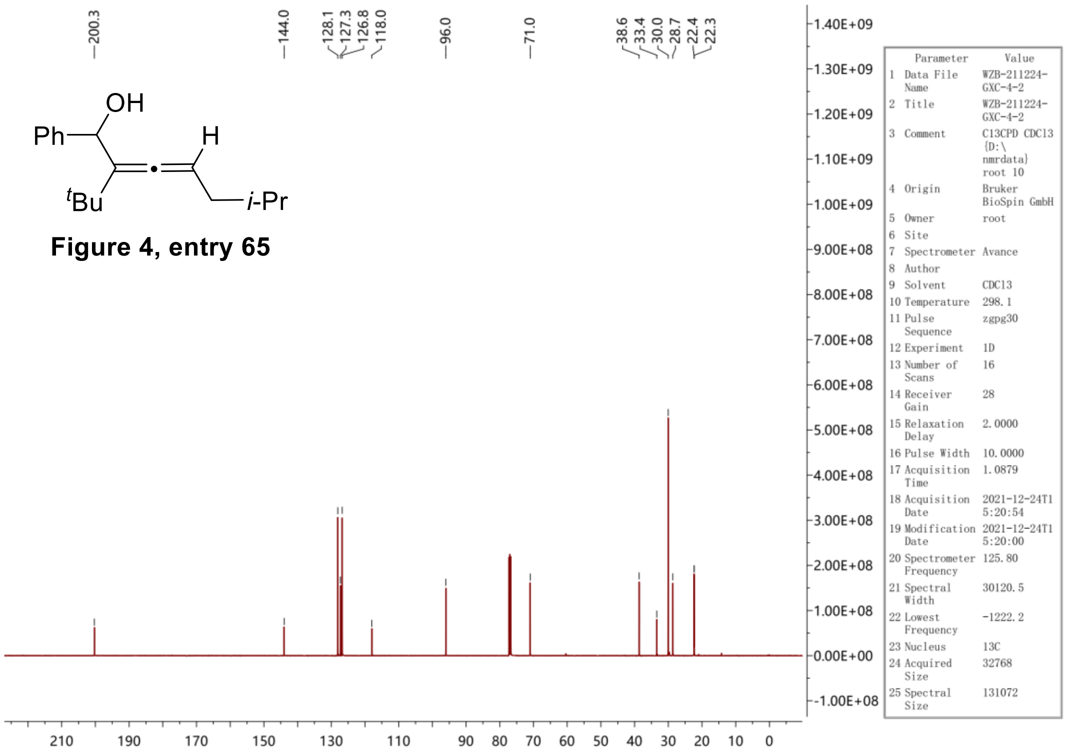

Supplementary Figure 143. ^{13}C NMR spectrum of compound **65**

^{1}H NMR (500 MHz, room temperature, $CDCl_3$)

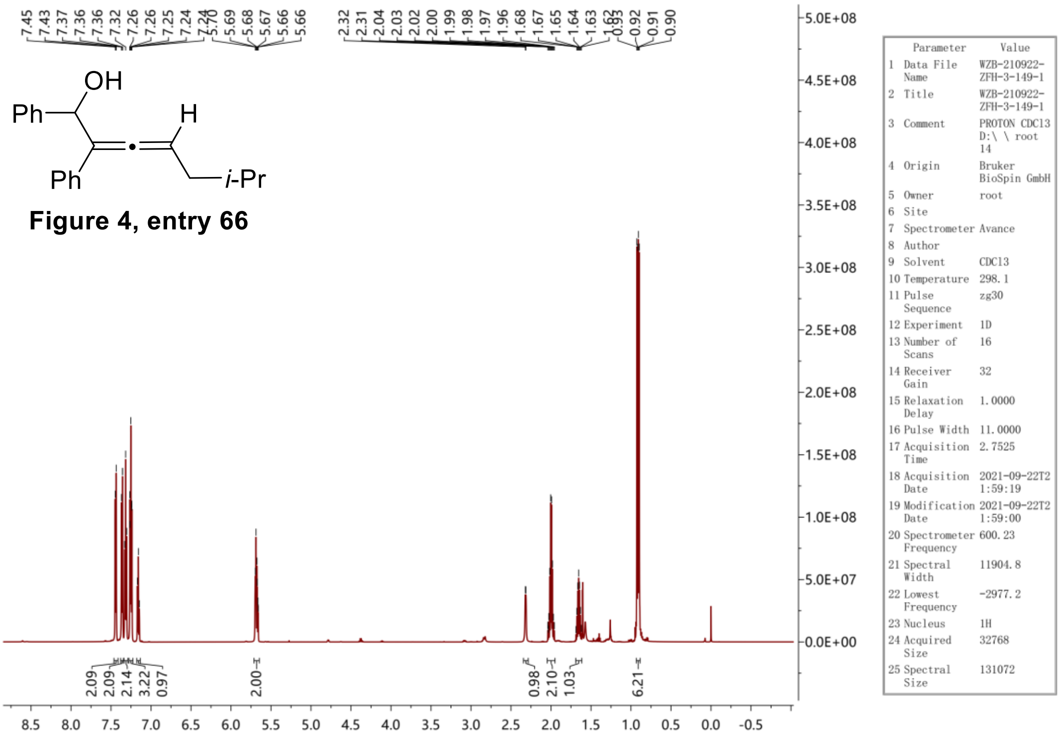

Supplementary Figure 144. ^{1}H NMR spectrum of compound **66**

^{13}C NMR (126 MHz, room temperature, $CDCl_3$)

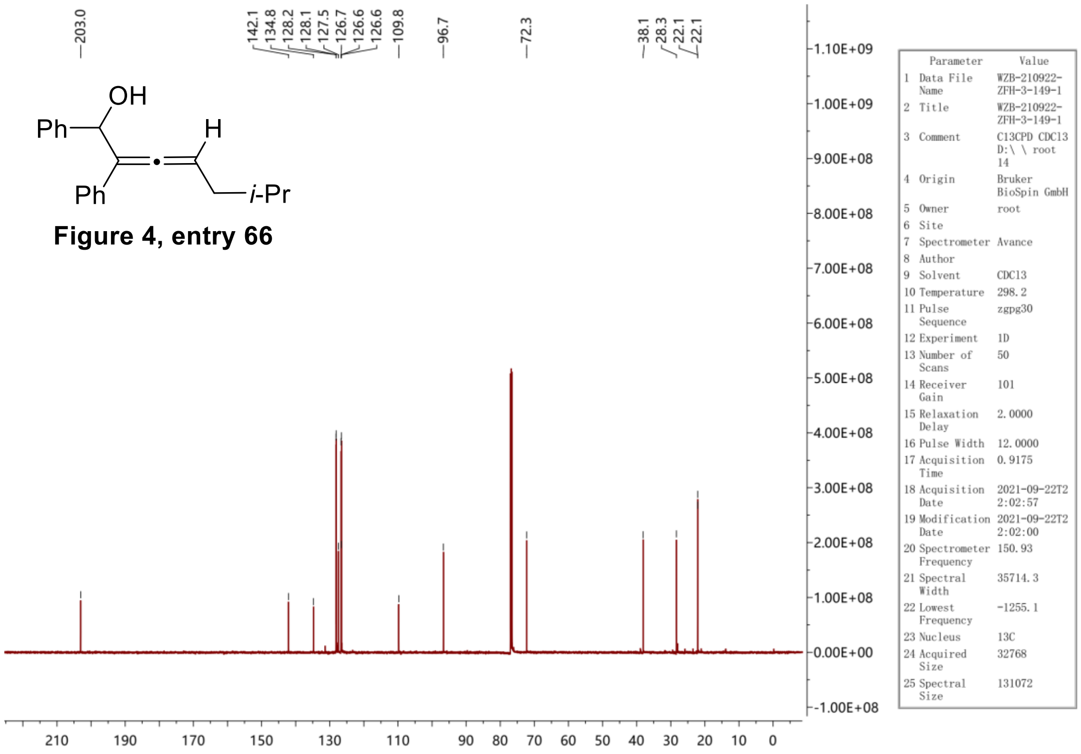

Supplementary Figure 145. ^{13}C NMR spectrum of compound **66**

^{1}H NMR (500 MHz, room temperature, $CDCl_3$)

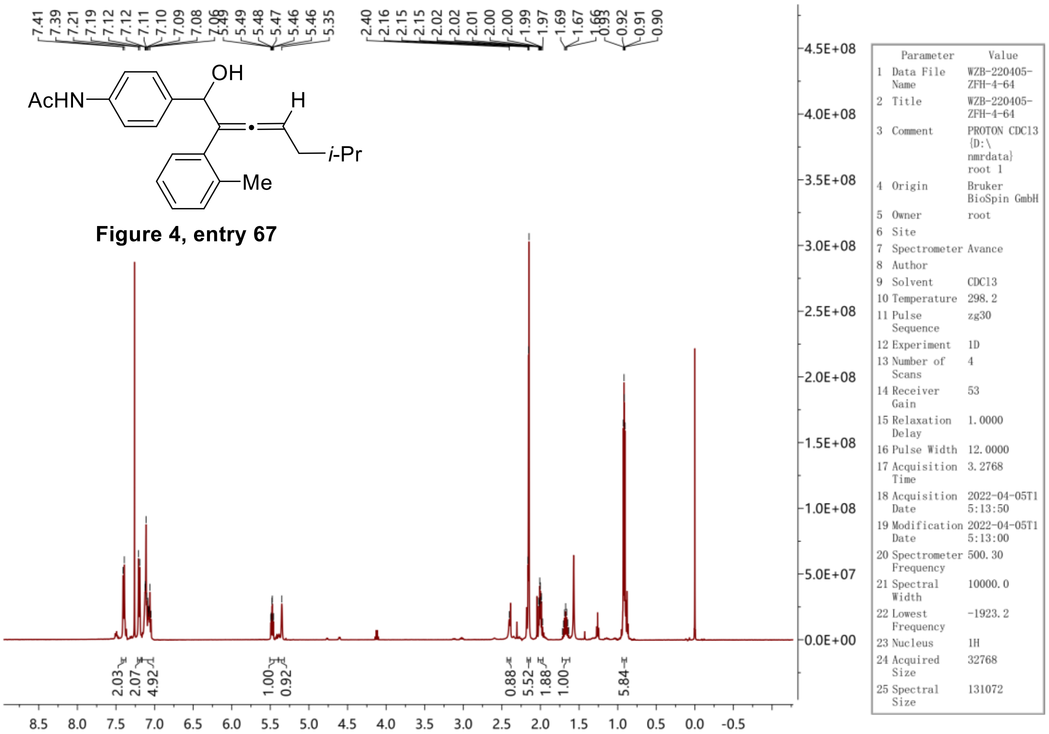

Supplementary Figure 146. ^{1}H NMR spectrum of compound **67**

^{13}C NMR (126 MHz, room temperature, $CDCl_3$)

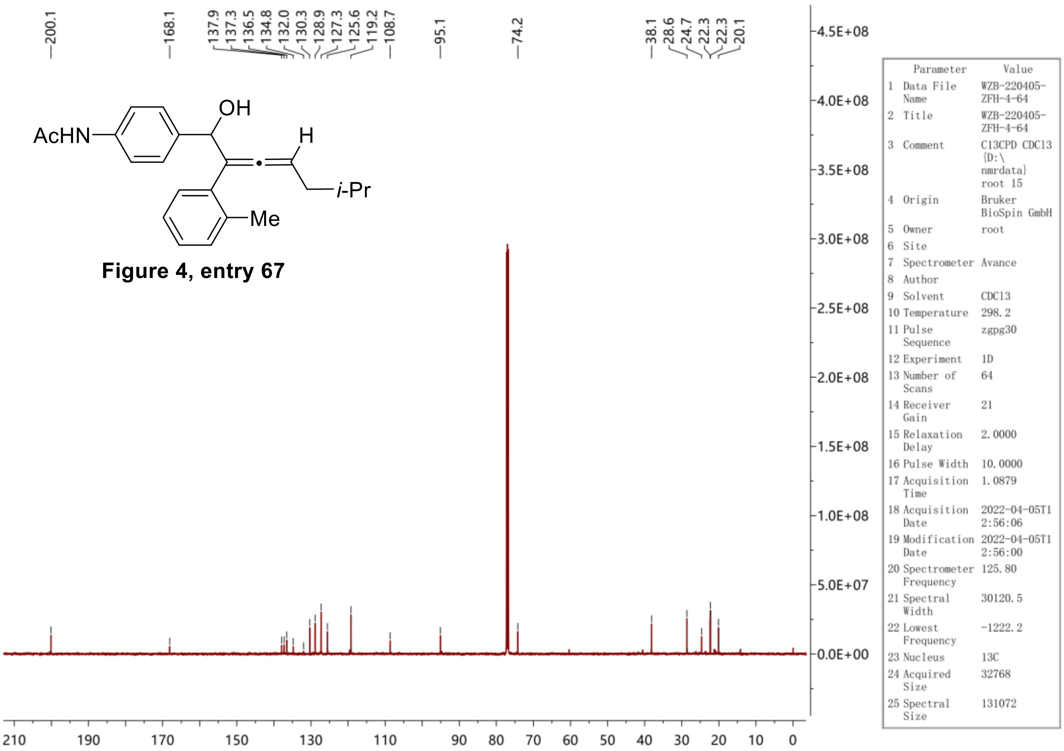

Supplementary Figure 147. ^{13}C NMR spectrum of compound **67**

^{1}H NMR (500 MHz, room temperature, $CDCl_3$)

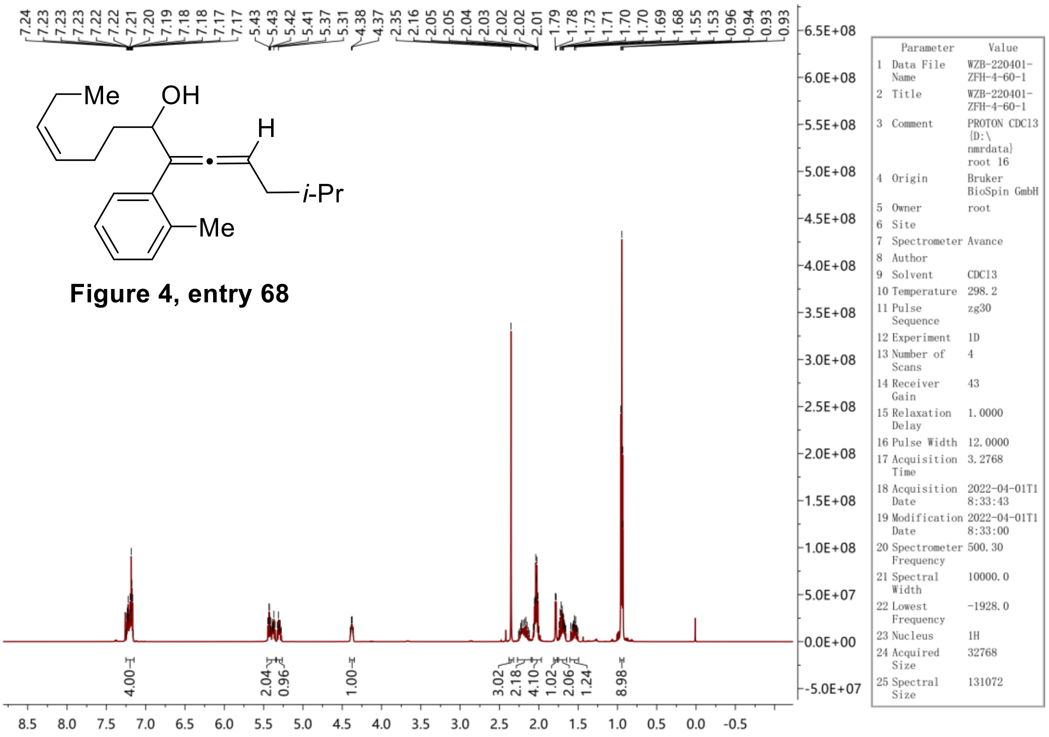

Supplementary Figure 148. ^{1}H NMR spectrum of compound **68**

^{13}C NMR (126 MHz, room temperature, $CDCl_3$)

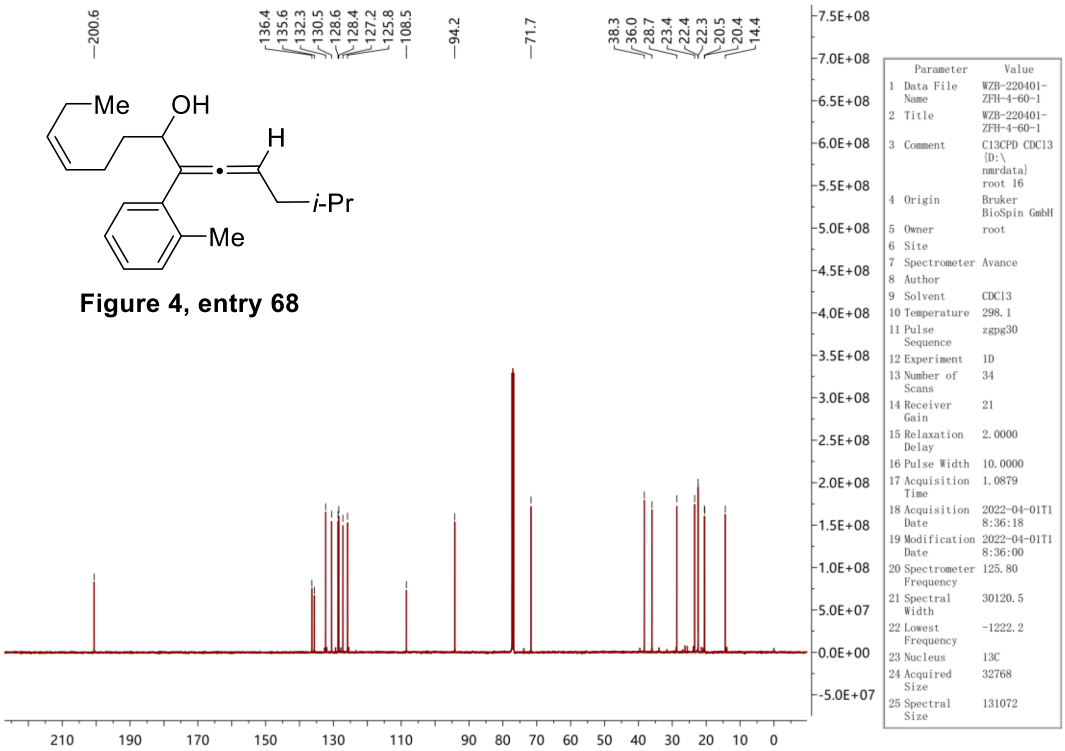

Supplementary Figure 149. ^{13}C NMR spectrum of compound **68**

^{1}H NMR (500 MHz, room temperature, $CDCl_3$)

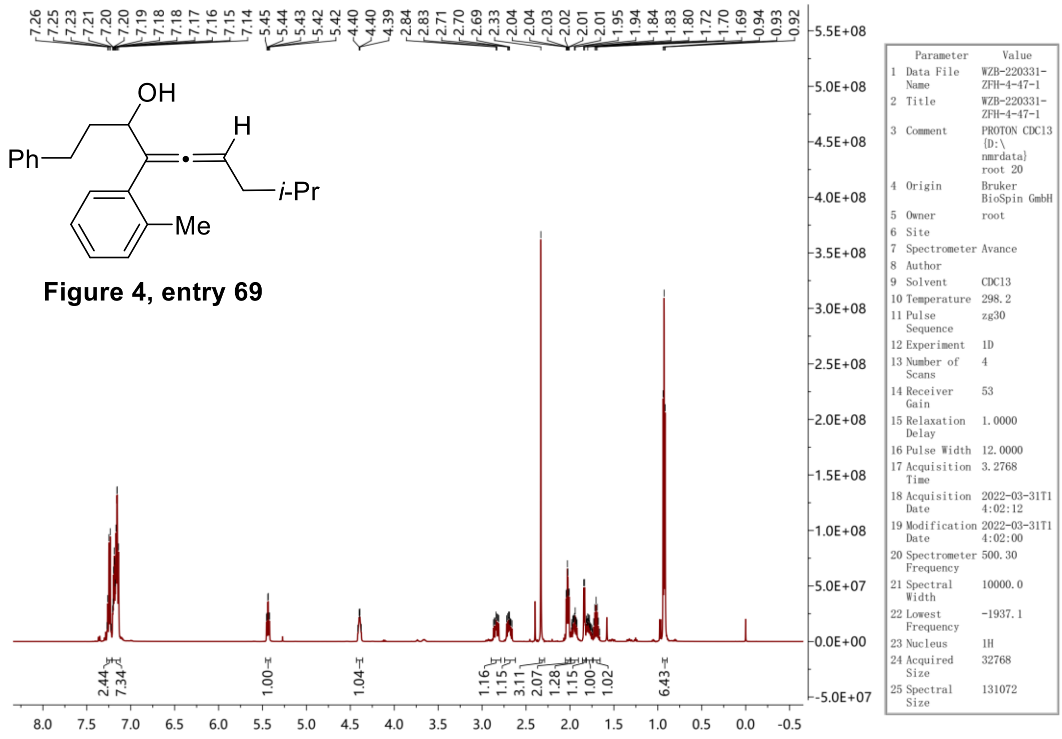

Supplementary Figure 150. ^{1}H NMR spectrum of compound **69**

^{13}C NMR (126 MHz, room temperature, $CDCl_3$)

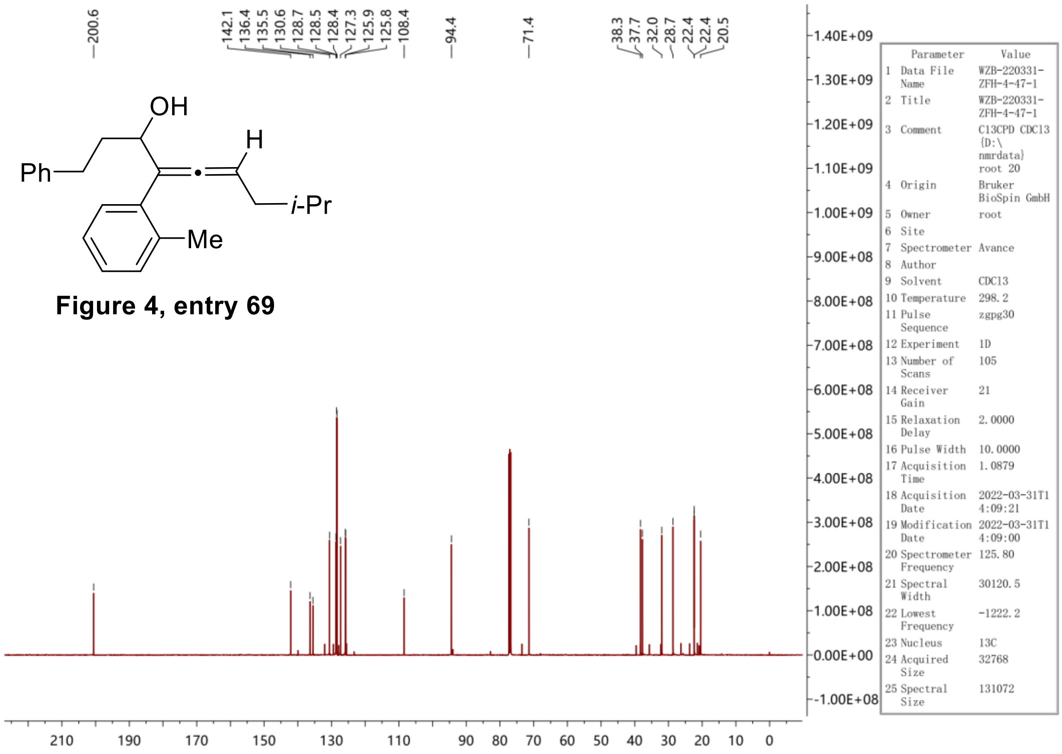

Supplementary Figure 151. ^{13}C NMR spectrum of compound **69**

^{1}H NMR (500 MHz, room temperature, $CDCl_3$)

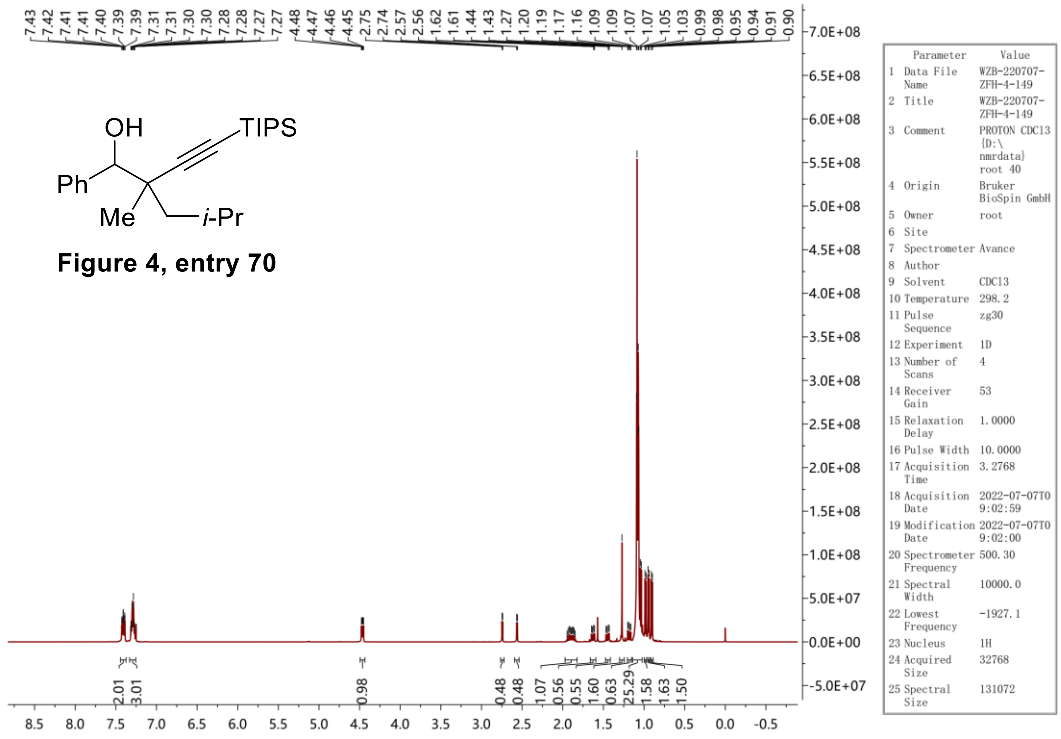

Supplementary Figure 152. ^{1}H NMR spectrum of compound **70**

^{13}C NMR (126 MHz, room temperature, $CDCl_3$)

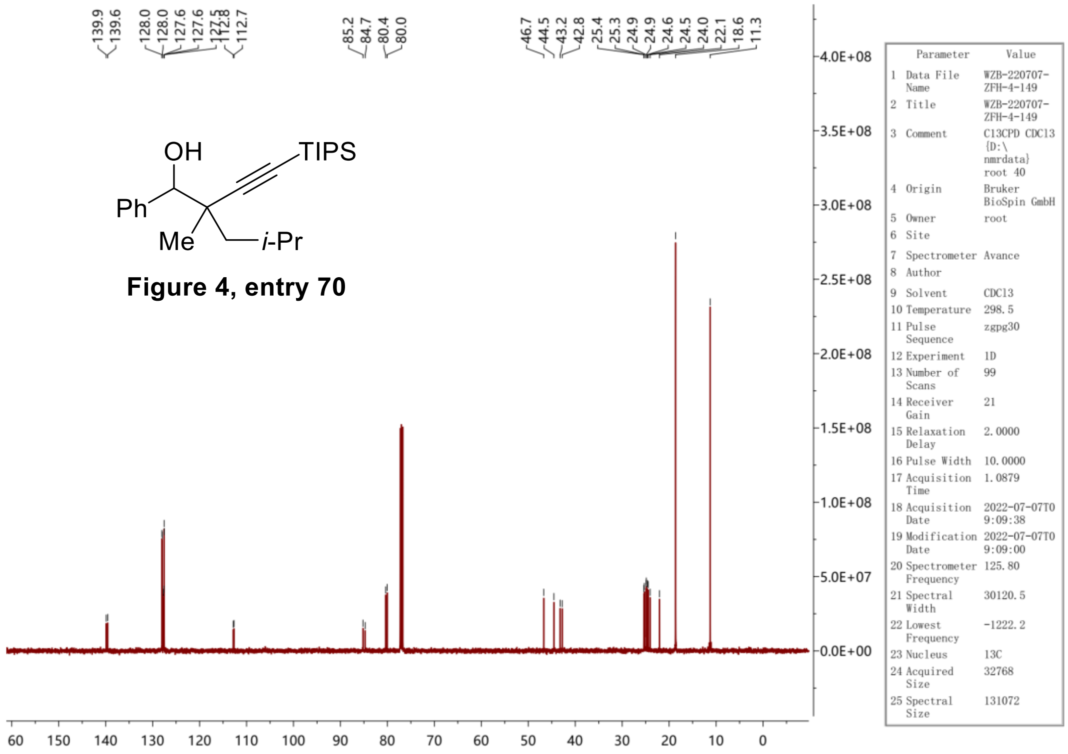

Supplementary Figure 153. ^{13}C NMR spectrum of compound **70**

^{1}H NMR (500 MHz, room temperature, $CDCl_3$)

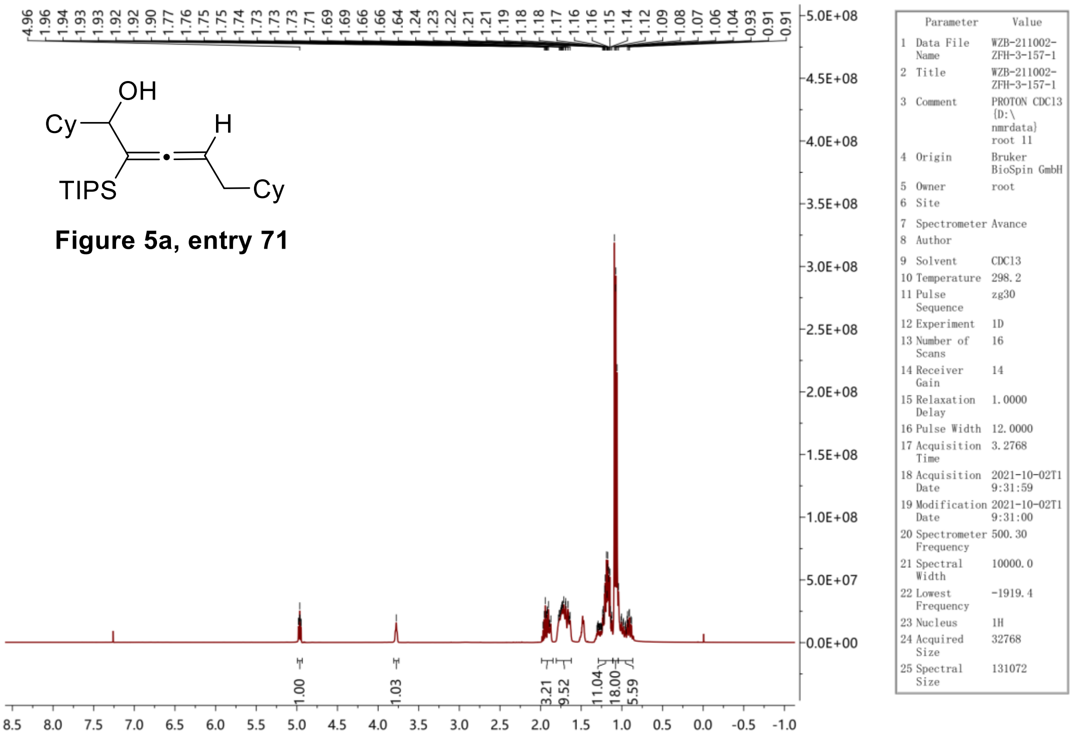

Supplementary Figure 154. ^{1}H NMR spectrum of compound **71**

^{13}C NMR (126 MHz, room temperature, $CDCl_3$)

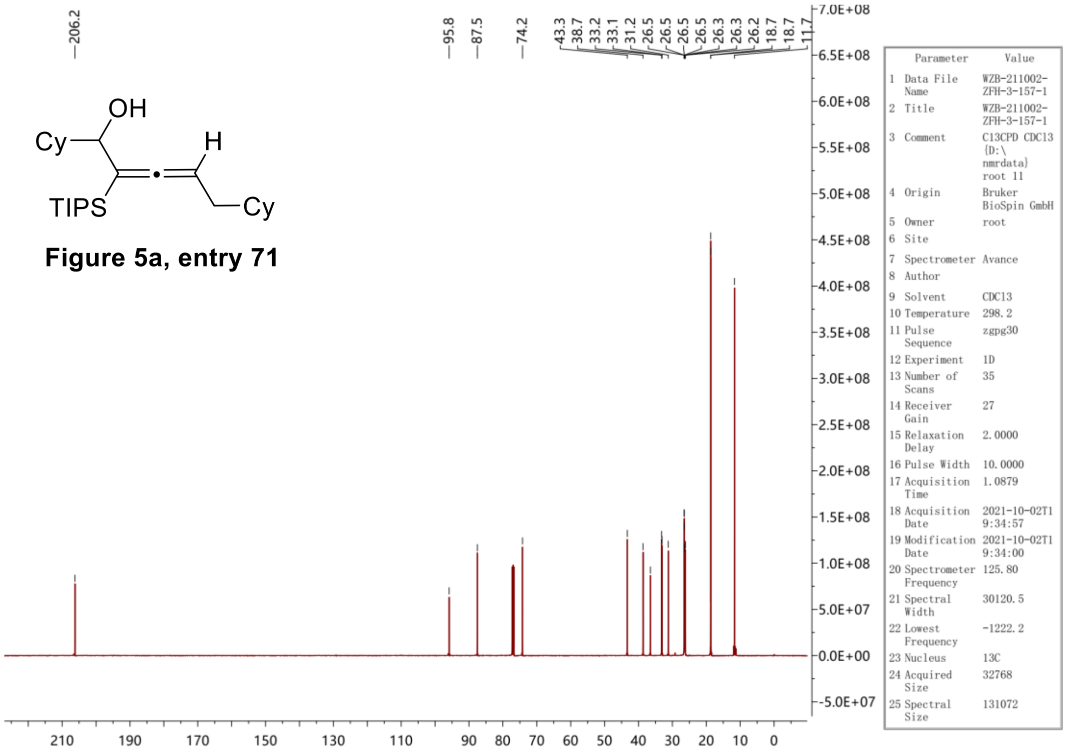

Supplementary Figure 155. ^{13}C NMR spectrum of compound **71**

^{1}H NMR (500 MHz, room temperature, $CDCl_3$)

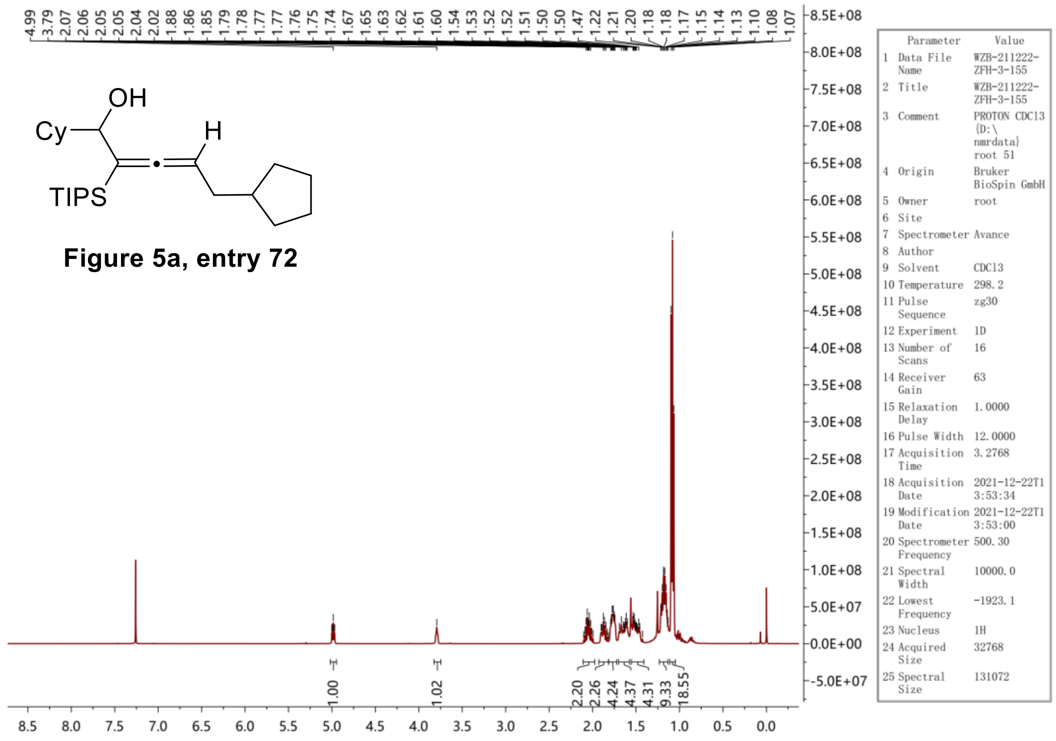

Supplementary Figure 156. ^{1}H NMR spectrum of compound **72**

^{13}C NMR (126 MHz, room temperature, $CDCl_3$)

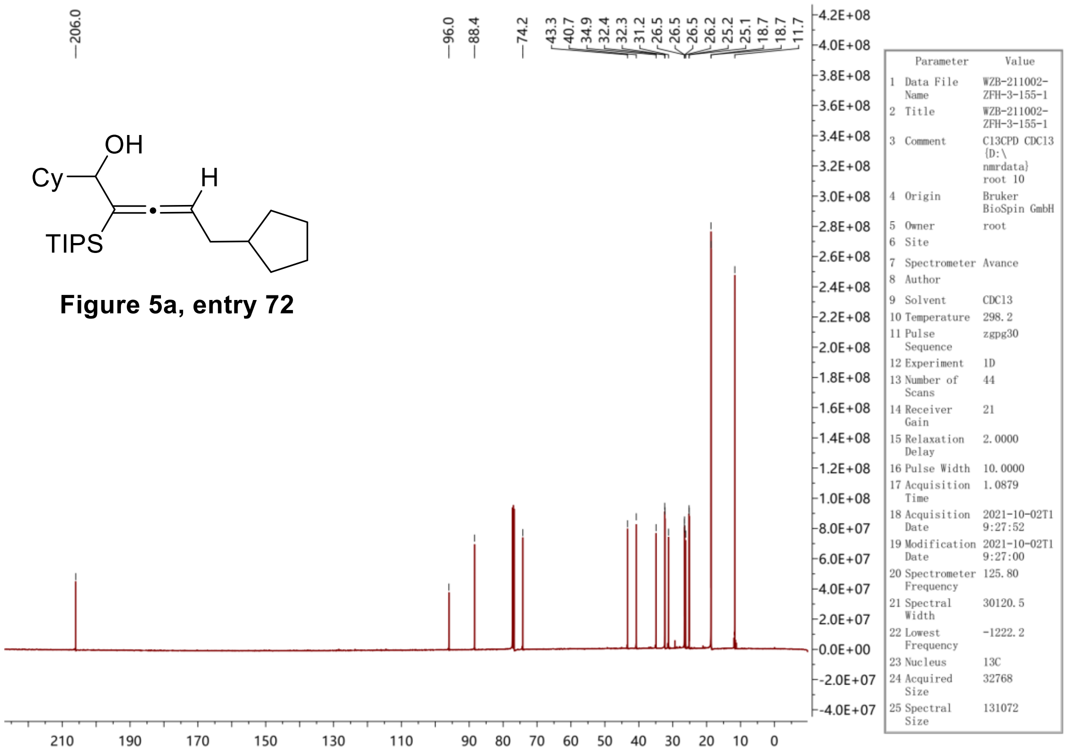

Supplementary Figure 157. ^{13}C NMR spectrum of compound **72**

^{1}H NMR (500 MHz, room temperature, $CDCl_3$)

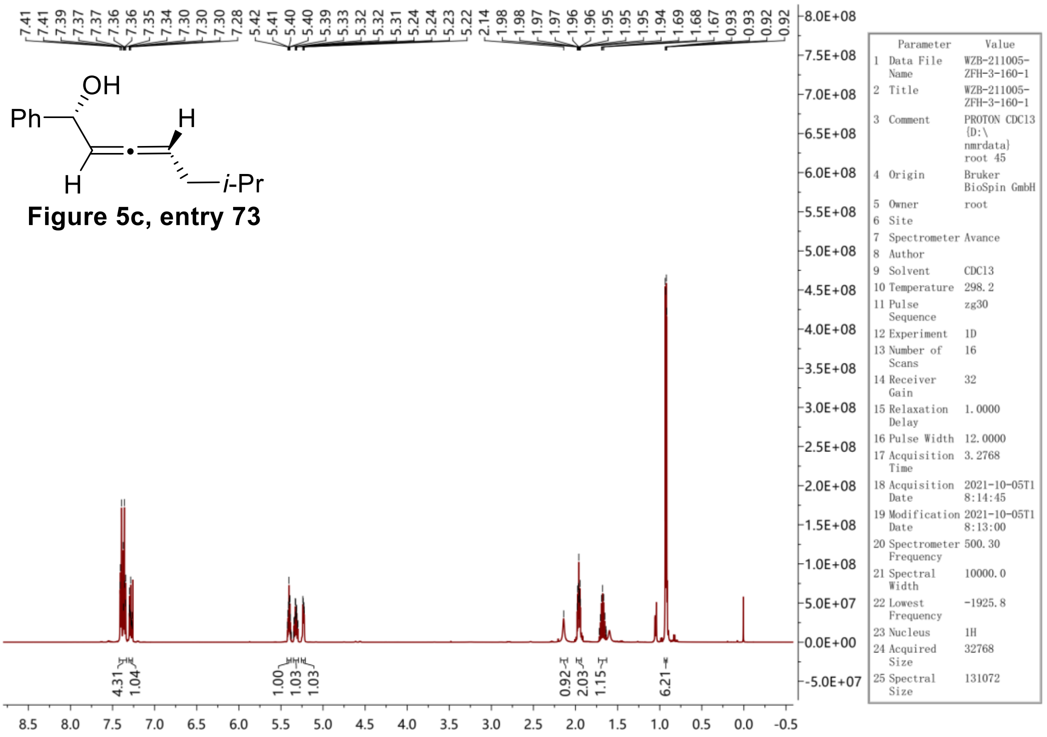

Supplementary Figure 158. ^{1}H NMR spectrum of compound **73**

^{13}C NMR (126 MHz, room temperature, $CDCl_3$)

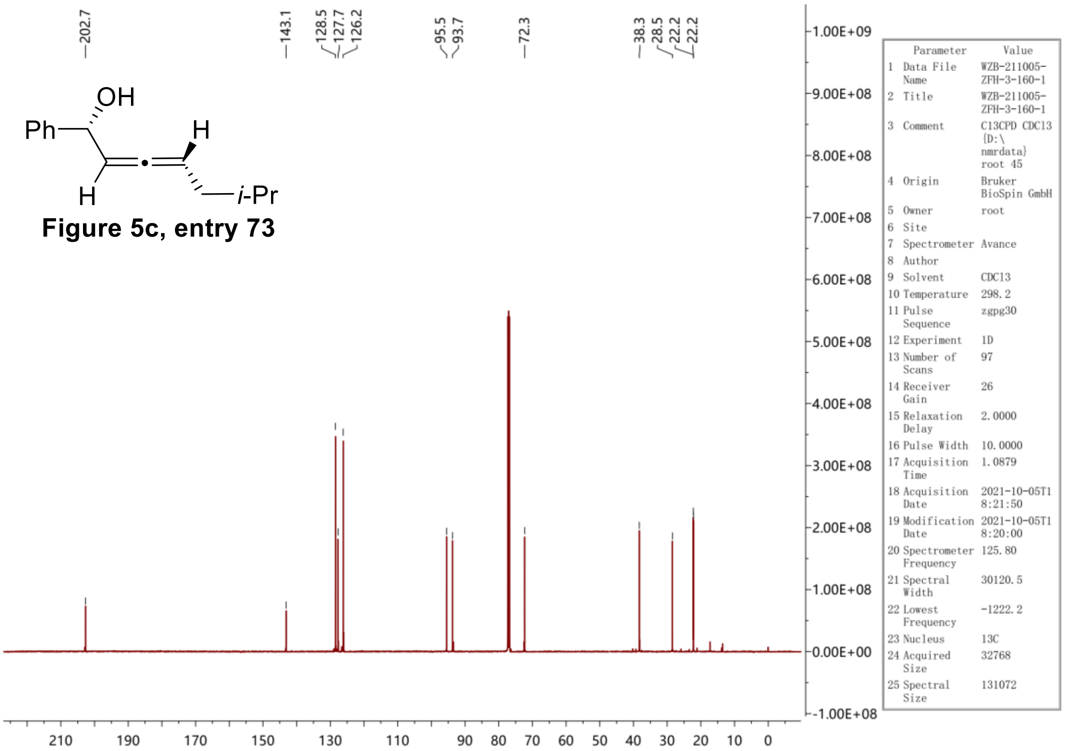

Supplementary Figure 159. ^{13}C NMR spectrum of compound **73**

^{1}H NMR (500 MHz, room temperature, $CDCl_3$)

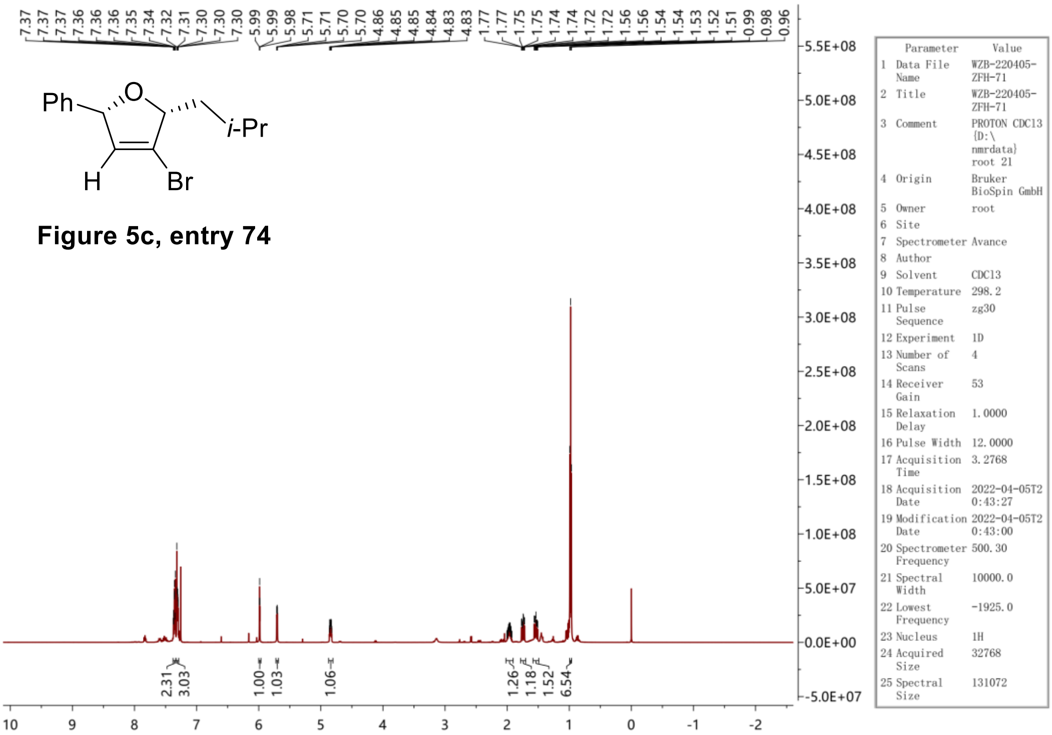

Supplementary Figure 160. ^{1}H NMR spectrum of compound **74**

^{13}C NMR (126 MHz, room temperature, $CDCl_3$)

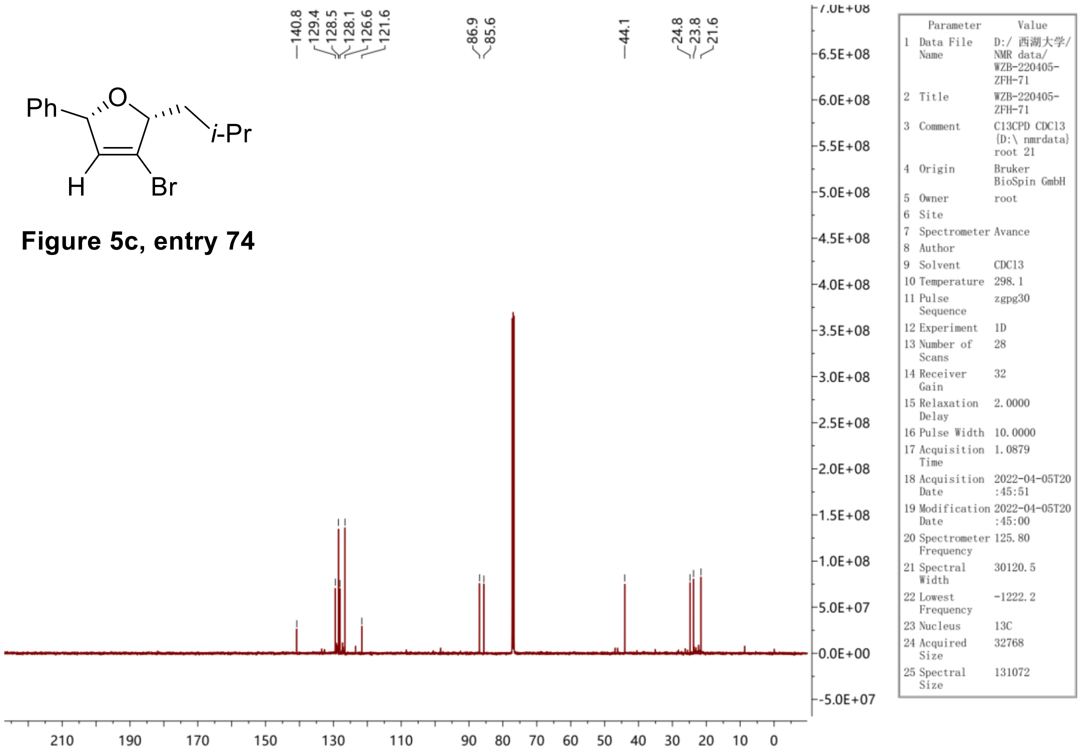

Supplementary Figure 161. ^{13}C NMR spectrum of compound **74**

^1H NMR (500 MHz, room temperature, $CDCl_3$)

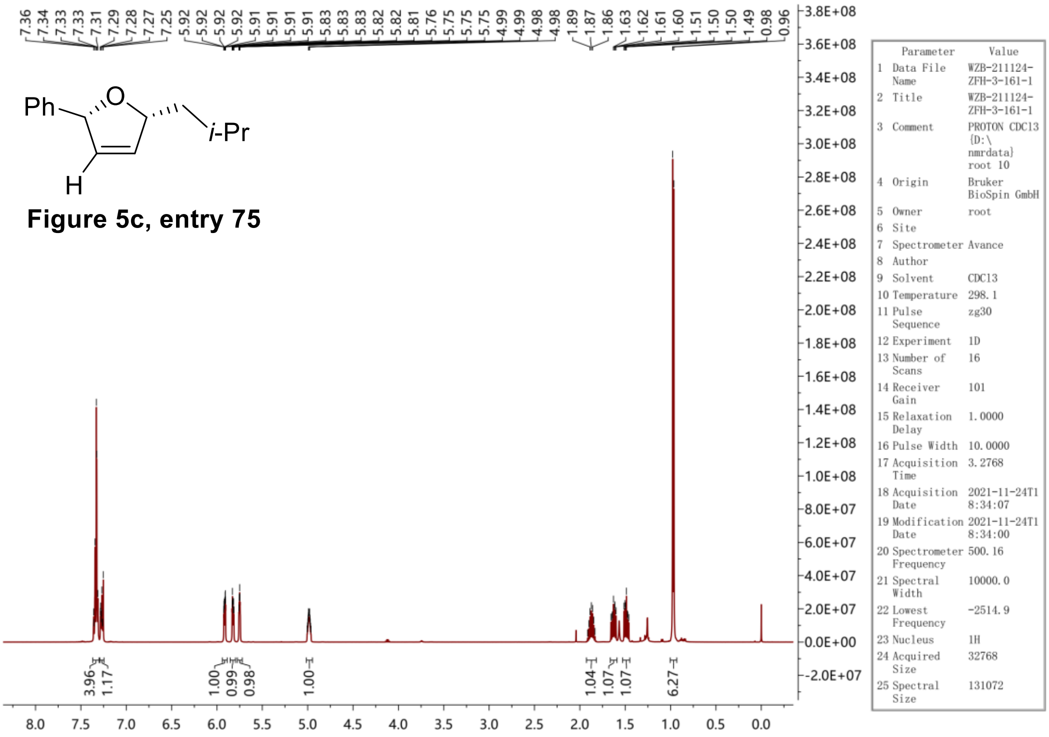

Supplementary Figure 162. ^1H NMR spectrum of compound **75**

^{13}C NMR (126 MHz, room temperature, $CDCl_3$)

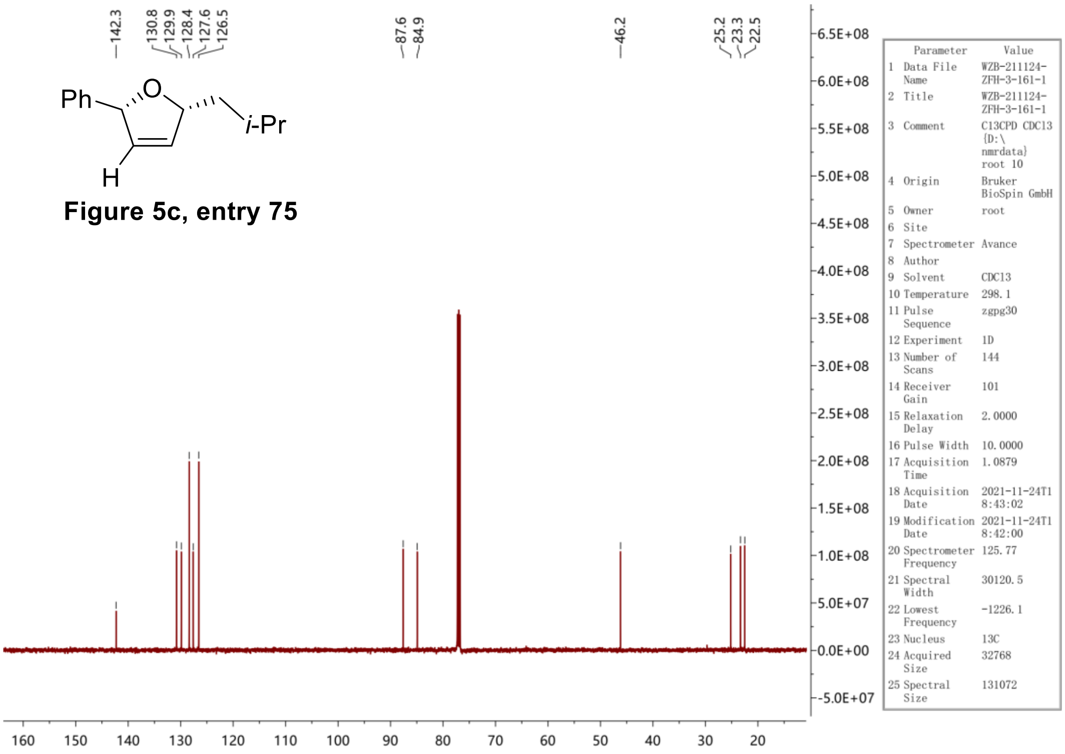

Supplementary Figure 163. ^{13}C NMR spectrum of compound **75**

^1H NMR (500 MHz, room temperature, $CDCl_3$)

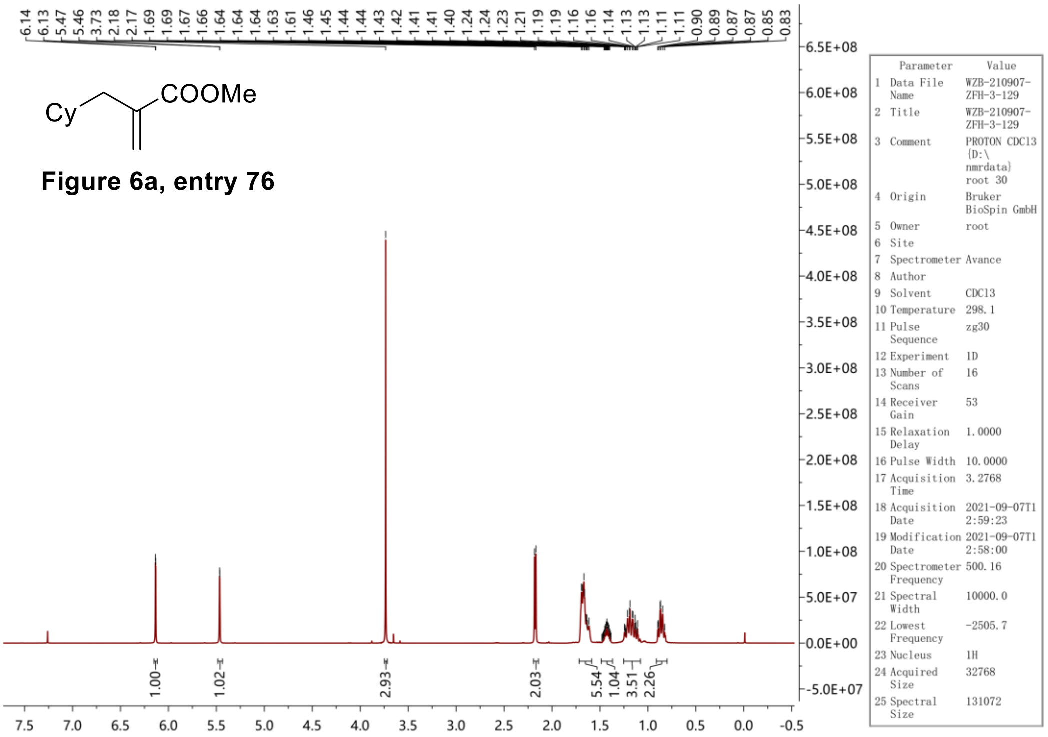

Supplementary Figure 164. ^1H NMR spectrum of compound **76**

^{13}C NMR (126 MHz, room temperature, $CDCl_3$)

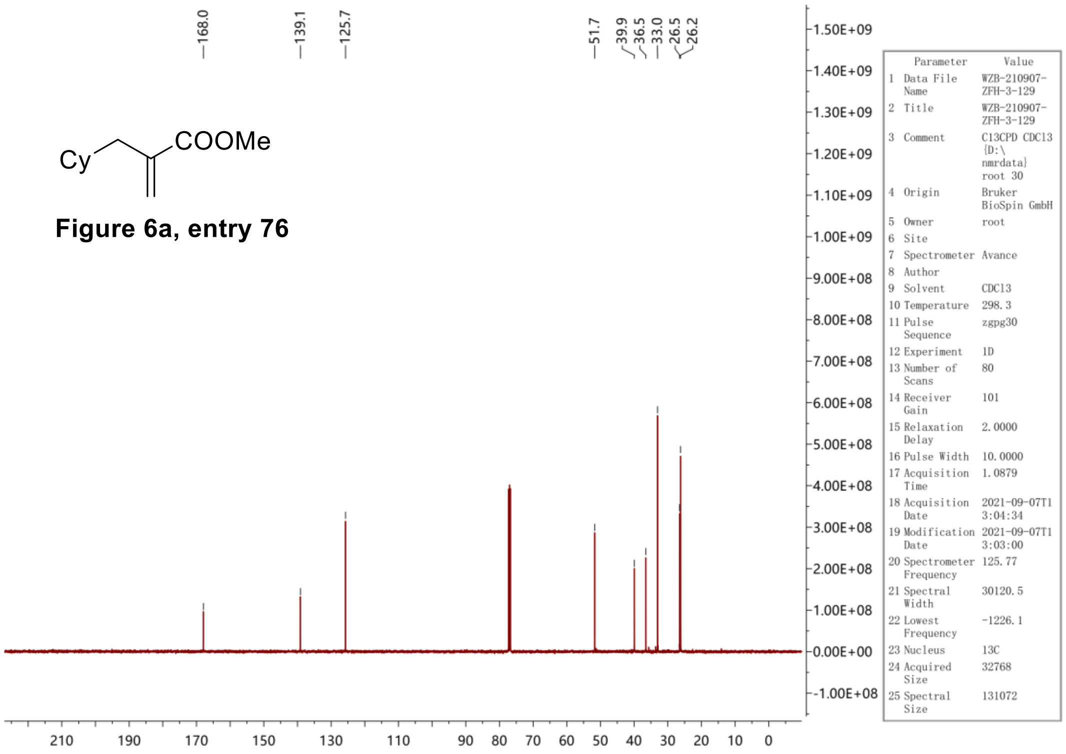

Supplementary Figure 165. ^{13}C NMR spectrum of compound **76**

1.9 Stereoselectivity Analysis

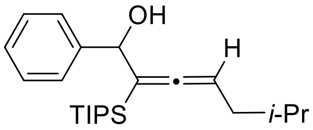

Figure 3, entry 3

(*R*,*S*)-**L1**: 95% ee; (*S*,*R*)-**L1**: 94% ee

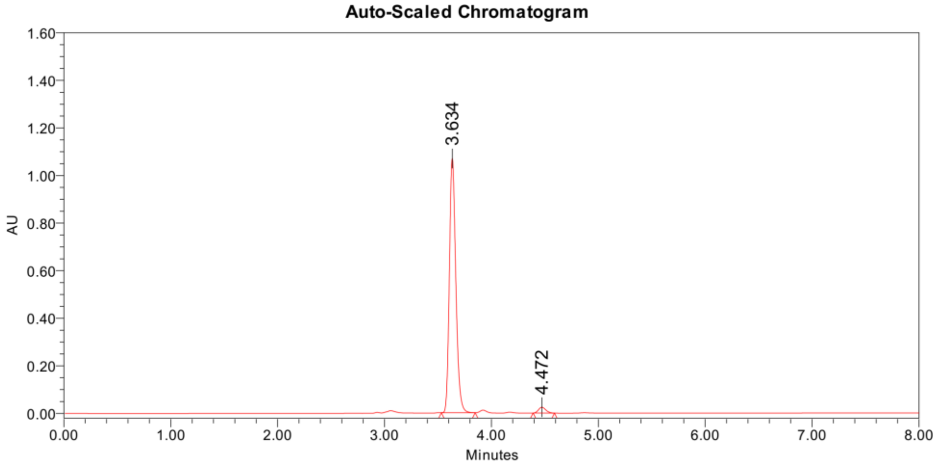

Peak Results

	Name	RT	Area	Height	% Area
1		3.634	4469601	1068964	97.46
2		4.472	116721	24542	2.54

Supplementary Figure 166. HPLC Spectra of **3** obtained from (*R*,*S*)-**L1**.

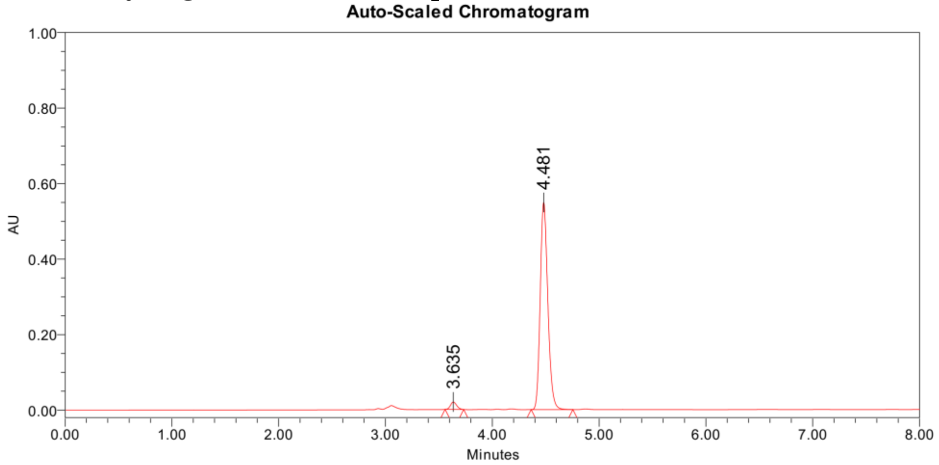

Peak Results

	Name	RT	Area	Height	% Area
1		3.635	78422	19526	2.80
2		4.481	2718060	548587	97.20

Supplementary Figure 167. HPLC Spectra of **3** obtained from (*S*,*R*)-**L1**.

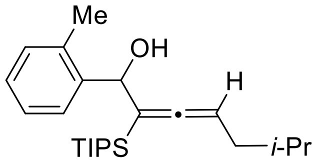

Figure 3, entry 4

(*R*,*S*)-**L1**: 96% ee; (*S*,*R*)-**L1**: 97% ee

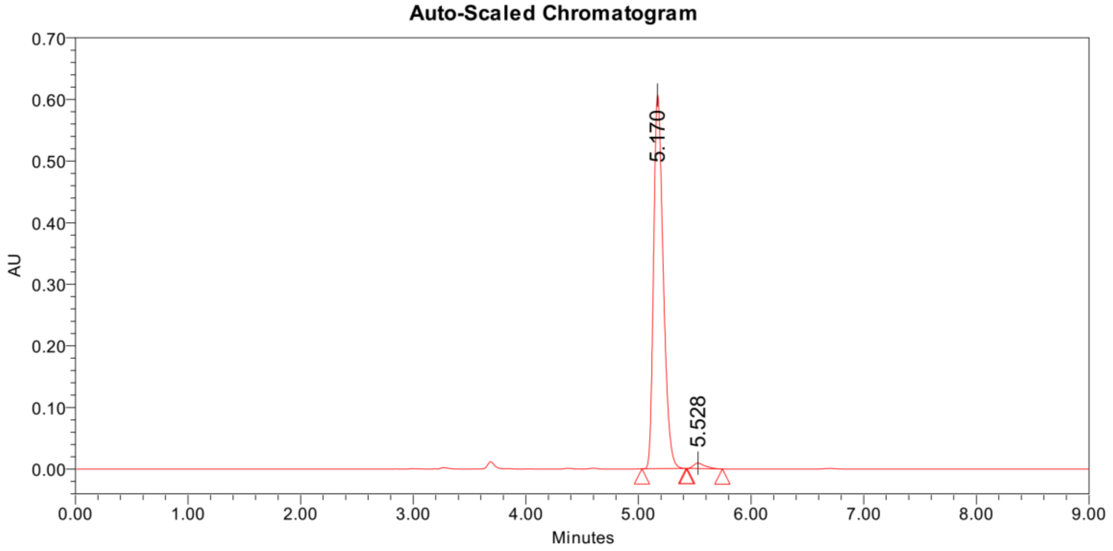

Peak Results

	Name	RT	Area	Height	% Area
1		5.170	3619223	606674	98.28
2		5.528	63440	8904	1.72

Supplementary Figure 168. HPLC Spectra of **4** obtained from (*R*,*S*)-**L1**.

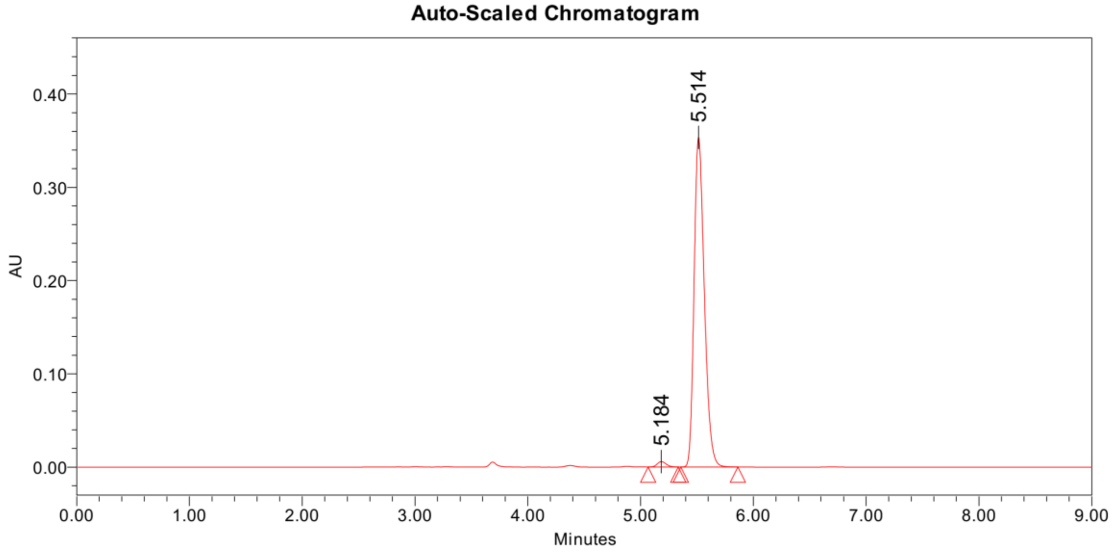

Peak Results

	Name	RT	Area	Height	% Area
1		5.184	32248	5869	1.42
2		5.514	2236333	353524	98.58

Supplementary Figure 169. HPLC Spectra of **4** obtained from (*S*,*R*)-**L1**.

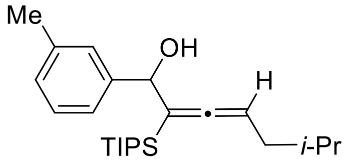

Figure 3, entry 5

(*R*,*S*)-**L1**: 93% ee; (*S*,*R*)-**L1**: 93% ee

Auto-Scaled Chromatogram

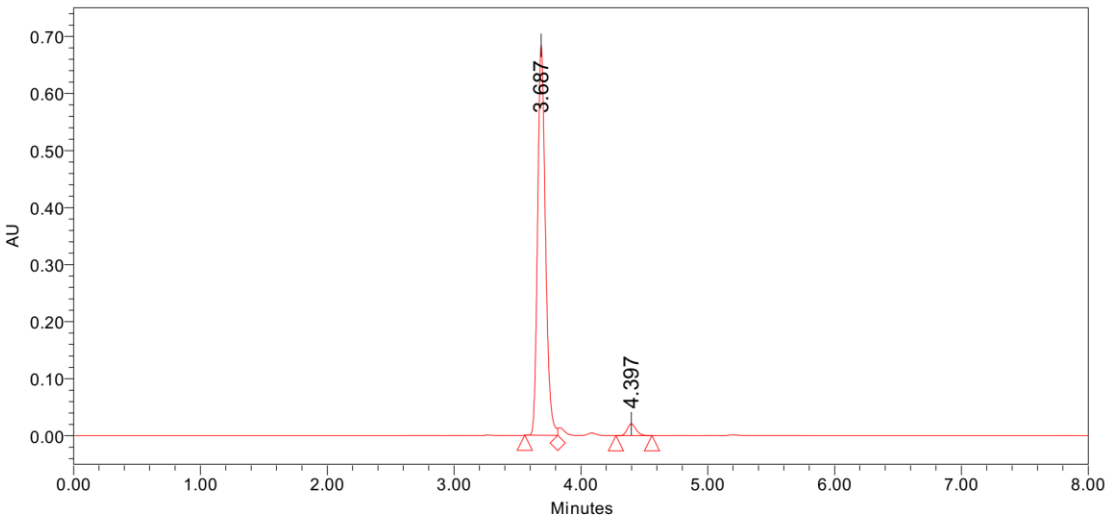

Peak Results

	Name	RT	Area	Height	% Area
1		3.687	2935493	683639	96.64
2		4.397	101924	21021	3.36

Supplementary Figure 170. HPLC Spectra of **5** obtained from (*R*,*S*)-**L1**.

Auto-Scaled Chromatogram

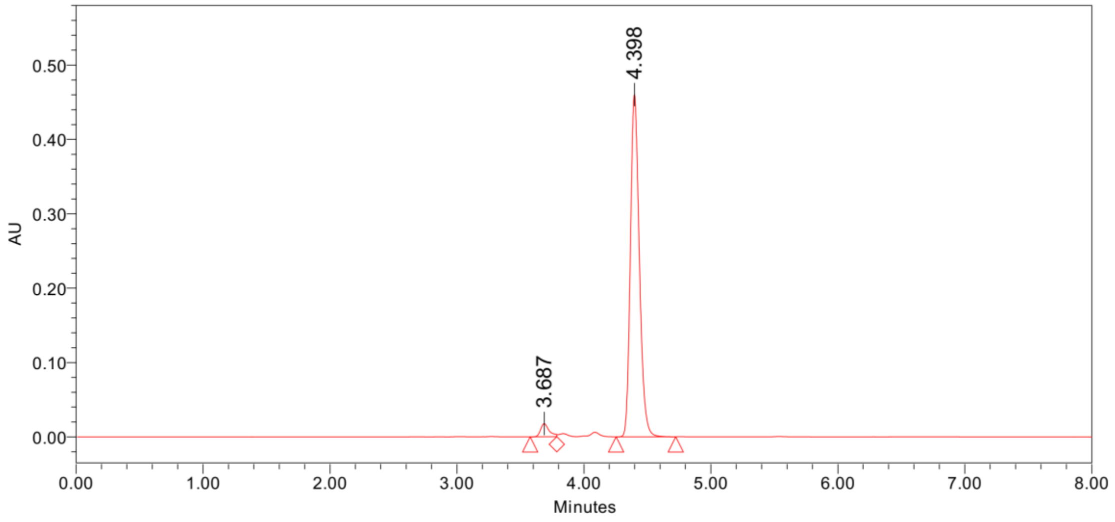

Peak Results

	Name	RT	Area	Height	% Area
1		3.687	82595	17699	3.54
2		4.398	2248771	460452	96.46

Supplementary Figure 171. HPLC Spectra of **5** obtained from (*S*,*R*)-**L1**.

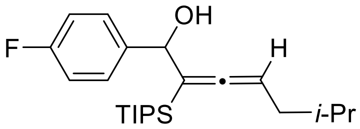

Figure 3, entry 6

(*R*,*S*)-**L1**: 93% ee; (*S*,*R*)-**L1**: 92% ee

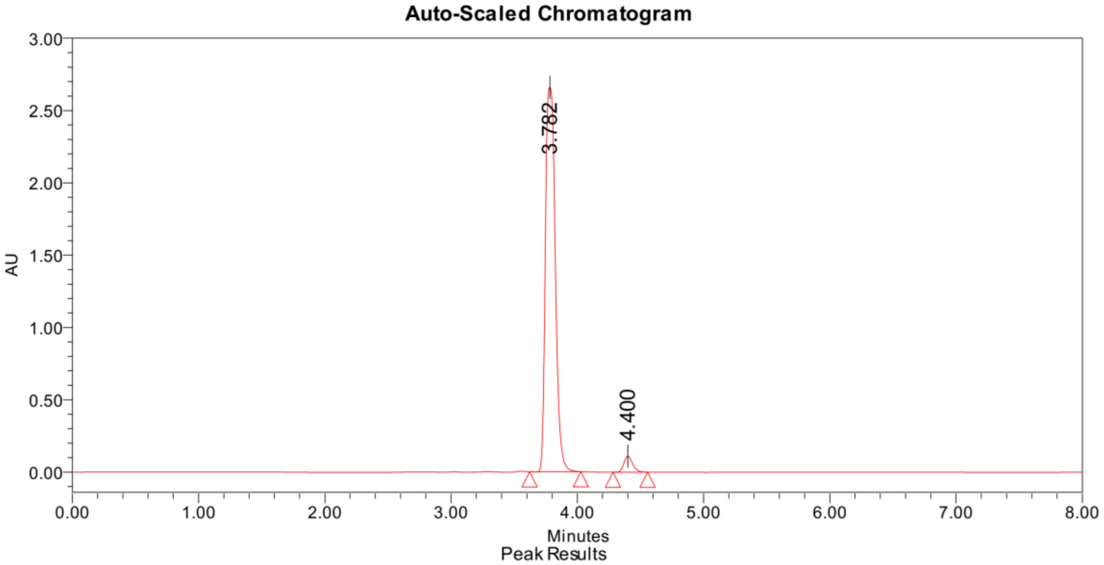

Peak Results

	Name	RT	Area	Height	% Area
1		3.782	14392628	2658607	96.45
2		4.400	530443	111004	3.55

Supplementary Figure 172. HPLC Spectra of **6** obtained from (*R*,*S*)-**L1**.

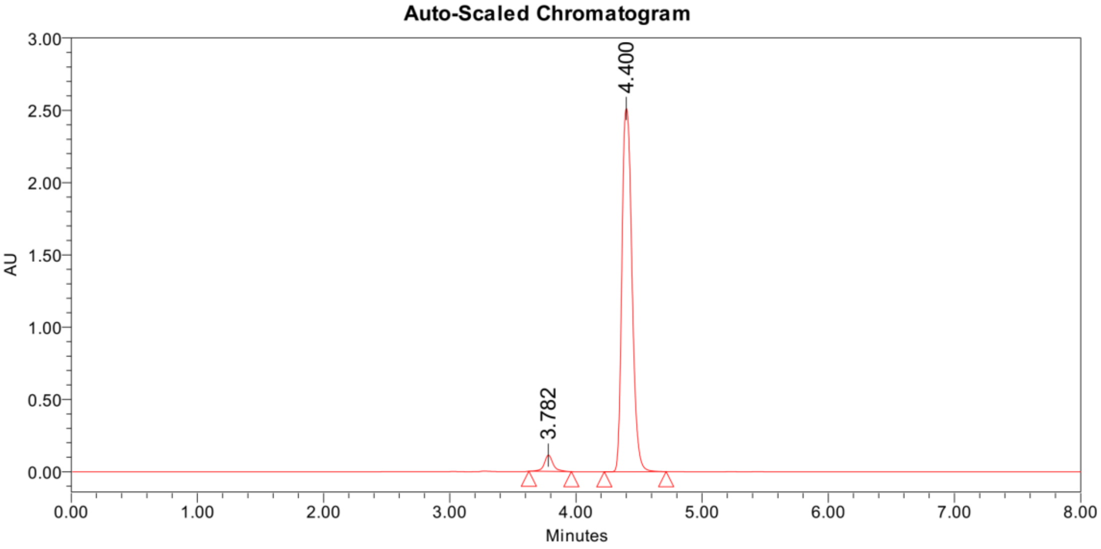

Peak Results

	Name	RT	Area	Height	% Area
1		3.782	549257	112928	3.80
2		4.400	13917207	2513561	96.20

Supplementary Figure 173. HPLC Spectra of **6** obtained from (*S*,*R*)-**L1**.

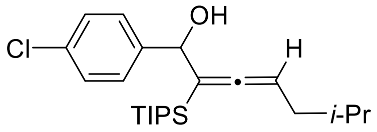

Figure 3, entry 7

(*R*,*S*)-**L1**: 91% ee; (*S*,*R*)-**L1**: 93% ee

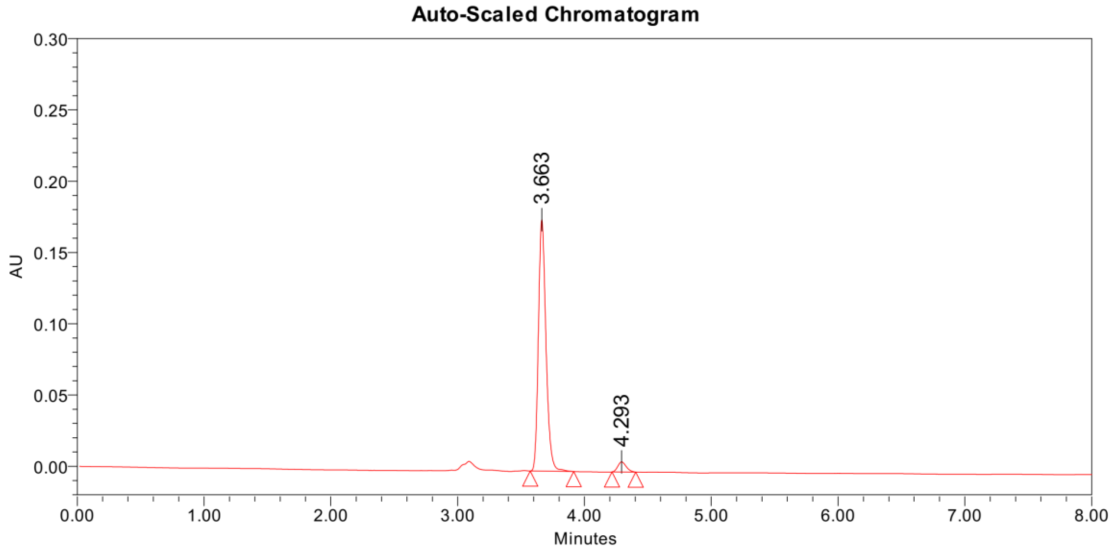

Peak Results

	Name	RT	Area	Height	% Area
1		3.663	732845	176387	95.74
2		4.293	32602	7094	4.26

Supplementary Figure 174. HPLC Spectra of **7** obtained from (*R*,*S*)-**L1**.

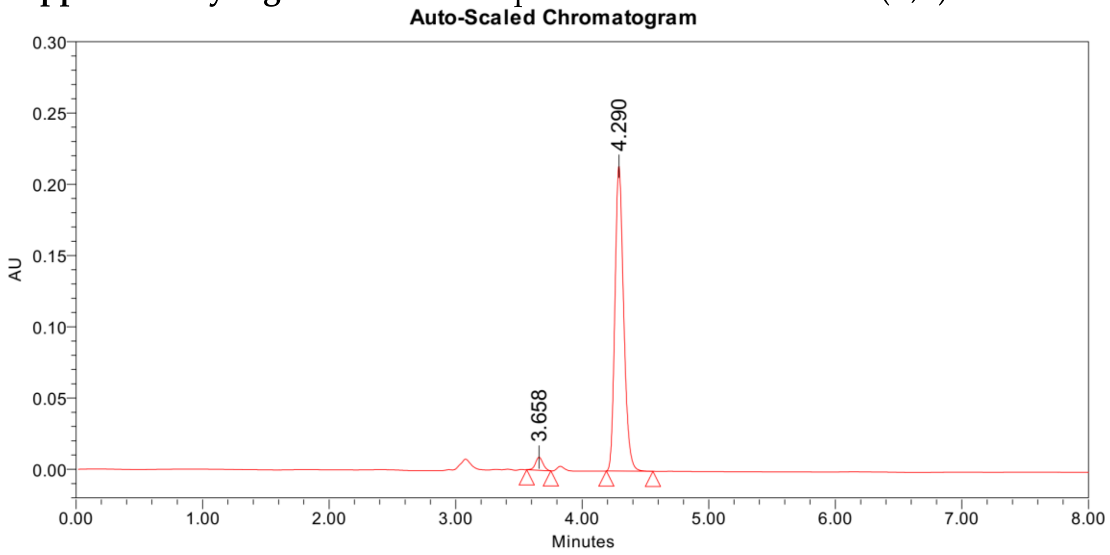

Peak Results

	Name	RT	Area	Height	% Area
1		3.658	37792	9206	3.54
2		4.290	1030671	214053	96.46

Supplementary Figure 175. HPLC Spectra of **7** obtained from (*S*,*R*)-**L1**.

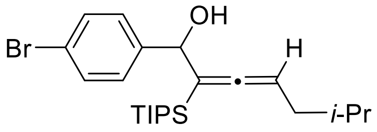

Figure 3, entry 8
(*R*,*S*)-**L1**: 93% ee; (*S*,*R*)-**L1**: 92% ee

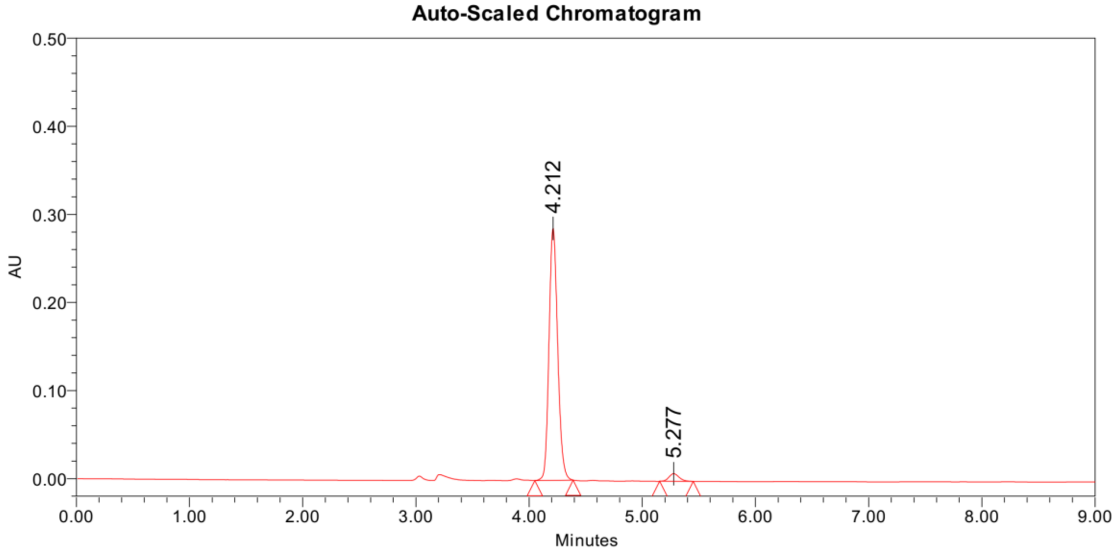

Peak Results

	Name	RT	Area	Height	% Area
1		4.212	1524001	286173	96.63
2		5.277	53169	8560	3.37

Supplementary Figure 176. HPLC Spectra of **8** obtained from (*R*,*S*)-**L1**.

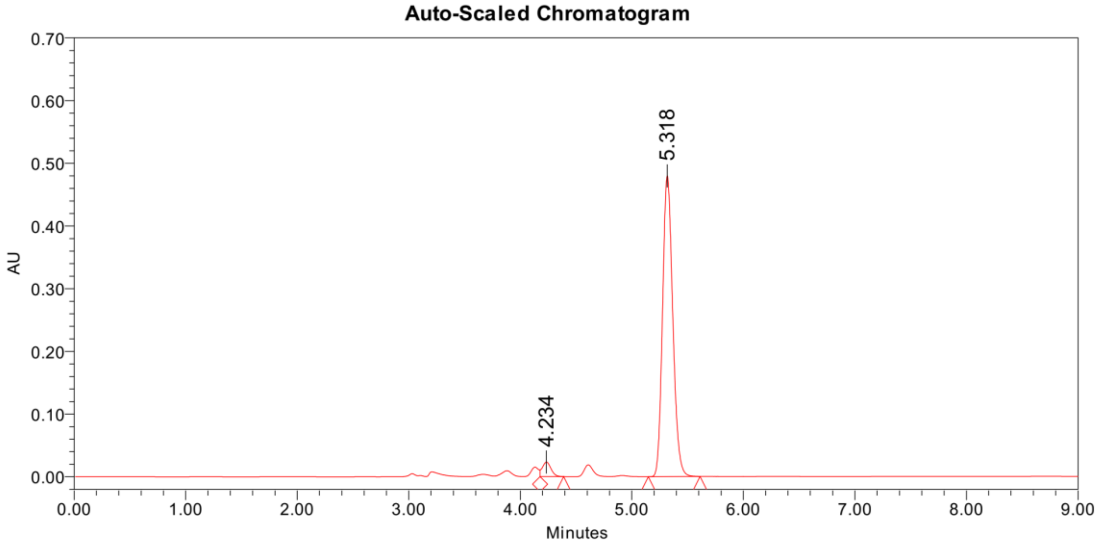

Peak Results

	Name	RT	Area	Height	% Area
1		4.234	126691	23230	3.99
2		5.318	3048872	479821	96.01

Supplementary Figure 177. HPLC Spectra of **8** obtained from (*S*,*R*)-**L1**.

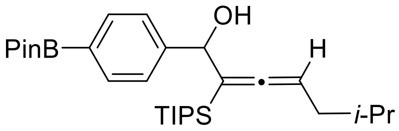

Figure 3, entry 9

(*R*,*S*)-**L1**: 96% ee; (*S*,*R*)-**L1**: 95% ee

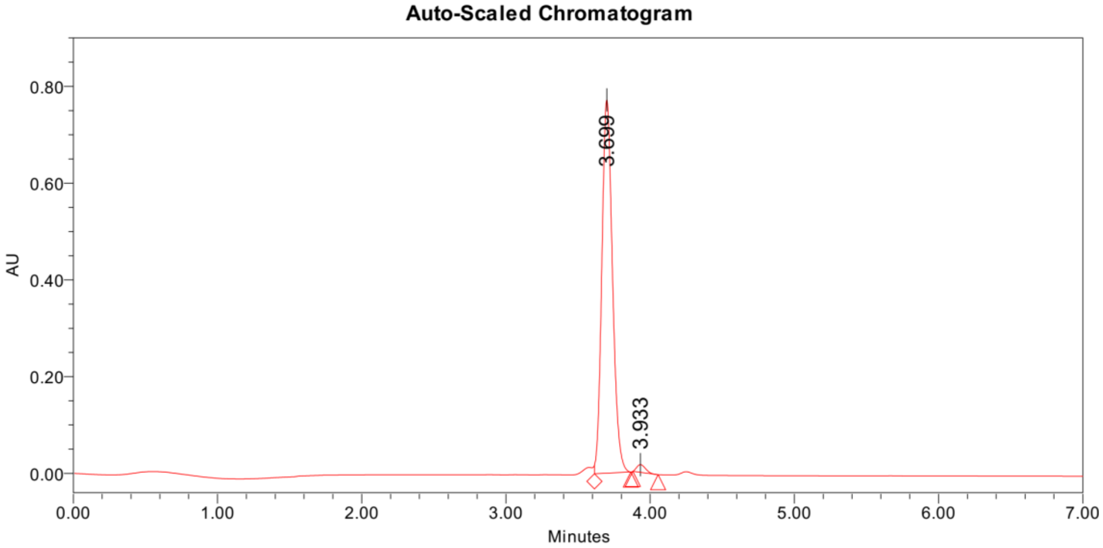

Peak Results

	Name	RT	Area	Height	% Area
1		3.699	3957883	771795	98.31
2		3.933	67934	16001	1.69

Supplementary Figure 178. HPLC Spectra of **9** obtained from (*R*,*S*)-**L1**.

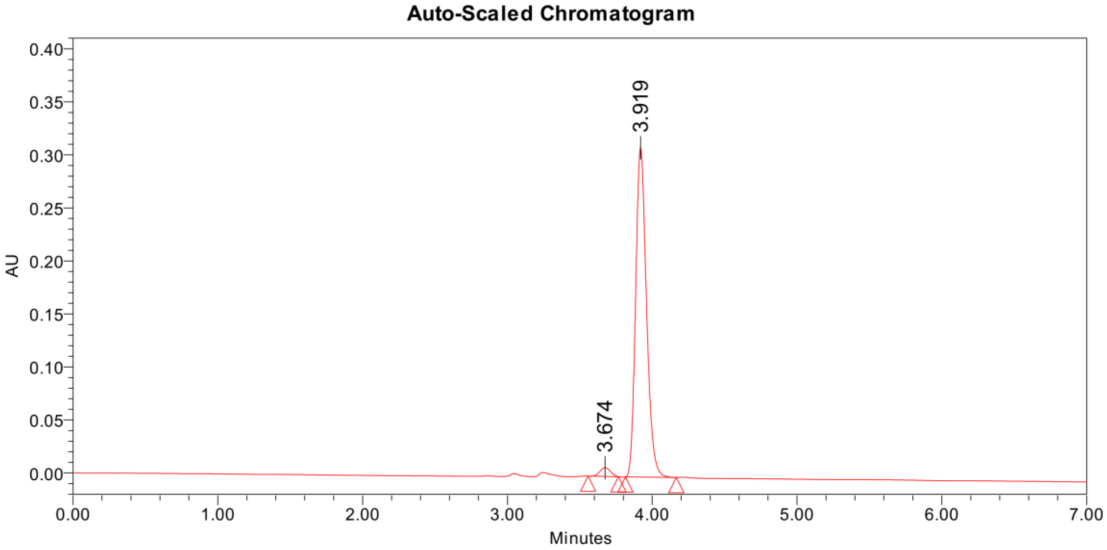

Peak Results

	Name	RT	Area	Height	% Area
1		3.674	41180	8208	2.48
2		3.919	1620579	310595	97.52

Supplementary Figure 179. HPLC Spectra of **9** obtained from (*S*,*R*)-**L1**.

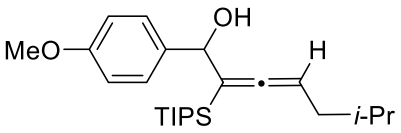

Figure 3, entry 10

(*R*,*S*)-**L1**: 95% ee; (*S*,*R*)-**L1**: 95% ee

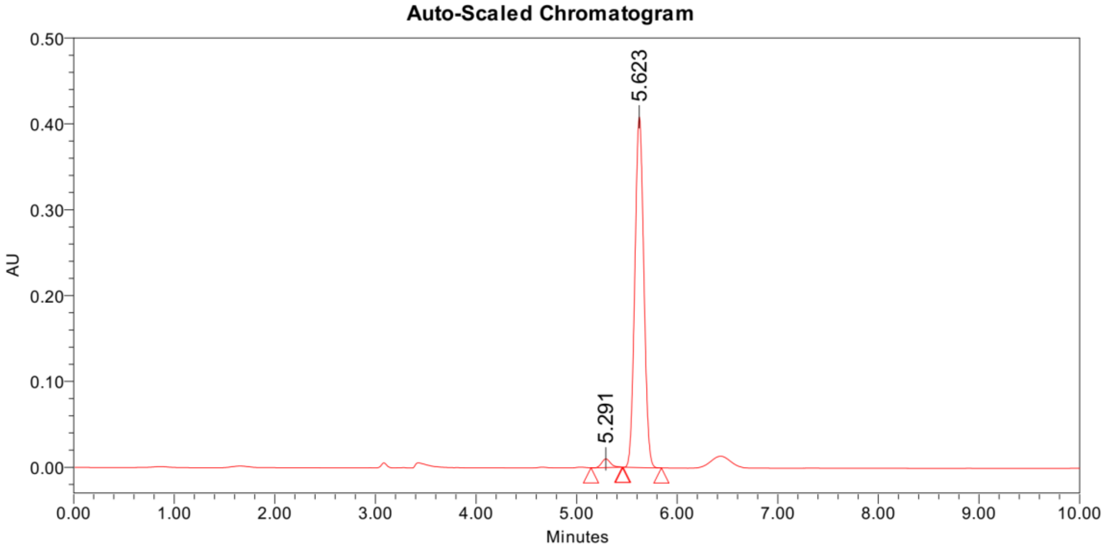

Peak Results

	Name	RT	Area	Height	% Area
1		5.291	60180	9941	2.32
2		5.623	2537734	408654	97.68

Supplementary Figure 180. HPLC Spectra of **10** obtained from (*R*,*S*)-**L1**.

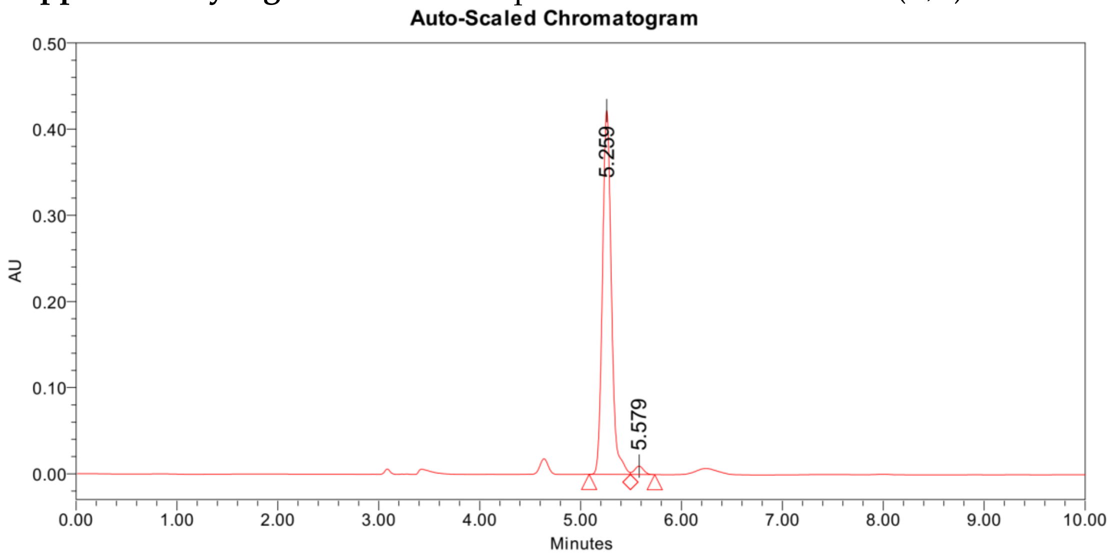

Peak Results

	Name	RT	Area	Height	% Area
1		5.259	2597467	422575	97.71
2		5.579	60897	9748	2.29

Supplementary Figure 181. HPLC Spectra of **10** obtained from (*S*,*R*)-**L1**.

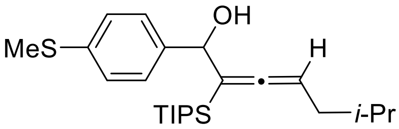

Figure 3, entry 11

(*R*,*S*)-**L1**: 95% ee; (*S*,*R*)-**L1**: 94% ee

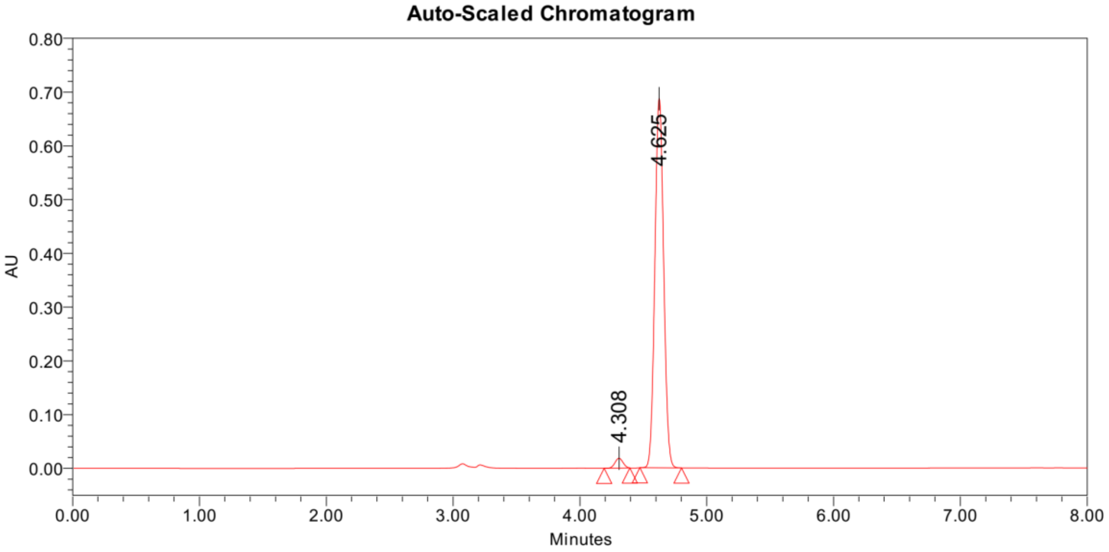

Peak Results

	Name	RT	Area	Height	% Area
1		4.308	80423	18310	2.37
2		4.625	3315242	686886	97.63

Supplementary Figure 182. HPLC Spectra of **11** obtained from (*R*,*S*)-**L1**.

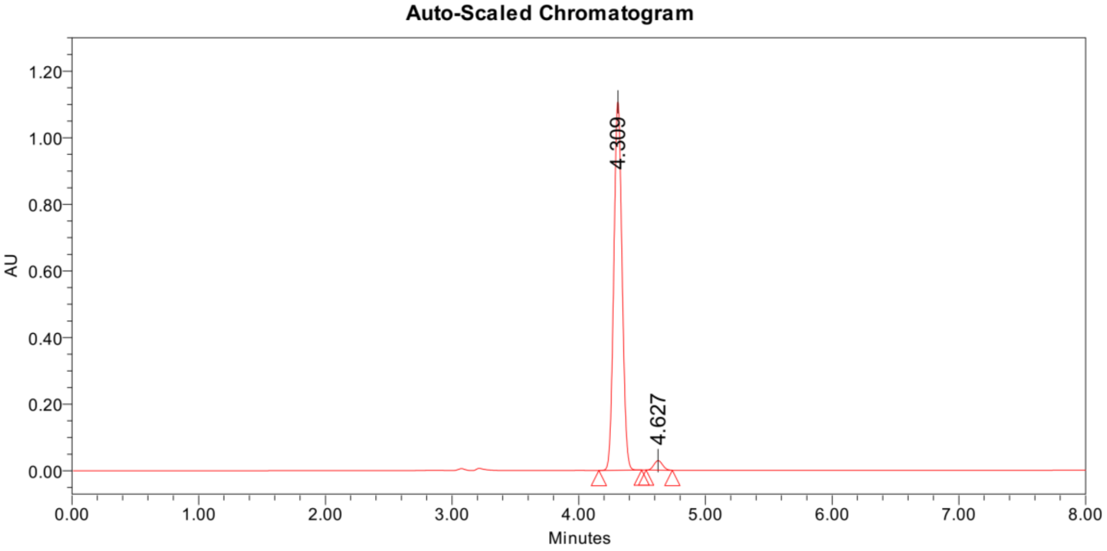

Peak Results

	Name	RT	Area	Height	% Area
1		4.309	5007791	1106631	97.36
2		4.627	135881	28671	2.64

Supplementary Figure 183. HPLC Spectra of **11** obtained from (*S*,*R*)-**L1**.

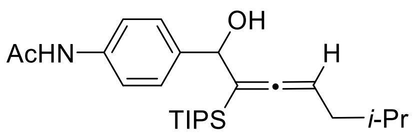

Figure 3, entry 12
(*R*,*S*)-**L1**: 94% ee; (*S*,*R*)-**L1**: 94% ee

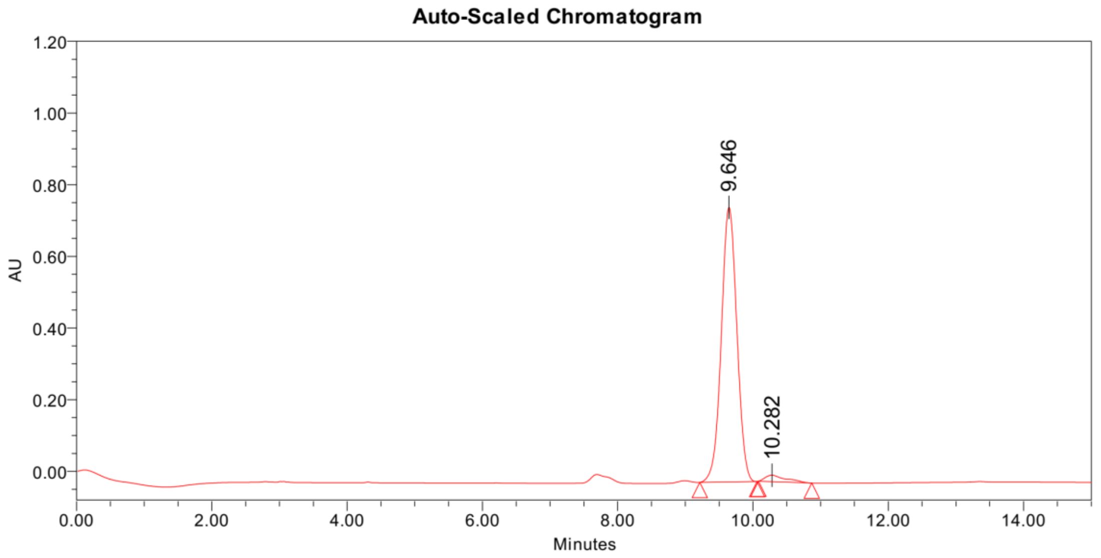

Peak Results

	Name	RT	Area	Height	% Area
1		9.646	12240010	766394	96.90
2		10.282	391481	18079	3.10

Supplementary Figure 184. HPLC Spectra of **12** obtained from (*R*,*S*)-**L1**.

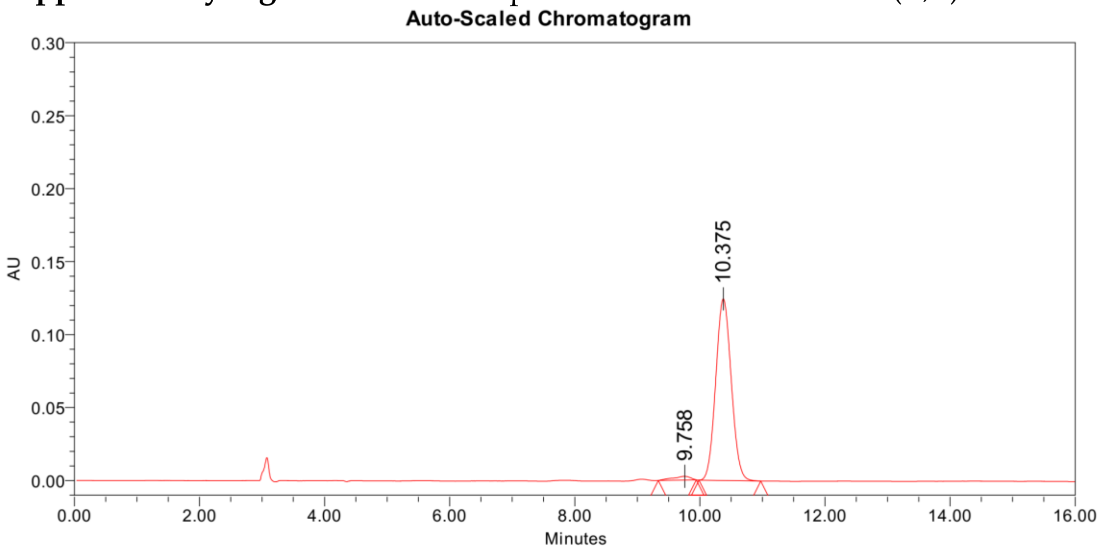

Peak Results

	Name	RT	Area	Height	% Area
1		9.758	49220	2534	2.24
2		10.375	2144482	124422	97.76

Supplementary Figure 185. HPLC Spectra of **12** obtained from (*S*,*R*)-**L1**.

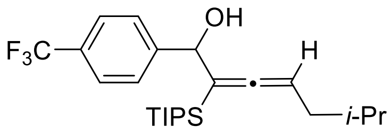

Figure 3, entry 13

(*R*,*S*)-**L1**: 94% ee; (*S*,*R*)-**L1**: 94% ee

Auto-Scaled Chromatogram

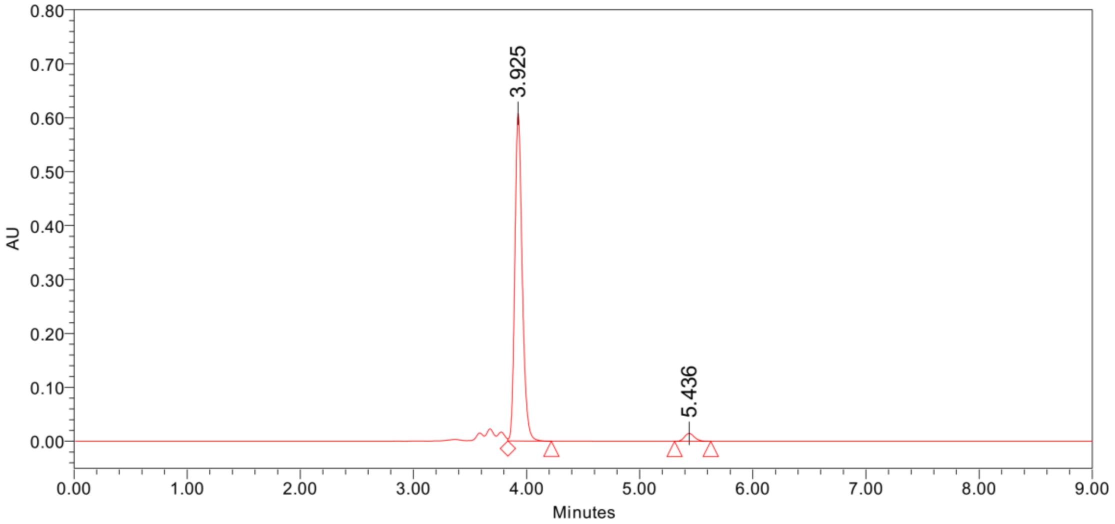

Peak Results

	Name	RT	Area	Height	% Area
1		3.925	2762210	607758	96.85
2		5.436	89911	14630	3.15

Supplementary Figure 186. HPLC Spectra of **13** obtained from (*R*,*S*)-**L1**.

Auto-Scaled Chromatogram

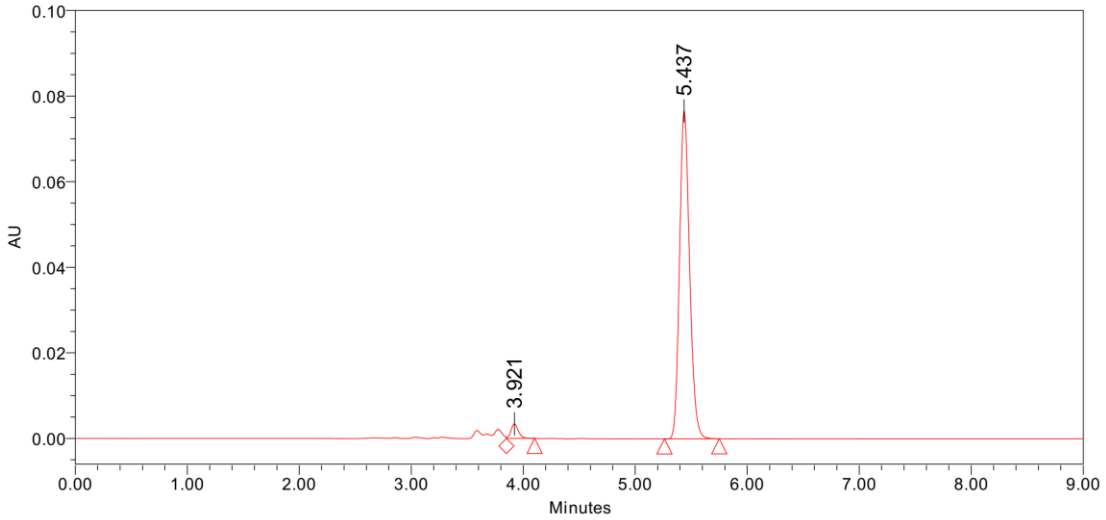

Peak Results

	Name	RT	Area	Height	% Area
1		3.921	15469	3396	3.12
2		5.437	479948	76658	96.88

Supplementary Figure 187. HPLC Spectra of **13** obtained from (*S*,*R*)-**L1**.

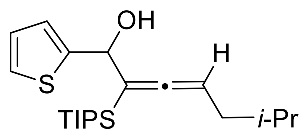

Figure 3, entry 14

(*R*,*S*)-**L1**: 97% ee; (*S*,*R*)-**L1**: 97% ee

Auto-Scaled Chromatogram

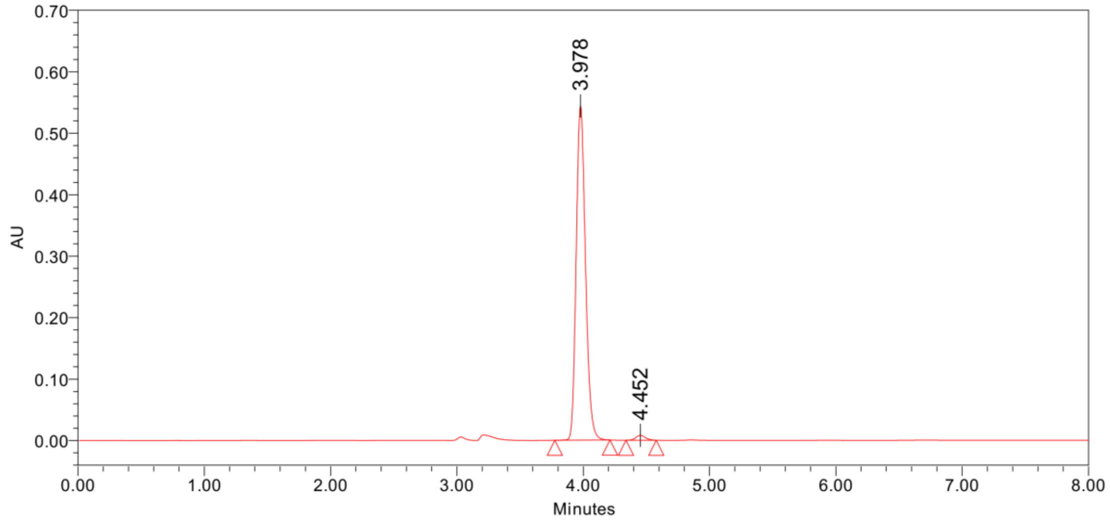

Peak Results

	Name	RT	Area	Height	% Area
1		3.978	2778864	543773	98.55
2		4.452	40983	7853	1.45

Supplementary Figure 188. HPLC Spectra of **14** obtained from (*R*,*S*)-**L1**.

Auto-Scaled Chromatogram

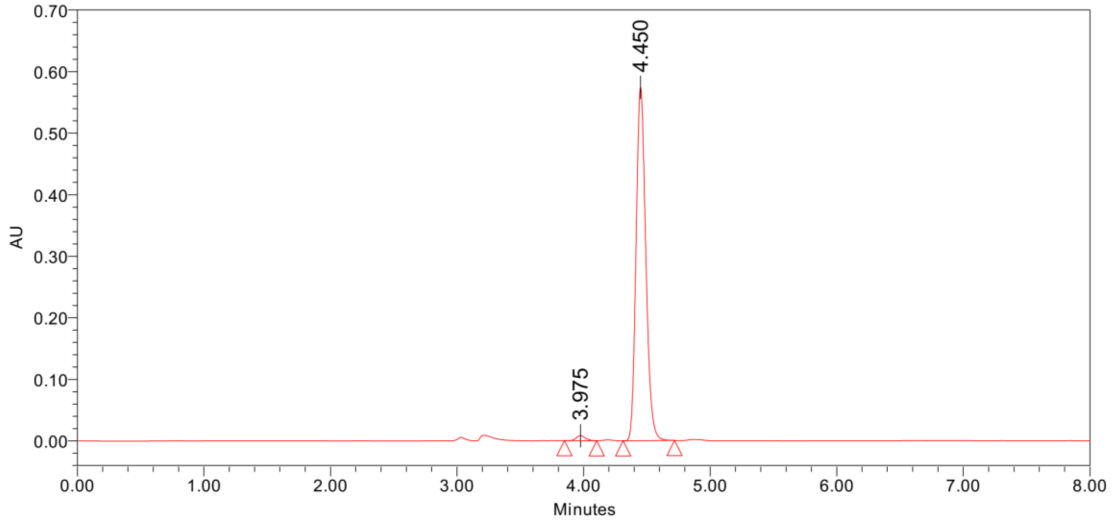

Peak Results

	Name	RT	Area	Height	% Area
1		3.975	39695	7897	1.28
2		4.450	3060442	574237	98.72

Supplementary Figure 189. HPLC Spectra of **14** obtained from (*S*,*R*)-**L1**.

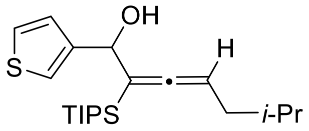

Figure 3, entry 15

(R,S)-**L1**: 95% ee; (S,R)-**L1**: 95% ee

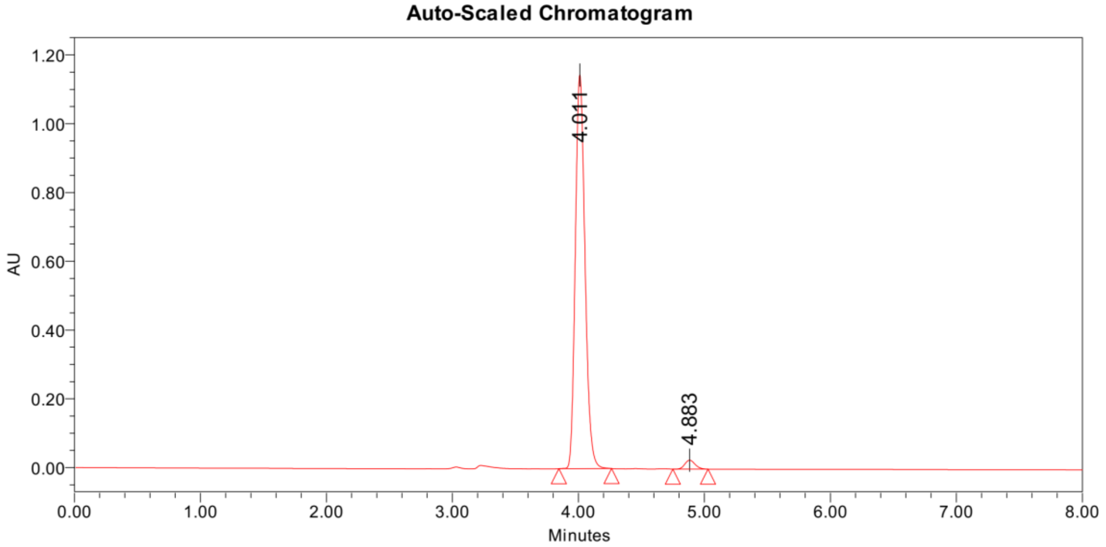

Peak Results

	Name	RT	Area	Height	% Area
1		4.011	6091470	1145033	97.64
2		4.883	147021	26076	2.36

Supplementary Figure 190. HPLC Spectra of **15** obtained from (R,S)-**L1**.

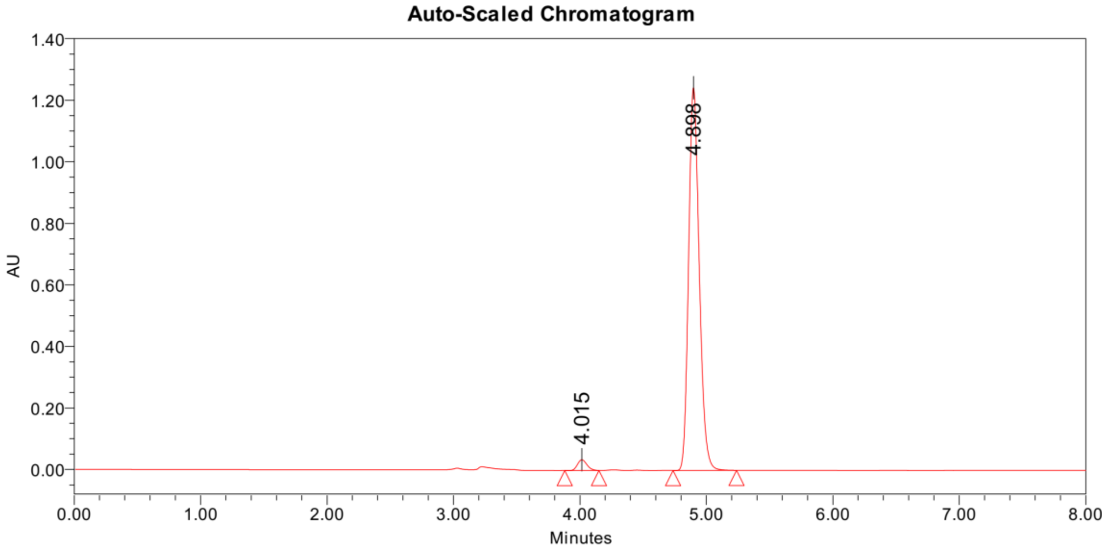

Peak Results

	Name	RT	Area	Height	% Area
1		4.015	180819	34868	2.43
2		4.898	7271602	1243900	97.57

Supplementary Figure 191. HPLC Spectra of **15** obtained from (S,R)-**L1**.

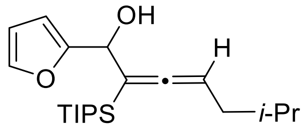

Figure 3, entry 16

(*R*,*S*)-**L1**: 88% ee; (*S*,*R*)-**L1**: 88% ee

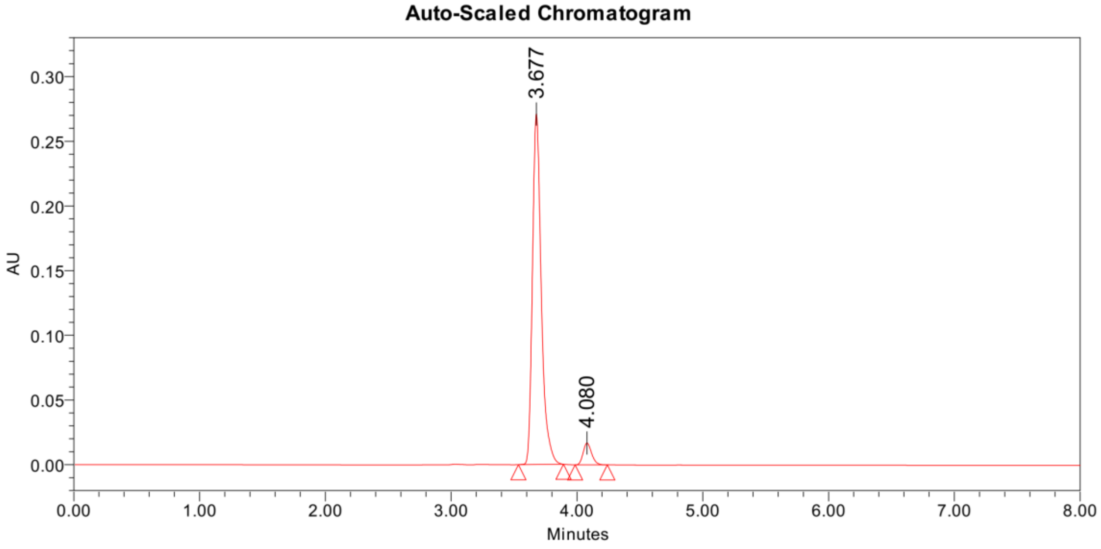

Peak Results

	Name	RT	Area	Height	% Area
1		3.677	1323230	271019	94.39
2		4.080	78673	16841	5.61

Supplementary Figure 192. HPLC Spectra of **16** obtained from (*R*,*S*)-**L1**.

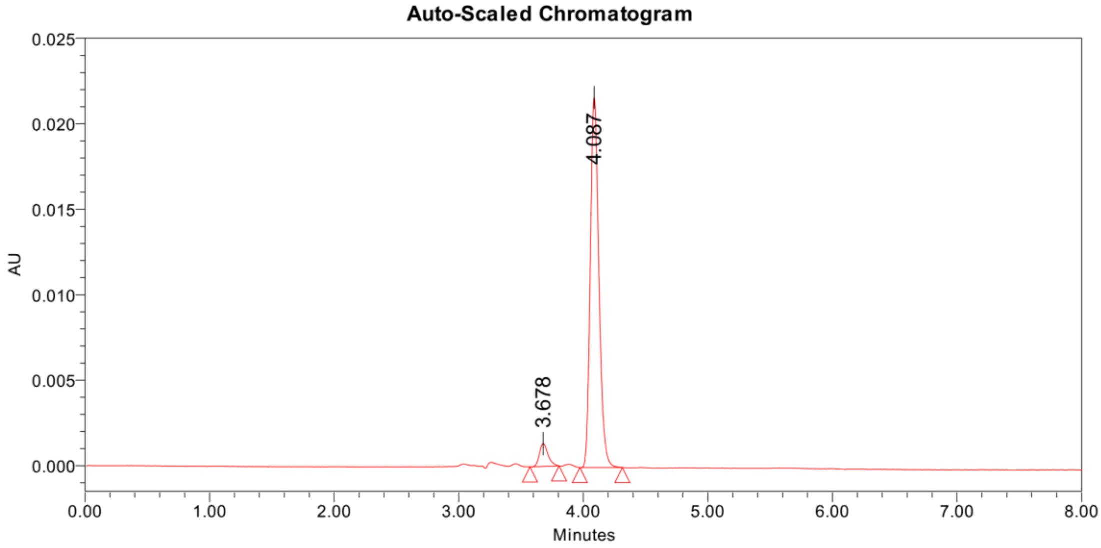

Peak Results

	Name	RT	Area	Height	% Area
1		3.678	6455	1343	5.86
2		4.087	103613	21655	94.14

Supplementary Figure 193. HPLC Spectra of **16** obtained from (*S*,*R*)-**L1**.

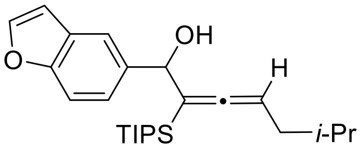

Figure 3, entry 17
(*R*,*S*)-**L1**: 92% ee; (*S*,*R*)-**L1**: 91% ee

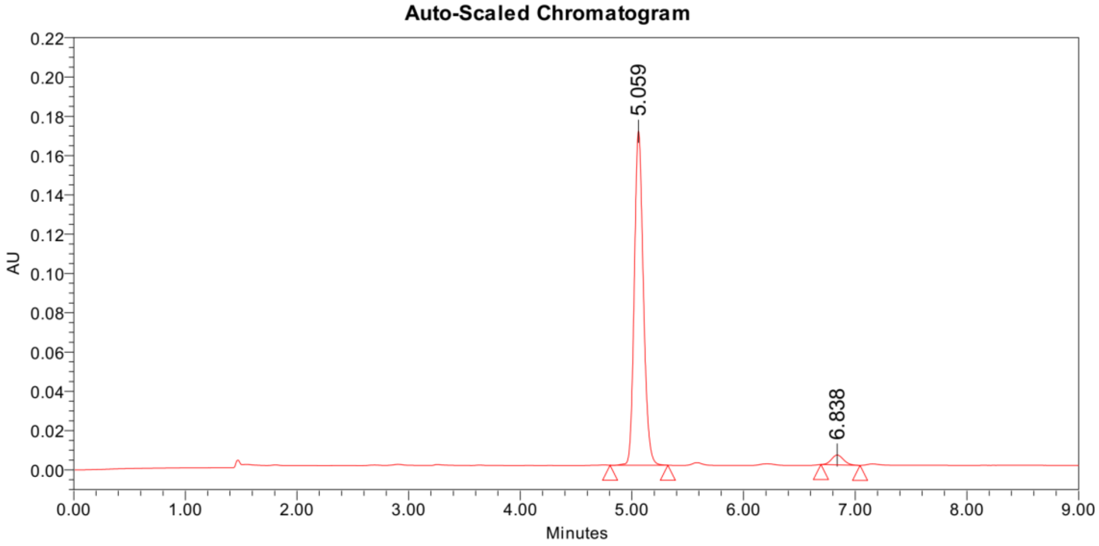

Peak Results

	Name	RT	Area	Height	% Area
1		5.059	961321	170134	96.27
2		6.838	37252	4987	3.73

Supplementary Figure 194. HPLC Spectra of **17** obtained from (*R*,*S*)-**L1**.

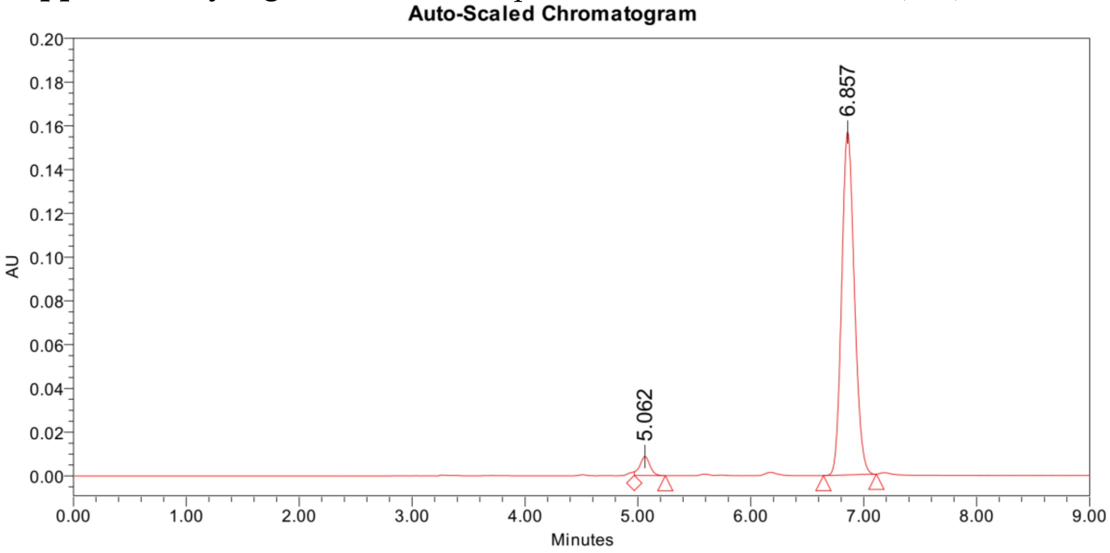

Peak Results

	Name	RT	Area	Height	% Area
1		5.062	54105	8686	4.22
2		6.857	1226927	156760	95.78

Supplementary Figure 195. HPLC Spectra of **17** obtained from (*S*,*R*)-**L1**.

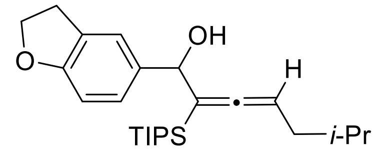

Figure 3, entry 18

(*R*,*S*)-**L1**: 94% ee; (*S*,*R*)-**L1**: 92% ee

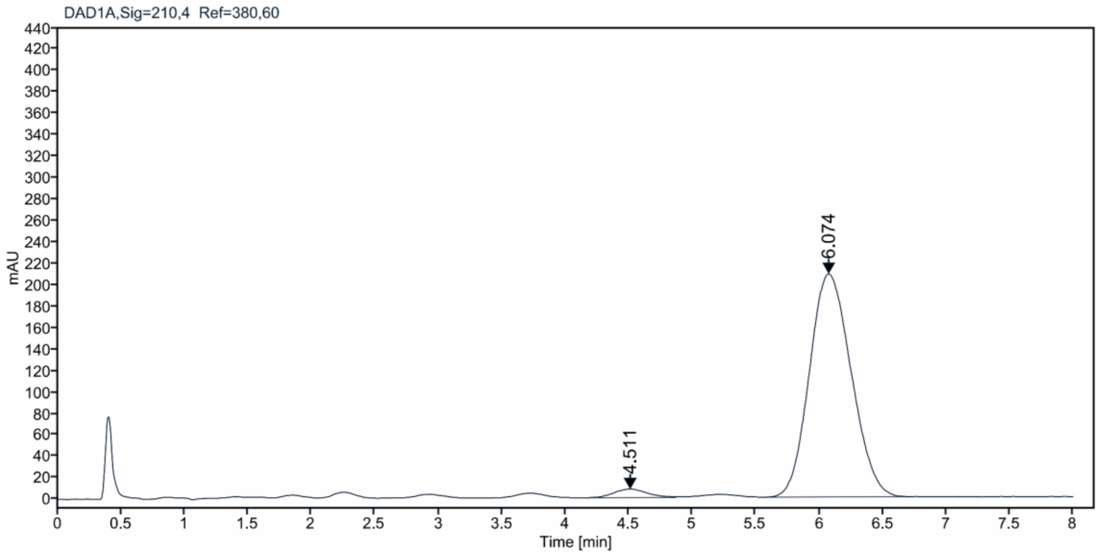

Signal: DAD1A,Sig=210,4 Ref=380,60

RT [min]	Type	Width (50%)	Area	Height	Area%
4.511	BV		147.7973	8.074	3.0048
6.074	BB		4770.8363	208.405	96.9952
		Sum	4918.6335		

Supplementary Figure 196. SFC Spectra of **18** obtained from (*R,S*)-**L1**.

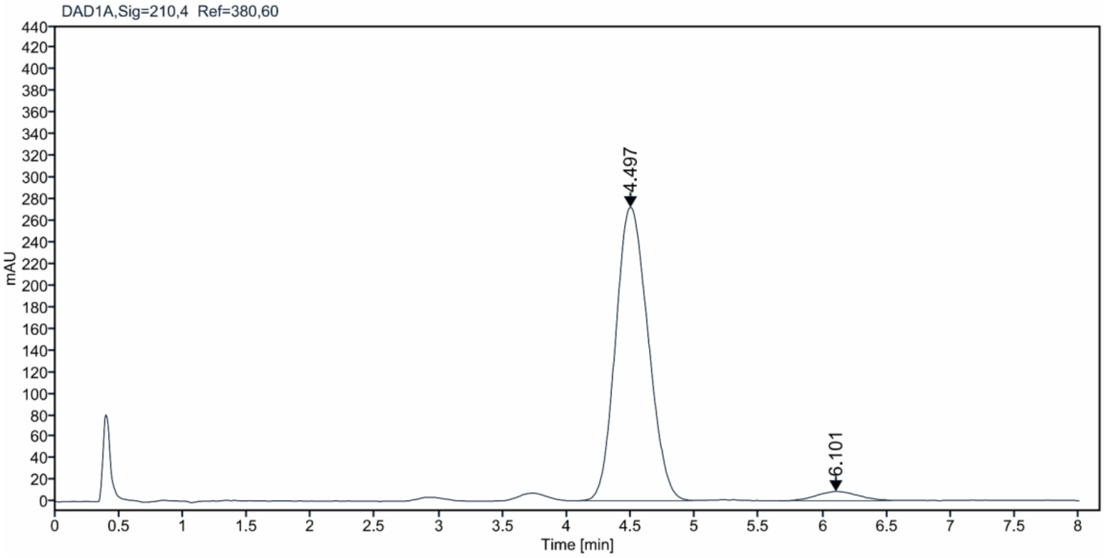

Signal: DAD1A,Sig=210,4 Ref=380,60

RT [min]	Type	Width (50%)	Area	Height	Area%
4.497	BV		4782.1791	271.513	96.1768
6.101	BV		190.0979	8.501	3.8232
		Sum	4972.2770		

Supplementary Figure 197. SFC Spectra of **18** obtained from (*S,R*)-**L1**.

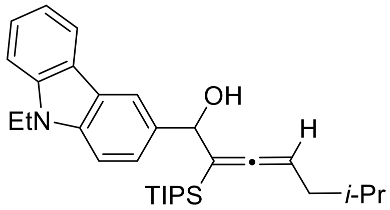

Figure 3, entry 19

(*R*,*S*)-**L1**: 94% ee; (*S*,*R*)-**L1**: 94% ee

Auto-Scaled Chromatogram

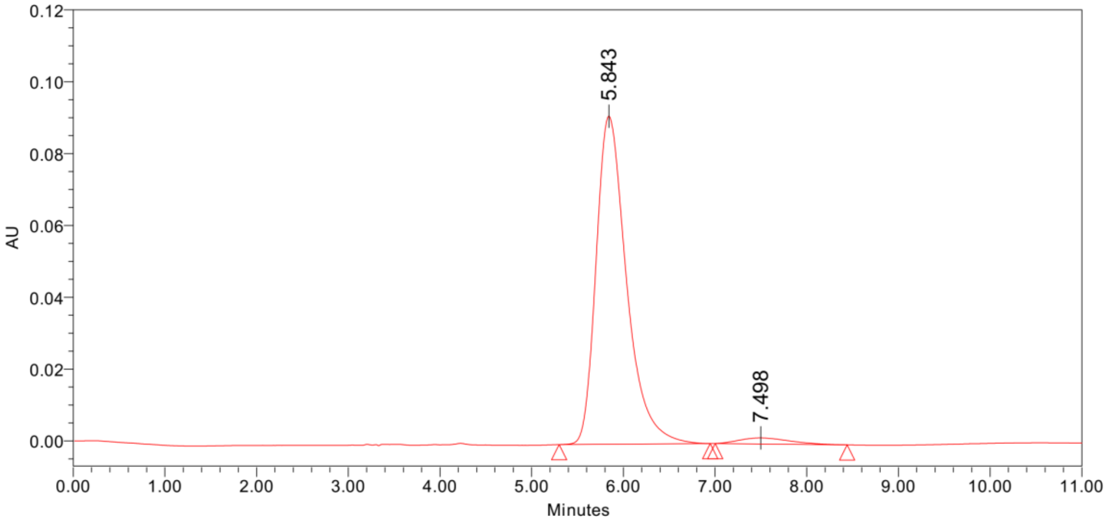

Peak Results

	Name	RT	Area	Height	% Area
1		5.843	2108361	91329	97.06
2		7.498	63766	1674	2.94

Supplementary Figure 198. HPLC Spectra of **19** obtained from (*R*,*S*)-**L1**.

Auto-Scaled Chromatogram

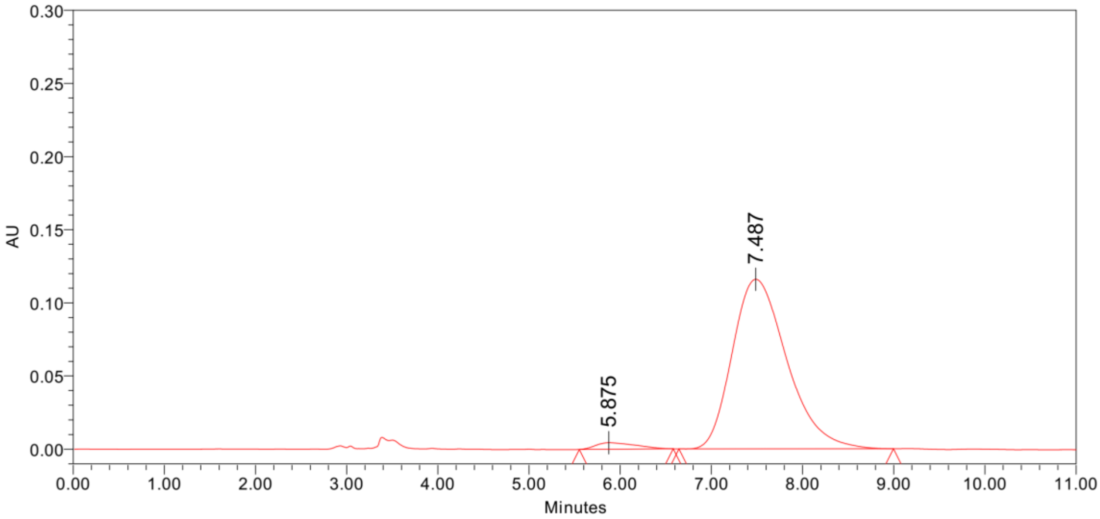

Peak Results

	Name	RT	Area	Height	% Area
1		5.875	135253	4463	2.72
2		7.487	4829121	115938	97.28

Supplementary Figure 199. HPLC Spectra of **19** obtained from (*S*,*R*)-**L1**.

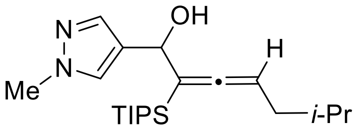

Figure 3, entry 20

(*R*,*S*)-**L1**: 96% ee; (*S*,*R*)-**L1**: 95% ee

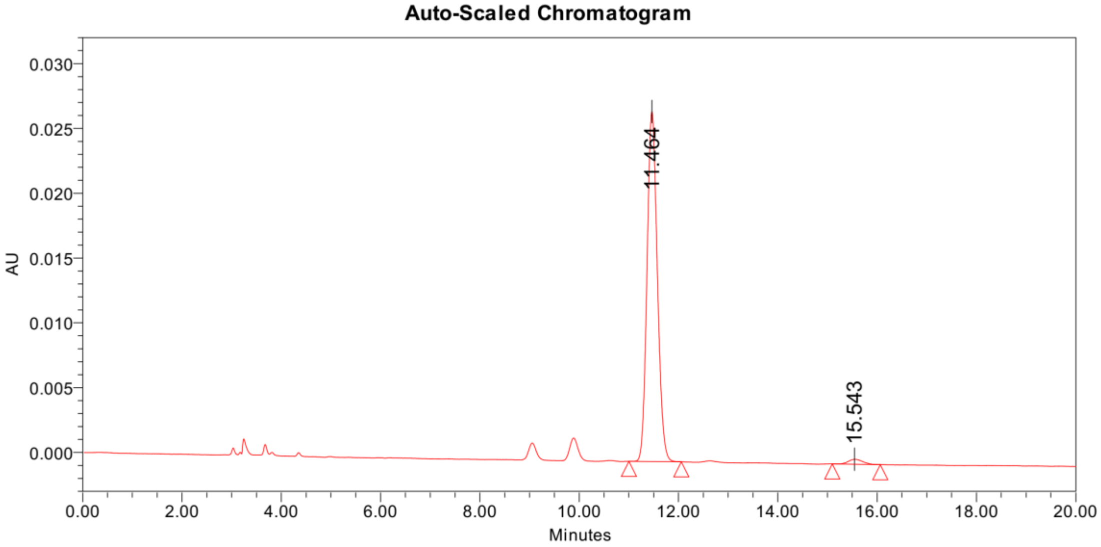

Peak Results

	Name	RT	Area	Height	% Area
1		11.464	389944	27009	98.10
2		15.543	7563	380	1.90

Supplementary Figure 200. HPLC Spectra of **20** obtained from (*R*,*S*)-**L1**.

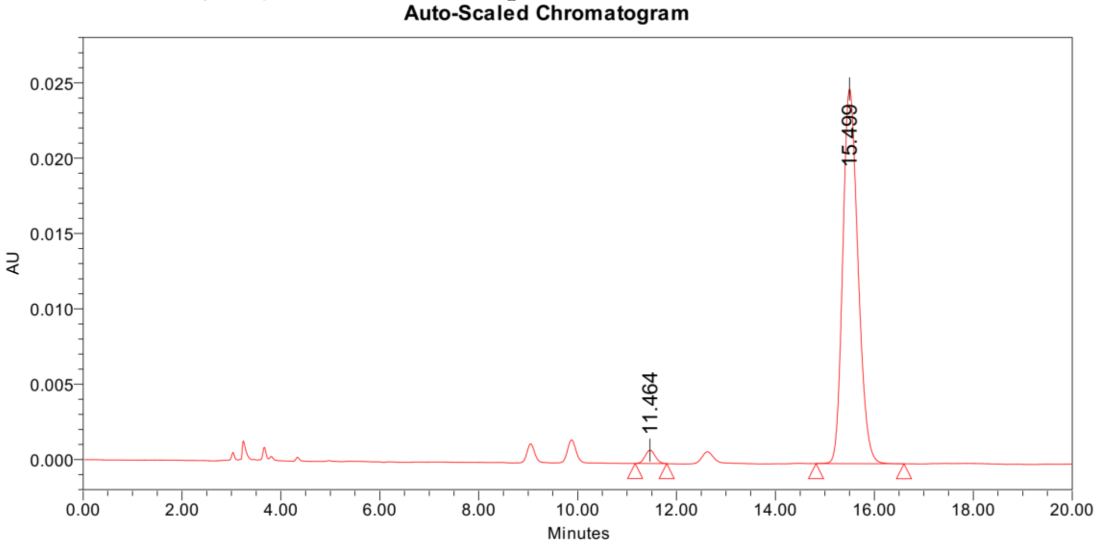

Peak Results

	Name	RT	Area	Height	% Area
1		11.464	12522	887	2.30
2		15.499	532516	24868	97.70

Supplementary Figure 201. HPLC Spectra of **20** obtained from (*S*,*R*)-**L1**.

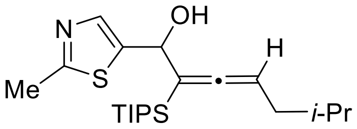

Figure 3, entry 21
(*R*,*S*)-**L1**: 94% ee; (*S*,*R*)-**L1**: 96% ee

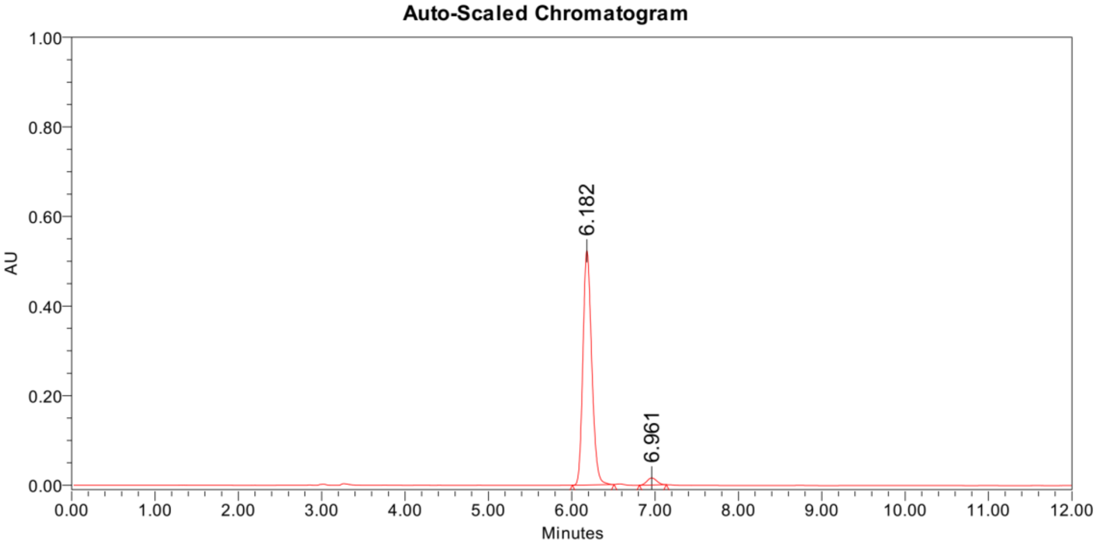

Peak Results

	Name	RT	Area	Height	% Area
1		6.182	3833387	523256	96.77
2		6.961	127826	15830	3.23

Supplementary Figure 202. HPLC Spectra of **21** obtained from (*R*,*S*)-**L1**.

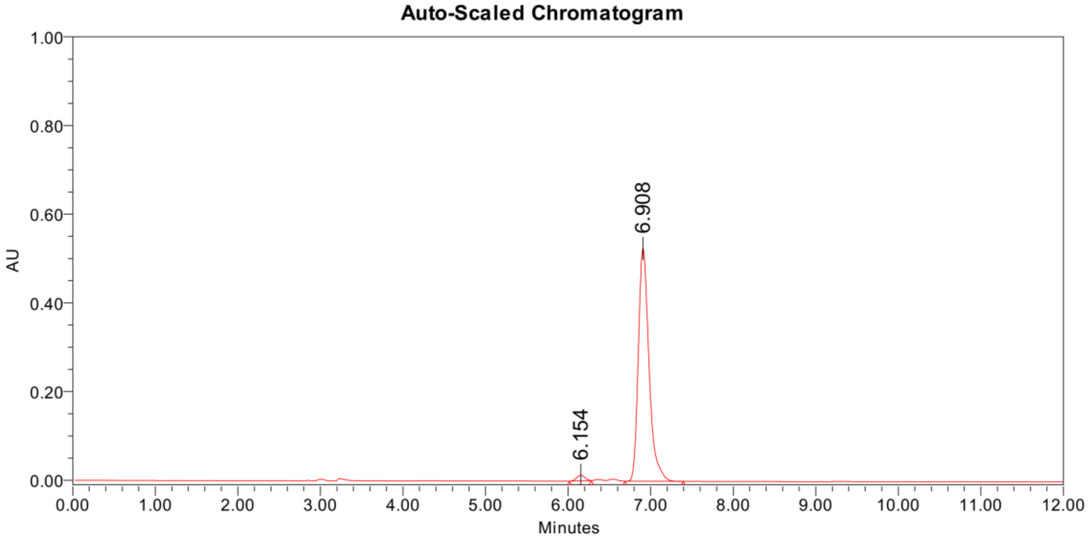

Peak Results

	Name	RT	Area	Height	% Area
1		6.154	86269	12525	1.79
2		6.908	4722592	525049	98.21

Supplementary Figure 203. HPLC Spectra of **21** obtained from (*S*,*R*)-**L1**.

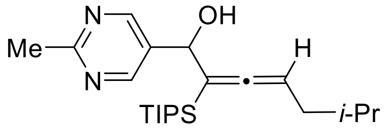

Figure 3, entry 22

(*R*,*S*)-**L1**: 94% ee; (*S*,*R*)-**L1**: 94% ee

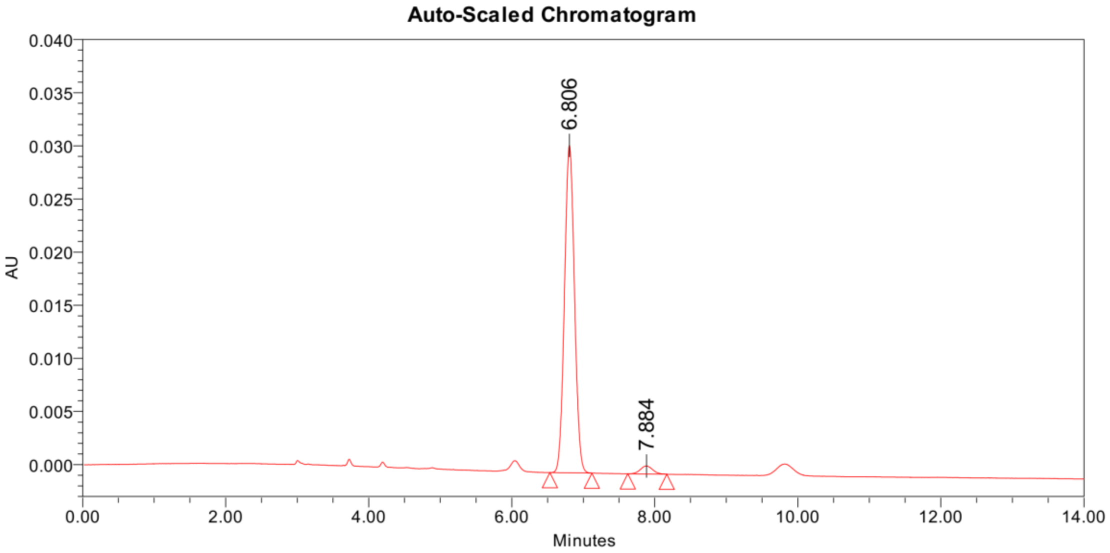

Peak Results

	Name	RT	Area	Height	% Area
1		6.806	299250	30813	97.09
2		7.884	8974	746	2.91

Supplementary Figure 204. HPLC Spectra of **22** obtained from (*R*,*S*)-**L1**.

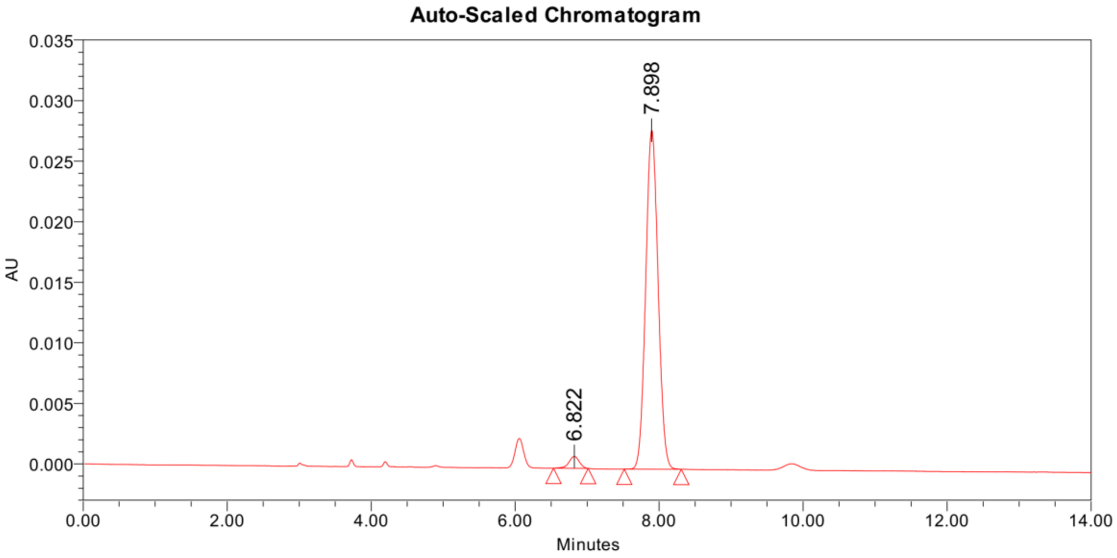

Peak Results

	Name	RT	Area	Height	% Area
1		6.822	9885	986	2.87
2		7.898	335068	27999	97.13

Supplementary Figure 205. HPLC Spectra of **22** obtained from (*S*,*R*)-**L1**.

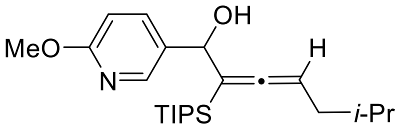

Figure 3, entry 23
(*R*,*S*)-**L1**: 94% ee; (*S*,*R*)-**L1**: 94% ee

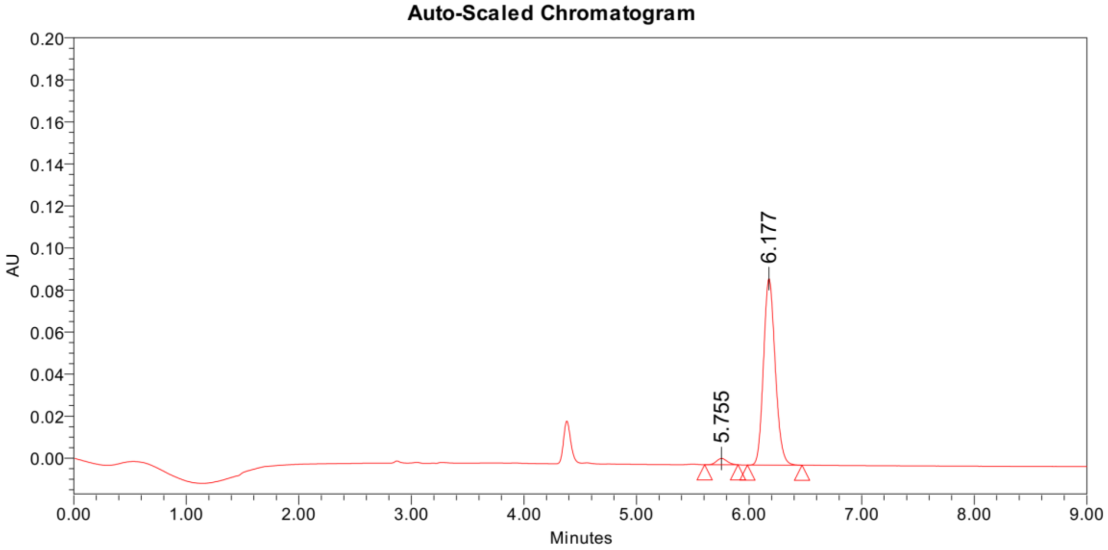

Peak Results

	Name	RT	Area	Height	% Area
1		5.755	19773	2982	2.99
2		6.177	642229	88734	97.01

Supplementary Figure 206. HPLC Spectra of **23** obtained from (*R*,*S*)-**L1**.

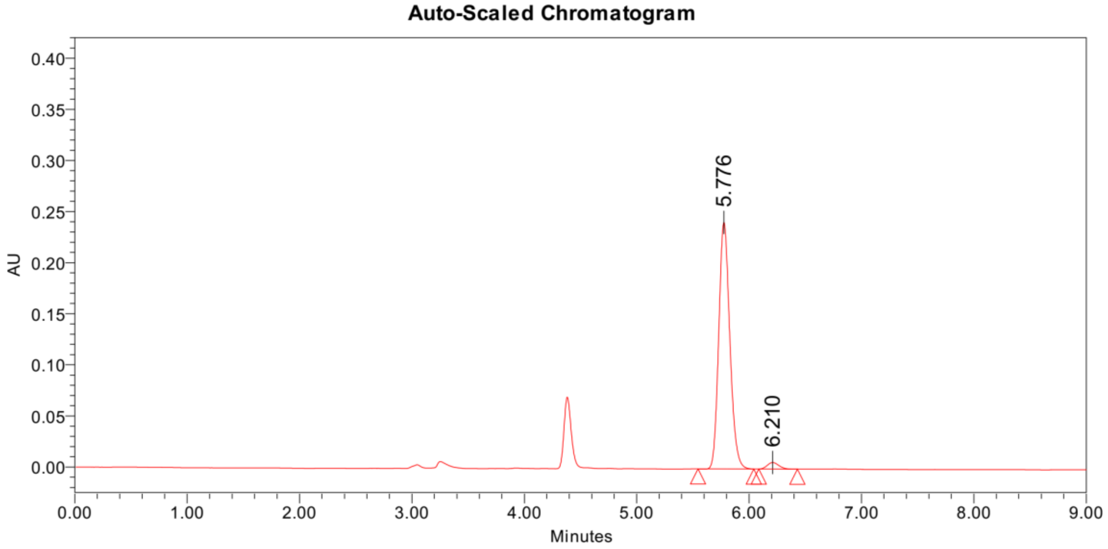

Peak Results

	Name	RT	Area	Height	% Area
1		5.776	1649670	241316	97.26
2		6.210	46388	6487	2.74

Supplementary Figure 207. HPLC Spectra of **23** obtained from (*S*,*R*)-**L1**.

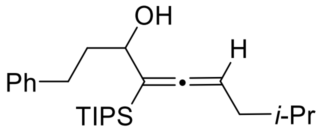

Figure 3, entry 24
(R,S)-**L1**: 88% ee; (S,R)-**L1**: 86% ee

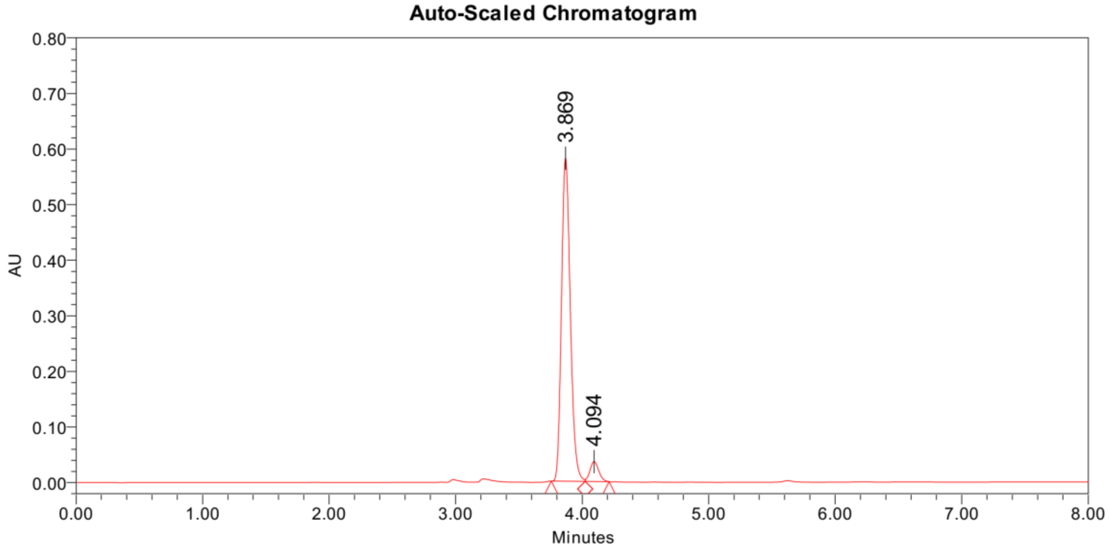

Peak Results

	Name	RT	Area	Height	% Area
1		3.869	2816400	581217	94.26
2		4.094	171568	35814	5.74

Supplementary Figure 208. HPLC Spectra of **24** obtained from (*R*,*S*)-**L1**.

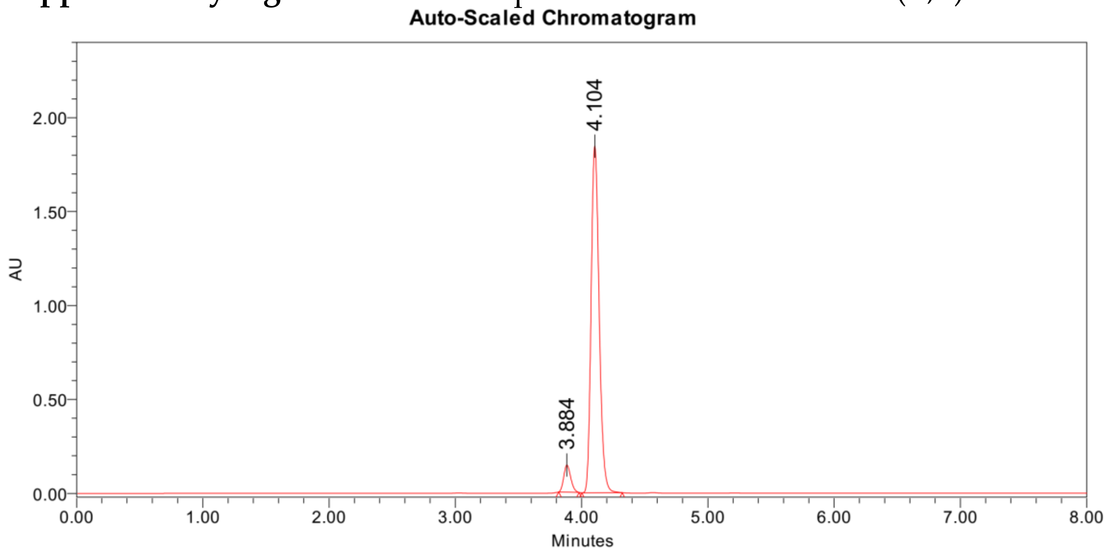

Peak Results

	Name	RT	Area	Height	% Area
1		3.884	554935	144307	6.39
2		4.104	8136088	1845488	93.61

Supplementary Figure 209. HPLC Spectra of **24** obtained from (*S*,*R*)-**L1**.

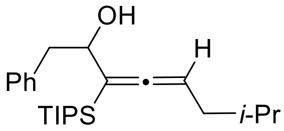

Figure 3, entry 25

(*R*,*S*)-**L1**: 88% ee; (*S*,*R*)-**L1**: 88% ee

Auto-Scaled Chromatogram

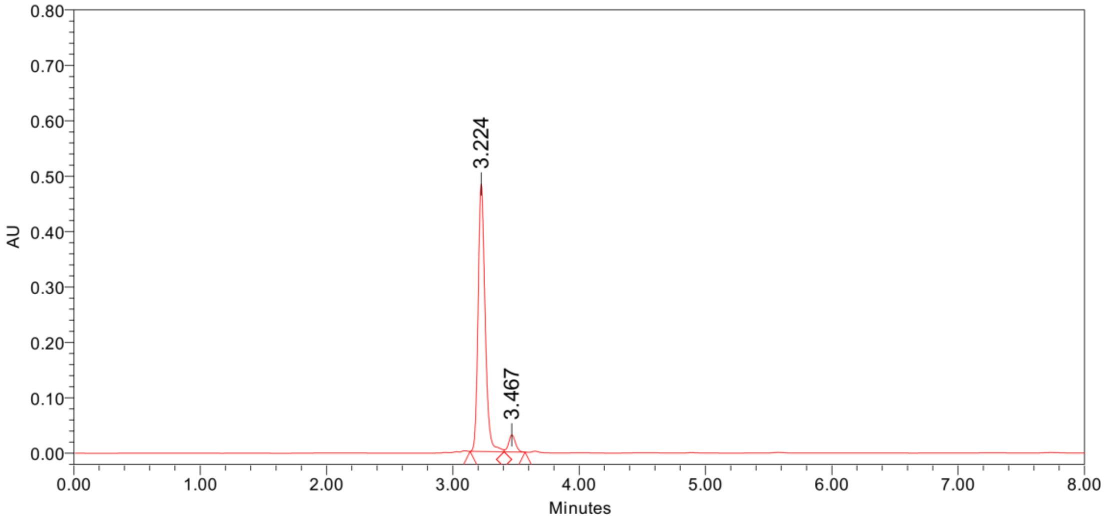

Peak Results

	Name	RT	Area	Height	% Area
1		3.224	1853421	483208	94.13
2		3.467	115491	30822	5.87

Supplementary Figure 210. HPLC Spectra of **25** obtained from (*R*,*S*)-**L1**.

Auto-Scaled Chromatogram

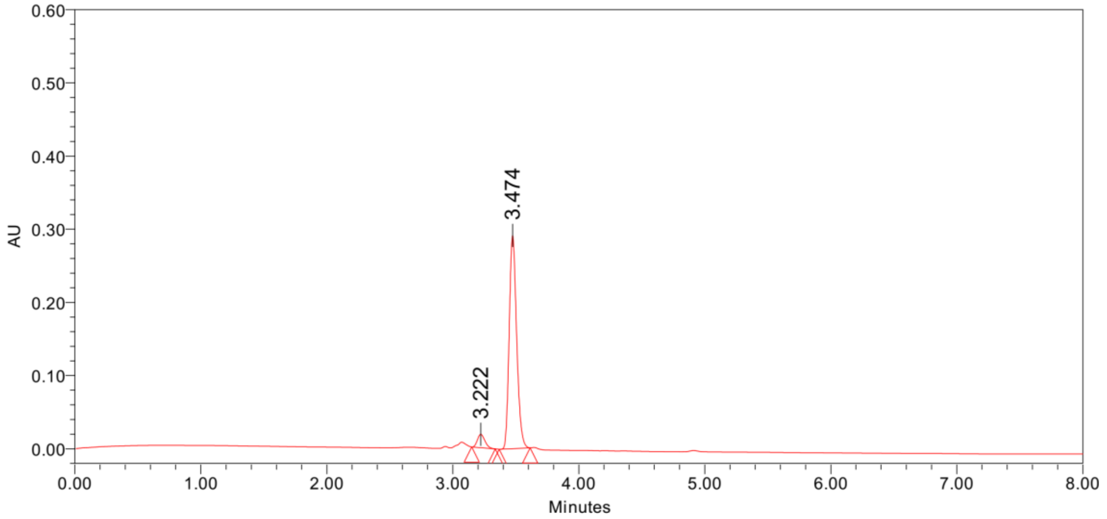

Peak Results

	Name	RT	Area	Height	% Area
1		3.222	75601	18325	5.97
2		3.474	1191459	291538	94.03

Supplementary Figure 211. HPLC Spectra of **25** obtained from (*S*,*R*)-**L1**.

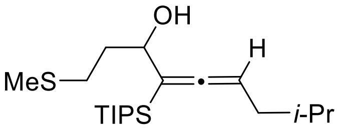

Figure 3, entry 26

(*R*,*S*)-**L1**: 80% ee; (*S*,*R*)-**L1**: 78% ee

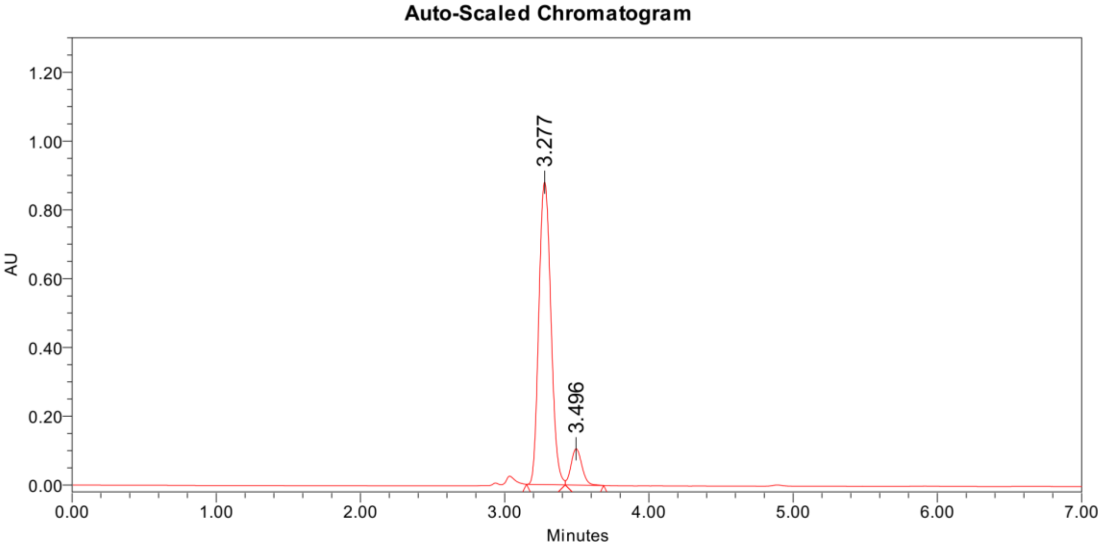

Peak Results

	Name	RT	Area	Height	% Area
1		3.277	5096379	879144	90.20
2		3.496	553444	105353	9.80

Supplementary Figure 212. HPLC Spectra of **26** obtained from (*R*,*S*)-**L1**.

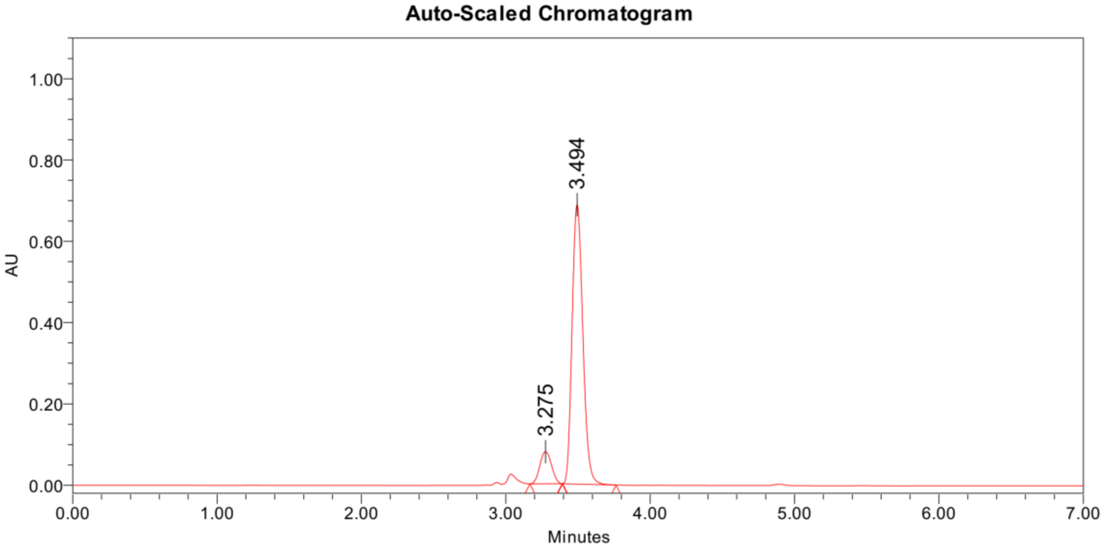

Peak Results

	Name	RT	Area	Height	% Area
1		3.275	444515	78889	11.09
2		3.494	3563489	687716	88.91

Supplementary Figure 213. HPLC Spectra of **26** obtained from (*S*,*R*)-**L1**.

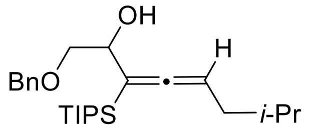

Figure 3, entry 27

(*R*,*S*)-**L1**: 82% ee; (*S*,*R*)-**L1**: 80% ee

Auto-Scaled Chromatogram

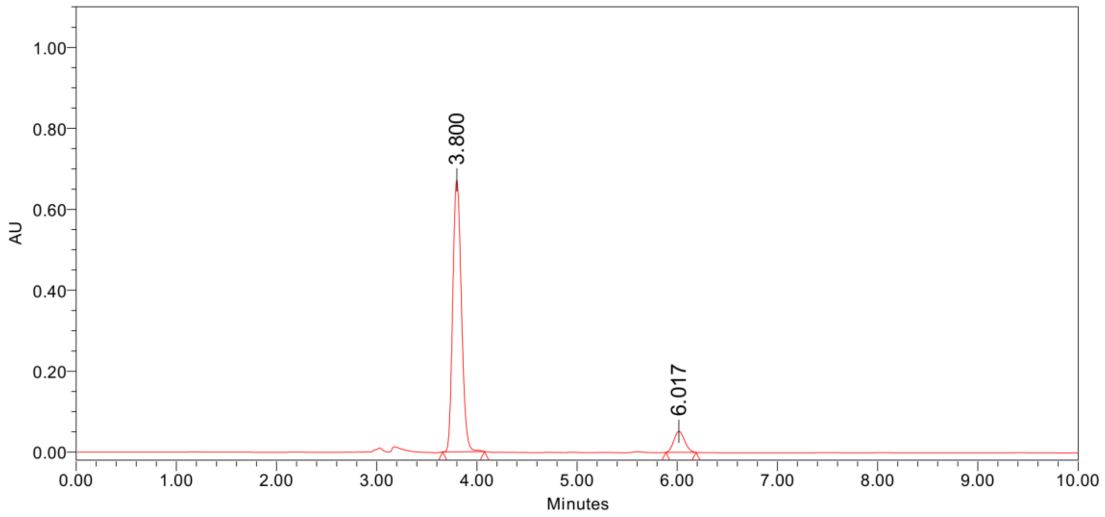

Peak Results

	Name	RT	Area	Height	% Area
1		3.800	4022995	671844	91.28
2		6.017	384109	51679	8.72

Supplementary Figure 214. HPLC Spectra of **27** obtained from (*R*,*S*)-**L1**.

Auto-Scaled Chromatogram

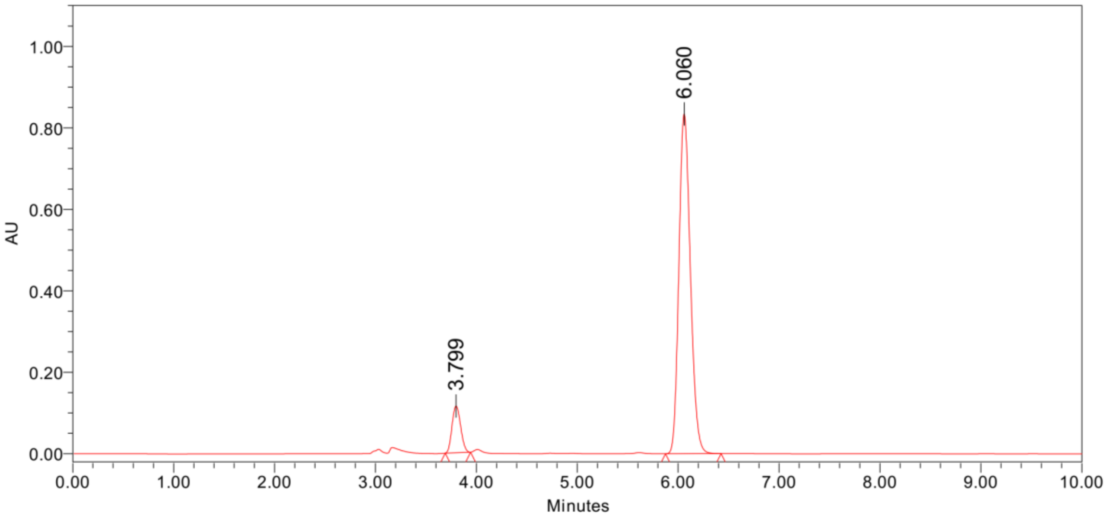

Peak Results

	Name	RT	Area	Height	% Area
1		3.799	682311	114539	9.39
2		6.060	6587074	834229	90.61

Supplementary Figure 215. HPLC Spectra of **27** obtained from (*S*,*R*)-**L1**.

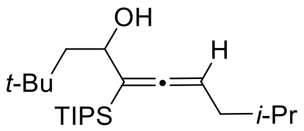

Figure 3, entry 28
(*R*,*S*)-**L1**: 82% ee; (*S*,*R*)-**L1**: 83% ee

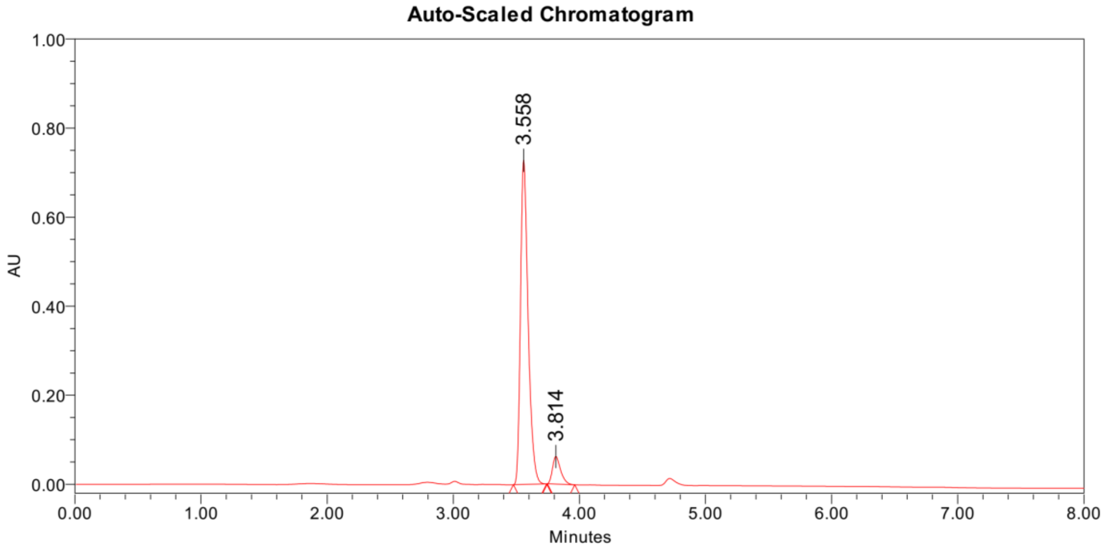

Peak Results

	Name	RT	Area	Height	% Area
1		3.558	3067036	728275	91.69
2		3.814	277936	61949	8.31

Supplementary Figure 216. HPLC Spectra of **28** obtained from (*R*,*S*)-**L1**.

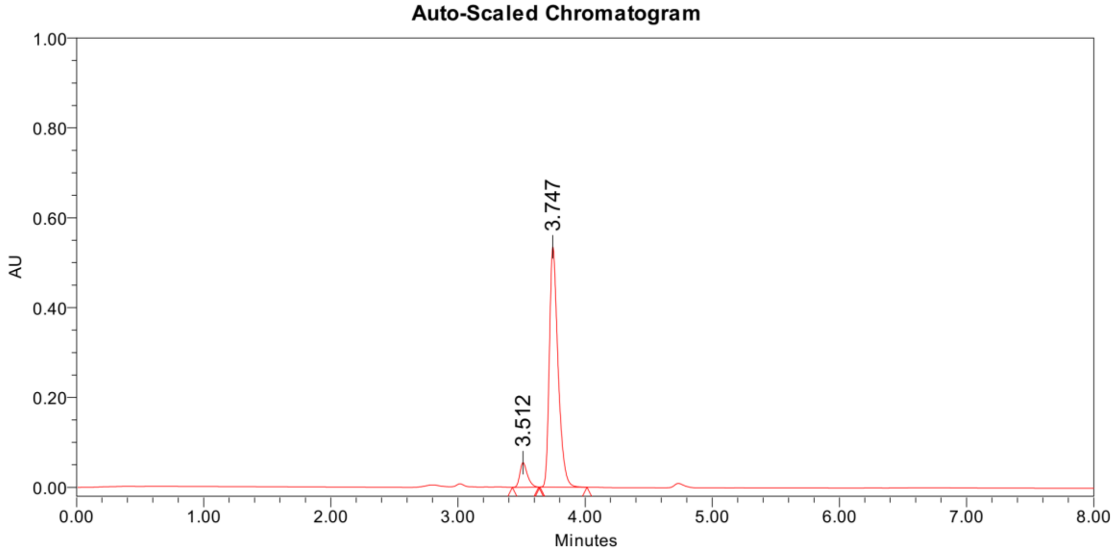

Peak Results

	Name	RT	Area	Height	% Area
1		3.512	226156	54670	8.18
2		3.747	2539961	534906	91.82

Supplementary Figure 217. HPLC Spectra of **28** obtained from (*S*,*R*)-**L1**.

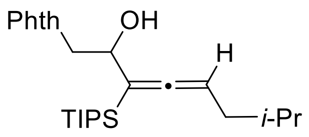

Figure 3, entry 29

(R,S)-**L1**: 89% ee; (S,R)-**L1**: 87% ee

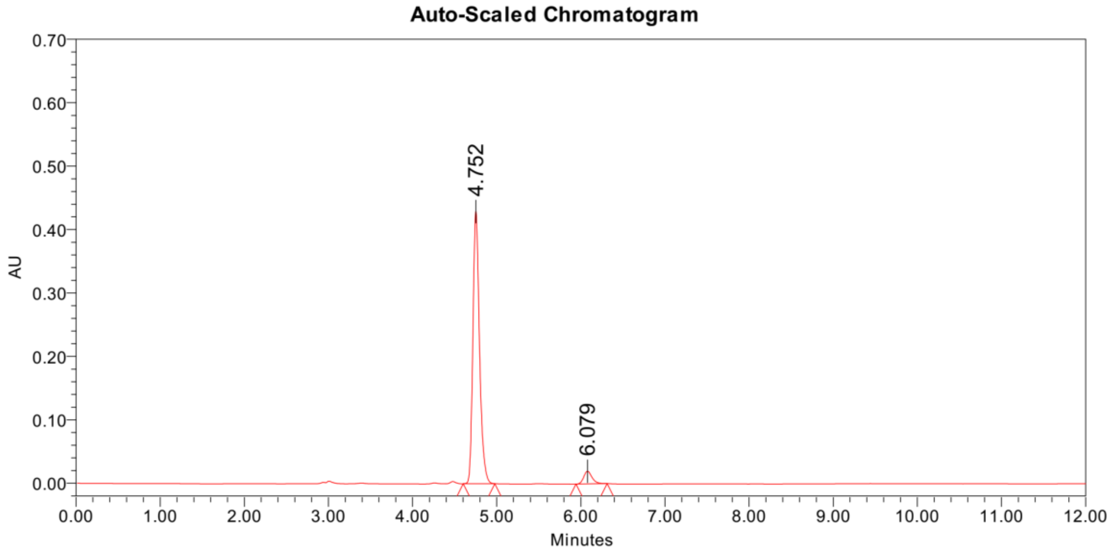

Peak Results

	Name	RT	Area	Height	% Area
1		4.752	2340367	429690	94.53
2		6.079	135523	19907	5.47

Supplementary Figure 218. HPLC Spectra of **29** obtained from (R,S)-**L1**.

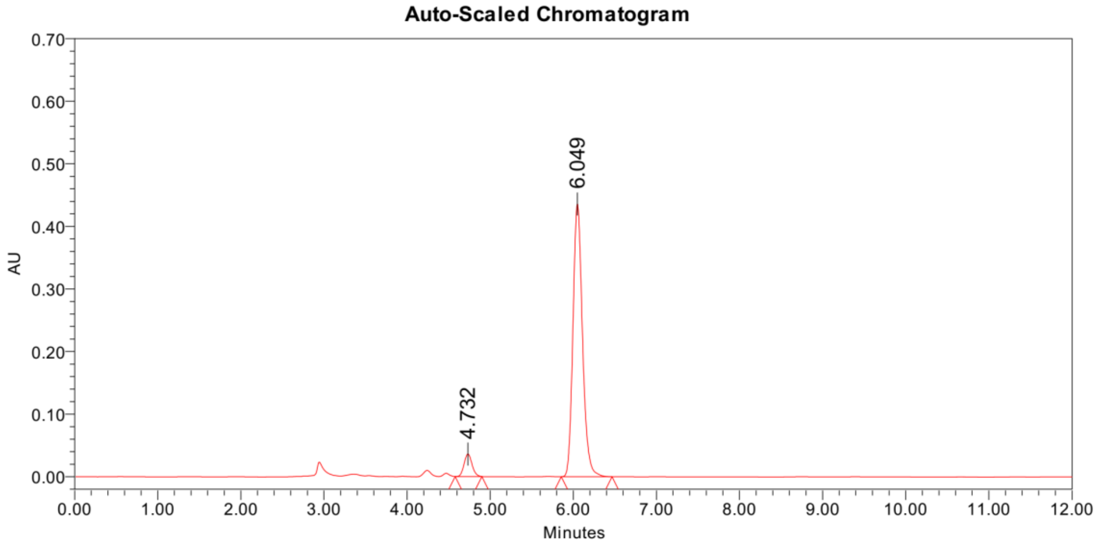

Peak Results

	Name	RT	Area	Height	% Area
1		4.732	240010	35988	6.46
2		6.049	3477076	436010	93.54

Supplementary Figure 219. HPLC Spectra of **29** obtained from (S,R)-**L1**.

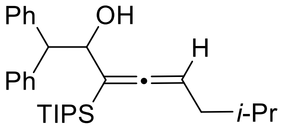

Figure 3, entry 30

(*R*,*S*)-**L1**: 96% ee; (*S*,*R*)-**L1**: 94% ee

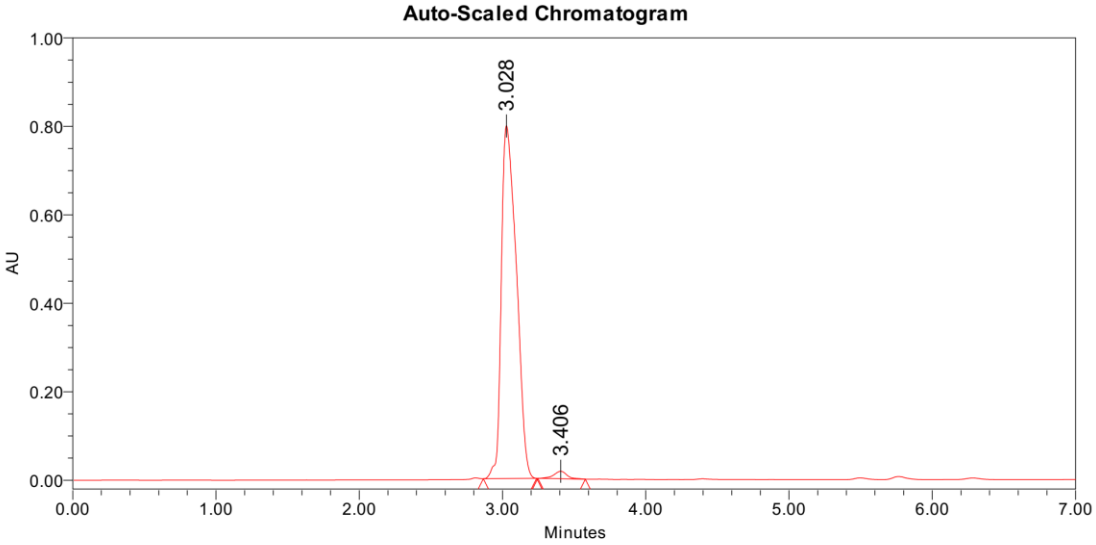

Peak Results

	Name	RT	Area	Height	% Area
1		3.028	5804205	797608	98.16
2		3.406	108933	16912	1.84

Supplementary Figure 220. HPLC Spectra of **30** obtained from (*R*,*S*)-**L1**.

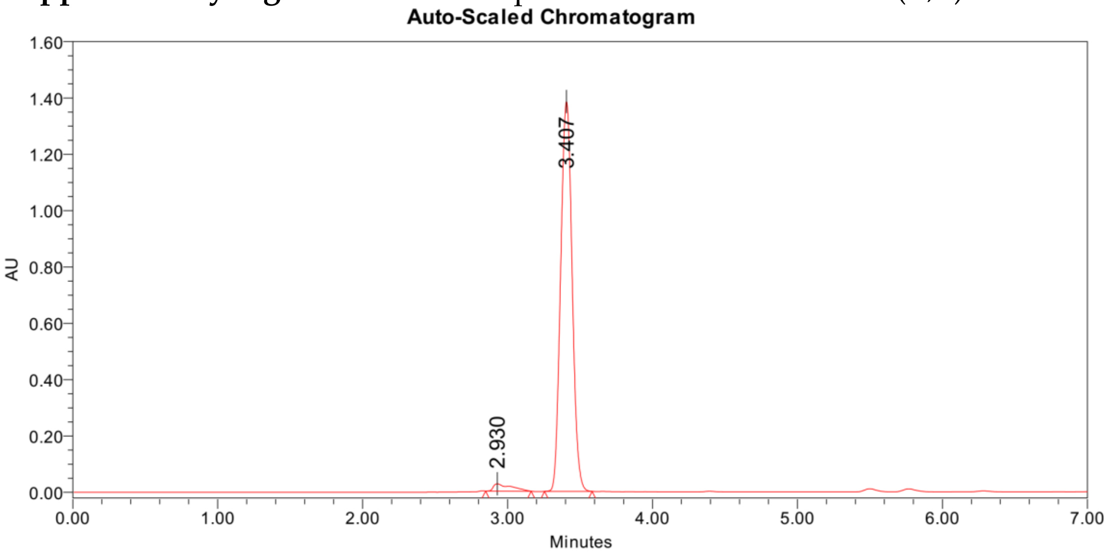

Peak Results

	Name	RT	Area	Height	% Area
1		2.930	219914	25815	2.81
2		3.407	7610462	1383978	97.19

Supplementary Figure 221. HPLC Spectra of **30** obtained from (*S*,*R*)-**L1**.

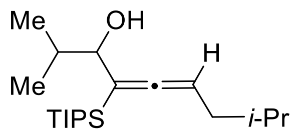

Figure 3, entry 31

(*R*,*S*)-**L1**: 96% ee; (*S*,*R*)-**L1**: 96% ee

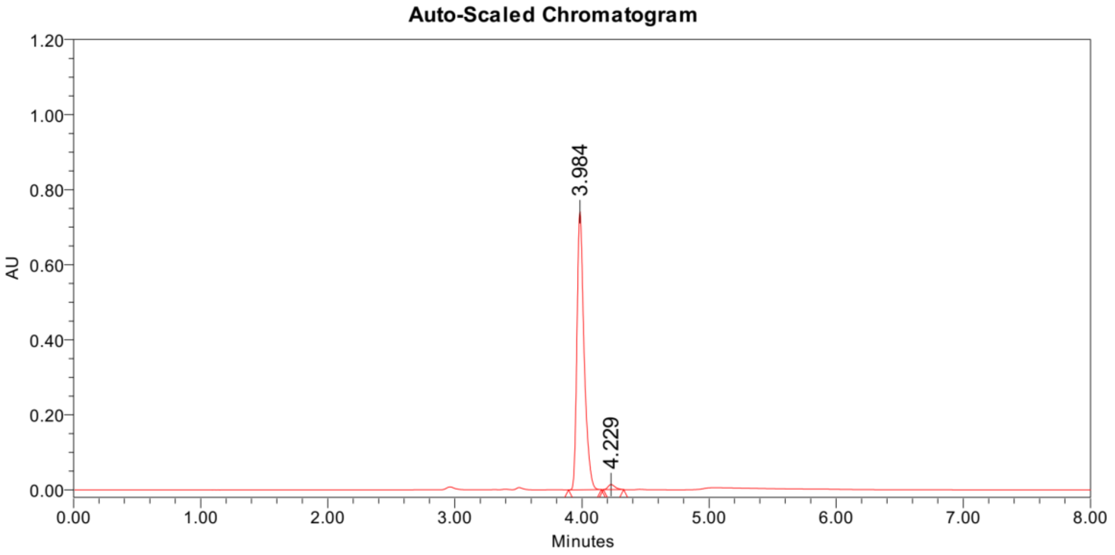

Peak Results

	Name	RT	Area	Height	% Area
1		3.984	2889046	741354	98.22
2		4.229	52353	13611	1.78

Supplementary Figure 222. HPLC Spectra of **31** obtained from (*R*,*S*)-**L1**.

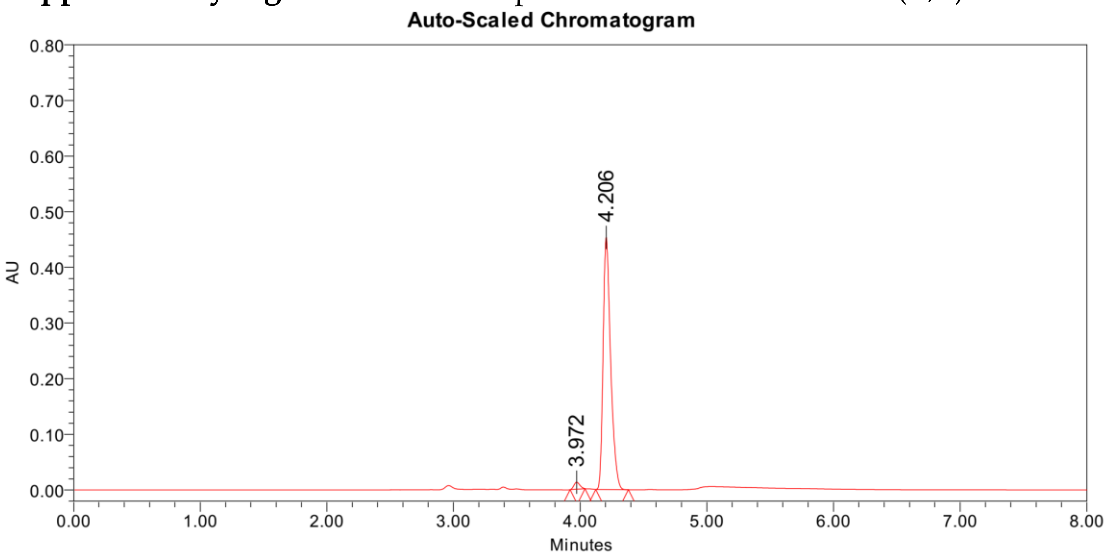

Peak Results

	Name	RT	Area	Height	% Area
1		3.972	41063	12665	2.15
2		4.206	1870039	453416	97.85

Supplementary Figure 223. HPLC Spectra of **31** obtained from (*S*,*R*)-**L1**.

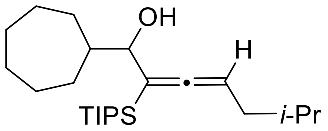

Figure 3, entry 32

(*R*,*S*)-**L1**: 97% ee; (*S*,*R*)-**L1**: 98% ee

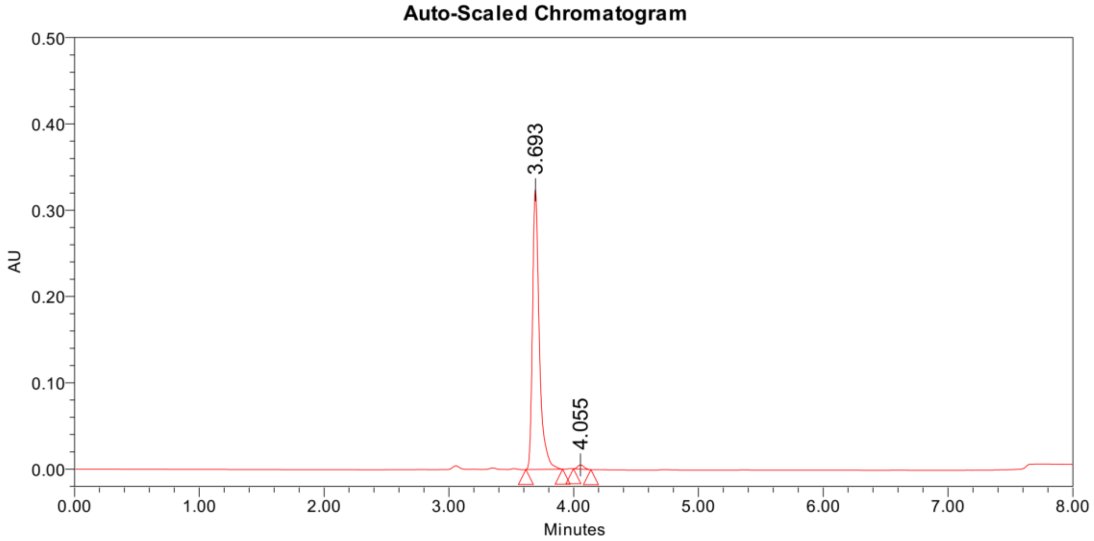

Peak Results

	Name	RT	Area	Height	% Area
1		3.693	1235430	324163	98.62
2		4.055	17307	4957	1.38

Supplementary Figure 224. HPLC Spectra of **32** obtained from (*R*,*S*)-**L1**.

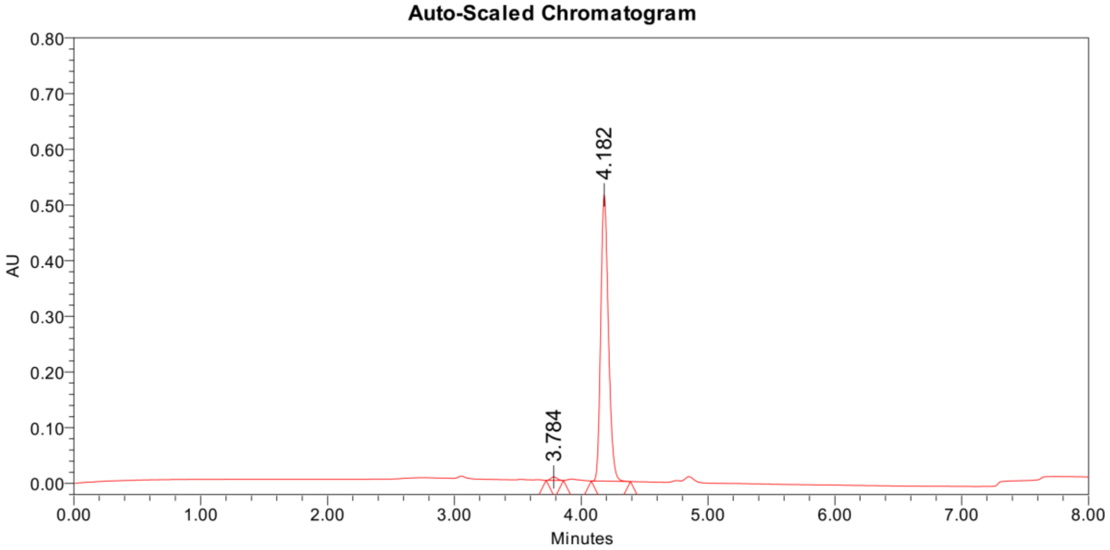

Peak Results

	Name	RT	Area	Height	% Area
1		3.784	20913	6171	0.96
2		4.182	2158633	514476	99.04

Supplementary Figure 225. HPLC Spectra of **32** obtained from (*S*,*R*)-**L1**.

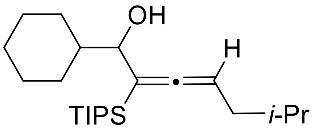

Figure 3, entry 33

(*R*,*S*)-**L1**: 94% ee; (*S*,*R*)-**L1**: 95% ee

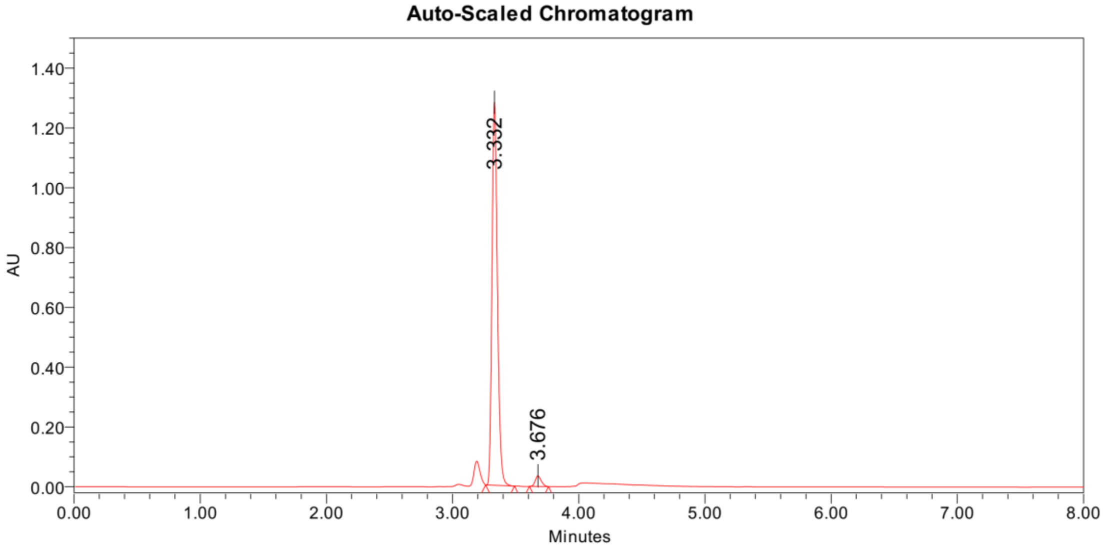

Peak Results

	Name	RT	Area	Height	% Area
1		3.332	3867018	1281354	97.05
2		3.676	117583	35612	2.95

Supplementary Figure 226. HPLC Spectra of **33** obtained from (*R*,*S*)-**L1**.

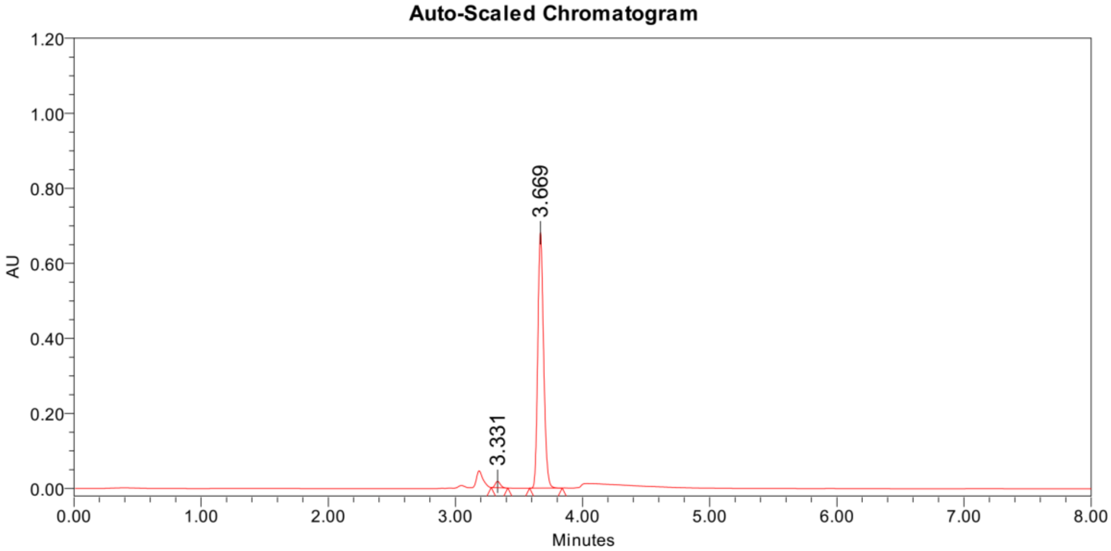

Peak Results

	Name	RT	Area	Height	% Area
1		3.331	47848	17260	2.16
2		3.669	2166605	681775	97.84

Supplementary Figure 227. HPLC Spectra of **33** obtained from (*S*,*R*)-**L1**.

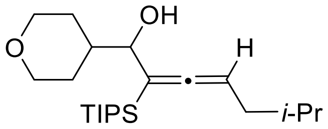

Figure 3, entry 34

(*R*,*S*)-**L1**: 92% ee; (*S*,*R*)-**L1**: 94% ee

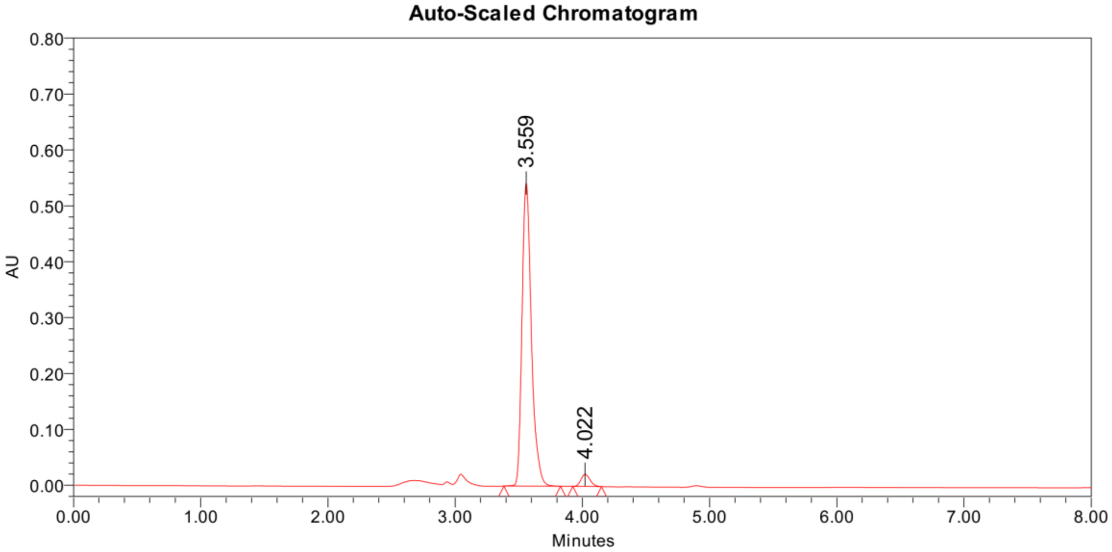

Peak Results

	Name	RT	Area	Height	% Area
1		3.559	2770711	542054	96.23
2		4.022	108692	21767	3.77

Supplementary Figure 228. HPLC Spectra of **34** obtained from (*R*,*S*)-**L1**.

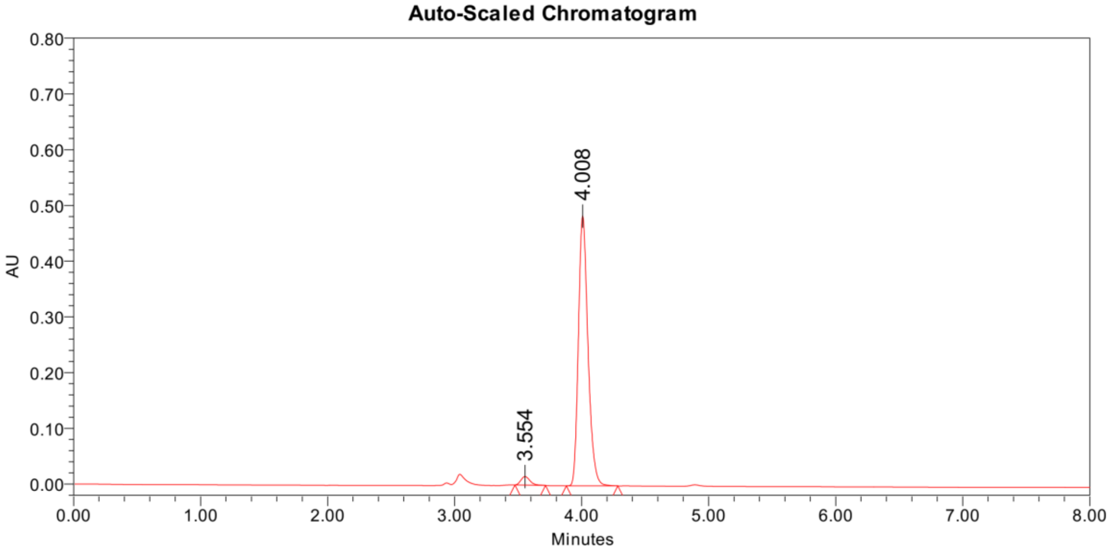

Peak Results

	Name	RT	Area	Height	% Area
1		3.554	76089	15279	2.91
2		4.008	2540891	484206	97.09

Supplementary Figure 229. HPLC Spectra of **34** obtained from (*S*,*R*)-**L1**.

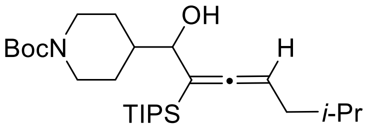

Figure 3, entry 35
(*R*,*S*)-**L1**: 92% ee; (*S*,*R*)-**L1**: 92% ee

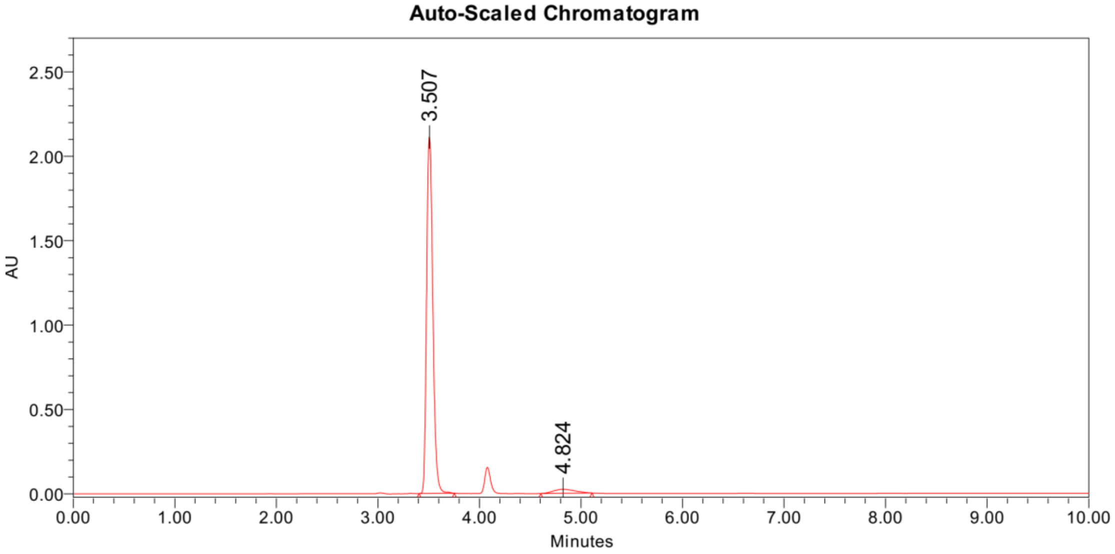

Peak Results

	Name	RT	Area	Height	% Area
1		3.507	9016936	2113154	96.24
2		4.824	352403	23610	3.76

Supplementary Figure 230. HPLC Spectra of **35** obtained from (*R*,*S*)-**L1**.

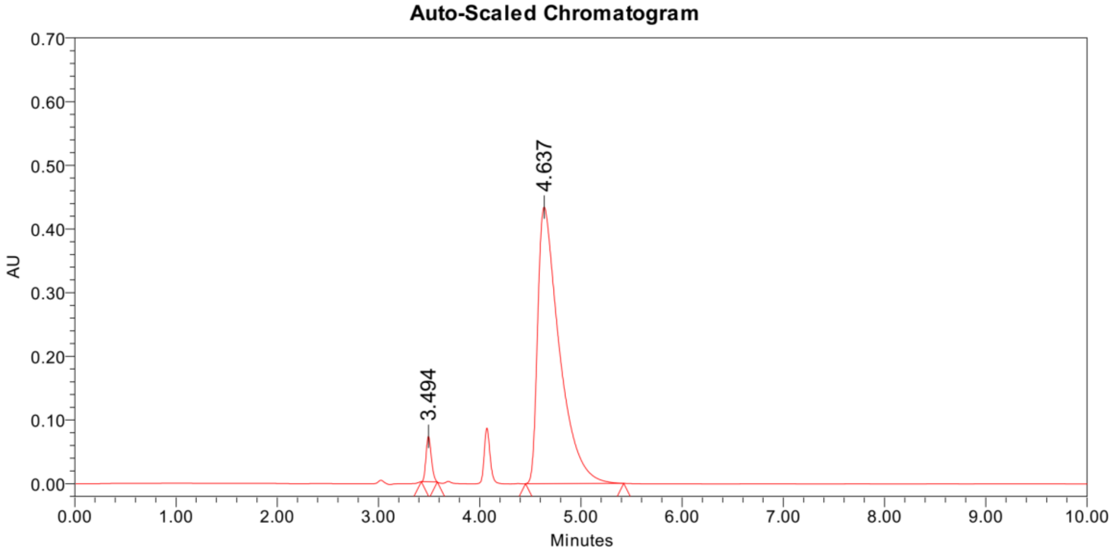

Peak Results

	Name	RT	Area	Height	% Area
1		3.494	261997	71488	3.91
2		4.637	6440489	434397	96.09

Supplementary Figure 231. HPLC Spectra of **35** obtained from (*S*,*R*)-**L1**.

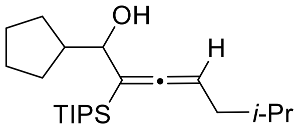

Figure 3, entry 36

(R,S)-**L1**: 92% ee; (S,R)-**L1**: 94% ee

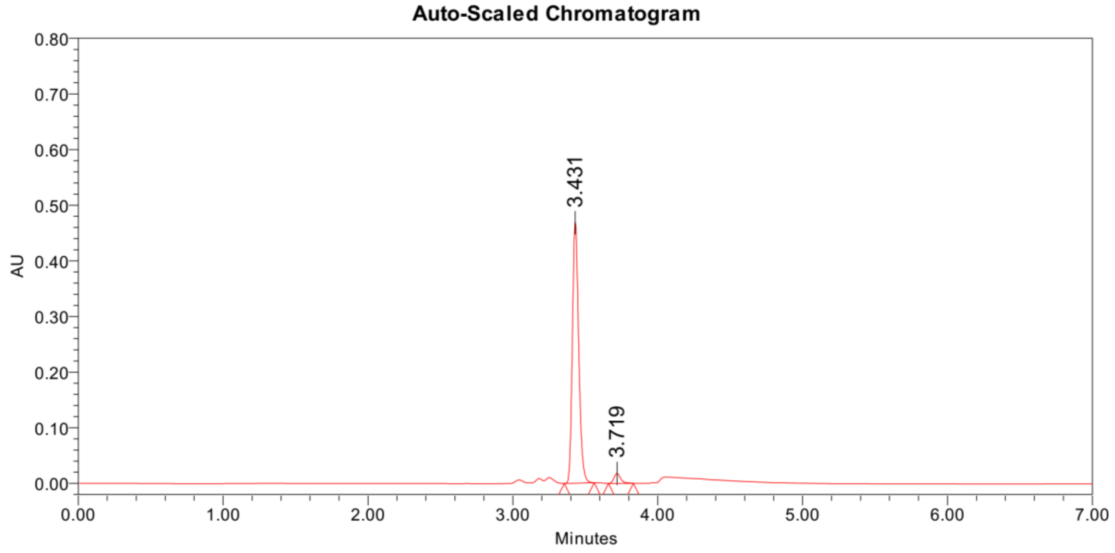

Peak Results

	Name	RT	Area	Height	% Area
1		3.431	1420912	469411	96.11
2		3.719	57522	17866	3.89

Supplementary Figure 232. HPLC Spectra of **36** obtained from (R,S)-**L1**.

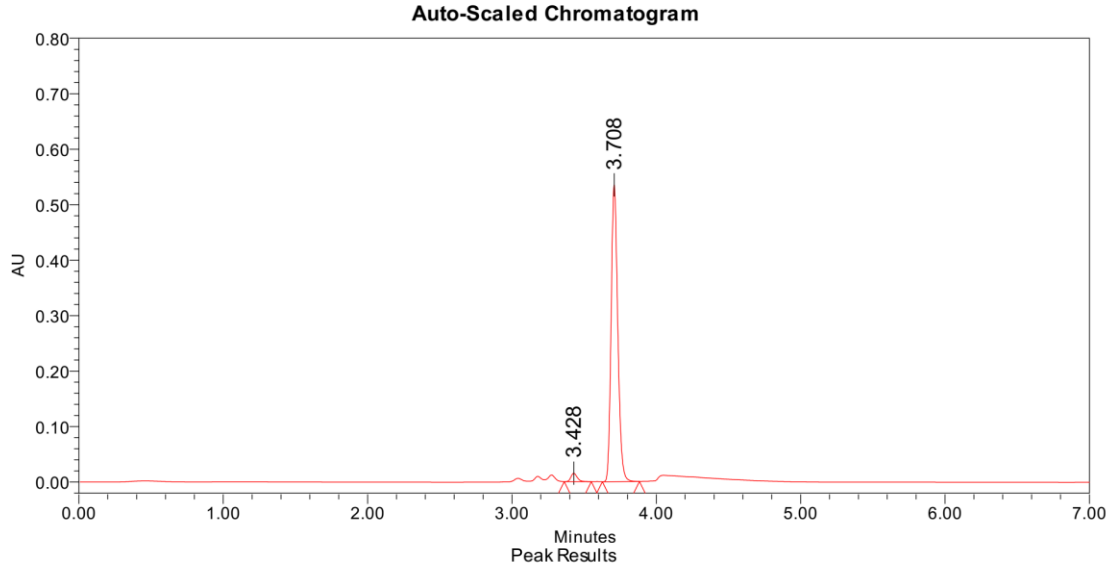

Peak Results

	Name	RT	Area	Height	% Area
1		3.428	46053	15191	2.61
2		3.708	1718248	535248	97.39

Supplementary Figure 233. HPLC Spectra of **36** obtained from (S,R)-**L1**.

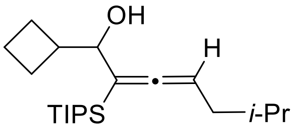

Figure 3, entry 37

(*R*,*S*)-**L1**: 92% ee; (*S*,*R*)-**L1**: 92% ee

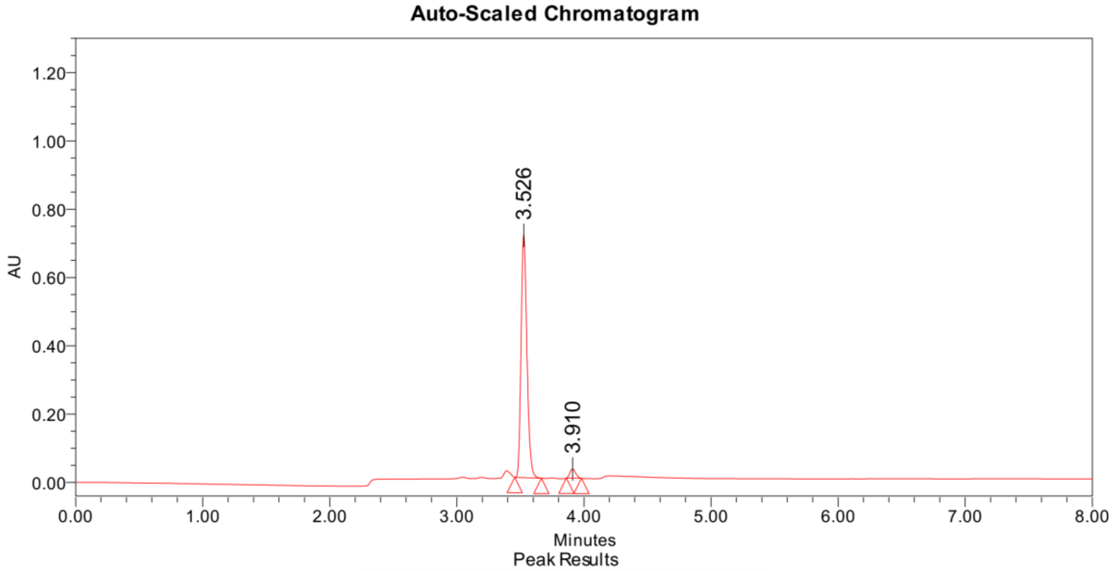

Peak Results

	Name	RT	Area	Height	% Area
1		3.526	2269157	710276	96.33
2		3.910	86429	27008	3.67

Supplementary Figure 234. HPLC Spectra of **37** obtained from (*R*,*S*)-**L1**.

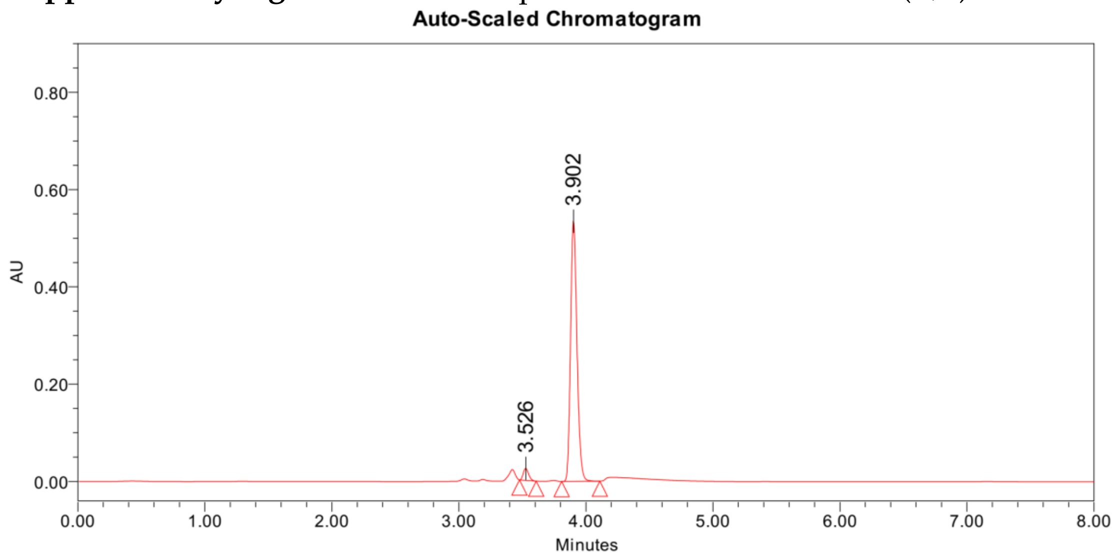

Peak Results

	Name	RT	Area	Height	% Area
1		3.526	72692	24751	3.73
2		3.902	1878239	535458	96.27

Supplementary Figure 235. HPLC Spectra of **37** obtained from (*S*,*R*)-**L1**.

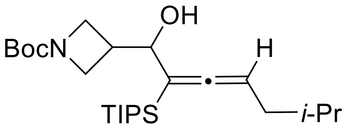

Figure 3, entry 38

(R,S)-**L1**: 91% ee; (S,R)-**L1**: 91% ee

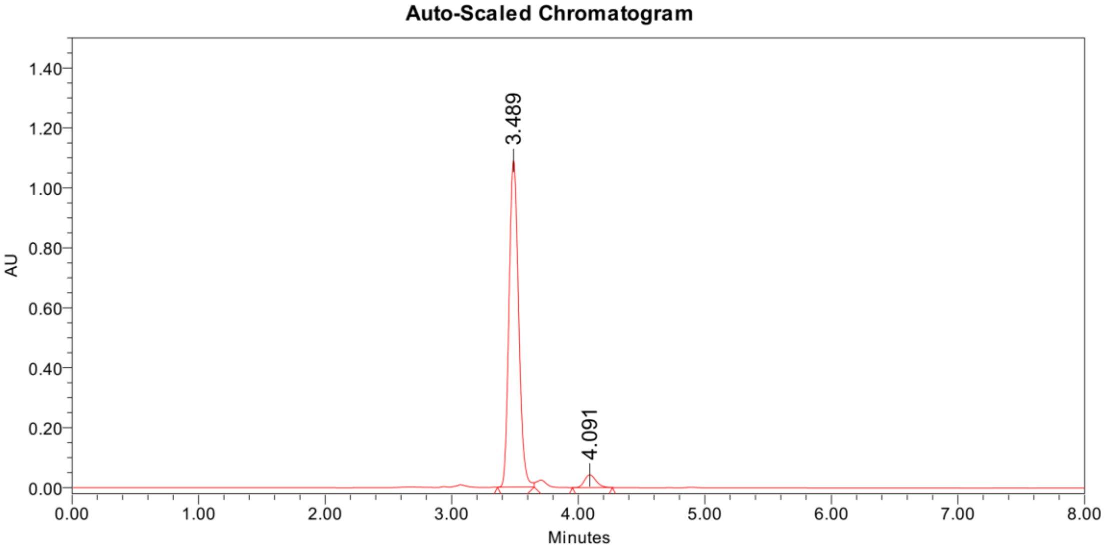

Peak Results

	Name	RT	Area	Height	% Area
1		3.489	5728005	1090390	95.53
2		4.091	268209	42422	4.47

Supplementary Figure 236. HPLC Spectra of **38** obtained from (R,S)-**L1**.

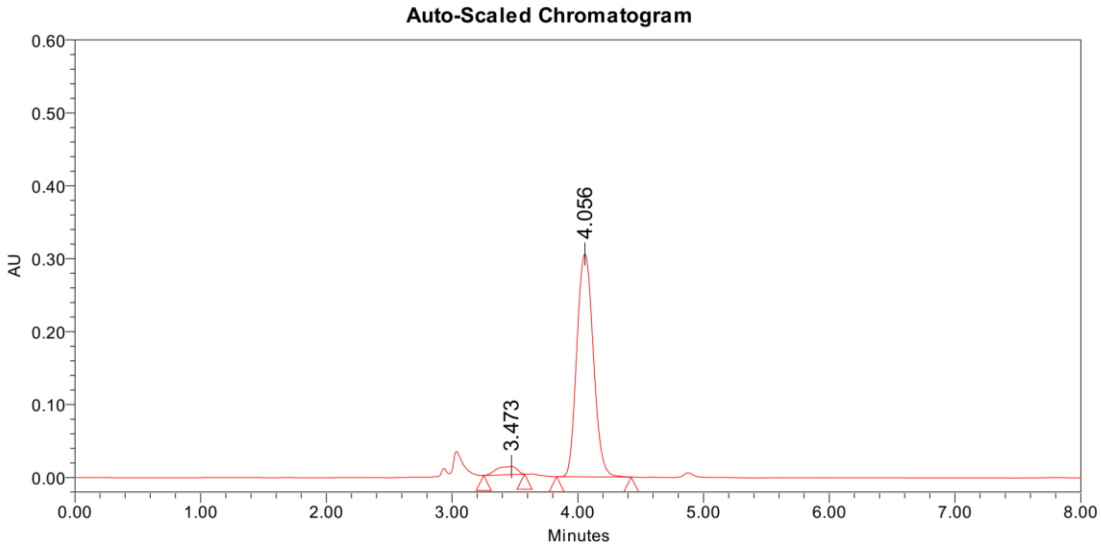

Peak Results

	Name	RT	Area	Height	% Area
1		3.473	126329	11081	4.33
2		4.056	2787946	305813	95.67

Supplementary Figure 237. HPLC Spectra of **38** obtained from (S,R)-**L1**.

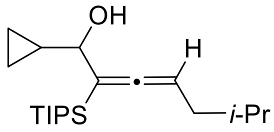

Figure 3, entry 39

(*R*,*S*)-**L1**: 90% ee; (*S*,*R*)-**L1**: 90% ee

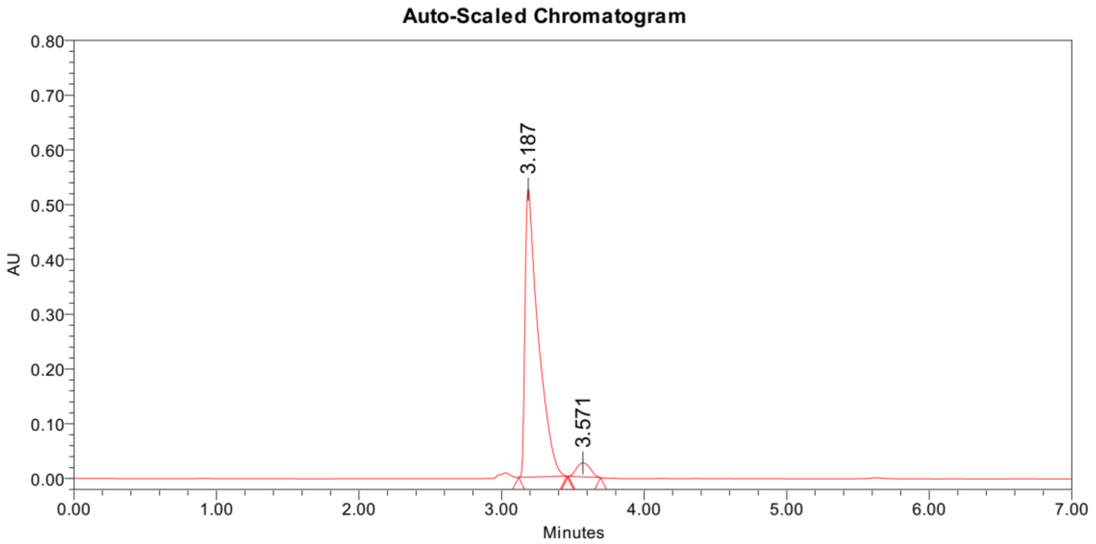

Peak Results

	Name	RT	Area	Height	% Area
1		3.187	3384664	526509	95.20
2		3.571	170490	25435	4.80

Supplementary Figure 238. HPLC Spectra of **39** obtained from (*R*,*S*)-**L1**.

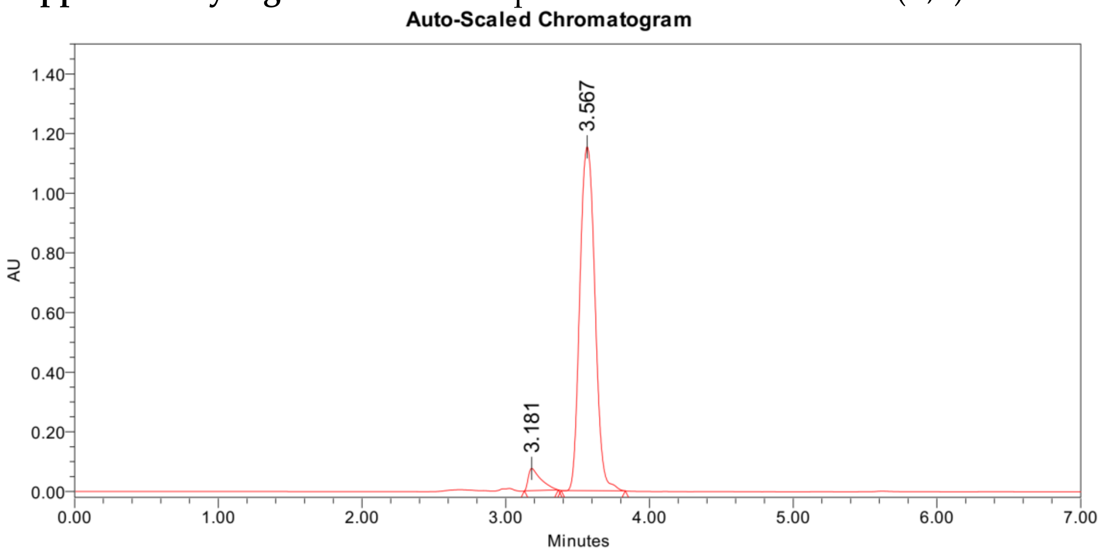

Peak Results

	Name	RT	Area	Height	% Area
1		3.181	451951	74741	5.08
2		3.567	8443701	1153028	94.92

Supplementary Figure 239. HPLC Spectra of **39** obtained from (*S*,*R*)-**L1**.

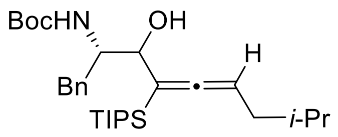

Figure 3, entry 40&41

(*R*,*S*)-**L1**: 1:99 dr; (*S*,*R*)-**L1**: 99:1 dr

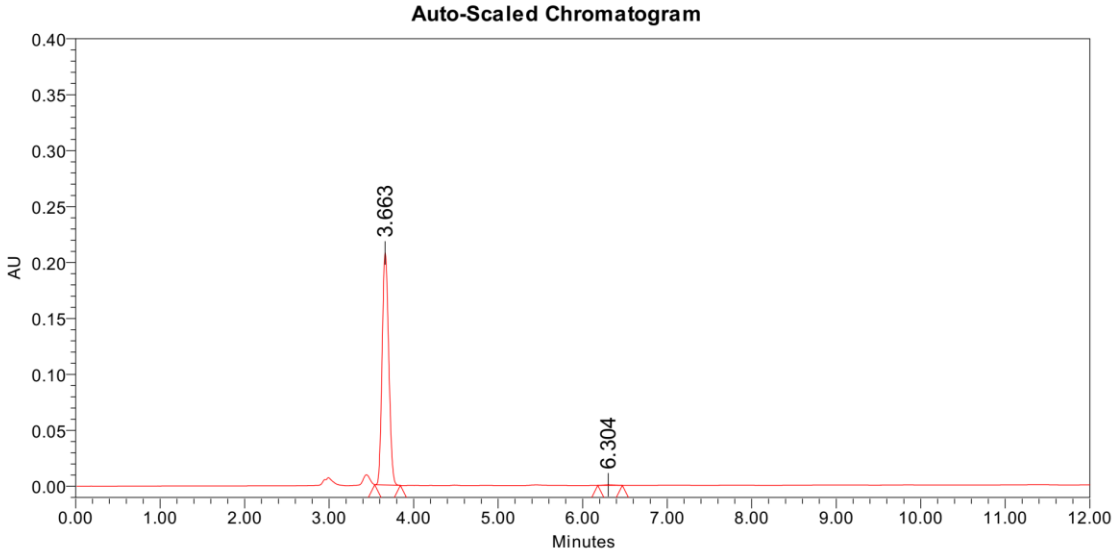

Peak Results

	Name	RT	Area	Height	% Area
1		3.663	1124300	207415	99.69
2		6.304	3550	497	0.31

Supplementary Figure 240. HPLC Spectra of **41** obtained from (*R*,*S*)-**L1**.

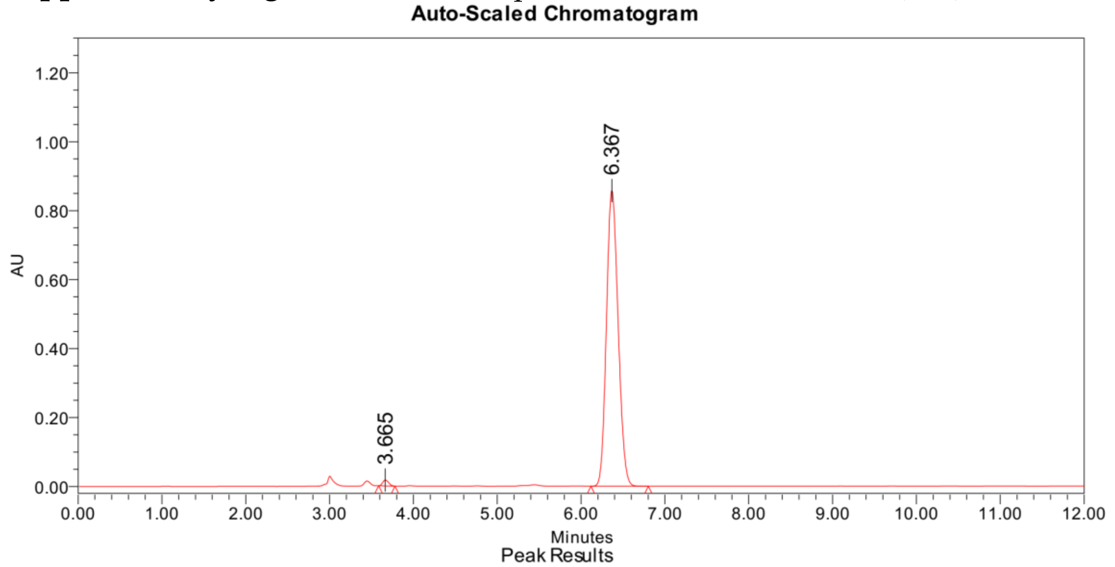

Peak Results

	Name	RT	Area	Height	% Area
1		3.665	84728	17224	1.01
2		6.367	8292133	857282	98.99

Supplementary Figure 241. HPLC Spectra of **40** obtained from (*S*,*R*)-**L1**.

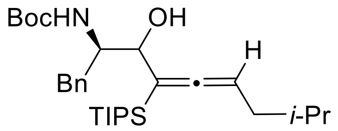

Figure 3, entry 42&43

(*R*,*S*)-**L1**: 1:99 dr; (*S*,*R*)-**L1**: 99:1 dr

Auto-Scaled Chromatogram

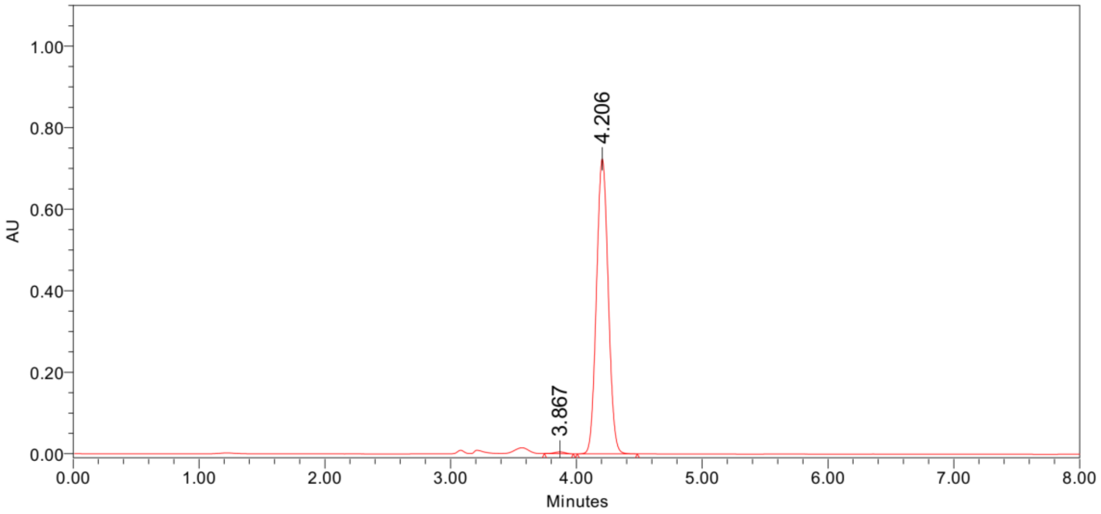

Peak Results

	Name	RT	Area	Height	% Area
1		3.867	25363	4335	0.54
2		4.206	4699942	724210	99.46

Supplementary Figure 242. HPLC Spectra of **43** obtained from (*R*,*S*)-**L1**.

Auto-Scaled Chromatogram

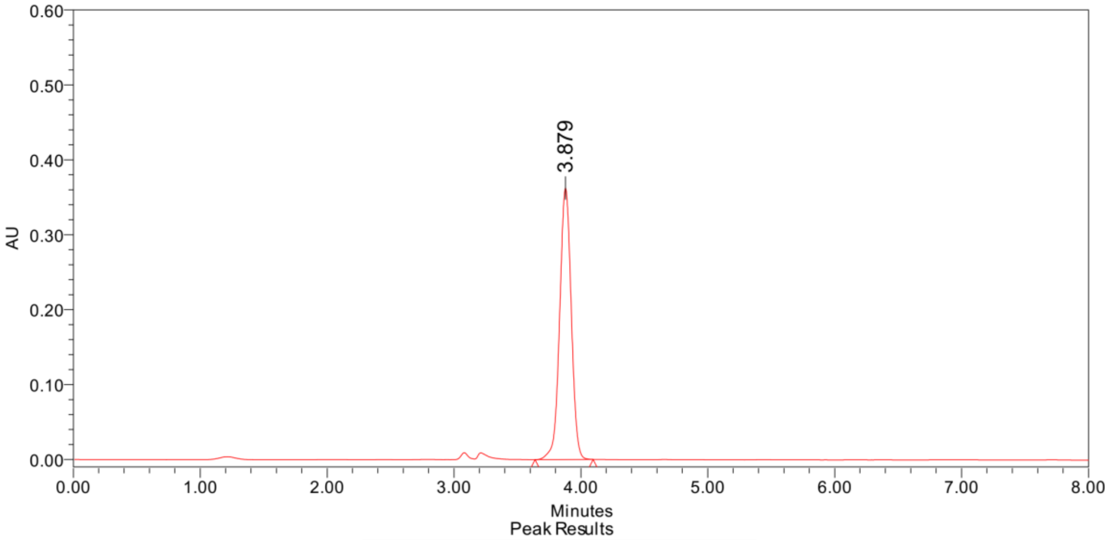

Peak Results

	Name	RT	Area	Height	% Area
1		3.879	2302424	362405	100.00

Supplementary Figure 243. HPLC Spectra of **42** obtained from (*S*,*R*)-**L1**.

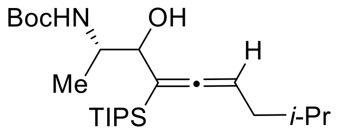

Figure 3, entry 44&45

(R,S)-**L1**: 1:99 dr; (S,R)-**L1**: 99:1 dr

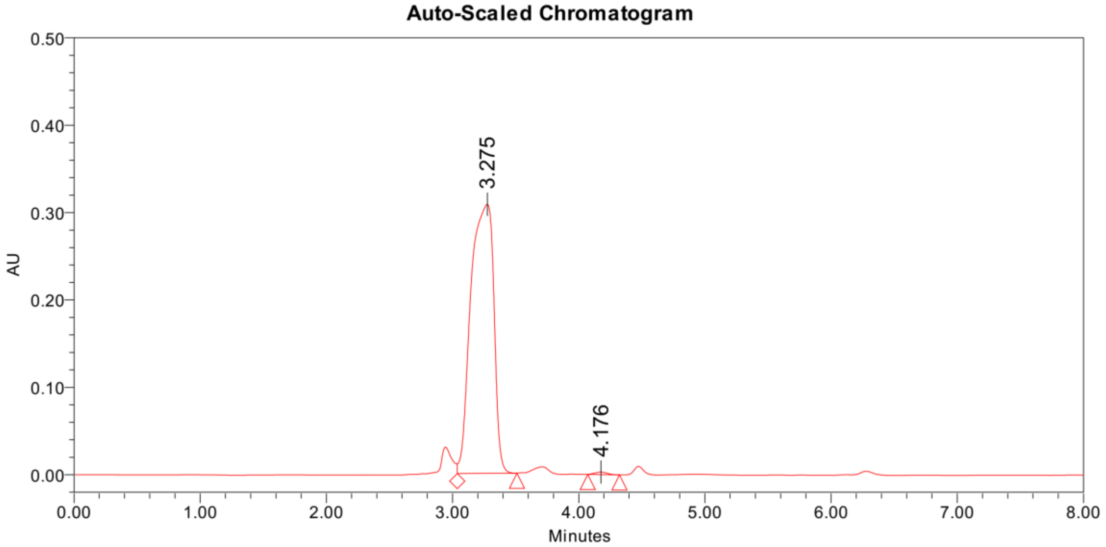

Peak Results

	Name	RT	Area	Height	% Area
1		3.275	3895746	307886	99.51
2		4.176	19097	2879	0.49

Supplementary Figure 244. HPLC Spectra of **45** obtained from (*R*,*S*)-**L1**.

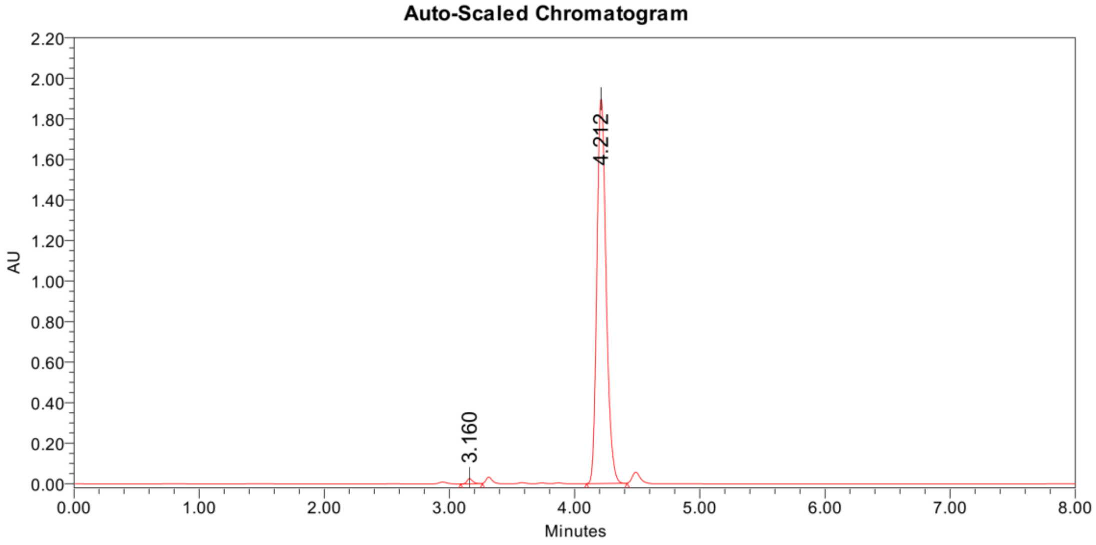

Peak Results

	Name	RT	Area	Height	% Area
1		3.160	79037	25676	0.80
2		4.212	9758960	1898353	99.20

Supplementary Figure 245. HPLC Spectra of **44** obtained from (*S*,*R*)-**L1**.

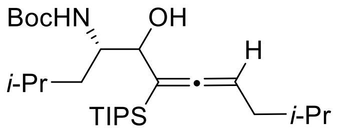

Figure 3, entry 46&47

(*R*,*S*)-**L1**: 1:99 dr; (*S*,*R*)-**L1**: 99:1 dr

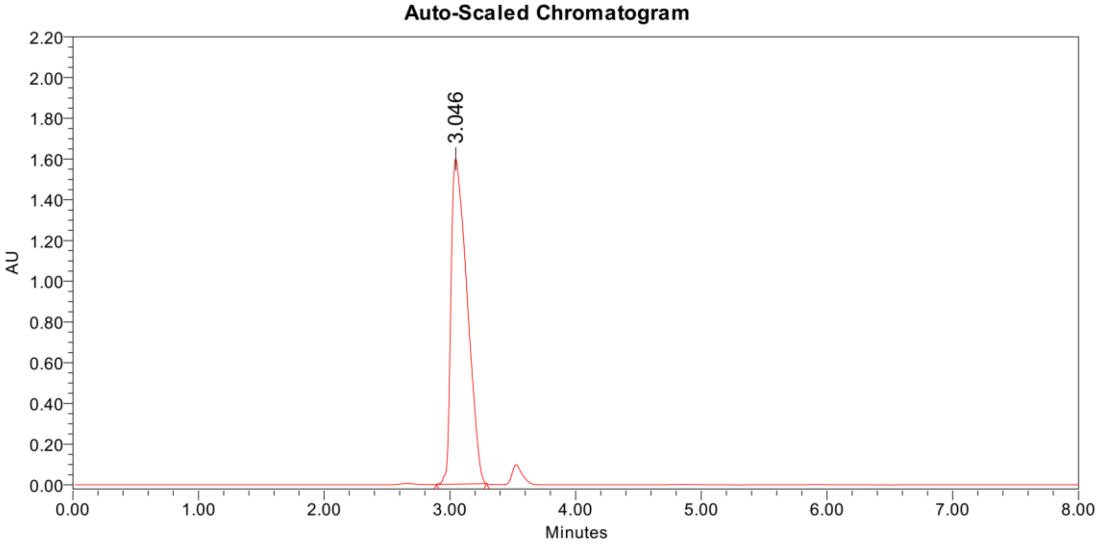

Peak Results

	Name	RT	Area	Height	% Area
1		3.046	14272518	1596958	100.00

Supplementary Figure 246. HPLC Spectra of **47** obtained from (*R*,*S*)-**L1**.

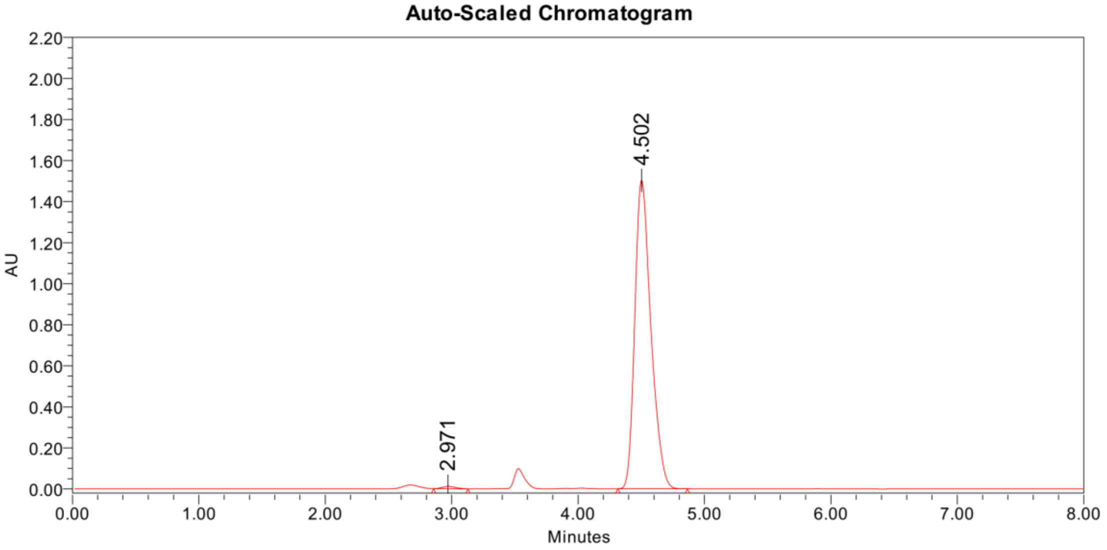

Peak Results

	Name	RT	Area	Height	% Area
1		2.971	84266	12516	0.63
2		4.502	13296432	1503037	99.37

Supplementary Figure 247. HPLC Spectra of **46** obtained from (*S*,*R*)-**L1**.

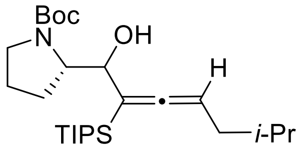

Figure 3, entry 48&49
(*R*,*S*)-**L1**: 1:99 dr; (*S*,*R*)-**L1**: 99:1 dr

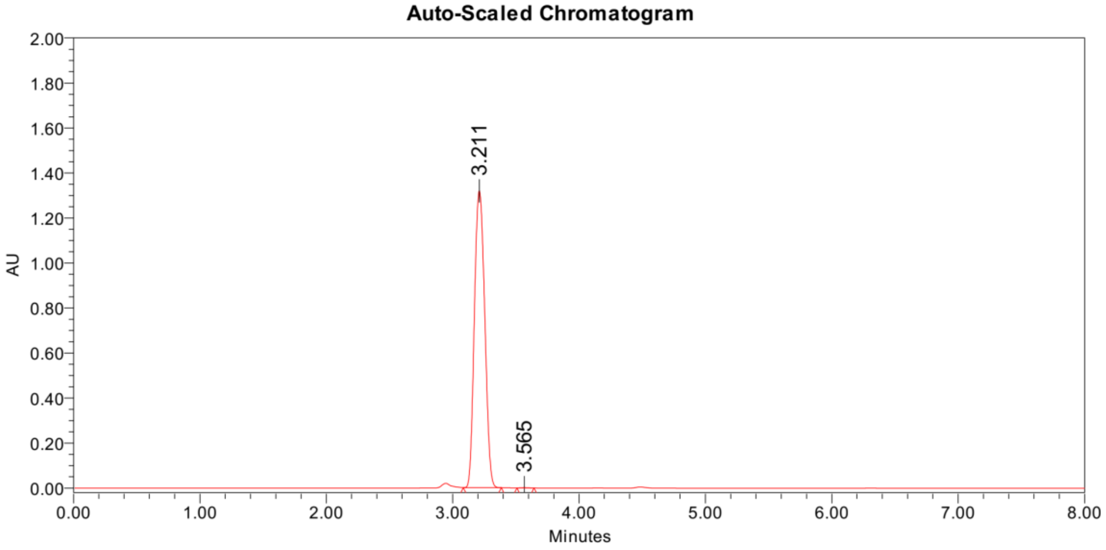

Peak Results

	Name	RT	Area	Height	% Area
1		3.211	7508554	1319417	99.94
2		3.565	4745	1117	0.06

Supplementary Figure 248. HPLC Spectra of **49** obtained from (*R*,*S*)-**L1**.

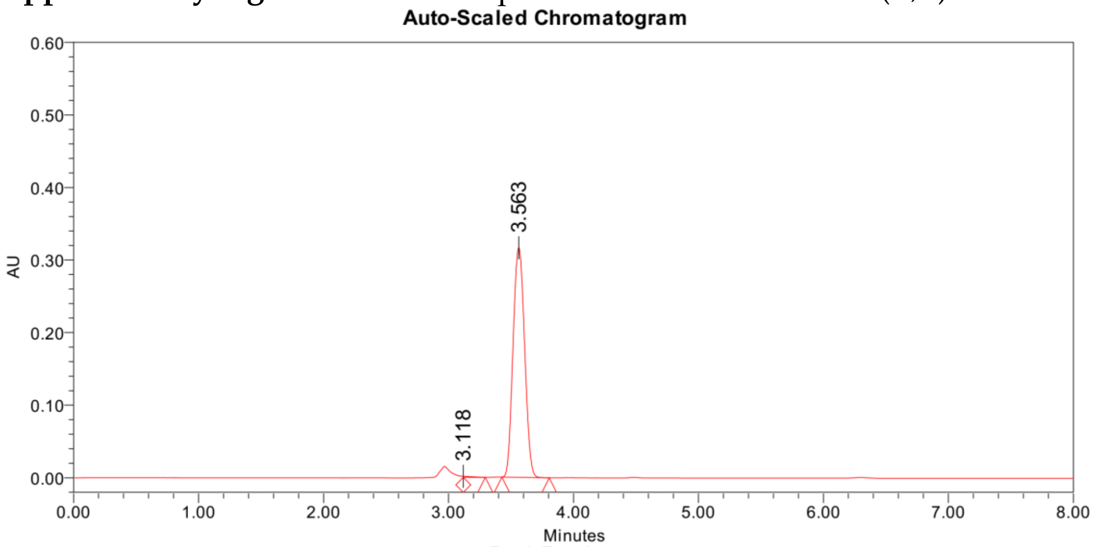

Peak Results

	Name	RT	Area	Height	% Area
1		3.118	8056	1502	0.40
2		3.563	2027654	316364	99.60

Supplementary Figure 249. HPLC Spectra of **48** obtained from (*S*,*R*)-**L1**.

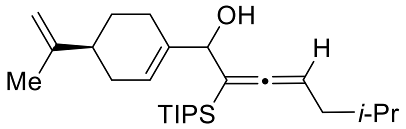

Figure 3, entry 50&51
(*R*,*S*)-**L1**: 1:99 dr; (*S*,*R*)-**L1**: 99:1 dr

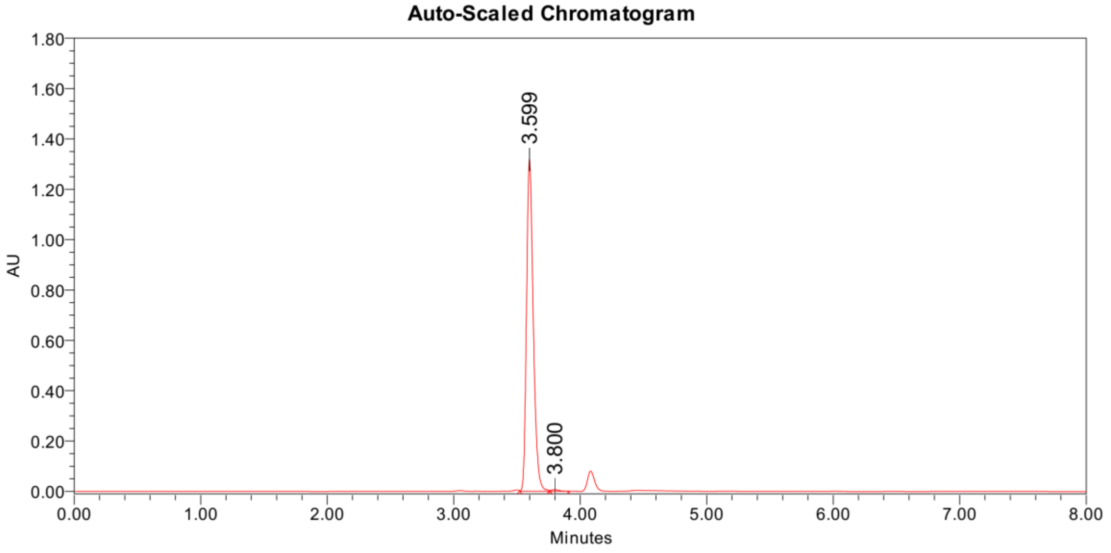

Peak Results

	Name	RT	Area	Height	% Area
1		3.599	4879196	1318740	99.45
2		3.800	27009	6288	0.55

Supplementary Figure 250. HPLC Spectra of **51** obtained from (*R*,*S*)-**L1**.

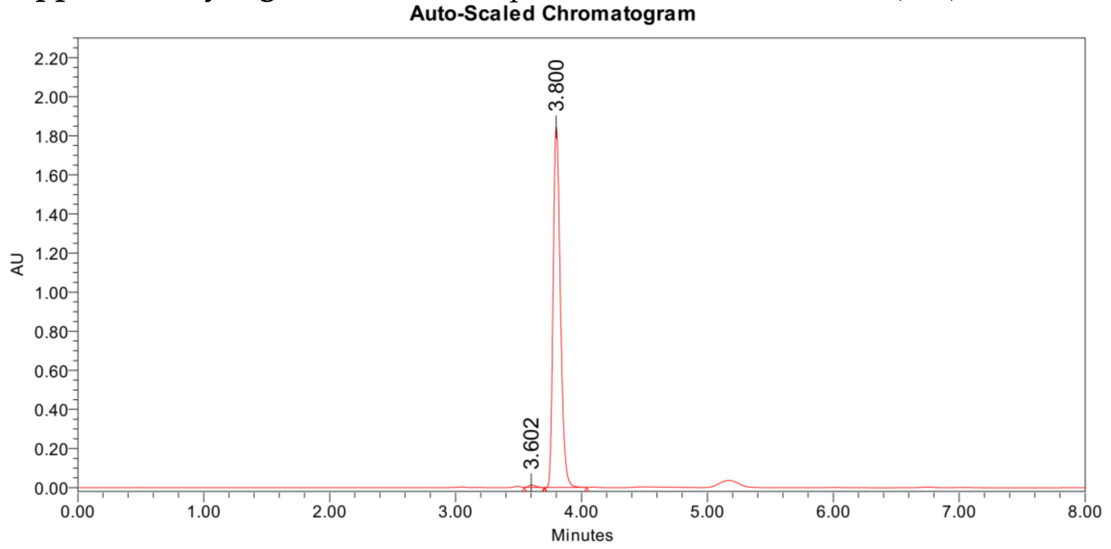

Peak Results

	Name	RT	Area	Height	% Area
1		3.602	47956	12759	0.65
2		3.800	7383653	1845111	99.35

Supplementary Figure 251. HPLC Spectra of **50** obtained from (*S*,*R*)-**L1**.

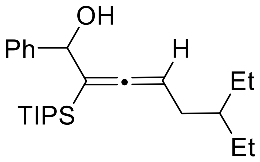

Figure 4, entry 52

(*R*,*S*)-**L1**: 95% ee; (*S*,*R*)-**L1**: 94% ee

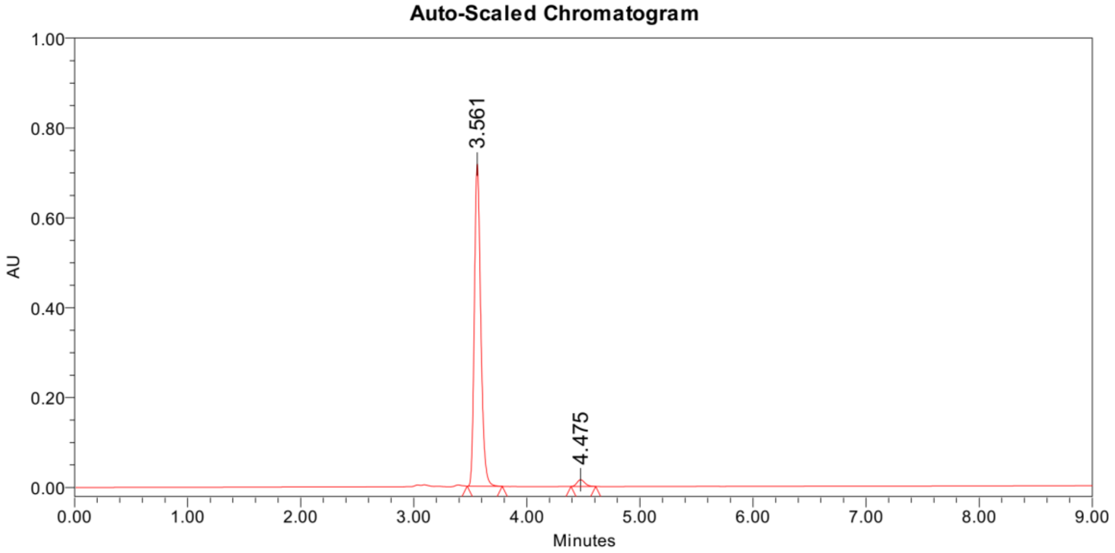

Peak Results

	Name	RT	Area	Height	% Area
1		3.561	2830099	718317	97.50
2		4.475	72481	15128	2.50

Supplementary Figure 252. HPLC Spectra of **52** obtained from (*R*,*S*)-**L1**.

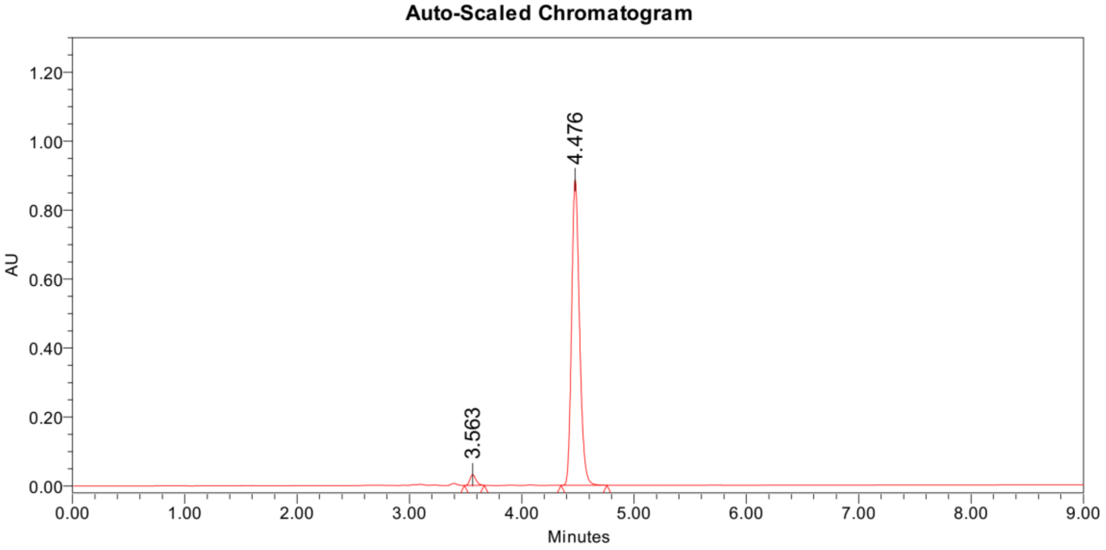

Peak Results

	Name	RT	Area	Height	% Area
1		3.563	117894	31162	2.64
2		4.476	4340225	887363	97.36

Supplementary Figure 253. HPLC Spectra of **52** obtained from (*S*,*R*)-**L1**.

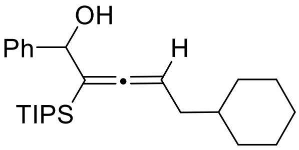

Figure 4, entry 53

(*R*,*S*)-**L1**: 95% ee; (*S*,*R*)-**L1**: 94% ee

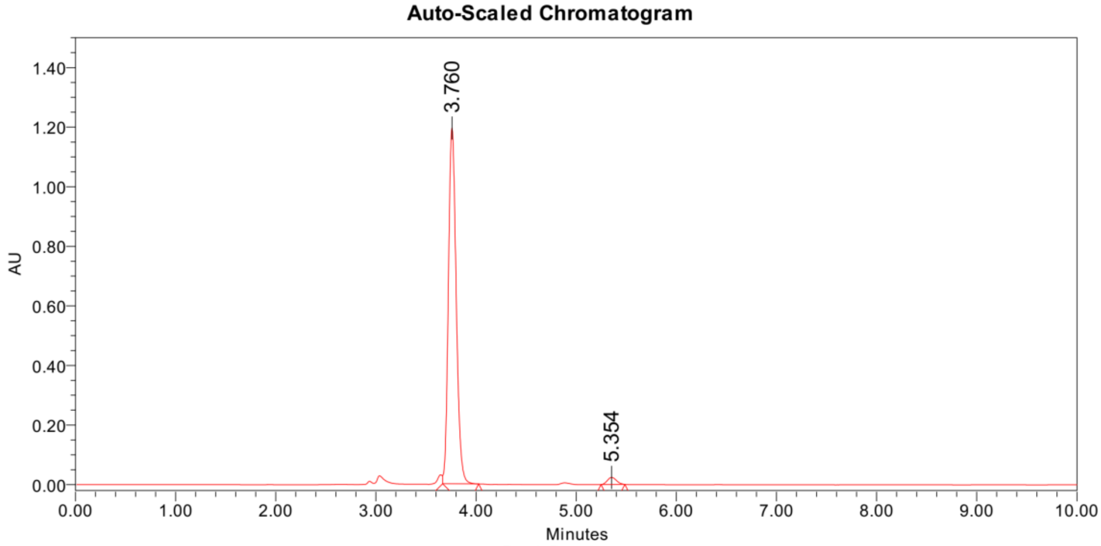

Peak Results

	Name	RT	Area	Height	% Area
1		3.760	6468501	1194509	97.75
2		5.354	149214	24007	2.25

Supplementary Figure 254. HPLC Spectra of **53** obtained from (*R*,*S*)-**L1**.

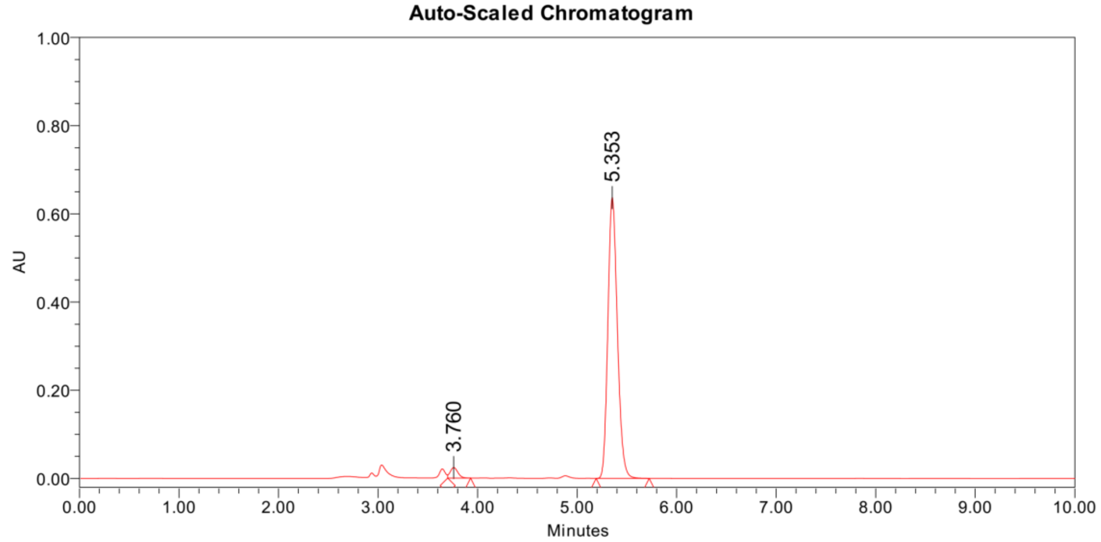

Peak Results

	Name	RT	Area	Height	% Area
1		3.760	127102	23783	2.92
2		5.353	4231705	636971	97.08

Supplementary Figure 255. HPLC Spectra of **53** obtained from (*S*,*R*)-**L1**.

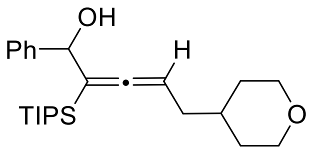

Figure 4, entry 54

(*R*,*S*)-**L1**: 93% ee; (*S*,*R*)-**L1**: 92% ee

Auto-Scaled Chromatogram

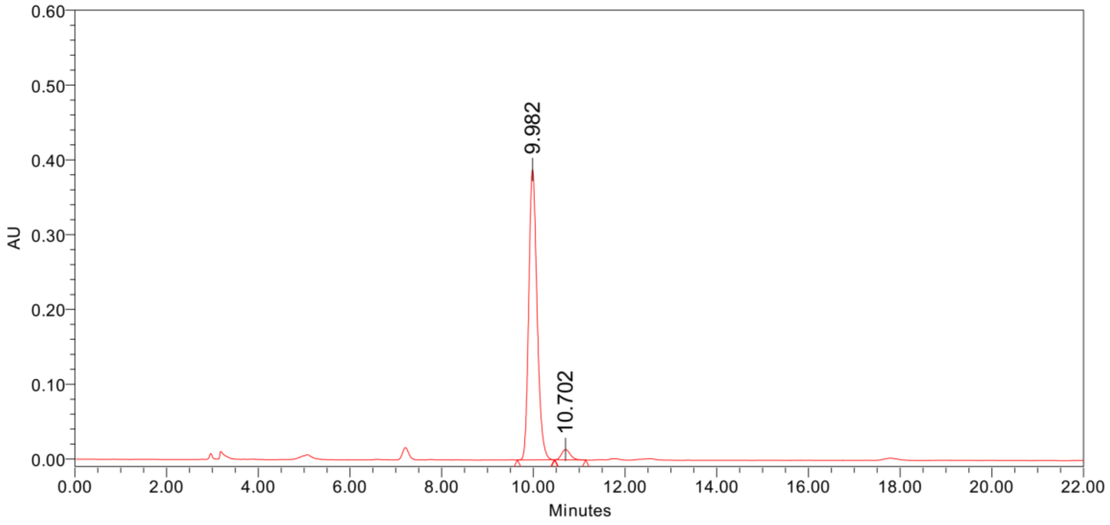

Peak Results

	Name	RT	Area	Height	% Area
1		9.982	4873025	388276	96.42
2		10.702	181114	13755	3.58

Supplementary Figure 256. HPLC Spectra of **54** obtained from (*R*,*S*)-**L1**.

Auto-Scaled Chromatogram

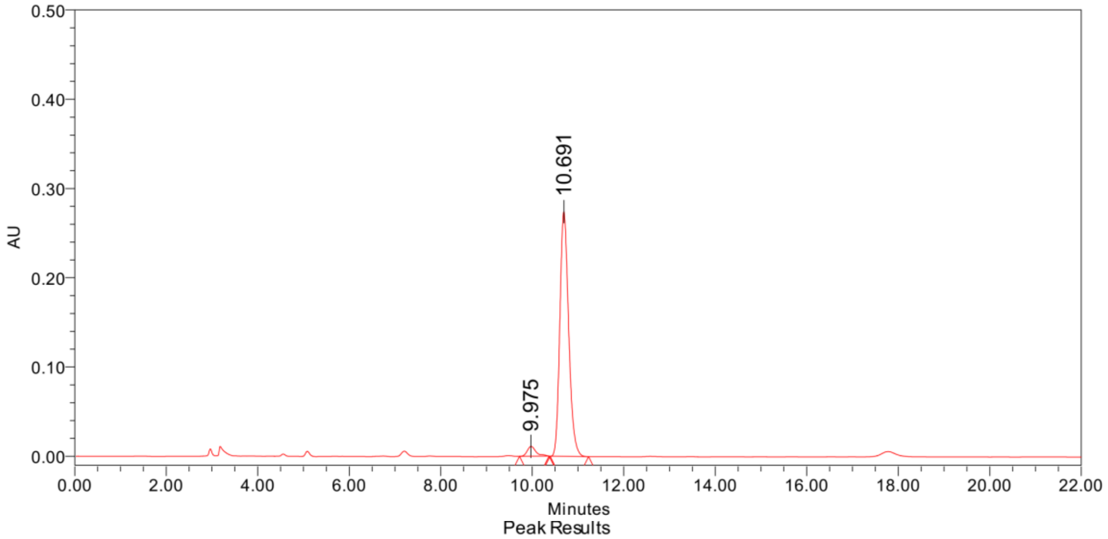

Peak Results

	Name	RT	Area	Height	% Area
1		9.975	147553	11060	3.87
2		10.691	3669125	274420	96.13

Supplementary Figure 257. HPLC Spectra of **54** obtained from (*S*,*R*)-**L1**.

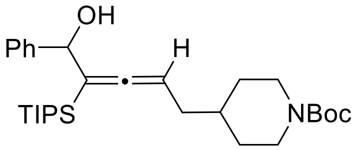

Figure 4, entry 55

(*R*,*S*)-**L1**: 92% ee; (*S*,*R*)-**L1**: 92% ee

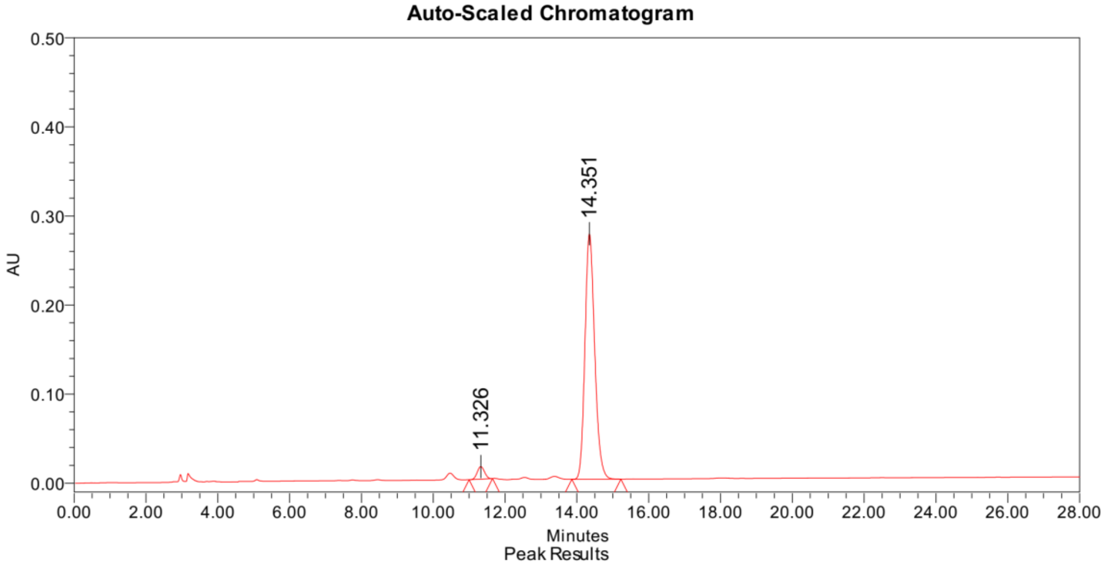

Peak Results

	Name	RT	Area	Height	% Area
1		11.326	196998	14080	3.65
2		14.351	5200502	275746	96.35

Supplementary Figure 258. HPLC Spectra of **55** obtained from (*R*,*S*)-**L1**.

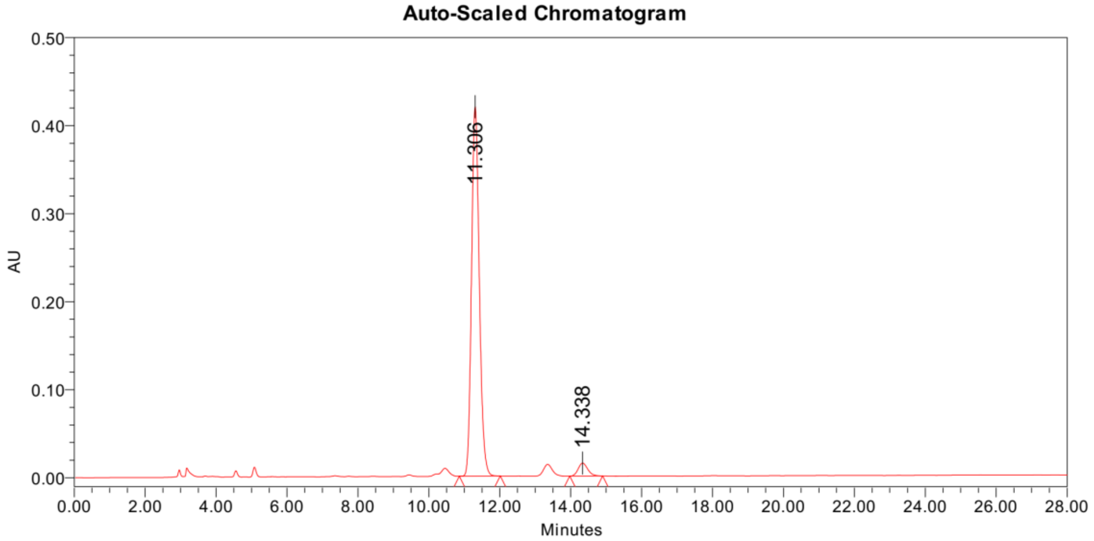

Peak Results

	Name	RT	Area	Height	% Area
1		11.306	6357551	419787	95.87
2		14.338	273631	14697	4.13

Supplementary Figure 259. HPLC Spectra of **55** obtained from (*S*,*R*)-**L1**.

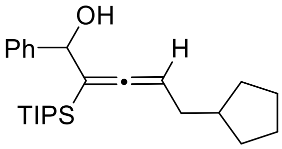

Figure 4, entry 56

(*R*,*S*)-**L1**: 95% ee; (*S*,*R*)-**L1**: 95% ee

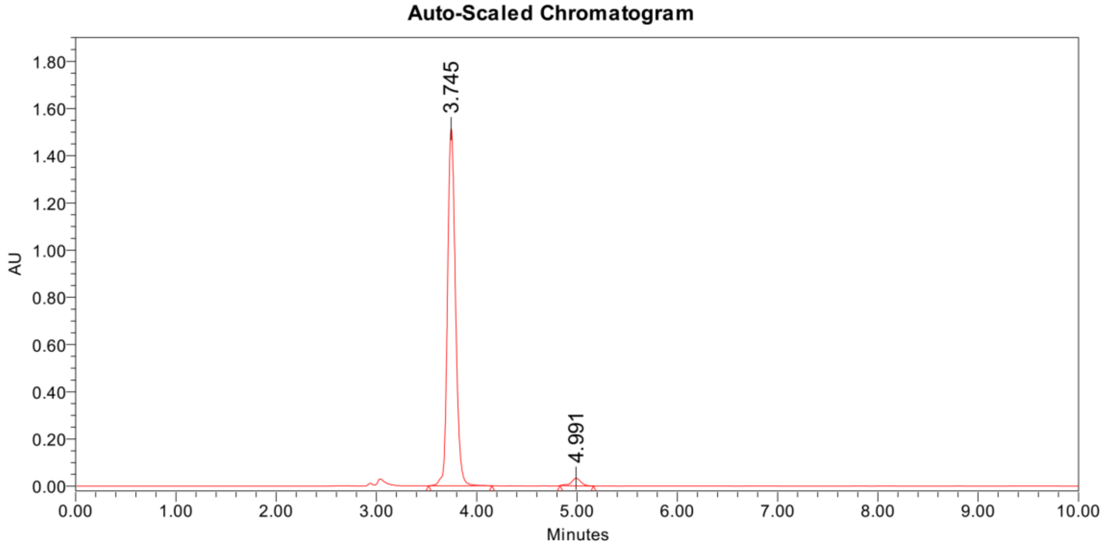

Peak Results

	Name	RT	Area	Height	% Area
1		3.745	8340223	1512821	97.56
2		4.991	209005	31804	2.44

Supplementary Figure 260. HPLC Spectra of **56** obtained from (*R*,*S*)-**L1**.

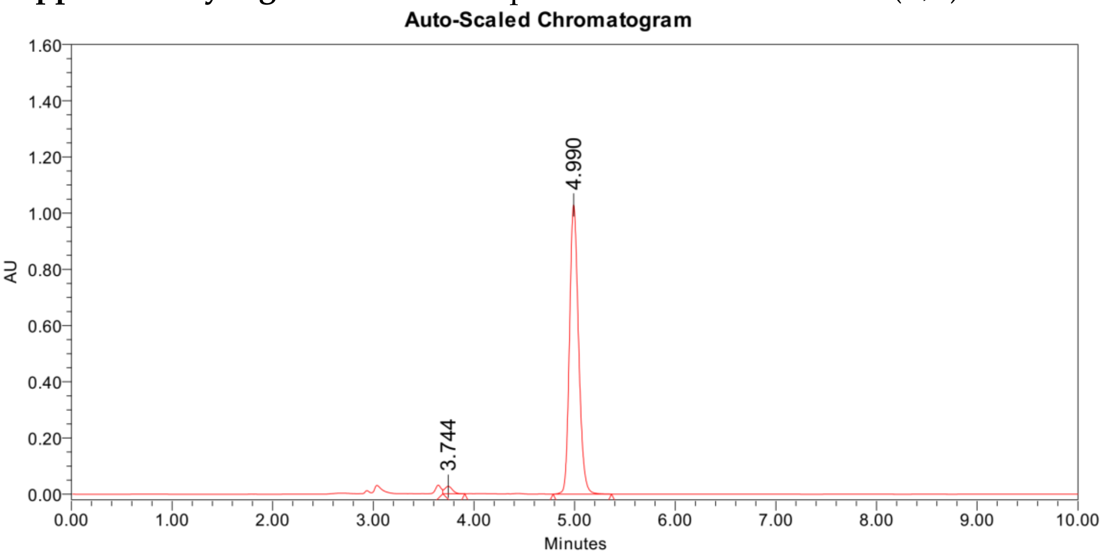

Peak Results

	Name	RT	Area	Height	% Area
1		3.744	144966	26732	2.21
2		4.990	6428520	1029375	97.79

Supplementary Figure 261. HPLC Spectra of **56** obtained from (*S*,*R*)-**L1**.

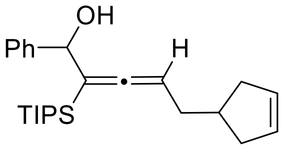

Figure 4, entry 57

(*R*,*S*)-**L1**: 92% ee; (*S*,*R*)-**L1**: 94% ee

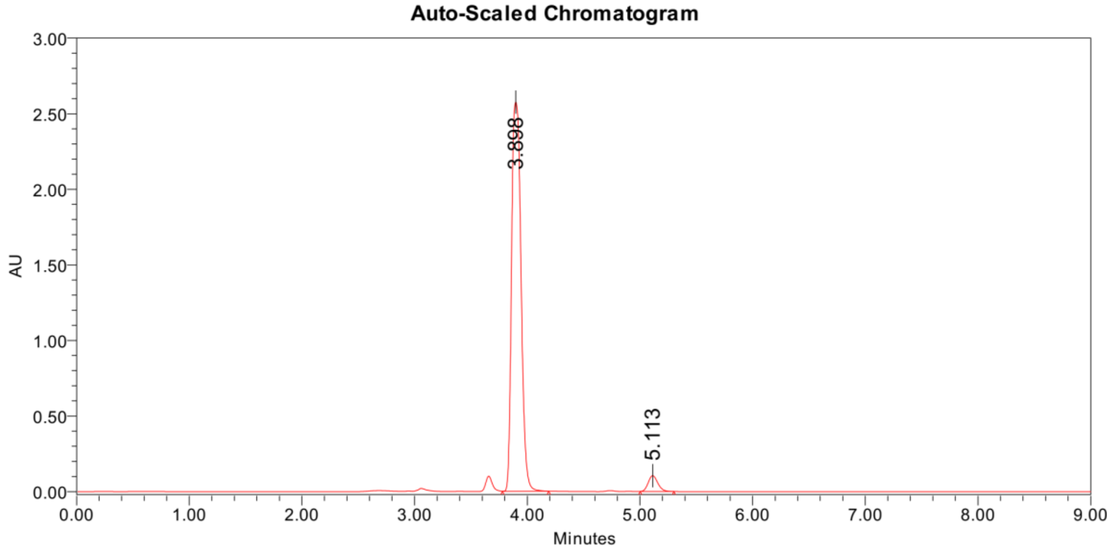

Peak Results

	Name	RT	Area	Height	% Area
1		3.898	14899476	2574716	96.00
2		5.113	620935	104949	4.00

Supplementary Figure 262. HPLC Spectra of **57** obtained from (*R*,*S*)-**L1**.

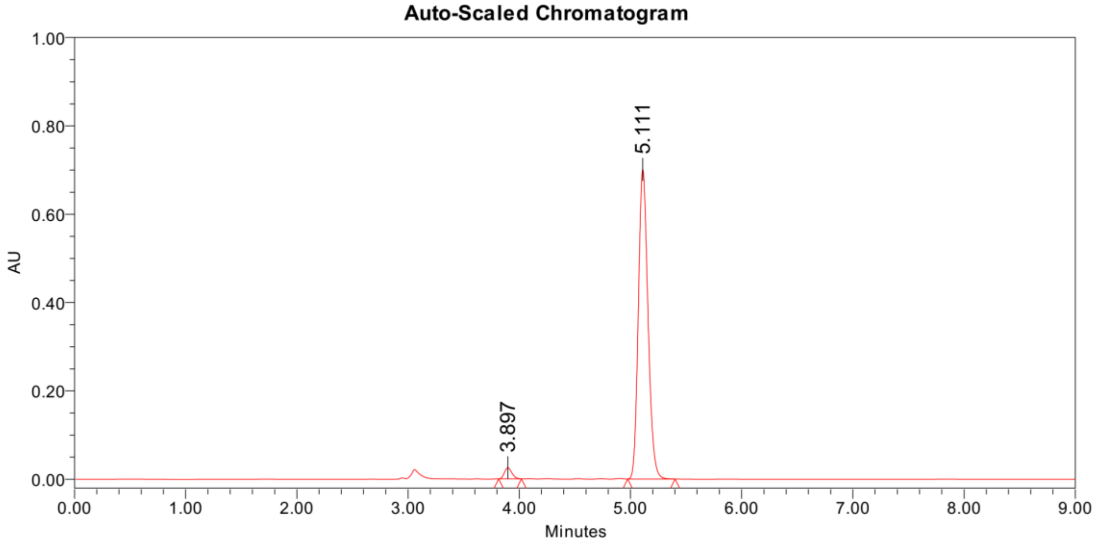

Peak Results

	Name	RT	Area	Height	% Area
1		3.897	117184	25126	2.70
2		5.111	4220003	701229	97.30

Supplementary Figure 263. HPLC Spectra of **57** obtained from (*S*,*R*)-**L1**.

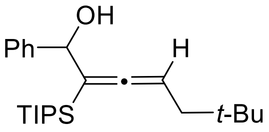

Figure 4, entry 58
(*R*,*S*)-**L1**: 96% ee; (*S*,*R*)-**L1**: 96% ee

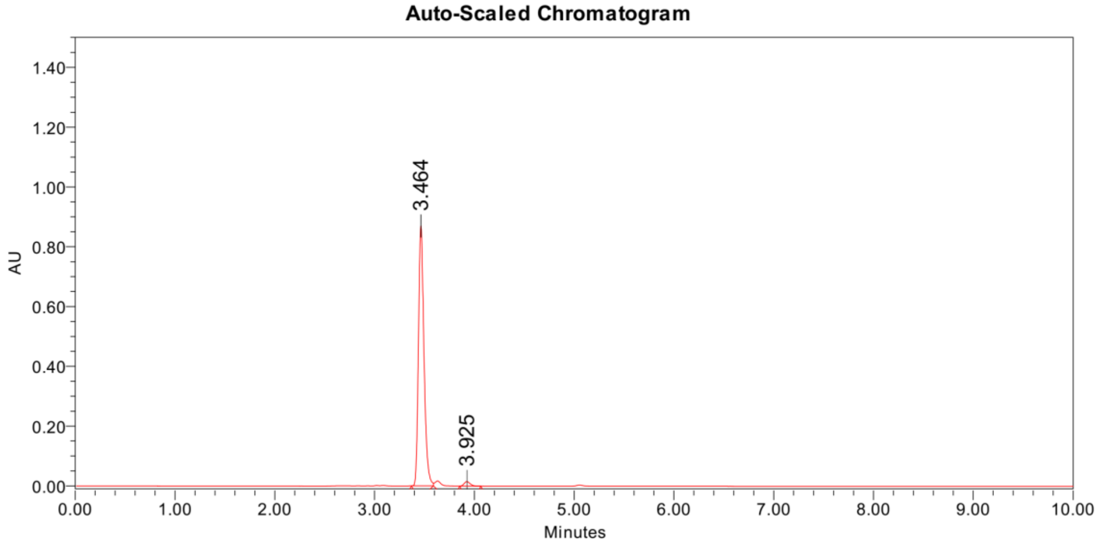

Peak Results

	Name	RT	Area	Height	% Area
1		3.464	3348832	869627	98.16
2		3.925	62743	15055	1.84

Supplementary Figure 264. HPLC Spectra of **58** obtained from (*R*,*S*)-**L1**.

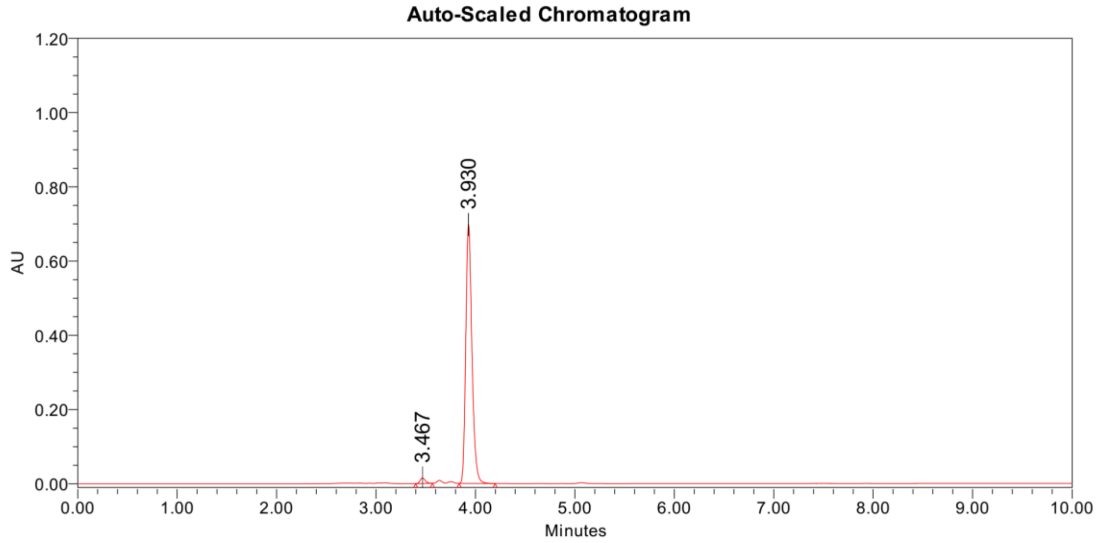

Peak Results

	Name	RT	Area	Height	% Area
1		3.467	53653	14870	1.75
2		3.930	3004184	697841	98.25

Supplementary Figure 265. HPLC Spectra of **58** obtained from (*S*,*R*)-**L1**.

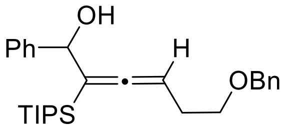

Figure 4, entry 59a
(*R*,*S*)-**L1**: 84% ee; (*S*,*R*)-**L1**: 86% ee

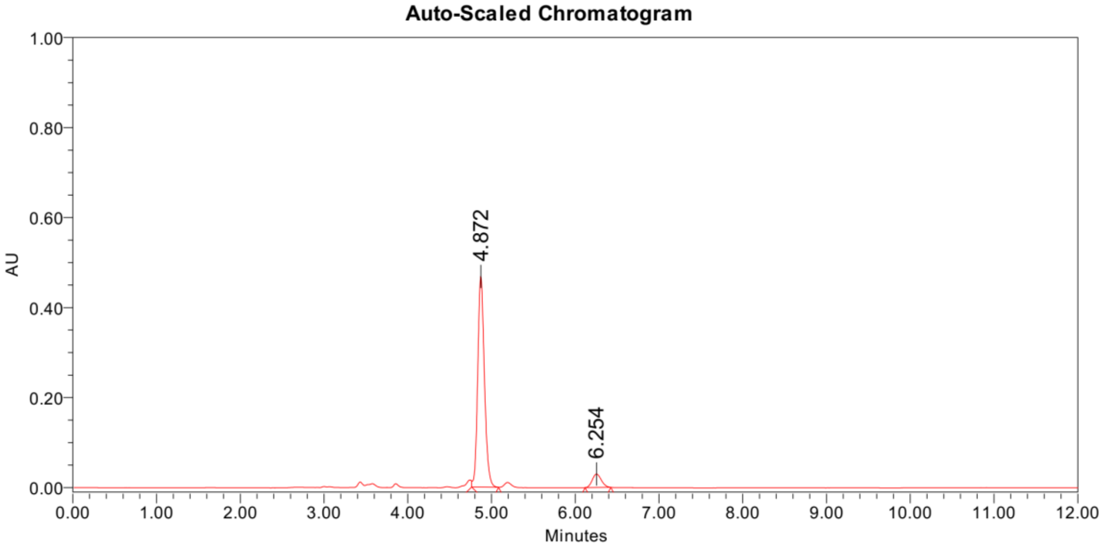

Peak Results

	Name	RT	Area	Height	% Area
1		4.872	2598801	468475	92.02
2		6.254	225324	30044	7.98

Supplementary Figure 266. HPLC Spectra of **59a** obtained from (*R*,*S*)-**L1**.

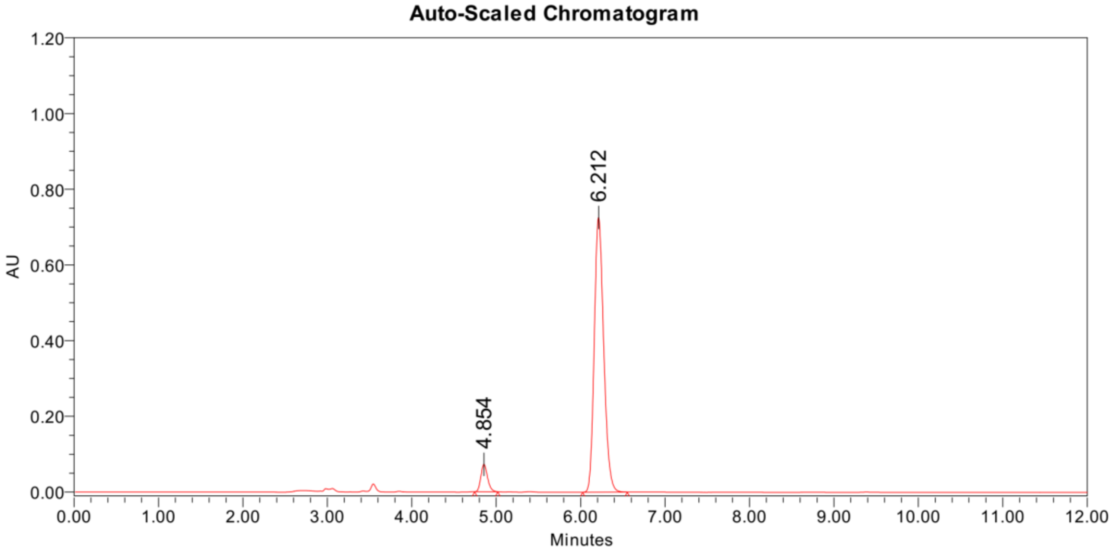

Peak Results

	Name	RT	Area	Height	% Area
1		4.854	396352	72446	6.56
2		6.212	5643888	725764	93.44

Supplementary Figure 267. HPLC Spectra of **59a** obtained from (*S*,*R*)-**L1**.

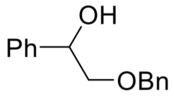

Figure 4, entry 59b

(*R*,*S*)-**L1**: 76% ee; (*S*,*R*)-**L1**: 76% ee

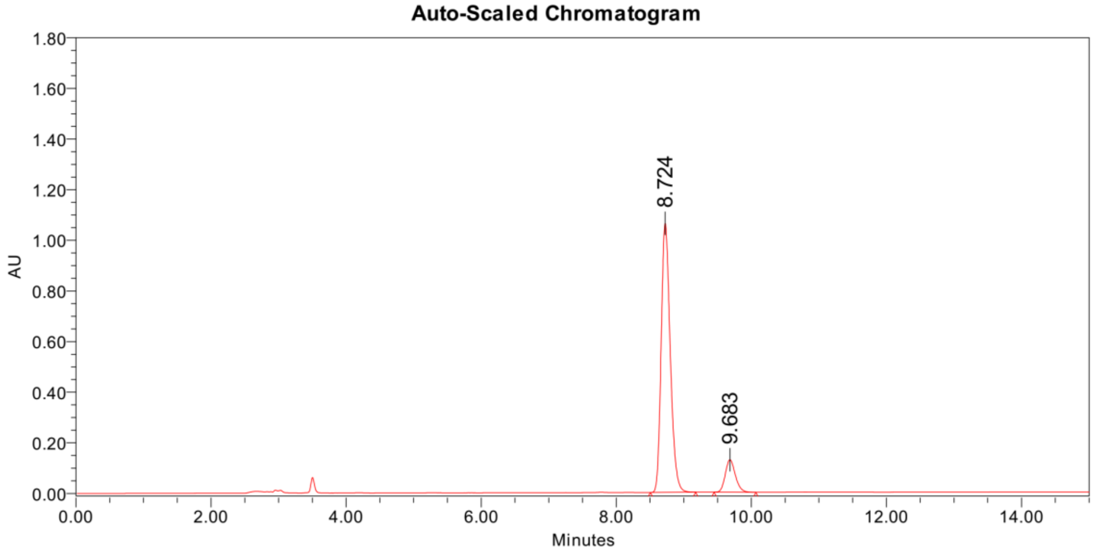

Peak Results

	Name	RT	Area	Height	% Area
1		8.724	9894257	1063151	88.20
2		9.683	1323733	128231	11.80

Supplementary Figure 268. HPLC Spectra of **59b** obtained from (*R*,*S*)-**L1**.

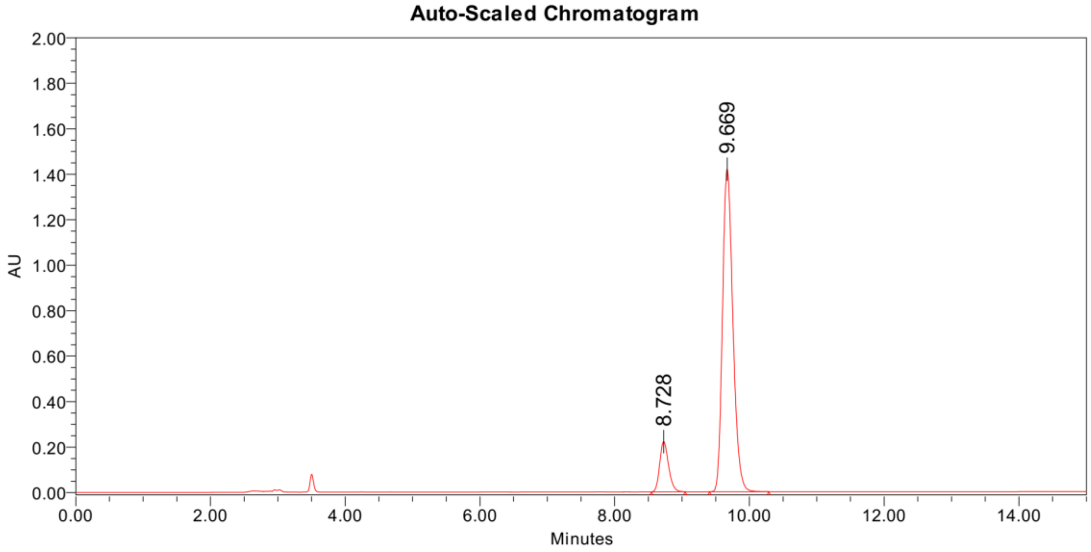

Peak Results

	Name	RT	Area	Height	% Area
1		8.728	2010941	220887	11.65
2		9.669	15250177	1418681	88.35

Supplementary Figure 269. HPLC Spectra of **59b** obtained from (*S*,*R*)-**L1**.

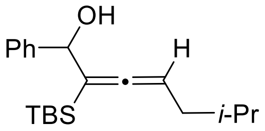

Figure 4, entry 60

(R,S)-**L1**: 92% ee; (S,R)-**L1**: 91% ee

Auto-Scaled Chromatogram

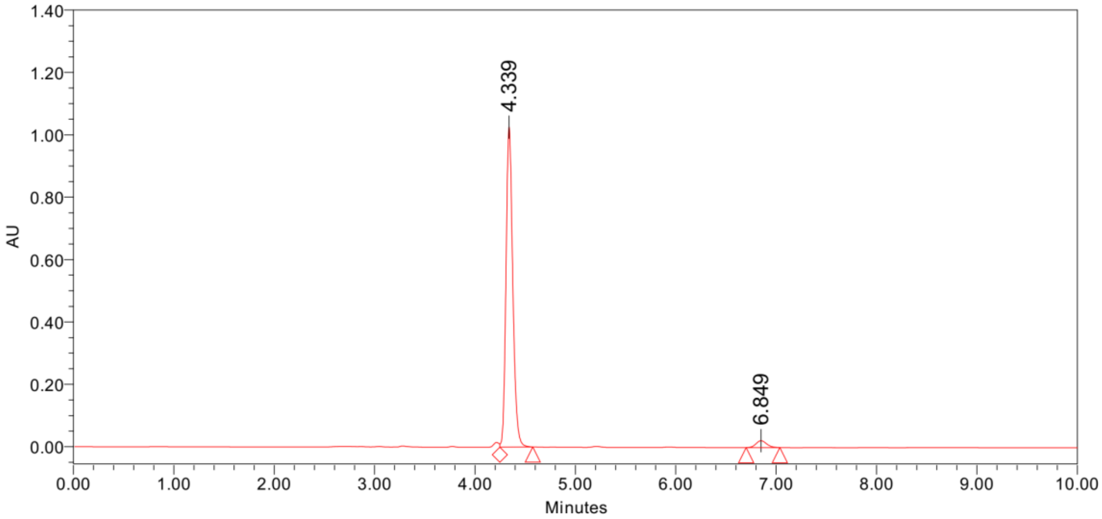

Peak Results

	Name	RT	Area	Height	% Area
1		4.339	4793370	1026111	96.72
2		6.849	162452	22095	3.28

Supplementary Figure 270. HPLC Spectra of **60** obtained from (R,S)-**L1**.

Auto-Scaled Chromatogram

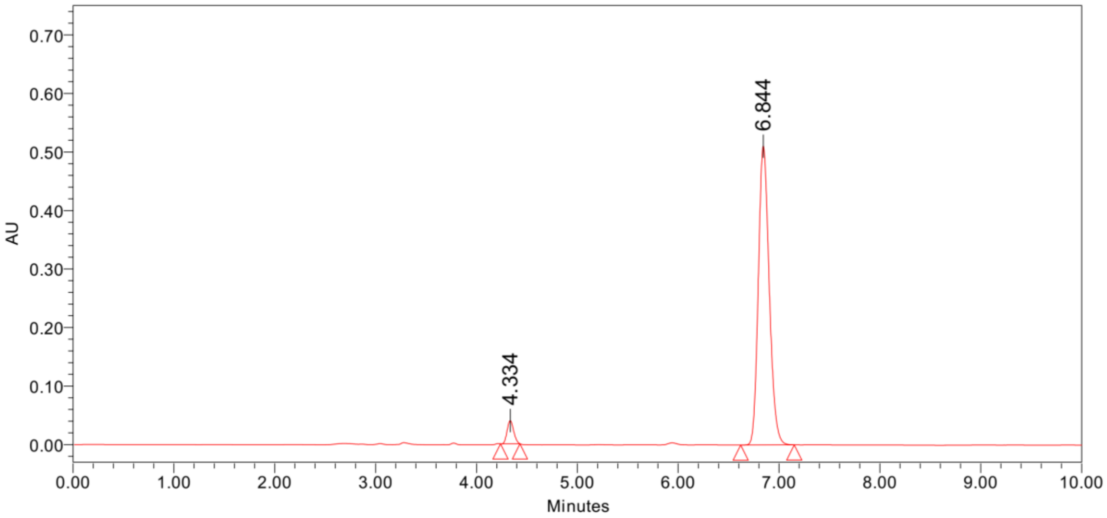

Peak Results

	Name	RT	Area	Height	% Area
1		4.334	179317	39660	4.48
2		6.844	3825389	510694	95.52

Supplementary Figure 271. HPLC Spectra of **60** obtained from (S,R)-**L1**.

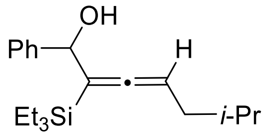

Figure 4, entry 61

(R,S)-**L1**: 93% ee; (S,R)-**L1**: 94% ee

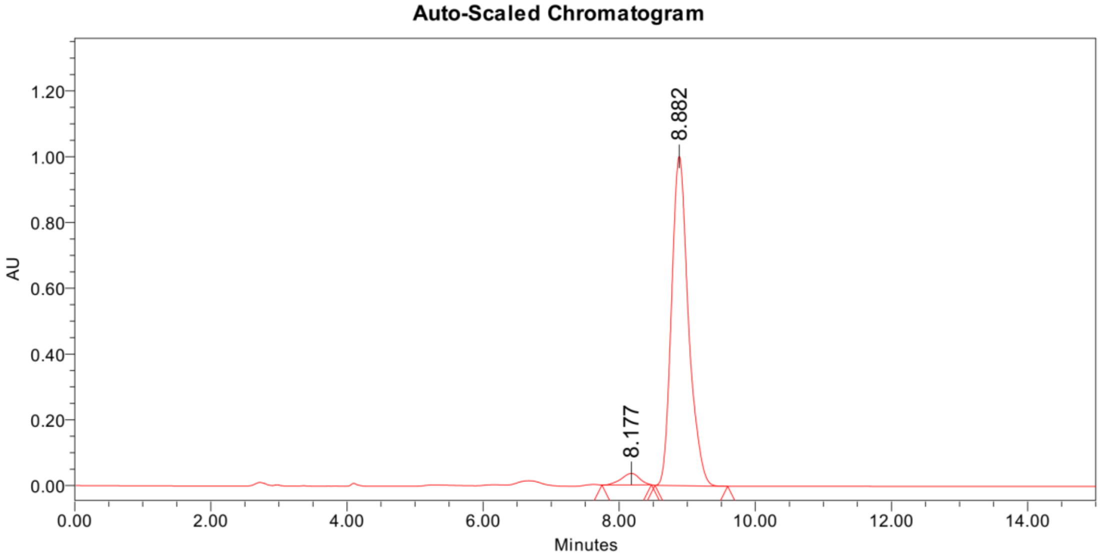

Peak Results

	Name	RT	Area	Height	% Area
1		8.177	644633	34920	3.59
2		8.882	17299569	1001574	96.41

Supplementary Figure 272. HPLC Spectra of **61** obtained from (R,S)-**L1**.

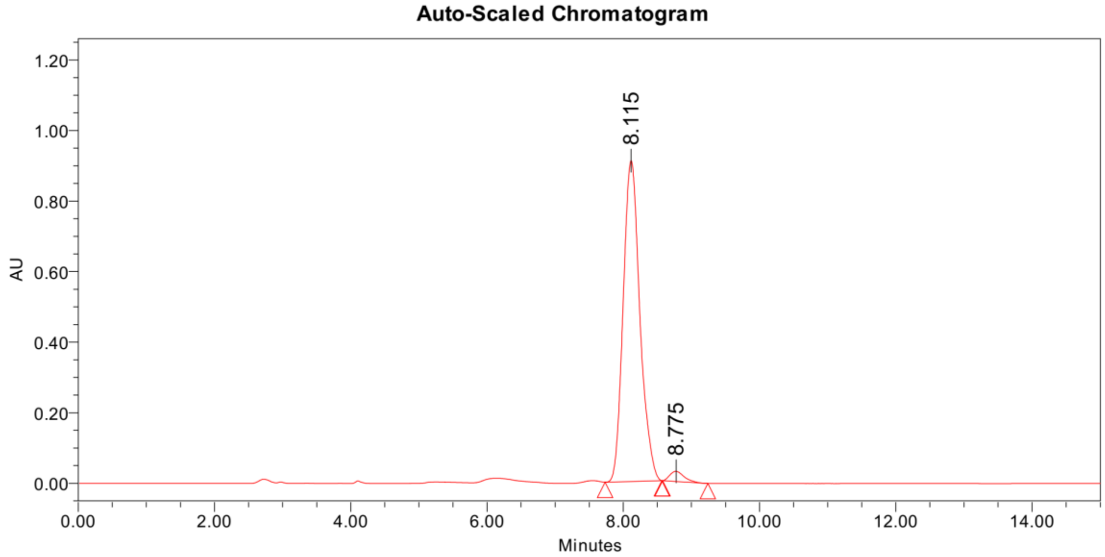

Peak Results

	Name	RT	Area	Height	% Area
1		8.115	15487292	909516	97.37
2		8.775	418478	28765	2.63

Supplementary Figure 273. HPLC Spectra of **61** obtained from (S,R)-**L1**.

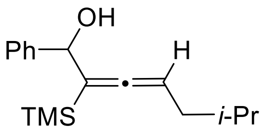

Figure 4, entry 62

(*R*,*S*)-**L1**: 95% ee; (*S*,*R*)-**L1**: 95% ee

Auto-Scaled Chromatogram

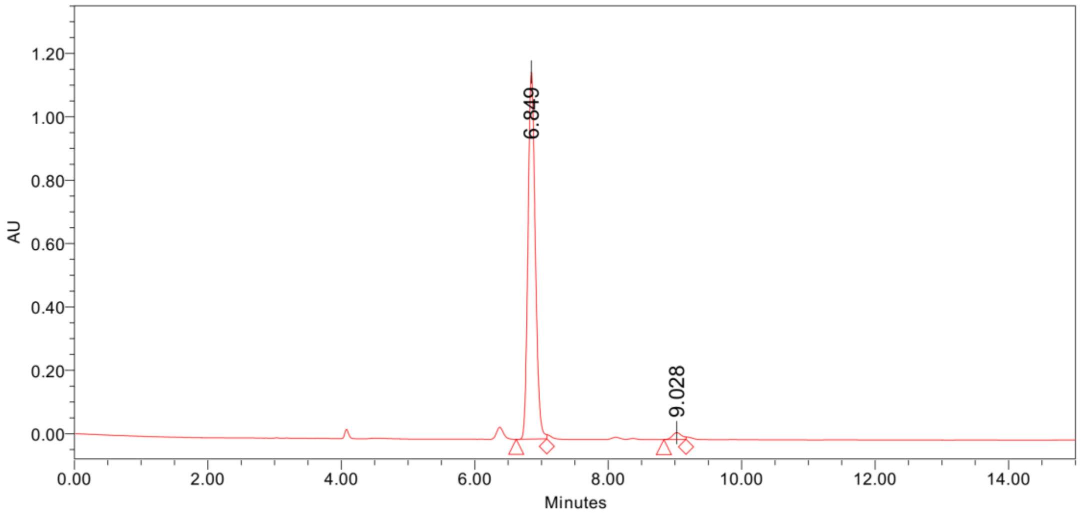

Peak Results

	Name	RT	Area	Height	% Area
1		6.849	8762743	1159239	97.69
2		9.028	207308	21236	2.31

Supplementary Figure 274. HPLC Spectra of **62** obtained from (*R*,*S*)-**L1**.

Auto-Scaled Chromatogram

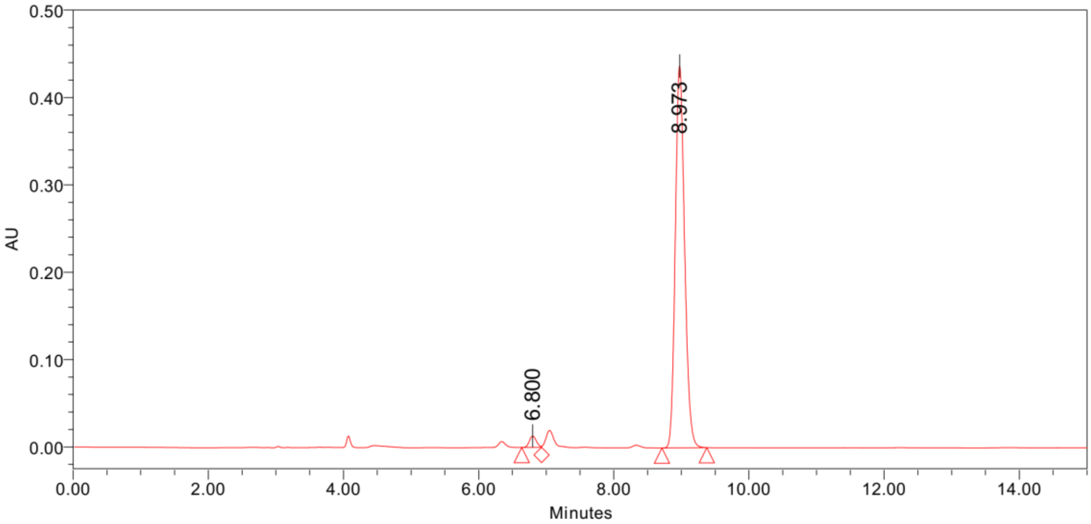

Peak Results

	Name	RT	Area	Height	% Area
1		6.800	94067	13104	2.20
2		8.973	4175223	437161	97.80

Supplementary Figure 275. HPLC Spectra of **62** obtained from (*S*,*R*)-**L1**.

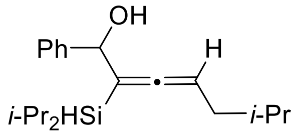

Figure 4, entry 63

(R,S)-**L1**: 96% ee; (S,R)-**L1**: 94% ee

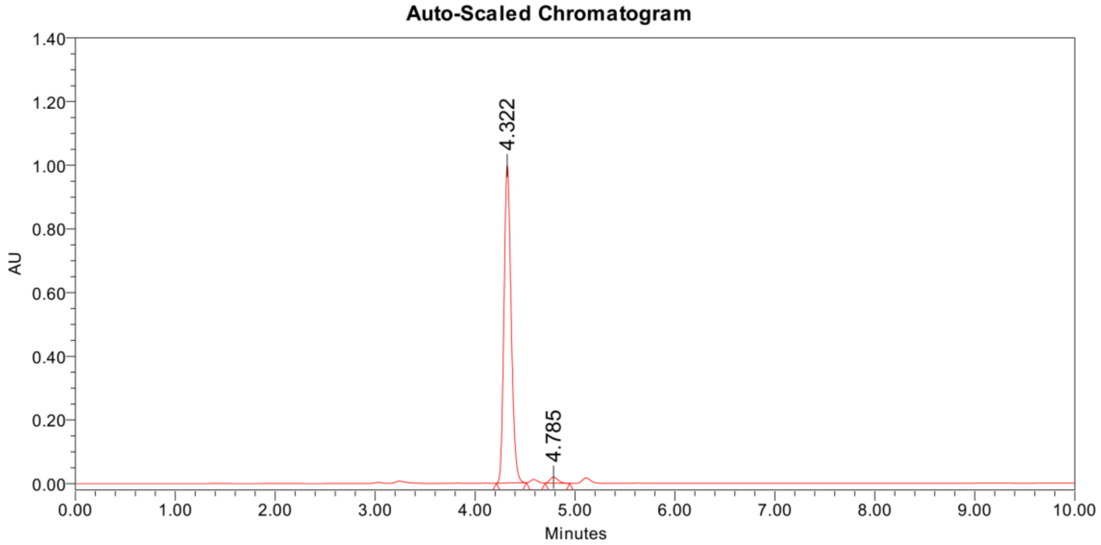

Peak Results

	Name	RT	Area	Height	% Area
1		4.322	4973104	997744	97.96
2		4.785	103519	19007	2.04

Supplementary Figure 276. HPLC Spectra of **63** obtained from (R,S)-**L1**.

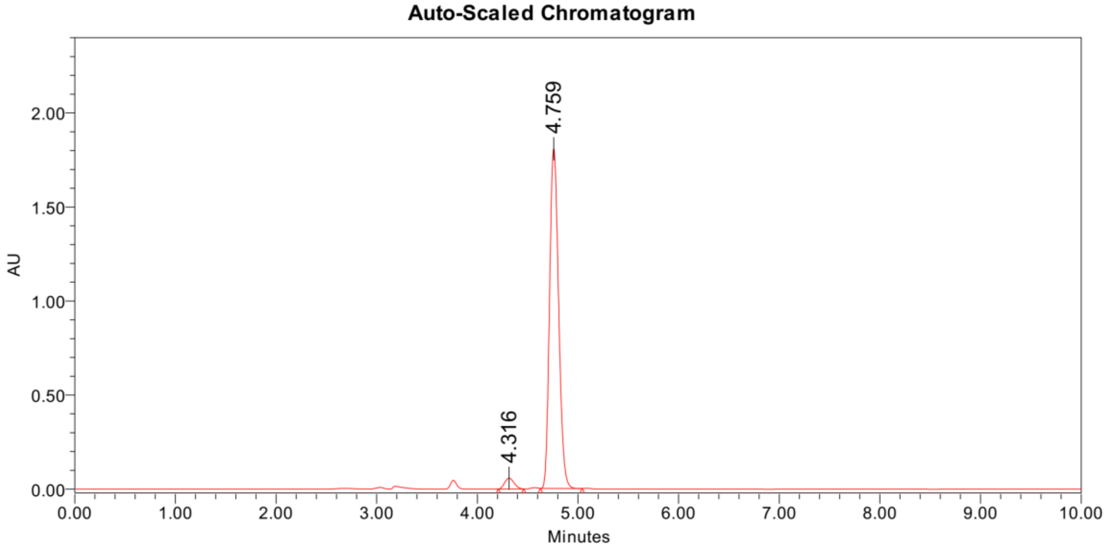

Peak Results

	Name	RT	Area	Height	% Area
1		4.316	370388	57016	3.19
2		4.759	11233966	1806220	96.81

Supplementary Figure 277. HPLC Spectra of **63** obtained from (S,R)-**L1**.

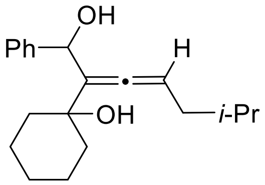

Figure 4, entry 64

(*R*,*S*)-**L1**: 97% ee; (*S*,*R*)-**L1**: 97% ee

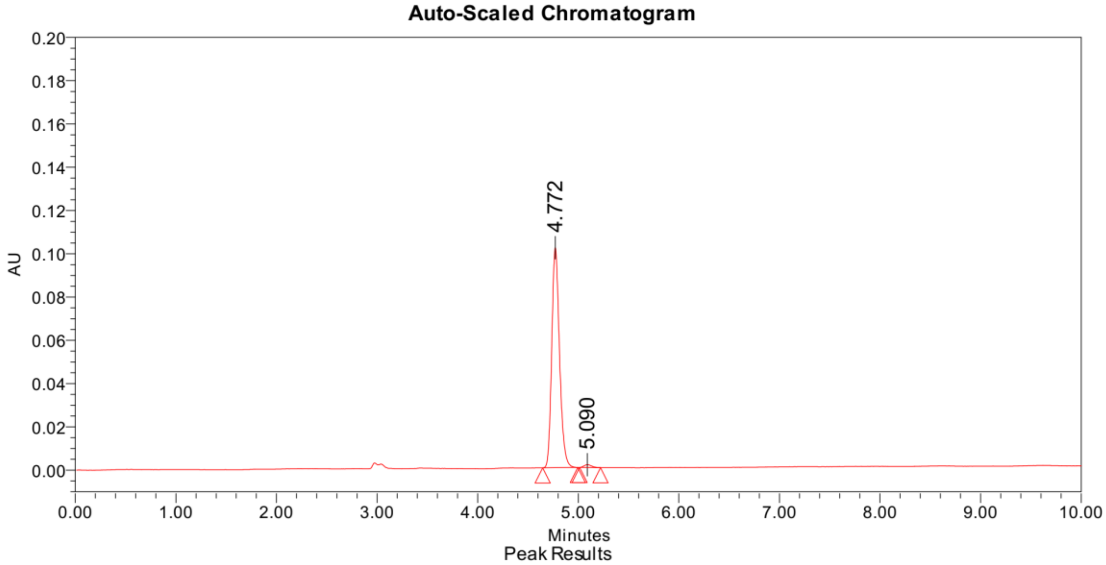

Peak Results

	Name	RT	Area	Height	% Area
1		4.772	547074	101709	98.76
2		5.090	6868	1297	1.24

Supplementary Figure 278. HPLC Spectra of **64** obtained from (*R*,*S*)-**L1**.

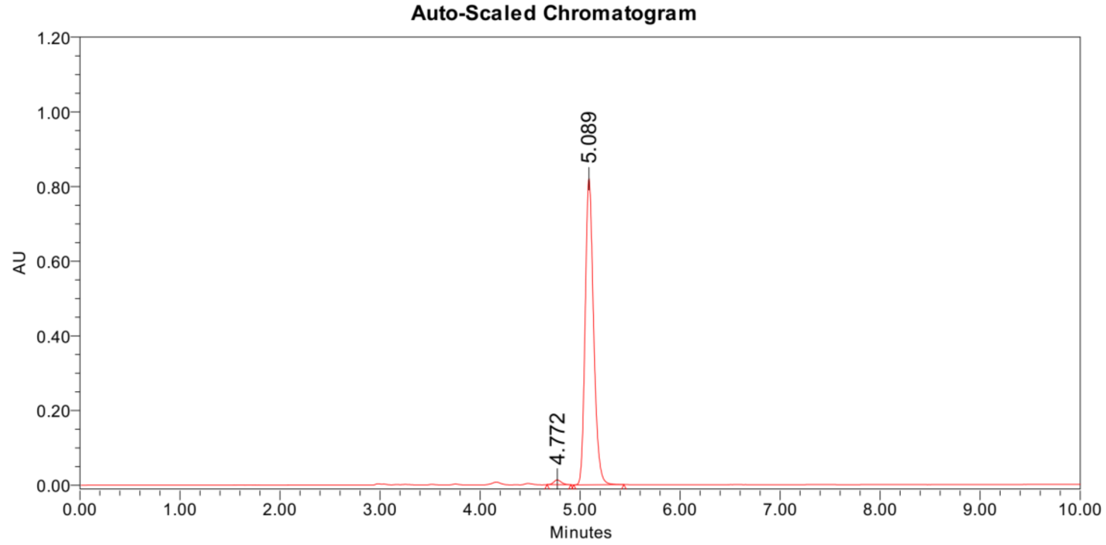

Peak Results

	Name	RT	Area	Height	% Area
1		4.772	66496	12677	1.36
2		5.089	4807922	820040	98.64

Supplementary Figure 279. HPLC Spectra of **64** obtained from (*S*,*R*)-**L1**.

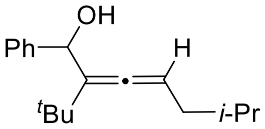

Figure 4, entry 65
(*R*,*S*)-**L1**: 80% ee; (*S*,*R*)-**L1**: 78% ee

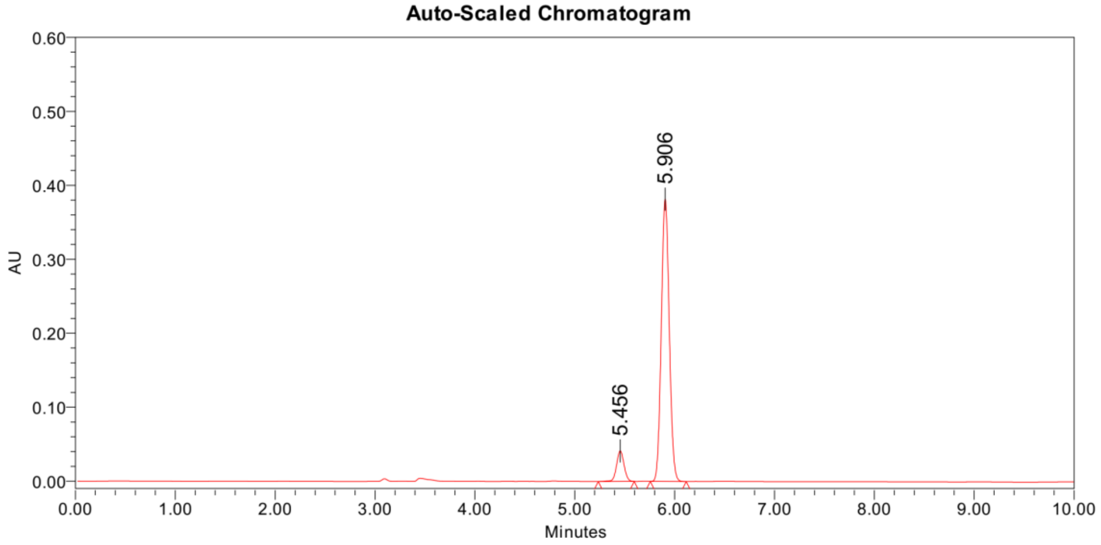

Peak Results

	Name	RT	Area	Height	% Area
1		5.456	217068	41370	9.30
2		5.906	2116433	381756	90.70

Supplementary Figure 280. HPLC Spectra of **65** obtained from (*R*,*S*)-**L1**.

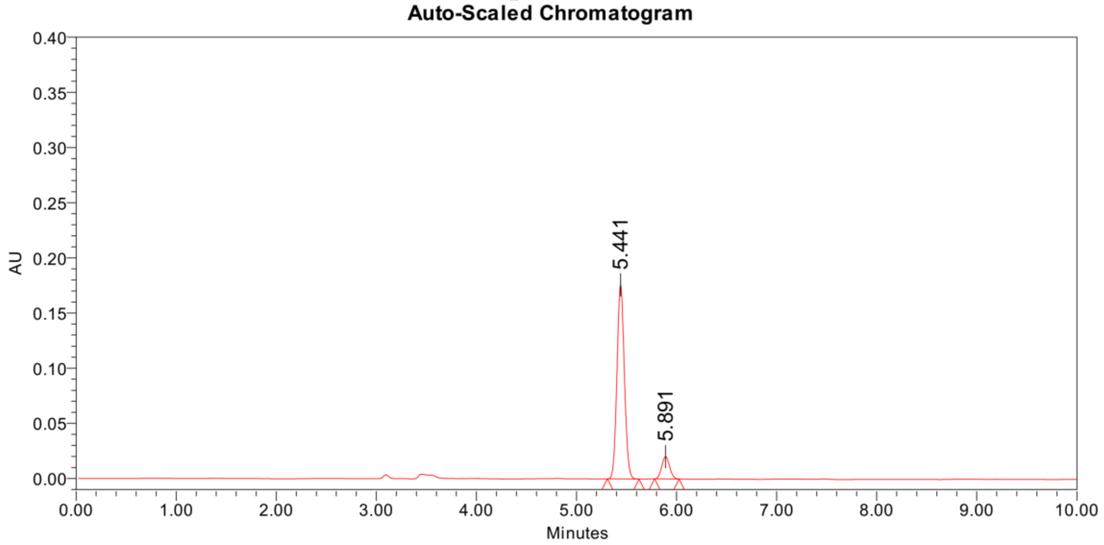

Peak Results

	Name	RT	Area	Height	% Area
1		5.441	907320	175926	89.12
2		5.891	110733	20208	10.88

Supplementary Figure 281. HPLC Spectra of **65** obtained from (*S*,*R*)-**L1**.

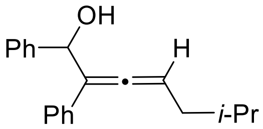

Figure 4, entry 66

(*R*,*S*)-**L1**: 98% ee; (*S*,*R*)-**L1**: 98% ee

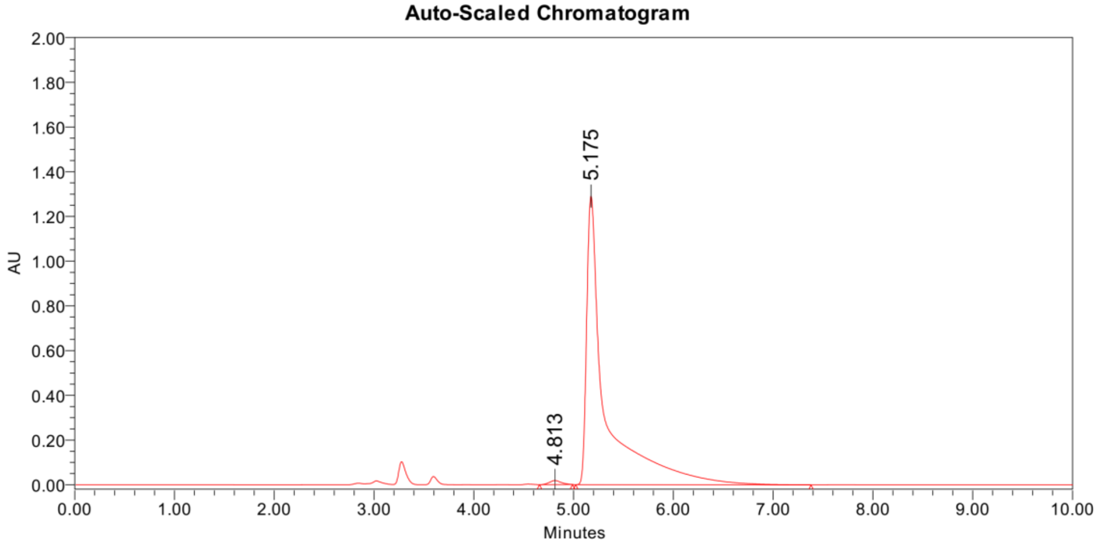

Peak Results

	Name	RT	Area	Height	% Area
1		4.813	147557	18227	0.86
2		5.175	16914364	1290822	99.14

Supplementary Figure 282. HPLC Spectra of **66** obtained from (*R*,*S*)-**L1**.

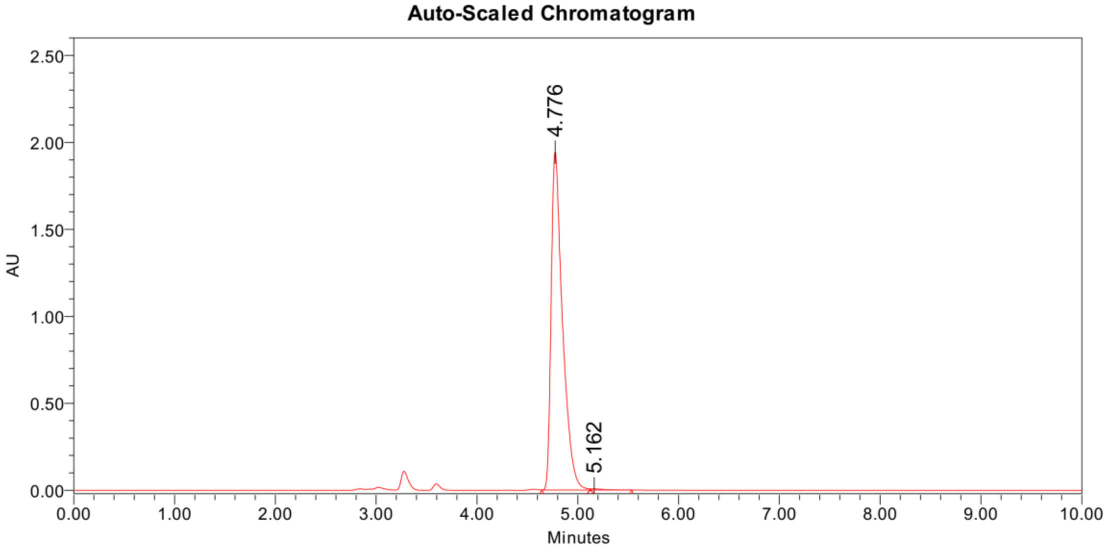

Peak Results

	Name	RT	Area	Height	% Area
1		4.776	15152840	1943898	99.62
2		5.162	58420	6788	0.38

Supplementary Figure 283. HPLC Spectra of **66** obtained from (*S*,*R*)-**L1**.

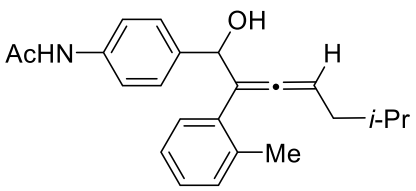

Scheme 2, entry 67
(*R,S*)-**L1**: 96% ee; (*S,R*)-**L1**: 95% ee

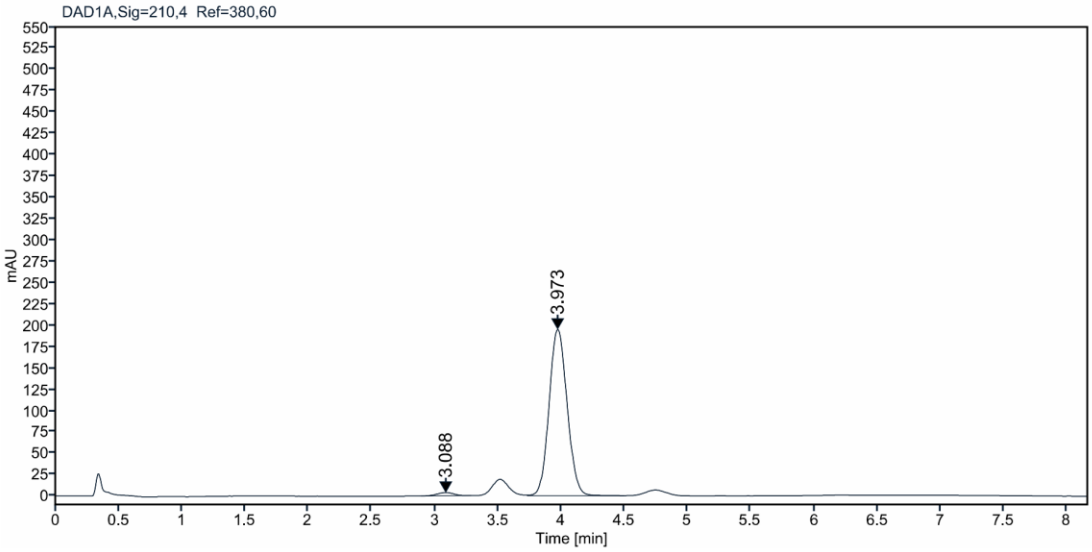

Signal: DAD1A,Sig=210,4 Ref=380,60

RT [min]	Type	Width (50%)	Area	Height	Area%
3.088	BB		36.4851	3.893	1.8489
3.973	VV		1936.8117	194.966	98.1511
		Sum	1973.2968		

Supplementary Figure 284. SFC Spectra of **67** obtained from (*R,S*)-**L1**.

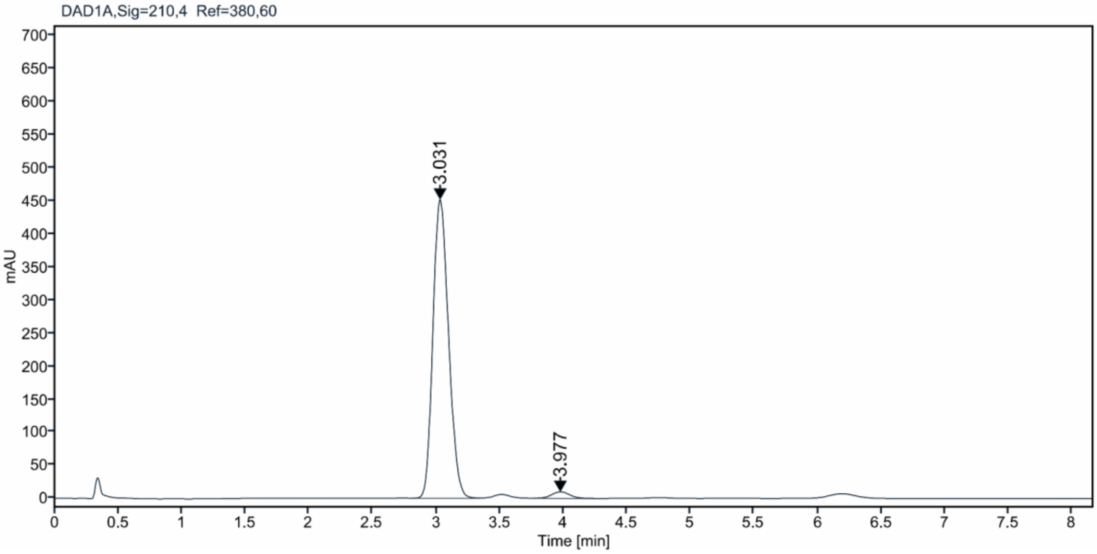

Signal: DAD1A,Sig=210,4 Ref=380,60

RT [min]	Type	Width (50%)	Area	Height	Area%
3.031	BV		3852.9361	452.160	97.6103
3.977	BB		94.3262	9.641	2.3897
		Sum	3947.2622		

Supplementary Figure 285. SFC Spectra of **67** obtained from (*S,R*)-**L1**.

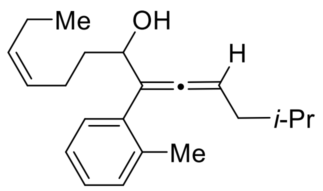

Figure 4, entry 68
(*R*,*S*)-**L1**: 93% ee; (*S*,*R*)-**L1**: 95% ee

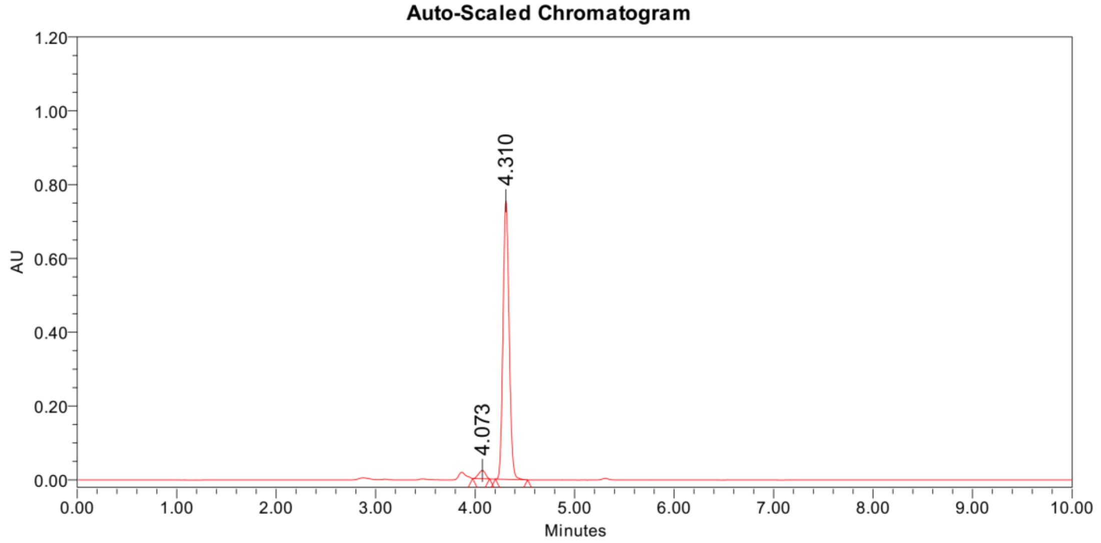

Peak Results

	Name	RT	Area	Height	% Area
1		4.073	106887	22501	3.22
2		4.310	3209338	754873	96.78

Supplementary Figure 286. HPLC Spectra of **68** obtained from (*R*,*S*)-**L1**.

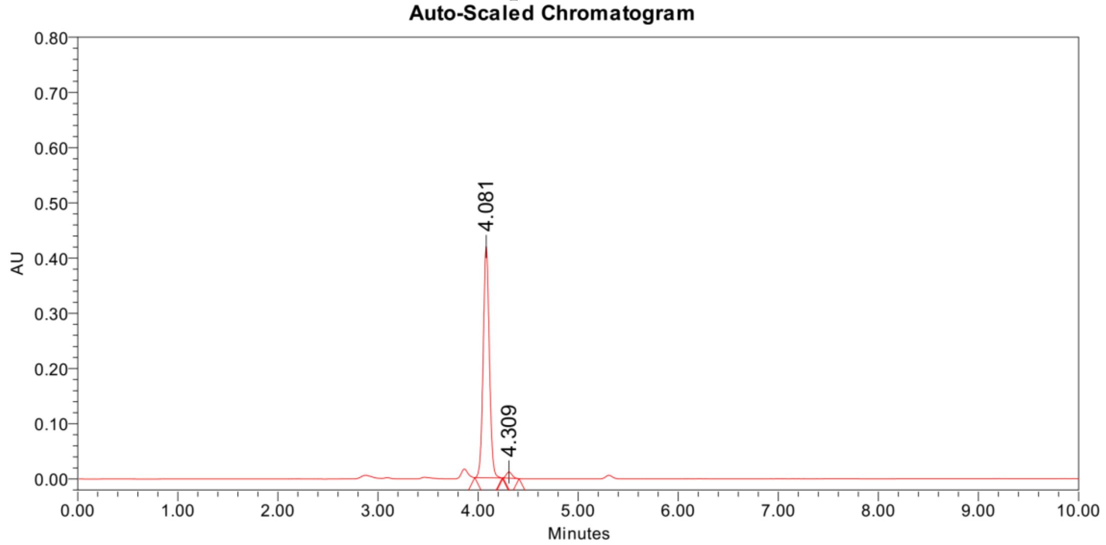

Peak Results

	Name	RT	Area	Height	% Area
1		4.081	1791550	419035	97.63
2		4.309	43574	11102	2.37

Supplementary Figure 287. HPLC Spectra of **68** obtained from (*S*,*R*)-**L1**.

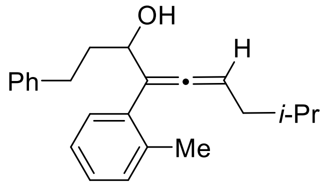

Figure 4, entry 69
(*R*,*S*)-**L1**: 94% ee; (*S*,*R*)-**L1**: 94% ee

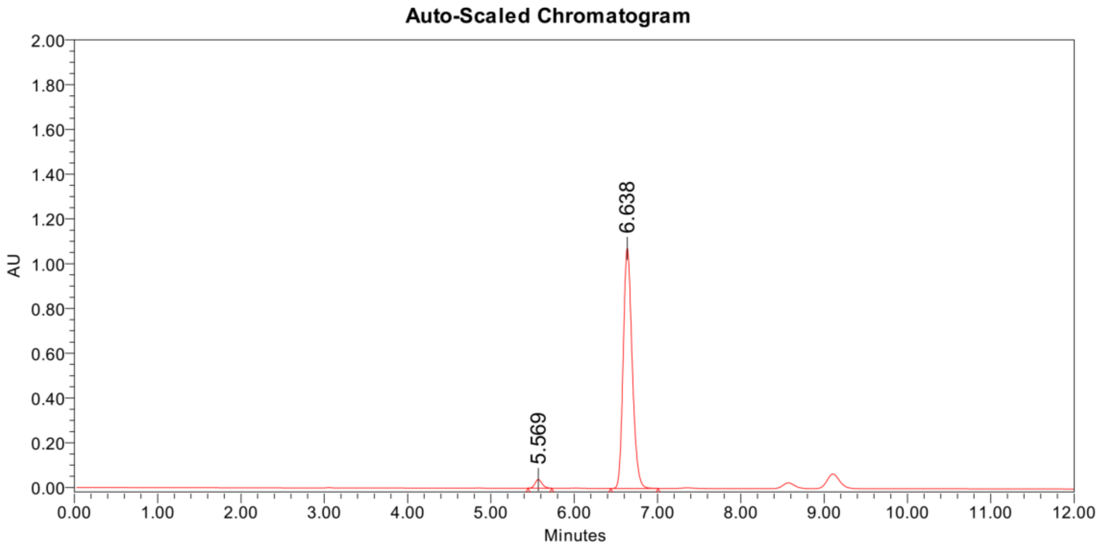

Peak Results

	Name	RT	Area	Height	% Area
1		5.569	230162	38815	2.80
2		6.638	7998301	1073022	97.20

Supplementary Figure 288. HPLC Spectra of **69** obtained from (*R*,*S*)-**L1**.

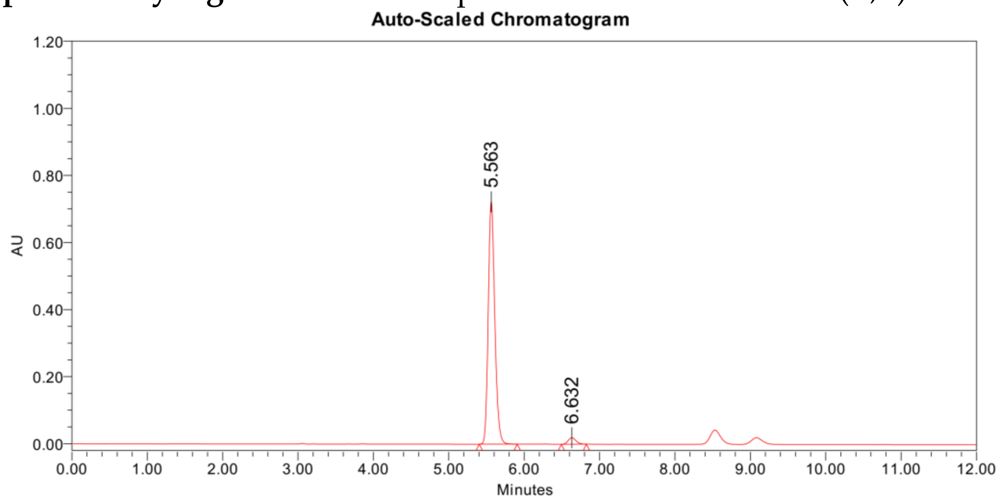

Peak Results

	Name	RT	Area	Height	% Area
1		5.563	4352879	722381	96.93
2		6.632	137832	19339	3.07

Supplementary Figure 289. HPLC Spectra of **69** obtained from (*S*,*R*)-**L1**.

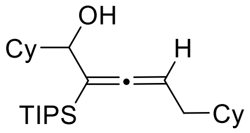

Figure 5a, entry 71

(*R*,*S*)-**L1**: 97% ee; (*S*,*R*)-**L1**: 96% ee

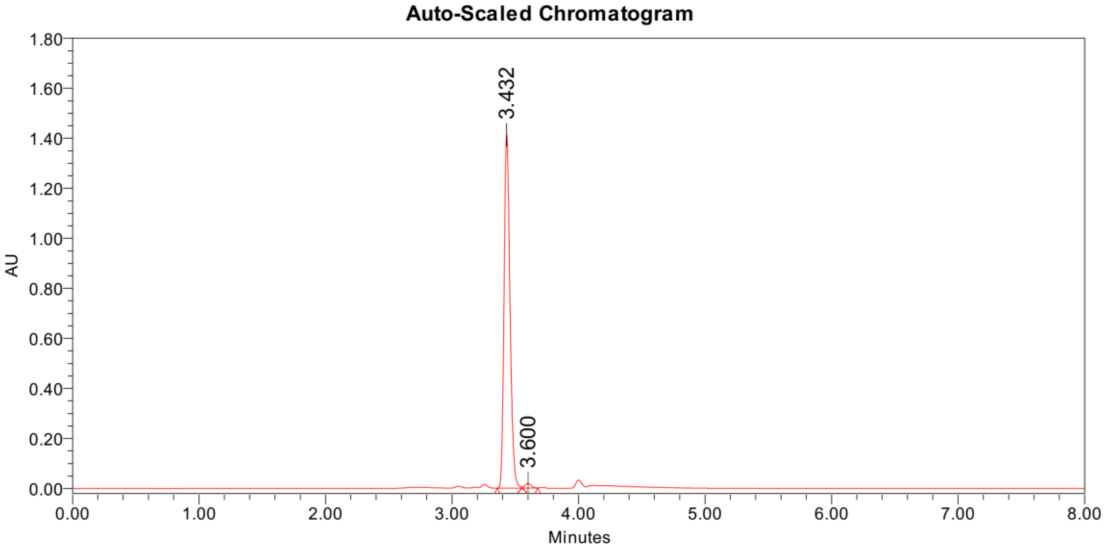

Peak Results

	Name	RT	Area	Height	% Area
1		3.432	4584421	1413372	98.65
2		3.600	62939	18992	1.35

Supplementary Figure 290. HPLC Spectra of **71** obtained from (*R*,*S*)-**L1**.

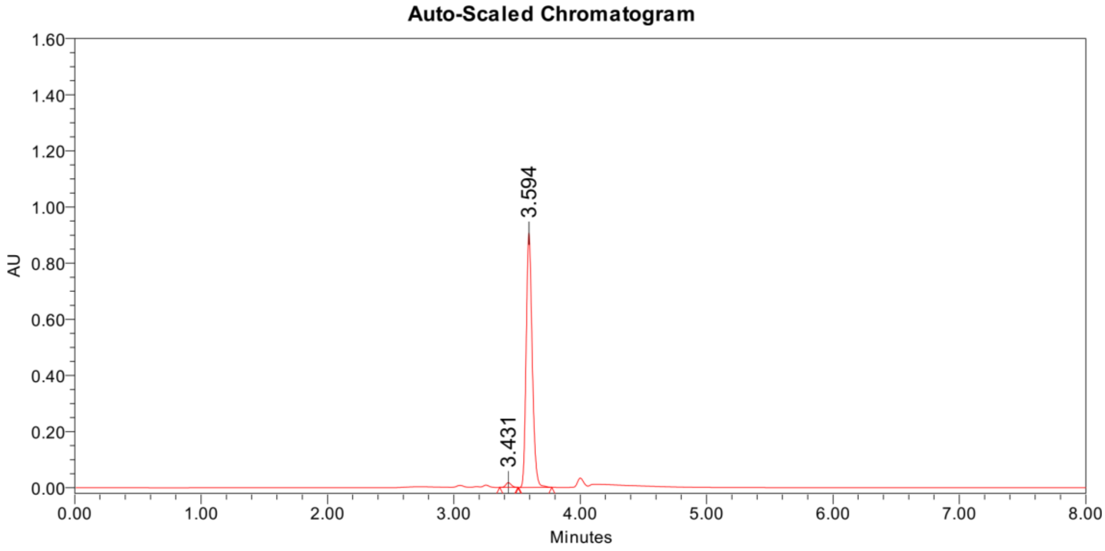

Peak Results

	Name	RT	Area	Height	% Area
1		3.431	52077	17030	1.70
2		3.594	3011333	906575	98.30

Supplementary Figure 291. HPLC Spectra of **71** obtained from (*S*,*R*)-**L1**.

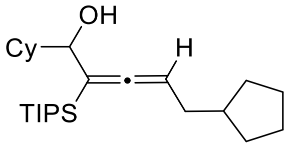

Figure 5a, entry 72

(*R*,*S*)-**L1**: 97% ee; (*S*,*R*)-**L1**: 98% ee

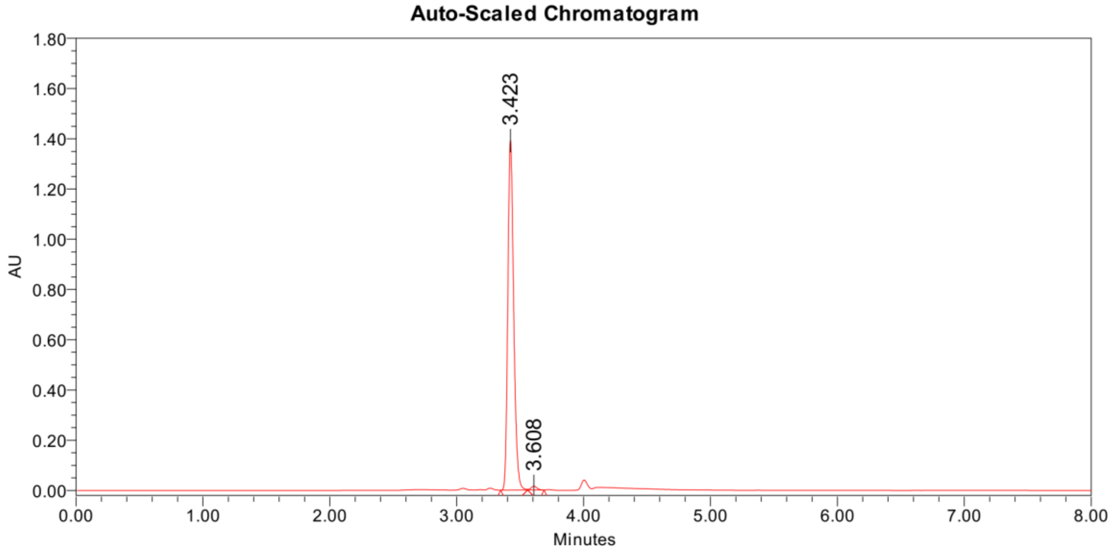

Peak Results

	Name	RT	Area	Height	% Area
1		3.423	4489848	1392862	98.88
2		3.608	50732	15482	1.12

Supplementary Figure 292. HPLC Spectra of **72** obtained from (*R*,*S*)-**L1**.

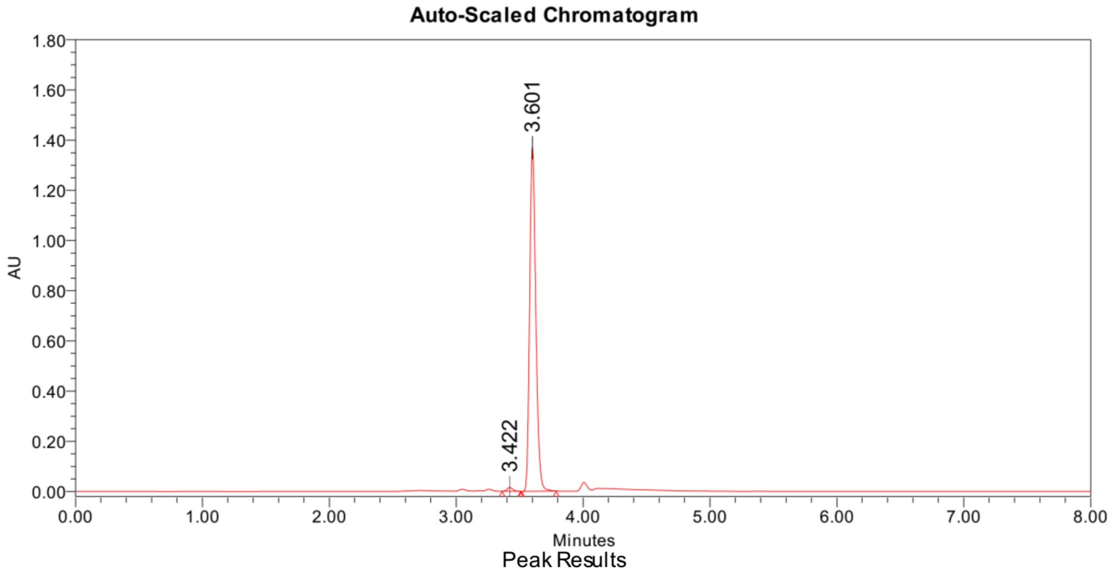

Peak Results

	Name	RT	Area	Height	% Area
1		3.422	45631	14853	0.98
2		3.601	4623912	1371335	99.02

Supplementary Figure 293. HPLC Spectra of **72** obtained from (*S*,*R*)-**L1**.

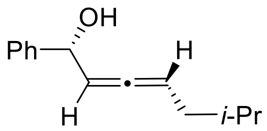

Figure 5c, 73

Auto-Scaled Chromatogram

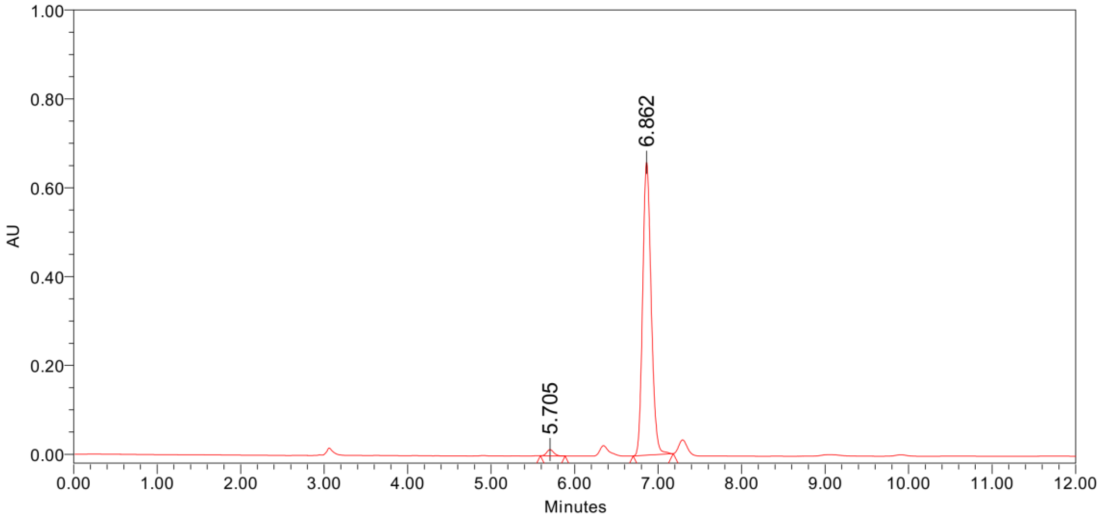

Peak Results

	Name	RT	Area	Height	% Area
1		5.705	81074	14081	1.71
2		6.862	4649264	659656	98.29

Supplementary Figure 294. HPLC Spectra of **73** obtained from (*R*,*S*)-**L1**.

Auto-Scaled Chromatogram

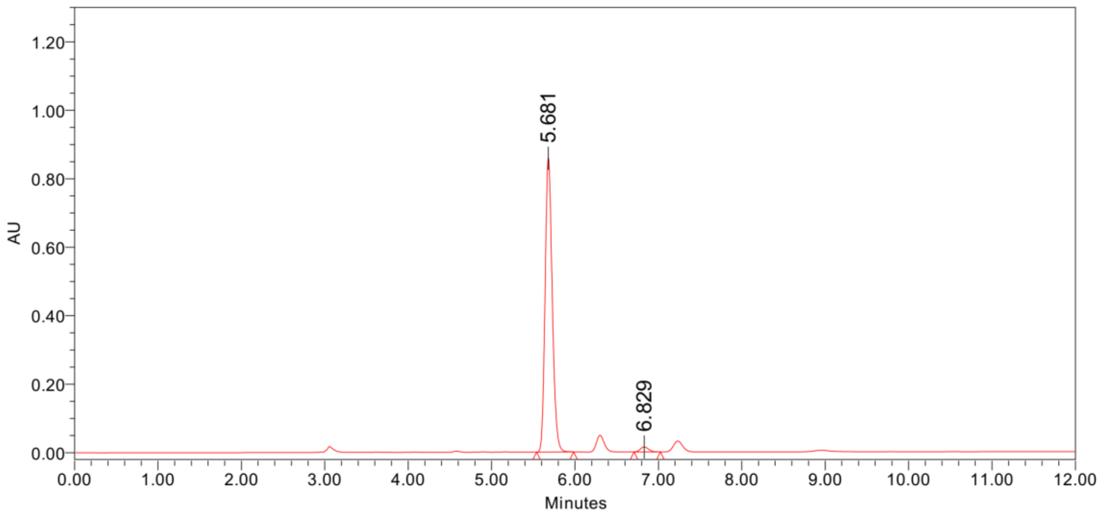

Peak Results

	Name	RT	Area	Height	% Area
1		5.681	5101740	858982	98.08
2		6.829	100009	14603	1.92

Supplementary Figure 295. HPLC Spectra of **73** obtained from (*S*,*R*)-**L1**.

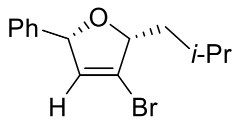

Figure 5c, 74

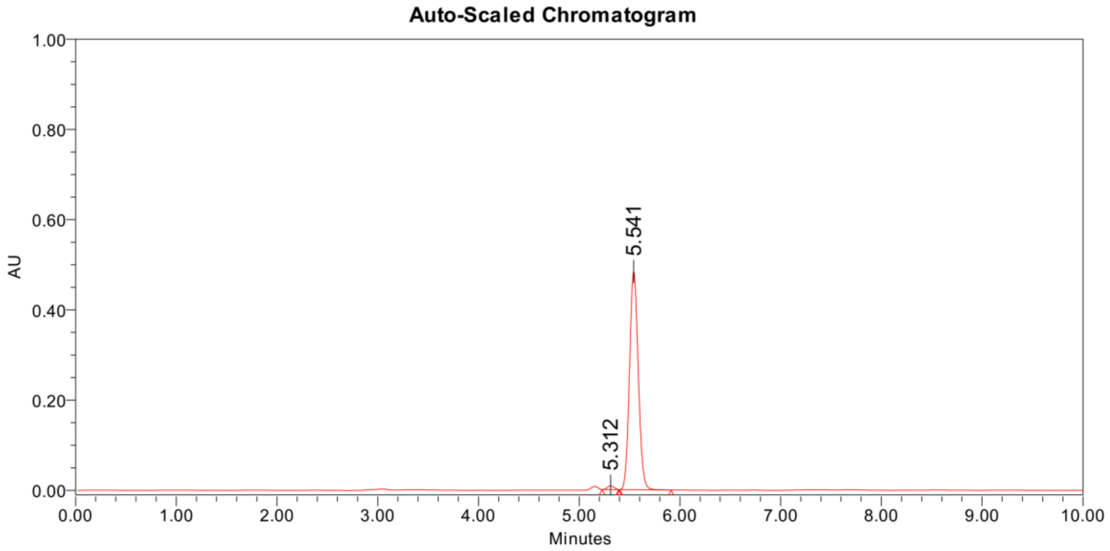

Peak Results

	Name	RT	Area	Height	% Area
1		5.312	37977	7976	1.29
2		5.541	2916164	483787	98.71

Supplementary Figure 296. HPLC Spectra of **74** obtained from (*R*,*S*)-**L1**.

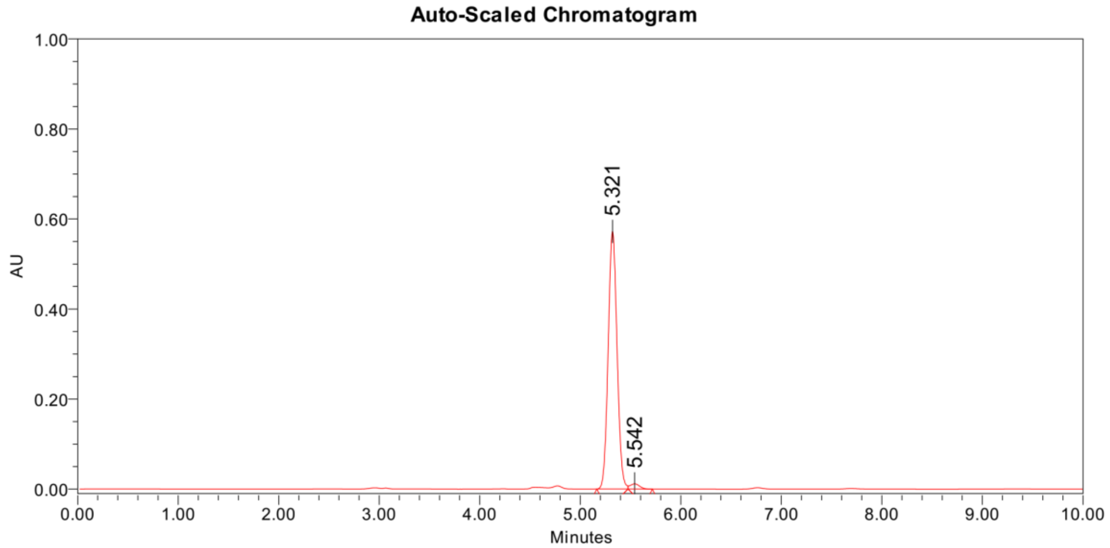

Peak Results

	Name	RT	Area	Height	% Area
1		5.321	3508285	572384	97.88
2		5.542	76141	11564	2.12

Supplementary Figure 297. HPLC Spectra of **74** obtained from (*S*,*R*)-**L1**.

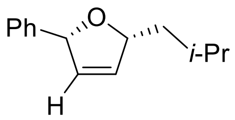

Figure 5c, 75

Auto-Scaled Chromatogram

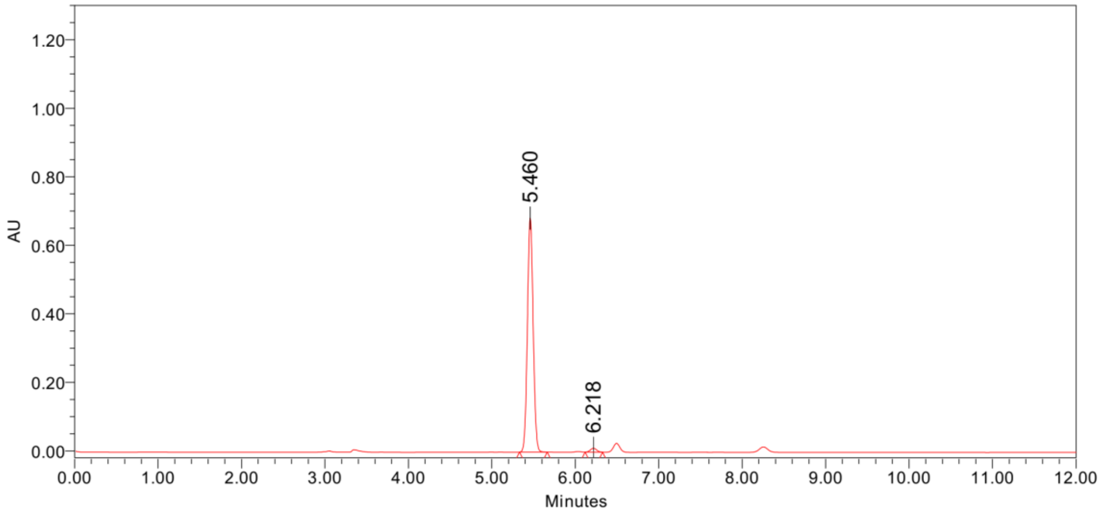

Peak Results

	Name	RT	Area	Height	% Area
1		5.460	3258859	683227	98.23
2		6.218	58627	11229	1.77

Supplementary Figure 298. HPLC Spectra of **75** obtained from (*R*,*S*)-**L1**.

Auto-Scaled Chromatogram

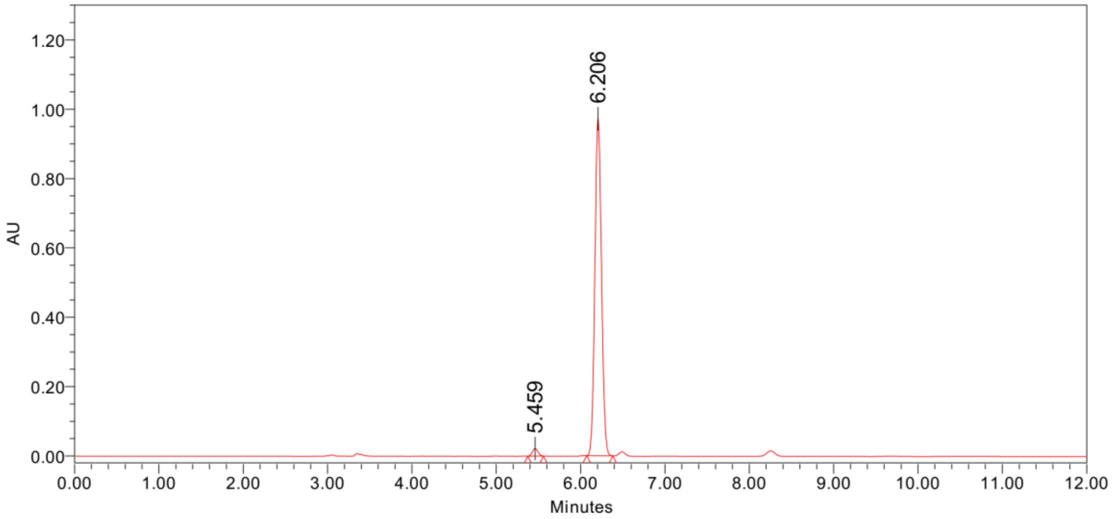

Peak Results

	Name	RT	Area	Height	% Area
1		5.459	100916	21905	1.86
2		6.206	5310553	972431	98.14

Supplementary Figure 299. HPLC Spectra of **75** obtained from (*S*,*R*)-**L1**.

II Supplementary References

(1) Nolin, K. A.; Ahn, R. W.; Kobayashi, Y.; Kennedy-Smith, J. J.; Toste, F. D.; Enantioselective Reduction of Ketones and Imines Catalyzed by (CN-Box)Re^{V}–Oxo Complexes. *Chem. Eur. J.* **2010**, *16*, 9555.

(2) Bai, Z.; Zhang, H.; Wang, H.; Yu, H.; Chen, G.; He, G.; Enantioselective Alkylamination of Unactivated Alkenes under Copper Catalysis. *J. Am. Chem. Soc.* **2021**, *143*, 1195.

(3) Cheng, J.-K.; Loh, T.-P.; Copper- and Cobalt-Catalyzed Direct Coupling of sp^3 α-Carbon of Alcohols with Alkenes and Hydroperoxides. *J. Am. Chem. Soc.* **2015**, *137*, 42.

(4) Li, C.; Wang, J.; Barton, L. M.; Yu, S.; Tian, M.; Peters, D. S.; Kumar, M.; Yu, A. W.; Johnson, K. A.; Chatterjee, A. K.; Yan, M.; Baran, P. S.; Decarboxylative Borylation. *Science* **2017**, *356*, 1045.

(5) Xie, Q.; Dong, G.; Programmable Ether Synthesis Enabled by Oxa-Matteson Reaction. *J. Am. Chem. Soc.* **2022**, *144*, 8498.

(6) Cismesia, M. A.; Yonn, T. P.; Characterizing Chain Processes in Visible Light Photoredox Catalysis. *Chem. Sci.* **2015**, *6*, 5426.

(7) Dolomanov, O. V.; Bourhis, L. J.; Gildea, R. J.; Howard, J. A. K.; Puschmann, H., OLEX2: a Complete Structure Solution, Refinement, and Analysis Program. *J. Appl. Cryst.* **2009,** *42*, 339.

(8) Sheldrick, G., SHELXT - Integrated Space-Group and Crystal-Structure Determination. *Acta Cryst. A* **2015,** *71*, 3.

(9) Sheldrick, G., Crystal Structure Refinement with SHELXL. *Acta Cryst. C* **2015,** *71*, 3.
